# Model-Based Analysis of SARS-CoV-2 Infections, Hospitalization and Outcome in Germany, the Federal States and Districts

**DOI:** 10.3390/v14102114

**Published:** 2022-09-24

**Authors:** Christiane Dings, Katharina Martha Götz, Katharina Och, Iryna Sihinevich, Quirin Werthner, Sigrun Smola, Marc Bliem, Felix Mahfoud, Thomas Volk, Sascha Kreuer, Jürgen Rissland, Dominik Selzer, Thorsten Lehr

**Affiliations:** 1 Department of Clinical Pharmacy, Saarland University, 66123 Saarbrücken, Germany; 2 Institute of Virology, Saarland University Medical Center, 66421 Homburg, Germany; 3 Helmholtz Institute for Pharmaceutical Research Saarland (HIPS), Helmholtz Centre for Infection Research (HZI), 66123 Saarbrücken, Germany; 4 CompuGroup Medical (CGM), 56070 Koblenz, Germany; 5 Department of Internal Medicine III (Cardiology, Angiology, Intensive Care Medicine), Saarland University Medical Center and Saarland University Faculty of Medicine, 66421 Homburg, Germany; 6 Institute for Medical Engineering and Science, Massachusetts Institute of Technology, Cambridge, MA 02139, USA; 7 Department of Anesthesiology, University Hospital of the Saarland, 66421 Homburg, Germany

**Keywords:** coronavirus disease 2019 (COVID-19), SARS-CoV-2, mathematical model, age, sex, testing strategy, variant of concern (VOC), intensive care, non-pharmaceutical interventions

## Abstract

The coronavirus disease 2019 (COVID-19) pandemic challenged many national health care systems, with hospitals reaching capacity limits of intensive care units (ICU). Thus, the estimation of acute local burden of ICUs is critical for appropriate management of health care resources. In this work, we applied non-linear mixed effects modeling to develop an epidemiological SARS-CoV-2 infection model for Germany, with its 16 federal states and 400 districts, that describes infections as well as COVID-19 inpatients, ICU patients with and without mechanical ventilation, recoveries, and fatalities during the first two waves of the pandemic until April 2021. Based on model analyses, covariates influencing the relation between infections and outcomes were explored. Non-pharmaceutical interventions imposed by governments were found to have a major impact on the spreading of SARS-CoV-2. Patient age and sex, the spread of variant B.1.1.7, and the testing strategy (number of tests performed weekly, rate of positive tests) affected the severity and outcome of recorded cases and could reduce the observed unexplained variability between the states. Modeling could reasonably link the discrepancies between fine-grained model simulations of the 400 German districts and the reported number of available ICU beds to coarse-grained COVID-19 patient distribution patterns within German regions.

## 1. Introduction

Severe acute respiratory syndrome coronavirus-2 (SARS-CoV-2) was first identified in December 2019 in Wuhan, China [1], and caused a worldwide pandemic with more than 550,000,000 confirmed cases and 6,300,000 coronavirus disease (COVID-19)-related deaths as of 21 July 2022 [2]. COVID-19 patients with severe courses of disease challenged national health care systems, with hospitals reaching capacity limits of intensive care units (ICUs) [3,4].

Because only a few countries successfully implemented major suppression or local eradication strategies [5], for the vast majority of the world, staying ahead of the pandemic regarding case management with non-pharmaceutical interventions (NPIs), vaccination programs, and health care resource management was and still is of vital importance [6]. The need for vigilance was particularly highlighted as new variants of concern (VOCs) such as lineage B.1.1.7 (Alpha) emerged and quickly spread globally due to a significant advantage in transmissibility compared to wild type [7]. Moreover, infections with some of the VOCs were associated with a higher risk of critical care admission and mortality [8].

The risk for severe courses of COVID-19 differs vastly between individuals, with previous studies identifying age and sex as strong predictors for severity and outcome [9,10,11,12]. As national testing capacities varied throughout the pandemic due to the availability of testing infrastructure [13], further longitudinal predictors might be needed to estimate the prospective number of expected hospitalized patients and fatalities from a number of confirmed SARS-CoV-2 infections. Here, mathematical modeling might be useful to analyze different predictors for COVID-19 hospitalizations and disease outcomes, connecting longitudinal data on confirmed infections and individual risk factors (such as age and sex) as well as systemic case-outcome-related factors (originating from, e.g., regional or national testing strategies) [14,15,16,17].

In this work, we developed a mathematical infectious disease model that describes the first two waves of the pandemic and the emergence of VOC B.1.1.7 in Germany until April 2021. The impact of covariates such as testing strategy, age, sex, and VOC emergence was evaluated to describe SARS-CoV-2 infections and the resulting number of COVID-19 inpatients, ICU patients with and without the need for mechanical ventilation, recoveries, and fatalities for Germany’s 16 federal states and 400 districts. Moreover, the developed model should serve as a foundation which can be adapted to changes in the pandemic, such as the dynamics of infectiousness, the severity of the disease due to new variants, improved therapy options, and advances in vaccination programs.

## 2. Materials and Methods

### 2.1. Epidemiological Data

The local ethics committee of the medical association of the Saarland granted ethical approval for this study (Ärztekammer des Saarlandes, Short title: “CoSim”; Bu 78/20). Data were gathered from several sources: Confirmed SARS-CoV-2 infections as well as COVID-19-related deaths and recoveries were collected at the federal state level from the database of the regional newspaper “Berliner Morgenpost”, which has compiled information from the John Hopkins University CSSE, reports from German authorities, and data from the Robert Koch Institute (RKI) and federal state health authorities since 27 January 2020 [18]. Data on confirmed cases at the district level as well as age- and sex-stratified information on SARS-CoV-2 infections were available from the RKI (age groups 0–4, 5–14, 15–34, 35–59, 60–79, and >80 years) [19].

If information was available, the dataset included the number of current COVID-19 inpatients and daily new inpatients as reported by the health ministries of the 16 federal states [20,21,22,23,24,25,26,27,28,29,30,31,32]. The number of occupied ICU beds with and without mechanical ventilation was available from the German Intensive Care Register DIVI and the RKI [33,34]. A detailed listing of epidemiological data sources can be found in Supplementary Table S1.

The model was informed by prior information on clinical outcomes obtained from the hospital financial information system MetaKIS (CompuGroup Medical (CGM), Koblenz, Germany). This database included a representative cohort of about 10% of all confirmed SARS-CoV-2 cases in German hospitals and provided information about diagnosis (ICD-10 coding), age, sex, and duration of treatment at various hospital wards, as well as duration of mechanical ventilation and outcome on a patient-specific level. In total, the database contained data of 30,723 patients admitted to hospitals between 23 March 2020 and 1 March 2021 with a positive test for infection with SARS-CoV-2 (ICD-10 Code U07.1: COVID-19, virus identified) and released from the hospital as recovered or perished (N = 23,810 and N = 6913, respectively). Recovered inpatients with an unusually short hospital stay of less than a day were excluded from the analysis (N = 1876). Patients with a record of ICU stay for less than one hour were handled as inpatients that only required treatment in the general ward. The analysis of data from the resulting 28,847 COVID-19 patients provided information about hospital admissions per confirmed case, admissions to the ICU, the need for mechanical ventilation, length of stay, and rates of in-hospital death differentiated by general ward, ICU, and need for mechanical ventilation. COVID-19 admission rates and mortality were stratified by sex and age via post hoc analysis, with age groups following the schema of the RKI (0–4, 5–14, 15–34, 35–59, 60–79, and >80 years). The duration of stay was stratified by clinical ward and outcome.

### 2.2. Model Development

Non-linear mixed-effects modeling was applied for the stepwise parametrization and development of a compartmental model consisting of three submodels for (I) infections, (II) hospitalization, and (III) outcomes of SARS-CoV-2 infections. The model was developed for Germany on a federal state level and subsequently applied to describe and analyze the pandemic in the 400 German districts.

Non-linear mixed-effects modeling was performed using the software NONMEM (7.4.3., ICON Development Solutions, Ellicott City, MD, USA). The statistical programming language R 3.6.3 (The R Foundation for Statistical Computing, Vienna, Austria) was used for dataset generation, statistical analysis, and visualization.

Model development was performed in two steps: First, the number of daily infections was described, with dependence on NPIs and other influences causing changes in infectiousness over time. Second, estimated parameters describing daily infections were fixed, and the numbers of daily inpatients, ICU patients, ventilated patients, and fatalities were described. A detailed description of submodel development is provided in the following sections.

### 2.3. Infections

The number of daily infections was described using the classical epidemiological compartment model [35], where the population is transferred through the stages susceptible (S) and infected (I) before counting as a confirmed case (C), as depicted in Equations (1)–(3)

(1) $$\frac{\mathrm{dS}}{\mathrm{dt}}=-\beta \left(t\right)\;*\;\frac{S\left(t\right)}{N}\;*\;I\left(t\right)$$

(2) $$\frac{\mathrm{dI}}{\mathrm{dt}}=\beta \left(t\right)\;*\;\frac{S\left(t\right)}{N}\;*\;I\left(t\right)-\gamma \;*\;I\left(t\right)$$

(3) $$\frac{\mathrm{dC}}{\mathrm{dt}}=y\;*\;I\left(t\right)$$

with N being the number of inhabitants. The transmission rate γ from the infectious stage to confirmed cases was fixed to an infectious period of 7 days, with γ = 1/7 based on 6 days of mean incubation time plus 1 day of lag between showing symptoms and registering a positive test result [36]. The transmission rate β(t) for the transfer from the susceptible to the infectious stage is related to γ and the infectiousness (R_α_) as depicted in Equation (4):

(4) $$\beta \left(t\right)=R_{\alpha }\left(t\right)\;*\;\gamma$$

The infectiousness was described depending on intrinsic local changes implemented at discrete points in time. Furthermore, when VOC B.1.1.7. started to emerge in Germany in the winter of 2020/2021, VOC infections were set to have a 35% increase in infectiousness compared to wild type, as estimated by Graham et al. [7].

### 2.4. Hospitalization and Outcome of COVID-19 Patients in Germany

To describe the number of hospitalized patients and ICU patients with and without mechanical ventilation, the modeled confirmed infections were then split into the four scenarios of interest: patients requiring no inpatient treatment (Q), patients requiring inpatient treatment without treatment at ICU (H), patients requiring ICU treatment without mechanical ventilation (ICU), and patients requiring mechanical ventilation (V). Each group was further split into the outcomes recovery and death. For each scenario and outcome, the mean times until discharge were derived from the clinical database and used as the mean transit time (MTT) for the transition of the population through the respective stages. Here, transit rates were defined as $\left(n+1\right)/\mathrm{MTT}$, with n being the number of additional transit compartments [37]. For this, models with one and two transit compartments and different structural models were tested, resulting in $8\;*\;\left(n+1\right)$ differential equations describing the SARS-CoV-2-positive population transitioning through the disease stages. To compute the total number of hospital inpatients at a certain time point, the numbers of patients in the transit compartments for each ward and outcome were calculated as described in Appendix A Equation (A54).

The covariates age, sex, fraction of cases infected with VOC B.1.1.7, testing strategy, and the number of daily and weekly infections per 100,000 inhabitants were tested for their impact on different model parameters. Model parameters for hospital ward admission and death rates were stratified by age and sex according to rates derived for each age group from the clinical database.

For example, to obtain the fraction of SARS-CoV-2-positive patients requiring inpatient treatment at time t, the fraction of cases with a certain age and sex $p\left(a,s,t\right)$, as reported with the number of cases by the RKI, was multiplied by the age- and sex-stratified risk for hospitalization according to the clinical database $\mathrm{fh}\left(a,s\right)$, resulting in the fraction of new cases requiring hospitalization $h\left(t\right)$ at a certain timepoint (Equation (5)).

(5) $$h\left(t\right)=\sum_{s=\mathrm{Male}}^{s=\mathrm{Female}}\sum_{a=\mathrm{Age}\;0-5}^{a=\mathrm{Age}\;80+}\mathrm{fh}\left(a,s\right)\;*\;p\left(a,s,t\right)$$

ICU, ventilation, and fatality rates were calculated accordingly, as described in Appendix A, Equations (A28), (A29), (A31), (A33) and (A35).

For other covariates, different linear, exponential, and sigmoid Emax models were tested to describe the impact of the covariates on different model parameters [38]. If the analysis of the clinical data and model goodness-of-fit plots revealed the need for rate changes at distinct time points that could not be explained by the previously discussed covariates, rate changes were included in the model with time-dependent sigmoid Emax models.

### 2.5. Model Parametrization and Mixed-Effects Modeling

If possible, model parameters were informed by literature or metrics derived from the clinical database. Missing parameters were estimated using first-order conditional estimation with interaction (FOCEI) [39] implemented in NONMEM. The objective function value (−2 log-likelihood; OFV), precision of parameter estimates reported as relative standard errors (RSE) [38], as well as visual inspection of the goodness-of-fit plots [40] were used as evaluation criteria for model selection.

Model parameters that could not be informed by literature or database analysis were estimated using fixed effects and random effects. For this, unknown changepoints in infectiousness and all hospitalization and outcome model parameters were estimated as fixed effects. Random effects were estimated for local infectiousness for every changepoint and the error models. Here, combined residual error models with additional and proportional errors were used for each modeled outcome (hospital, ICU, ventilated, deaths) except for the cumulative cases, where a combined additional and exponential error was assumed.

For the model describing the number of cases, the changepoints of infectiousness were either fixed to dates of reported changes in federal or local policy regarding NPIs, or they were estimated as fixed effects (see Supplementary Table S3). For the implementation of changepoints, the NONMEM model event time parameter (MTIME) was used. A random effect model was used to describe the differences in change of infectiousness between federal states or districts, assuming a log-normal distribution with a prior of 30% CV.

The development of the infectiousness model was initiated at the beginning of the pandemic in Germany and was updated weekly. Here, previously estimated changepoints and infectiousness were fixed, and only the latest three changepoints and $R\left(t\right)s$ were estimated every week. If the implementation of a new infectiousness changepoint led to a significant improvement in the description of the data (*p* < 0.01, dOFV > 11.345 for 3 degrees of freedom), it was retained in the model. Changepoint starting values were set to be at least 5 days apart from the previous changepoint. For the estimation process, $R\left(t\right)$ values were clamped to a range between 0 and 3. For the simulation of infection trajectories for every state and district, maximum a posteriori estimation was used to estimate individual model parameters. The simulation of infections within the whole German population was accomplished using the population estimates.

For the hospitalization and outcome models, the estimated individual parameters from the infectiousness model were fixed. For the hospitalization and outcome models’ parametrization, only fixed effects were used (see Appendix B Table A1). Here, starting values were set to assure that parameters ranged within reasonable boundaries (e.g., the fraction of patients requiring inpatient treatment would not exceed 1).

## 3. Results

### 3.1. Clinical Database

The clinical database covered approximately 10% of all German COVID-19 inpatients from 139 hospitals and included a total of 28,847 COVID-19 inpatients (53% males) with a median age of 73 years (interquartile range of 57–83 years). A comprehensive summary of the data can be found in Supplementary Table S2. During the study period, 18% (N = 5235) of inpatients required ICU treatment, and 67% (N = 3508) of ICU inpatients required mechanical ventilation. Overall, 24% (N = 6913) of inpatients died. The average time until discharge and time spent on each ward were stratified by outcome (Table 1). The mean time until discharge (recovery or death) was 2.1-fold longer for recovered patients who needed ICU treatment compared with patients treated in the general ward. Patients dying had a shorter duration of inpatient treatment in comparison to recovering inpatients. In particular, patients requiring mechanical ventilation showed a 46% shorter hospital stay compared with recovered patients. On average, inpatients required mechanical ventilation for 28% of their stay until recovery (8 days), whereas patients dying were ventilated for 63% of their hospital stay (9.8 days). Moreover, patients requiring ventilation spent on average 43% (12.7 days) or 68% (10.5 days) of their time as inpatients in the ICU if the outcome was recovery or death, respectively. Patients without the need for mechanical ventilation spent 29% (5.9 days) or 44% (8.8 days) of their hospital stay at the ICU when recovering or dying, respectively.

### 3.2. Model Structure

**Figure 1:**
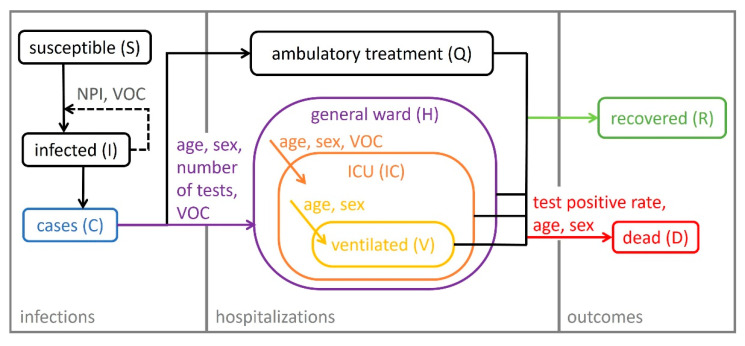
Schematic representation of the epidemiological compartment model. Solid arrows indicate the flow of individuals between compartments during the infection/disease process. Covariates influencing the flow rates are assigned to the respective arrows. Dashed arrows indicate the influence of a compartment value on the rates. NPI: non-pharmaceutical interventions, VOC: fraction of cases infected with the variant of concern B.1.1.7, number of tests: number of weekly performed PCR tests in Germany.

**Figure 2:**
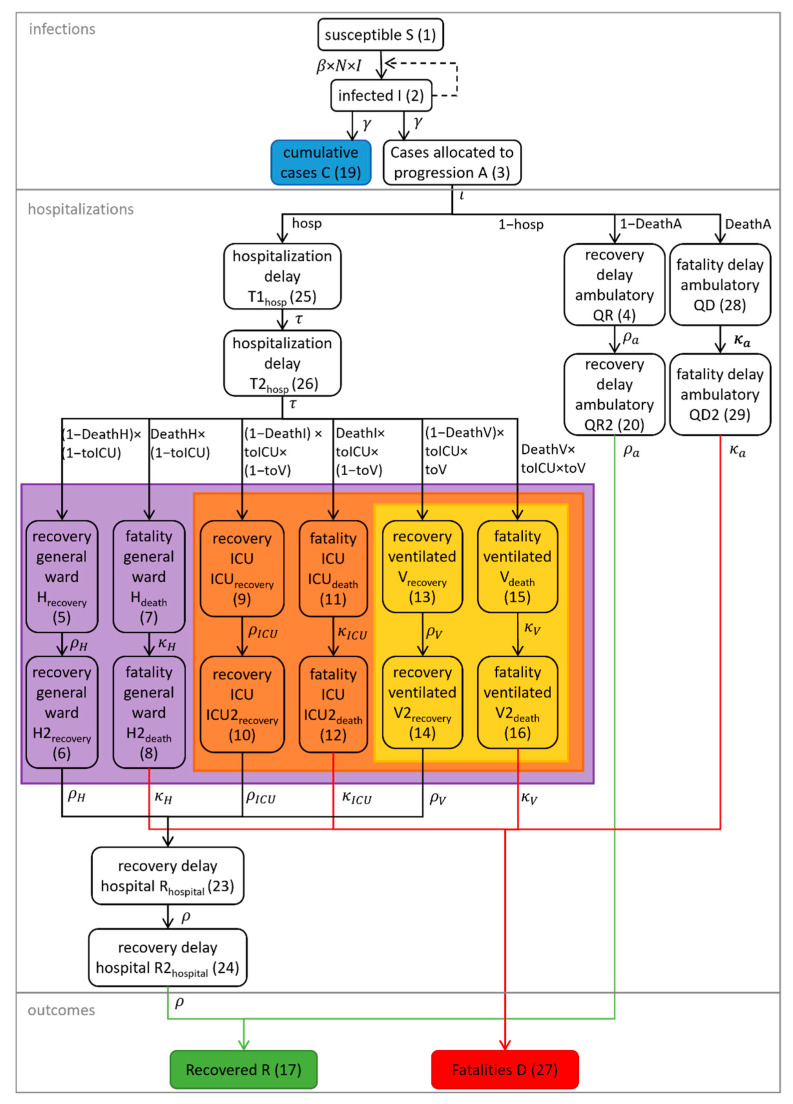
Detailed depiction of the compartmental model including flow rate constants. Numbers represent the compartment numbers used in the NONMEM model file. Not depicted are compartment numbers 18, 21, and 22, which were used for the computation of daily deaths, daily hospitalizations, and cumulative ICU patients. The violet, orange, and yellow areas represent the compartments used for the calculation of inpatients, ICU patients, and ventilated patients, respectively, according to Equations (A52)–(A54) (see Appendix A).

The course of the COVID-19 pandemic in the 16 federal states and 400 districts of Germany from the beginning of the pandemic (25 February 2020, first verified SARS-CoV-2 infection in Germany leading to an outbreak with untraceable chains of infection) until the beginning of the third wave (1 April 2021) was analyzed using a comprehensive epidemiological compartment model. The model consists of 29 ordinary differential equations (ODEs) that describe the population transitioning through seven infection and disease-relevant stages. Figure 1 illustrates a simplified representation with submodels for infections, hospitalizations, and outcomes. Figure 2 shows a comprehensive overview of all model compartments and references to the ODEs listed in Appendix A. The NONMEM model files (infection-only and full model) can be found in the Supplementary Materials.

**Figure 3:**
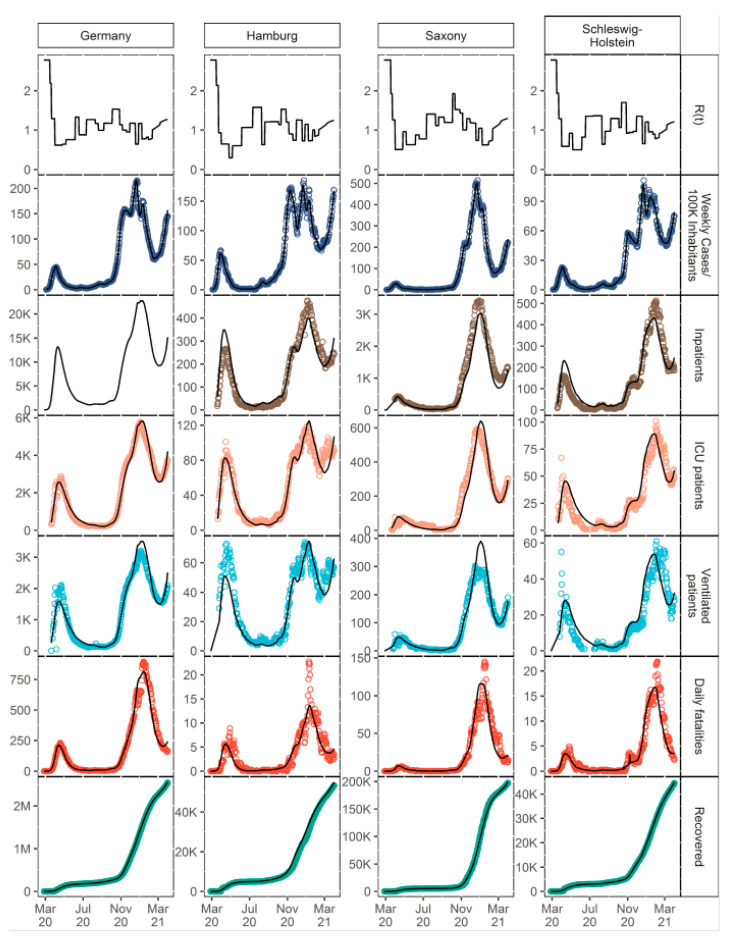
Descriptive performance plots for Germany and three selected federal states. Points: observations, lines: individual model predictions. Information about the total number of inpatients was not available for Germany in total.

As in classical epidemiological SIR compartment models [35], the population is assigned to one of the three stages: susceptible (S), infected (I), and recovered (R). In the presented model, three new stages were implemented: quarantined patients in an ambulatory setting (Q), inpatients, and disease-related fatalities (D). Inpatients were divided into patients in a general ward (H), ICU patients (IC), and patients who need mechanical ventilation during any period of their ICU stay (V). Moreover, inpatients could recover or die at each of these stages. Age, sex, the fraction of patients infected with VOC B.1.1.7, the number of weekly PCR tests, and test positivity rate were identified as significant covariates influencing the rate and distribution of new inpatients as well as outcomes. Model parameters which were not informed by metrics derived from the clinical database but estimated as fixed effects can be found in Table A1 of Appendix B. The model described the number of hospitalizations (stratified by hospital ward and need for mechanical ventilation), recoveries, and COVID-19-related deaths for Germany and the 16 federal states very well. Figure 3 shows the time courses of all relevant observations and model predictions for Germany and three selected federal states, and Supplementary Figure S1 shows all federal states. Further details on the submodel structures are described in the following sections.

### 3.3. Infectiousness

**Figure 4:**
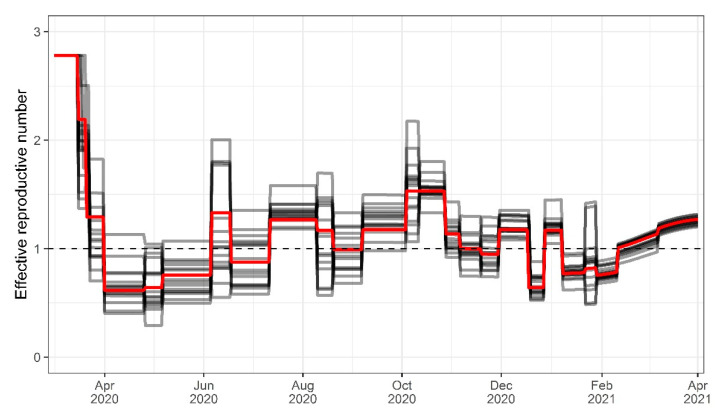
Changes in effective reproductive number over time for Germany (red line) and the federal states (grey lines).

Cases were described by a stepwise estimation of the infectiousness depending on NPIs, estimated infectiousness changepoints, and the fraction of cases infected with VOC B.1.1.7. In total, 24 significant infectiousness changepoints could be observed or estimated over the 48 weeks of investigation, with a mean period of 15.5 days between changes (minimum 6 days, maximum 30 days), as depicted in Figure 4.

For 15 out of 24 changepoints, variation in infectiousness could be attributed to changes in NPI policies (e.g., inception or lifting of mandates by the federal or state governments). Seven changepoints could be linked to other causes, such as local superspreading events during periods with low daily confirmed cases or raised awareness and voluntary contact reductions within the population at the beginning of the pandemic. For two changepoints (4 October 2020 and 30 November 2020), no exogenous cause for the significant increase in infectiousness could be identified. In our estimations, school closures at the beginning of the pandemic led to an average reduction of infectiousness of approximately 31%. Furthermore, curfew or contact-restraining orders that were reinforced on average 5 days after school closure led to a further reduction of infectiousness by 42%. With the resolution of the nationwide so-called “lockdown light” on 28 October 2020 (including the shutdown of restaurants, bars, and leisure and sports facilities, as well as limitations for retail stores and contact restrictions), the infectiousness was reduced on average by 28%.

The coefficient of variation (CV) of the infectiousness between the states was on average 17.7% (range 3.9–40.6%). When NPIs were reinforced by federal state governments, the average CV was 23.2% (6.9–40.6%). After the reinforcement of nationwide NPIs, the average inter-state variability was lower (11.2%, range 3.9–29%). All infectiousness changepoints, the resulting effective reproductive numbers (R(t)), and associated events, such as changes in NPI policies, are listed in Supplementary Table S3.

### 3.4. Hospitalization and Outcome of COVID-19 Patients in Germany

In the presented model, infected individuals were allocated to two different paths: (i) a quarantine path for patients in home quarantine (Q) and (ii) a hospital path for inpatients (T). Analysis of our clinical database and modeling outcomes revealed that the hospitalization rate $hosp\left(t\right)$ (Appendix A Equation (A8)) was dependent on the age and sex of the infected individuals, the number of PCR tests (NT) performed weekly in Germany, and the fraction of infections with VOC B.1.1.7. Furthermore, a changepoint could be observed with a noticeable shift in the hospitalization rate. All effects are presented in detail in the following sections.

Inpatients were split into three groups depending on severity of illness: patients only treated in a general ward, patients in an ICU without ventilation, and patients being ventilated. Furthermore, all three groups were stratified per outcome (recovery or death). Hence, for inpatients, six groups of wards and outcomes were defined, with specific times until discharge for each group. All patients recovering enter recovery transit compartments (Appendix A Equations (A48) and (A49)) after their stay in the respective hospital ward before they are counted as recovered (R) to adjust recovery time to the RKI definition (14 days after discharge). In contrast to the recovered patients, inpatient fatalities (D) were counted without delay. The model works under the simplification that recovered patients are immune to reinfection with SARS-CoV-2 wild type and VOC B.1.1.7 [41].

### 3.5. Age and Sex

**Figure 5:**
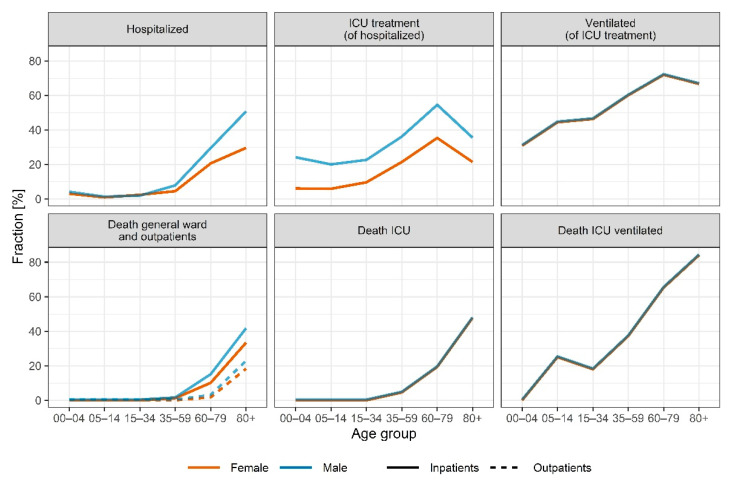
Fractions of confirmed cases hospitalized, treated in an ICU, and ventilated and death rates stratified by age and sex as extracted from the clinical database and data from RKI.

**Figure 6:**
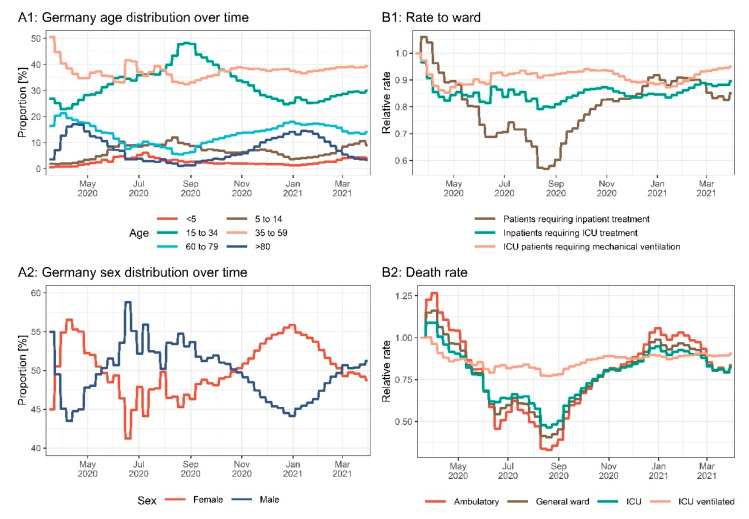
Age (**A1**) and sex (**A2**) distribution of confirmed cases over time. Relative changes in fractions of confirmed cases hospitalized, treated in an ICU, and ventilated (**B1**) as well as death rates (**B2**) resulting from changes in the age and sex distributions of the confirmed cases over time.

Age and sex were significant covariates impacting the severity of disease and outcome (*p* < 0.001). We calculated the fractions of confirmed SARS-CoV-2 cases receiving inpatient treatment by age and sex based on the clinical database and the reported cases from RKI. The fractions of inpatients receiving treatment at the ICU and mechanical ventilation as well as fatality rates stratified by age and sex were calculated based on the clinical database. An overview of the fractions is depicted in Figure 5 and listed in Supplementary Table S4. The risk of hospitalization and death was highest in elderly male patients (50.3% for patients > 80 years), and the risk of inpatients needing intensive care treatment increased with age and was highest for patients aged 60 to 80 (54.1% for male patients). For most age groups, female confirmed SARS-CoV-2 cases had a lower risk of hospitalization compared to male cases (24% to 41% risk ratio). However, for female cases age 15 to 34, a higher risk of hospitalization (females 2.38% vs. males 1.54%, 55% risk ratio) could be observed. Figure 6 depicts the demographic changes of the confirmed cases over time and the resulting changes in fractions of confirmed cases hospitalized, treated in an ICU, and ventilated, as well as death rates.

### 3.6. Variants of Concern

In winter 2020/2021, VOC B.1.1.7. started to emerge in Germany. In our model, the fraction of infections with VOC B.1.1.7 ($VOC\left(t\right)$) was estimated using an exponential growth function (Equation (6)) with a growth rate k *=* 0.072 and an initial fraction of infections with VOC B.1.1.7 ($F_{\mathrm{init}}t$) of 0.2% according to Volz et al. [42]. The initial time ($t_{\mathrm{init}}$) was estimated to fit the model to the fractions reported by the RKI at 17 February 2021 for the preceding 6 weeks [43]:

(6) $$\mathrm{VOC}\left(t\right)=1/(1+\frac{1-F_{\mathrm{init}}}{F_{\mathrm{init}}}{\;*\;e}^{-k\;*\;\left(t-t_{\mathrm{init}}\right)}).$$

VOC B.1.1.7. was first detected on 24 December 2020 in Germany [44]. However, retrospective analysis showed that it had already emerged in November 2020 [25]. Our model estimated 0.02% of infections with VOC B.1.1.7 as of 3 December 2020.

**Figure 7:**
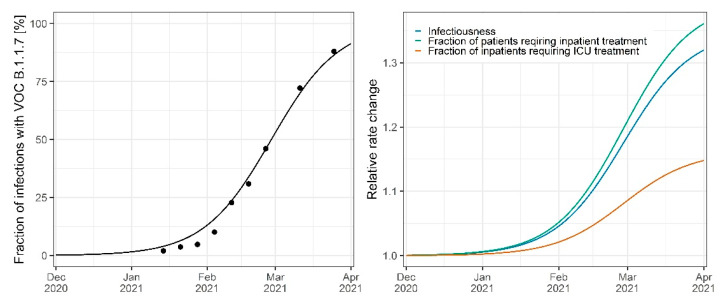
Fraction of infections with VOC B.1.1.7 in Germany (**left**) and impact of VOC B.1.1.7 on the infectiousness, fraction of patients requiring inpatient treatment, and fraction of inpatients requiring ICU treatment (**right**). Points indicate observed fraction of infections in Germany. Lines indicate the model-predicted fraction or rate changes.

Variant spreading and variant-associated infectiousness were fixed in our model to be 35% higher in comparison to wild type as reported by Graham et al. [7] (Figure 7). The stepwise change of infectiousness due to NPIs was estimated for the infectiousness of the wild type $R_{\alpha \mathrm{WT}}$, with $R_{\alpha }\left(t\right)=R_{\alpha \mathrm{WT}}\left(t\right)$ before the emergence of B.1.1.7 at $t_{\mathrm{init}}$ with:

(7) $$R_{\alpha }\left(t\right)=R_{\alpha \mathrm{WT}}\left(t\right)\;*\;\left(1+0.35\;*\;VOC\left(t\right)\right).$$

The impact of VOC B.1.1.7 on disease severity was estimated by fitting VOC-dependent changes in various model rates to the observed inpatient and fatality data from the federal states. As depicted in Figure 7, the fraction of patients requiring inpatient treatment increased by 39.5% (RSE 14.1%, *p* < 0.001), and the fraction of inpatients requiring intensive care treatment increased by 16.2% (RSE 33.1%, *p* < 0.001) for patients infected with VOC B.1.1.7 in comparison to infections with the wild type. An additional change in inpatient death rates due to VOC B.1.1.7 was not significant in our analysis.

### 3.7. Testing Strategy

**Figure 8:**
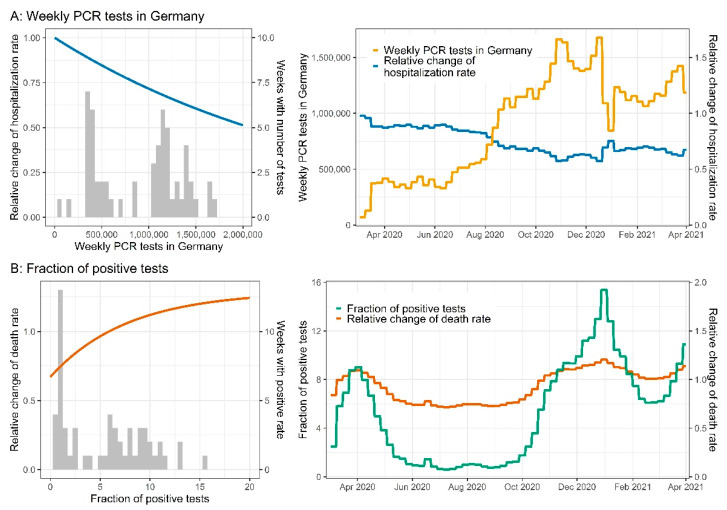
(**A**) Weekly PCR tests in Germany. Blue lines indicate the change in the hospitalization rate vs. the number of tests performed (**left plot**) and time (**right plot**). The histogram represents the number of weeks with the respective number of weekly tests. The yellow line represents the weekly tests vs. time. (**B**) Fraction of positive tests. Orange lines indicate the change in the death rate vs. the number of tests (**left plot**) and time (**right plot**). The histogram represents the number of weeks with the respective fraction of positive tests. The green line represents the fraction of positive tests vs. time.

The number of weekly performed PCR tests had a significant impact (*p* < 0.001) on the fraction of hospitalized confirmed cases (more performed tests were positively correlated with fewer hospitalizations, see Figure 8A). Moreover, the fraction of positive tests had a significant effect (*p* < 0.001) on the fatality rate of outpatients and patients in a general ward (higher positive ratios were associated with a higher fatality rate, see Figure 8B).

### 3.8. Hospitalization Rates and Time Effects

Investigation of inpatient data and model changepoint analysis revealed shifts in disease stage transitions and outcome dynamics that could not solely be explained by the already included covariates. The time until discharge of mechanically ventilated patients decreased noticeably during the summer of 2020, and a changepoint was estimated by the model on 10 June 2020, with a significant reduction of the time until discharge by 65% (from 47.3 days to 16.7 days, *p* < 0.001).

Furthermore, comparing data from our clinical database and newly confirmed cases over time, a drop in the fraction of patients requiring inpatient treatment could be observed in September 2020, and the model estimated a significant decrease in the fraction requiring inpatient treatment of 49.6% on 9 September 2020 (RSE 1.6%, *p* < 0.001). Simultaneously, the rate of inpatients requiring ICU treatment increased (*p* < 0.001). This increase was significantly higher in seven (Bavaria, Berlin, Bremen, Hamburg, Hesse, North Rhine-Westphalia, and Saarland) of the 16 federal states (29.0% [RSE 4.2%] vs. 9.4% [RSE 10.2%]). However, the overall fraction of confirmed cases requiring ICU treatment still declined by 33.7–43.8%. The rate of modeled outpatient deaths increased to 23% for male patients older than 79 years during the second wave around 15 December 2020 and decreased again to 0% around 11 March 2021 (*p* < 0.001).

**Figure 9:**
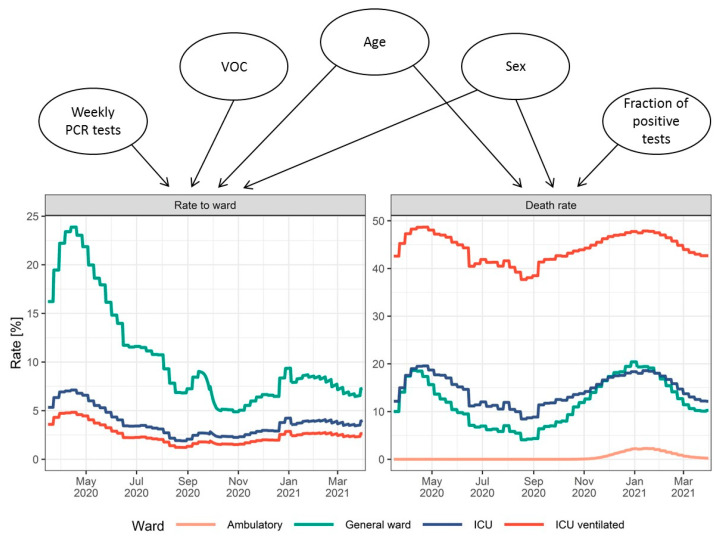
Covariate impacts on the modeled fraction of cases requiring treatment in different wards (**left**) and fatality rates differentiated by ward (**right**) resulting from covariate changes over time in Germany.

Figure 9 depicts the model flow rates that result from the incorporation of all previously described covariates and time effects.

### 3.9. German Districts

**Figure 10:**
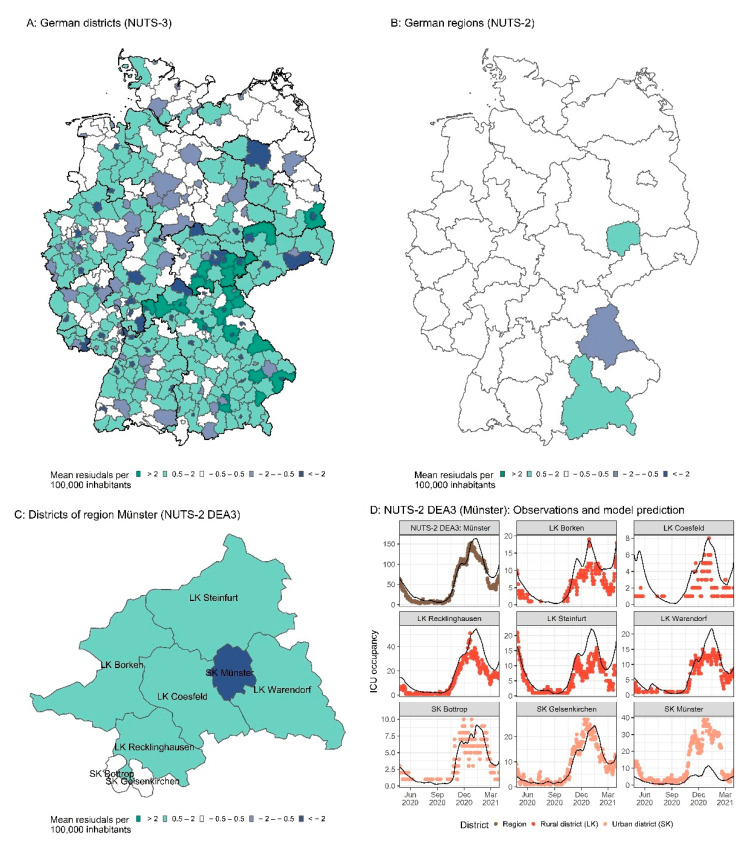
ICU prediction on the district level, (**A**): Mean residuals of ICU predictions per 100,000 inhabitants per district (NUTS-3). (**B**): Mean residuals of ICU predictions per 100,000 inhabitants per government region (NUTS-2). (**C**): Mean residuals of ICU predictions in counties per 100,000 inhabitants in exemplary government region DEA3. (**D**): Observations and model predictions of ICU occupancy for the full exemplary government region DEA3 and its districts. Points indicate observations, and lines indicate model predictions. LK refers to “Landkreis” (rural district). SK refers to “Stadtkreis” (urban district).

The model parameterized for Germany and the 16 federal states was subsequently applied to the pandemic in the 400 German districts. The number of weekly cases in each district was described very well using the estimated federal state model changepoints for infectiousness with a district-specific random-effect estimation of the infectiousness (see Supplementary Figure S2). Moreover, the number of ICU patients was calculated based on the estimated number of local cases and the respective age and sex distributions. Figure 10 and Supplementary Figure S2 show that predictions of ICU patients and ventilated ICU patients are in reasonable agreement with the observed number of patients for many, but not all districts. To investigate which districts were not described well, we calculated the mean residuals of ICU predictions per 100,000 inhabitants for each district. Here, for 101 districts (25.3%) predictions were in good agreement with the observed data (residuals between −0.5 and 0.5), whereas for 113 districts (28.3%), larger underpredictions (residuals < −0.5) were seen, and for 182 districts (45.5%), sizable overpredictions (residuals > 0.5) could be observed. For four rural districts (1%), no information regarding the number of ICU inpatients was available. Discrepancies between observed and predicted ICU inpatient numbers were consistent in both total ICU occupancy and ICU patients receiving mechanical ventilation, with a Pearson correlation coefficient of the residuals (ICU inpatients and mechanically ventilated inpatients) of R^2^ = 0.81 (*p* < 0.001). It could be observed that the number of available ICU beds per inhabitant was significantly higher in urban districts (German “Stadtkreise”, SK) compared to rural districts (German “Landkreise”, LK; median 5.44 vs. 1.87 beds/1,000,000 inhabitants for SK and LK, respectively, *p* < 0.001). Further investigations revealed a correlation between the mean discrepancies and the number of ICU beds available per inhabitant (R^2^ = 0.62, *p* < 0.001). This relationship is also demonstrated via a closer investigation of an exemplary region in the Midwest of Germany (Figure 10C,D). The urban district Münster, which maintains a large academic hospital, hosts more ICU patients than predicted based on the cases of this district, whereas the surrounding rural districts host fewer patients than expected, especially at peak times of the pandemic.

We also investigated the predictive performance at the regional level (NUTS-2). NUTS (Nomenclature of Territorial Units for Statistics) is a European standard for the subdivision of countries into units of approximately the same population size while favoring administrative units [45]. NUTS-2 aggregates the districts (NUTS-3) into regions with 0.8 to 3 million inhabitants, most of which are smaller than the federal states (NUTS-1). In Germany, 38 NUTS-2 regions exist which correspond to governmental regions known as “Regierungsbezirke” in Germany. Predictions on the NUTS-2 level showed that occupied ICU beds were well predicted (Figure 10D), and over- or underprediction could be eliminated for 92% (35 of 38) of the regions (Figure 10B).

## 4. Discussion

In the presented work we developed a comprehensive mathematical model that describes the number of SARS-CoV-2 infections, inpatients, ICU patients with and without mechanical ventilation, recoveries, and fatalities for the first two waves of the SARS-CoV-2 pandemic in Germany, its 16 federal states, and its 400 districts. Here, the description of hospitalized patients and fatalities was based solely on the number of infections and on the age and sex of the patients, variant of concern B.1.1.7, and the testing strategy (number of tests performed weekly, test positive rate) as covariates.

To describe the number of infections, the change in infectiousness was estimated at discrete changepoints considering inter-state and inter-district variability over time. The use of changepoints allowed us to accurately describe the number of infections without a daily infectiousness reevaluation and to only incorporate and analyze significant epidemiological changes in the infection dynamics. Still, the average estimated infectiousness was similar to the infectiousness estimated using continuous (daily) variations [46].

Over the 48 weeks of investigation, 24 significant changepoints of infectiousness could be observed or estimated, with a mean period of 15.5 days between consecutive changepoints. During the winter months (October until February), the period between changepoints was smaller (mean 18.3 days, range 7–30 days and mean 11.8 days, range 6.2–18.4 days in summer and winter, respectively), which can be attributed to the faster spread of SARS-CoV-2 due to seasonality and the consequential high frequency of changes in NPIs. When the German government initiated nationwide measures to control the pandemic in October 2020 [47], the inter-state variability in R(t) decreased in comparison to the beginning of the pandemic, when federal state governments enacted individual measures that were only effective locally (23.2% CV and 11.2% CV before and after October 2020, respectively).

A total of 21 of the 24 changepoints of infectiousness could be attributed to changes in NPI policies. The closing of schools in the spring of 2020, for example, led to an average decrease in infectiousness of 31%, which is in line with several other studies where a decrease between 0% and 60% was documented [48]. Contact restrictions at the beginning of the pandemic reduced infectiousness by 42%. However, because other NPIs were stacked and nested during these times, the results regarding the extent of these effects are likely biased. The reopening and closure of schools during the summer and fall of 2020 were subject to different local regulations and were implemented at different points in time, which rendered evaluation of the effects of school reopening and closure on $R\left(t\right)$ impossible for the summer and fall of 2020.

The numbers of inpatients, ICU patients, ventilated patients, recoveries, and fatalities were well described based on the predicted number of infections and the age and sex of the infected patients, VOC B.1.1.7, the number of weekly performed PCR tests, and the test positivity rate. Here, the analysis of metrics derived from the clinical database MetaKIS allowed the analysis of the typical hospital stay of inpatients with COVID-19, including the time until discharge and time spent in the ICU and with mechanical ventilation. With a mean length of hospital and ICU stay of 12.7 and 9.3 days, respectively, our analysis of individual inpatient data was in line with observations from Berger et al. [49], which were obtained by dividing the respective current number of patients by the respective cumulative number of patients (length of hospital and ICU stay of 14.3 and 12.8 days, respectively).

Moreover, our analysis is in line with other studies showing that the risk of hospitalization and death increases exponentially with increasing age [9,10,11,14,15,50]. The risk of ICU treatment among hospitalized patients was highest in the age group 60–80, which is in agreement with results from Switzerland and the United States, where patients aged 55–74 had the highest risk of ICU treatment [15,50]. The higher risk of more severe outcomes in older patients (such as the need for inpatient treatment, need for ICU treatment, and fatality) might be driven by two main factors: (i) older patients often have more comorbidities such as obesity, hypertension, diabetes, cardiovascular diseases, and chronic respiratory diseases which impose a higher risk of severe disease [51]; and (ii) the ageing immune system results in hyperinflammatory, pathological innate responses as well as ineffective T cell response and diminished antibody maturation [50,51]. Differences in the immune response might also explain the higher risk of severe disease in male patients [52]. Herein, the highest risk was observed among elderly male patients, which is in line with previous findings [50].

The length of hospital stay and duration of ICU treatment or mechanical ventilation were also explored and stratified by the age and sex of the patients. The mean length of stay was similar for females and males (12.3 and 13.1 days, respectively), which is in line with findings from the United States by Ohsfeldt et al. [53] of 8.5 and 9.6 days for females and males, respectively. The difference between female and male patients was even smaller when analyzed by ward, as more male patients received ICU treatment, which is associated with a longer stay. For patients staying in a general ward only, both male and female patients had an average length of stay of 11.2 days; for ICU patients, the average length of stay was 19.6 days for females and 19.7 days for males.

Older patients stayed in the hospital for a longer period (e.g., 7.4 days and 13.1 days for patients < 35 and ≥35 years, respectively). However, this difference was smaller when considering the higher fraction of older patients requiring ICU treatment: Patients not requiring ICU treatment stayed on average 6.7 and 11.5 days, for ages < 35 and ≥35 years, respectively. ICU patients had an average stay of 16.5 and 19.8 days for ages <35 and ≥35 years, respectively. Furthermore, patients younger than 35 years old only represented 6.5% of all inpatients. Hence, the impact of correcting the duration of stay for the age of the patients was very small in comparison to the added level of complexity to the model.

VOC B.1.1.7 has been shown to have increased ACE2 binding and cell infectivity due to a mutation on the spike protein which results in an advantage in transmissibility and an increase in disease severity compared to the wild type. By assessment of the proportion of B.1.1.7 cases and the number of reinfections, Graham et al. estimated the increase in the effective reproduction number to be 35% based on data from the United Kingdom [7], which we used to describe the number of cases in our model, resulting in a good correlation between the increase of cases and the appearance of VOC B.1.1.7.

At the time of model development, the impact of VOC B.1.1.7 on the severity of disease was not clear. A study from Denmark reported a relative risk of hospital admission of 1.42 [54], and a study from the United Kingdom reported a mortality hazard ratio of 1.64 in comparison to the wild type [55]. Both studies adjusted for confounding patient characteristics such as age and sex. Using our multivariate approach, we differentiated between the increase in the fraction of patients hospitalized (39.5%) and the fraction treated in an ICU (16.2%) in Germany while also considering other model covariates, such as age, sex, and testing strategy. The increase in hospitalization was similar to the increase found in Denmark [54]. In our model, no significant rise in death rates in the respective hospital wards could be observed. However, due to the increase in the fractions hospitalized and treated in an ICU, which are both linked to higher fatality rates, the apparent case fatality rate increased.

A comprehensive testing strategy was anticipated to result in a lower dark figure of cases and consequently a lower fraction of severe cases, as the fraction of detected asymptomatic infections would increase accordingly. This hypothesis was confirmed by our model, as the number of weekly tests was associated with a decrease in the fraction of hospitalized patients, and the test positivity rate was correlated with an increase in death rates. Here, both covariates improved the model performance significantly. The impact of the testing strategy has been previously discussed by Modi et al., who estimated infection rates based on the number of fatalities in Italy [56]. Liang et al. observed a similar link between mortality and the number of tests per 100 inhabitants in a dataset comprising 169 countries [57].

The combination of information from the clinical dataset and mathematical modeling allowed us to identify and quantify changes in the dynamics of the pandemic, which could not solely be explained by changes in the age and sex distribution of the infected population over time or other covariates. A significant decrease in the time until discharge for recovering of ventilated patients of 64.8% could be discovered in the summer of 2020. The date of change (8 August 2020) corresponded well to the time at which dexamethasone treatment for severe COVID-19 cases was first discussed and clinical guidelines were revised [58]. The model-estimated decrease of the time until discharge is in line with the work of Tomazini et al., who observed in 299 patients with COVID-19–associated acute respiratory distress syndrome that treatment with dexamethasone increased the number of ventilator-free days in recovered patients by 65.0% without decreasing mortality [59].

During the second wave of the pandemic in September 2020, the fraction of confirmed cases requiring inpatient and ICU treatment decreased. This trend was observed in the clinical database and confirmed by our model, with an estimated decrease in the hospitalization rate of 49.6% and of the patients requiring ICU treatment of 41.9% on 28 September 2020. While the underlying causes remain unknown, these changes might have been driven by an increased awareness of the disease and higher acceptance of comprehensive testing. Subsequently, the number of detected asymptomatic cases might have increased. For seven states, a significantly higher fraction of inpatients treated in an ICU could be observed (29% vs. 9%, *p* < 0.01). However, no explicit cause for the difference between these states was found.

Daily fatalities during the second wave could not be explained by the model’s covariates, nor could an increase in death rates be observed for hospitalized patients recorded in the clinical database. To describe excess fatalities, we assumed that this increase might have occurred for the death rate of outpatients, for which no explicit data were available in Germany. This assumption is in line with press reports that covered the high fatality rates in nursing homes and press critiques regarding nursing home residents not receiving adequate inpatient treatment during the time of unexplainable excess deaths [60].

In contrast to previously published epidemiological modeling approaches [14,15,61], the presented model was developed using data from the federal states (NUTS-1 level) and was successfully applied to describe the number of ICU patients in the whole country (NUTS-0 level), regions (NUTS-2 level), and districts (NUTS-3 level). The prediction of the district ICU and ventilated patient numbers underlines the importance of the covariate effects (age and sex) for the cases. However, for some districts, discrepancies between observations and model predictions could be observed. This was more pronounced for districts neighboring each other and correlated with the number of available ICU beds per inhabitant in the respective districts. Districts with overpredicted ICU occupancies were mostly rural districts with smaller or less specialized hospitals. Districts reporting higher ICU occupancies than predicted were often urban districts with larger hospitals (some of which are university hospitals) and higher ICU capacities. Hence, it seems plausible that small hospitals in districts with fewer ICU beds available tended to transfer patients to neighboring districts with larger hospitals and higher ICU capacities. Despite the observed discrepancies for some districts (NUTS-3 level), the model provided valuable information for the management of regional ICU capacities (NUTS-2 level).

Epidemiological ODE models describing the SARS-CoV-2 pandemic in other countries that were published previously used only stratification of infected patients by age [14,15,61] and ignored stratification by sex. However, some models used a more comprehensive stratification for age groups [15,61]. Here, our analysis was limited to the seven age groups for which the RKI provided the number of daily cases. Further limitations of our analysis originate from restricted access to information concerning the number of German COVID-19 inpatients, the background of fatalities, and further demographic information of all patients. For VOC B.1.1.7, only data for the nationwide average spread were available. Additionally, anonymization of the clinical database patient data prevented the tracking of transferred patients. Hence, the exclusion of patients with unknown outcomes from the analysis might have introduced bias regarding the estimation of inpatient outcome rates and duration of stay.

## 5. Conclusions

The presented mathematical model is an accurate tool for describing and analyzing the COVID-19 pandemic in Germany. It provides valuable insights into the expected number of inpatients and allows the simulation of different scenarios not only considering different levels of infectiousness but also the investigated covariates. Thereby, the model displayed the high effectiveness of NPIs to reduce the number of cases. Furthermore, the model could successfully be applied to describe the number of cases and ICU patients at the regional level (NUTS-2), highlighting the sufficiency of the covariates and the generalizable character of the model. Hence, the model might be applicable to other countries with similar health care systems. Furthermore, through the modeling process and when analyzing the patient data from the clinical database, changes in the course of the pandemic due to VOC B.1.1.7 and improved treatment modalities could be implemented successfully, demonstrating the flexibility of the model in adapting to further dynamic changes. The adaption to other VOCs and vaccinations has been ongoing over the past year, and the resulting model is available as a publicly accessible online simulation tool at https://covid-simulator.com/ (accessed on 1 September 2022).

## Figures and Tables

**Table 1:** Mean time until discharge, proportion of time on ICU and on a mechanical ventilator as well as standard deviations (sd) derived from the clinical database as used in the model for patients in the general ward, ICU inpatients, and ICU inpatients who needed mechanical ventilation as well as outcomes recovery and death.

Ward (Fraction of Patients)	Outcome (Fraction of Patients by Ward)	Total Duration until Discharge [Days] (sd)	Proportion of Time in ICU [%] (sd)	Proportion of Time Ventilated [%] (sd)
General ward only (81.8%)	Recovery (82.2%)	11.5 (11.4)		
	Death (17.8%)	10.6 (11.2)		
ICU without ventilation (6.0%)	Recovery (76.1%)	20.4 (17.1)	29 (96)	
	Death (23.9%)	20.0 (20.5)	44 (33)	
ICU with ventilation (12.2%)	Recovery (34.5%)	28.6 (18.3)	43 (39)	28 (21)
	Death (65.5%)	15.5 (12.6)	68 (31)	63 (34)

## Data Availability

Data can be made available upon request.

## Supplementary Materials

The following supporting information can be downloaded at: https://www.mdpi.com/article/10.3390/v14102114/s1, Figure S1: Descriptive performance plots for Germany and all federal states; Figure S2: Descriptive performance plots for German counties (NUTS-3); Model file S1: NONMEM model file of the infectiousness model; Model file S2: NONMEM model file of the full model; Table S1: Data Sources; Table S2: Summary of MetaKIS data. Age is summarized as median and interquartile range; Table S3: Changes in infectiousness according to NPIs and model-estimated changepoints; Table S4: Fractions of confirmed cases hospitalized, treated in an ICU, and ventilated and death rates as functions of age and sex as extracted from the MetaKIS database.

## Appendix A

The differential equations depicting the transfer of COVID-19 patients through different disease stages are shown below.

(A1) $$\frac{\mathrm{dS}}{\mathrm{dt}}=-\beta \left(t\right)\;*\;\frac{S\left(t\right)}{N}\;*\;I\left(t\right)$$

(A2) $$\frac{\mathrm{dI}}{\mathrm{dt}}=\beta \left(t\right)\;*\;\frac{S\left(t\right)}{N}\;*\;I\left(t\right)-\gamma \;*\;I\left(t\right)$$

(A3) $$\frac{\mathrm{dC}}{\mathrm{dt}}=y\;*\;I\left(t\right)$$

with N being the number of inhabitants. The transmission rate γ from the infectious stage (I) to confirmed cases (C) was fixed to an infectious period of 7 days, with γ = 1/7 based on 6 days of mean incubation time plus 1 day of lag between showing symptoms and registering a positive test result [36]. $Β\left(t\right)$ is defined as the transmission rate from the susceptible (S) to the infectious stage by contact with an infected subject and is related to γ and the infectiousness ($R_{\alpha }$) as depicted in Equation (A4):

(A4) $$\beta \left(t\right)=R_{\alpha }\left(t\right)\;*\;\gamma$$

The effective reproduction number $R\left(t\right)$ was defined as

(A5) $$R\left(t\right)=R_{\alpha }\left(t\right)\;*\;\frac{S\left(t\right)}{N}$$

The transmission rate ι from the allocator compartment (A) to the quarantine or hospital pathway was fixed to a high value (100 day^−1^) to model a short time lag.

(A6) $$\frac{\mathrm{dA}}{\mathrm{dt}}=\gamma \;*\;I\left(t\right)-\iota \;*\;A\left(t\right)$$

The age- and sex-specific fraction of hospitalized COVID-19 patients ($\mathrm{fh}\left(a,s\right)$) was derived from the number of hospitalized COVID-19 patients recorded in the clinical database and the number of cases according to RKI data as shown in Supplementary Table S4. The fraction hospitalized was corrected by an estimated fixed effect factor (Hosp), as the clinical database only included information on approximately 10% of German COVID-19 inpatients. The age-, sex-, and time-dependent hospitalization rate h(t) was calculated as the sum of each age- and sex-specific rate ($\mathrm{fh}\left(a,s\right)$) multiplied by the fraction of newly infected patients assigned to the respective age group at each time ($p\left(a,s,t\right)$):

(A7) $$h\left(t\right)=\sum_{s=\mathrm{Male}}^{s=\mathrm{Female}}\sum_{a=\mathrm{Age}\;0-5}^{a=\mathrm{Age}\;80+}\mathrm{fh}\left(a,s\right)\;*\;p\left(a,s,t\right)$$

For the influence of the numbers of weekly performed PCR tests in Germany, an exponential effect model with factor ${\mathrm{Fntest}}_{\mathrm{Hosp}}$, normalized for the average number of 1,000,000 tests per week, described the data best. The effect (${\mathrm{fH}}_{\mathrm{VOC}}$) of the fraction of VOC B.1.1.7 infections on the hospitalization rate was estimated using a linear effect model. Furthermore, a decrease in the hospitalization rate was observed both in the clinical database as well as in the number of occupied hospital beds. This decrease was implemented using a time-dependent hill equation, with fKH as the relative decrease in hospitalization rate and CPHosp as the timepoint at which the half-maximum change occurred. Hence, the final hospitalization rate $\mathrm{hosp}\left(t\right)$ considering all covariates can be depicted as:

(A8) $$\mathrm{hosp}\left(t\right)=h\left(t\right)\;*\;\mathrm{Ch}\left(t\right)$$

(A9) $$\mathrm{Ch}\left(t\right)={\;e}^{-{\mathrm{Fntest}}_{\mathrm{Hosp}}\;*\;\frac{\mathrm{NT}\left(t\right)}{1,000,000}}\;*\;\left(1+\mathrm{VOC}\left(t\right){\;*\;\mathrm{fH}}_{\mathrm{VOC}}\right)\;*\;\left(1-\frac{{\mathrm{fKH}\;*\;t}^{\mathrm{hill}}}{{\mathrm{CPHosp}}^{\mathrm{hill}}+t^{\mathrm{hill}}}\right)$$

The average delay between a positive test result and the deterioration of COVID-19 patients leading to hospitalization was implemented via two transit compartments ($T1_{\mathrm{hosp}}$, $T2_{\mathrm{hosp}}$) and the transit rate τ.

(A10) $$\frac{\mathrm{dT}1_{\mathrm{hosp}}}{\mathrm{dt}}=\mathrm{hosp}\left(t\right)\;*\;\iota \;*\;A\left(t\right)-\tau \;*\;T1_{\mathrm{hosp}}\left(t\right)$$

(A11) $$\frac{\mathrm{dT}2_{\mathrm{hosp}}}{\mathrm{dt}}=\tau \;*\;T1_{\mathrm{hosp}}\left(t\right)-\tau \;*\;T2_{\mathrm{hosp}}\left(t\right)$$

Quarantined individuals in an ambulatory setting (QR) were counted as recovered patients after 14 days, per the RKI definition of recovered cases; thus, the transit rate through the recovery compartments was defined as $\rho_{a}\;$= 2/14 day^−1^. The fraction of death among the outpatients (fa(t)) was estimated as dependent on age and sex (similar to calculations of the hospitalization rate, with $\mathrm{fda}\left(a,s\right)$ as age- and sex-specific death rate) and multiplied by the fraction of outpatients of each age group:

(A12) $$\mathrm{fa}\left(t\right)=\sum_{s=\mathrm{Male}}^{s=\mathrm{Female}}\sum_{a=\mathrm{Age}\;0-5}^{a=\mathrm{Age}\;80+}\mathrm{fda}\left(a,s\right)\;*\;\left(1-\mathrm{fh}\left(a,s,t\right)\;*\;\mathrm{Ch}\left(t\right)\right)\;*\;p\left(a,s,t\right)$$

Due to the lack of information regarding the course of COVID-19 in outpatients, it was assumed to be similar to the course of inpatients in a general ward. Hence, the average time to death for outpatients (${\mathrm{TD}}_{H,D}$) was set to the average time to death observed in inpatients in a general ward; the transit rate $\kappa_{a}\kappa_{a}=2/{\mathrm{TD}}_{H,D}$ for outpatients with outcome death was calculated as $\kappa_{a}=2/{\mathrm{TD}}_{H,D}$. Due to the lack of data on outpatient deaths in Germany, this death rate was estimated as a fixed effect.

Over time, several changes in death rates could be observed. Data from the clinical database showed a clear correlation between the death rate in general wards and the number of new infections as well as the positivity rate of PCR tests for SARS-CoV-2 (PR). Hence, both parameters were tested as covariates using different empirical effect models. The influence of PR on the death rate of general ward patients as well as outpatients improved the model performance significantly and was implemented using an exponential function with the estimated fixed effects ${\mathrm{PQ}}_{\max}$, ${\mathrm{PQ}}_{\min}$, and ${\mathrm{PQ}}_{\mathrm{rate}}$:

(A13) $${\mathrm{PQ}}_{\mathrm{Death}}\left(t\right)={\;\mathrm{PQ}}_{\max}\;*\;\left(1-{\mathrm{PQ}}_{\min}{\;*\;e}^{-\mathrm{PR}\left(t\right){\;*\;\mathrm{PQ}}_{\mathrm{rate}}}\right)$$

Initially, the model included only inpatient fatalities. All ambulatory patients recovered. However, at the beginning of the year 2021, we noticed an underestimation of COVID-19-related deaths by the model. At that time, reports about COVID-19-related deaths in German nursing homes accumulated [60]. Therefore, different rate changes in the death rate in an ambulatory setting were tested during this period. A model with an increase at the beginning of November 2020 followed by a decrease in February 2021 described the data best. These changes were implemented using empirical time-dependent hill functions with fixed effects regarding the extent ($D_{\mathrm{OUT}}$), the slope (${\mathrm{hill}}_{D}$), and the timepoints (${\mathrm{CP}}_{D1}$, ${\mathrm{CP}}_{D2}$) of the rate change:

(A14) $$\mathrm{DeathA}\left(t\right)=\;\mathrm{fa}\left(t\right)\;*\;\frac{D_{\mathrm{OUT}}{\;*\;t}^{{\mathrm{hill}}_{D}}}{{\mathrm{CP}}_{D1}{}^{{\mathrm{hill}}_{D}}+t^{{\mathrm{hill}}_{D}}}\;*\;\left(1-\frac{t^{{\mathrm{hill}}_{D}}}{{\mathrm{CP}}_{D2}{}^{{\mathrm{hill}}_{D}}+t^{{\mathrm{hill}}_{D}}}\right){\;*\;\mathrm{PQ}}_{\mathrm{Death}}\left(t\right)$$

Equations (A15)–(A18) describe the transit of patients in an ambulatory setting until they count as recovered (Equations (A15) and (A16)) or dead (Equations (A17) and (A18)).

(A15) $$\frac{{\mathrm{dQ}}_{R}}{\mathrm{dt}}=\left(1-\mathrm{DeathA}\left(t\right)\right)\;*\;\left(1-\mathrm{hosp}\left(t\right)\right)\;*\;\iota \;*\;A\left(t\right)-\rho_{a}{\;*\;Q}_{R}\left(t\right)$$

(A16) $$\frac{{\mathrm{dQ}}_{R2}}{\mathrm{dt}}=\rho_{a}{\;*\;Q}_{R}\left(t\right)-\rho_{a}{\;*\;Q}_{R2}\left(t\right)$$

(A17) $$\frac{{\mathrm{dQ}}_{D}}{\mathrm{dt}}=\mathrm{DeathA}\left(t\right)\;*\;\left(1-\mathrm{hosp}\left(t\right)\right)\;*\;\iota \;*\;A\left(t\right)-\kappa_{a}{\;*\;Q}_{D}\left(t\right)$$

(A18) $$\frac{{\mathrm{dQ}}_{D2}}{\mathrm{dt}}=\kappa_{a}{\;*\;Q}_{D}\left(t\right)-\kappa_{a}{\;*\;Q}_{D2}\left(t\right)$$

Transit rates for each ward were calculated by dividing the number of transit compartments by the time until discharge, with κ depicting the rates for dying patients, ρ for recovering patients, and suffixes H, ICU, and V for patients in a general ward, in an ICU, and receiving mechanical ventilation, respectively.

(A19) $$\kappa_{H}=\frac{2}{{\mathrm{TD}}_{H,D}}$$

(A20) $$\rho_{H}=\frac{2}{{\mathrm{TD}}_{H,R}}$$

(A21) $$\kappa_{\mathrm{ICU}}=\frac{2}{{\mathrm{TD}}_{I,D}}$$

(A22) $$\rho_{\mathrm{ICU}}=\frac{2}{{\mathrm{TD}}_{I,R}}$$

(A23) $$\kappa_{V}=\frac{2}{{\mathrm{TD}}_{V,D}}$$

(A24) $$\rho_{V}\left(t\right)=\frac{2}{{\mathrm{TD}}_{V,R}\left(t\right)}$$

Observational data from the clinical database showed a change in the time until discharge of recovering ventilated patients. This change was implemented into the model using a hill equation with estimated fixed effects $f_{\mathrm{Vent}1}$, $f_{\mathrm{Vent}2}$, ${\mathrm{CP}}_{\mathrm{Vent}}$, and hill:

(A25) $${\mathrm{TD}}_{V,R}\left(t\right)={\;\mathrm{TD}}_{V,R,\mathrm{base}}{\;*\;f}_{\mathrm{Vent}1}\;*\;\left(1+\frac{f_{\mathrm{Vent}2}{\;*\;t}^{\mathrm{hill}}}{{\mathrm{CP}}_{\mathrm{Vent}}{}^{\mathrm{hill}}+t^{\mathrm{hill}}}\right)$$

To describe the number of patients requiring treatment in an ICU, the rate had to be adapted with estimated fixed effects toICU1 and toICU2 at the timepoint ${\mathrm{CP}}_{\mathrm{Hosp}}$ at which the change in the hospitalization rate occurred. Furthermore, including an effect of $\mathrm{VOC}\left(t\right)$ improved the description of occupied ICU beds significantly. Therefore, the fraction of patients requiring ICU treatment was calculated from the age- and sex-specific fraction $I\left(t\right)$ and the covariate effects $\mathrm{Ci}\left(t\right)$:

(A26) $$\mathrm{toICU}\left(t\right)=I\left(t\right)\;*\;\mathrm{Ci}\left(t\right)$$

(A27) $$\mathrm{Ci}\left(t\right)=\;\mathrm{toICU}1\;*\;\left(1+\frac{\mathrm{toICU}2{\;*\;t}^{\mathrm{hill}}}{{\mathrm{CP}}_{\mathrm{Hosp}}{}^{\mathrm{hill}}+t^{\mathrm{hill}}}\right)\;*\;\left(1+\mathrm{VOC}\left(t\right){\;*\;\mathrm{fICU}}_{\mathrm{VOC}}\right)$$

(A28) $$I\left(t\right)=\sum_{s=\mathrm{Male}}^{s=\mathrm{Female}}\sum_{a=\mathrm{Age}\;0-5}^{a=\mathrm{Age}\;80+}\mathrm{fh}\left(a,s\right)\;*\;\mathrm{Ch}\left(t\right)\;*\;\mathrm{fi}\left(a,s\right)\;*\;p\left(a,s,t\right)$$

The fraction of patients requiring mechanical ventilation was dependent on the age and sex of the patients in an ICU:

(A29) $$\mathrm{toV}\left(t\right)=\sum_{s=\mathrm{Male}}^{s=\mathrm{Female}}\sum_{a=\mathrm{Age}\;0-5}^{a=\mathrm{Age}\;80+}\begin{array}{c} \;\;\;\;\;\;\;h\left(a,s\right)\;*\;Ch\left(t\right)\;*\;fi\left(a,s\right)\;*\;\\ \;\;\;\;\;\;Ci\left(t\right)\;*\;fv\left(a,s\right)\;*\;p\left(a,s,t\right) \end{array}$$

As described previously, the death rate in general wards (${\mathrm{Death}}_{H}$) was dependent on the age and sex of the patients $\mathrm{hd}\left(t\right)$ and changed with the rate of positive tests:

(A30) $${\mathrm{Death}}_{H}\left(t\right)=\mathrm{hd}\left(t\right){\;*\;\mathrm{PQ}}_{\mathrm{Death}}\left(t\right)$$

(A31) $$\mathrm{hd}\left(t\right)=\sum_{s=\mathrm{Male}}^{s=\mathrm{Female}}\sum_{a=\mathrm{Age}\;0-5}^{a=\mathrm{Age}\;80+}\mathrm{fh}\left(a,s\right)\;*\;\mathrm{Ch}\left(t\right)\;*\;\mathrm{fdh}\left(a,s\right)\;*\;p\left(a,s,t\right)$$

To assure a stable relation between the median death rates in each ward, the death rates for ICU $\mathrm{id}\left(t\right)$ and ventilated patients $\mathrm{vd}\left(t\right)$ extracted from the clinical database were corrected for the implantation of the covariate PR on the death rate in general wards by multiplying them with the influence of the median observed PR of 3.12% during the time of the investigation. The resulting death rates for ICU patients (${\mathrm{Death}}_{I}$) and ventilated patients (${\mathrm{Death}}_{V}$) are depicted in Equations (A32) and (A34).

(A32) $${\mathrm{Death}}_{I}\left(t\right)=\;\mathrm{id}\left(t\right){\;*\;\;\mathrm{PQ}}_{\max}\;*\;{\left(1-{\mathrm{PQ}}_{\min}{\;*\;e}^{-3.12{*\mathrm{PQ}}_{\mathrm{rate}}}\right)}_{\;}$$

(A33) $$\mathrm{id}\left(t\right)=\sum_{s=\mathrm{Male}}^{s=\mathrm{Female}}\sum_{a=\mathrm{Age}\;0-5}^{a=\mathrm{Age}\;80+}\;\;\;\;\;{\begin{array}{c} fh\left(a,s\right)\;*\;Ch\left(t\right)\;*\;fi\left(a,s\right)\;*\;\\ Ci\left(t\right)\;*\;fdi\left(a,s\right)\;*\;p\left(a,s,t\right) \end{array}}_{\;}$$

(A34) $${\mathrm{Death}}_{V}\left(t\right)=\;\mathrm{vd}\left(t\right){\;*\;\;\mathrm{PQ}}_{\max}\;*\;{\left(1-{\mathrm{PQ}}_{\min}{\;*\;e}^{-3.12{*\mathrm{PQ}}_{\mathrm{rate}}}\right)}_{\;}$$

(A35) $$\mathrm{vd}\left(t\right)=\sum_{s=\mathrm{Male}}^{s=\mathrm{Female}}\sum_{a=\mathrm{Age}\;0-5}^{a=\mathrm{Age}\;80+}{\begin{array}{c} \;\;\;\;fh\left(a,s\right)\;*\;Ch\left(t\right)\;*\;fi\left(a,s\right)\;*\;Ci\left(t\right)\;*\;\\ fv\left(a,s\right)\;*\;fdv\left(a,s\right)\;*\;p\left(a,s,t\right) \end{array}}_{\;}$$

The following ODE system was used for the description of COVID-19 inpatients, with H depicting patients in a general ward only, ICU depicting patients in an ICU during any portion of their stay, and V depicting patients requiring mechanical ventilation during their stay, with the suffixes death and recovery for the outcome of the patients:

(A36) $$\frac{{\mathrm{dH}}_{\mathrm{death}}}{\mathrm{dt}}={\mathrm{Death}}_{H}\left(t\right)\;*\;\tau \;*\;\left(1-\mathrm{toICU}\left(t\right)\right)\;*\;T2_{\mathrm{hosp}}\left(t\right)-\kappa_{H}{\;*\;H}_{\mathrm{death}}\left(t\right)$$

(A37) $$\frac{\mathrm{dH}2_{\mathrm{death}}}{\mathrm{dt}}=\kappa_{H}{\;*\;H}_{\mathrm{death}}\left(t\right)-\kappa_{H}\;*\;H2_{\mathrm{death}}\left(t\right)$$

(A38) $$\frac{{\mathrm{dICU}}_{\mathrm{death}}}{\mathrm{dt}}={\mathrm{Death}}_{I}\left(t\right)\;*\;\tau \;*\;\mathrm{toICU}\left(t\right)\;*\;\left(1-\mathrm{toV}\left(t\right)\right)\;*\;T2_{\mathrm{hosp}}\left(t\right)-{\;\kappa }_{\mathrm{ICU}}{\;*\;\mathrm{ICU}}_{\mathrm{death}}\left(t\right)$$

(A39) $$\frac{\mathrm{dICU}2_{\mathrm{death}}}{\mathrm{dt}}=\kappa_{\mathrm{ICU}}{\;*\;\mathrm{ICU}}_{\mathrm{death}}\left(t\right)-\kappa_{\mathrm{ICU}}\;*\;\mathrm{ICU}2_{\mathrm{death}}\left(t\right)$$

(A40) $$\frac{{\mathrm{dV}}_{\mathrm{death}}}{\mathrm{dt}}={\mathrm{Death}}_{V}\left(t\right)\;*\;\tau \;*\;\mathrm{toICU}\left(t\right)\;*\;\mathrm{toV}\left(t\right)\;*\;T2_{\mathrm{hosp}}\left(t\right)-\kappa_{V}{\;*\;V}_{\mathrm{death}}\left(t\right)$$

(A41) $$\frac{\mathrm{dV}2_{\mathrm{death}}}{\mathrm{dt}}=\kappa_{V}{\;*\;V}_{\mathrm{death}}\left(t\right)-\kappa_{V}\;*\;V2_{\mathrm{death}}\left(t\right)$$

(A42) $$\frac{{\mathrm{dH}}_{\mathrm{recovery}}}{\mathrm{dt}}=\left(1-{\mathrm{Death}}_{H}\left(t\right)\right)\;*\;\tau \;*\;\left(1-\mathrm{toICU}\left(t\right)\right)\;*\;T2_{\mathrm{hosp}}\left(t\right)-{\;\rho }_{H}{\;*\;H}_{\mathrm{recovery}}\left(t\right)$$

(A43) $$\frac{\mathrm{dH}2_{\mathrm{recovery}}}{\mathrm{dt}}=\rho_{H}{\;*\;H}_{\mathrm{recovery}}\left(t\right)-\rho_{H}\;*\;H2_{\mathrm{recovery}}\left(t\right)$$

(A44) $$\frac{{\mathrm{dICU}}_{\mathrm{recovery}}}{\mathrm{dt}}=\left(1-{\mathrm{Death}}_{I}\left(t\right)\right)\;*\;\tau \;*\;\mathrm{toICU}\left(t\right)\;*\;\left(1-\mathrm{toV}\left(t\right)\right)\;*\;T2_{\mathrm{hosp}}\left(t\right)\;-\;\rho_{\mathrm{ICU}}{\;*\;\mathrm{ICU}}_{\mathrm{recovery}}\left(t\right)$$

(A45) $$\frac{\mathrm{dICU}2_{\mathrm{recovery}}}{\mathrm{dt}}=\rho_{\mathrm{ICU}}{\;*\;\mathrm{ICU}}_{\mathrm{recovery}}\left(t\right)-\rho_{\mathrm{ICU}}\;*\;\mathrm{ICU}2_{\mathrm{recovery}}\left(t\right)$$

(A46) $$\frac{{\mathrm{dV}}_{\mathrm{recovery}}}{\mathrm{dt}}=\left(1-{\mathrm{Death}}_{V}\left(t\right)\right)\;*\;\tau \;*\;\mathrm{toICU}\left(t\right)\;*\;\mathrm{toV}\left(t\right)\;*\;T2_{\mathrm{hosp}}\left(t\right)\;-{\;\rho }_{V}\left(t\right){\;*\;V}_{\mathrm{recovery}}\left(t\right)$$

(A47) $$\frac{\mathrm{dV}2_{\mathrm{alive}}}{\mathrm{dt}}=\rho_{V}\left(t\right){\;*\;V}_{\mathrm{recovery}}\left(t\right)-\rho_{V}\left(t\right)\;*\;V2_{\mathrm{recovery}}\left(t\right)$$

All patients recovering enter the recovery transit compartments (Equations (A48) and (A49)) after their stay in their respective hospital wards before they are counted as recovered (R) to adjust recovery time to the RKI definition (14 days), with the transit rate ρ=2/14.

(A48) $$\frac{{\mathrm{dR}}_{\mathrm{hospital}}}{\mathrm{dt}}=\rho_{H}\;*\;H2_{\mathrm{recovery}}\left(t\right)+\rho_{\mathrm{ICU}}\;*\;\mathrm{ICU}2_{\mathrm{recovery}}\left(t\right)+\rho_{V}\;*\;\;V2_{\mathrm{recovery}}\left(t\right)-{\rho \;*\;R}_{\mathrm{hospital}}\left(t\right)t)$$

(A49) $$\frac{\mathrm{dR}2_{\mathrm{hospital}}}{dt}={\rho \;*\;R}_{\mathrm{hospital}}\left(t\right)-\rho \;*\;R2_{\mathrm{hospital}}\left(t\right)$$

The total number of recovered patients is calculated by Equation (A50):

(A50) $$\frac{\mathrm{dR}}{\mathrm{dt}}=\rho_{a}\;*\;\mathrm{QR}2\left(t\right)+\rho \;*\;R_{\mathrm{hospital}}\left(t\right)$$

In contrast to the recovered patients, fatalities (D) in hospitals were counted without delay.

(A51) $$\frac{dD}{dt}=\kappa_{H}\;*\;H2_{\mathrm{death}}\left(t\right)+\kappa_{\mathrm{ICU}}\;*\;\mathrm{ICU}2_{\mathrm{death}}\left(t\right)+\kappa_{V}\;*\;V2_{\mathrm{death}}\left(t\right)+{\;\kappa }_{a}\;*\;\mathrm{QD}2\left(t\right)$$

For all federal states, the number of patients currently in an ICU and being ventilated was multiplied by the fraction of time the patients stayed in the respective wards (Equations (A54)–(A56)). The ministries of the city-states of Berlin, Hamburg, and Bremen explicitly commented that a notable fraction of COVID-19 inpatients were not registered residents of the respective cities and hence did not contribute to the respective case counts. To account for this bias, correcting factors for Berlin, Bremen, and Hamburg were calculated: The number of resident inpatients in each city and the total number of inpatients (city residents and non-city residents) were available from 17 December 2020 to 7 January 2021. The factors ${\mathrm{fBL}}_{\mathrm{Berlin}}$, ${\mathrm{fBL}}_{\mathrm{Bremen}}$, and ${\mathrm{fBL}}_{\mathrm{Hamburg}}$ were calculated as the mean ratio of total inpatients to resident inpatients. Furthermore, for the federal state Hesse, the only inpatient data available consisted of the sum of confirmed and suspected COVID-19 cases in hospitals. Hence, the factor for Hesse inpatients ${\mathrm{fBL}}_{\mathrm{Hesse}}$ was calculated as the mean ratio of confirmed COVID-19 patients to the sum of suspected and confirmed cases.

The number of currently ventilated patients ($\mathrm{Ventilated}\left(t\right)$, Equation (A52)), current ICU patients ($\mathrm{ICU}\left(t\right)$, Equation (A53)), and currently hospitalized patients ($\mathrm{Hospitalized}\left(t\right)$, Equation (A54)) can be calculated by multiplying the number of patients in the ODE system (as depicted by Equations (A36)–(A47)) at time t with the time fractions spent in each ward as depicted in Table 1, with:

(A52) $$\mathrm{Ventilated}\left(t\right)=\left(\begin{array}{c} PVent_{V,R}\;*\;\left(V_{\mathrm{alive}}\left(t\right)+V2_{\mathrm{alive}}\left(t\right)\right)+\\ PVent_{V,D}\;*\;\left(V_{\mathrm{death}}\left(t\right)+V2_{\mathrm{death}}\left(t\right)\right) \end{array}\right){\;*\;\mathrm{fBL}}_{\mathrm{State}}\;$$

(A53) $$\mathrm{ICU}\left(t\right)=\left(\begin{array}{c} PICU_{I,R}\;*\;\left({\mathrm{ICU}}_{\mathrm{alive}}\left(t\right)+\mathrm{ICU}2_{\mathrm{alive}}\left(t\right)\right)+\\ PICU_{I,D}\;*\;\left({\mathrm{ICU}}_{\mathrm{death}}\left(t\right)+\mathrm{ICU}2_{\mathrm{death}}\left(t\right)\right)+\\ PICU_{V,R}\;*\;\left(V_{\mathrm{alive}}\left(t\right)+V2_{\mathrm{alive}}\left(t\right)\right)+\\ PICU_{V,D}\;*\;\left({\;V}_{\mathrm{death}}\left(t\right)+V2_{\mathrm{death}}\left(t\right)\right) \end{array}\right){\;*\;\mathrm{fBL}}_{\mathrm{State}}$$

(A54) $$\mathrm{Hospitalized}\left(t\right)=\left(\begin{array}{c} H_{\mathrm{alive}}\left(t\right)+H2_{\mathrm{alive}}\left(t\right)+\\ H_{\mathrm{death}}\left(t\right)+H2_{\mathrm{death}}\left(t\right)+\\ ICU_{\mathrm{alive}}\left(t\right)+ICU2_{\mathrm{alive}}\left(t\right)+\\ ICU_{\mathrm{death}}\left(t\right)+ICU2_{\mathrm{death}}\;\left(t\right)+\\ V_{\mathrm{alive}}\left(t\right)+V2_{\mathrm{alive}}\left(t\right)+\\ V_{\mathrm{death}}\left(t\right)+V2_{\mathrm{death}}\left(t\right) \end{array}\right){\;*\;\mathrm{fBL}}_{\mathrm{State}}$$

## Appendix B

**Table A1:** Model parameter estimates for the hospitalization and outcome model.

Model Parameter	Unit	Population Estimate	RSE [%]	Parameter Description
Population Parameters (Fixed Effects)				
Hosp	-	0.99	-	Factor on the age-specific hospitalization rates
fKH	-	−0.496	1.6	Relative change in hospitalization rate at ${\mathrm{CP}}_{Hosp}$
${\mathrm{CP}}_{Hosp}$	Day *	281	0.2	Time of hospitalization rate change
hill	-	100	-	Hill factor of time-dependent hospitalization and ICU rate change
${\mathrm{Fntest}}_{Hosp}$	10^6^/Tests	0.344	3.7	Slope of death rate change per 1,000,000 PCR tests performed
$f_{\mathrm{Vent}1}$	-	1.69	0.9	Factor time to discharge of recovering ventilated patients
$f_{\mathrm{Vent}2}$	-	−0.648	1.6	Relative change in fBEAT1 at ${\mathrm{CP}}_{Vent}$
${\mathrm{CP}}_{Vent}$	Day *	228	0.5	Time of change in fVent1
toICU1	-	0.476	0.9	Factor on the age-specific ICU rates
$\mathrm{toICU}2_{\alpha }$	-	0.29	4.2	Relative change of toICU for some states ** at ${\mathrm{CP}}_{Hosp}$
$\mathrm{toICU}2_{\beta }$	-	0.0904	10.2	Relative change of toICU for the states other than ** at ${\mathrm{CP}}_{Hosp}$
$\mathrm{toICU}2_{\gamma }$	-	0.153	0.0	Relative change of toICU Germany at ${\mathrm{CP}}_{Hosp}$
$PQ_{max}$	-	1.05	2.1	Maximum death rate change depending on the test positivity rate
$PQ_{min}$	-	0.48	2.7	Minimum death rate change depending on the test positivity rate
$PQ_{rate}$	1/%	0.129	9.9	Slope of death rate change depending on the test positivity rate
$D_{\mathrm{OUT}}$	day^−1^	0.226	5.4	Death rate of outpatients between ${\mathrm{CP}}_{D1}$ and ${\mathrm{CP}}_{D2}$
${\mathrm{CP}}_{D1}$	Day *	348	0.6	Time of change in $D_{\mathrm{OUT}}$
${\mathrm{CP}}_{D2}$	Day *	446	0.8	Time of change in $D_{\mathrm{OUT}}$
$hill_{D}$	-	27	10.6	Hill factor of time-dependent death rate changes
${\mathrm{fH}}_{\mathrm{VOC}}$	-	0.395	14.1	Relative hospitalization rate change for VOC B.1.1.7
${\mathrm{fICU}}_{\mathrm{VOC}}$	-	0.162	33.1	Relative rate change to ICU for VOC B.1.1.7
τ	-	100	-	Transit rate from confirmed case to inpatient
**Residual Errors**				
$E_{cases}$	%CV	0.55	3	Exponential error cases
$A_{cases}$	SD	32.6	4.6	Additive error cases
$P_{ICU}$	%CV	23.3	2.8	Proportional error ICU
$A_{ICU}$	SD	4.56	5	Additive error ICU
$P_{death}$	%CV	9.78	2.9	Proportional error fatalities
$A_{death}$	SD	11.8	6.8	Additive error fatalities
$P_{hosp}$	%CV	35.2	3.5	Proportional error hospitalizations
$A_{hosp}$	SD	11.8	13	Additive error hospitalizations
$P_{vent}$	%CV	29.7	2.6	Proportional error ventilated patients
$A_{vent}$	SD	1.48	9.3	Additive error ventilated patients
$P_{dailydeath}$	%CV	80.4	2.7	Proportional error daily fatalities
$A_{dailydeath}$	SD	0.36	6.4	Additive error daily fatalities
$P_{dailyhosp}$	%CV	261	3.5	Proportional error daily fatalities and hospitalizations
$A_{dailyhosp}$	SD	1.46	17	Additive error daily fatalities and hospitalizations

* as days since 22 December 2019; ** Bavaria, Berlin, Bremen, Hamburg, Hesse, North Rhine-Westphalia, and Saarland.
