# Supplementary material for: Model-Based Analysis of SARS-CoV-2 Infections, Hospitalization and Outcome in Germany, the Federal States and Districts
Source: Viruses. 2022 Sep 24;14(10):2114. doi: 10.3390/v14102114 (PMC9607468; doi:10.3390/v14102114)
Supplement: Supplementary file 1 [file viruses-14-02114-s001.zip › Figure S1.pdf]

Baden−Wuerttemberg

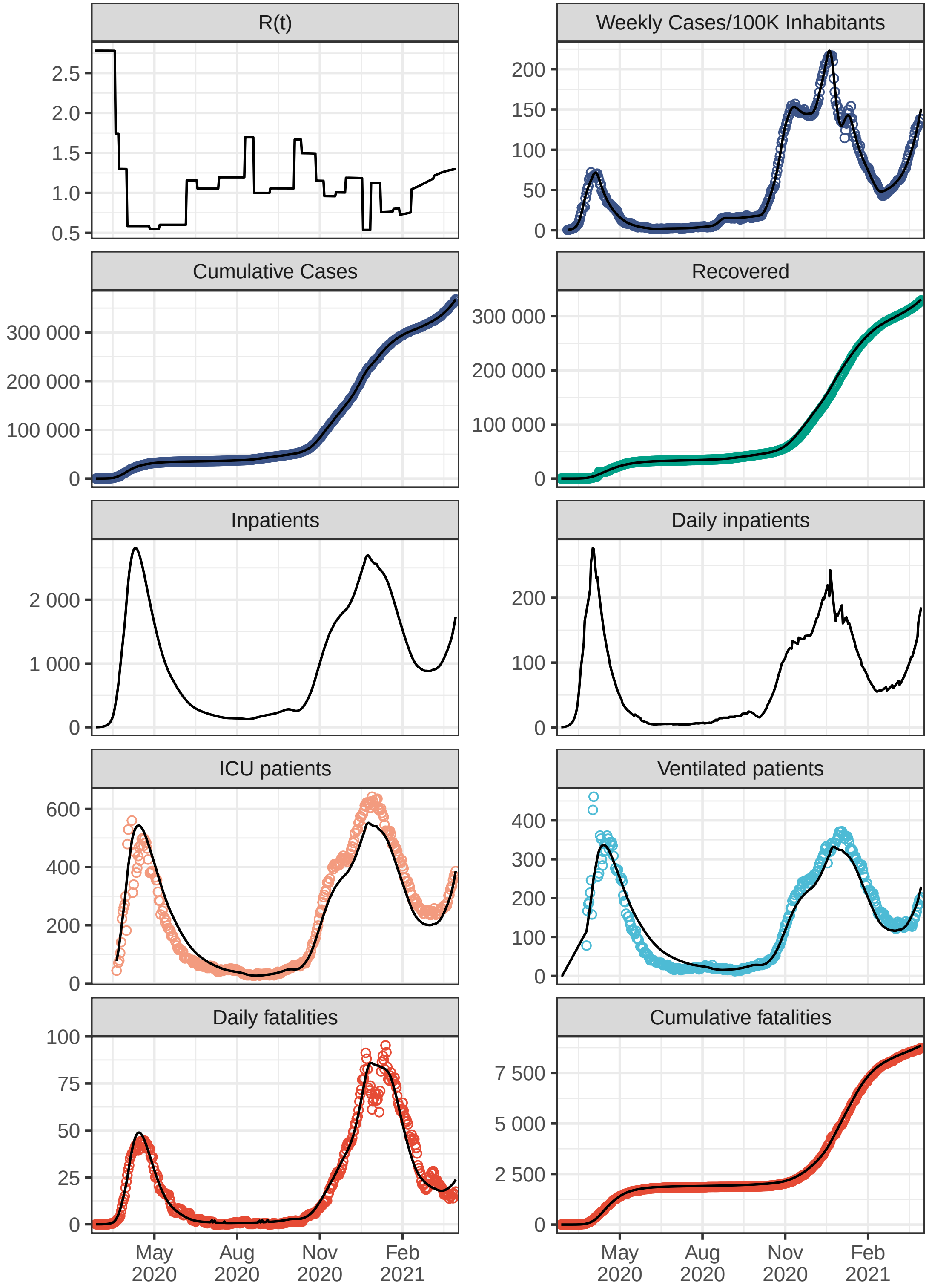

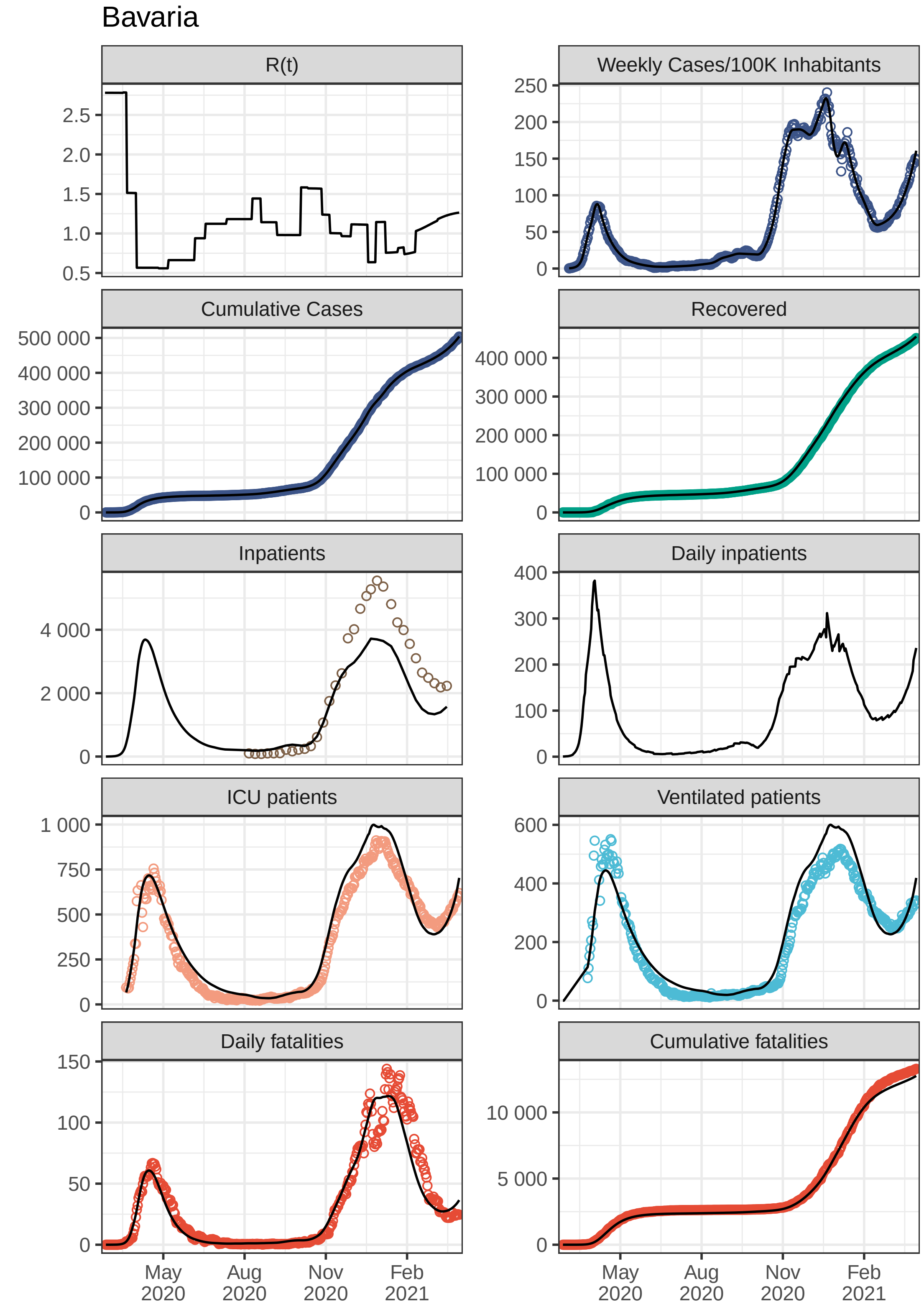

Bavaria
R(t)
Weekly Cases/100K Inhabitants
Cumulative Cases
Recovered
Inpatients
Daily inpatients
ICU patients
Ventilated patients
Daily fatalities
Cumulative fatalities
May 2020
Aug 2020
Nov 2020
Feb 2021

Berlin

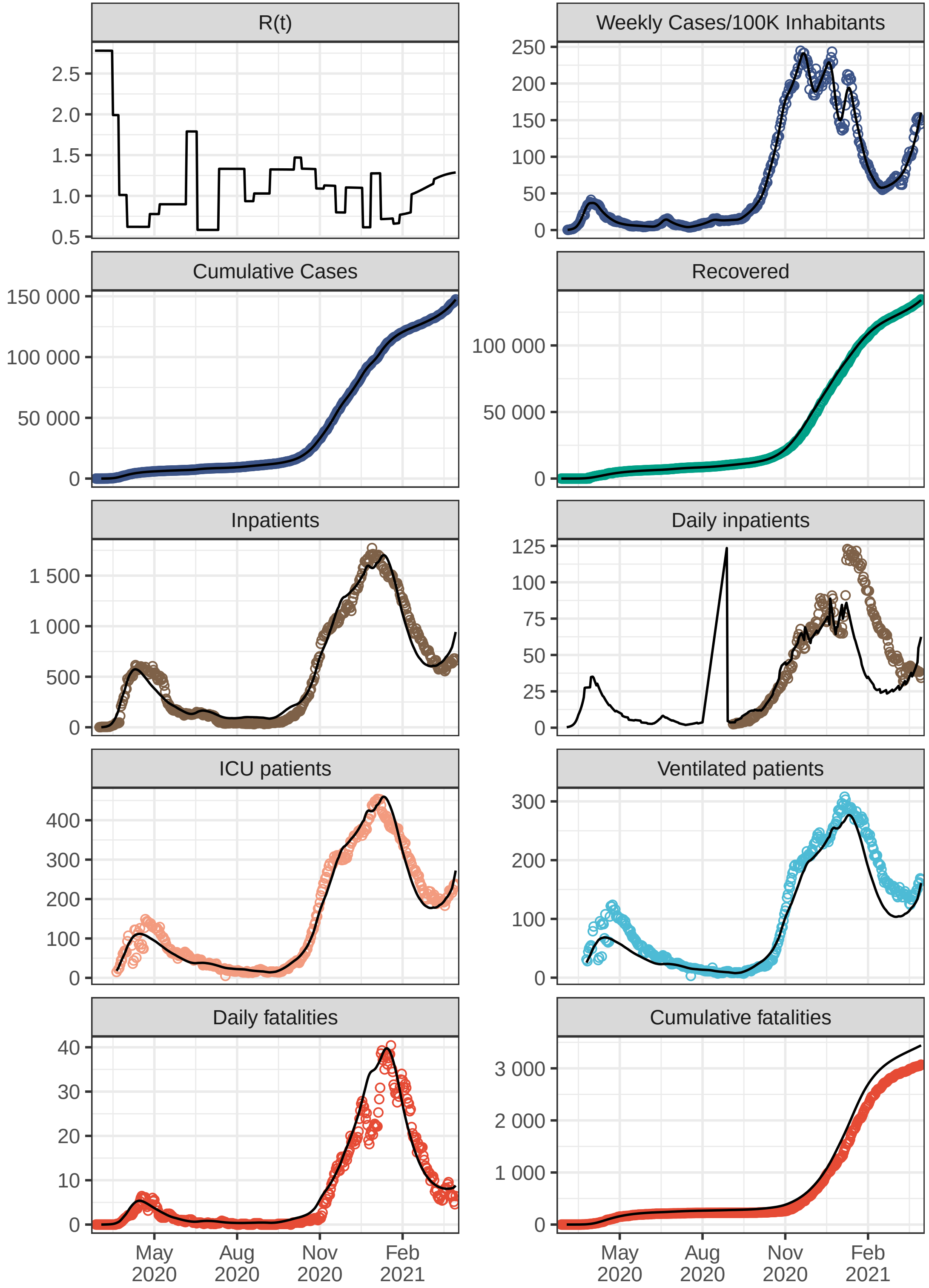

R(t)
Weekly Cases/100K Inhabitants
Cumulative Cases
Recovered
Inpatients
Daily inpatients
ICU patients
Ventilated patients
Daily fatalities
Cumulative fatalities
May 2020
Aug 2020
Nov 2020
Feb 2021

Brandenburg

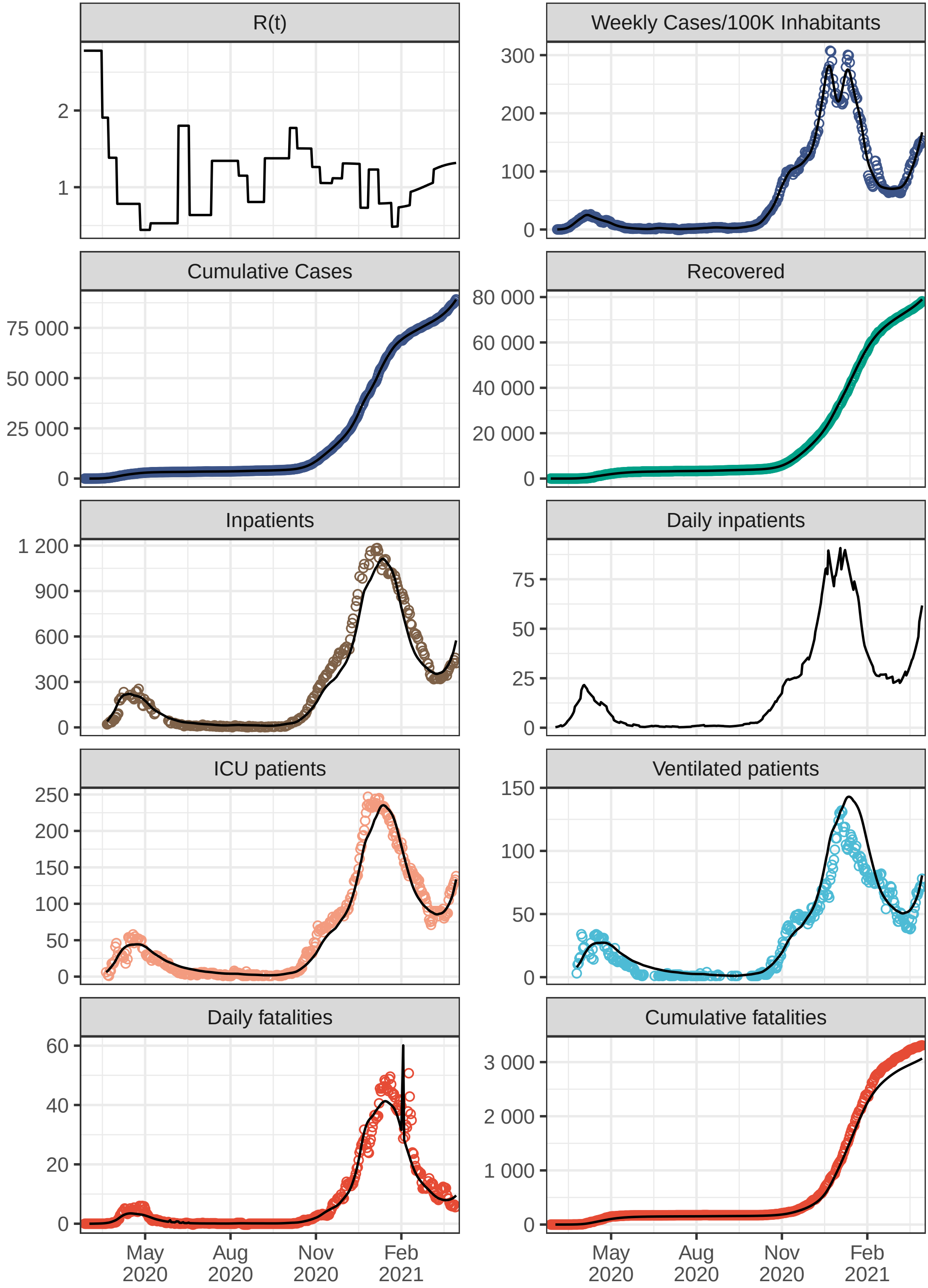

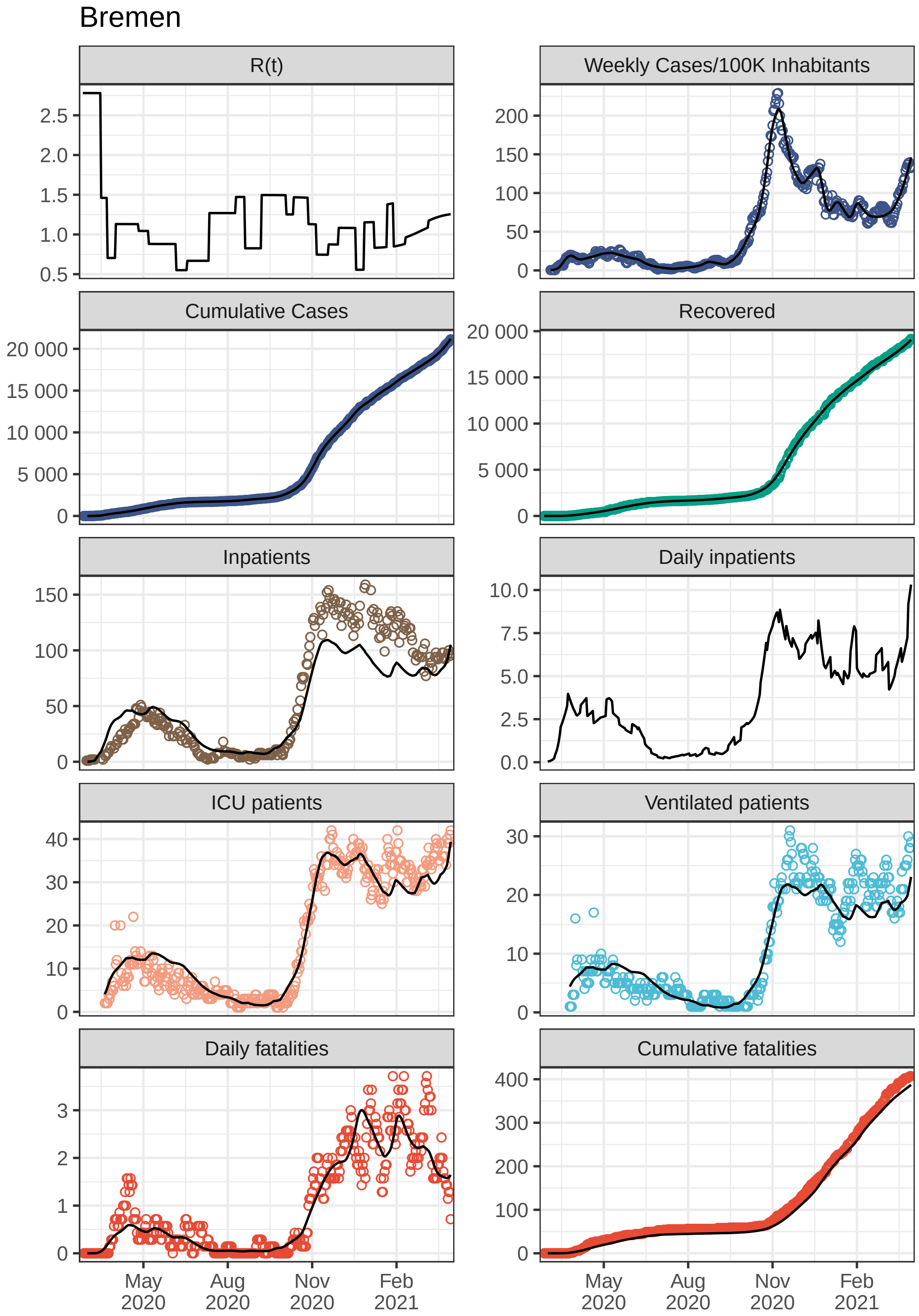

Bremen
R(t)
Weekly Cases/100K Inhabitants
Cumulative Cases
Recovered
Inpatients
Daily inpatients
ICU patients
Ventilated patients
Daily fatalities
Cumulative fatalities
May 2020
Aug 2020
Nov 2020
Feb 2021

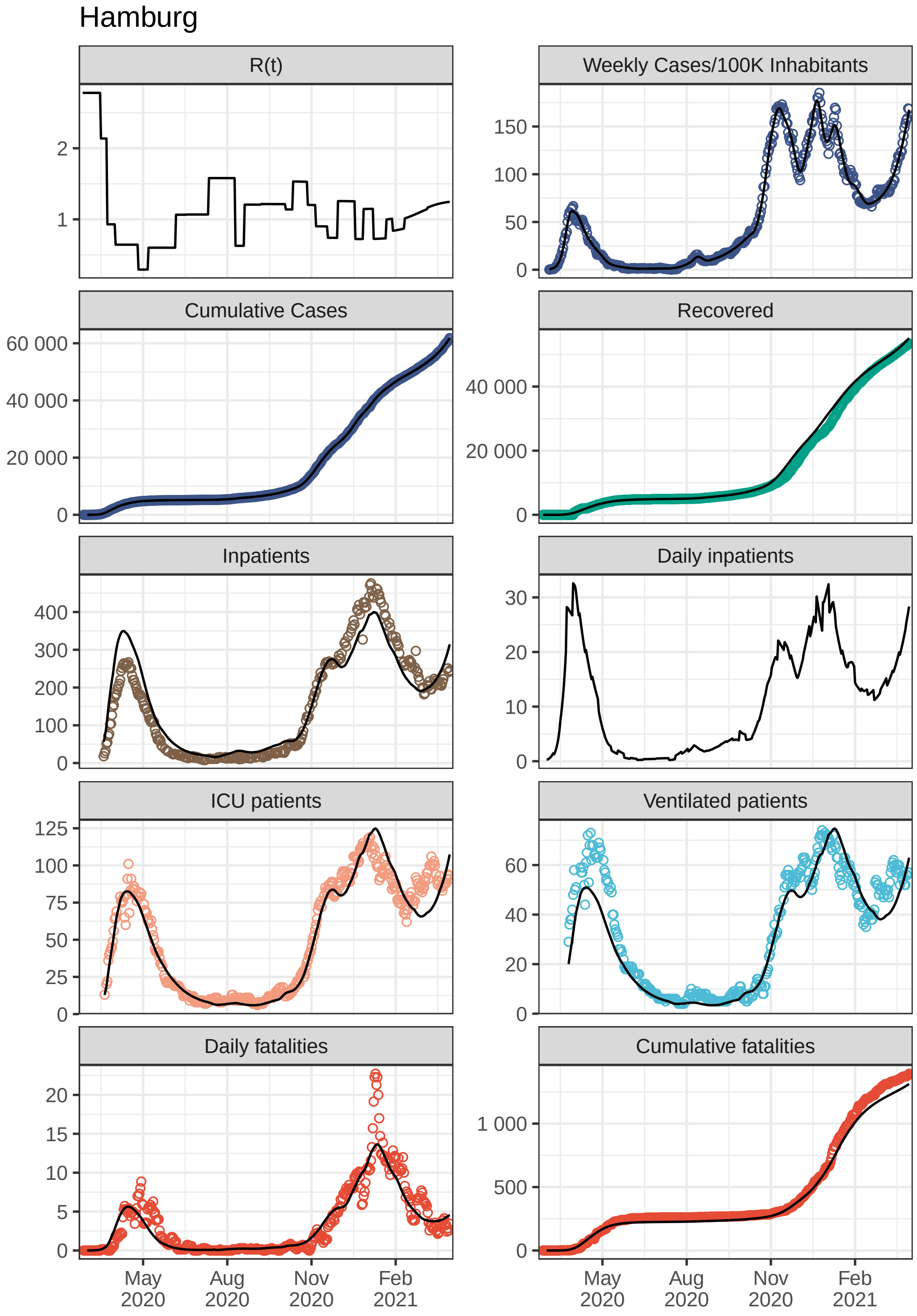
Hamburg
R(t)
Weekly Cases/100K Inhabitants
Cumulative Cases
Recovered
Inpatients
Daily inpatients
ICU patients
Ventilated patients
Daily fatalities
Cumulative fatalities
May 2020
Aug 2020
Nov 2020
Feb 2021

Hesse

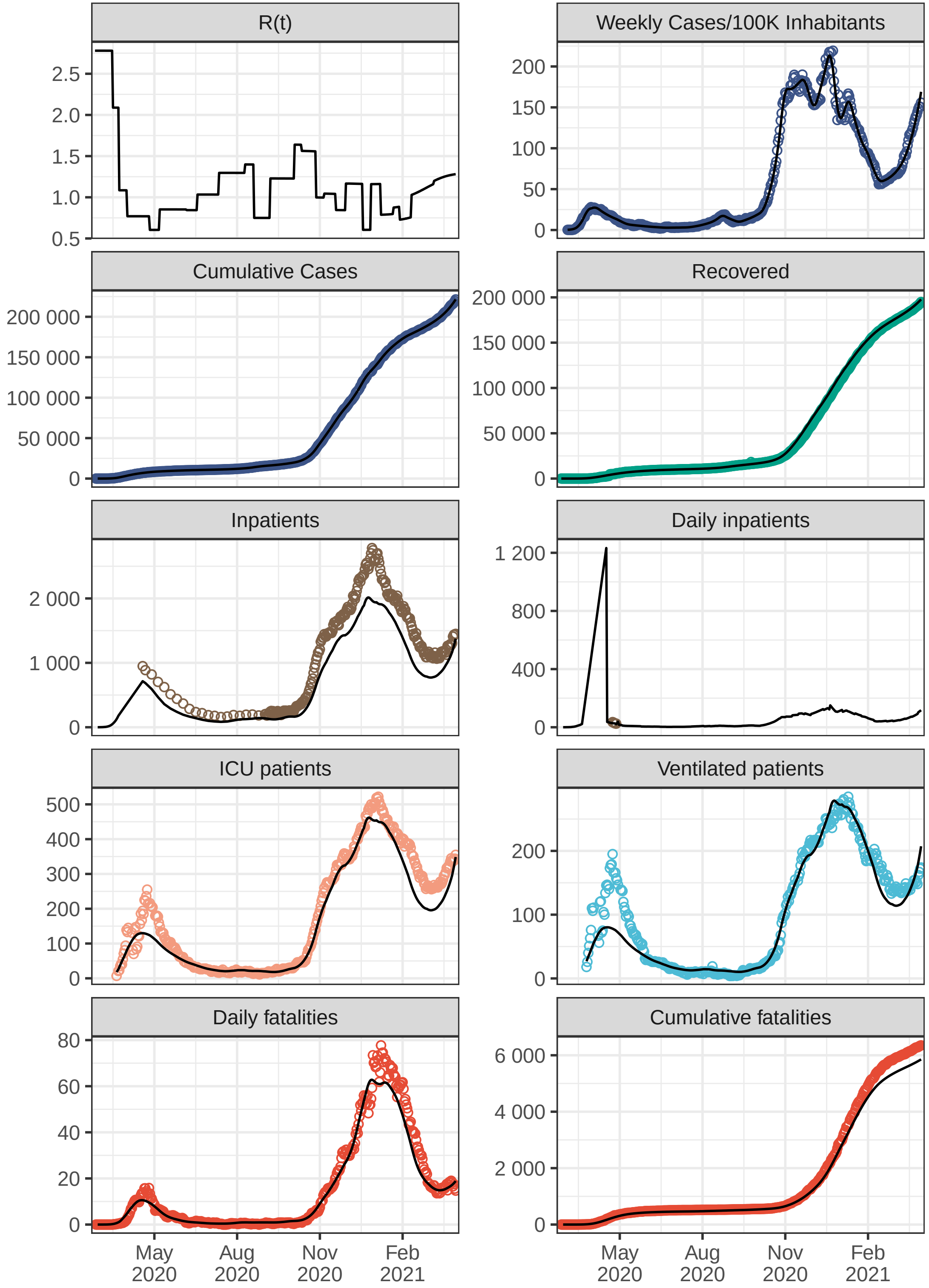

R(t)
Weekly Cases/100K Inhabitants
Cumulative Cases
Recovered
Inpatients
Daily inpatients
ICU patients
Ventilated patients
Daily fatalities
Cumulative fatalities
2.5
2.0
1.5
1.0
0.5
200
150
100
50
0
200 000
150 000
100 000
50 000
2 000
1 000
1 200
800
400
500
400
300
200
100
80
60
40
20
6 000
4 000
May 2020
Aug 2020
Nov 2020
Feb 2021

Mecklenburg−Vorpommern

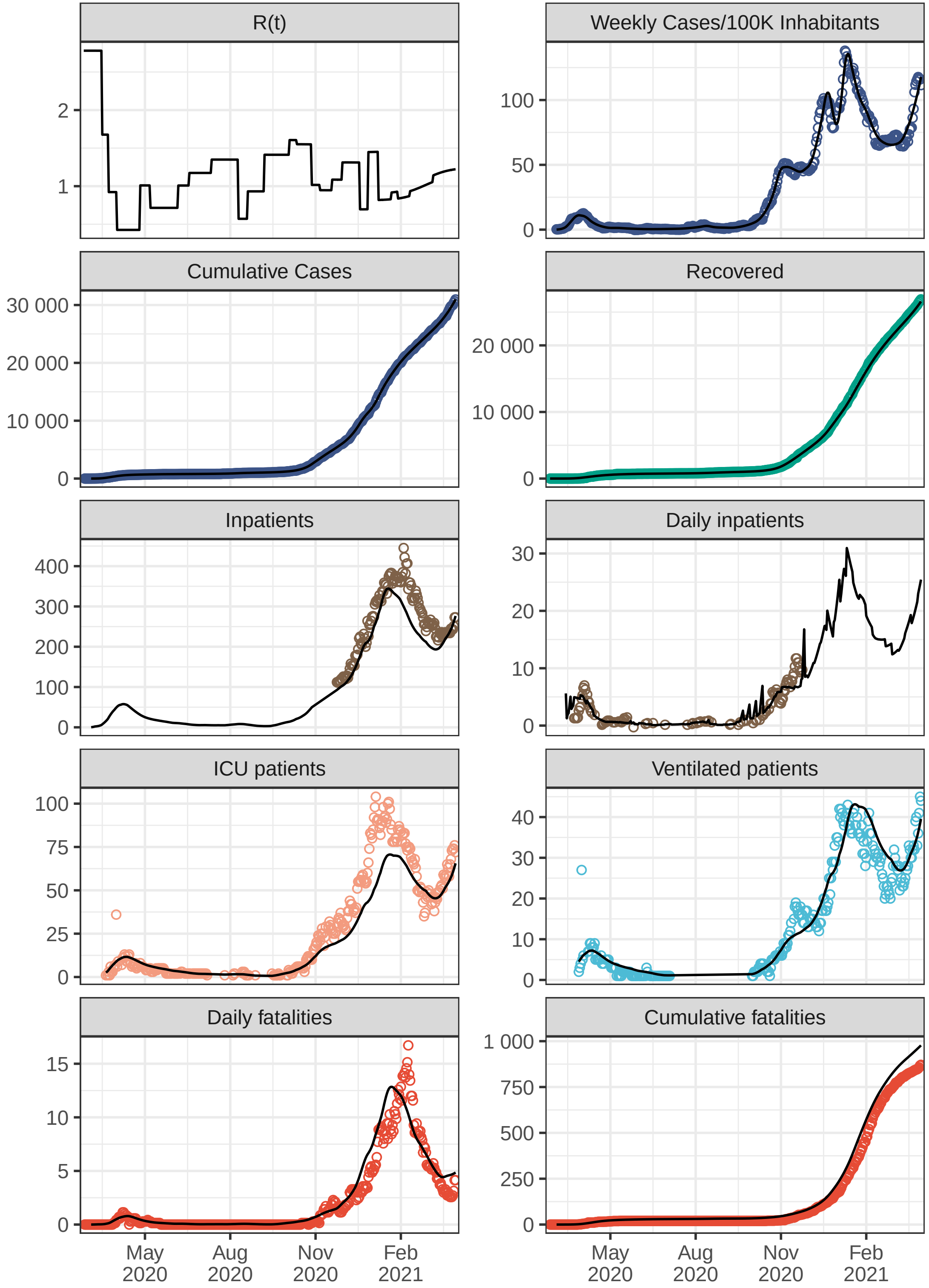

Lower Saxony

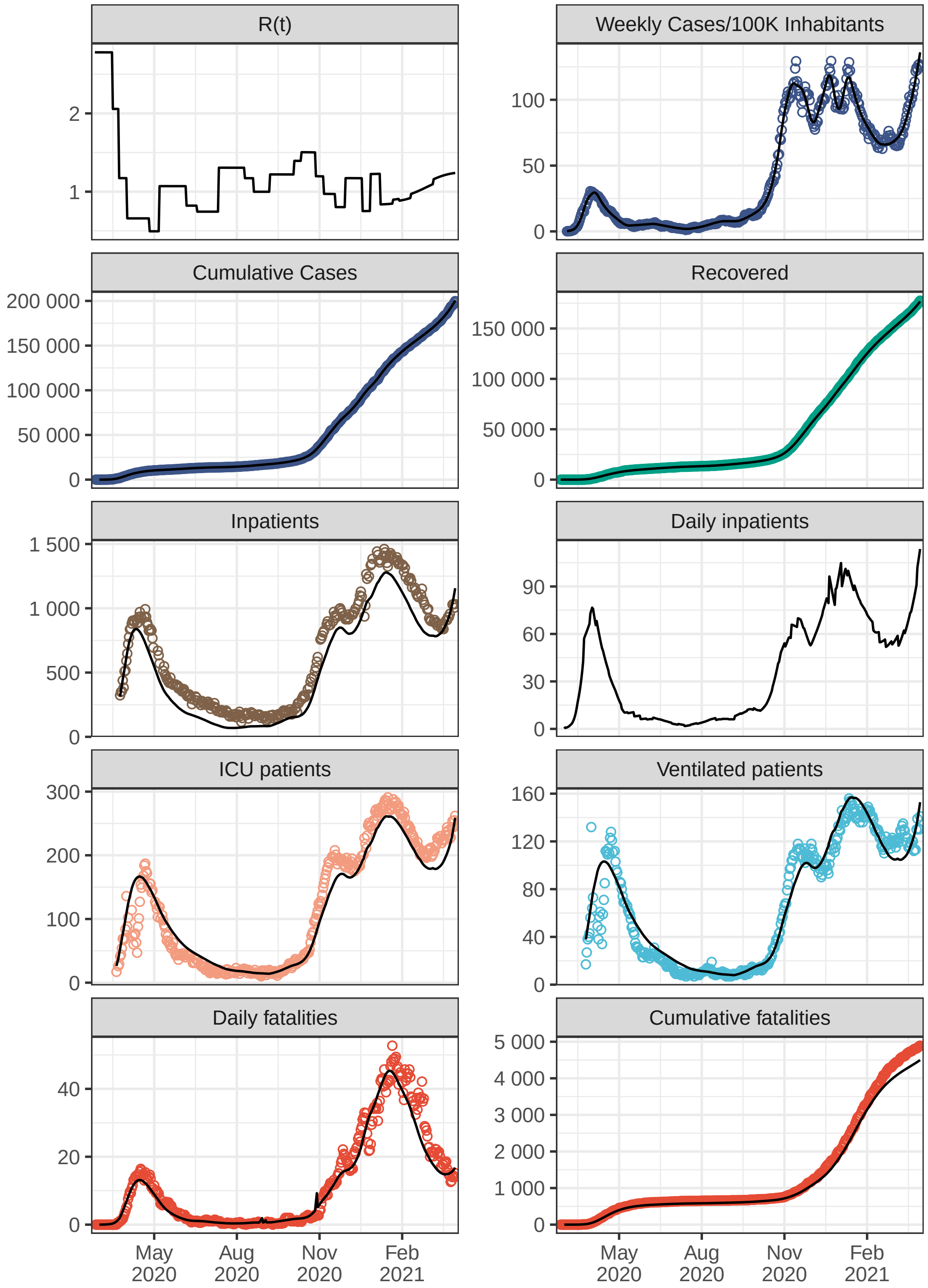

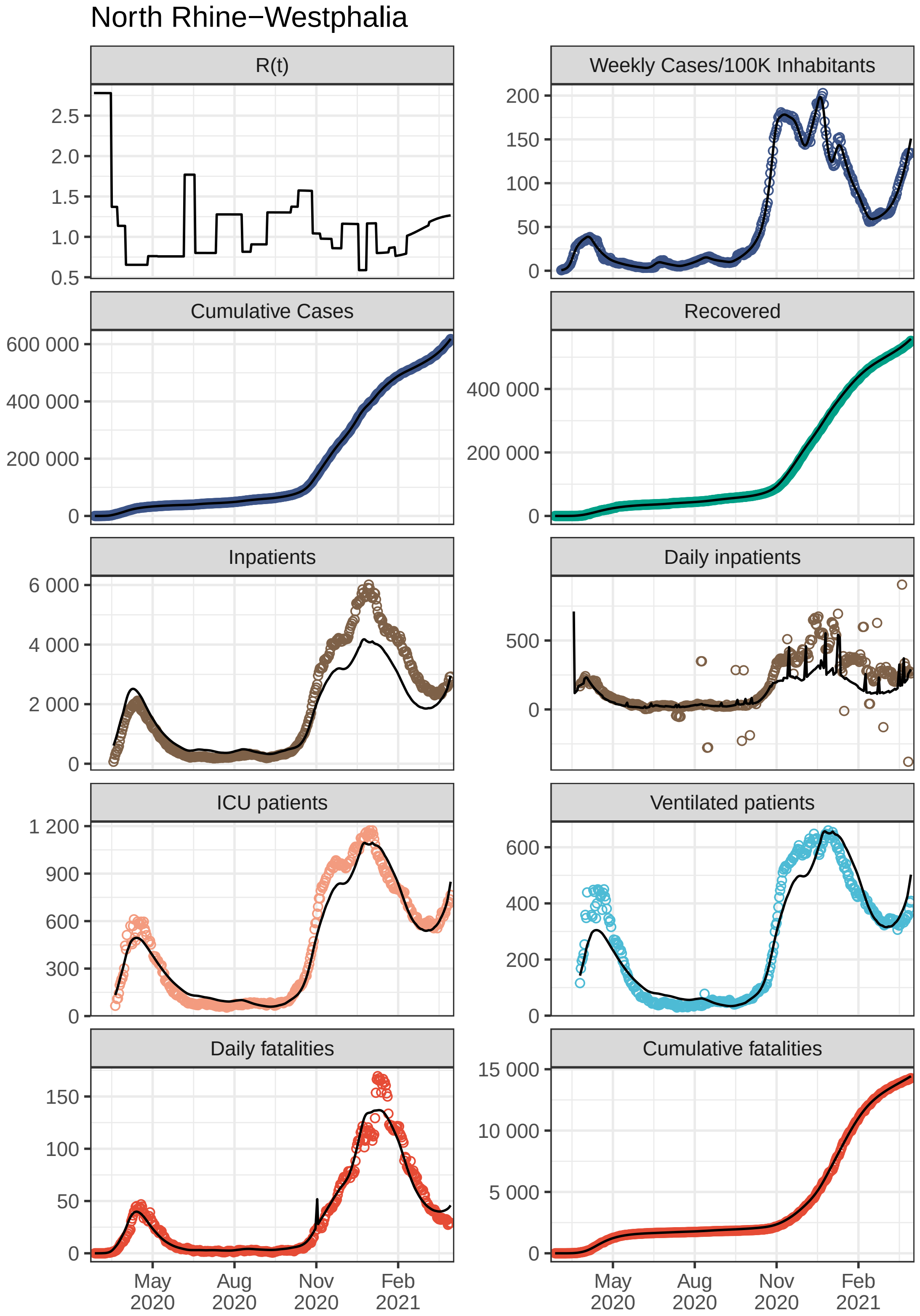
North Rhine−Westphalia
R(t)
Weekly Cases/100K Inhabitants
Cumulative Cases
Recovered
Inpatients
Daily inpatients
ICU patients
Ventilated patients
Daily fatalities
Cumulative fatalities
May 2020
Aug 2020
Nov 2020
Feb 2021

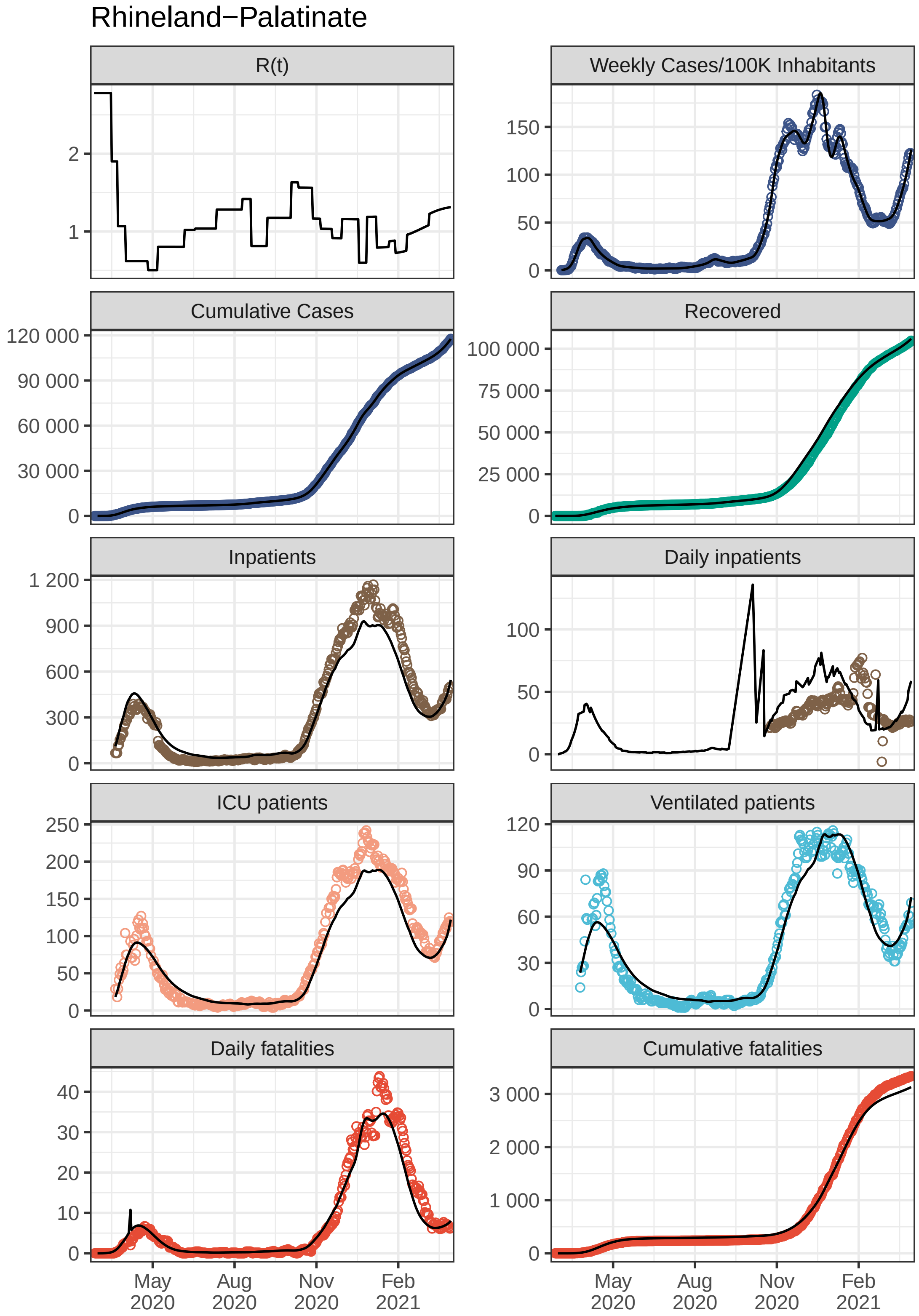

Rhineland−Palatinate
R(t)
Weekly Cases/100K Inhabitants
Cumulative Cases
Recovered
Inpatients
Daily inpatients
ICU patients
Ventilated patients
Daily fatalities
Cumulative fatalities
May 2020
Aug 2020
Nov 2020
Feb 2021

Saarland

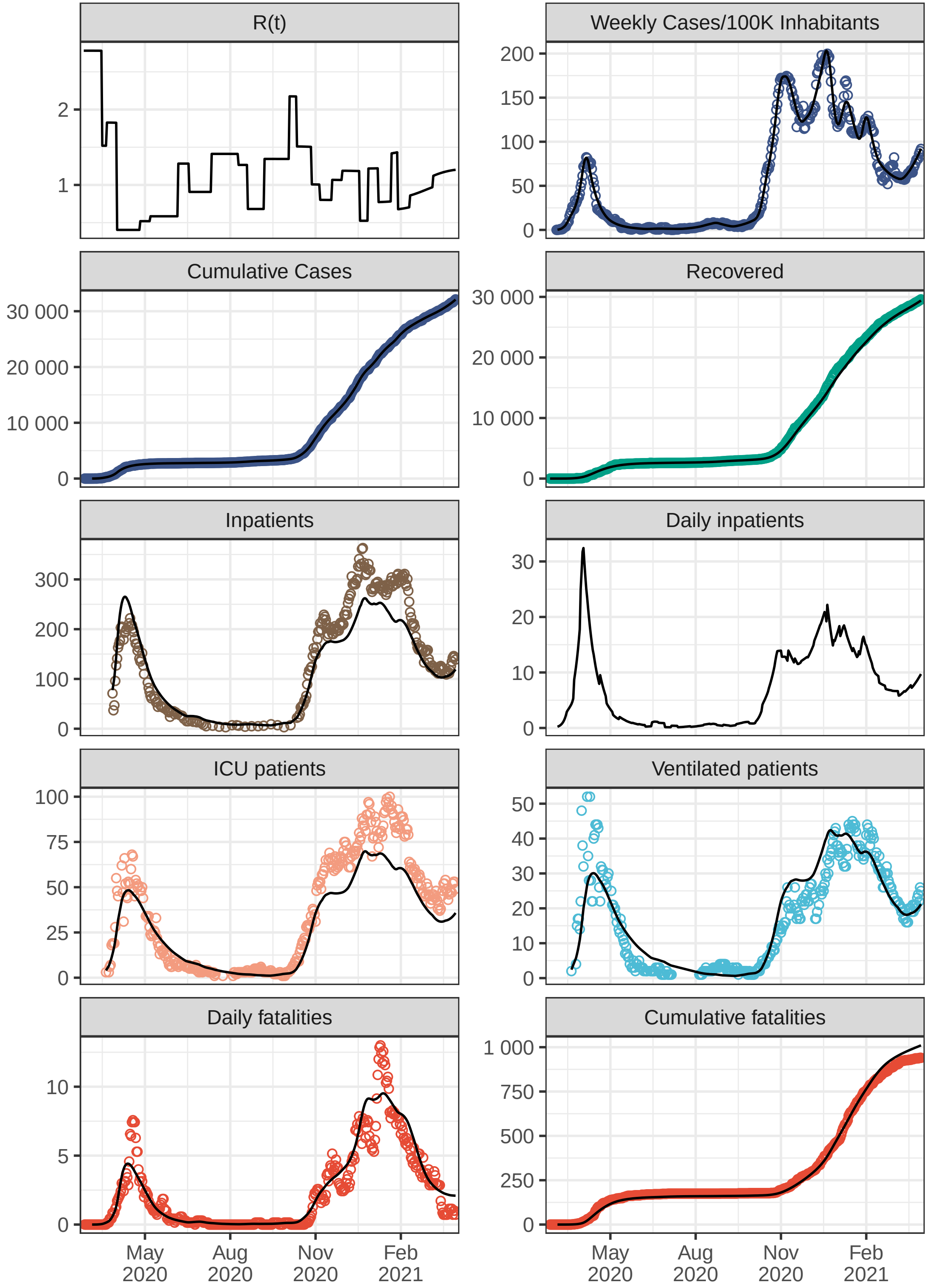
R(t)
Weekly Cases/100K Inhabitants
Cumulative Cases
Recovered
Inpatients
Daily inpatients
ICU patients
Ventilated patients
Daily fatalities
Cumulative fatalities
May 2020
Aug 2020
Nov 2020
Feb 2021

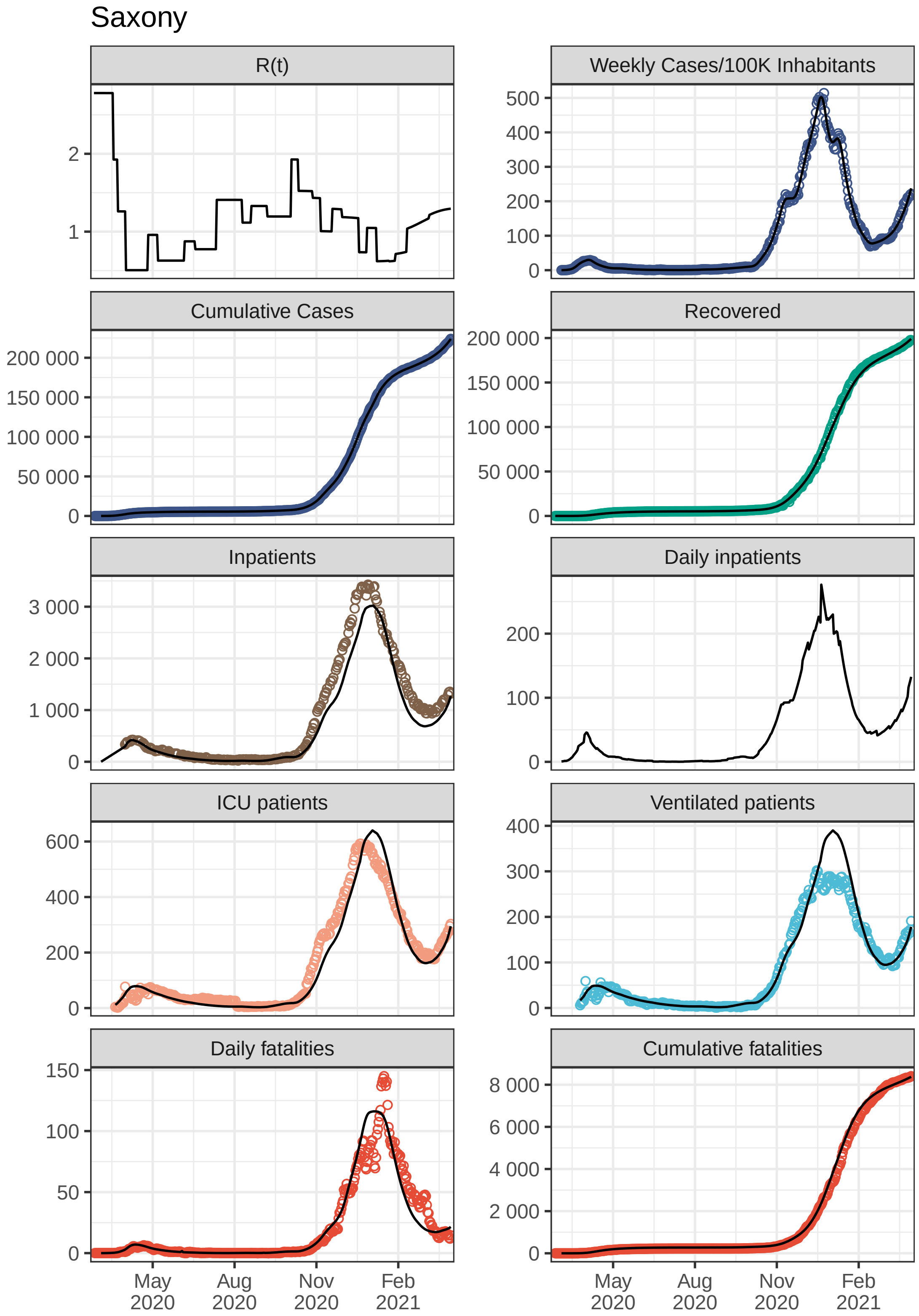

Saxony
R(t)
Weekly Cases/100K Inhabitants
Cumulative Cases
Recovered
Inpatients
Daily inpatients
ICU patients
Ventilated patients
Daily fatalities
Cumulative fatalities
May 2020
Aug 2020
Nov 2020
Feb 2021

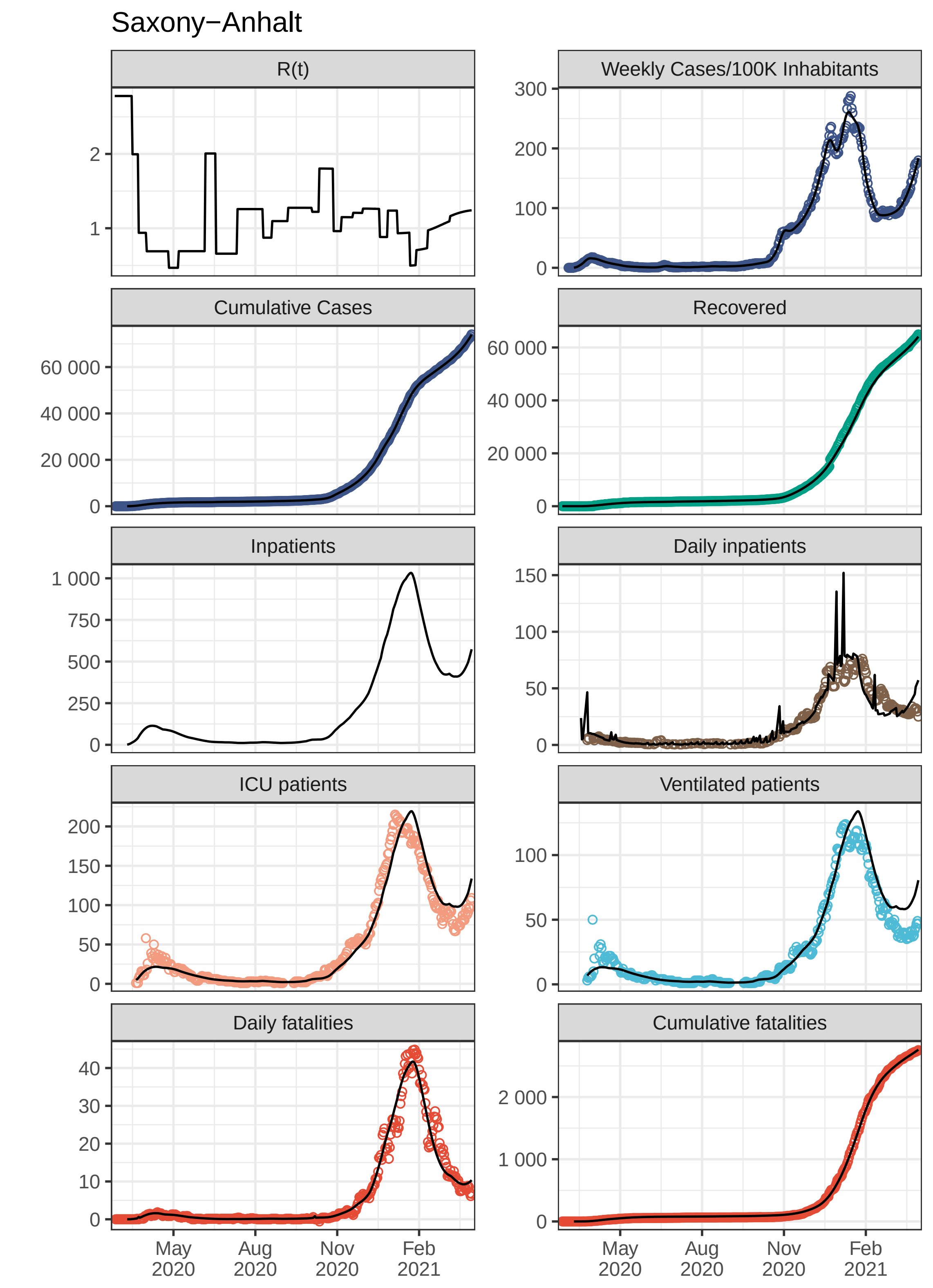

Saxony−Anhalt
R(t)
Weekly Cases/100K Inhabitants
Cumulative Cases
Recovered
Inpatients
Daily inpatients
ICU patients
Ventilated patients
Daily fatalities
Cumulative fatalities
May 2020
Aug 2020
Nov 2020
Feb 2021

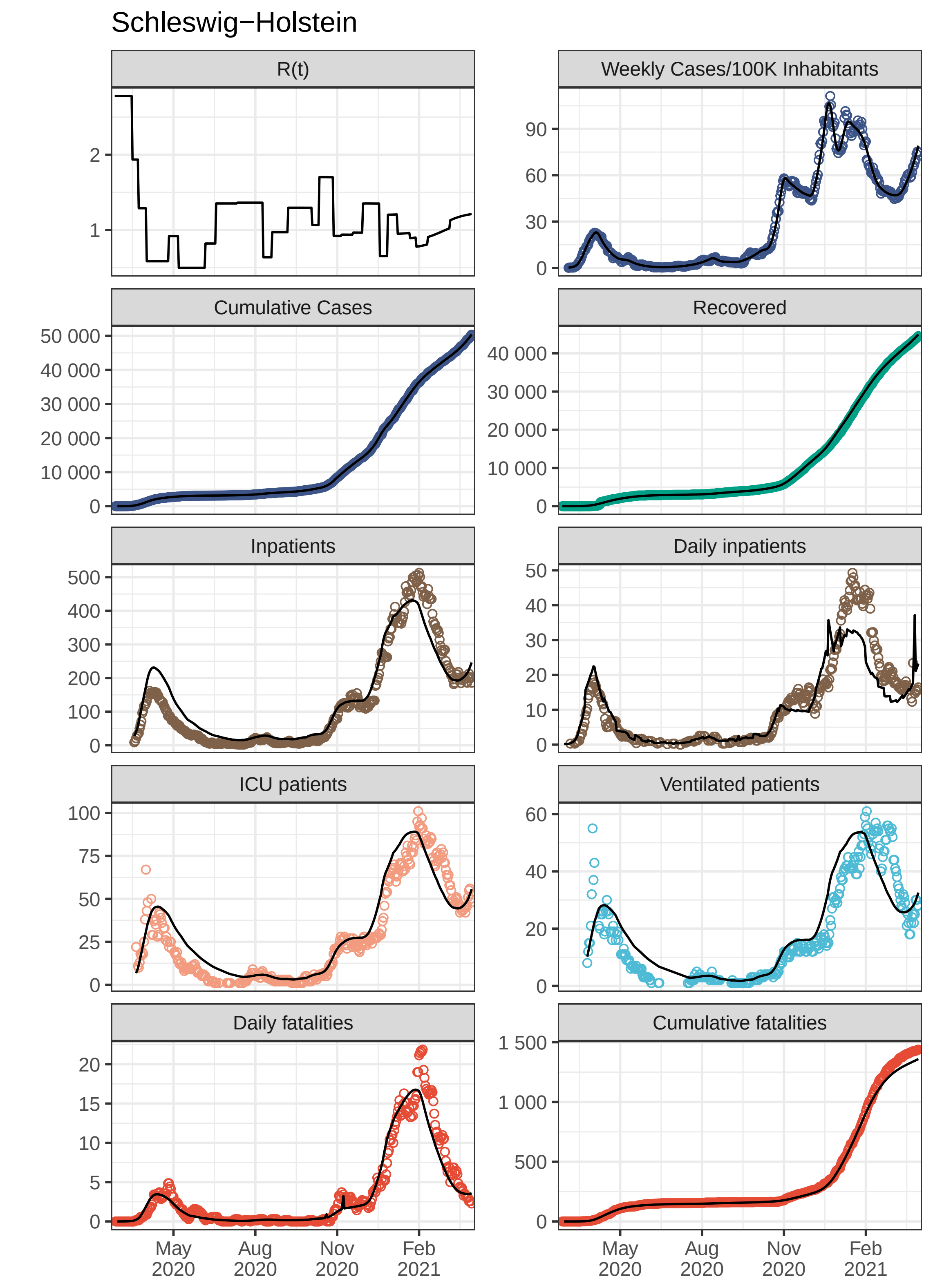

Schleswig−Holstein
R(t)
Weekly Cases/100K Inhabitants
Cumulative Cases
Recovered
Inpatients
Daily inpatients
ICU patients
Ventilated patients
Daily fatalities
Cumulative fatalities
May 2020
Aug 2020
Nov 2020
Feb 2021

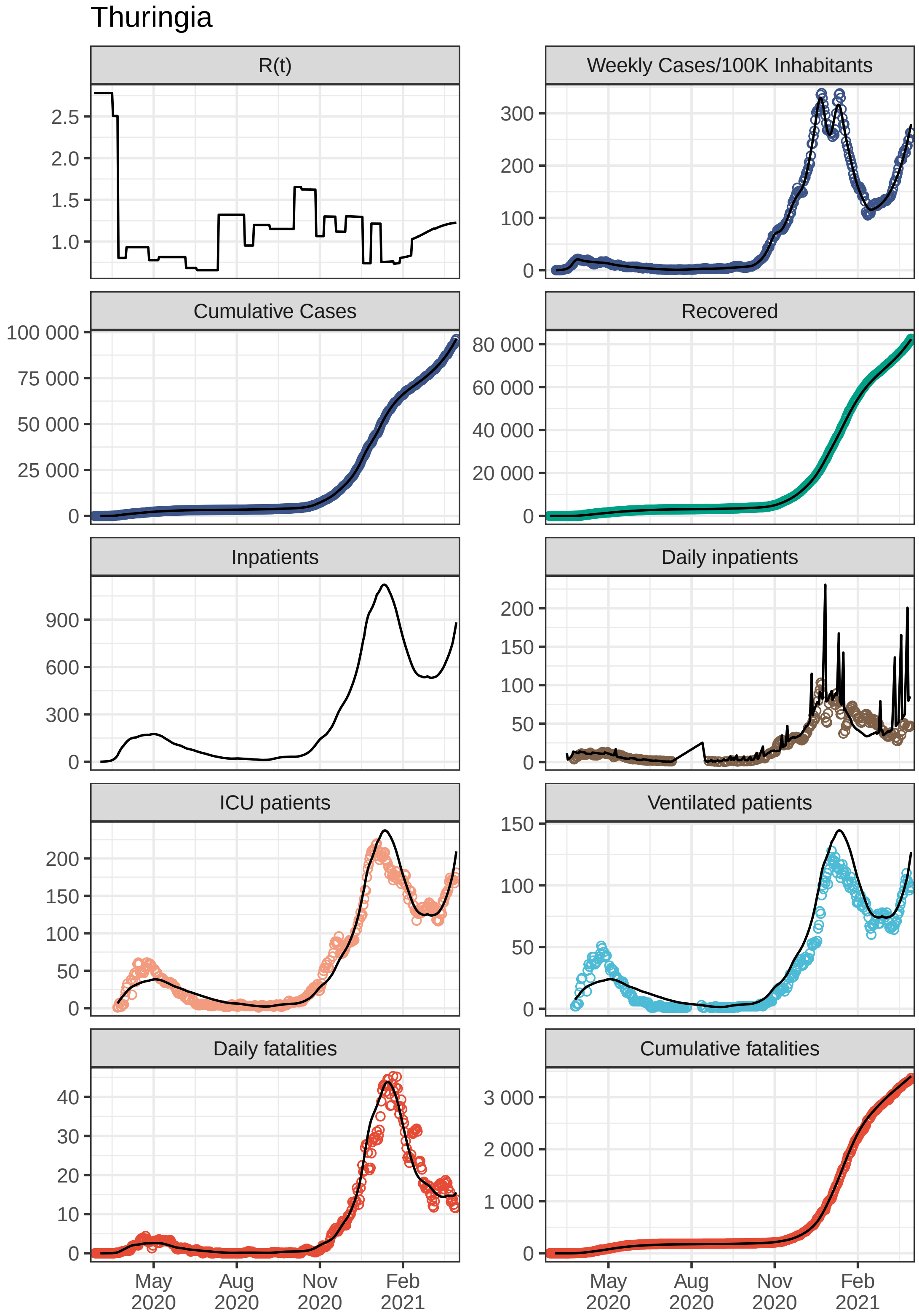
Thuringia
R(t)
2.5
2.0
1.5
1.0
Weekly Cases/100K Inhabitants
300
200
100
0
Cumulative Cases
100 000
75 000
50 000
25 000
0
Recovered
80 000
60 000
40 000
20 000
0
Inpatients
900
600
300
0
Daily inpatients
200
150
100
50
0
ICU patients
200
150
100
50
0
Ventilated patients
150
100
50
0
Daily fatalities
40
30
20
10
0
Cumulative fatalities
3 000
2 000
1 000
0
May 2020
Aug 2020
Nov 2020
Feb 2021

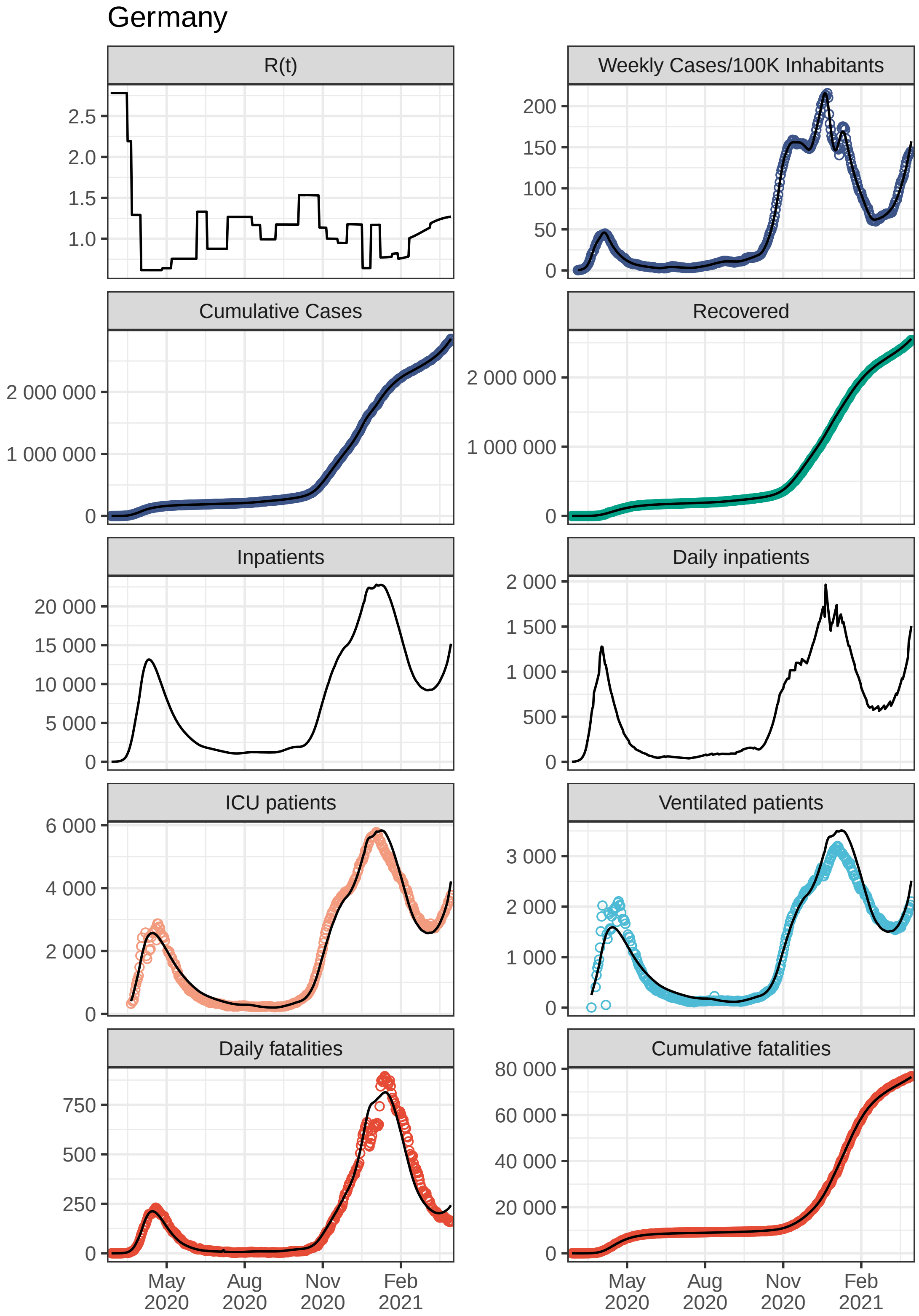
Germany
R(t)
Weekly Cases/100K Inhabitants
Cumulative Cases
Recovered
Inpatients
Daily inpatients
ICU patients
Ventilated patients
Daily fatalities
Cumulative fatalities
May 2020
Aug 2020
Nov 2020
Feb 2021
