# Supplementary material for: Model-Based Analysis of SARS-CoV-2 Infections, Hospitalization and Outcome in Germany, the Federal States and Districts
Source: Viruses. 2022 Sep 24;14(10):2114. doi: 10.3390/v14102114 (PMC9607468; doi:10.3390/v14102114)

LK Ahrweiler

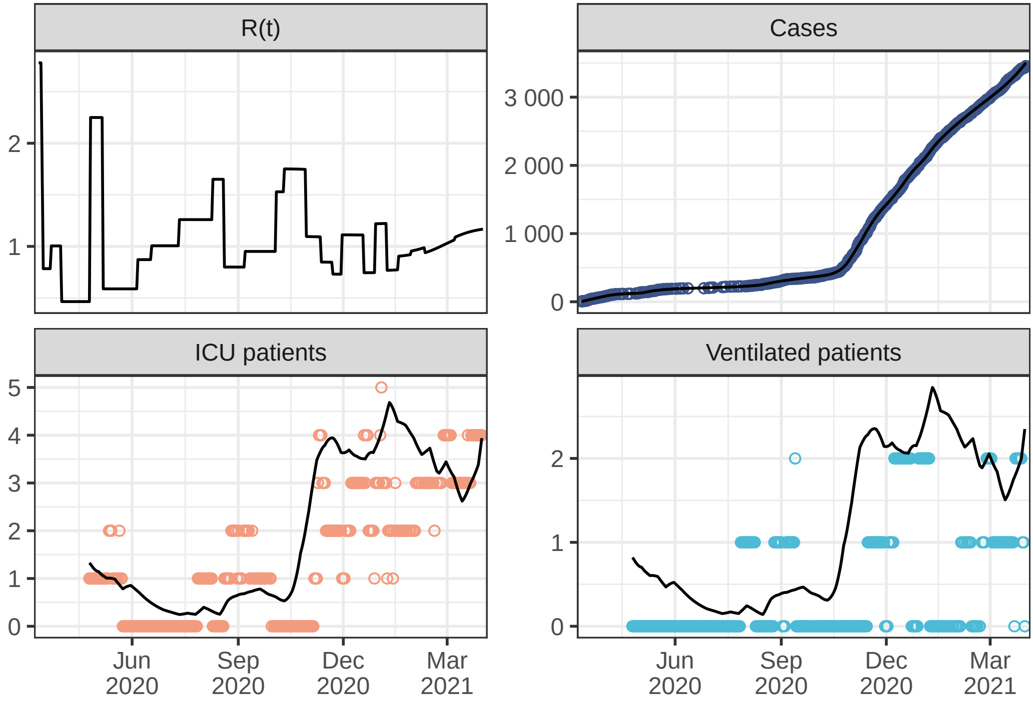

LK Aichach−Friedberg

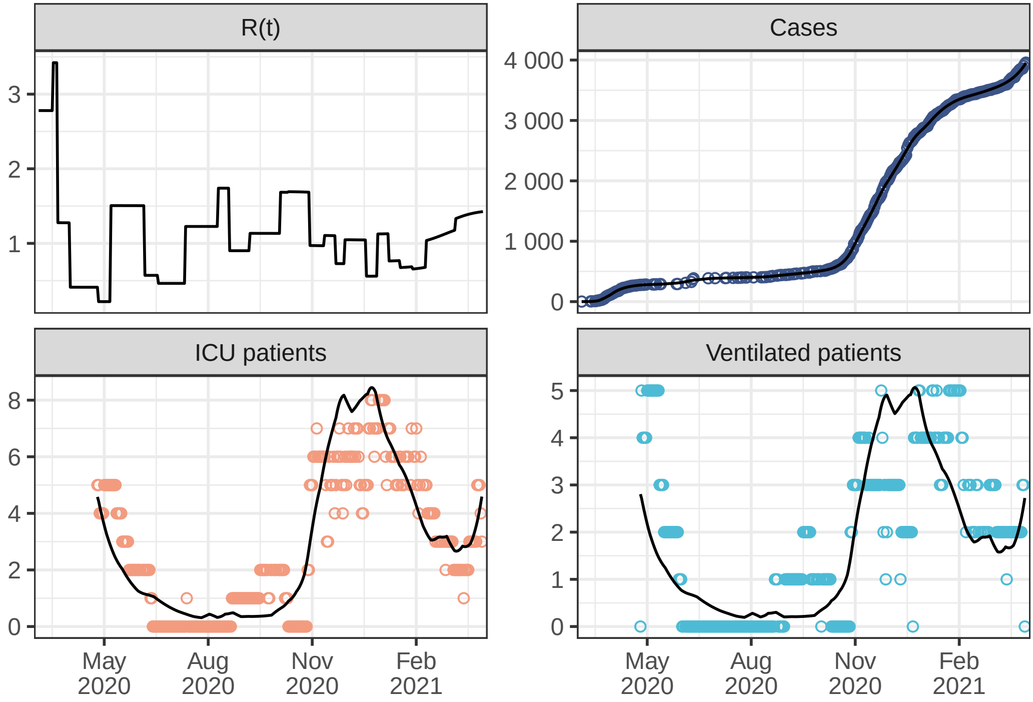

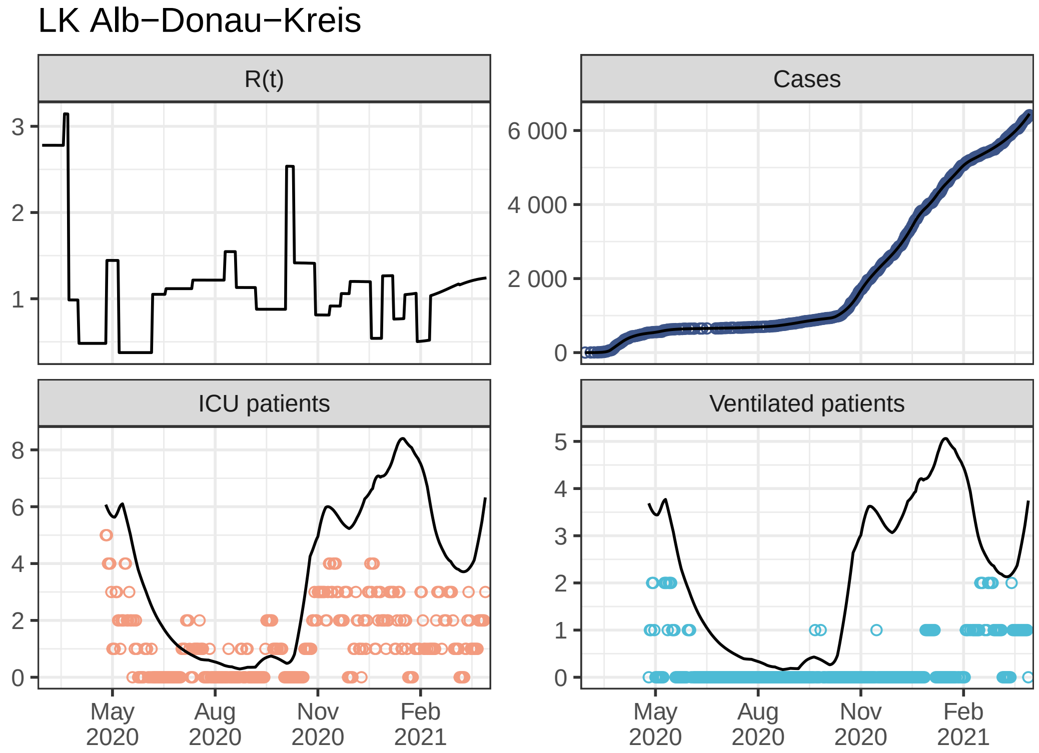
LK Alb−Donau−Kreis
R(t)
Cases
ICU patients
Ventilated patients
3
2
1
6 000
4 000
2 000
0
8
6
4
2
0
5
4
3
2
1
0
May 2020
Aug 2020
Nov 2020
Feb 2021

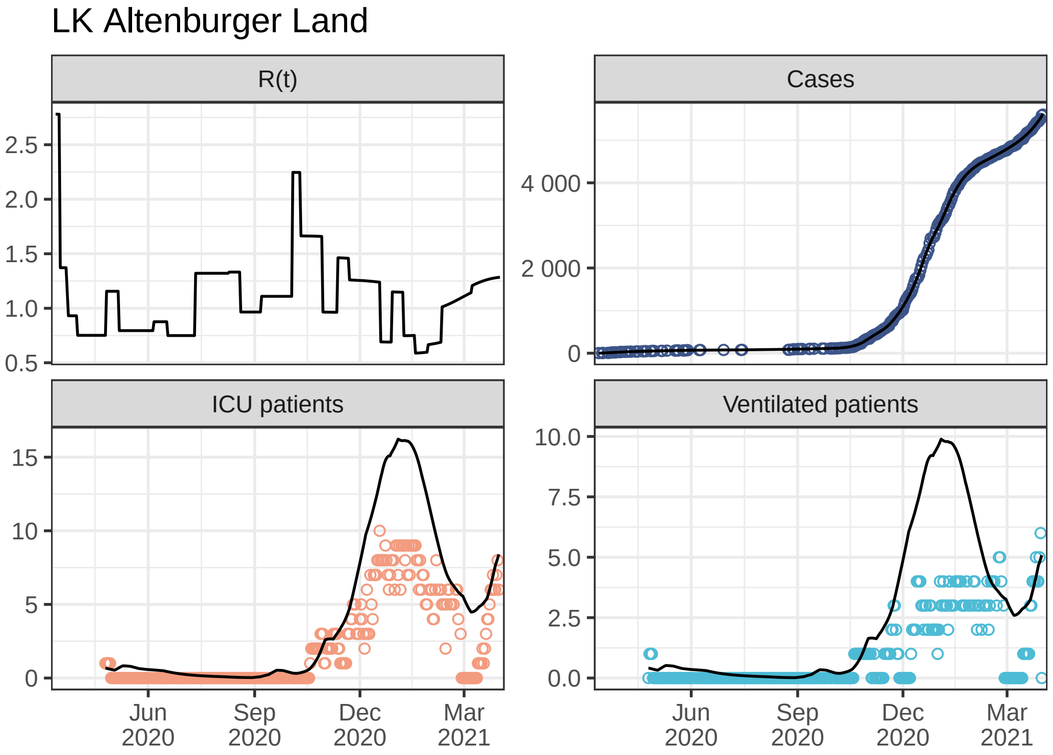
LK Altenburger Land
R(t)
Cases
ICU patients
Ventilated patients
2.5
2.0
1.5
1.0
0.5
4 000
2 000
0
15
10
5
0
10.0
7.5
5.0
2.5
0.0
Jun 2020
Sep 2020
Dec 2020
Mar 2021

LK Altenkirchen

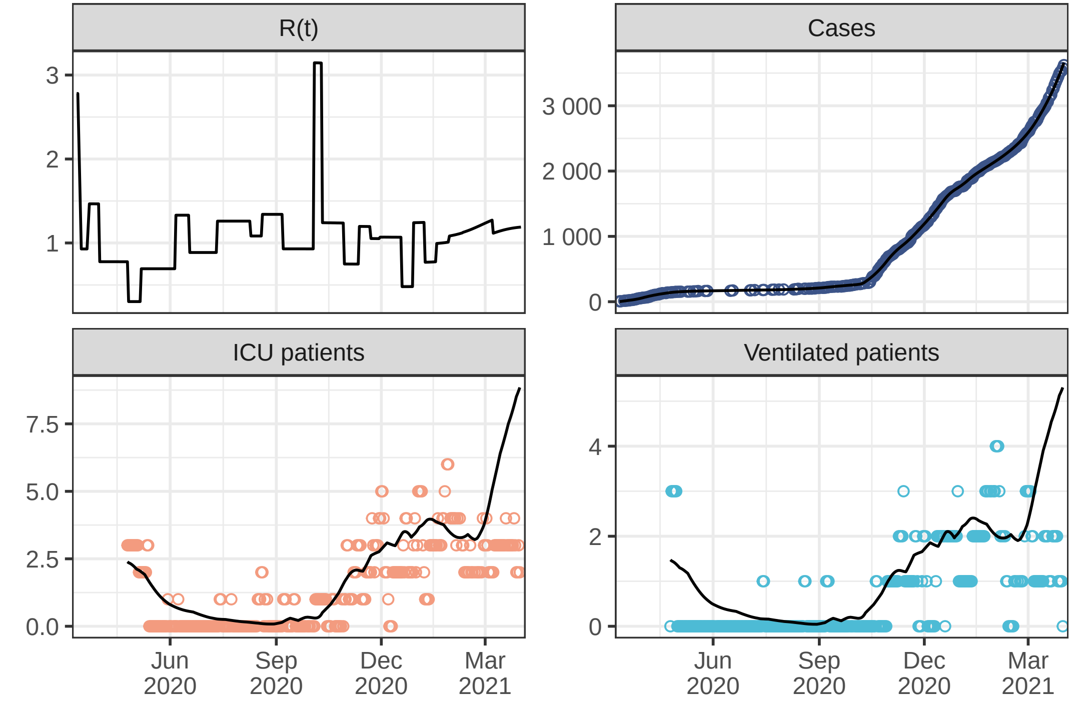

LK Altmarkkreis Salzwedel

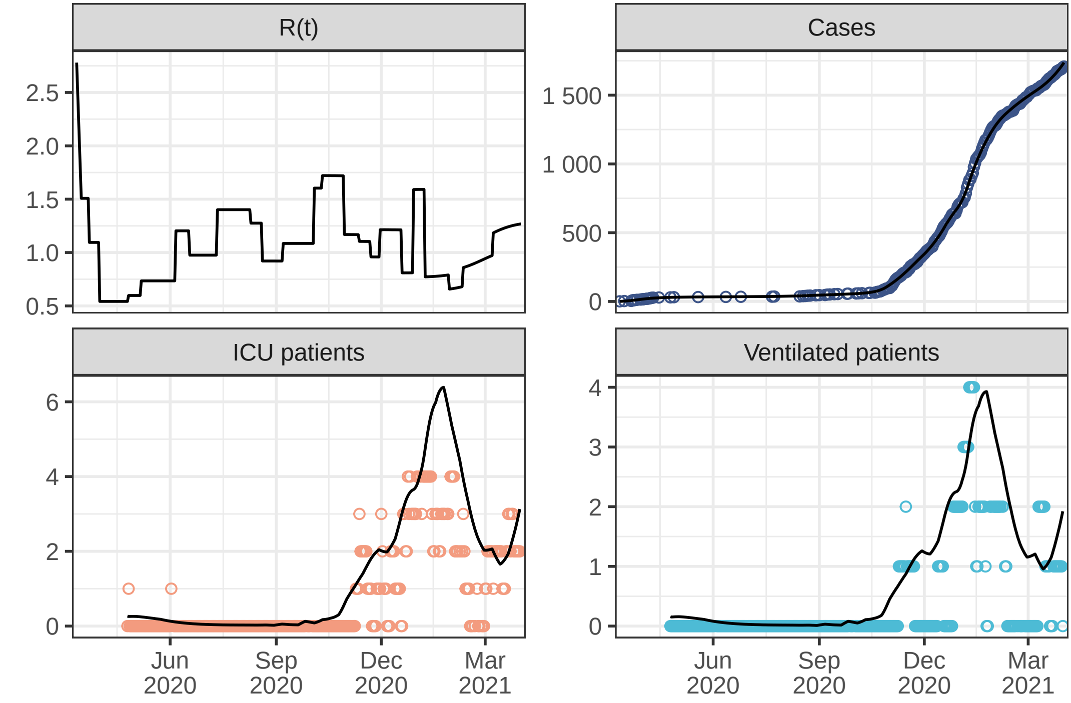

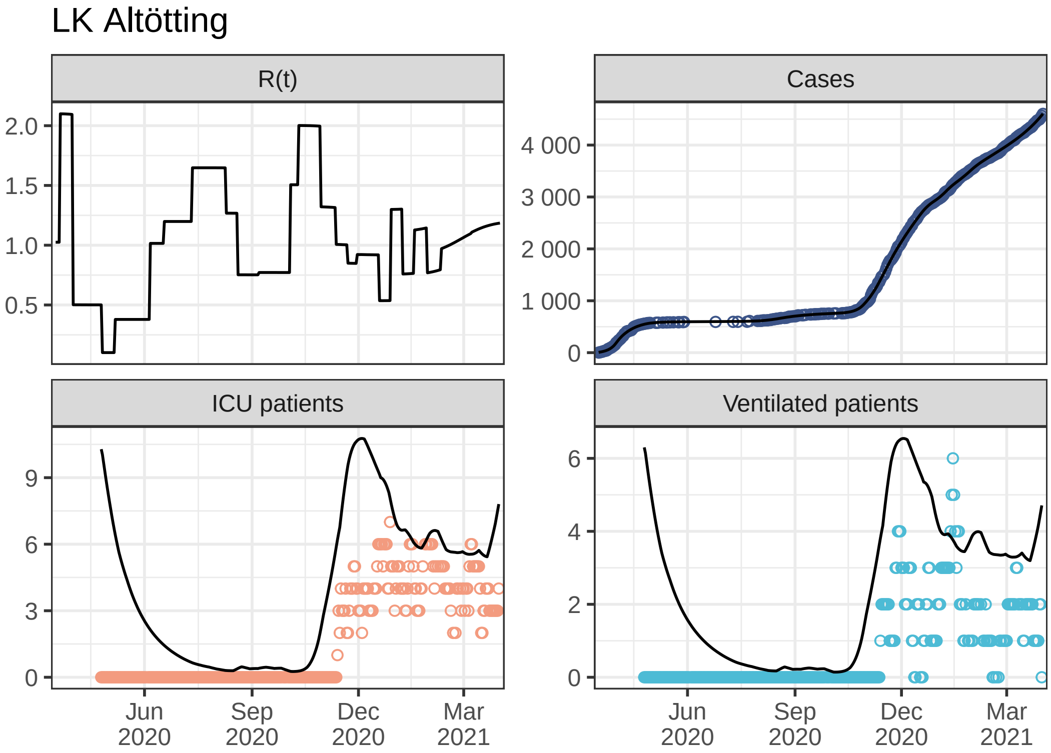
LK Altötting
R(t)
2.0
1.5
1.0
0.5
Cases
4 000
3 000
2 000
1 000
0
ICU patients
9
6
3
0
Ventilated patients
6
4
2
0
Jun 2020
Sep 2020
Dec 2020
Mar 2021

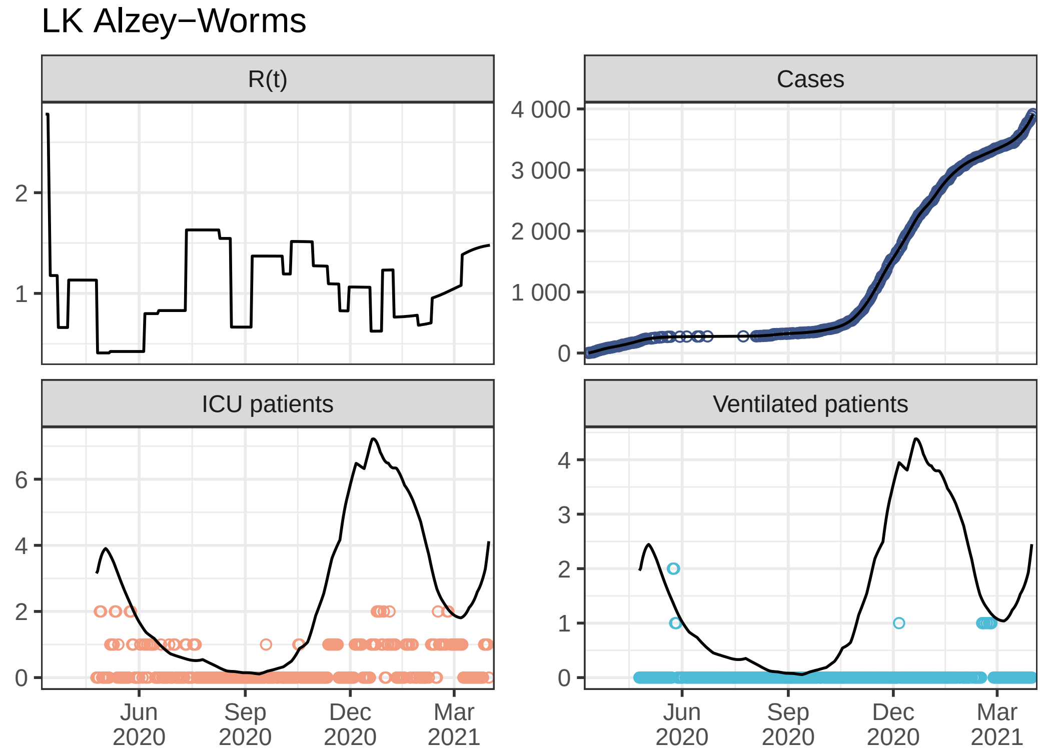
LK Alzey−Worms
R(t)
2
1
Cases
4 000
3 000
2 000
1 000
0
ICU patients
6
4
2
0
Ventilated patients
4
3
2
1
0
Jun 2020
Sep 2020
Dec 2020
Mar 2021

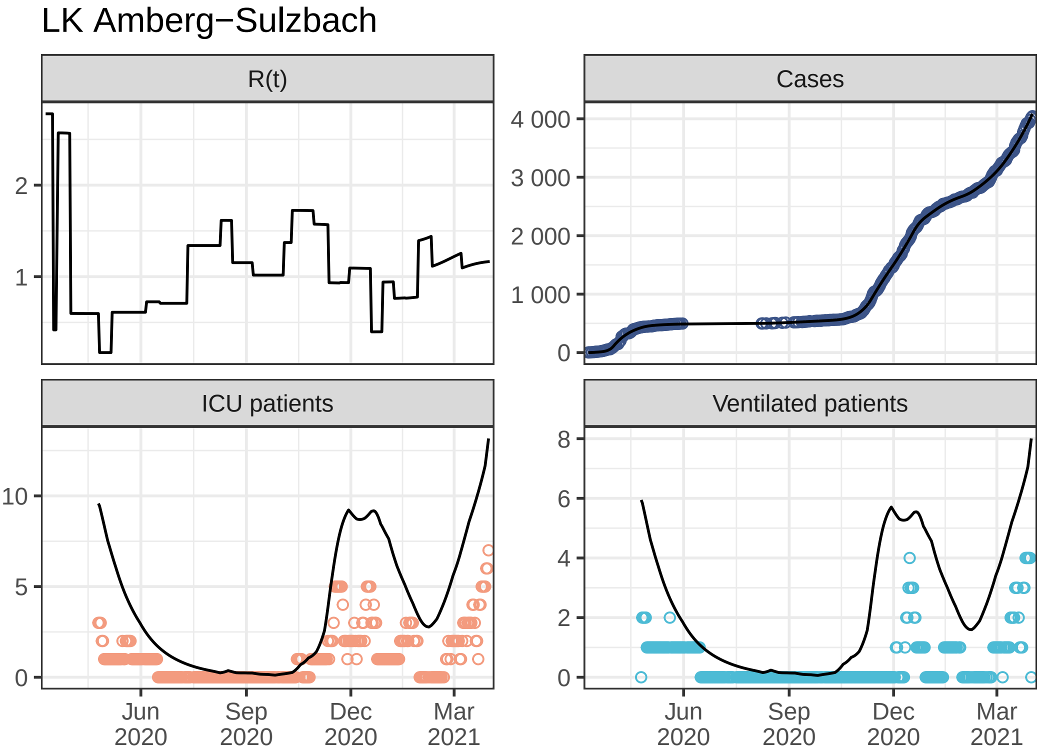
LK Amberg−Sulzbach
R(t)
Cases
ICU patients
Ventilated patients
2
1
4 000
3 000
2 000
1 000
0
10
5
0
8
6
4
2
0
Jun 2020
Sep 2020
Dec 2020
Mar 2021

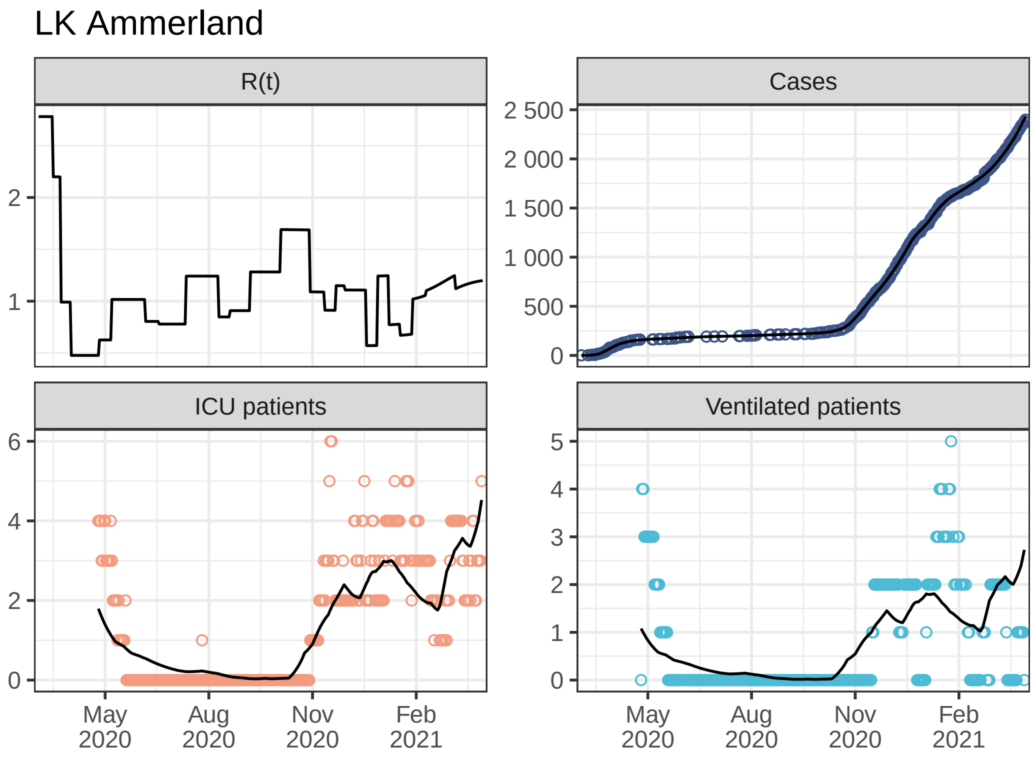
LK Ammerland
R(t)
Cases
ICU patients
Ventilated patients
2
1
2 500
2 000
1 500
1 000
500
0
6
4
2
0
5
4
3
2
1
0
May 2020
Aug 2020
Nov 2020
Feb 2021

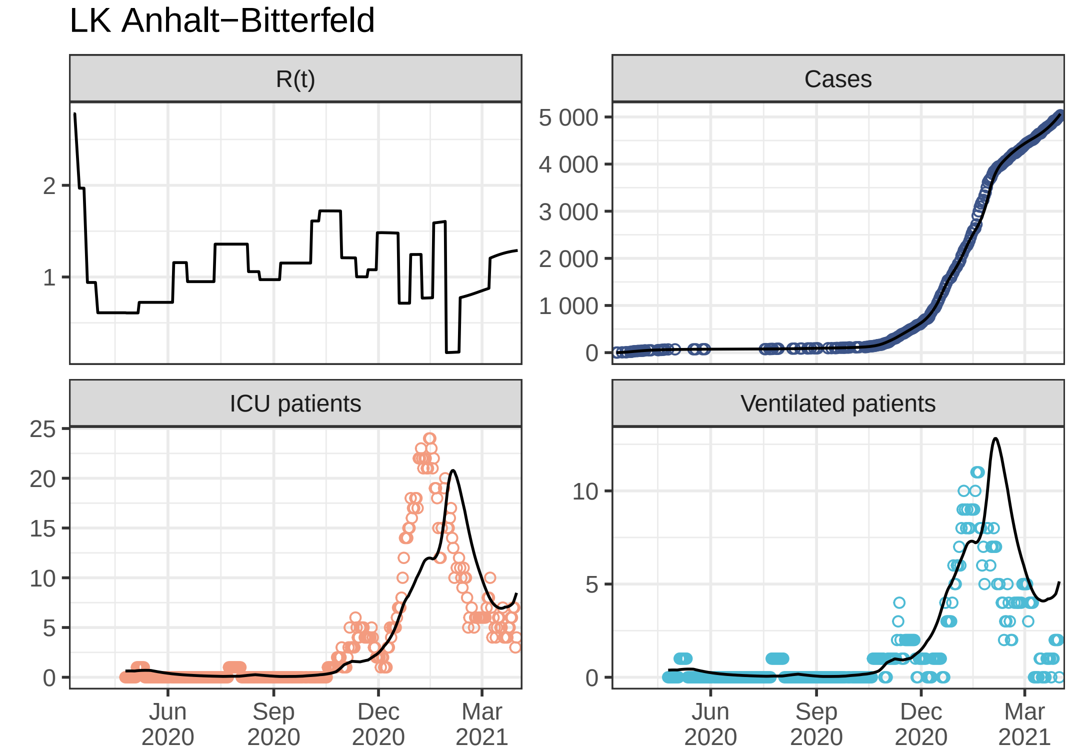
LK Anhalt−Bitterfeld
R(t)
Cases
ICU patients
Ventilated patients
2
1
5 000
4 000
3 000
2 000
1 000
0
25
20
15
10
5
0
10
5
0
Jun 2020
Sep 2020
Dec 2020
Mar 2021

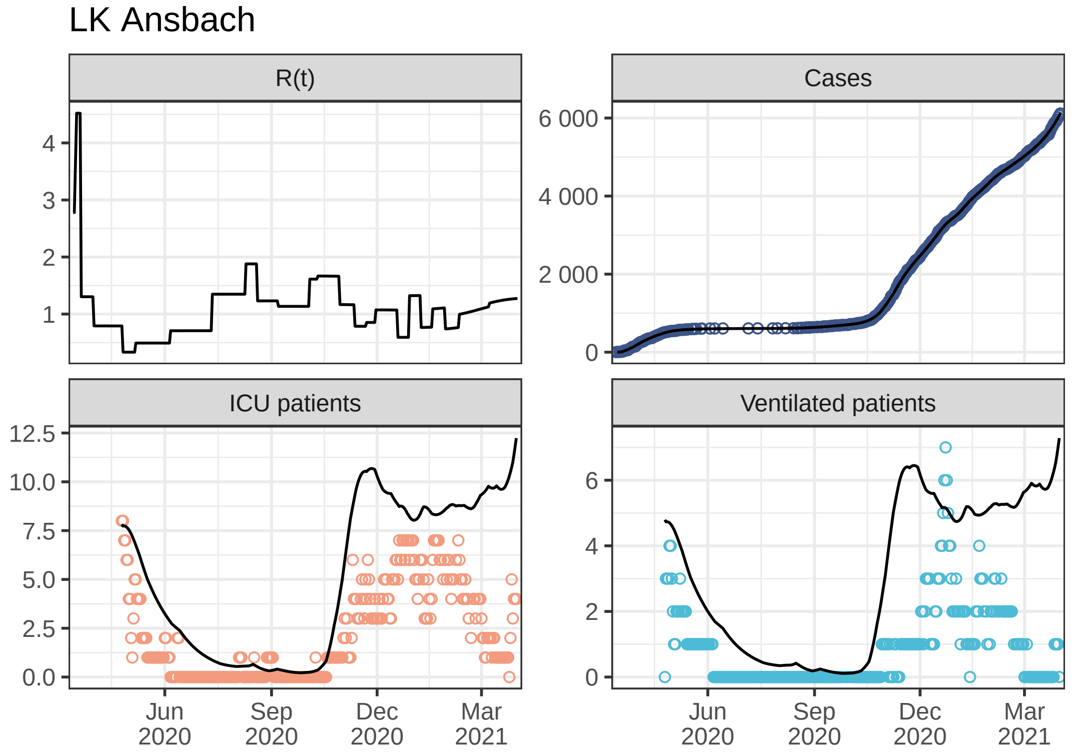
LK Ansbach
R(t)
Cases
ICU patients
Ventilated patients
4
3
2
1
6 000
4 000
2 000
0
12.5
10.0
7.5
5.0
2.5
0.0
6
4
2
0
Jun 2020
Sep 2020
Dec 2020
Mar 2021

LK Aschaffenburg

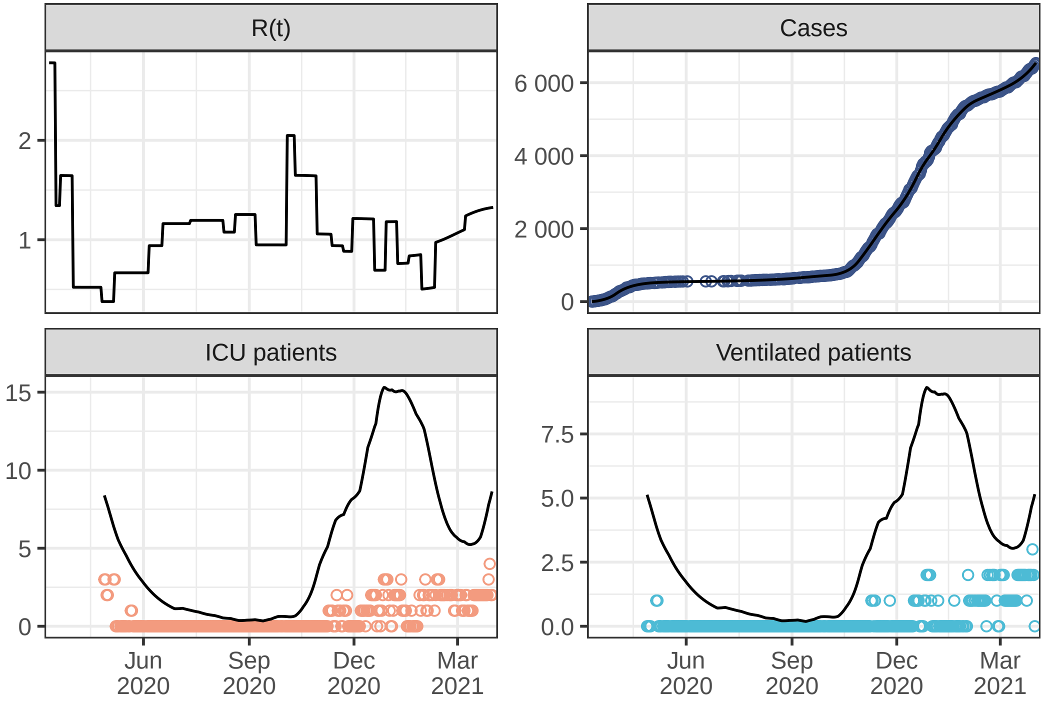

LK Augsburg

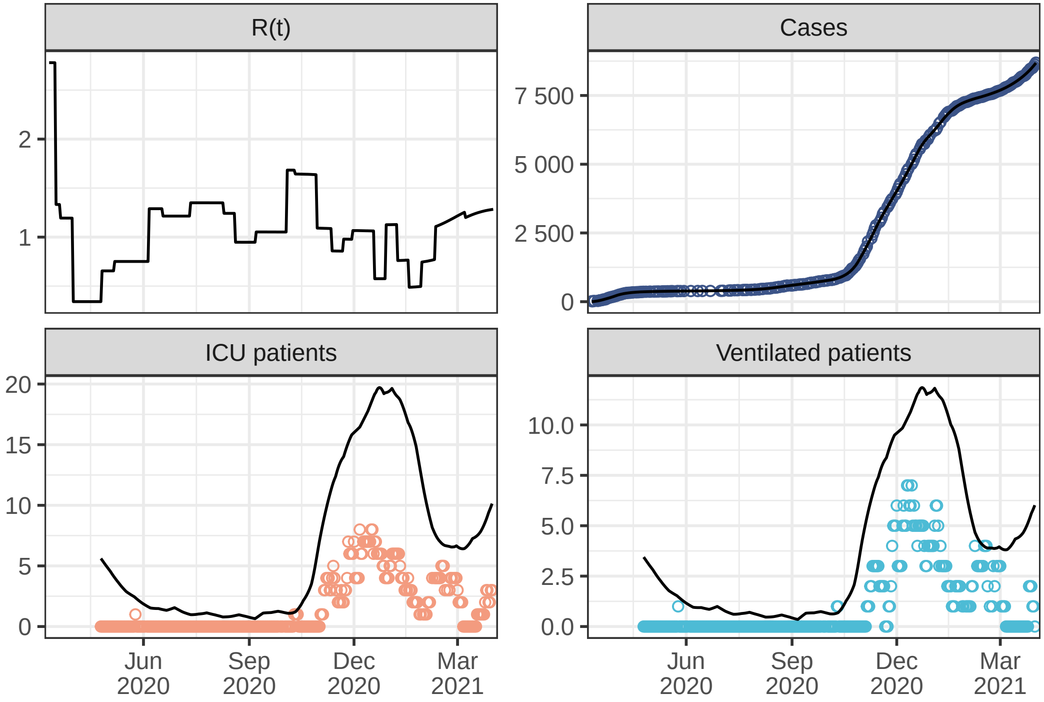

LK Aurich

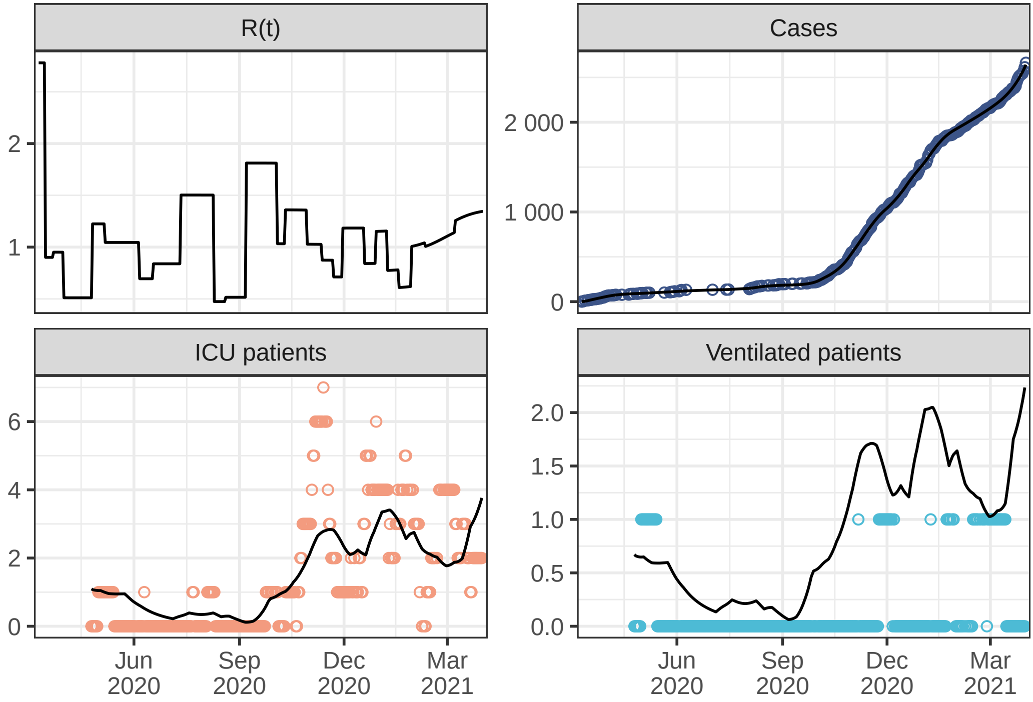

LK Bad Dürkheim

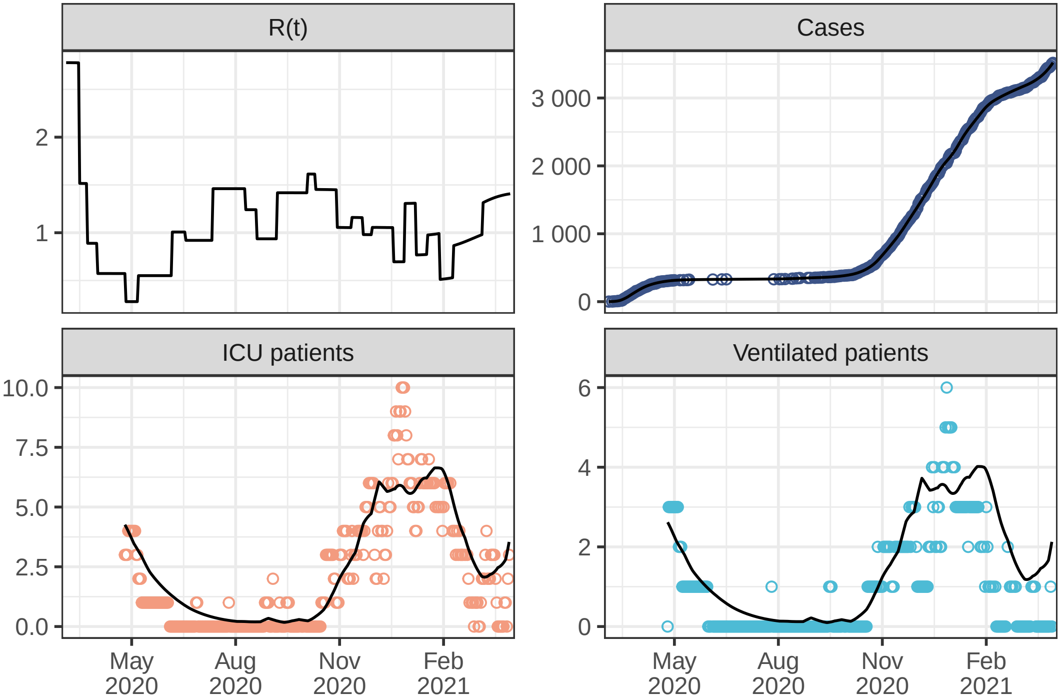

LK Bad Kissingen

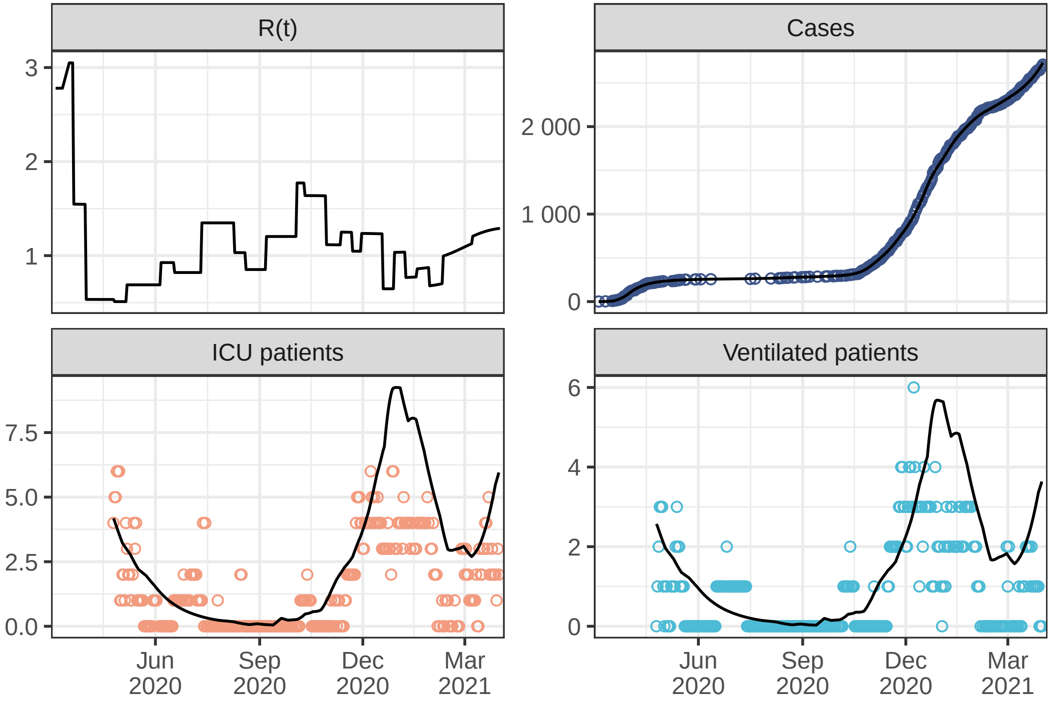

LK Bad Kreuznach

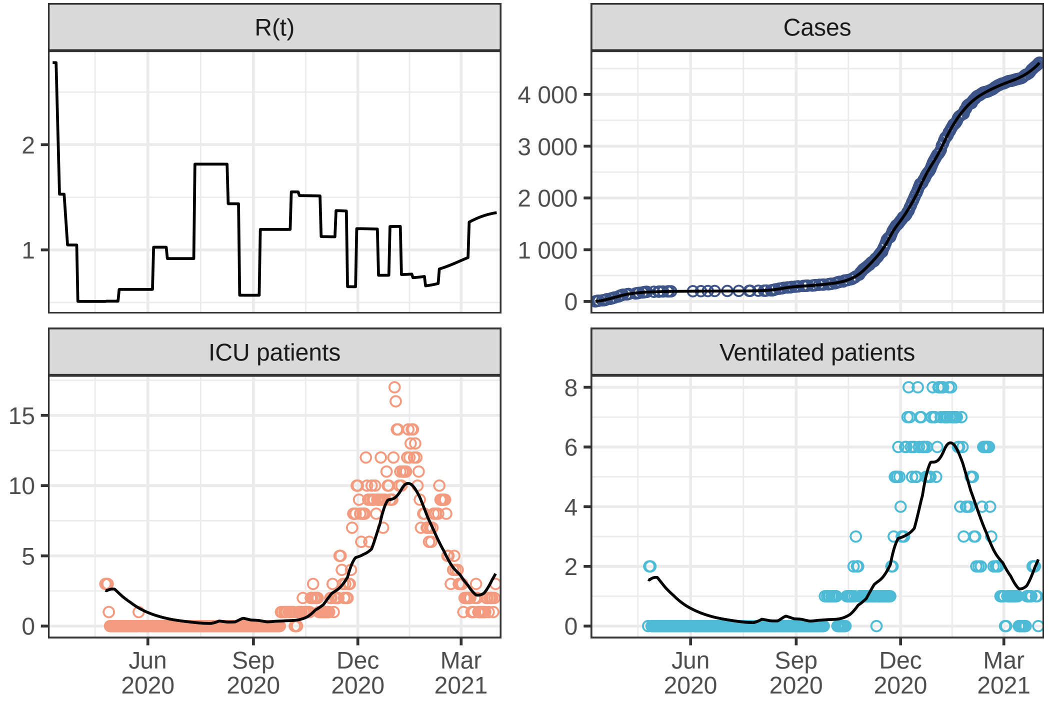

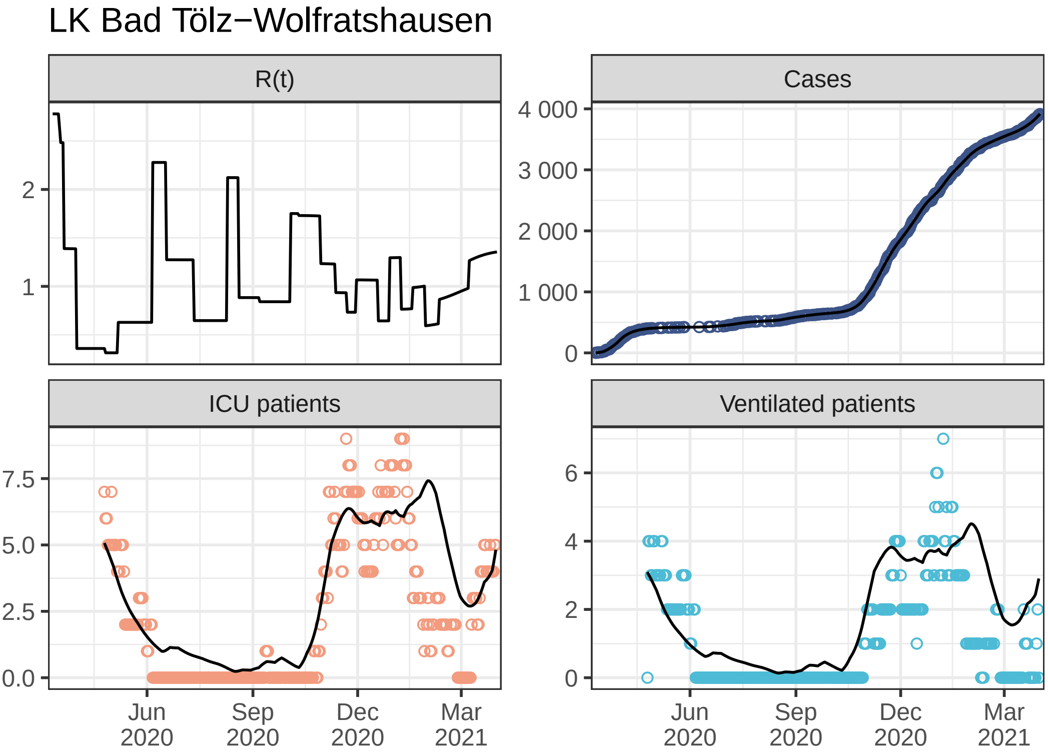
LK Bad Tölz−Wolfratshausen
R(t)
Cases
ICU patients
Ventilated patients
1
2
0
1 000
2 000
3 000
4 000
0.0
2.5
5.0
7.5
0
2
4
6
Jun 2020
Sep 2020
Dec 2020
Mar 2021

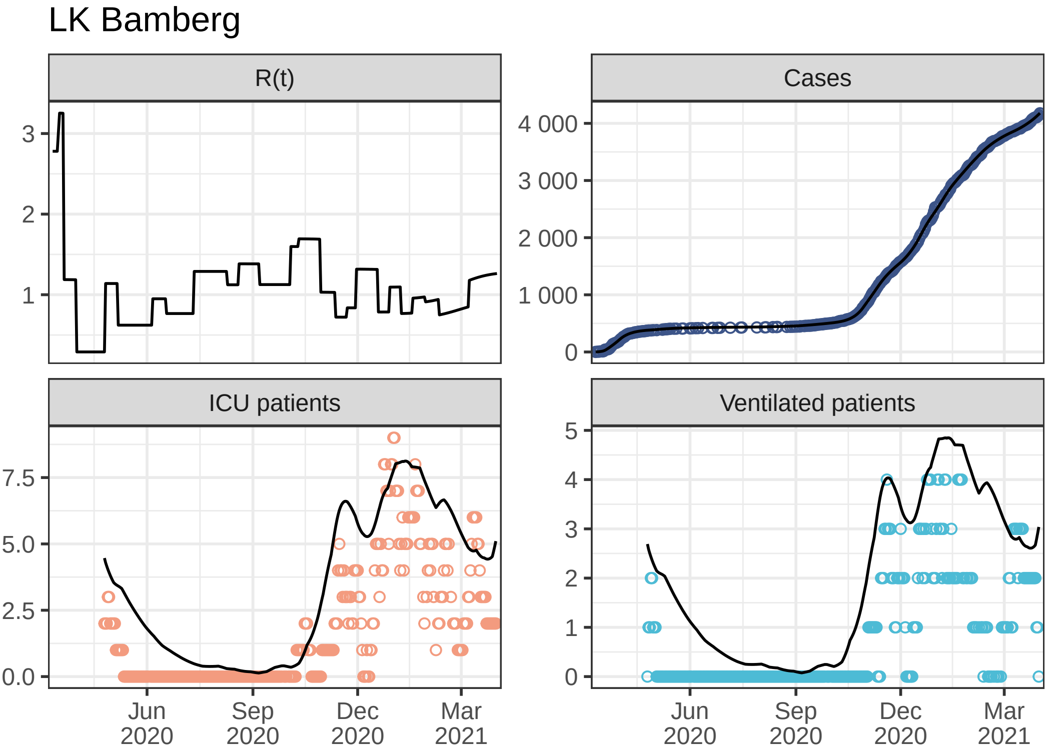
LK Bamberg
R(t)
Cases
ICU patients
Ventilated patients
1
2
3
0
1 000
2 000
3 000
4 000
0.0
2.5
5.0
7.5
0
1
2
3
4
5
Jun 2020
Sep 2020
Dec 2020
Mar 2021

LK Barnim

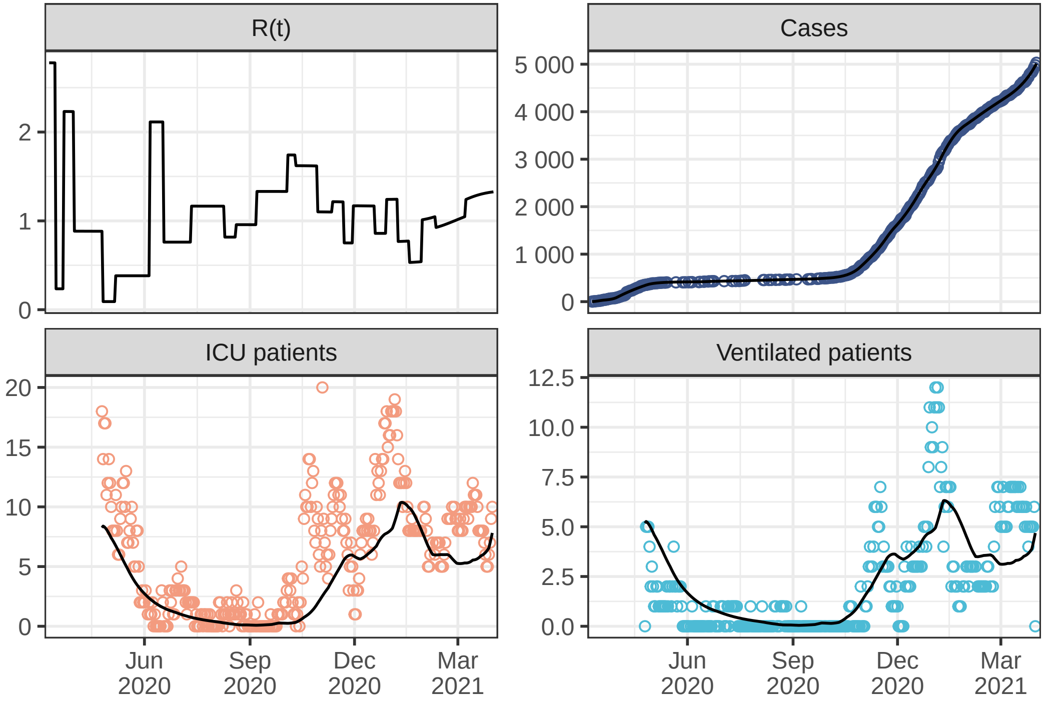

LK Bautzen

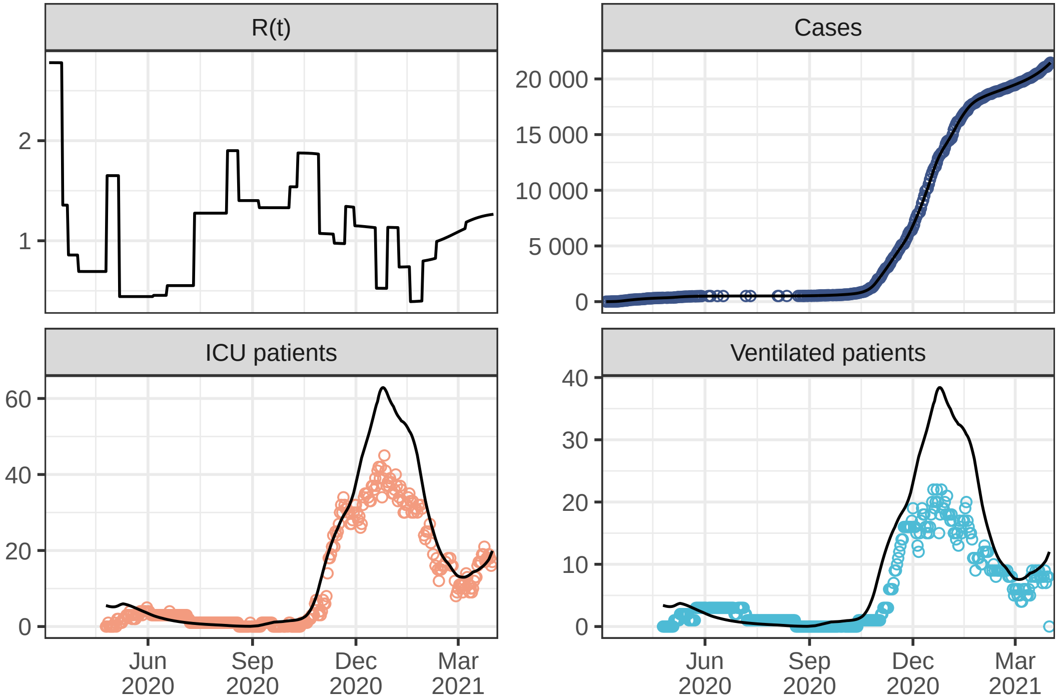

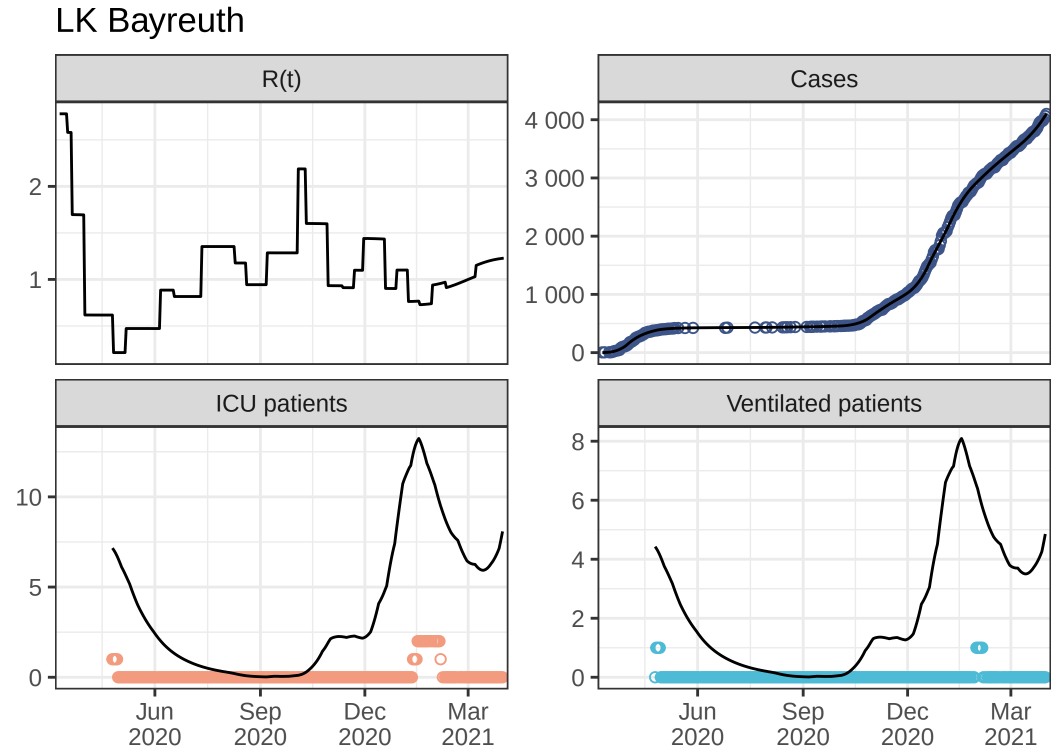
LK Bayreuth
R(t)
Cases
ICU patients
Ventilated patients
2
1
4 000
3 000
2 000
1 000
0
10
5
0
8
6
4
2
0
Jun 2020
Sep 2020
Dec 2020
Mar 2021

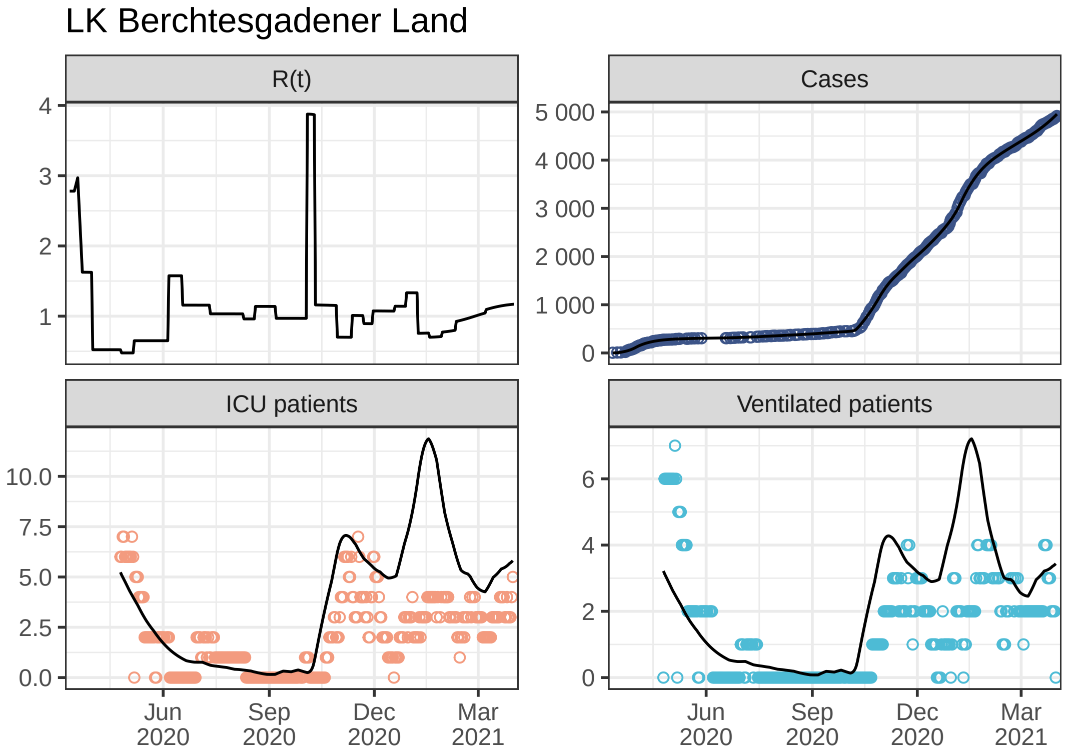
LK Berchtesgadener Land
R(t)
Cases
ICU patients
Ventilated patients
4
3
2
1
5 000
4 000
3 000
2 000
1 000
0
10.0
7.5
5.0
2.5
0.0
6
4
2
0
Jun 2020
Sep 2020
Dec 2020
Mar 2021

LK Bergstraße

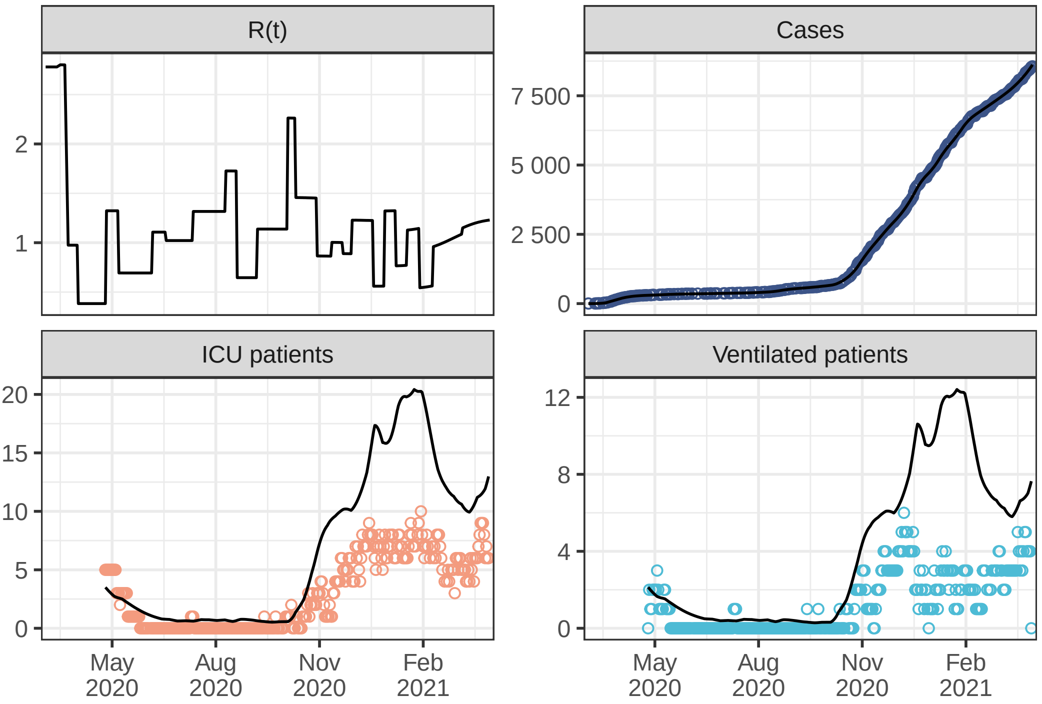

LK Bernkastel−Wittlich

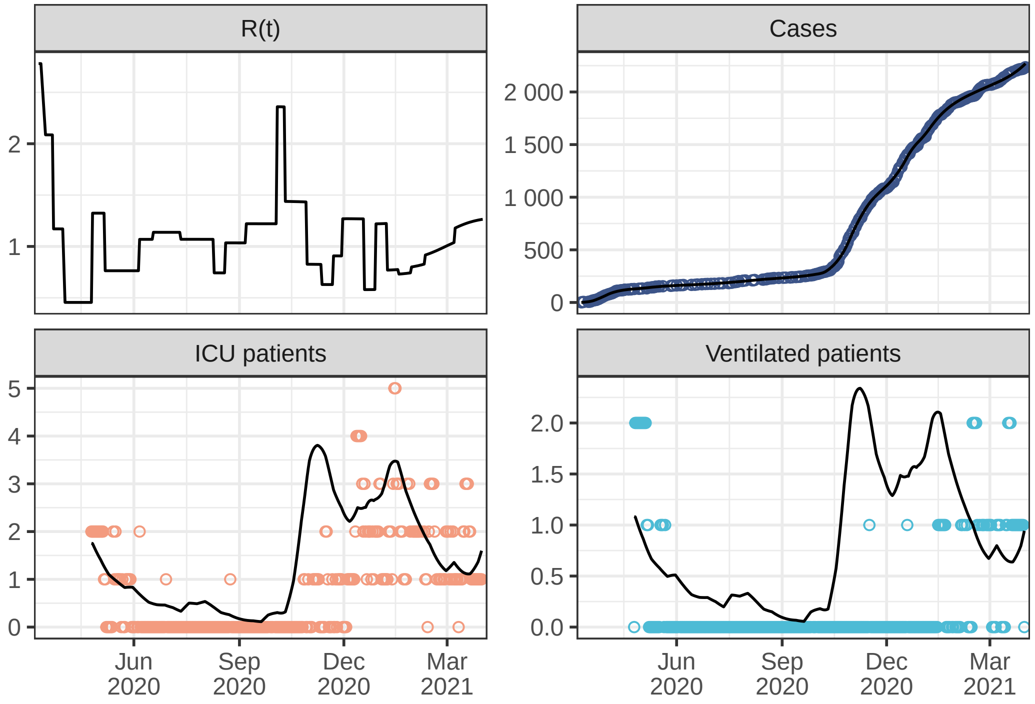

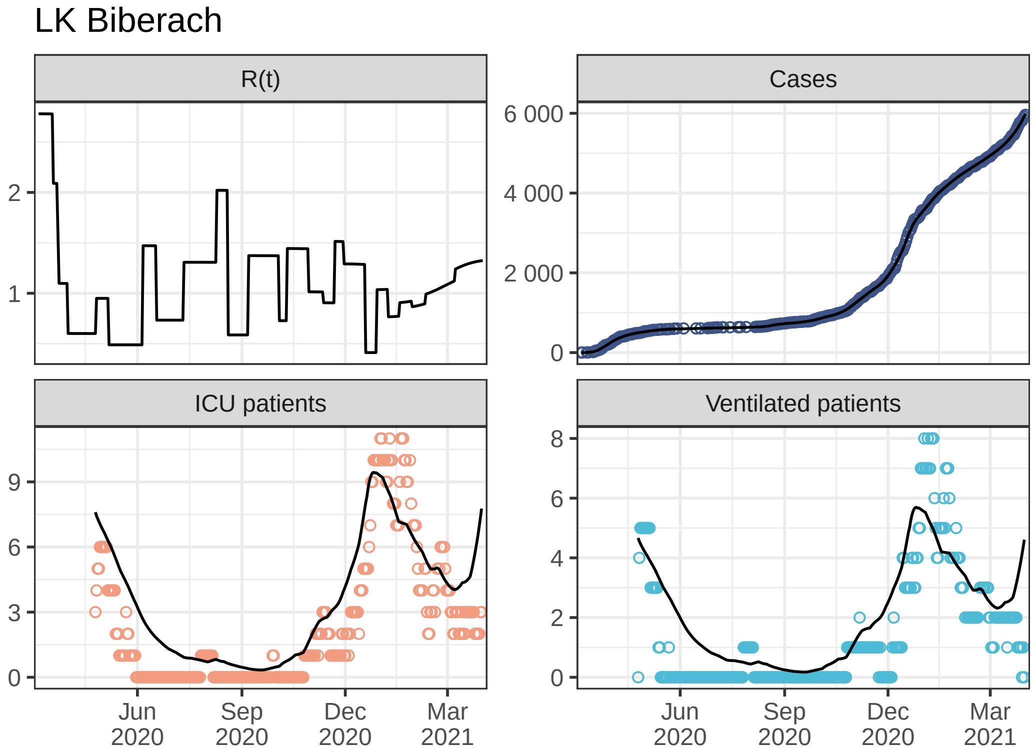
LK Biberach
R(t)
Cases
ICU patients
Ventilated patients
1
2
6 000
4 000
2 000
0
9
6
3
0
8
6
4
2
0
Jun 2020
Sep 2020
Dec 2020
Mar 2021

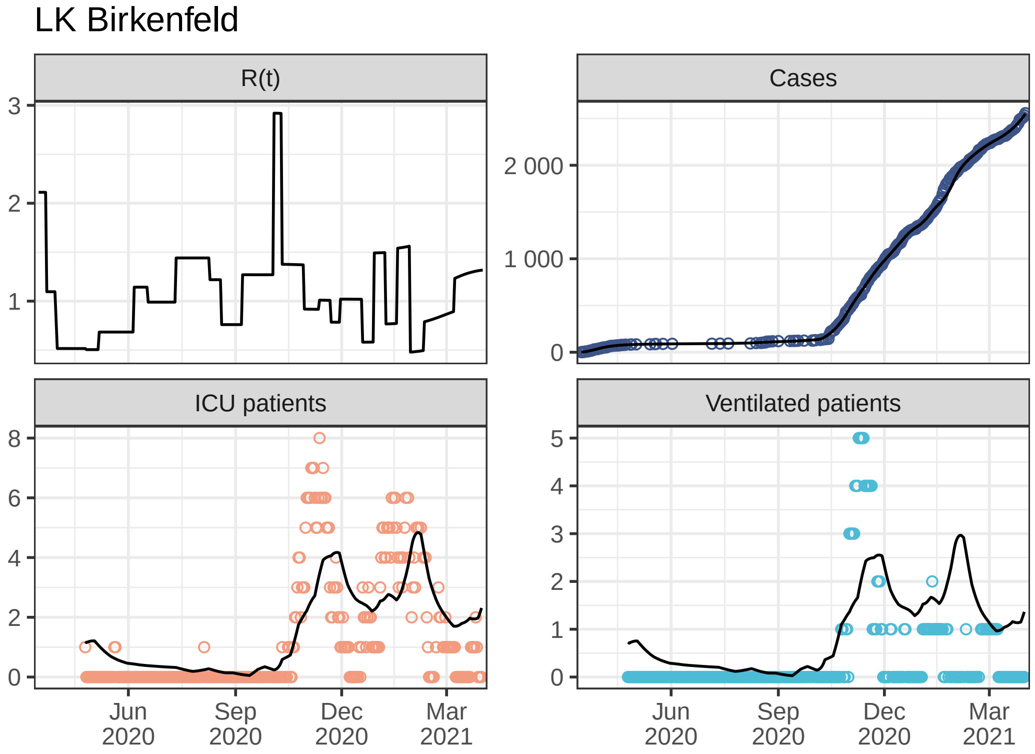
LK Birkenfeld
R(t)
Cases
ICU patients
Ventilated patients
3
2
1
2 000
1 000
0
8
6
4
2
0
5
4
3
2
1
0
Jun 2020
Sep 2020
Dec 2020
Mar 2021

LK Bitburg−Prüm

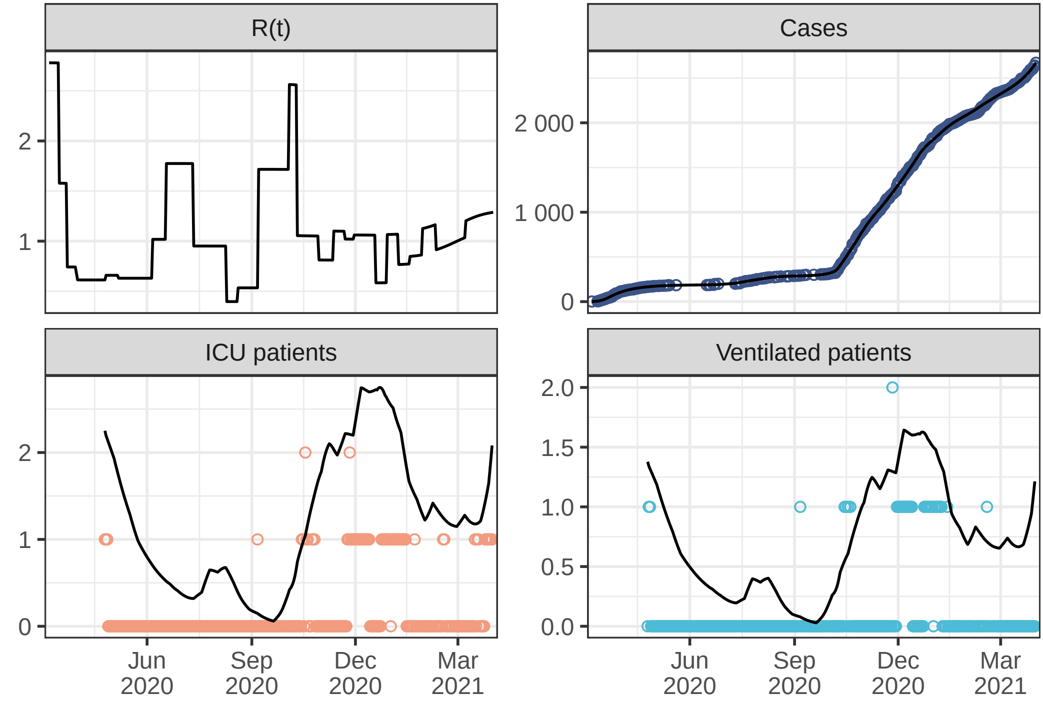

LK Böblingen

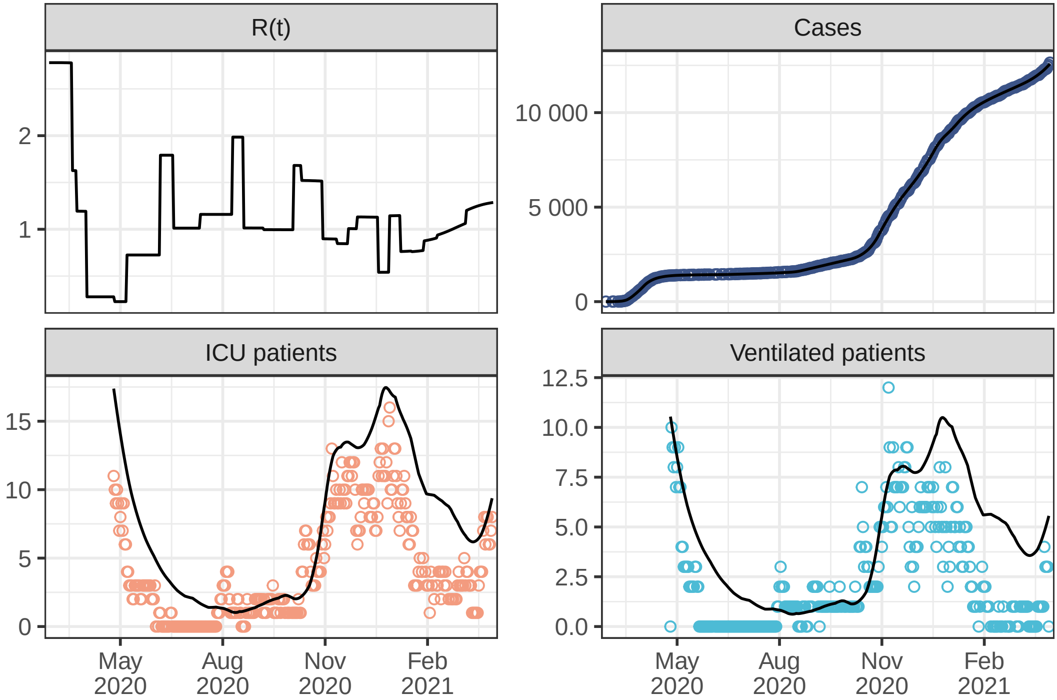

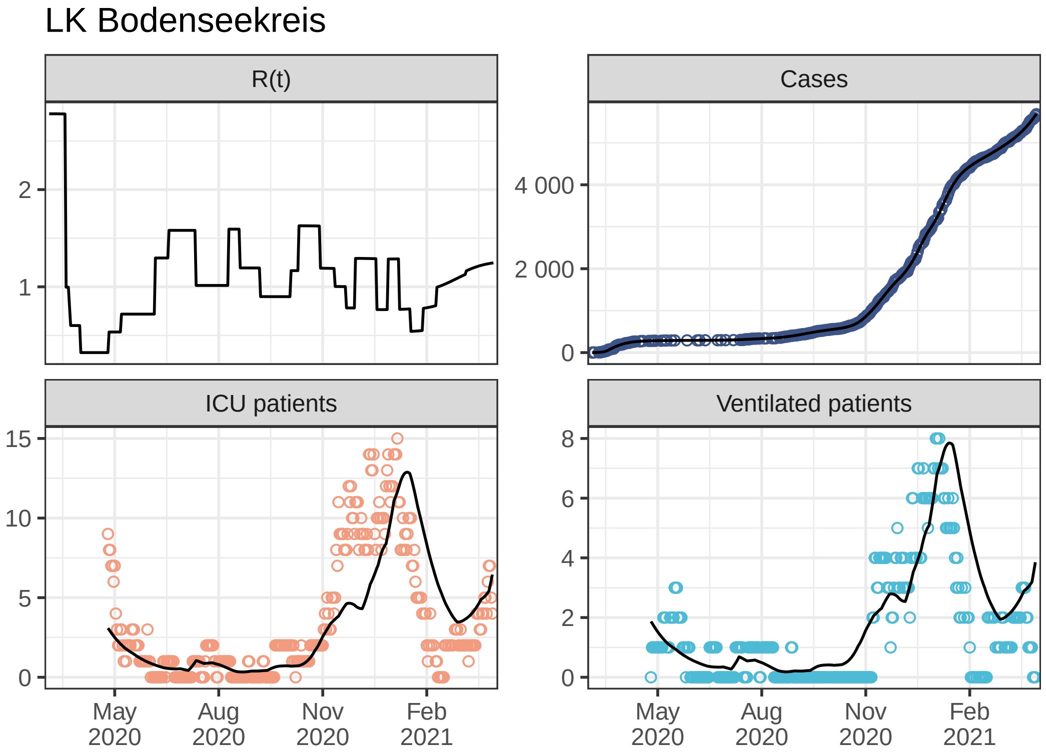
LK Bodenseekreis
R(t)
Cases
ICU patients
Ventilated patients
2
1
4 000
2 000
0
15
10
5
0
8
6
4
2
0
May 2020
Aug 2020
Nov 2020
Feb 2021

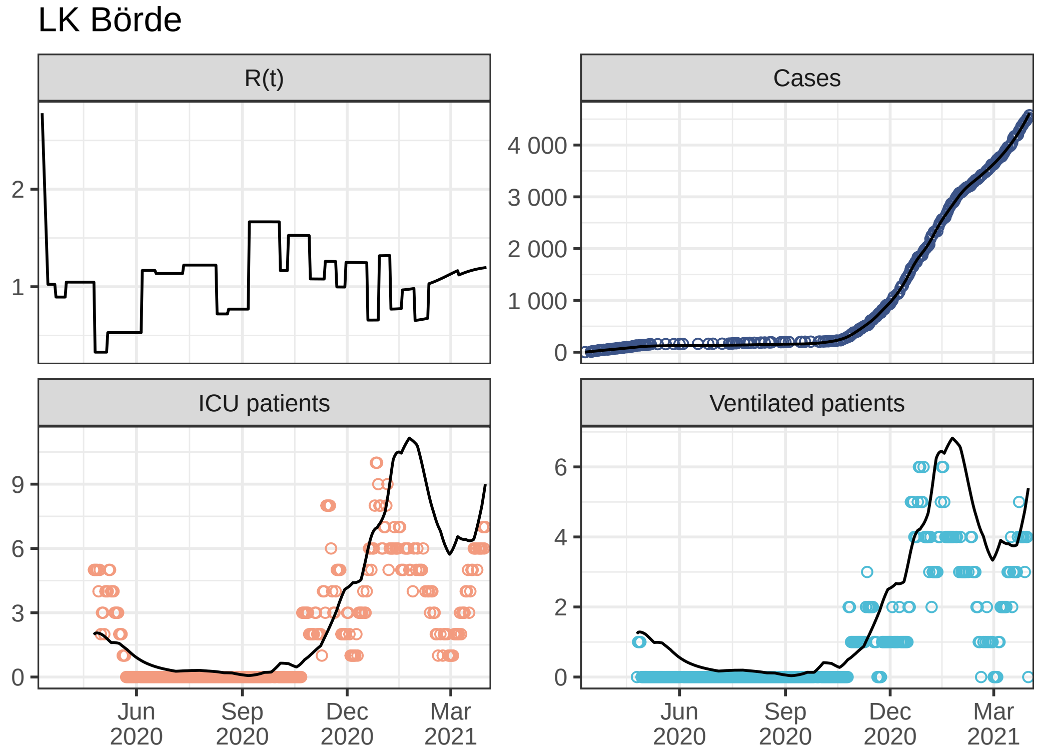
LK Börde
R(t)
Cases
ICU patients
Ventilated patients
2
1
4 000
3 000
2 000
1 000
0
9
6
3
0
6
4
2
0
Jun 2020
Sep 2020
Dec 2020
Mar 2021

LK Borken

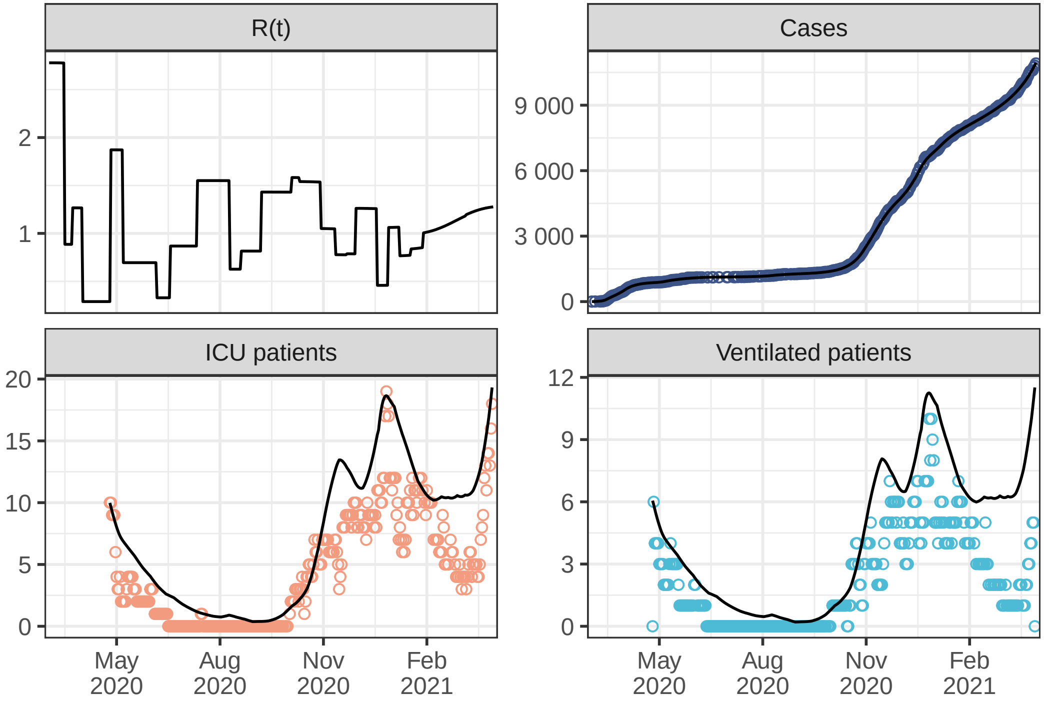

LK Breisgau−Hochschwarzwald

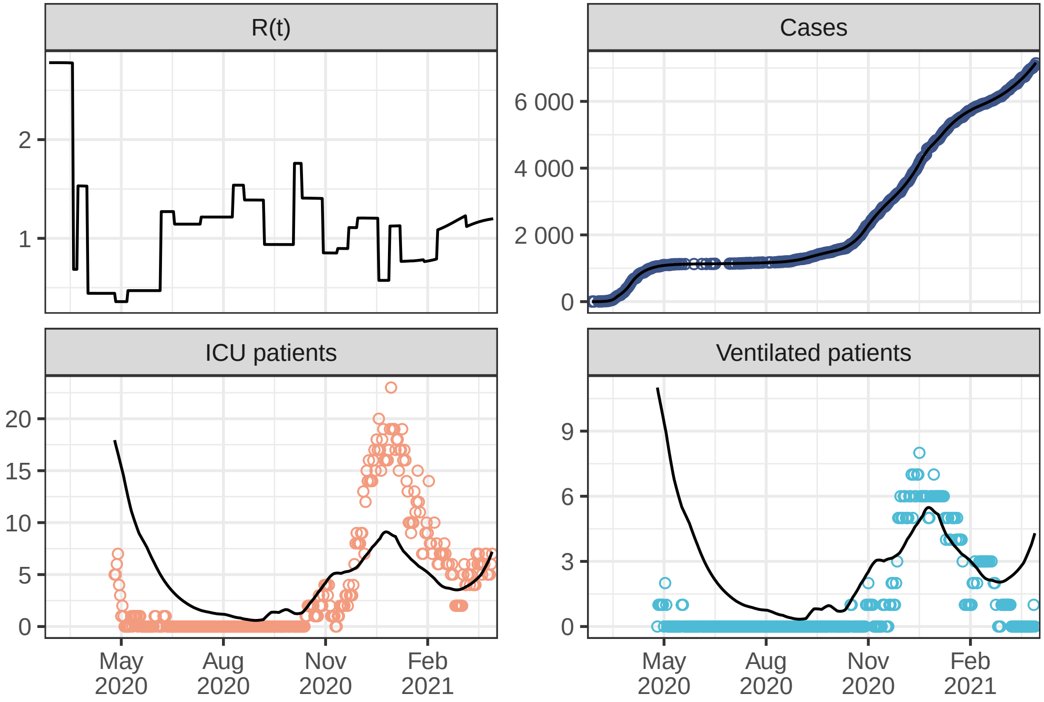

LK Burgenlandkreis

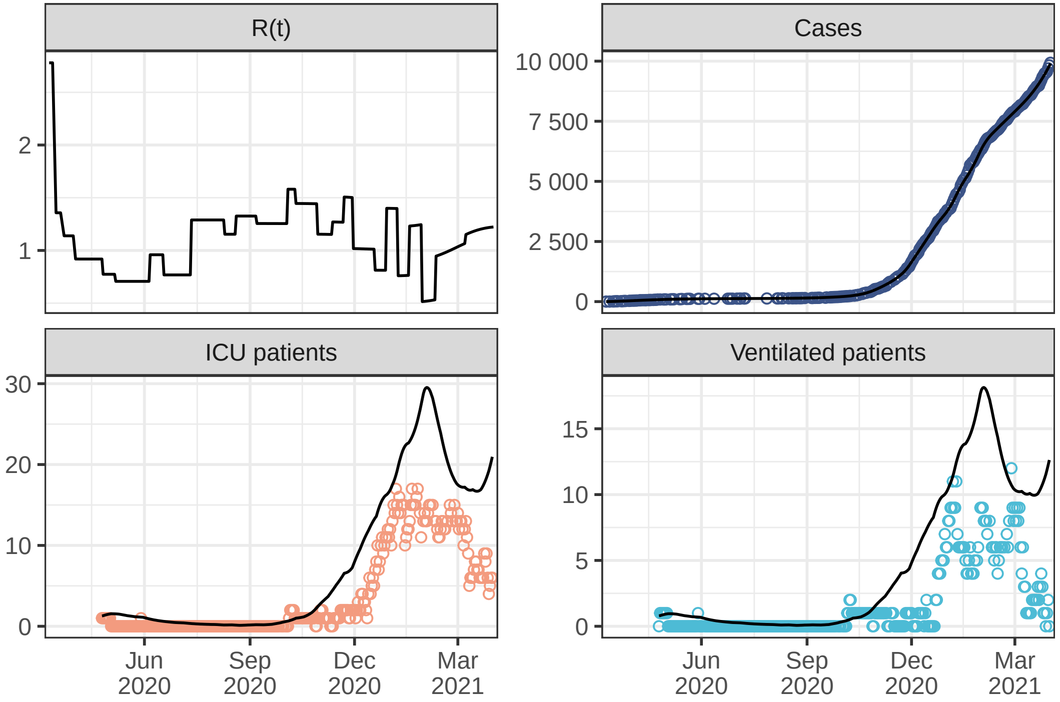

LK Calw

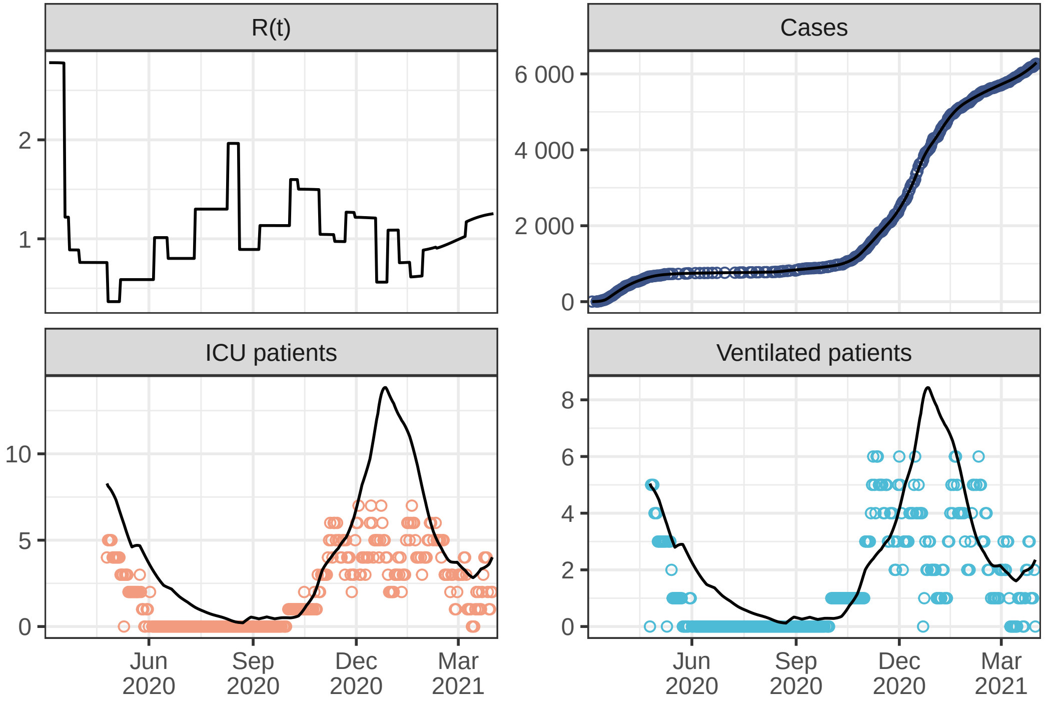

LK Celle

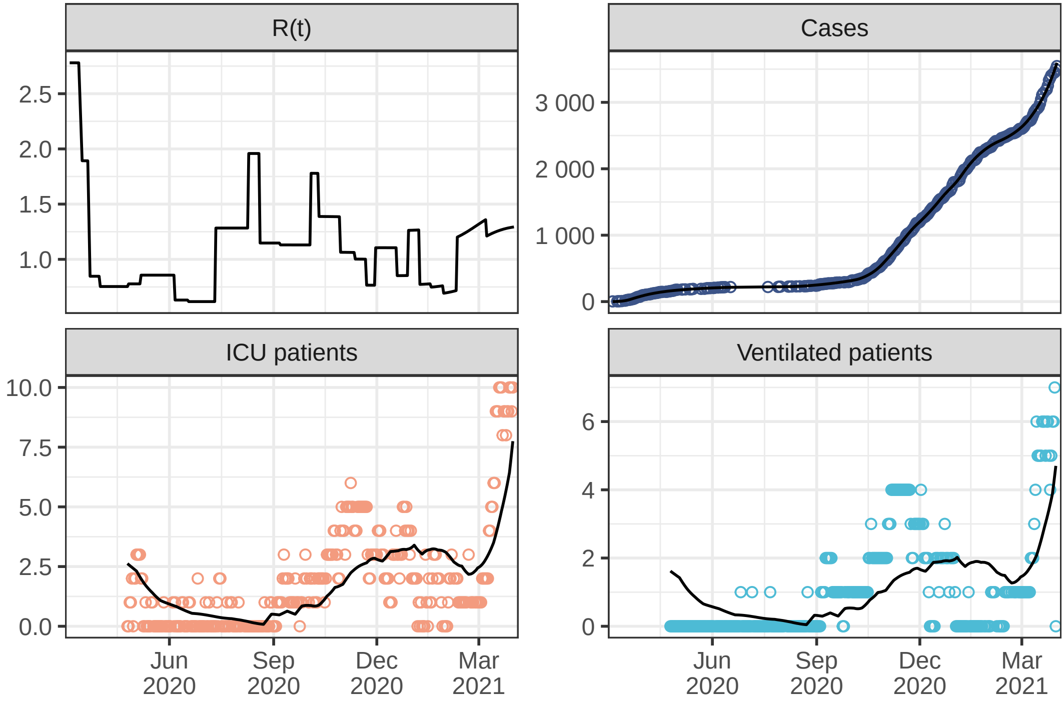

LK Cham

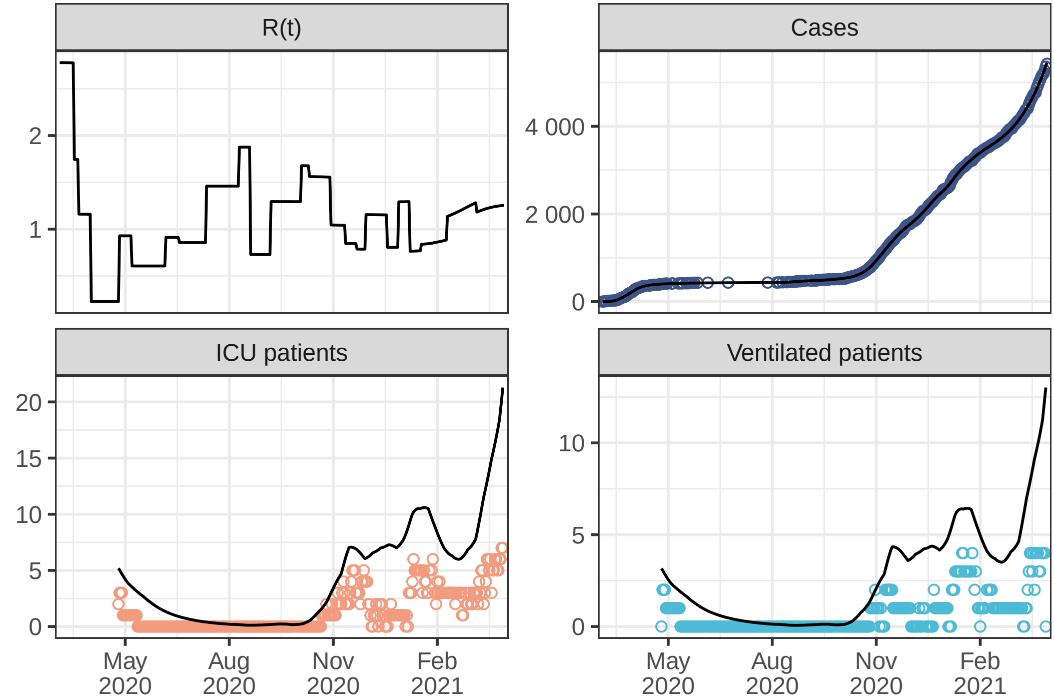

LK Cloppenburg

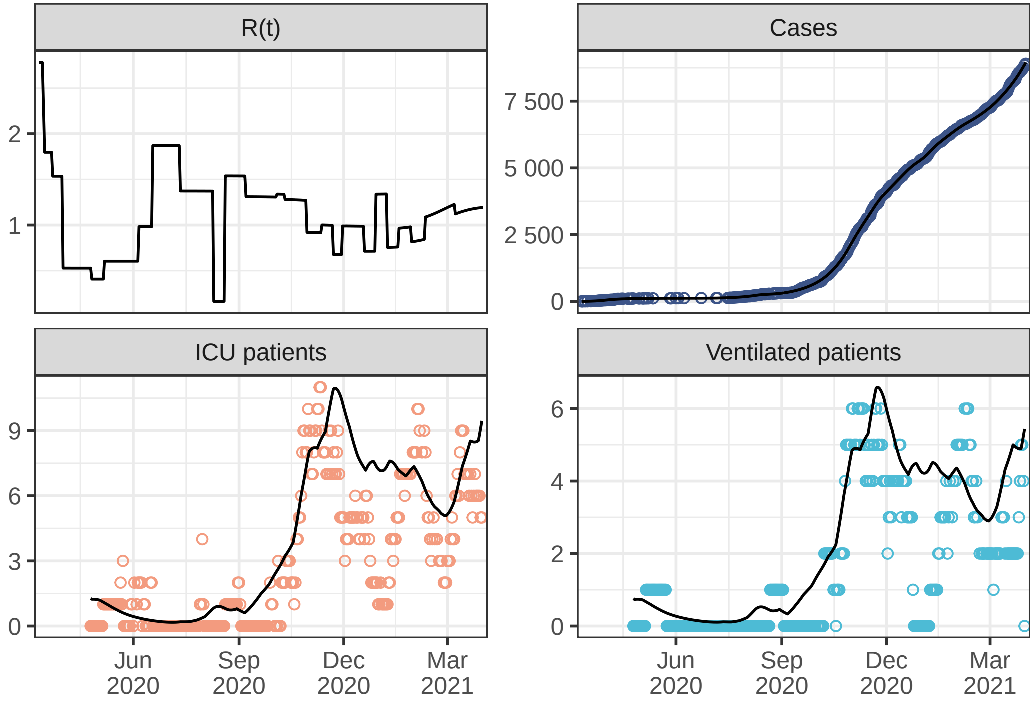

LK Coburg

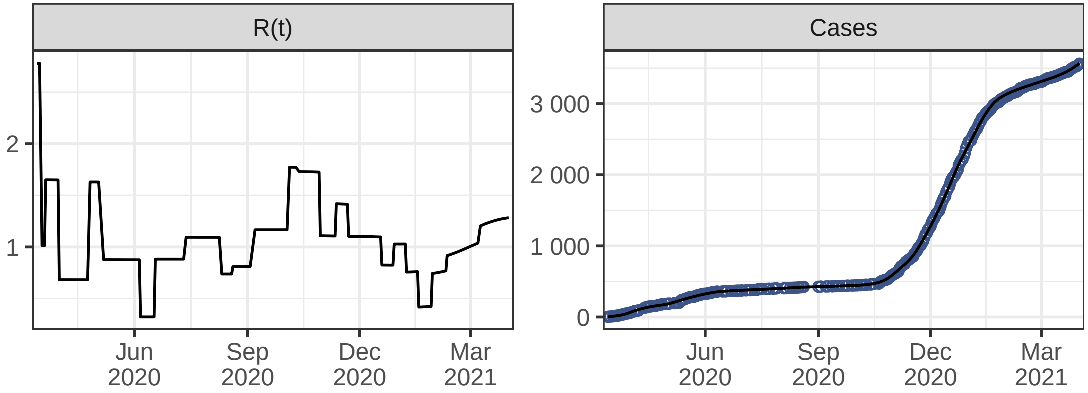

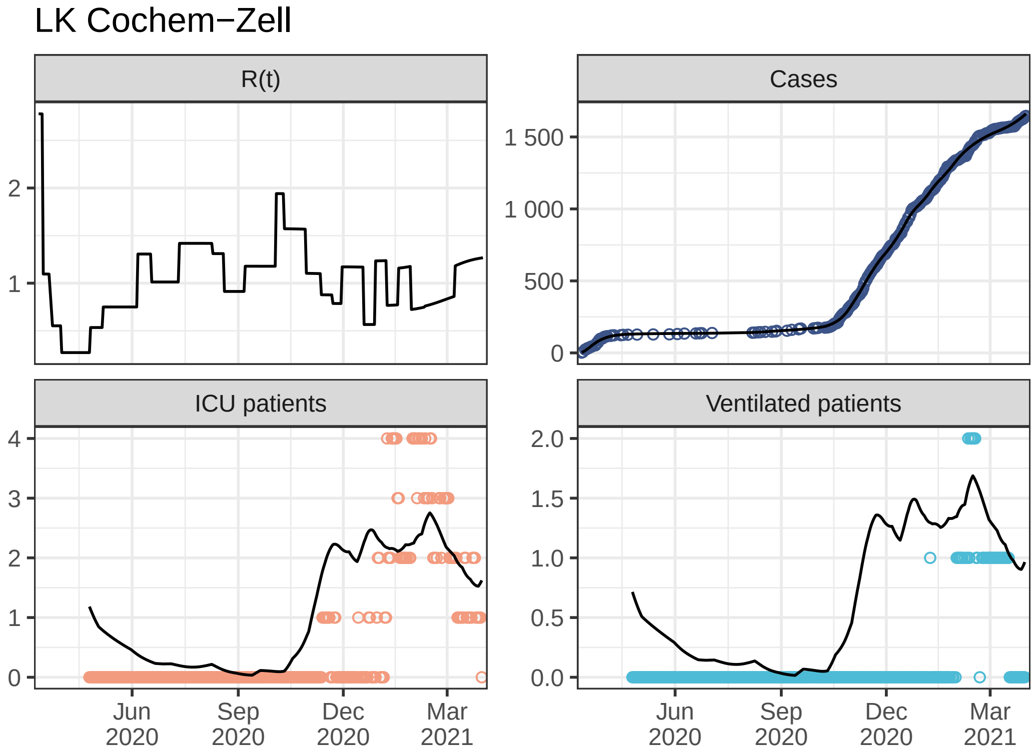
LK Cochem−Zell
R(t)
Cases
ICU patients
Ventilated patients
Jun 2020
Sep 2020
Dec 2020
Mar 2021

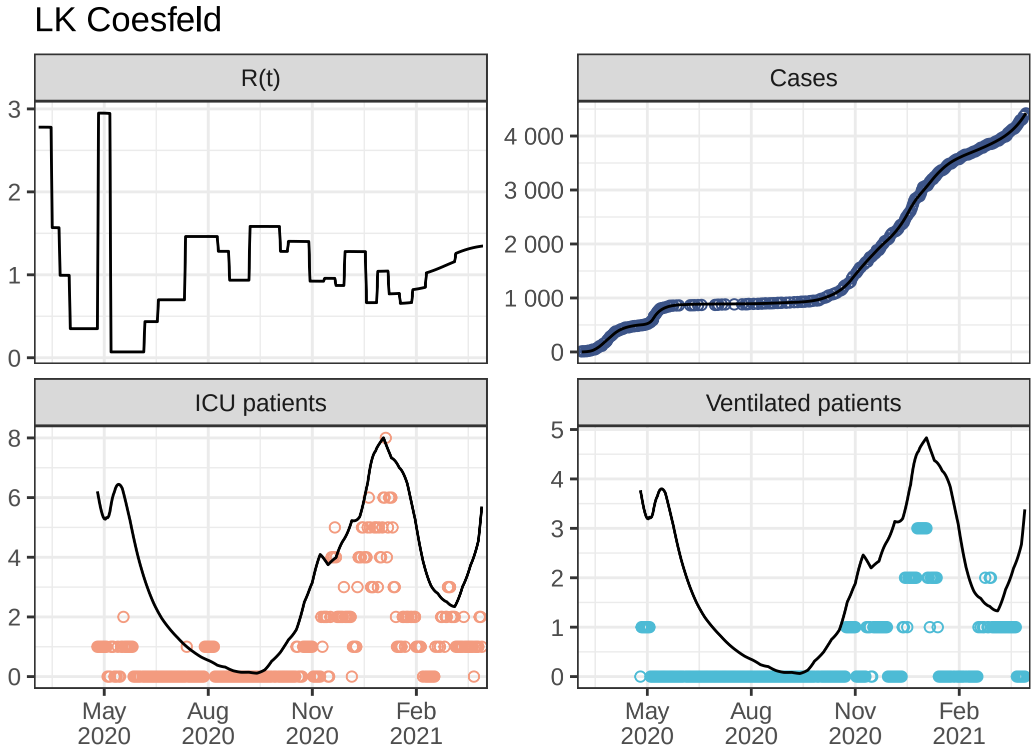
LK Coesfeld
R(t)
Cases
ICU patients
Ventilated patients
May 2020
Aug 2020
Nov 2020
Feb 2021

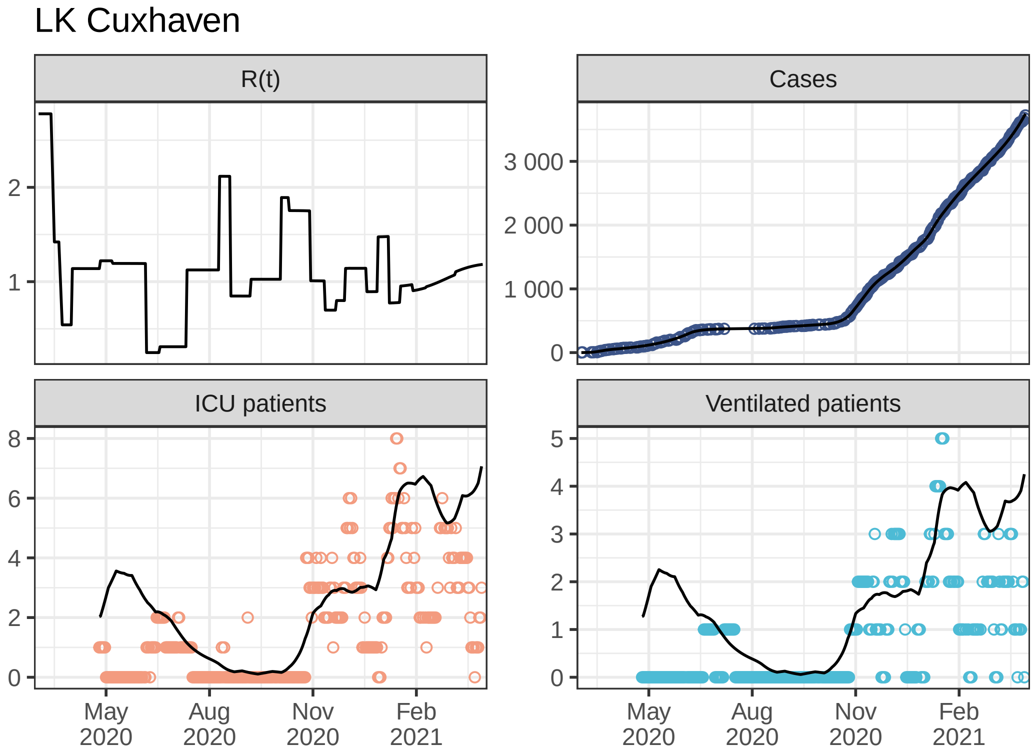
LK Cuxhaven
R(t)
Cases
ICU patients
Ventilated patients
2
1
3 000
2 000
1 000
0
8
6
4
2
0
5
4
3
2
1
0
May 2020
Aug 2020
Nov 2020
Feb 2021

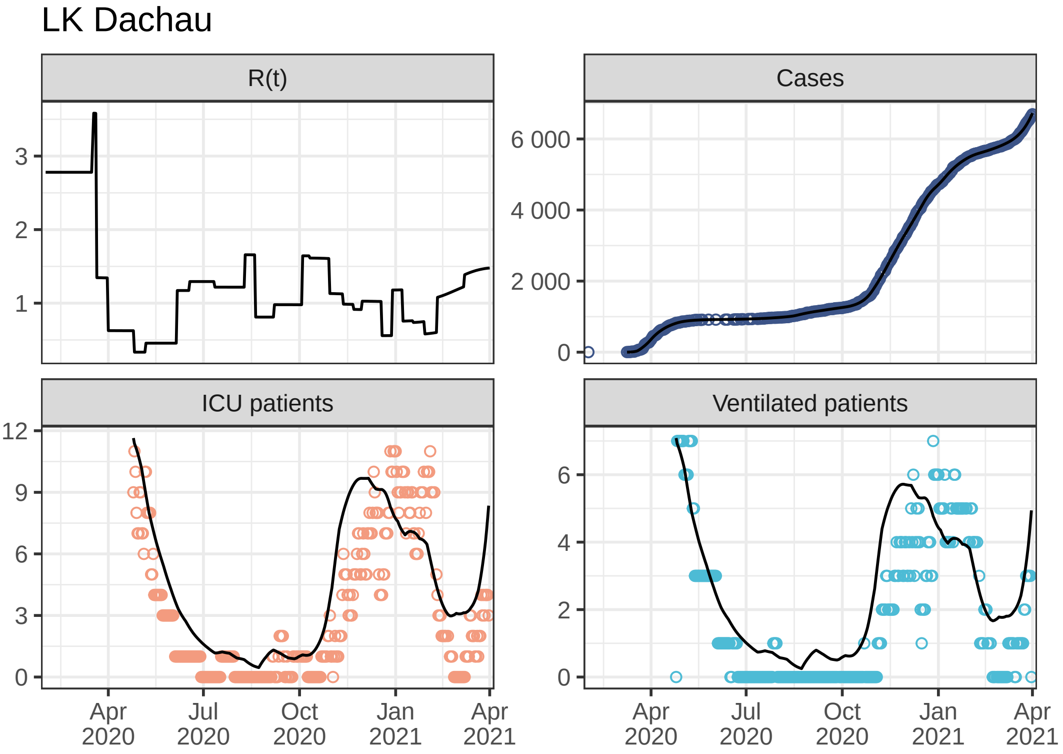
LK Dachau
R(t)
Cases
ICU patients
Ventilated patients
3
2
1
6 000
4 000
2 000
0
12
9
6
3
0
6
4
2
0
Apr 2020
Jul 2020
Oct 2020
Jan 2021
Apr 2021

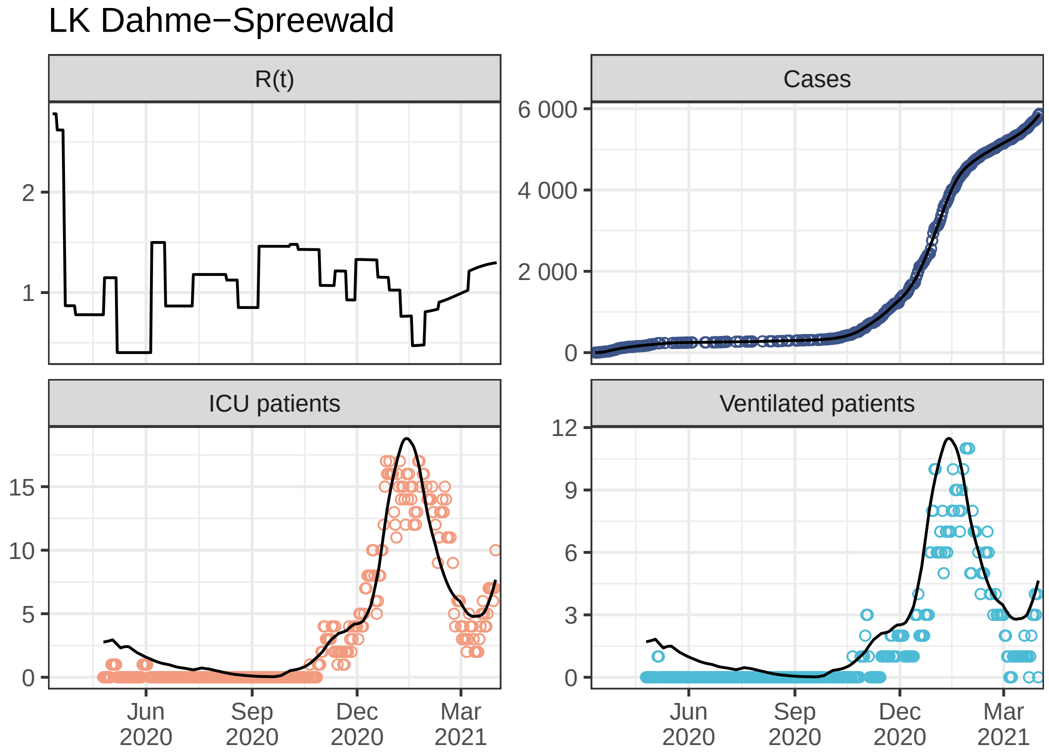
LK Dahme−Spreewald
R(t)
Cases
ICU patients
Ventilated patients
Jun 2020
Sep 2020
Dec 2020
Mar 2021

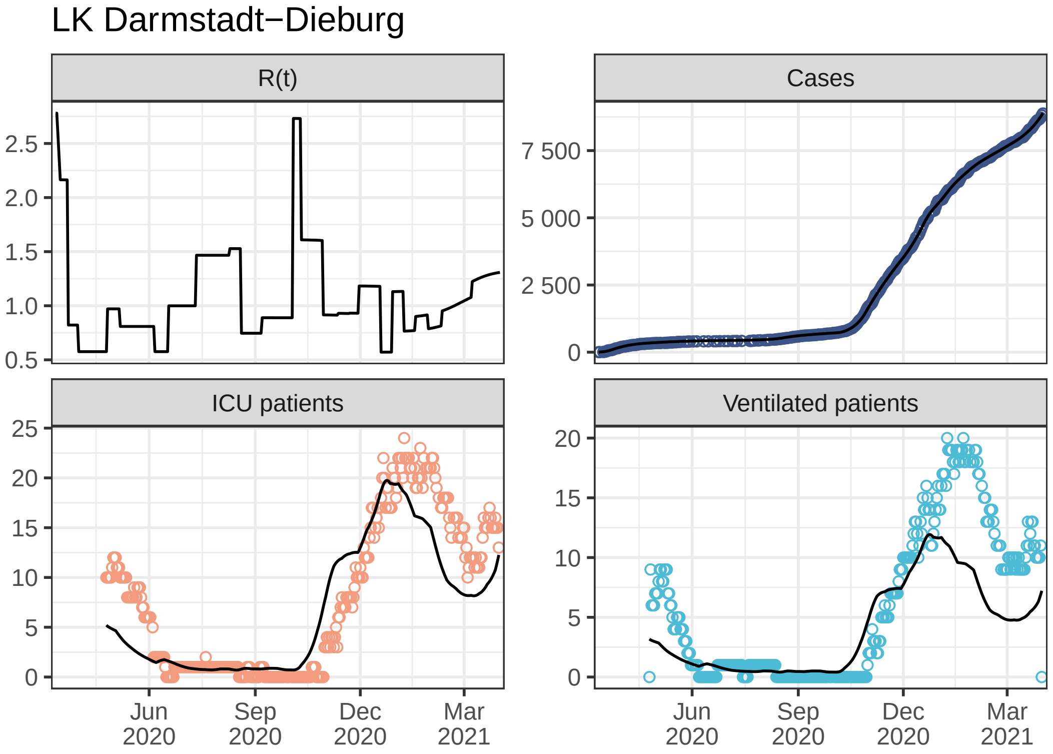
LK Darmstadt−Dieburg
R(t)
Cases
ICU patients
Ventilated patients
Jun 2020
Sep 2020
Dec 2020
Mar 2021

LK Deggendorf

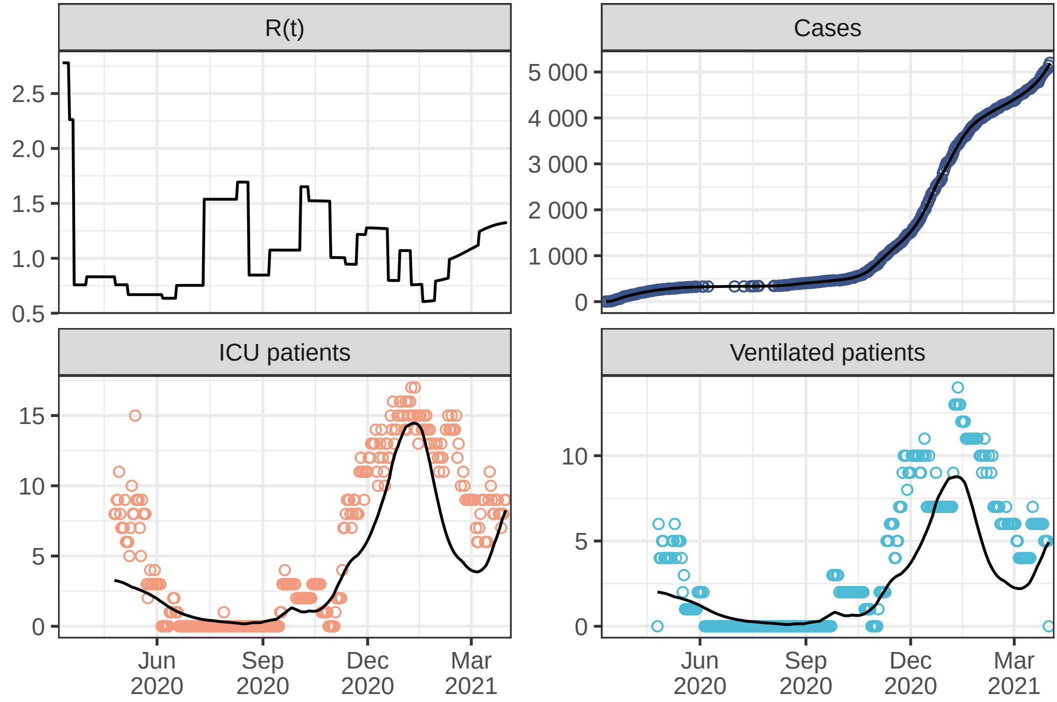

LK Diepholz

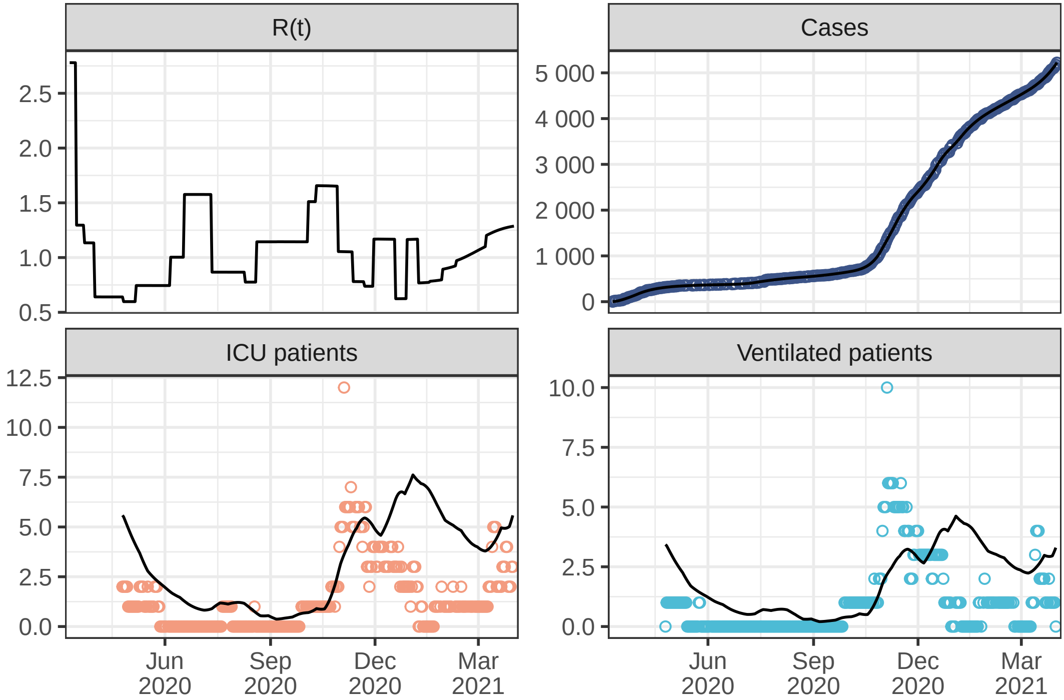

LK Dillingen a.d.Donau

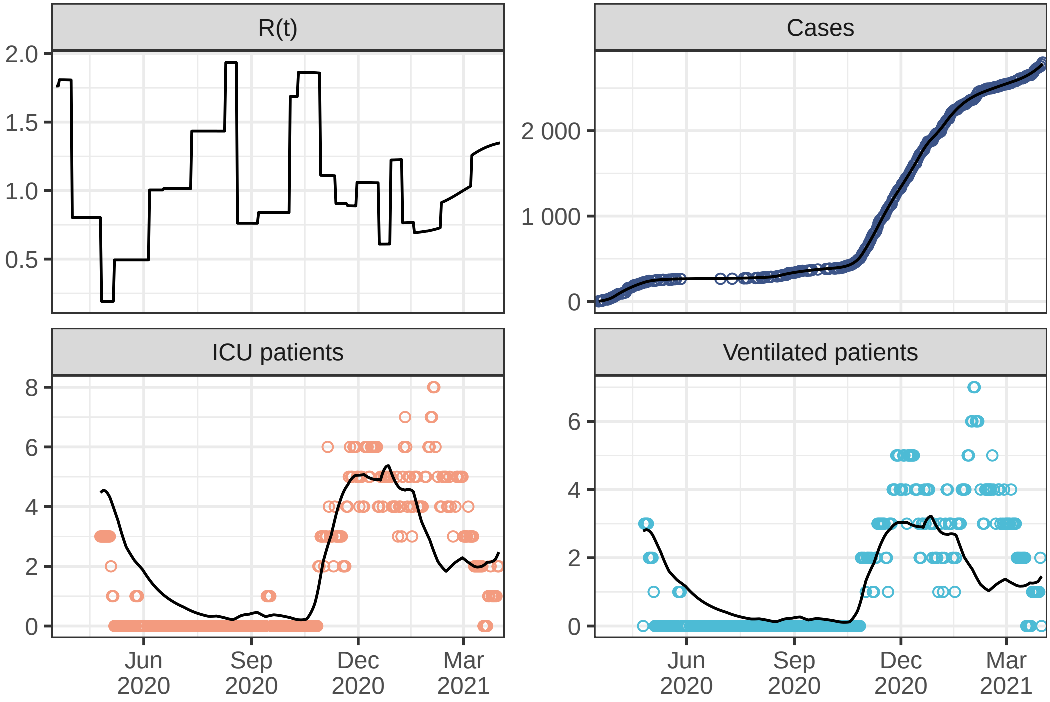

LK Dingolfing−Landau

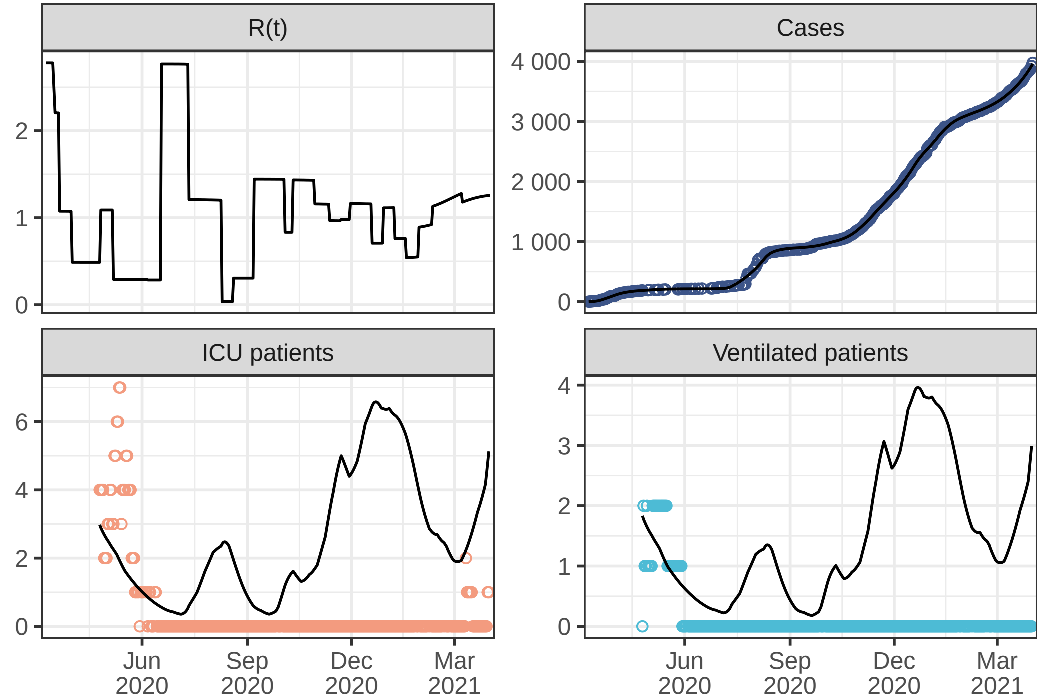

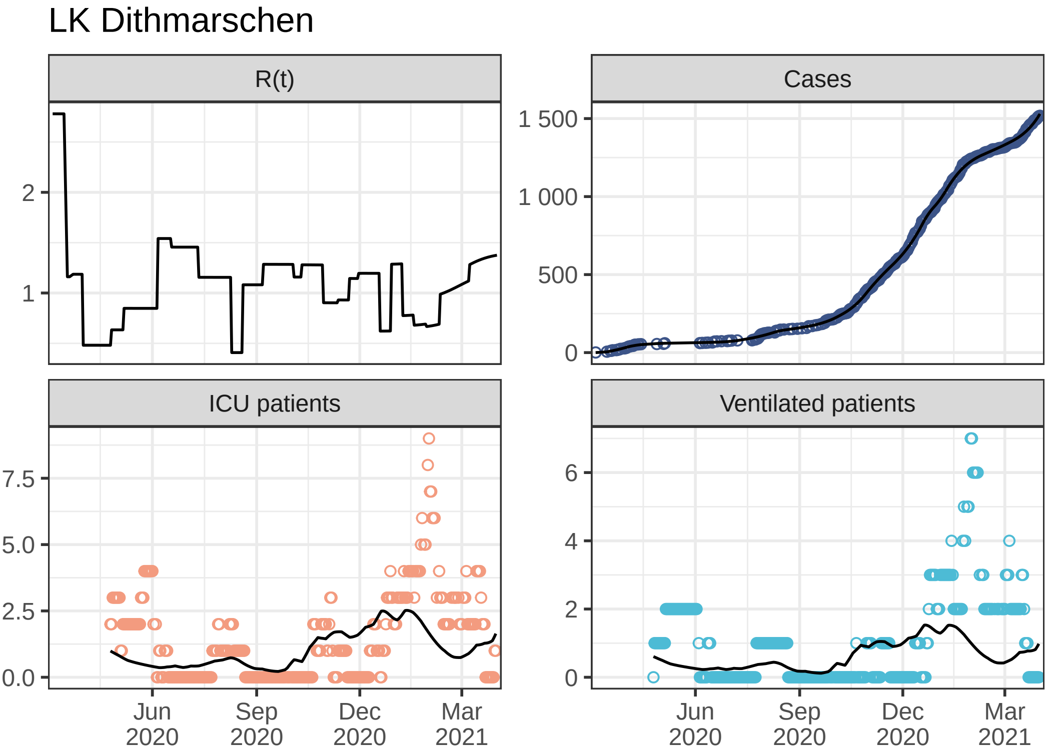
LK Dithmarschen
R(t)
Cases
ICU patients
Ventilated patients
2
1
1 500
1 000
500
0
7.5
5.0
2.5
0.0
6
4
2
0
Jun 2020
Sep 2020
Dec 2020
Mar 2021
Jun 2020
Sep 2020
Dec 2020
Mar 2021

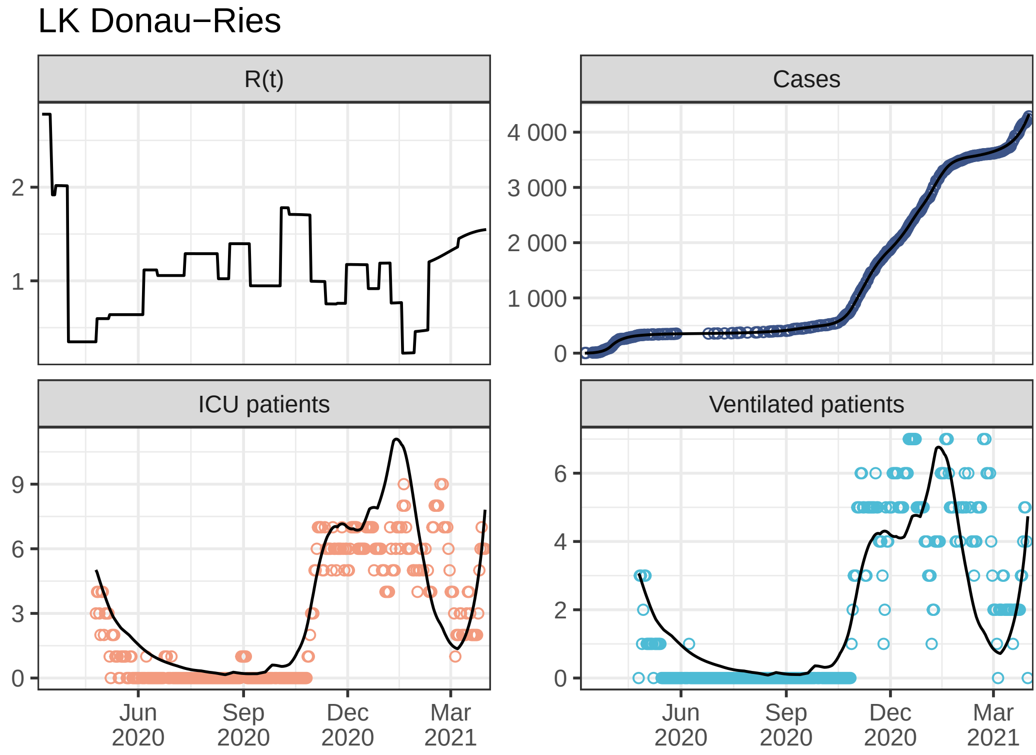
LK Donau−Ries
R(t)
Cases
ICU patients
Ventilated patients
2
1
4 000
3 000
2 000
1 000
0
9
6
3
0
6
4
2
0
Jun 2020
Sep 2020
Dec 2020
Mar 2021
Jun 2020
Sep 2020
Dec 2020
Mar 2021

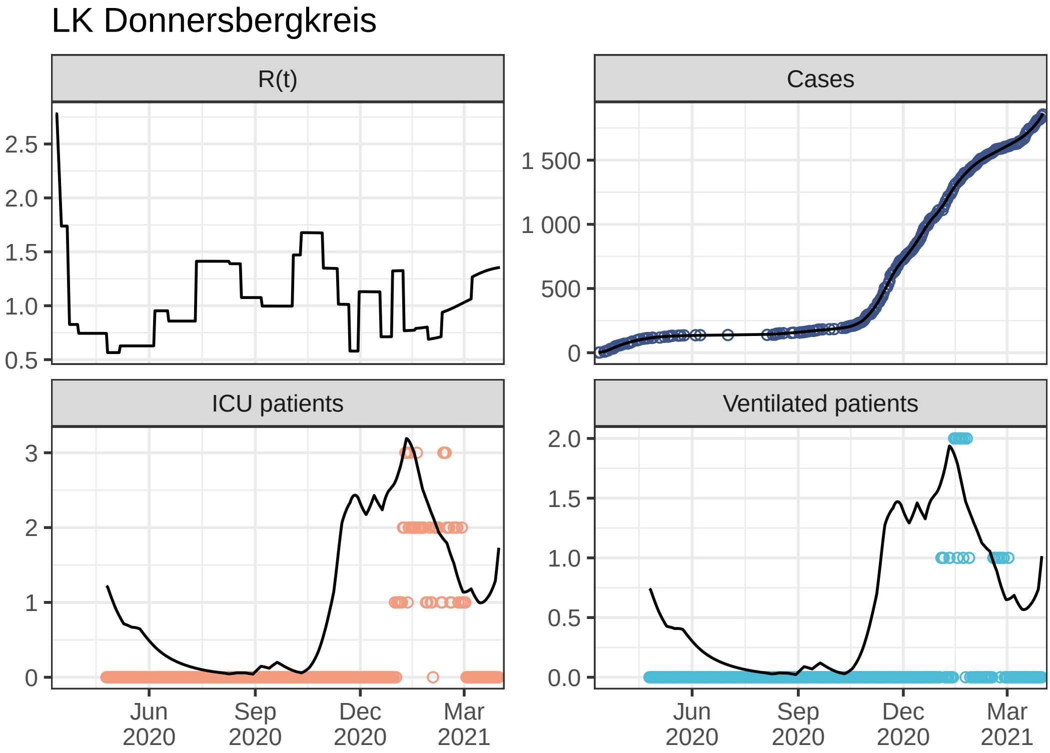
LK Donnersbergkreis
R(t)
Cases
ICU patients
Ventilated patients
2.5
2.0
1.5
1.0
0.5
1 500
1 000
500
0
3
2
1
0
2.0
1.5
1.0
0.5
0.0
Jun 2020
Sep 2020
Dec 2020
Mar 2021

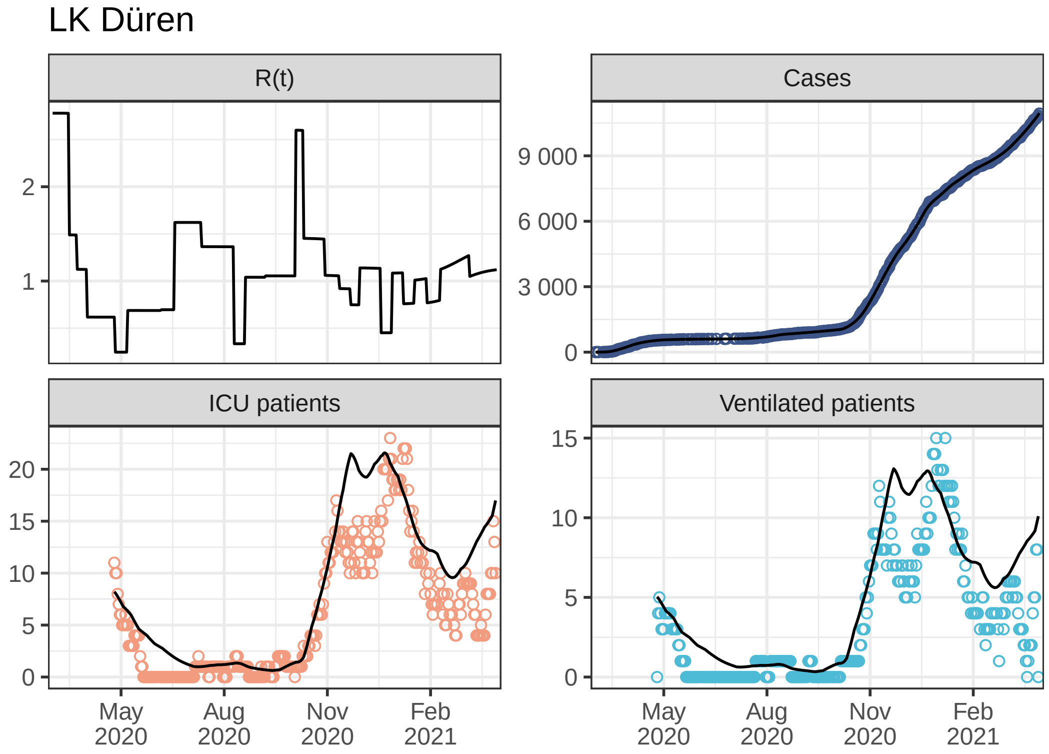
LK Düren
R(t)
Cases
ICU patients
Ventilated patients
2
1
9 000
6 000
3 000
0
20
15
10
5
0
15
10
5
0
May 2020
Aug 2020
Nov 2020
Feb 2021

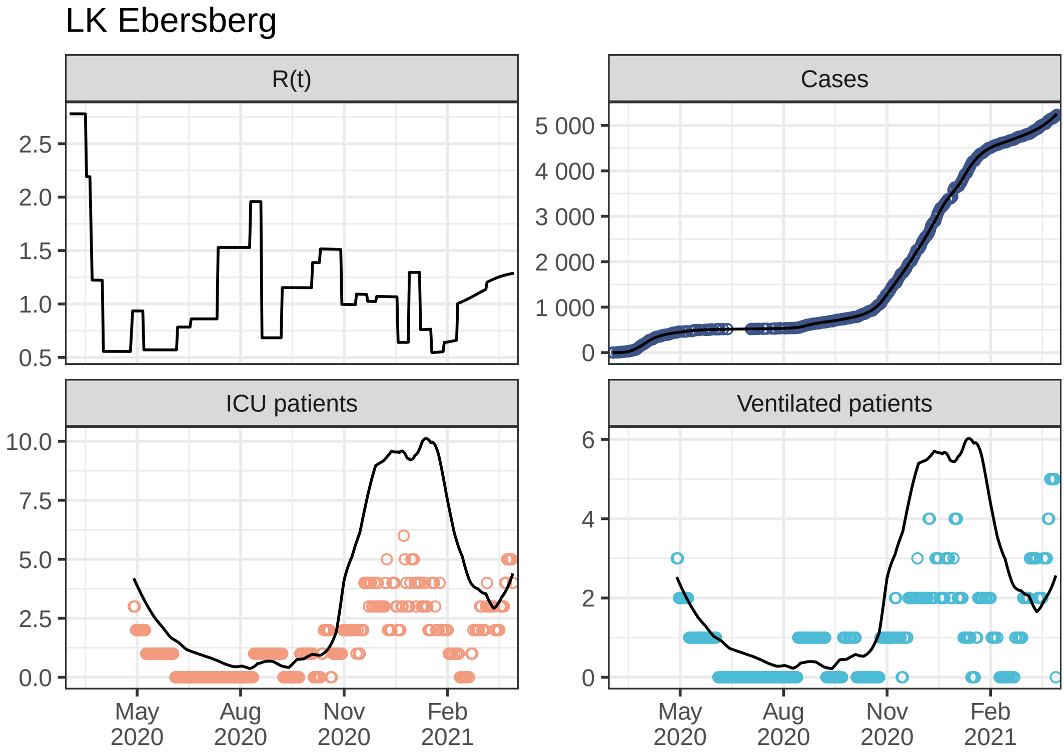
LK Ebersberg
R(t)
Cases
ICU patients
Ventilated patients
2.5
2.0
1.5
1.0
0.5
5 000
4 000
3 000
2 000
1 000
0
10.0
7.5
5.0
2.5
0.0
6
4
2
0
May 2020
Aug 2020
Nov 2020
Feb 2021

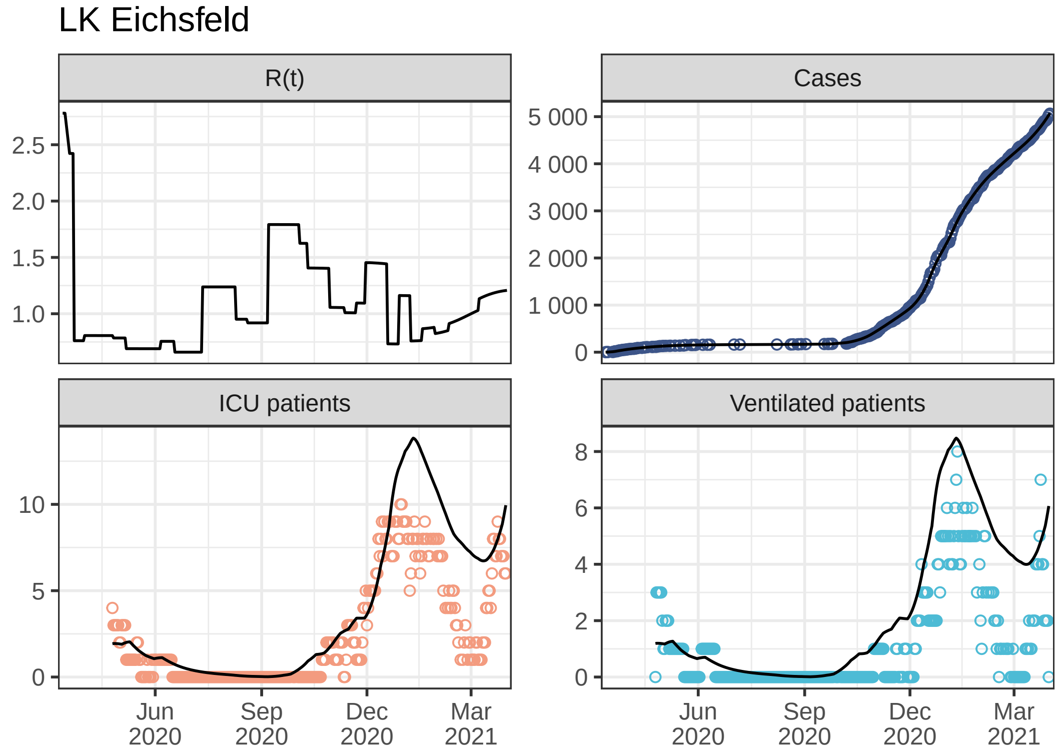
LK Eichsfeld
R(t)
Cases
ICU patients
Ventilated patients
2.5
2.0
1.5
1.0
5 000
4 000
3 000
2 000
1 000
0
10
5
0
8
6
4
2
0
Jun 2020
Sep 2020
Dec 2020
Mar 2021

LK Eichstätt

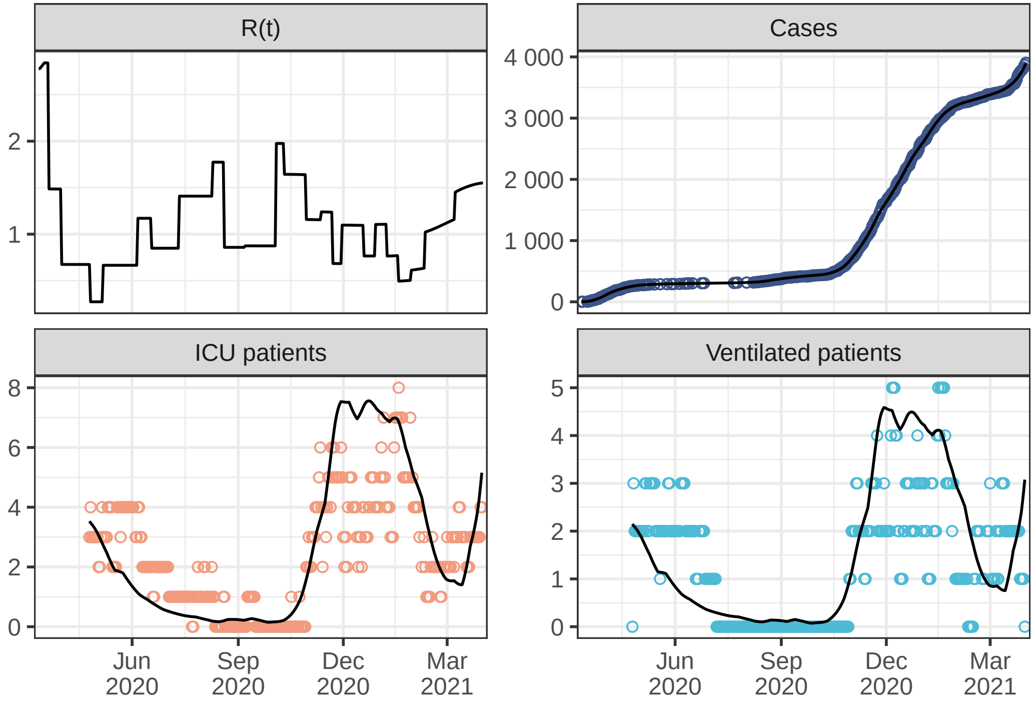

LK Elbe−Elster

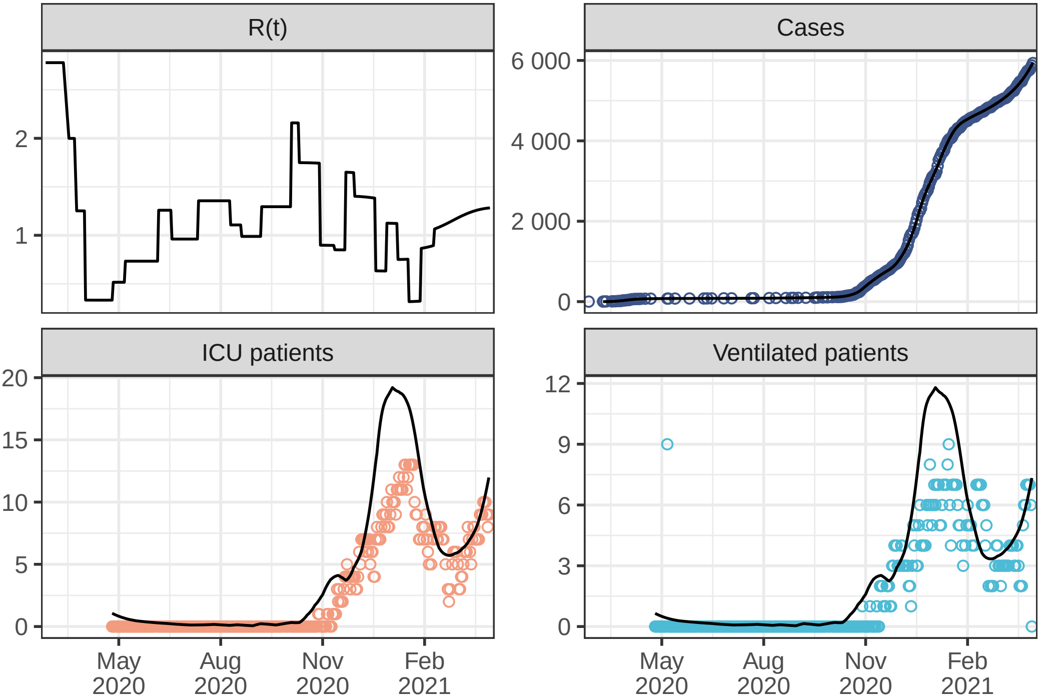

LK Emmendingen

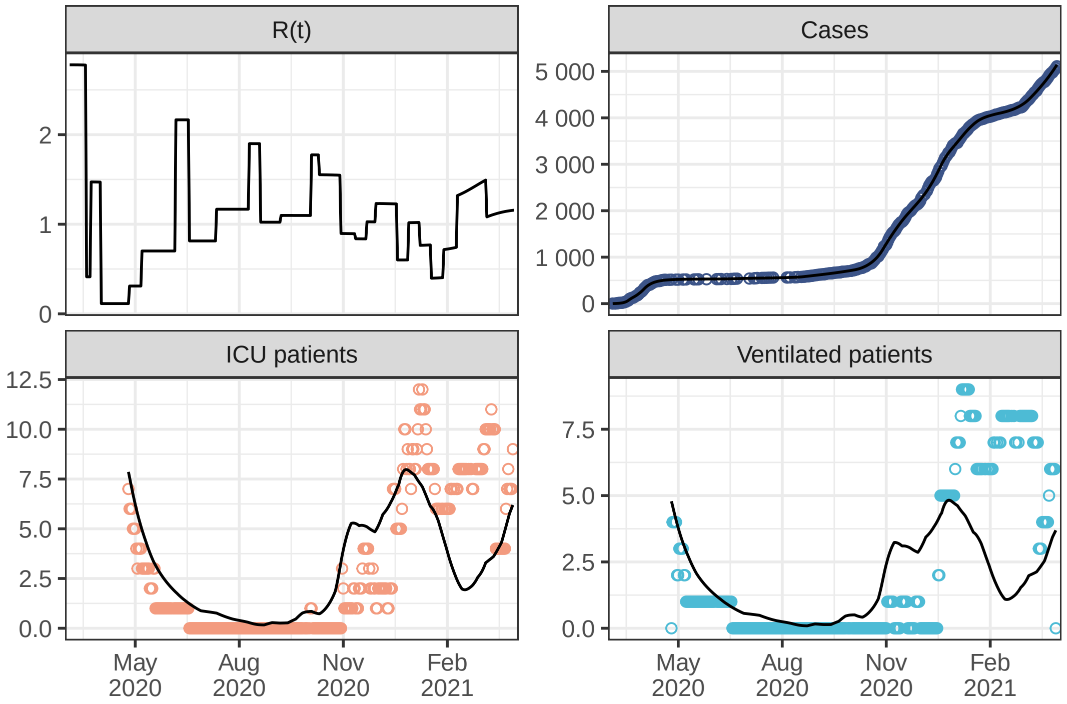

LK Emsland

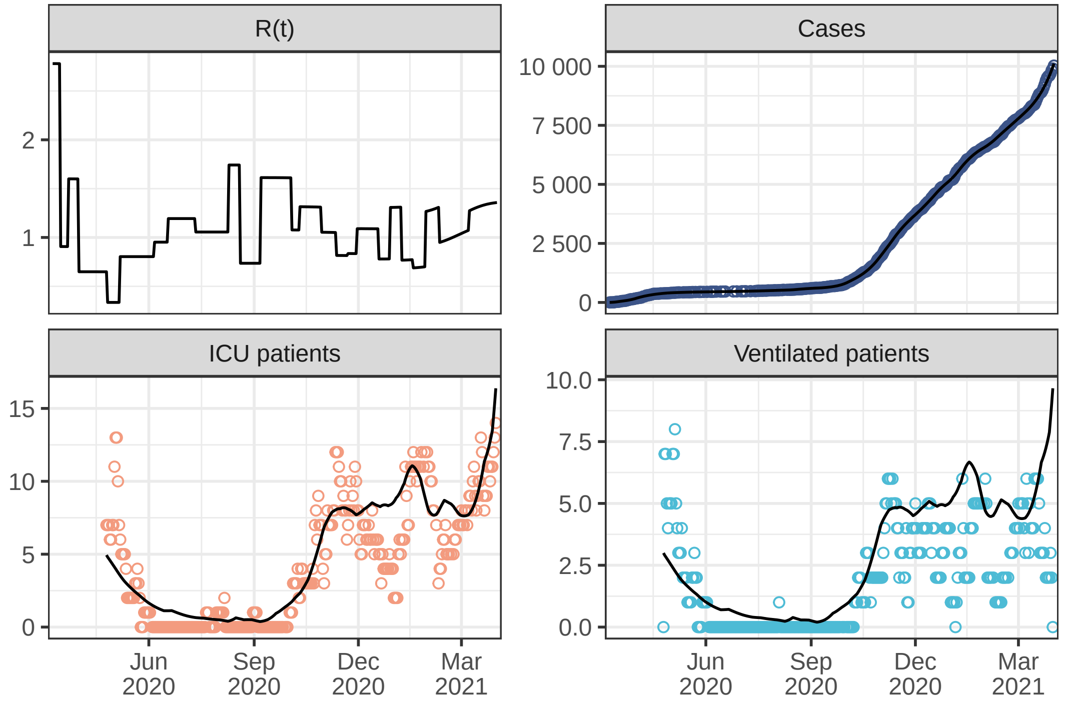

LK Ennepe−Ruhr−Kreis

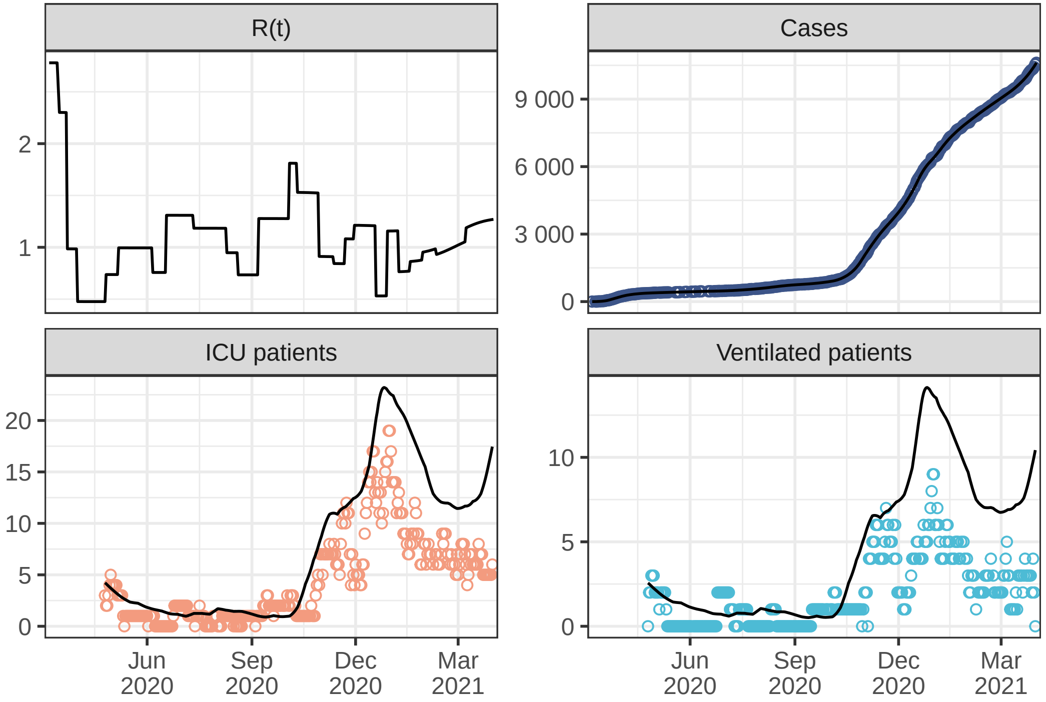

LK Enzkreis

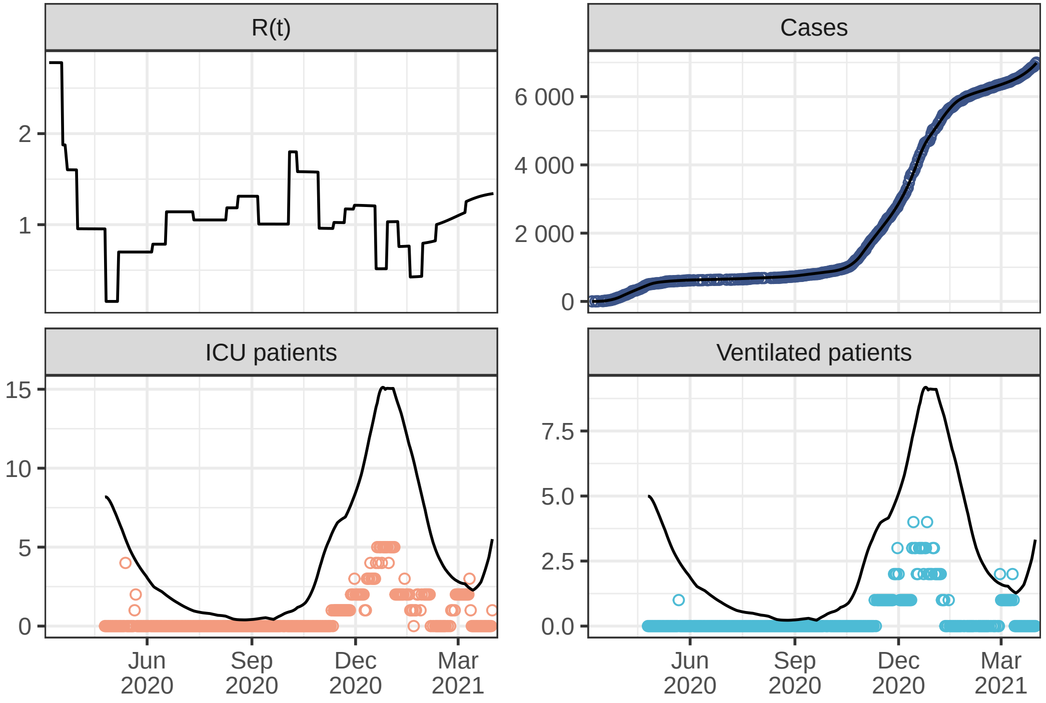

LK Erding

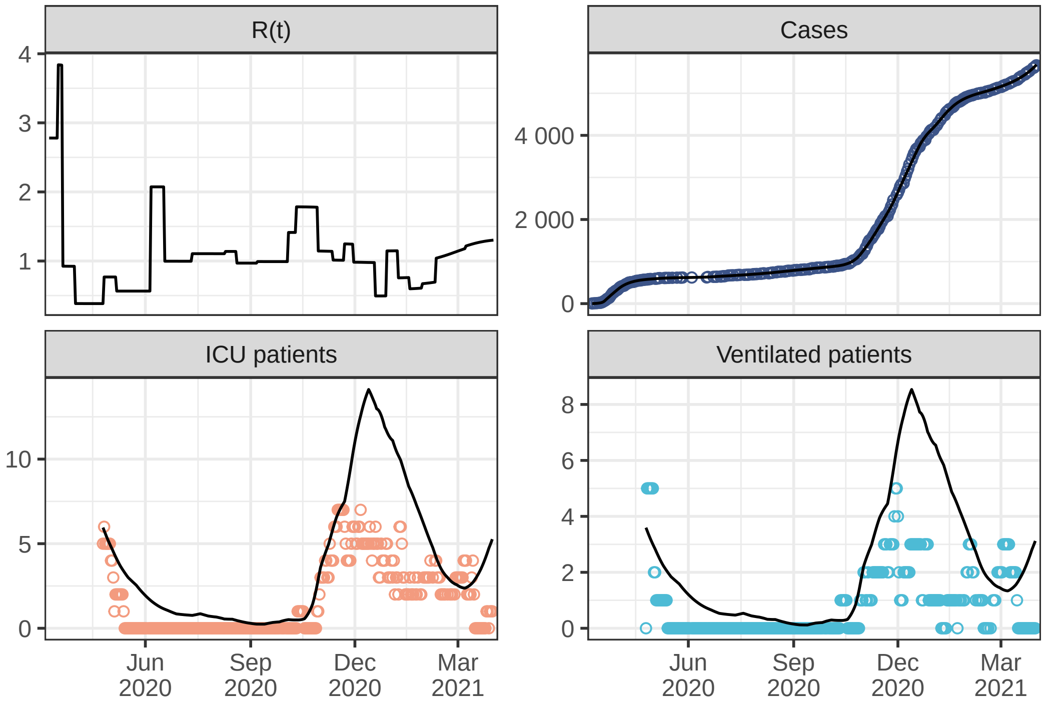

LK Erlangen−Höchstadt

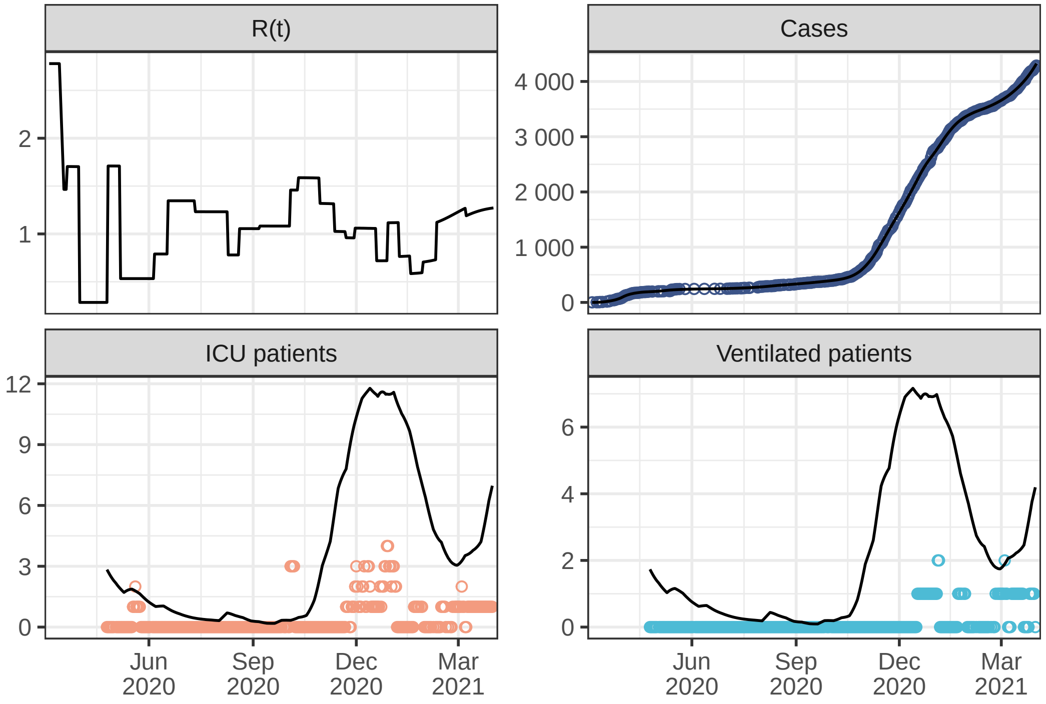

LK Erzgebirgskreis

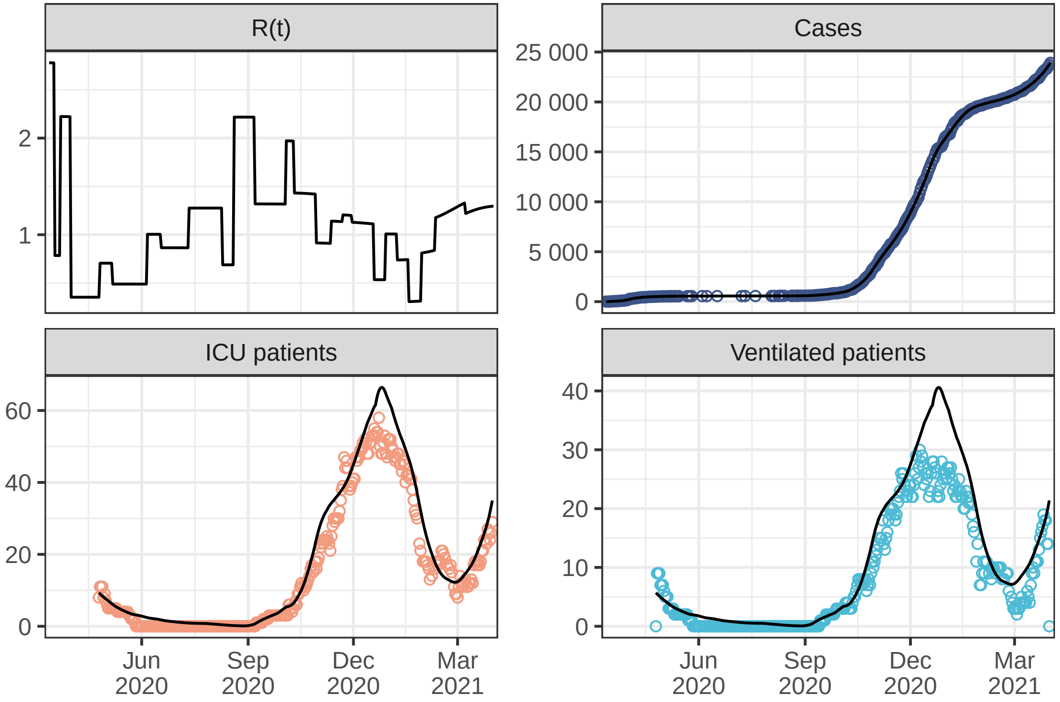

LK Esslingen

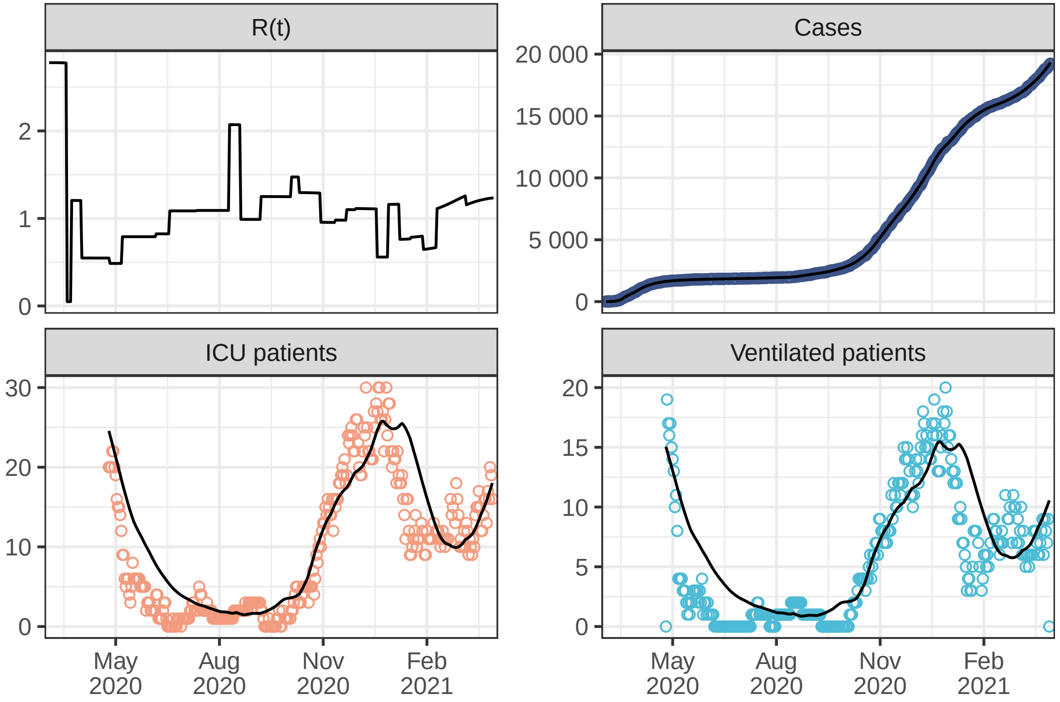

LK Euskirchen

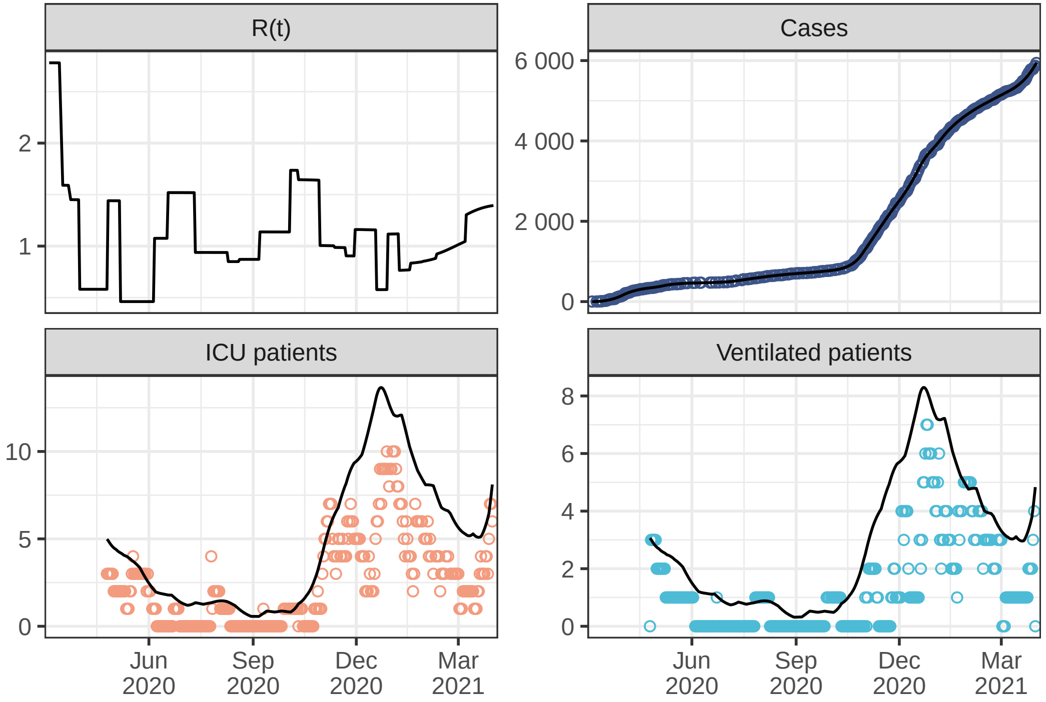

LK Forchheim

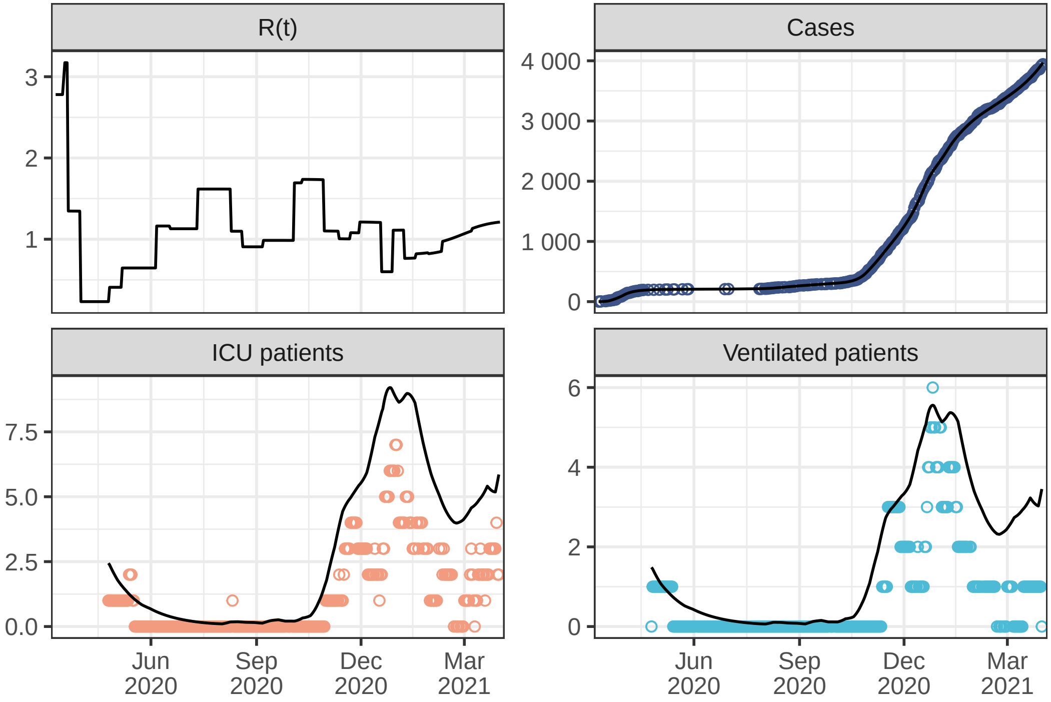

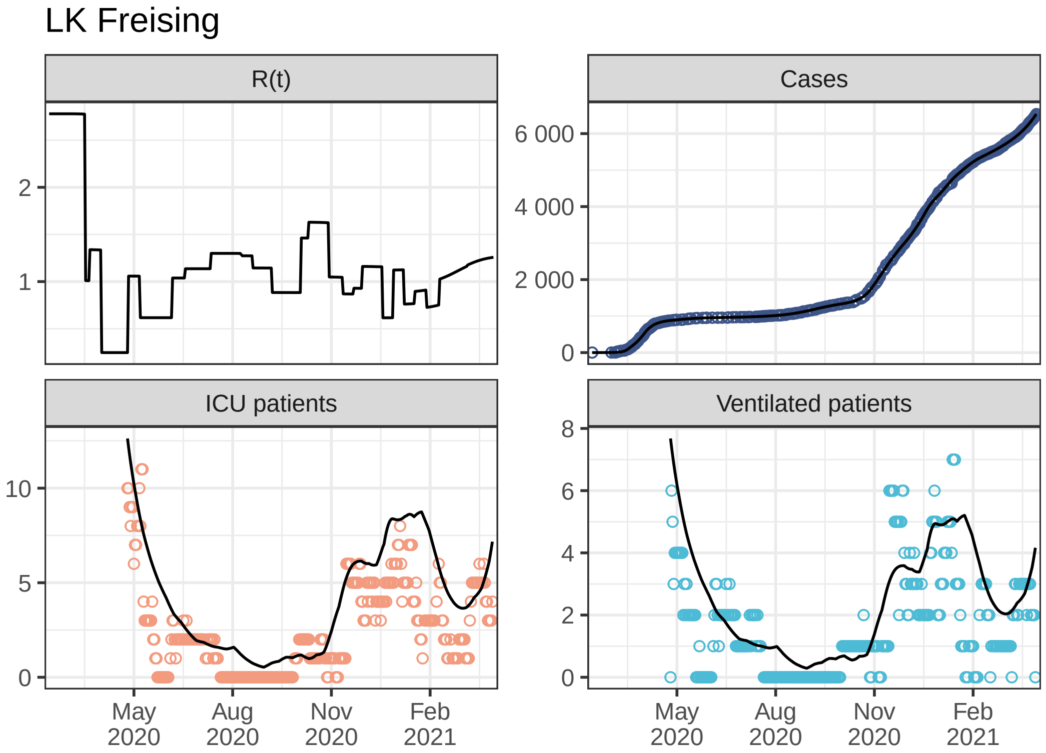
LK Freising
R(t)
Cases
ICU patients
Ventilated patients
2
1
6 000
4 000
2 000
0
10
5
0
8
6
4
2
0
May 2020
Aug 2020
Nov 2020
Feb 2021

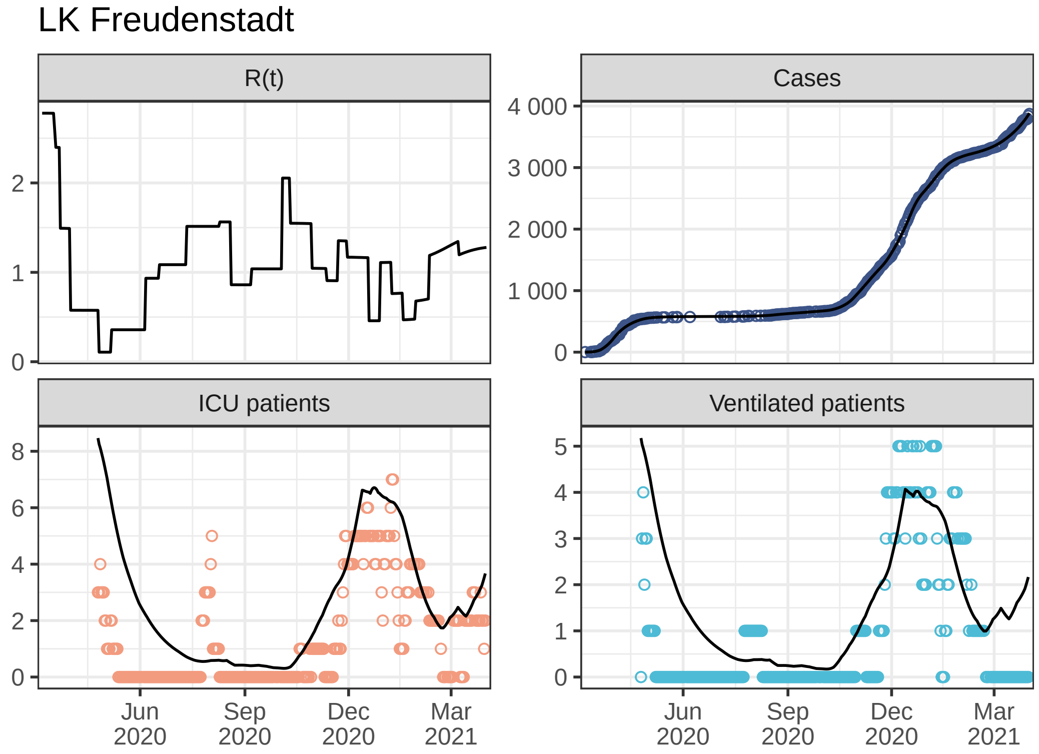
LK Freudenstadt
R(t)
Cases
ICU patients
Ventilated patients
2
1
0
4 000
3 000
2 000
1 000
0
8
6
4
2
0
5
4
3
2
1
0
Jun 2020
Sep 2020
Dec 2020
Mar 2021

LK Freyung−Grafenau

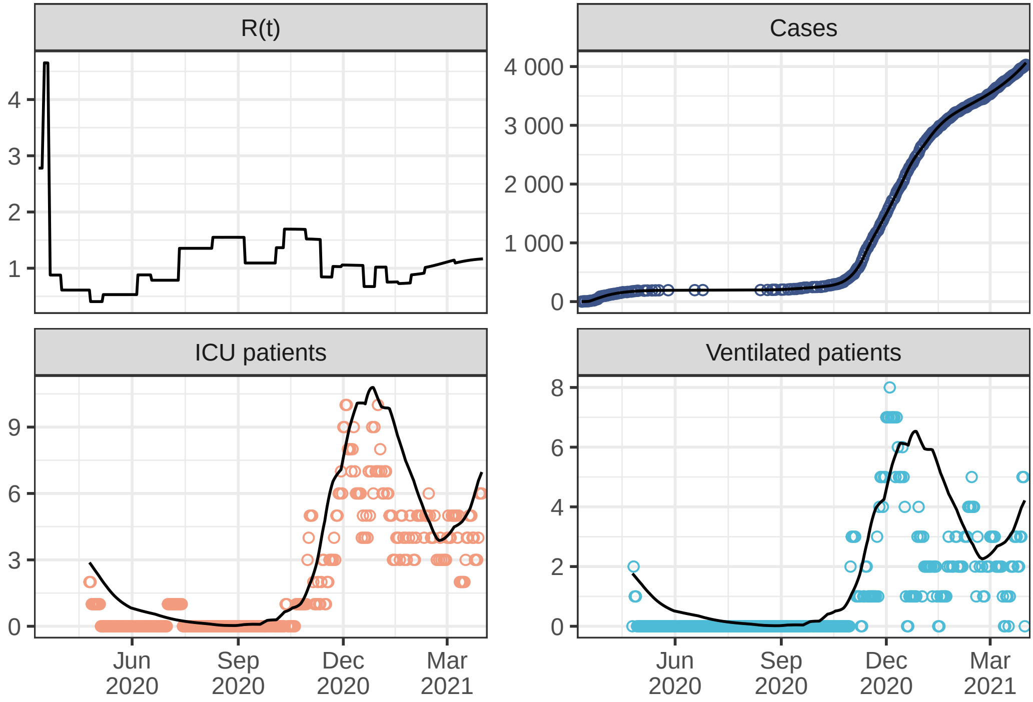

LK Friesland

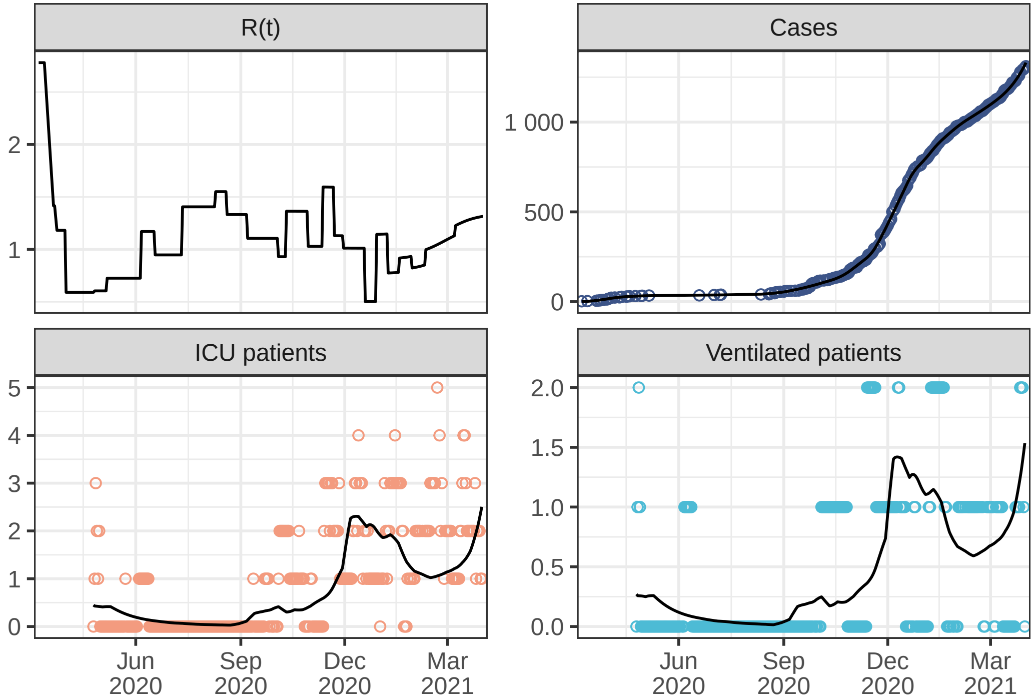

LK Fulda

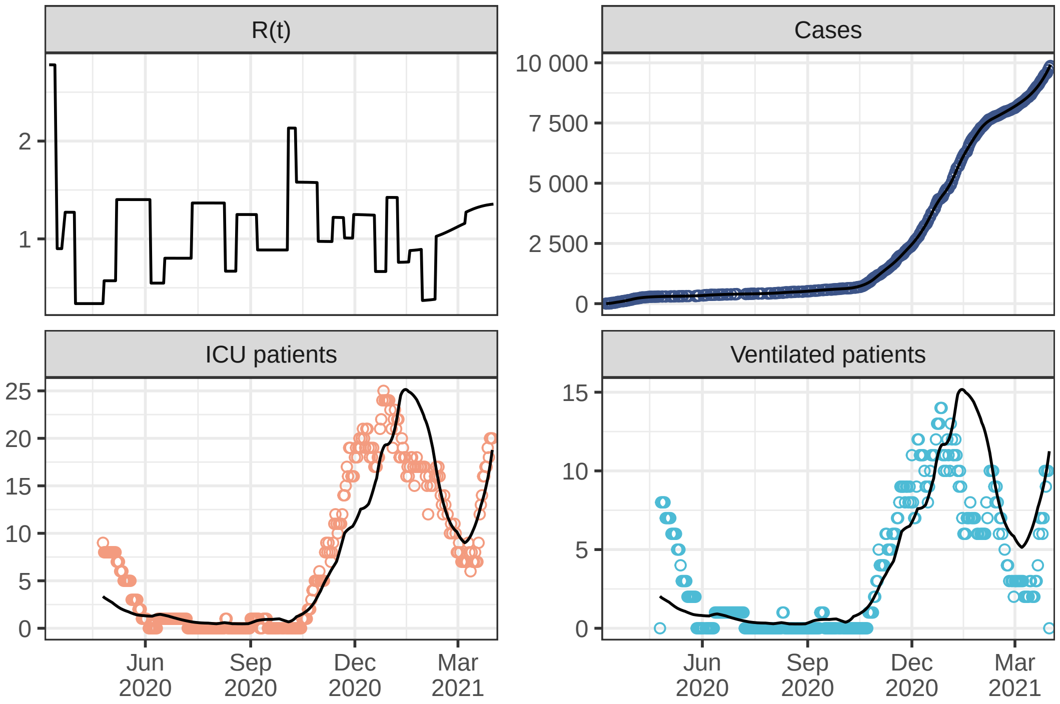

LK Fürstenfeldbruck

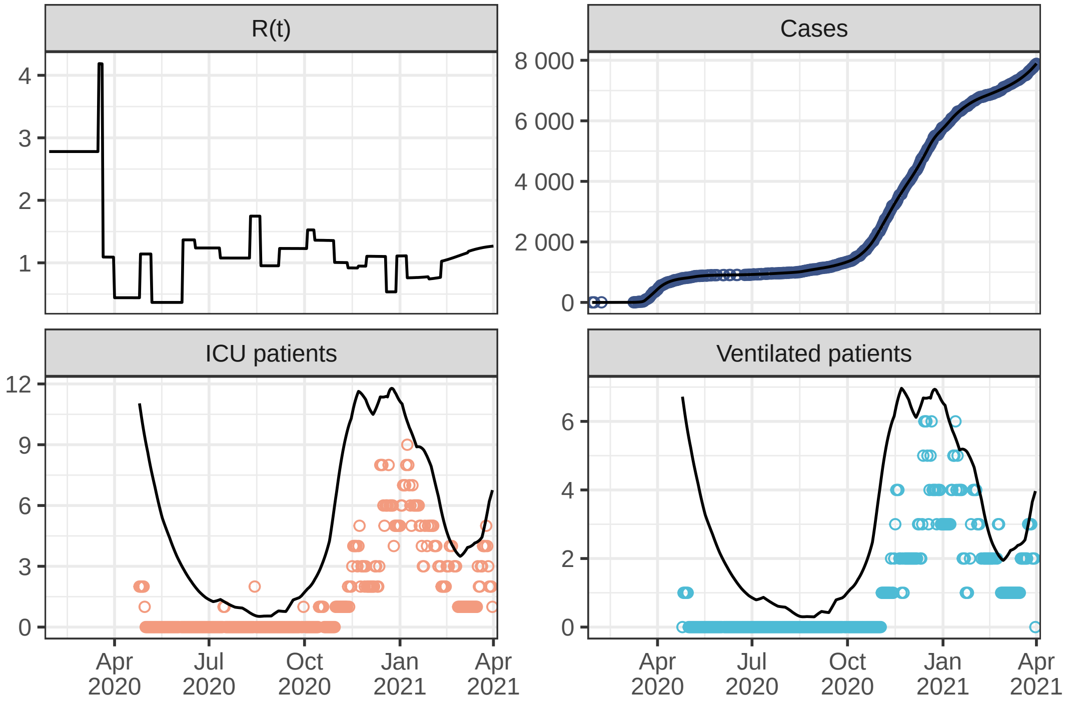

LK Fürth

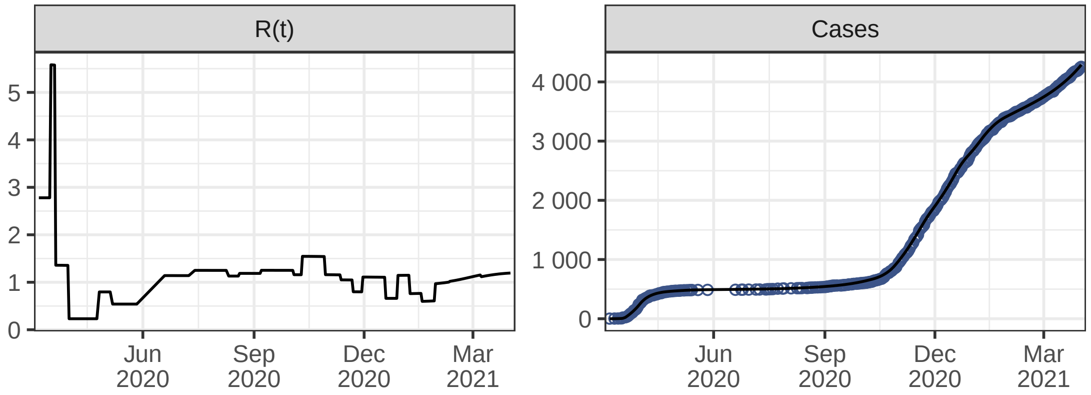

LK Garmisch−Partenkirchen

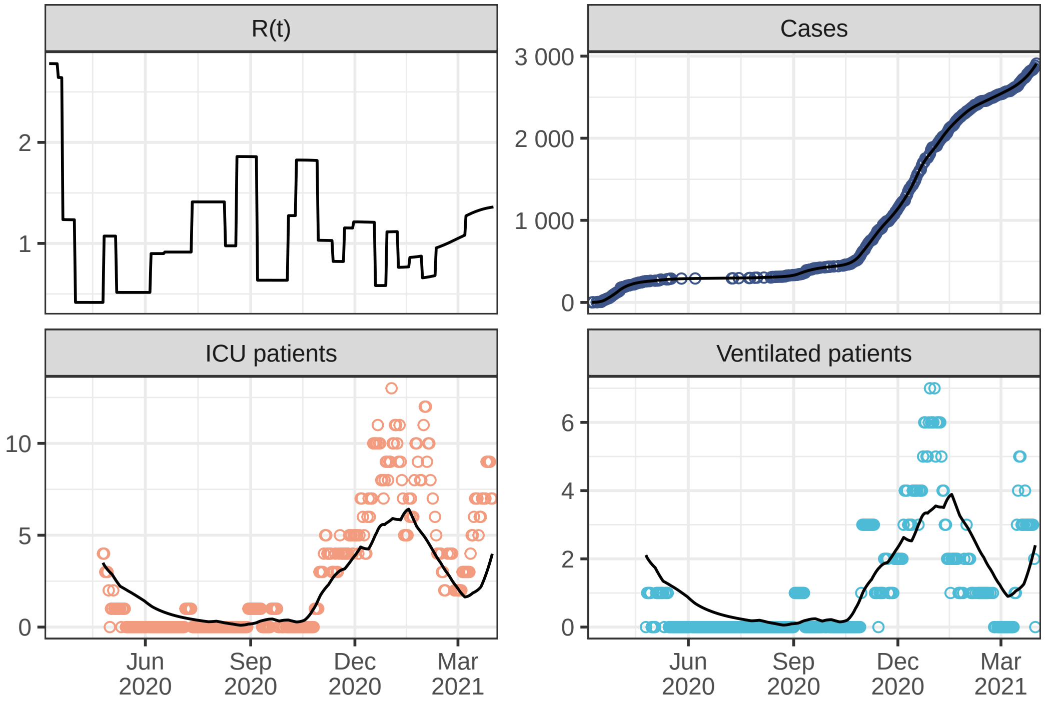

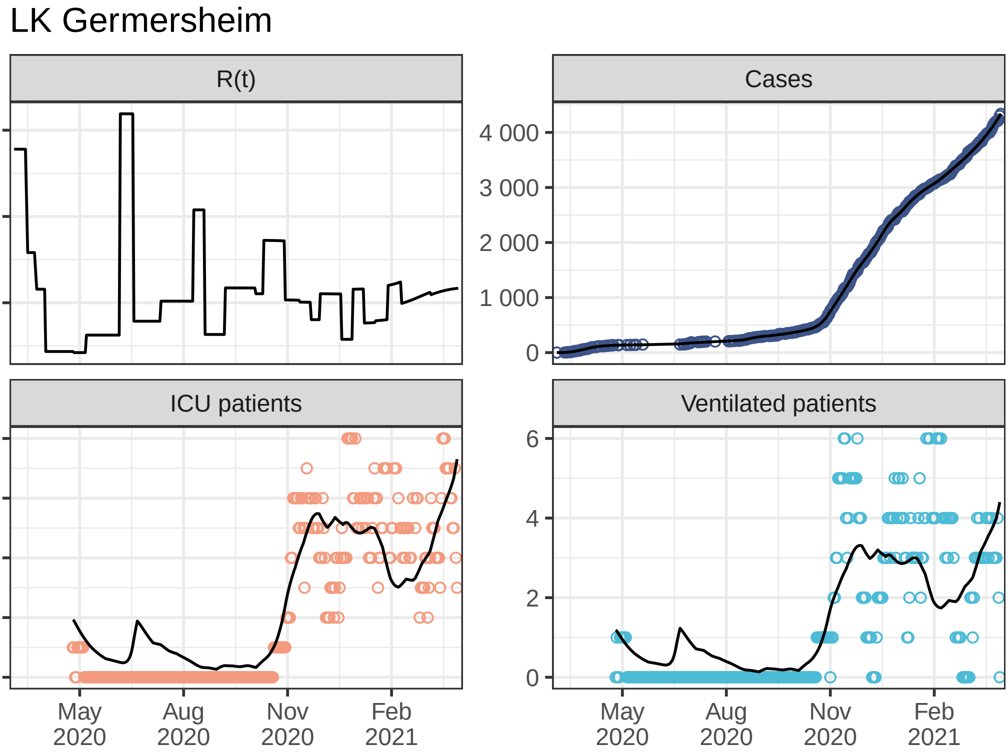
LK Germersheim
R(t)
Cases
ICU patients
Ventilated patients
3
2
1
4 000
3 000
2 000
1 000
0
8
6
4
2
0
6
4
2
0
May 2020
Aug 2020
Nov 2020
Feb 2021

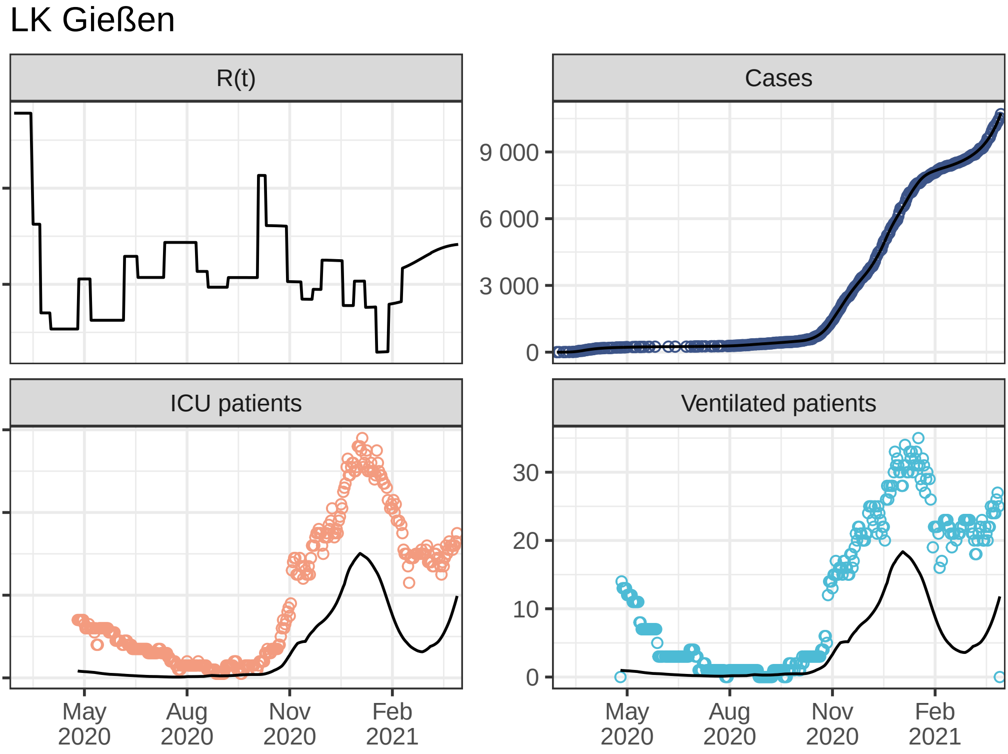
LK Gießen
R(t)
Cases
ICU patients
Ventilated patients
2
1
9 000
6 000
3 000
0
60
40
20
0
30
20
10
0
May 2020
Aug 2020
Nov 2020
Feb 2021

LK Gifhorn

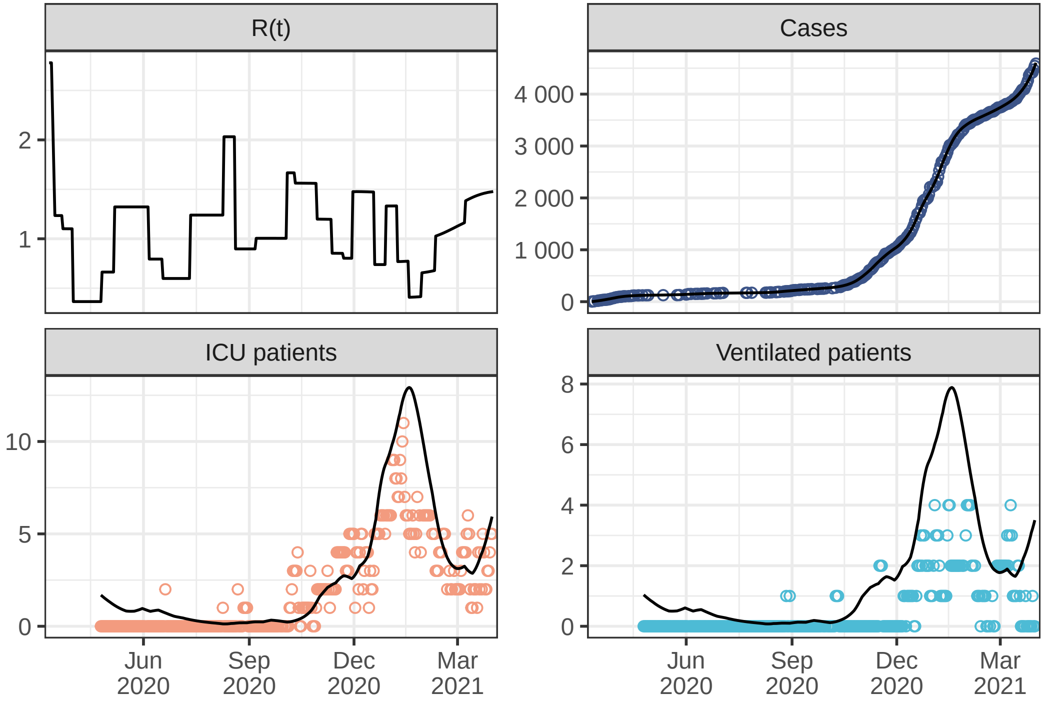

LK Göppingen

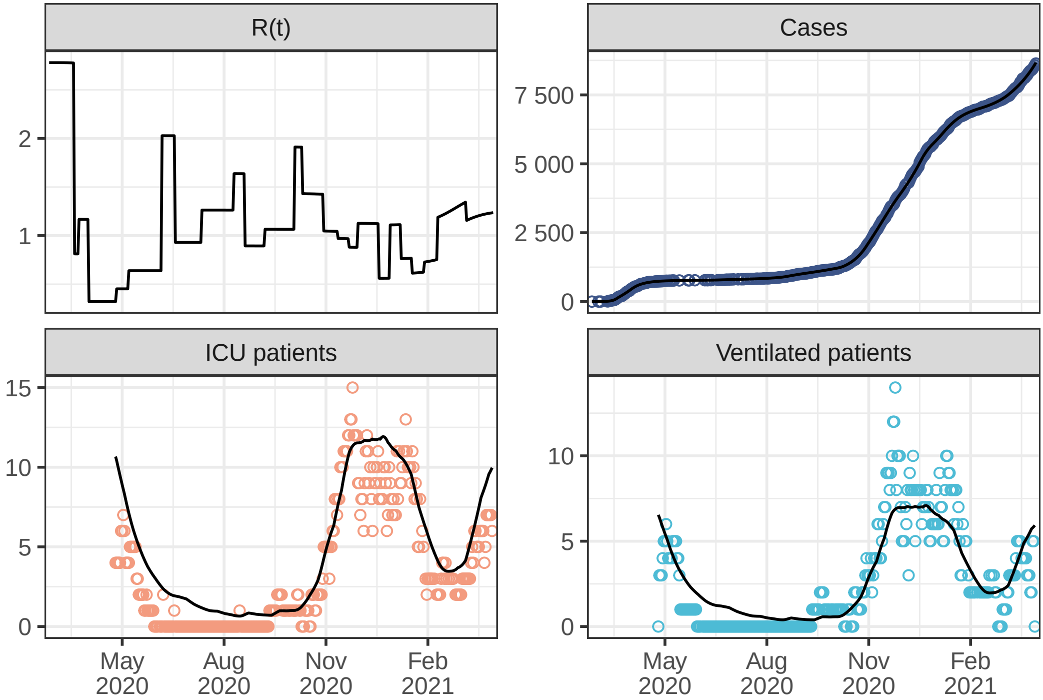

LK Görlitz

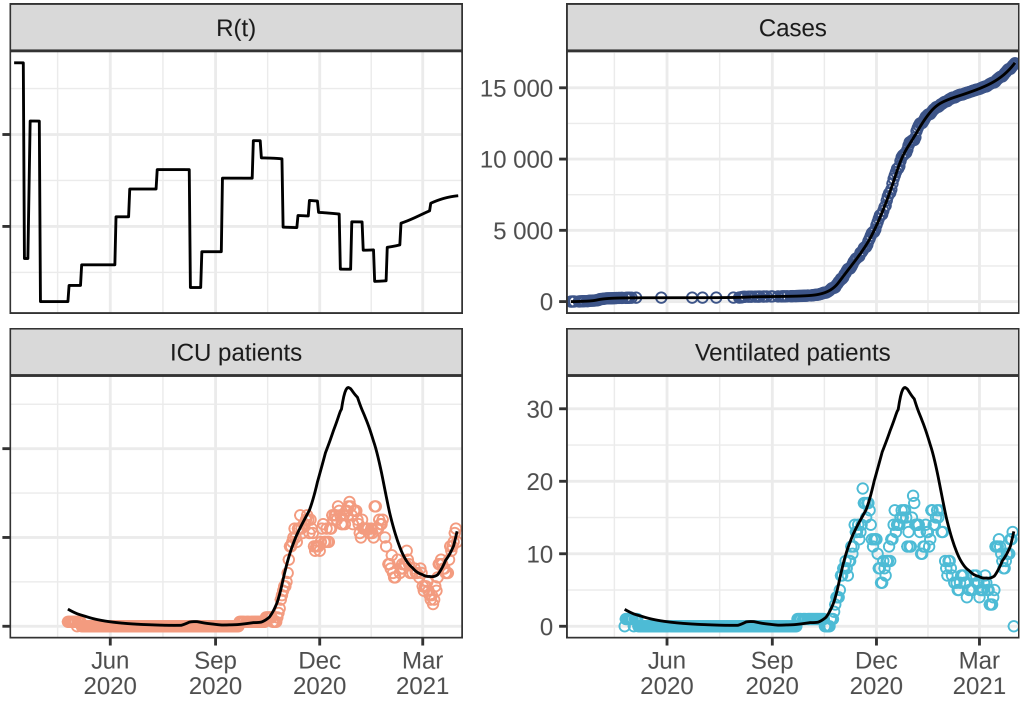

LK Goslar

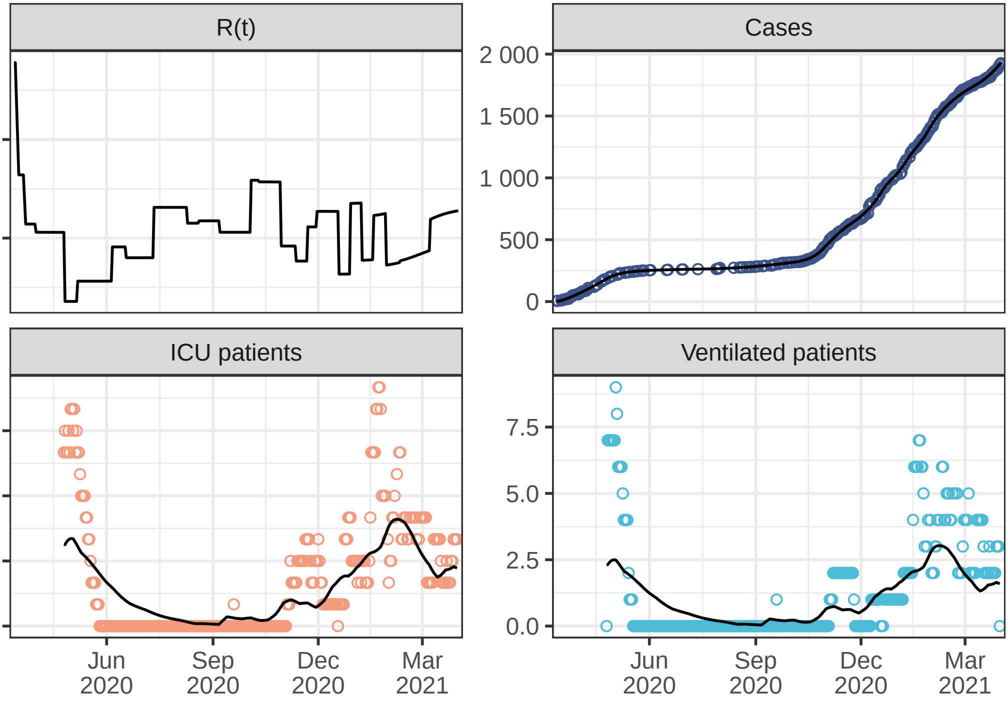

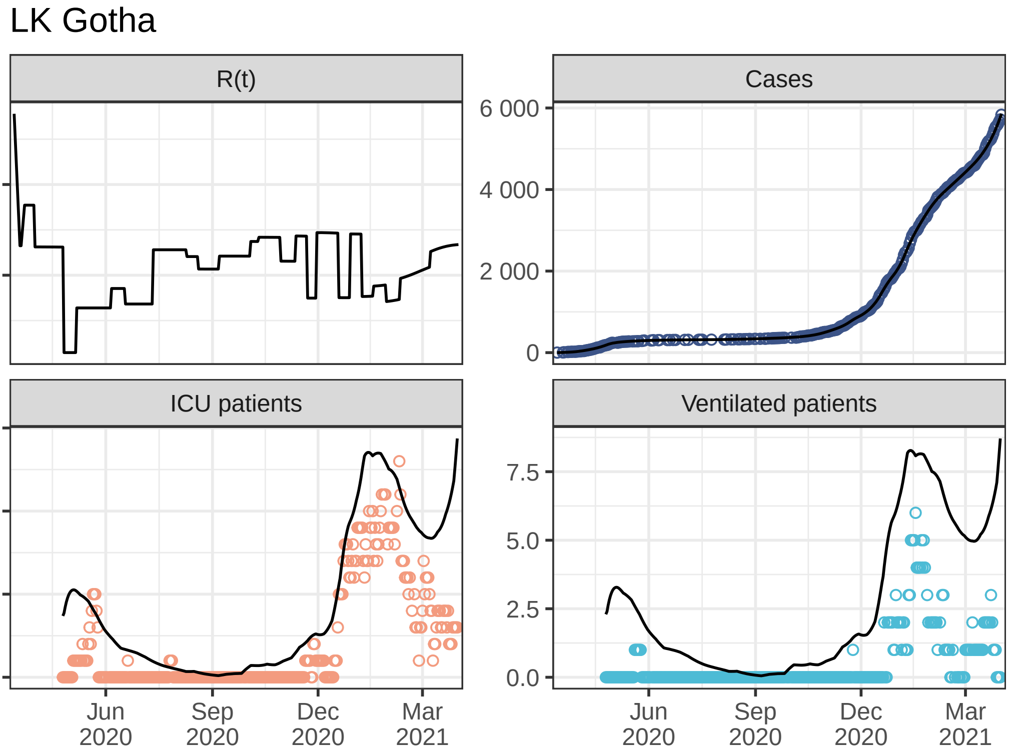
LK Gotha
R(t)
Cases
ICU patients
Ventilated patients
Jun 2020
Sep 2020
Dec 2020
Mar 2021

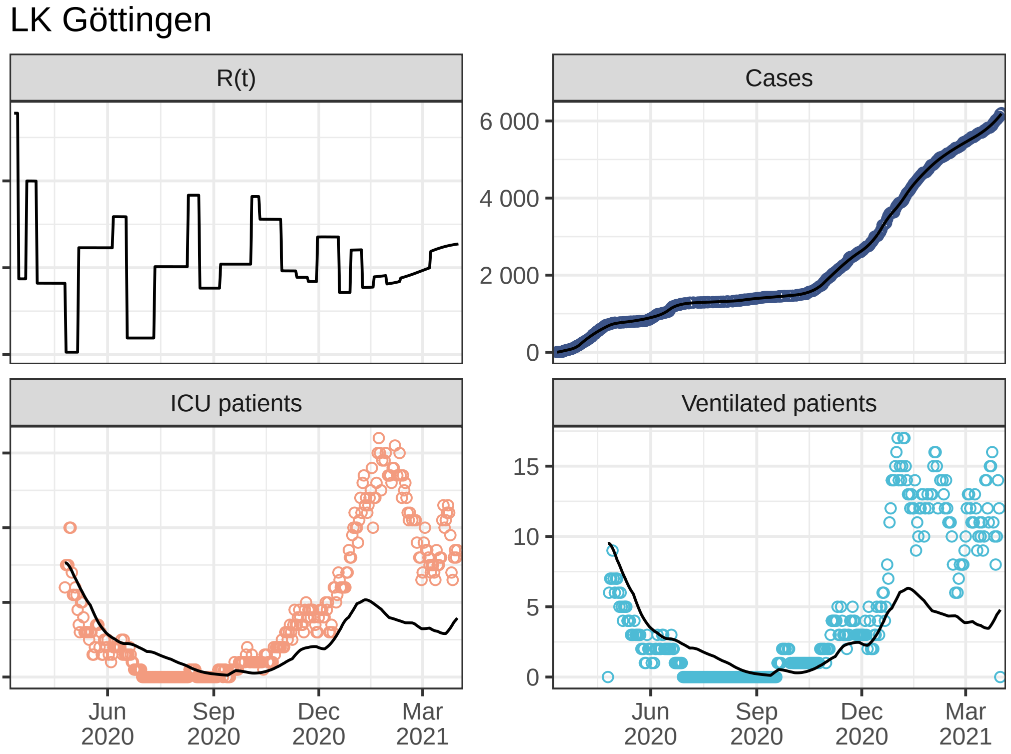
LK Göttingen
R(t)
Cases
ICU patients
Ventilated patients
Jun 2020
Sep 2020
Dec 2020
Mar 2021

LK Grafschaft Bentheim

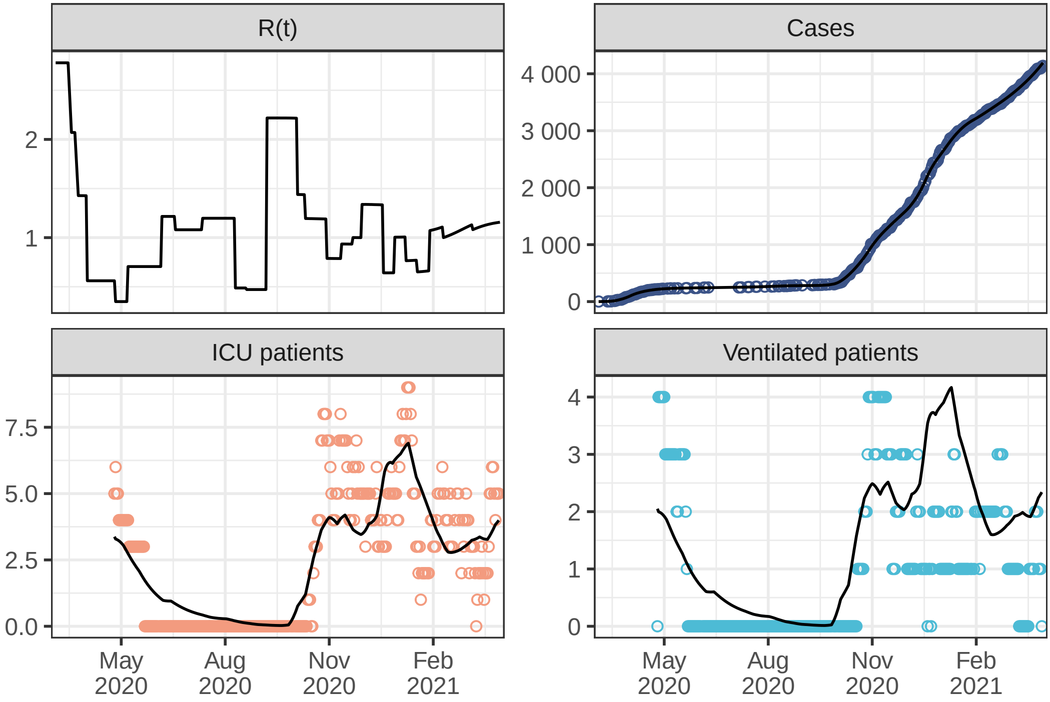

LK Greiz

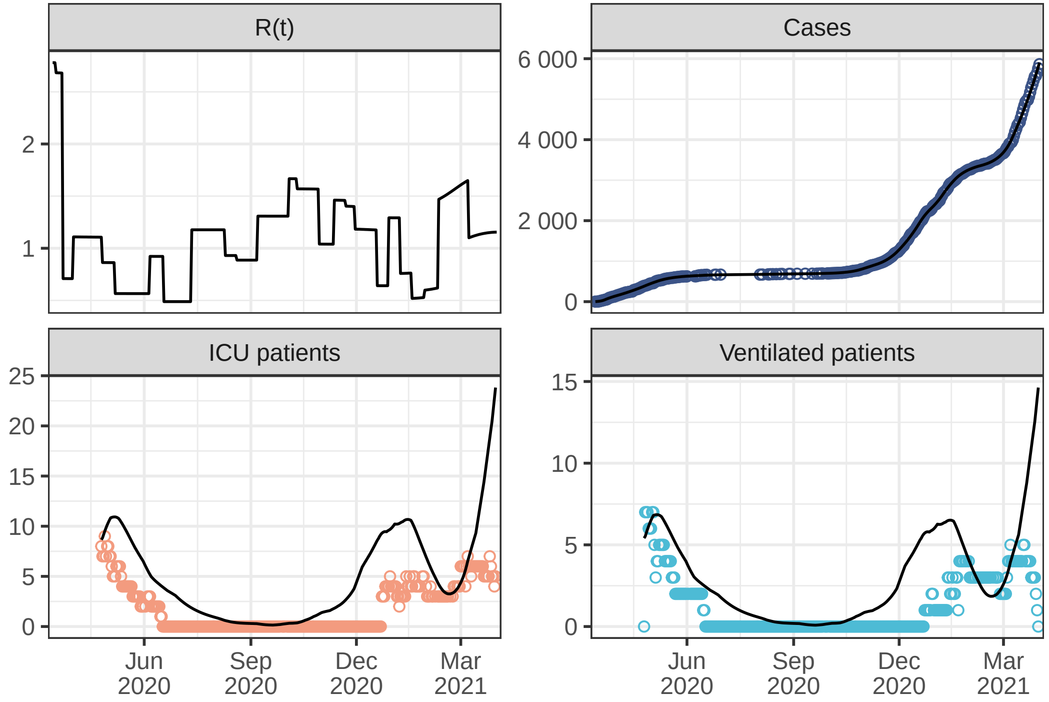

LK Groß−Gerau

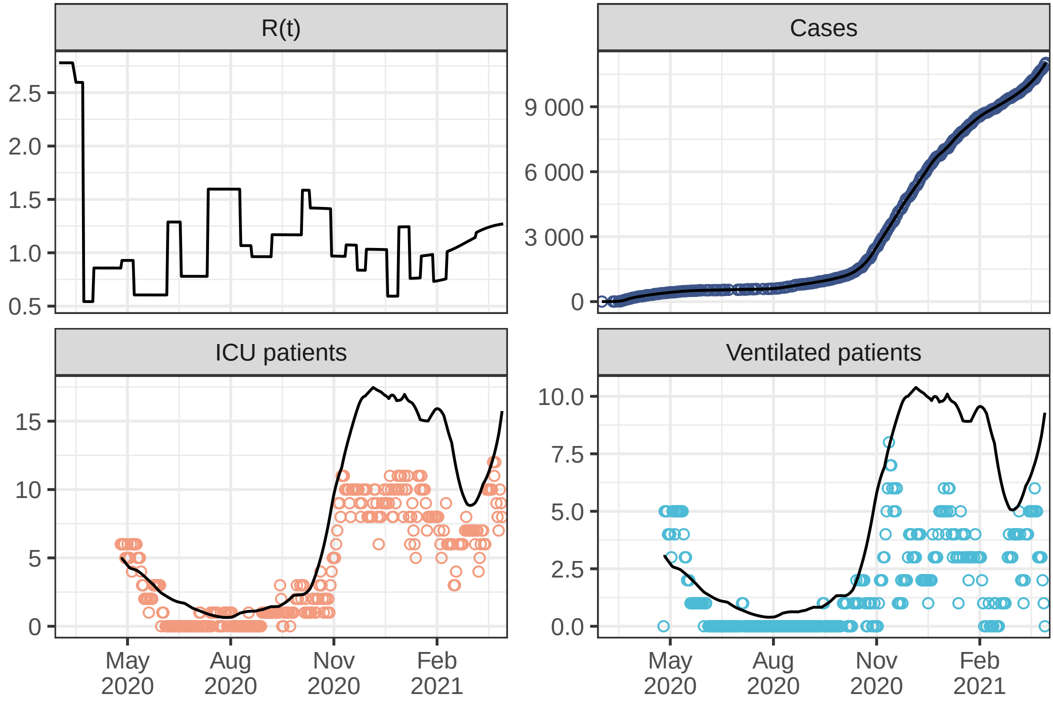

LK Günzburg

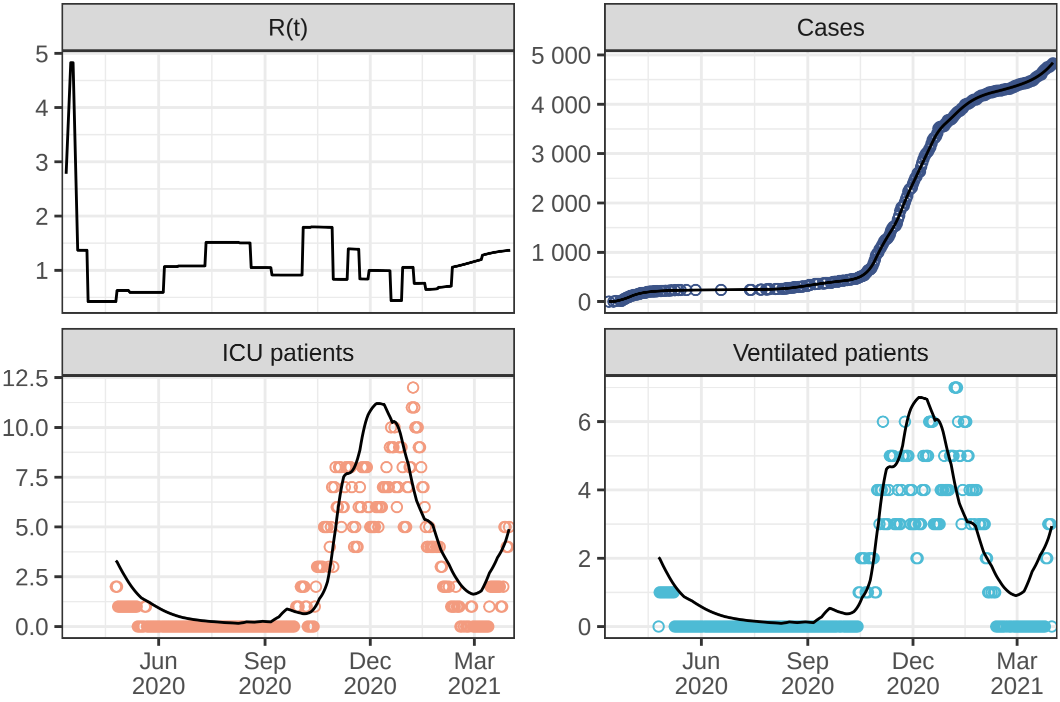

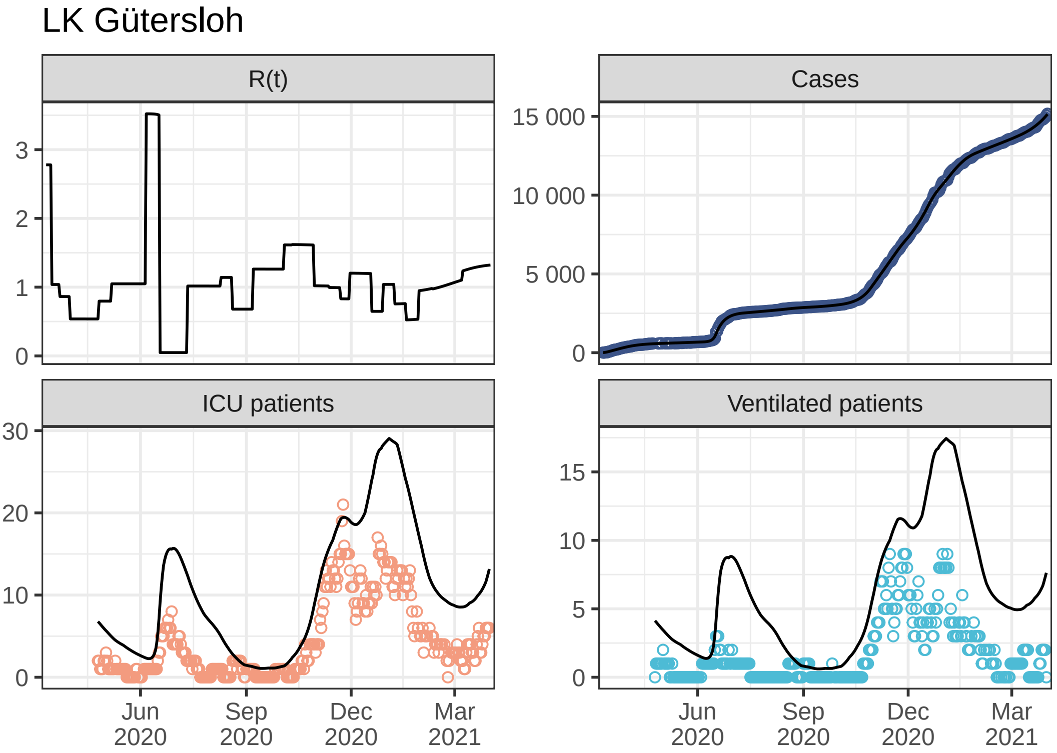

LK Gütersloh
R(t)
Cases
ICU patients
Ventilated patients
0
1
2
3
0
5 000
10 000
15 000
0
10
20
30
0
5
10
15
Jun 2020
Sep 2020
Dec 2020
Mar 2021

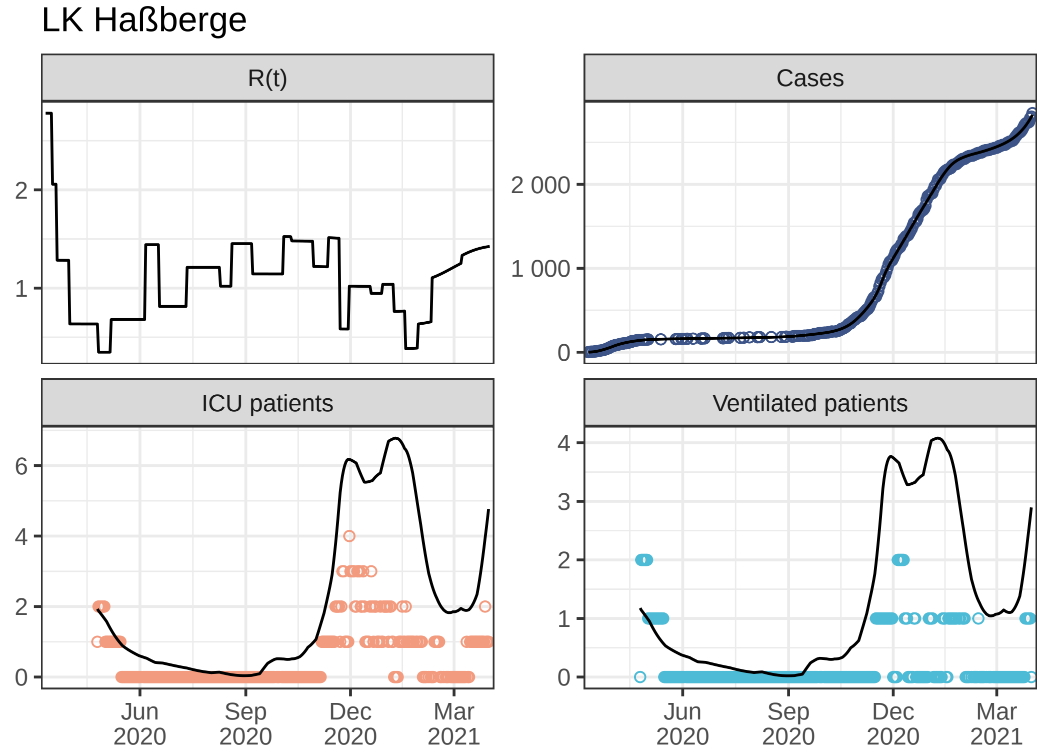

LK Haßberge
R(t)
Cases
ICU patients
Ventilated patients
1
2
0
1 000
2 000
0
2
4
6
0
1
2
3
4
Jun 2020
Sep 2020
Dec 2020
Mar 2021

LK Hameln−Pyrmont

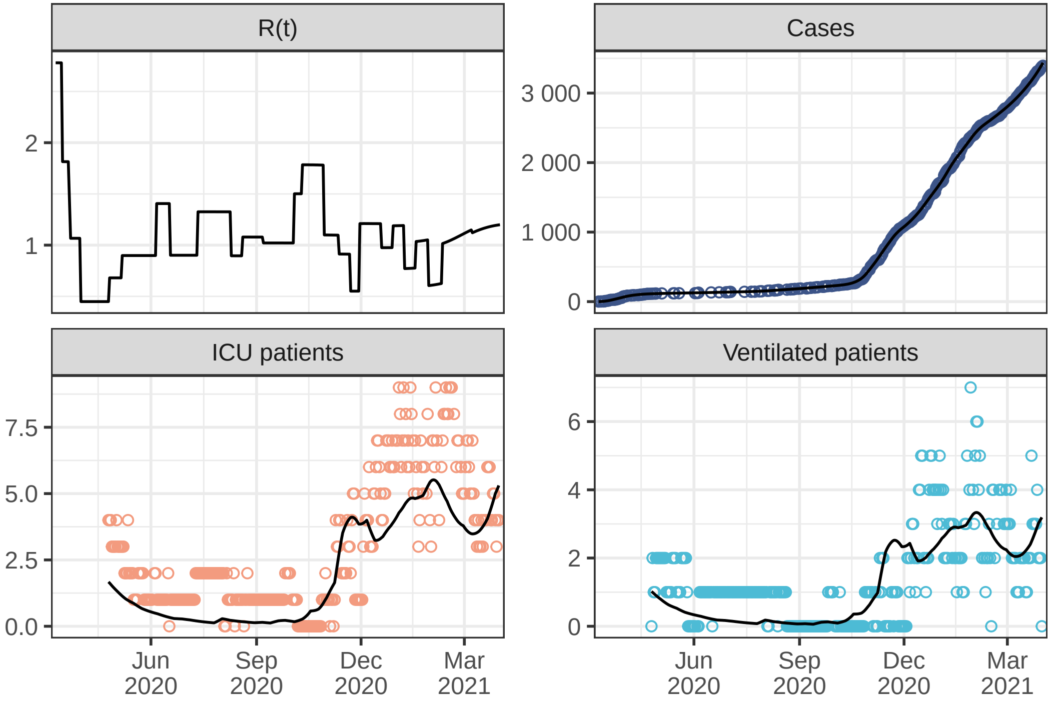

LK Harburg

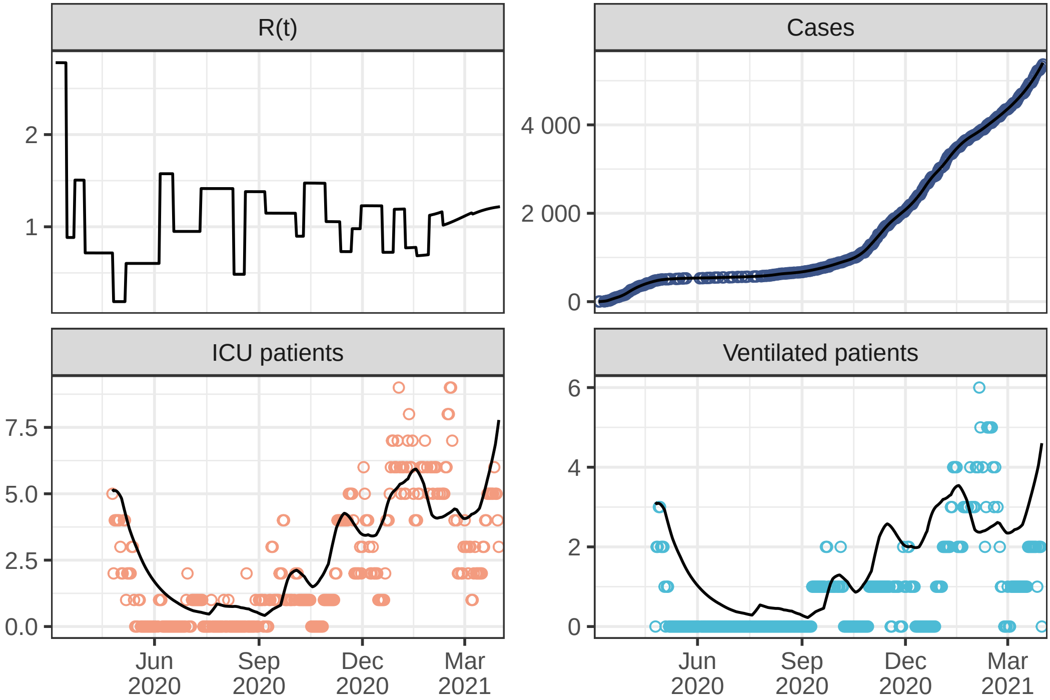

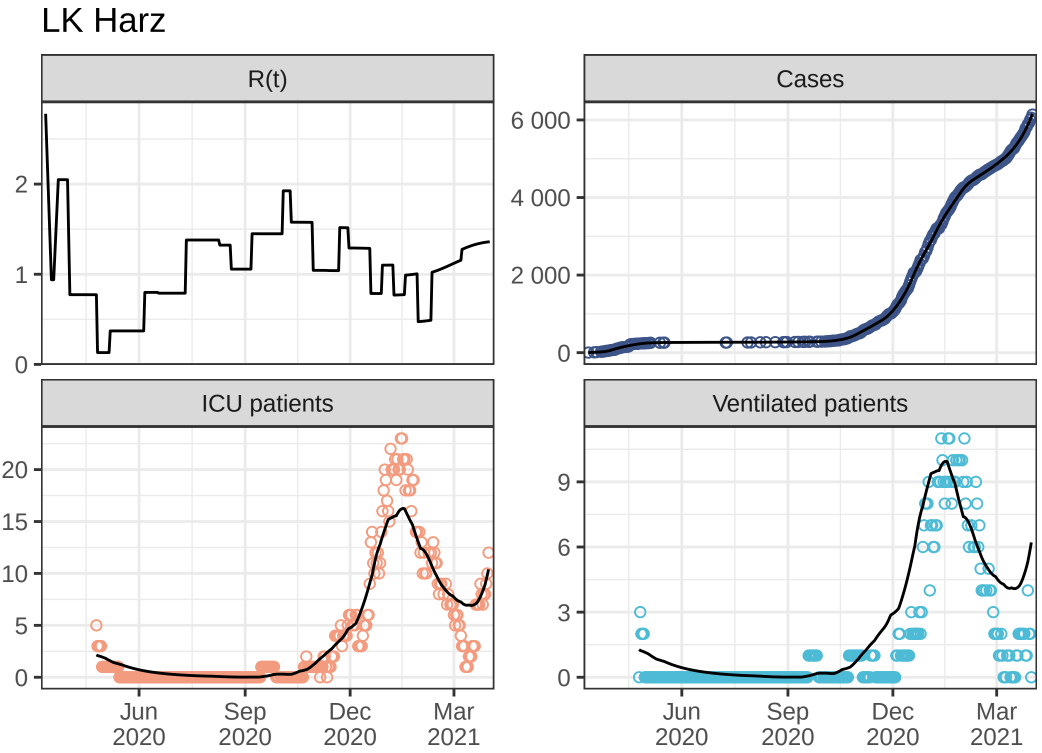
LK Harz
R(t)
Cases
ICU patients
Ventilated patients
0
1
2
0
2 000
4 000
6 000
0
5
10
15
20
0
3
6
9
Jun 2020
Sep 2020
Dec 2020
Mar 2021

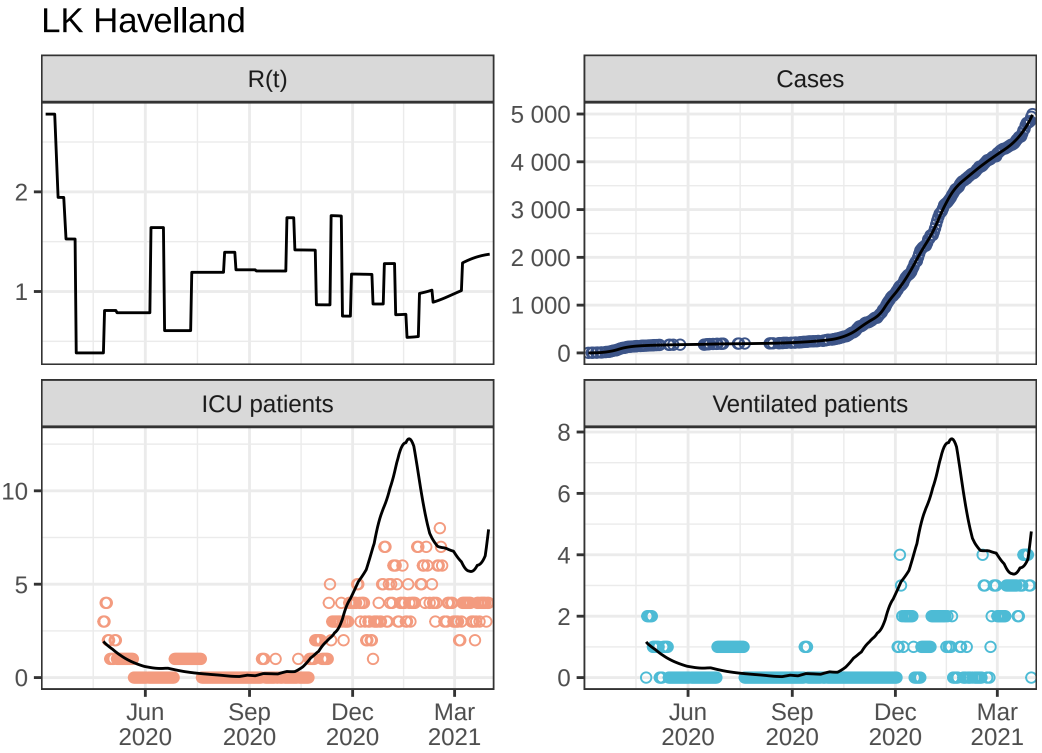
LK Havelland
R(t)
Cases
ICU patients
Ventilated patients
1
2
0
1 000
2 000
3 000
4 000
5 000
0
5
10
0
2
4
6
8
Jun 2020
Sep 2020
Dec 2020
Mar 2021

LK Heidekreis

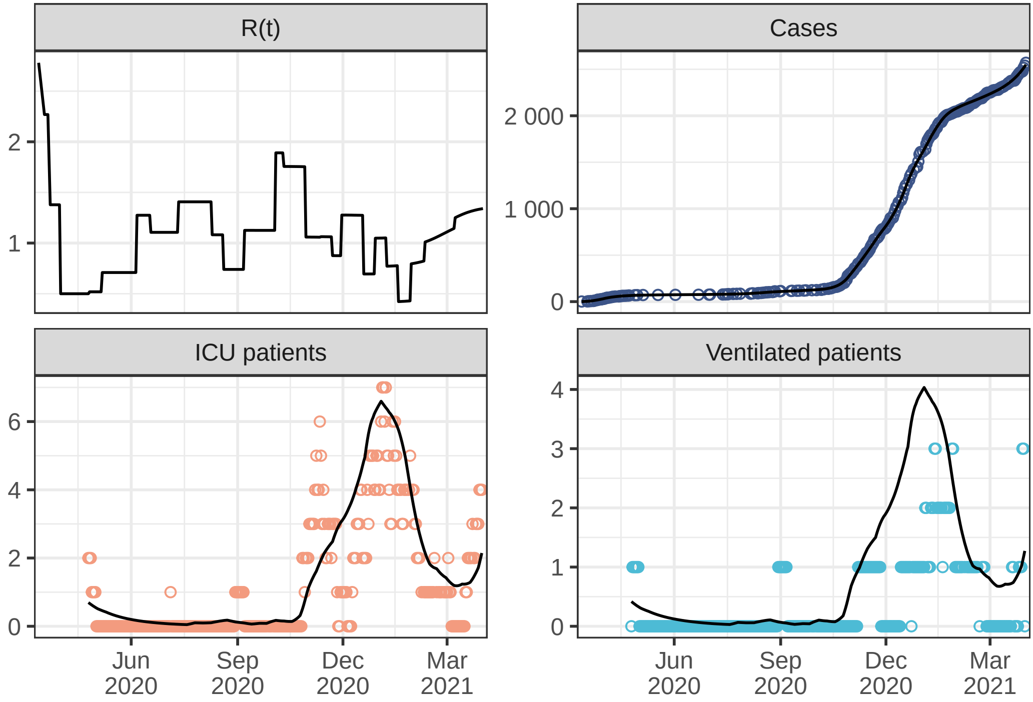

LK Heidenheim

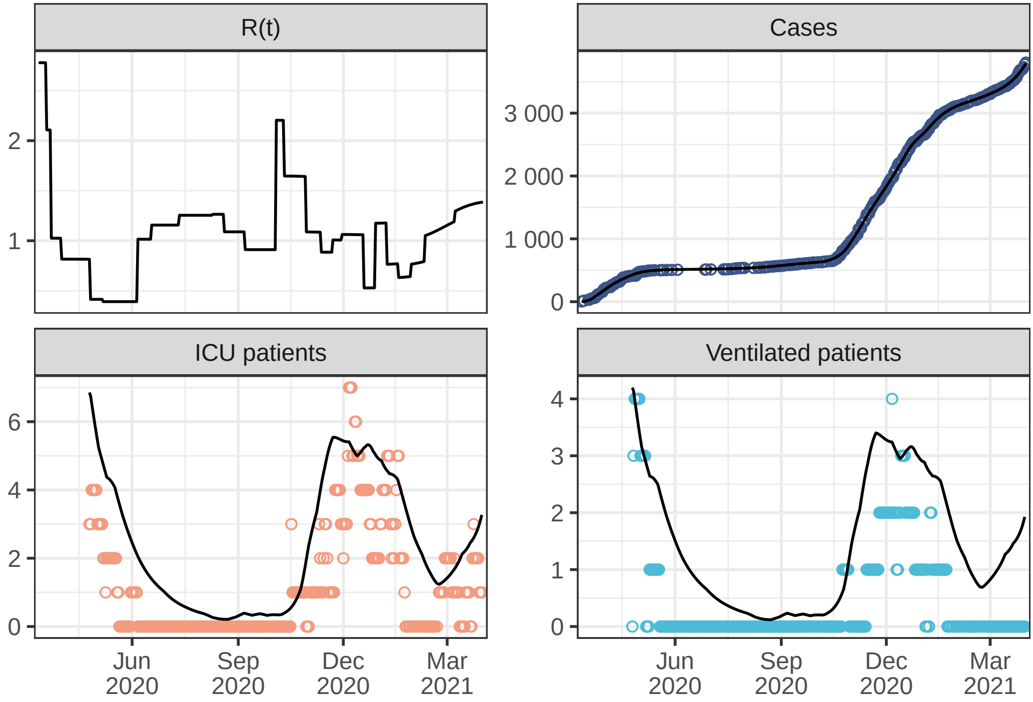

LK Heilbronn

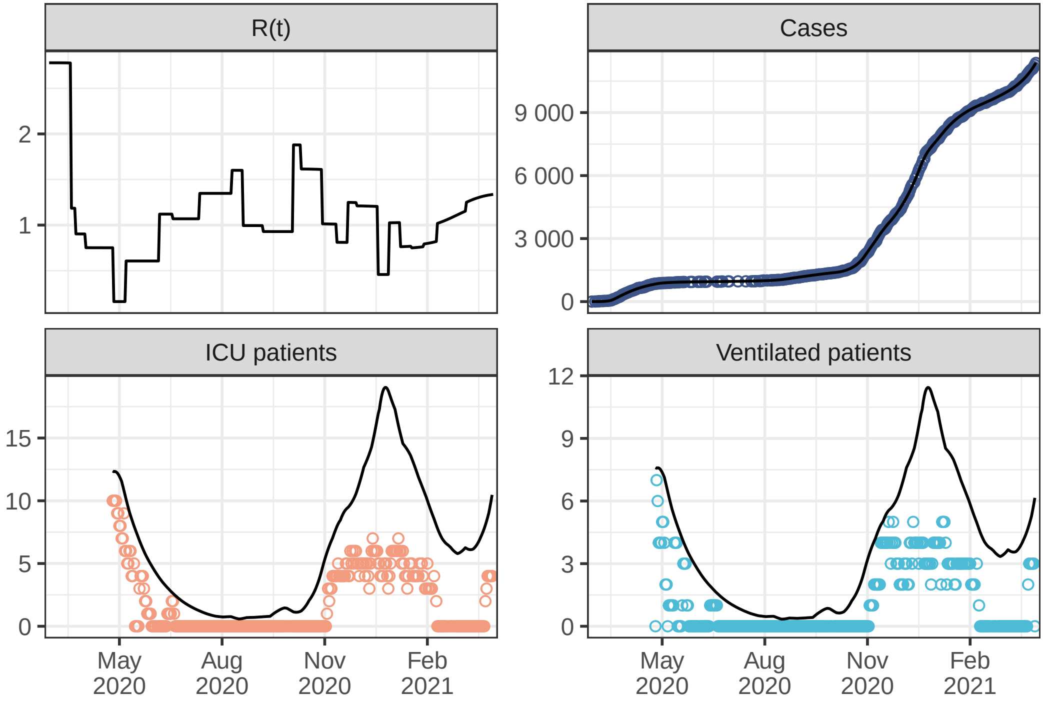

LK Heinsberg

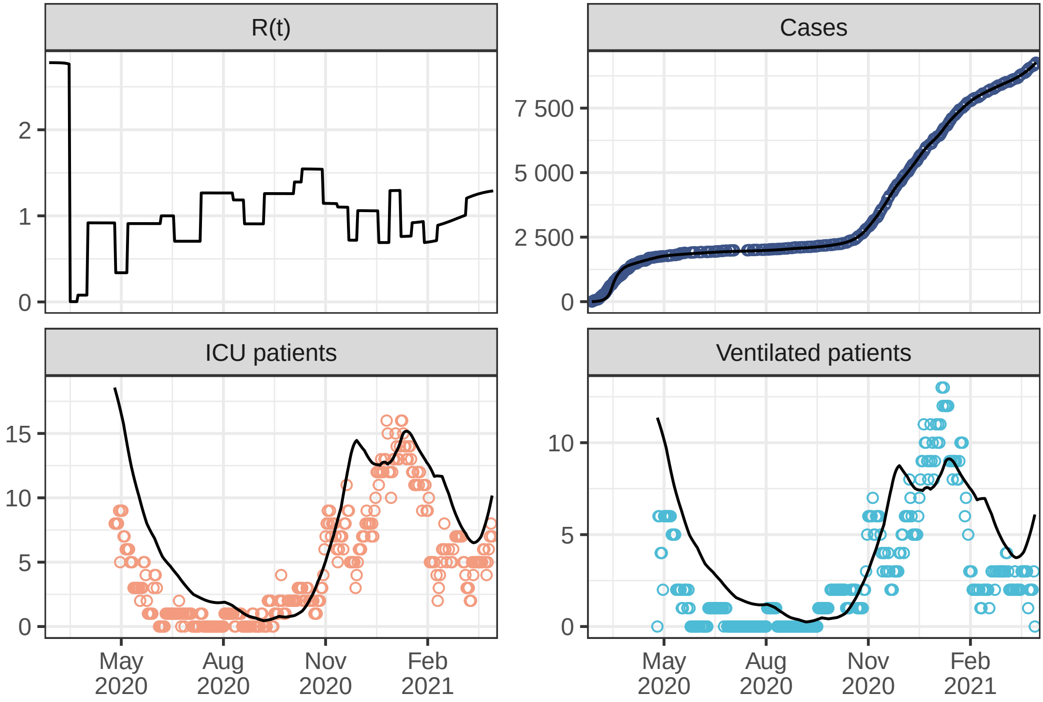

LK Helmstedt

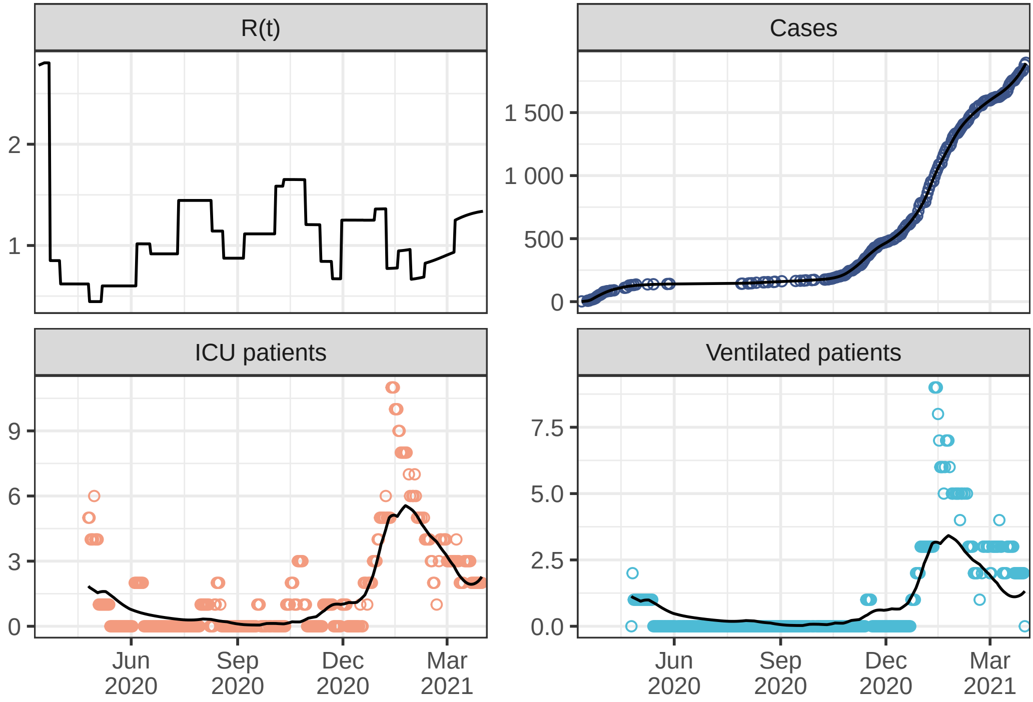

LK Herford

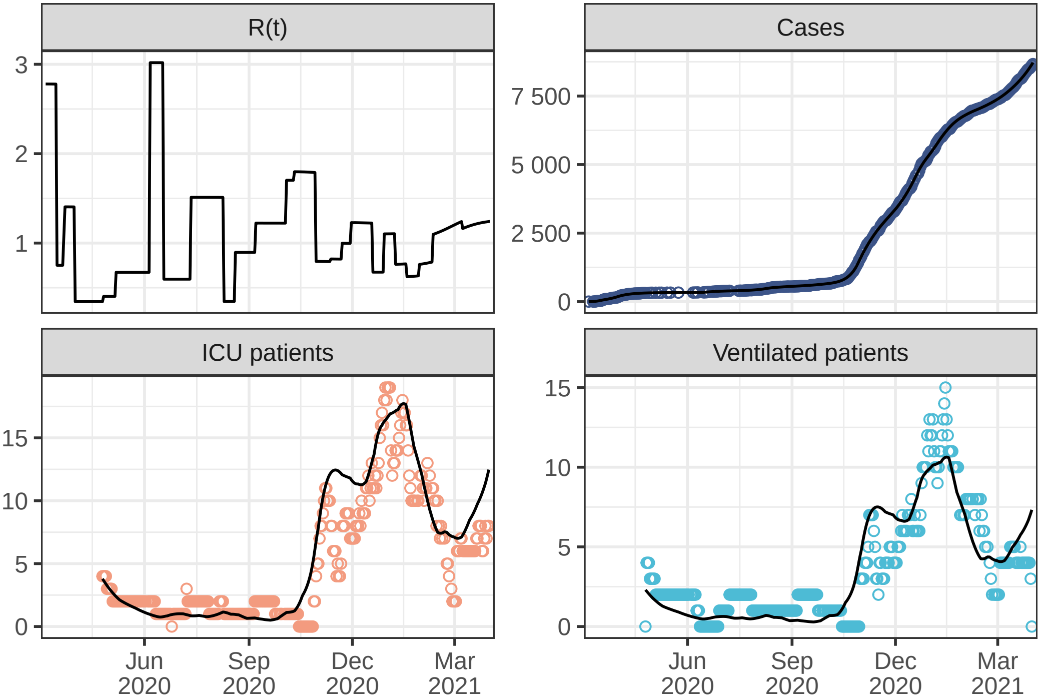

LK Hersfeld−Rotenburg

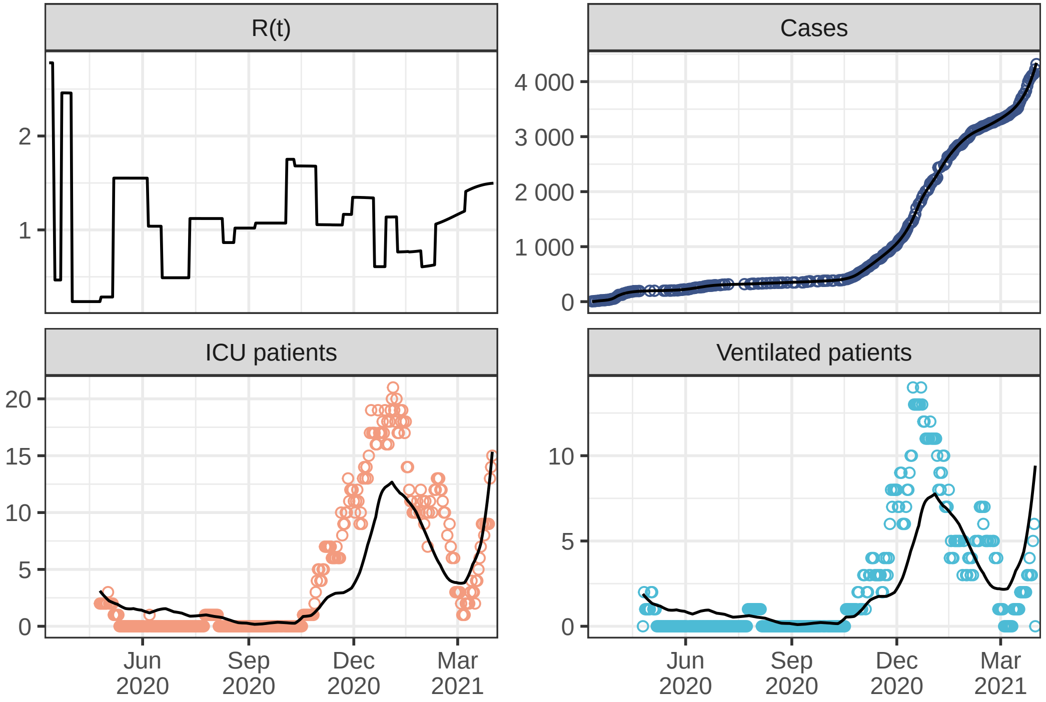

LK Herzogtum Lauenburg

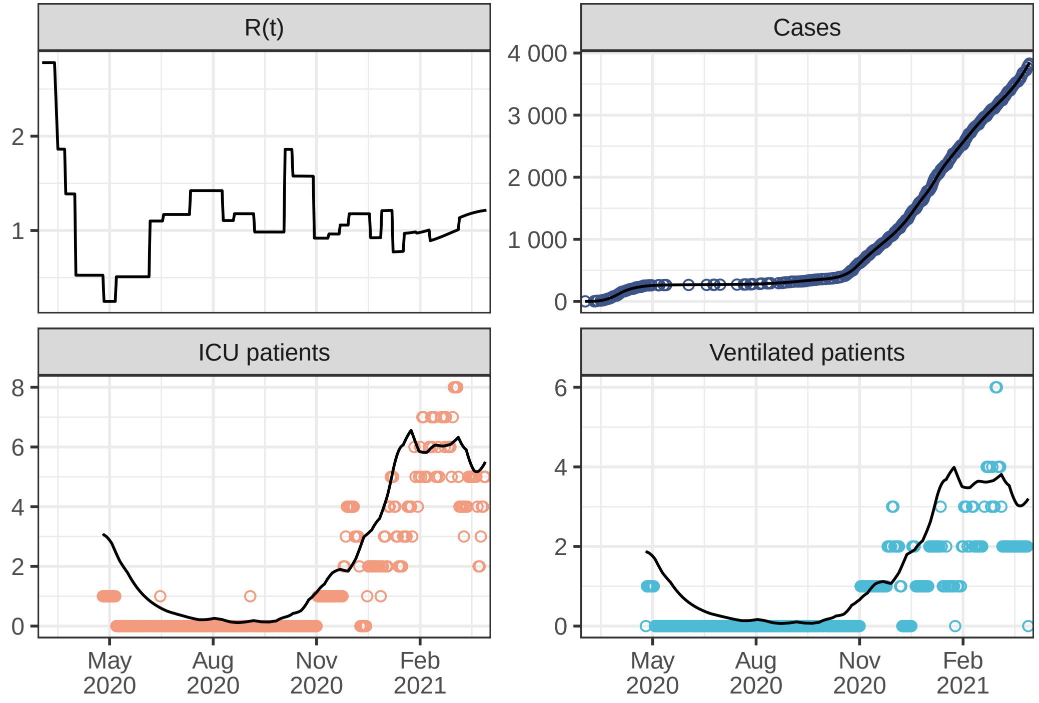

LK Hildburghausen

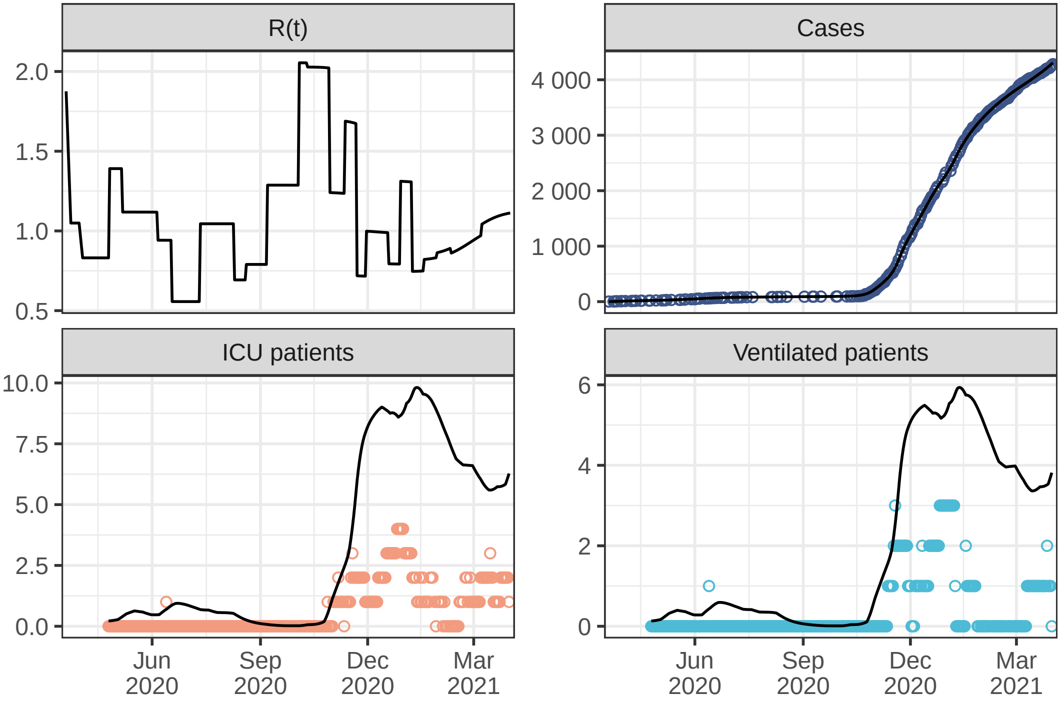

LK Hildesheim

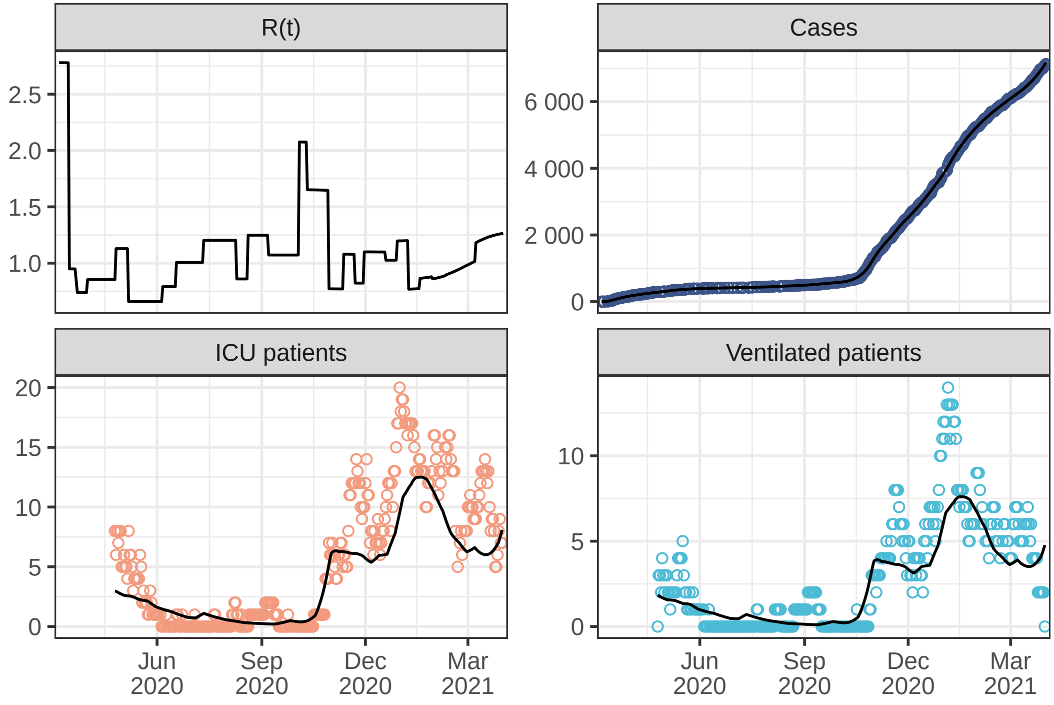

LK Hochsauerlandkreis

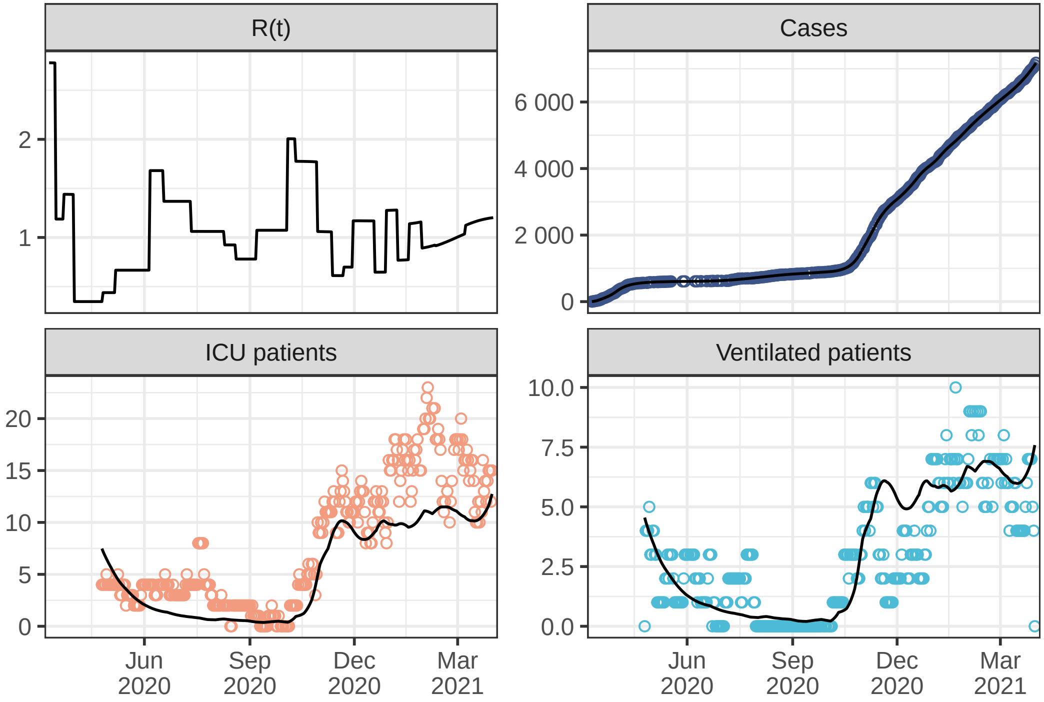

LK Hochtaunuskreis

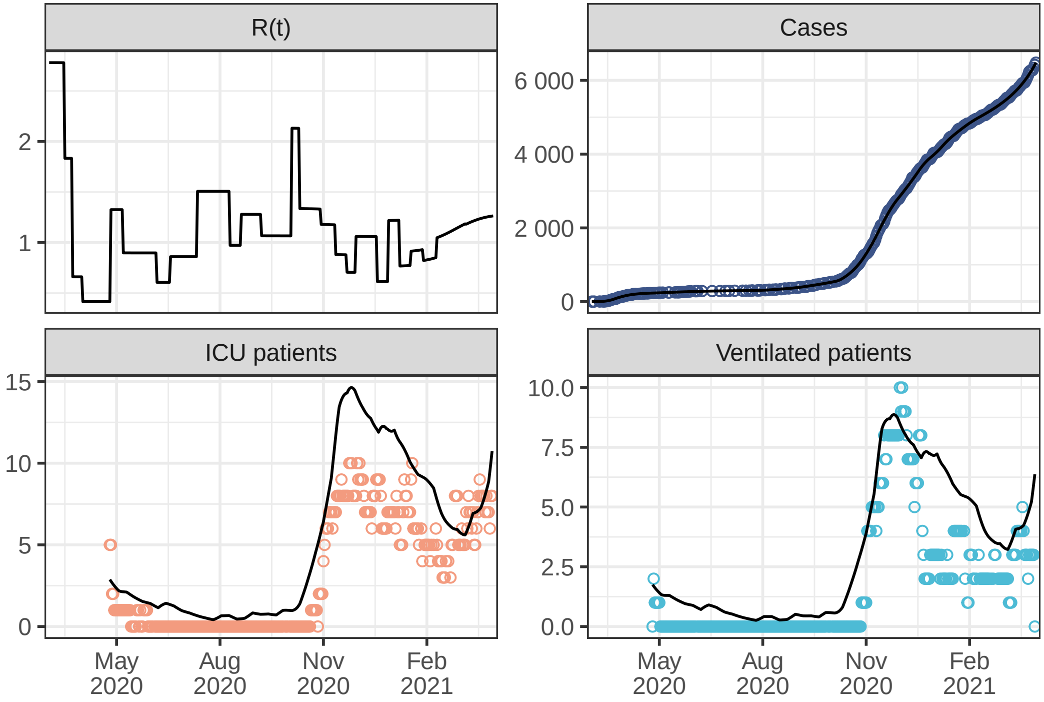

LK Hof

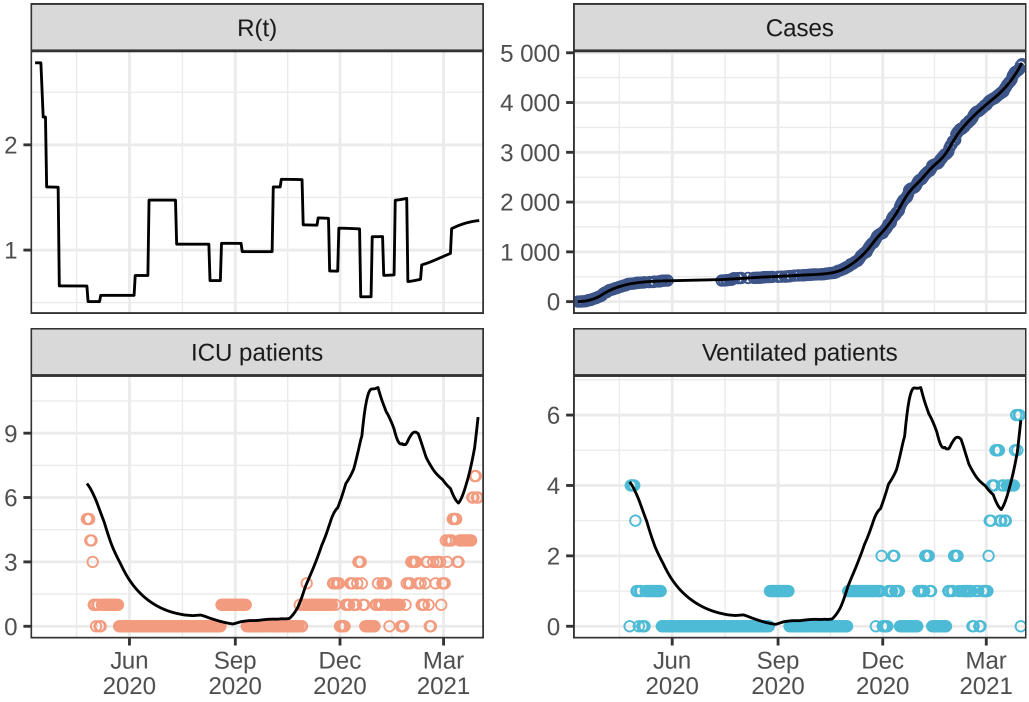

LK Hohenlohekreis

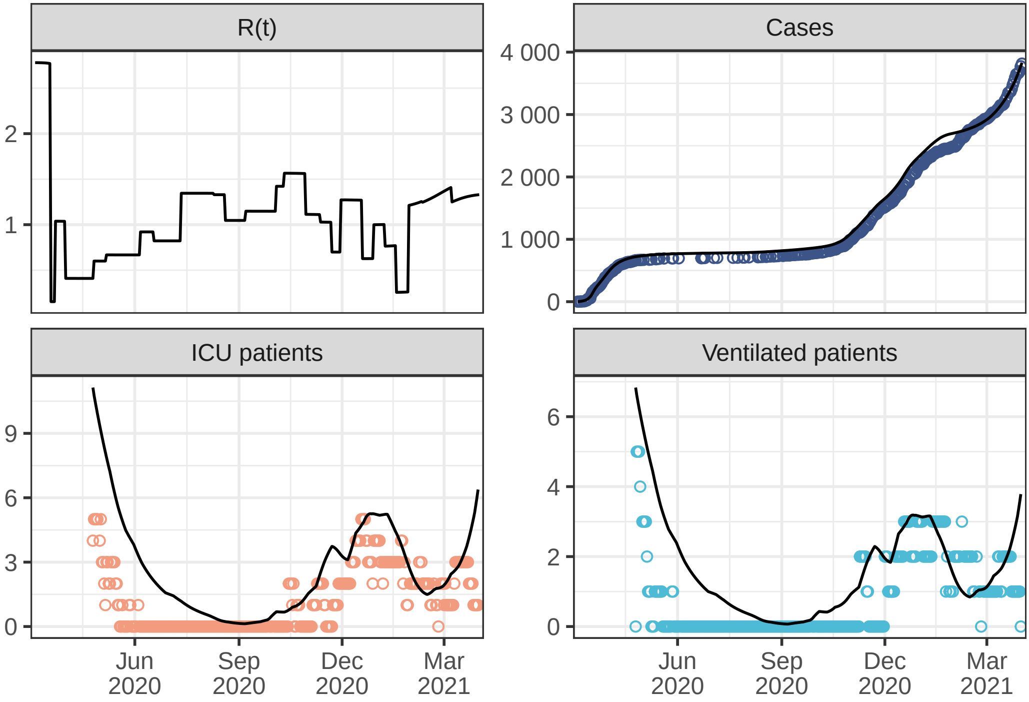

LK Holzminden

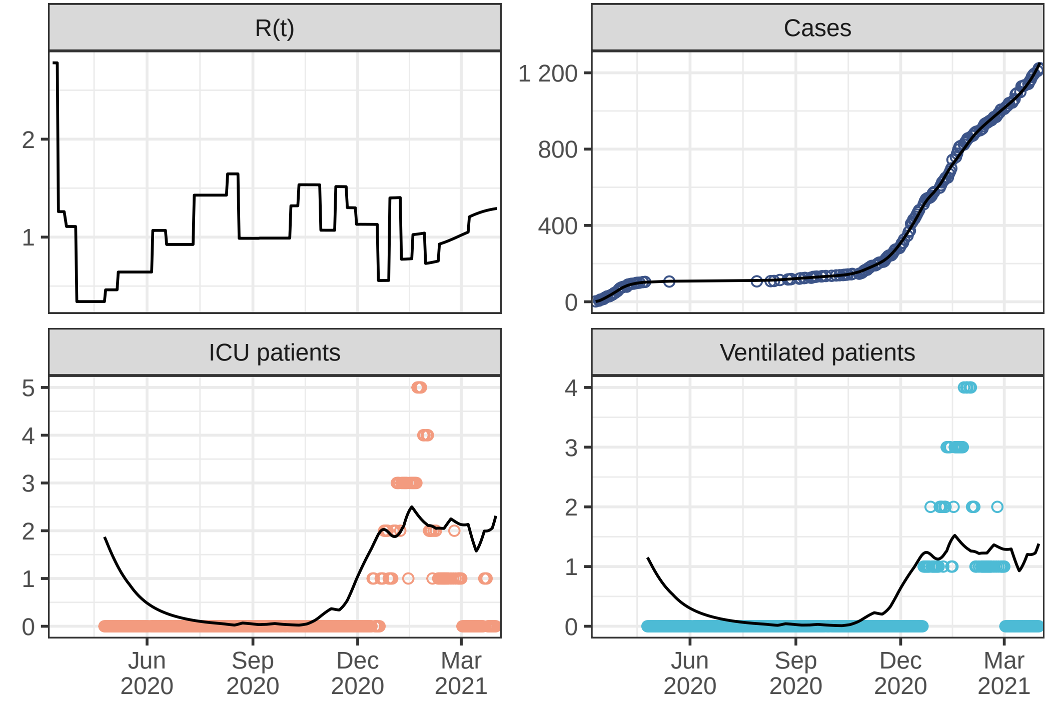

LK Höxter

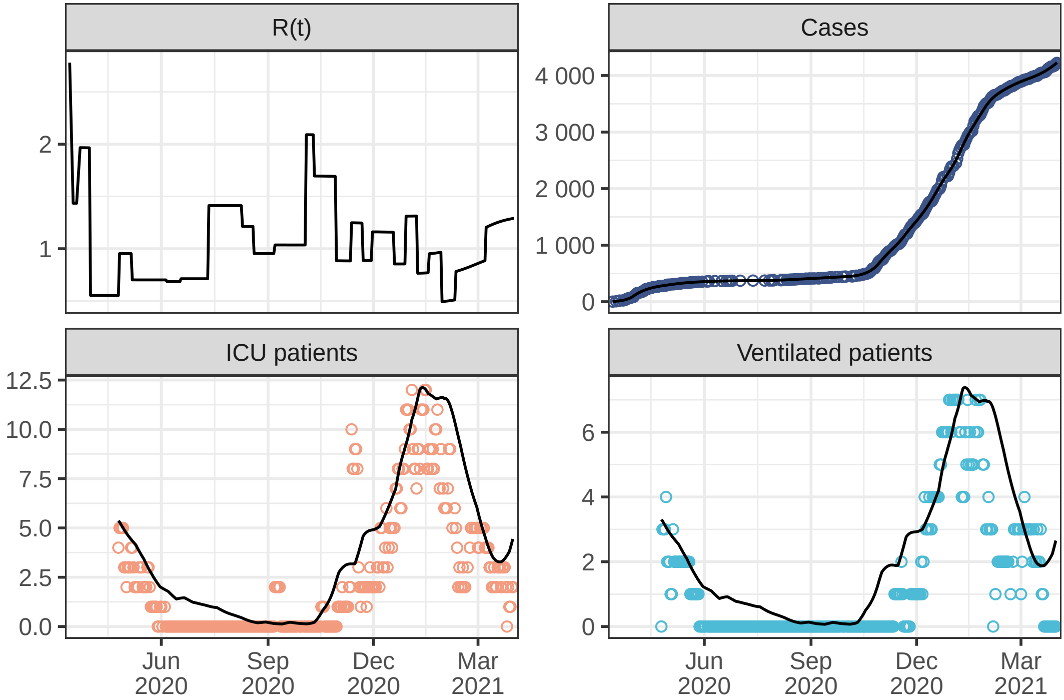

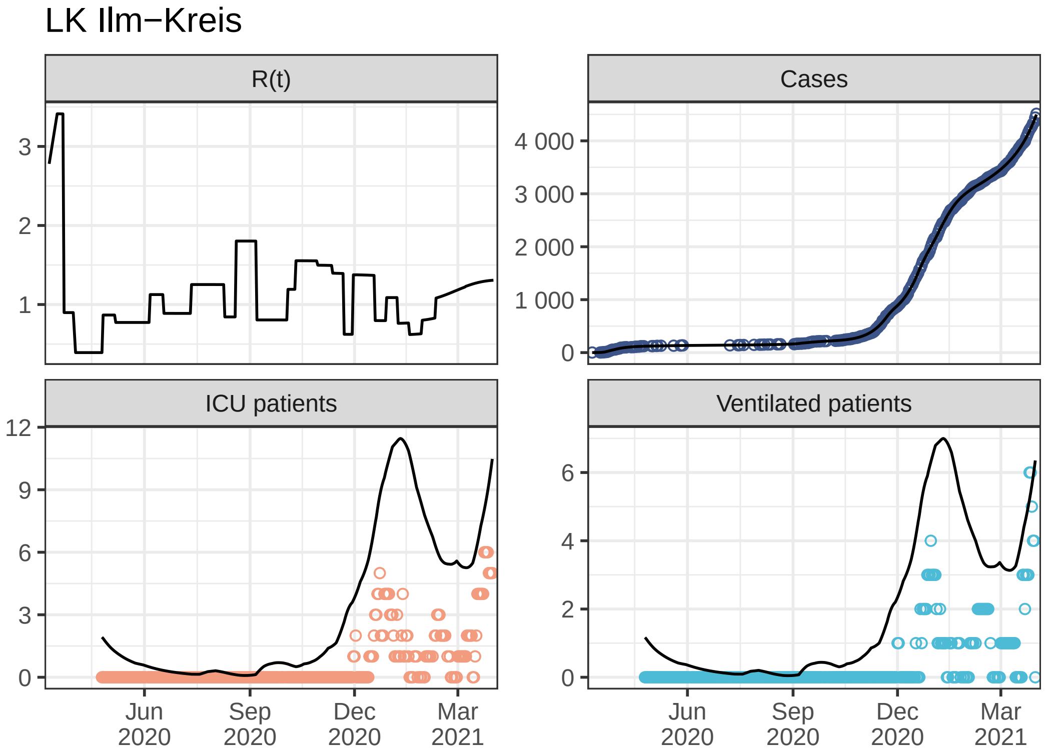
LK Ilm−Kreis
R(t)
Cases
ICU patients
Ventilated patients
1
2
3
0
1 000
2 000
3 000
4 000
0
3
6
9
12
0
2
4
6
Jun 2020
Sep 2020
Dec 2020
Mar 2021

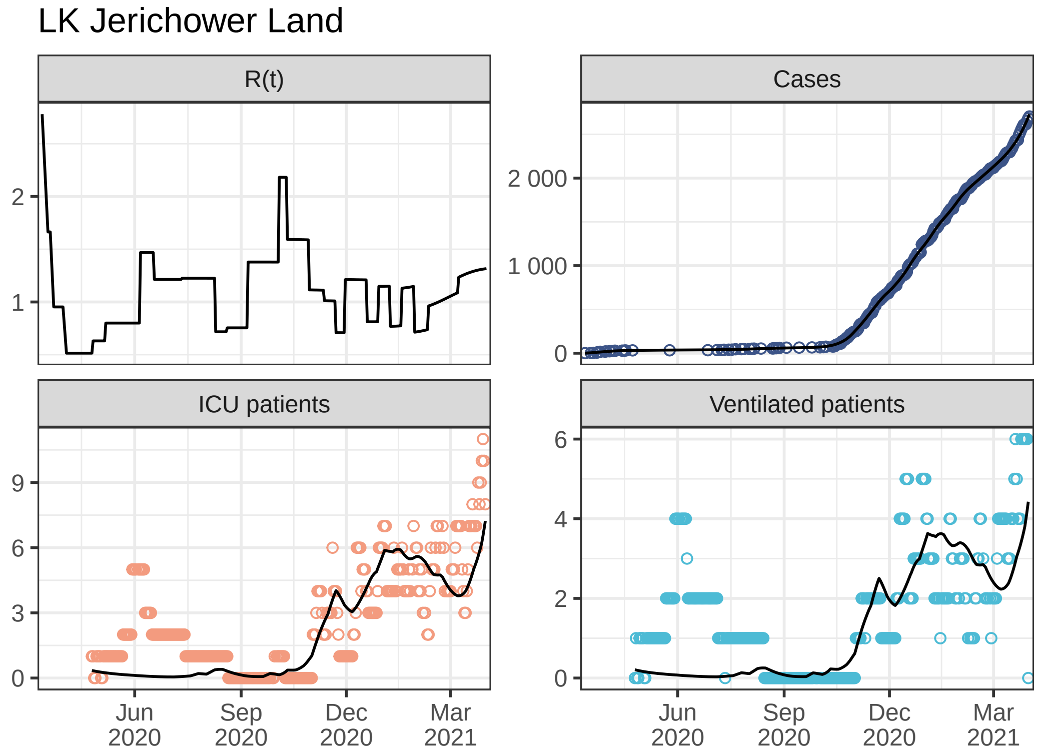
LK Jerichower Land
R(t)
Cases
ICU patients
Ventilated patients
1
2
0
1 000
2 000
0
3
6
9
0
2
4
6
Jun 2020
Sep 2020
Dec 2020
Mar 2021

LK Kaiserslautern

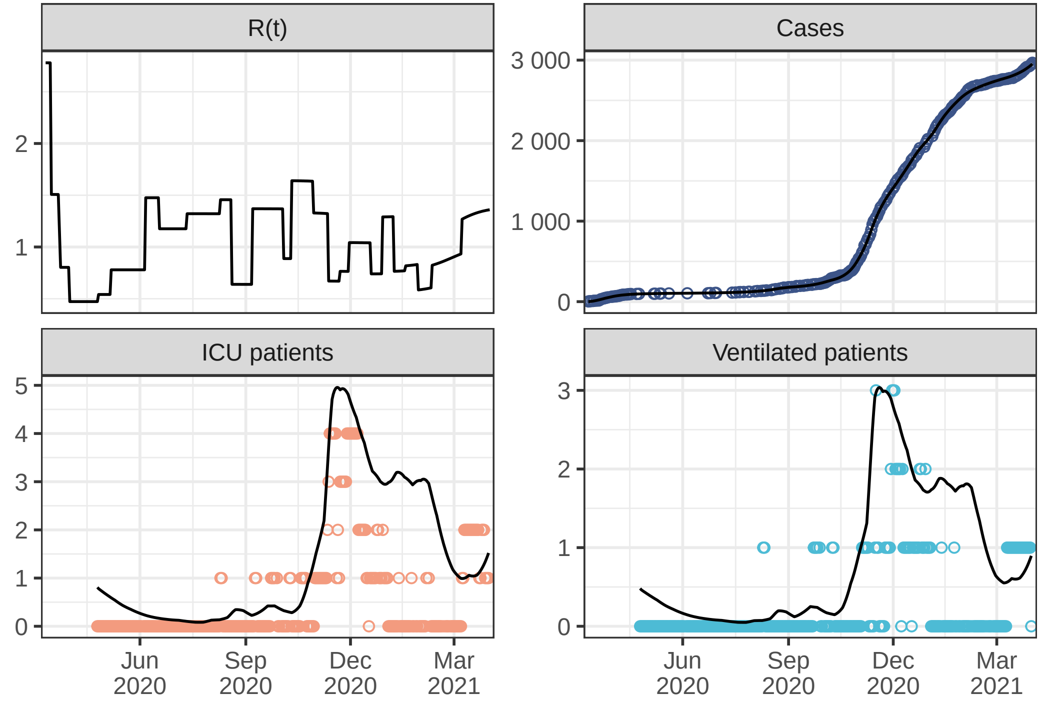

LK Karlsruhe

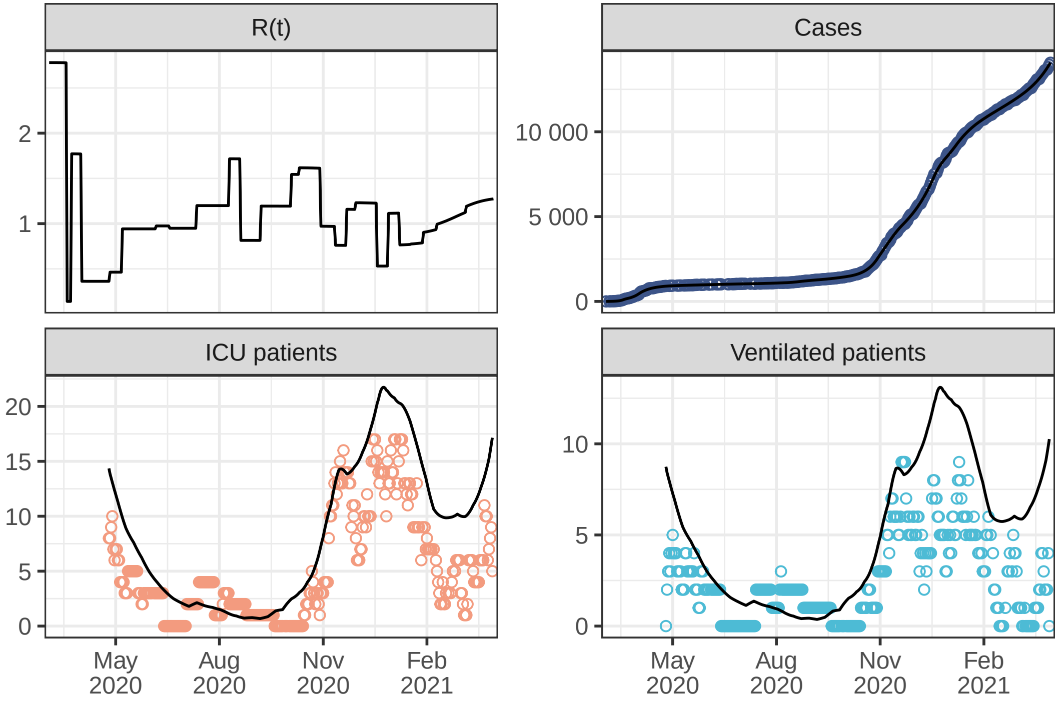

LK Kassel

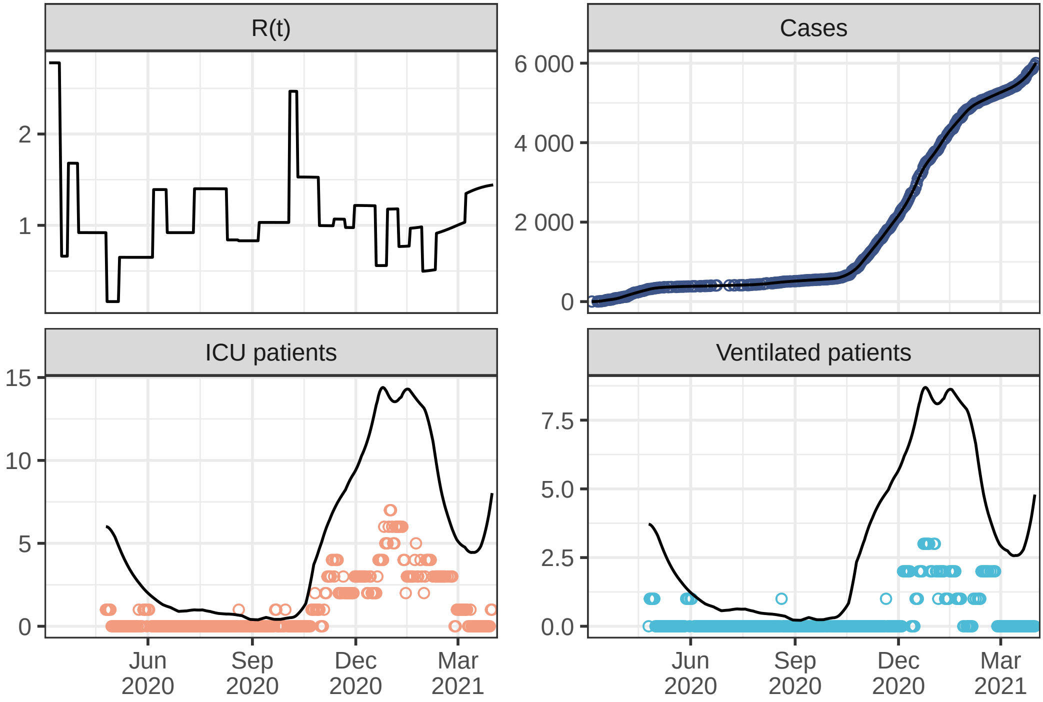

LK Kelheim

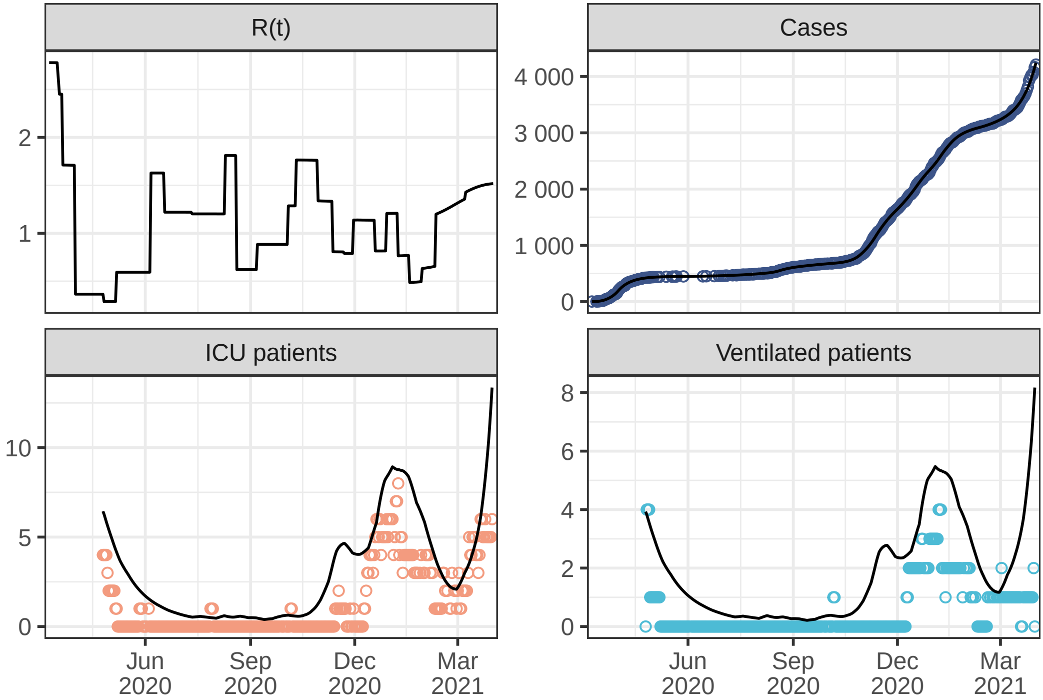

LK Kitzingen

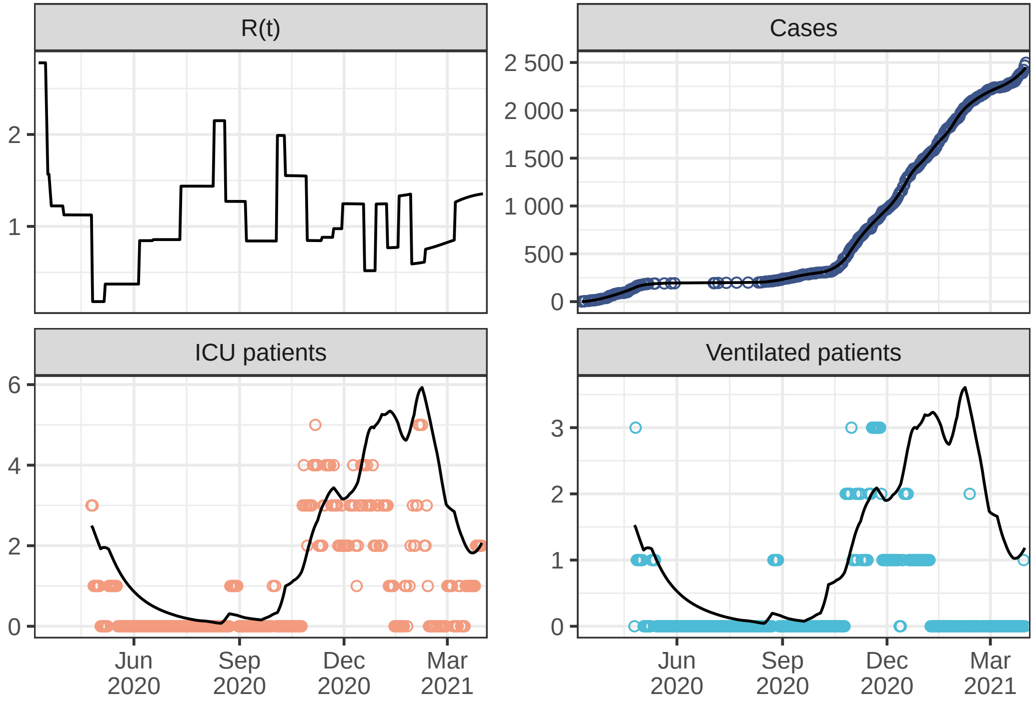

LK Kleve

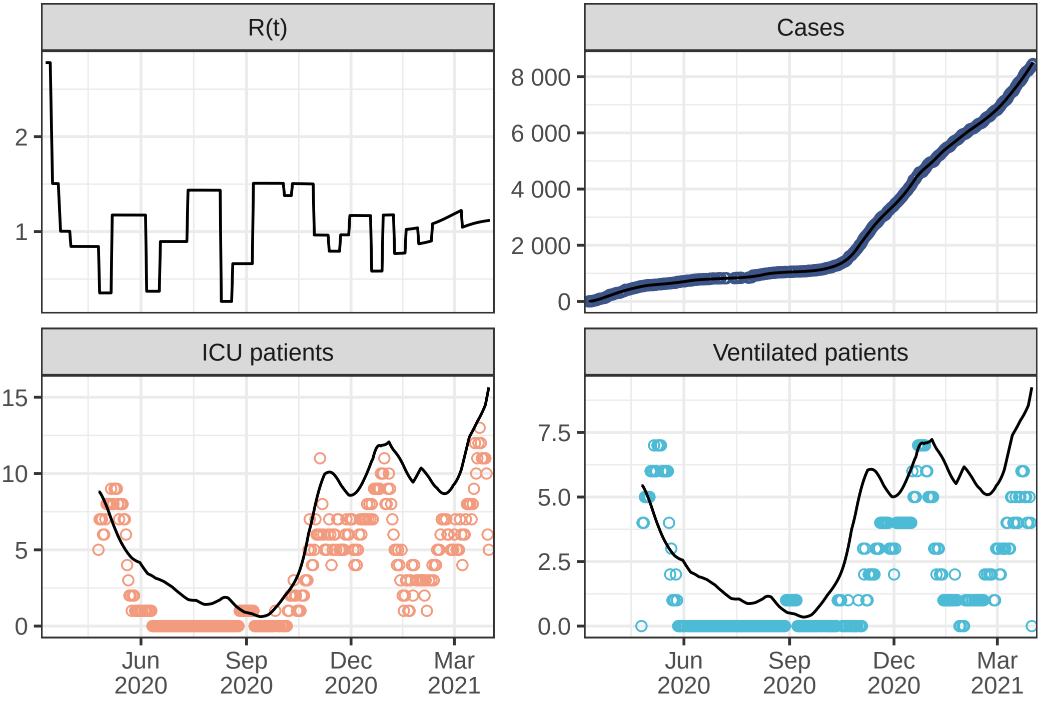

LK Konstanz

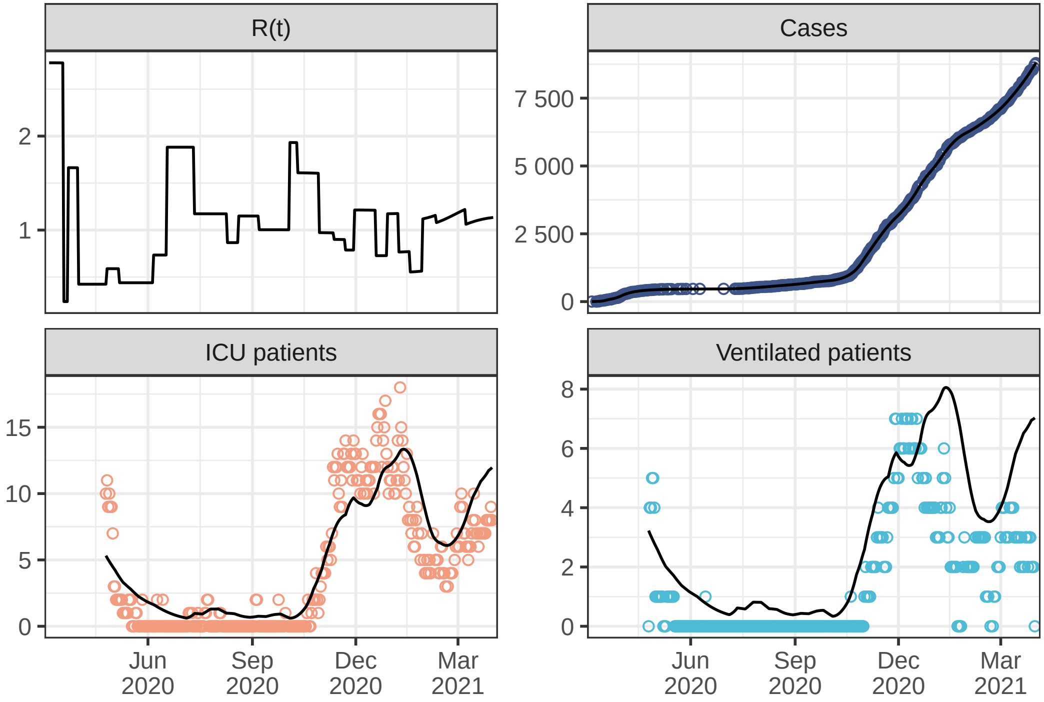

LK Kronach

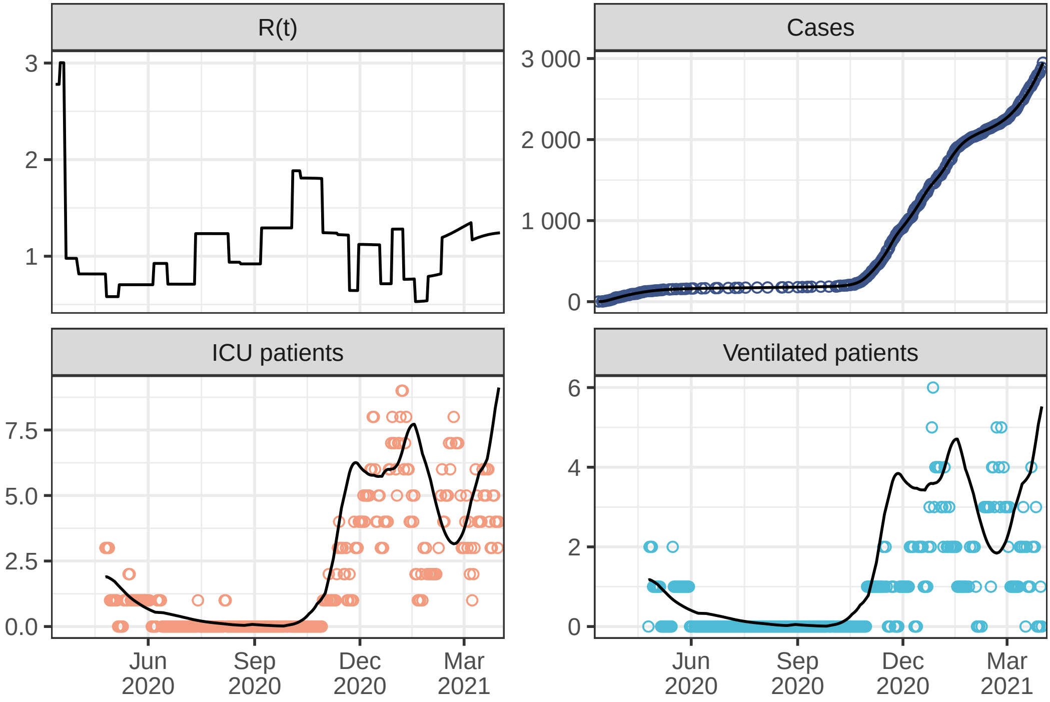

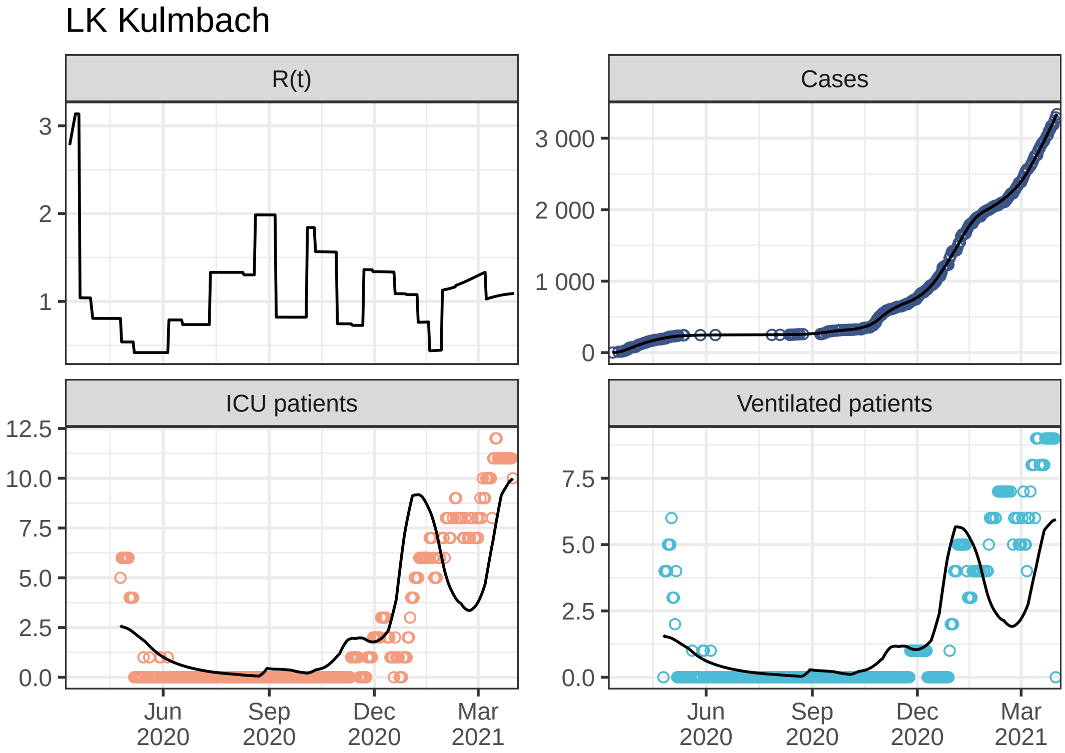
LK Kulmbach
R(t)
Cases
ICU patients
Ventilated patients
3
2
1
3 000
2 000
1 000
0
12.5
10.0
7.5
5.0
2.5
0.0
Jun 2020
Sep 2020
Dec 2020
Mar 2021

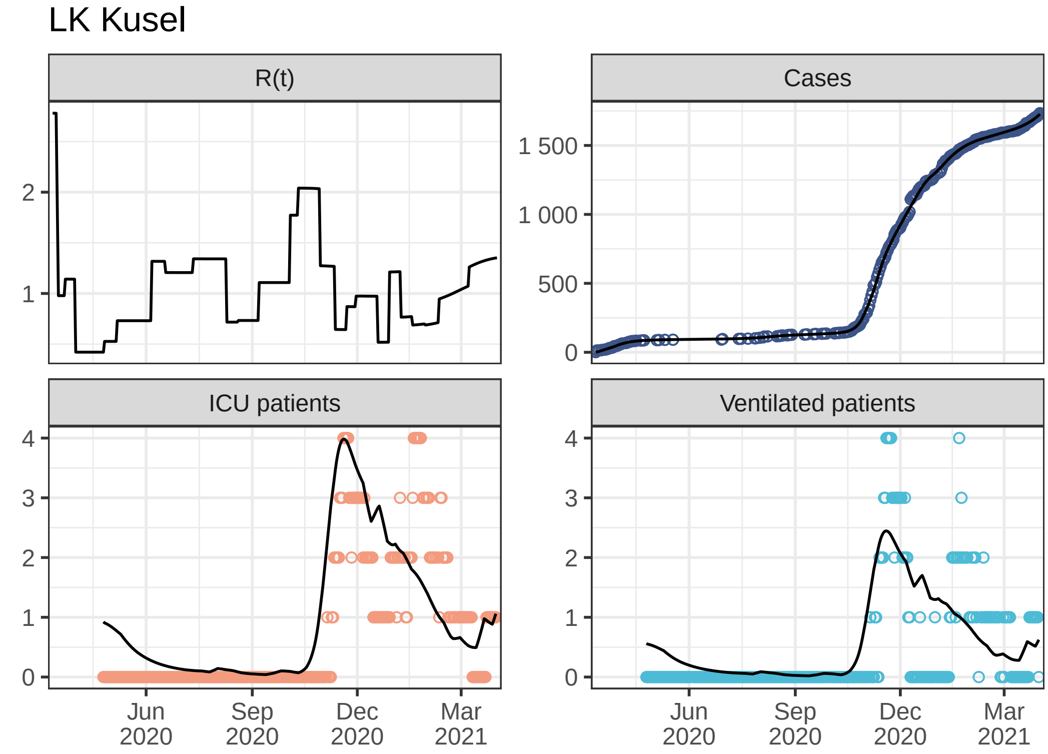
LK Kusel
R(t)
Cases
ICU patients
Ventilated patients
2
1
1 500
1 000
500
0
4
3
2
1
0
Jun 2020
Sep 2020
Dec 2020
Mar 2021

LK Kyffhäuserkreis

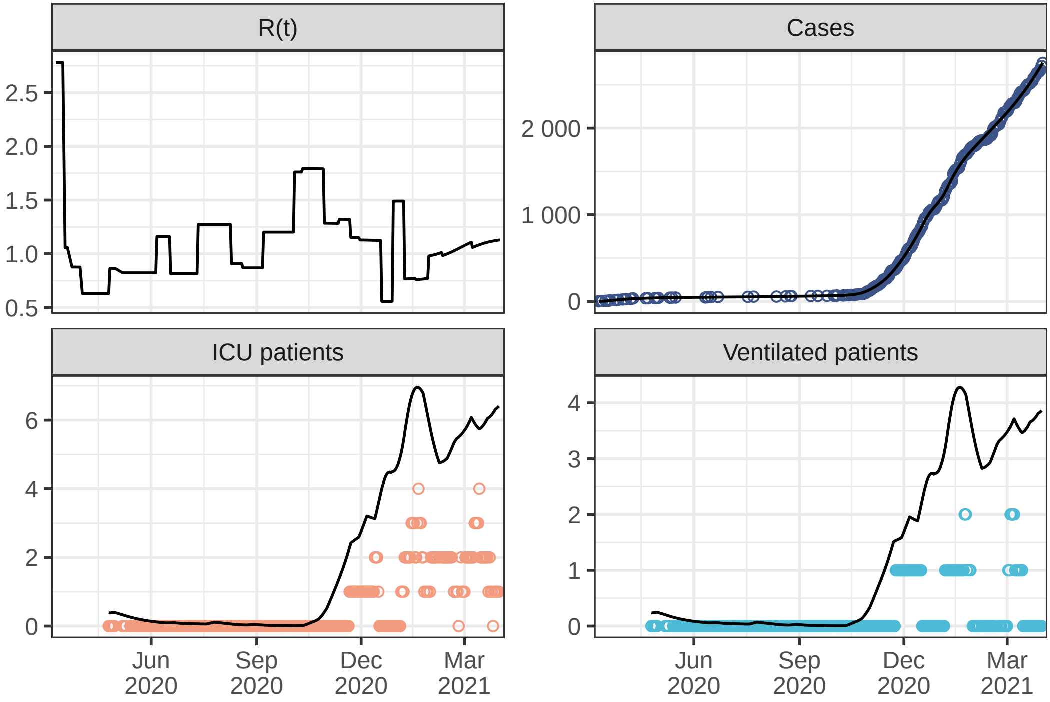

LK Lahn−Dill−Kreis

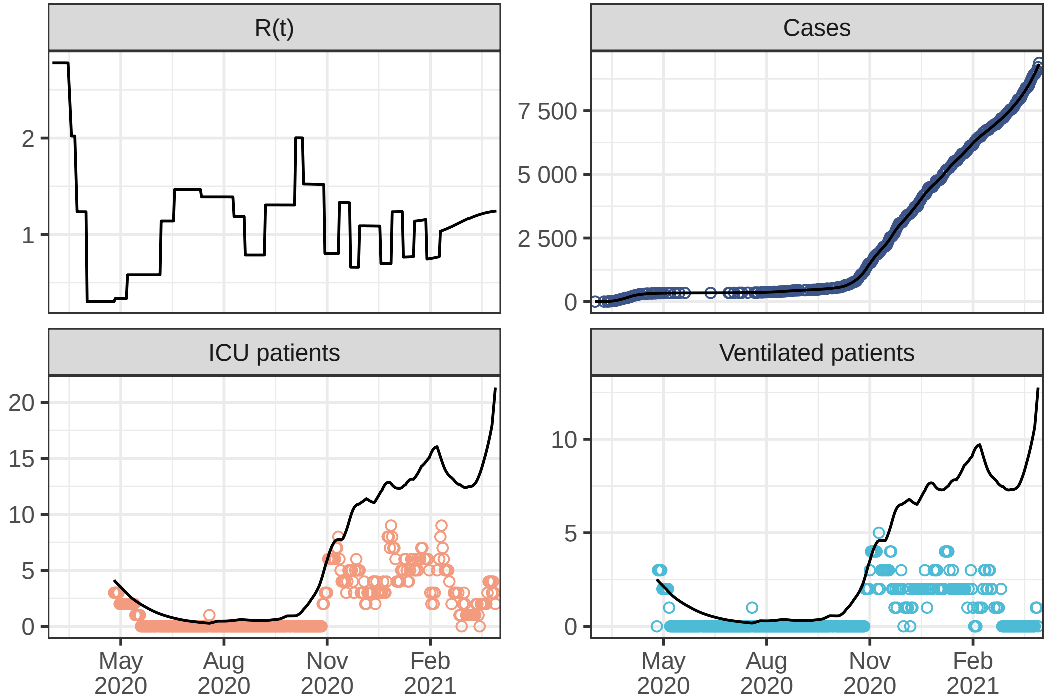

LK Landsberg a.Lech

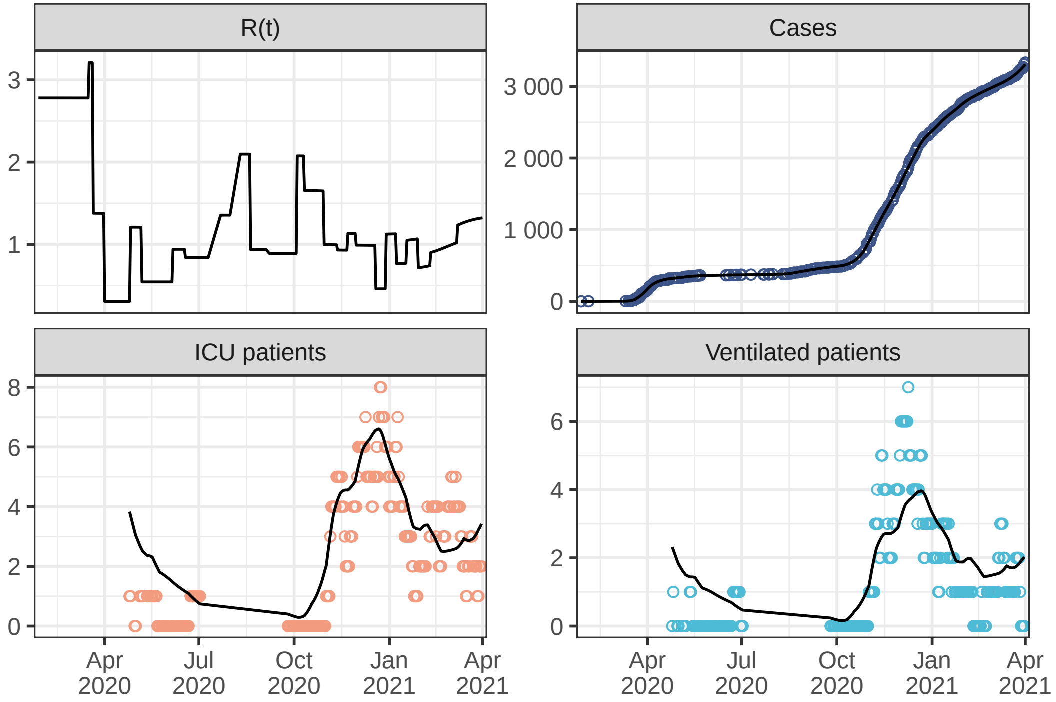

LK Landshut

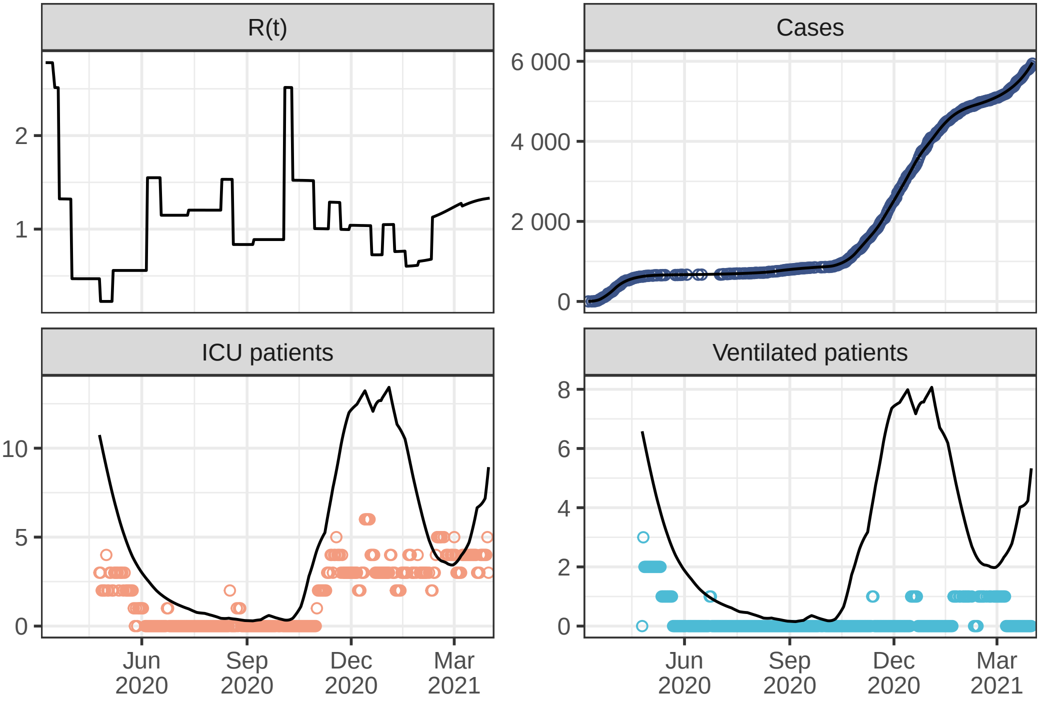

LK Leer

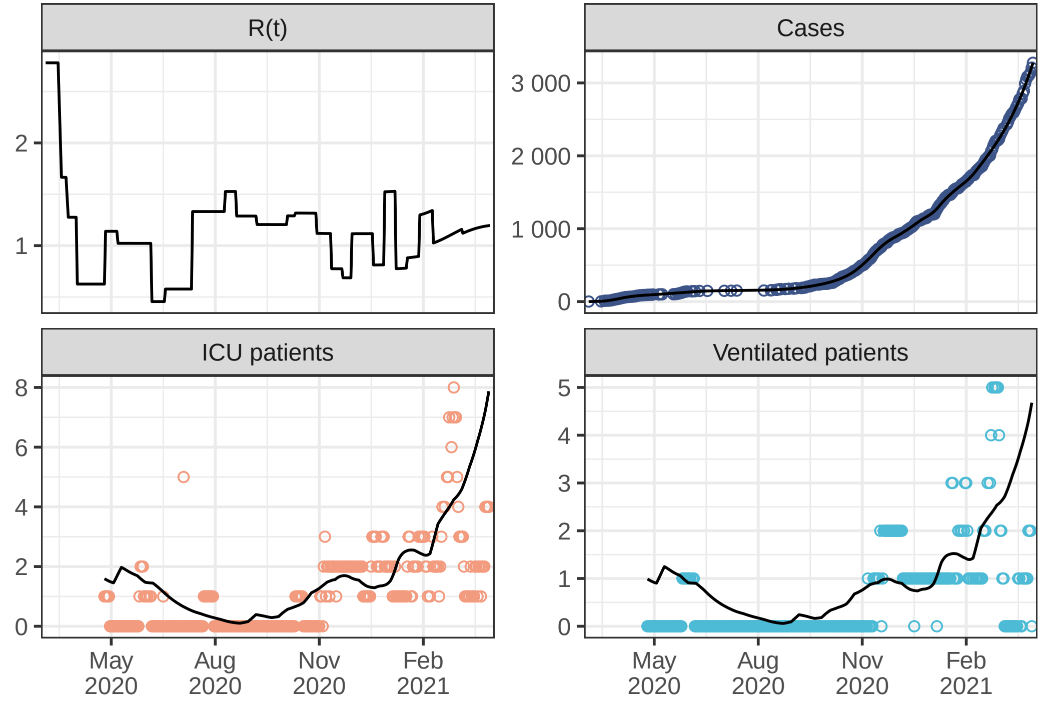

LK Leipzig

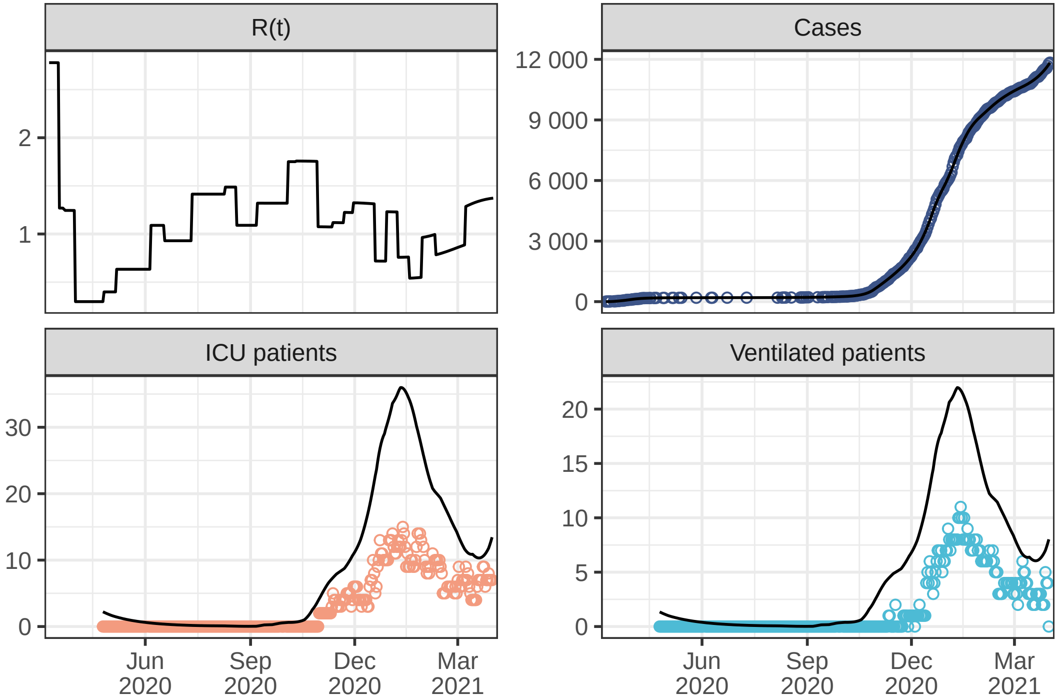

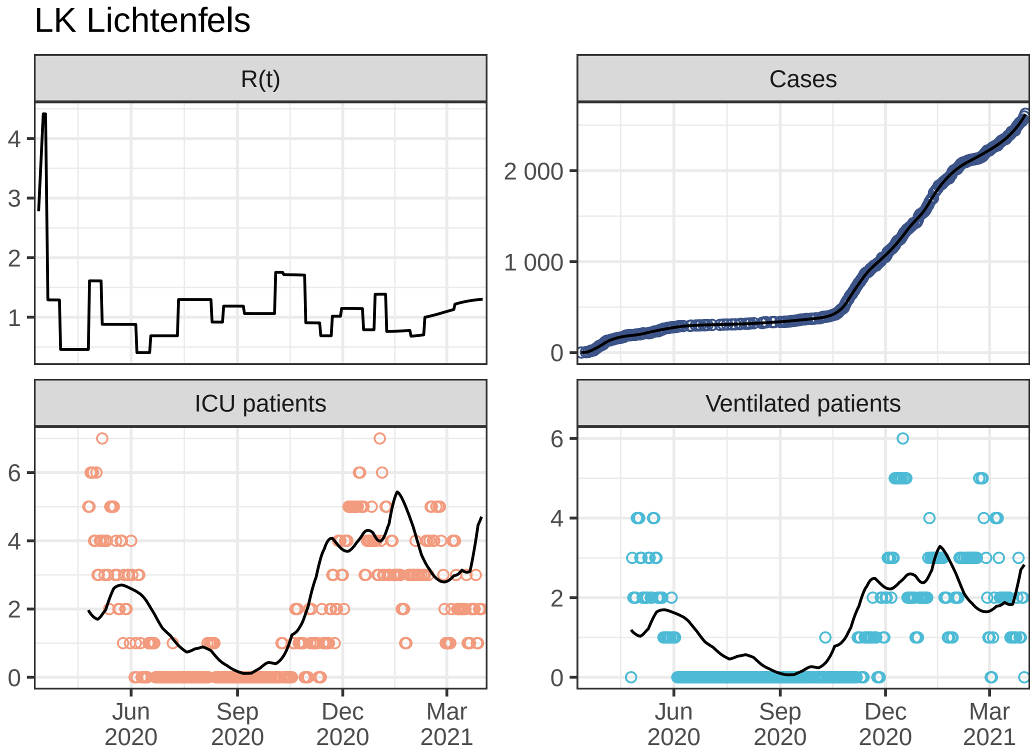

LK Lichtenfels
R(t)
Cases
ICU patients
Ventilated patients
4
3
2
1
2 000
1 000
0
6
4
2
0
Jun 2020
Sep 2020
Dec 2020
Mar 2021

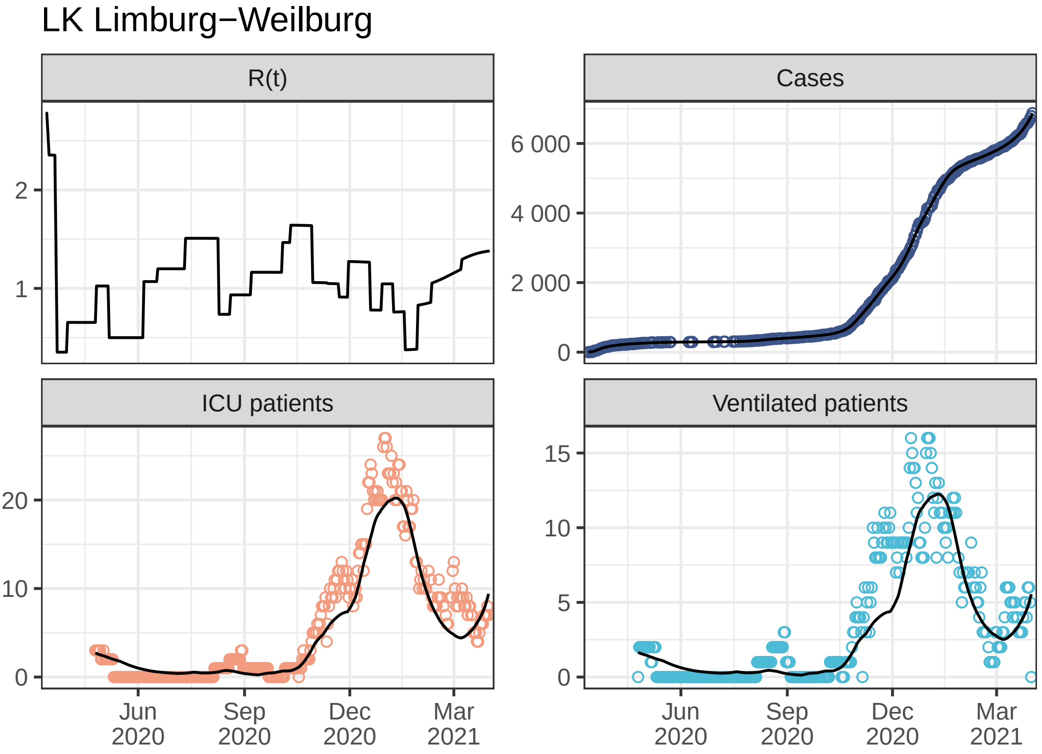

LK Limburg−Weilburg
R(t)
Cases
ICU patients
Ventilated patients
2
1
6 000
4 000
2 000
0
20
10
0
15
10
5
0
Jun 2020
Sep 2020
Dec 2020
Mar 2021

LK Lindau

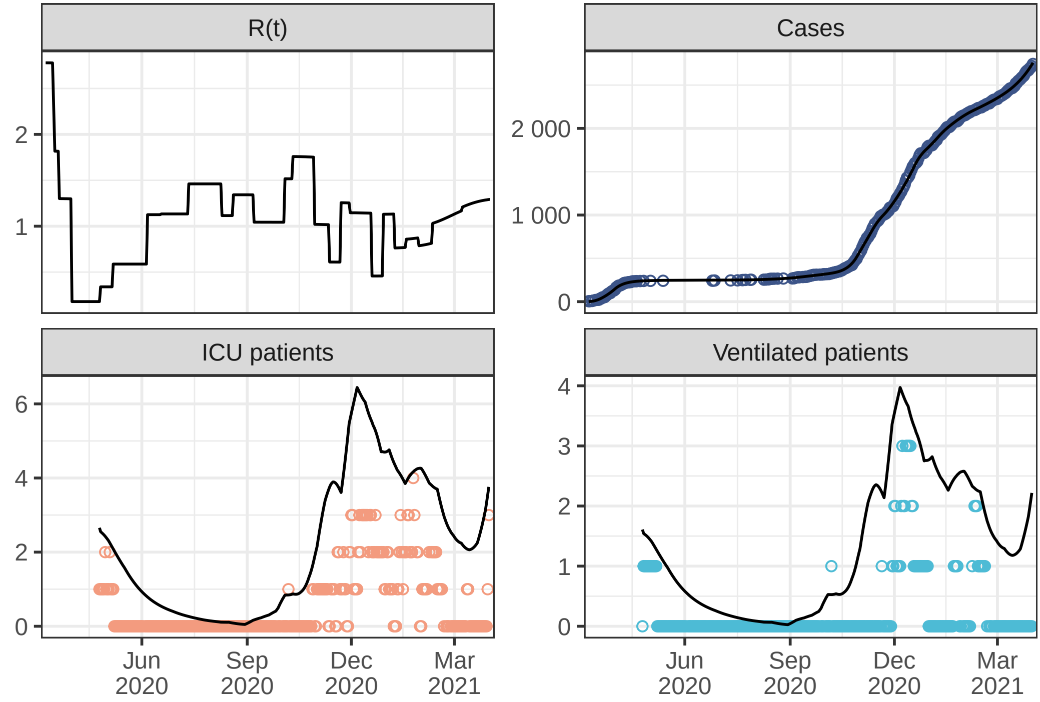

LK Lippe

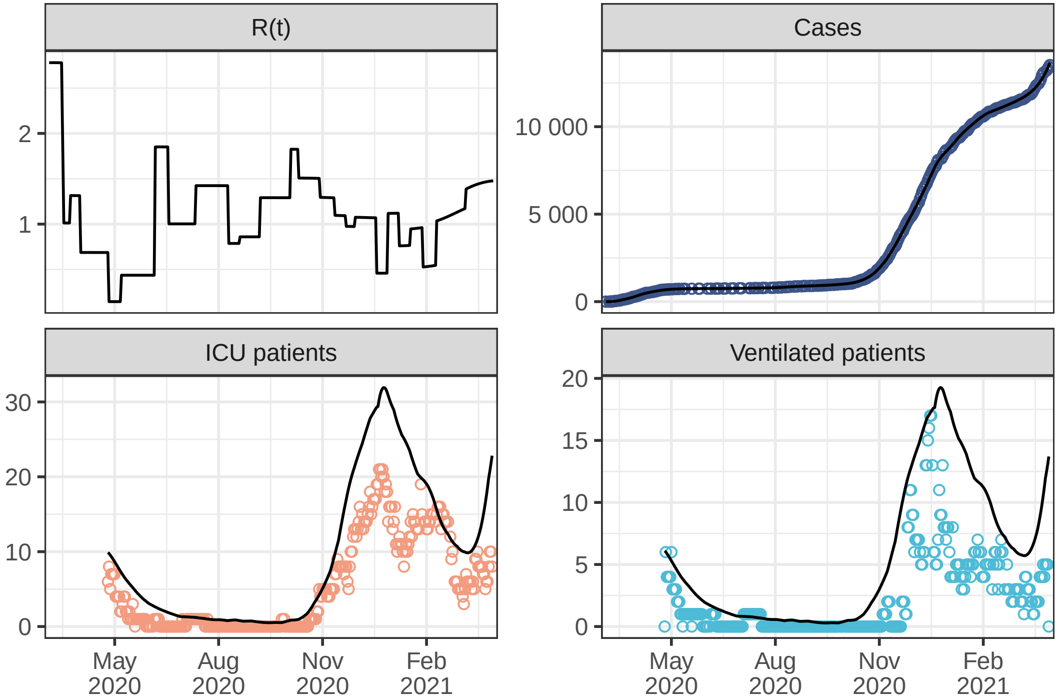

LK Lörrach

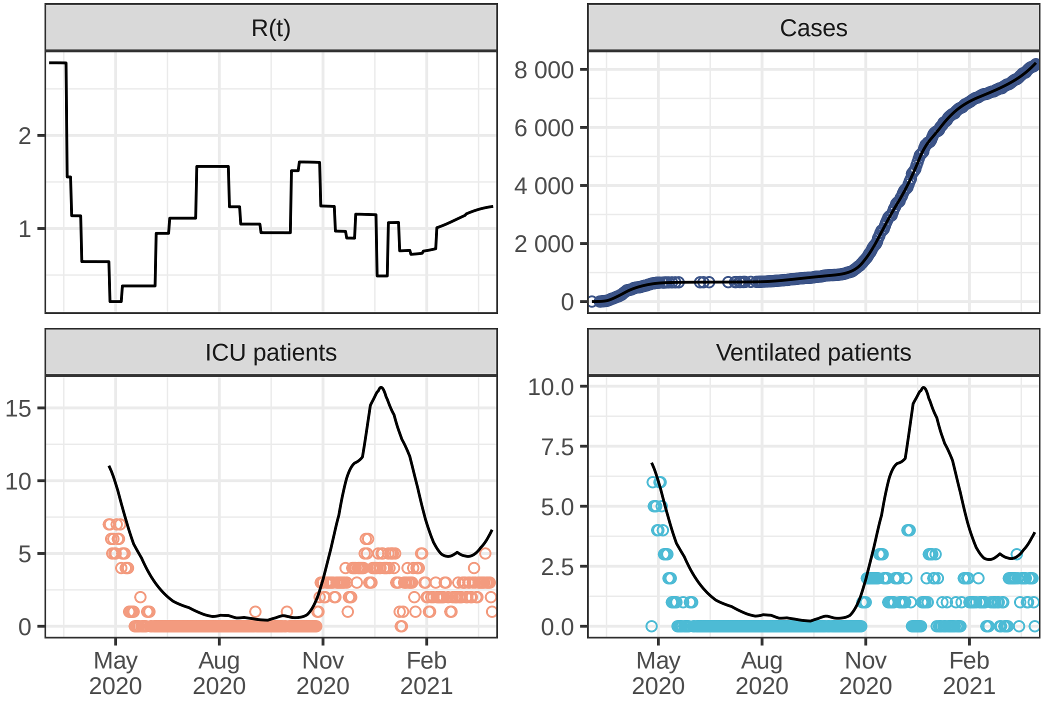

LK Lüchow−Dannenberg

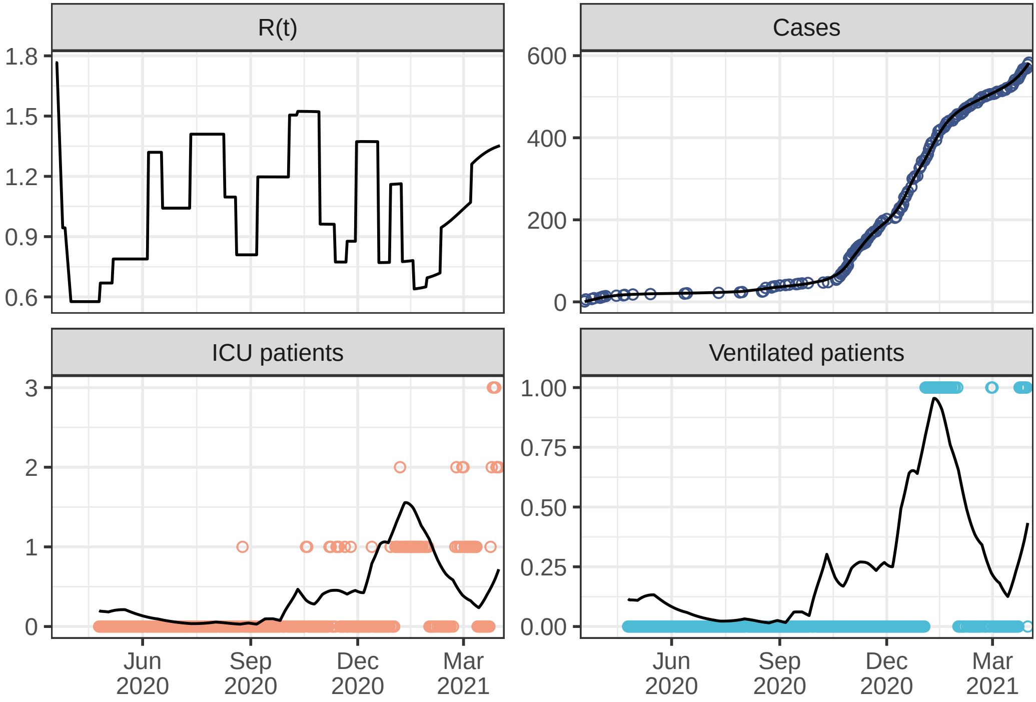

LK Ludwigsburg

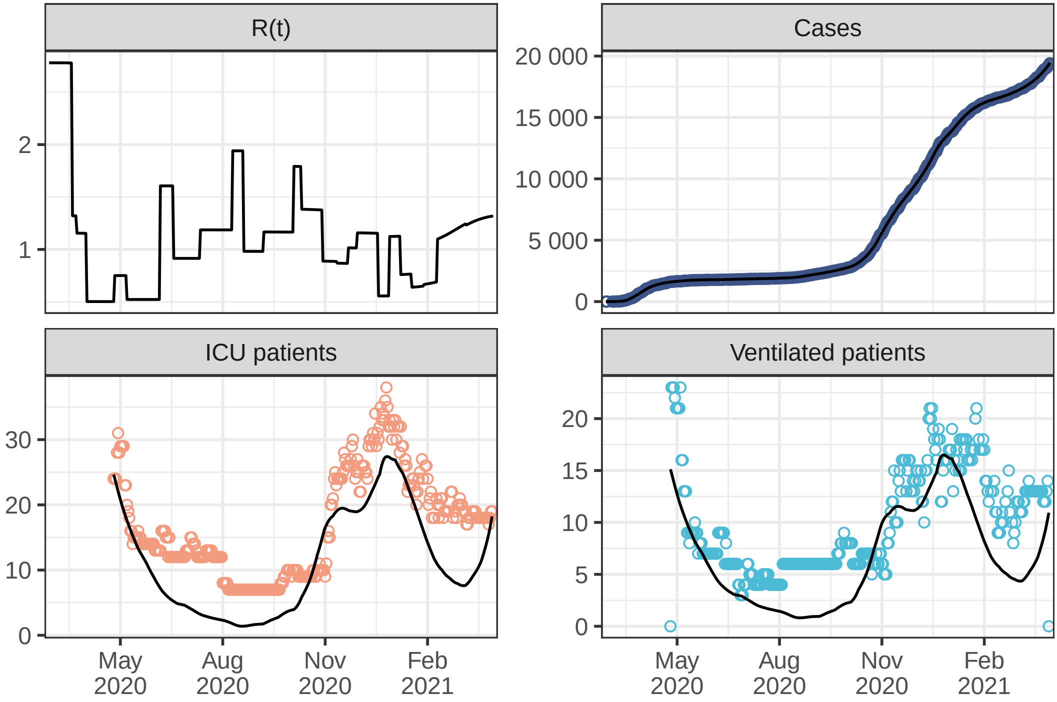

LK Ludwigslust−Parchim

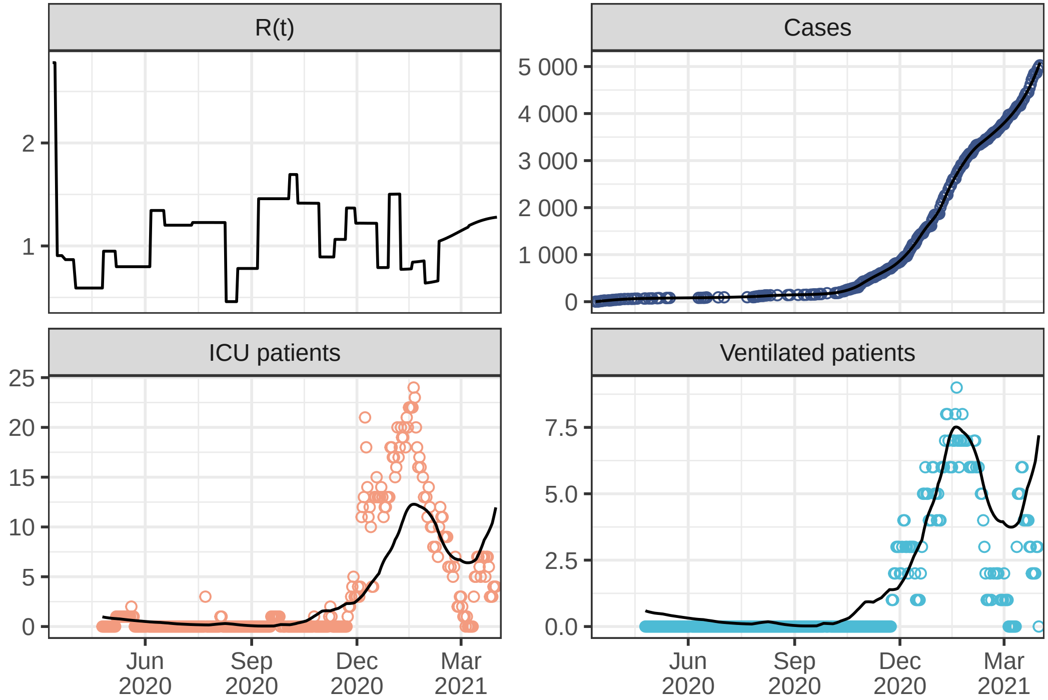

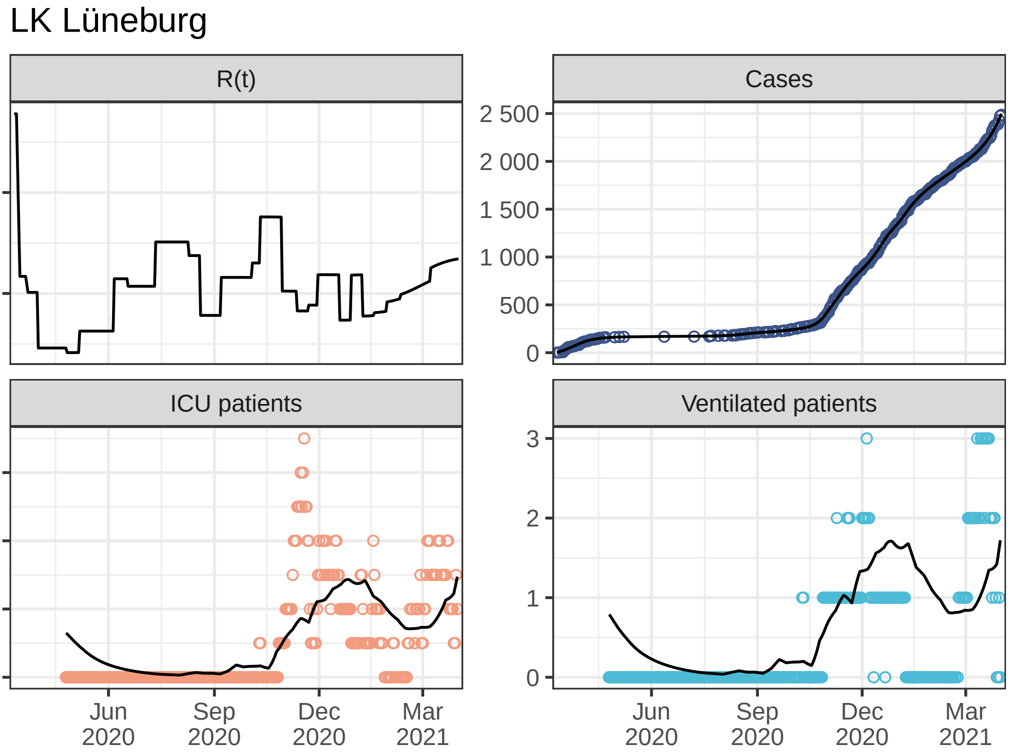
LK Lüneburg
R(t)
Cases
ICU patients
Ventilated patients
2
1
2 500
2 000
1 500
1 000
500
0
6
4
2
0
3
2
1
0
Jun 2020
Sep 2020
Dec 2020
Mar 2021

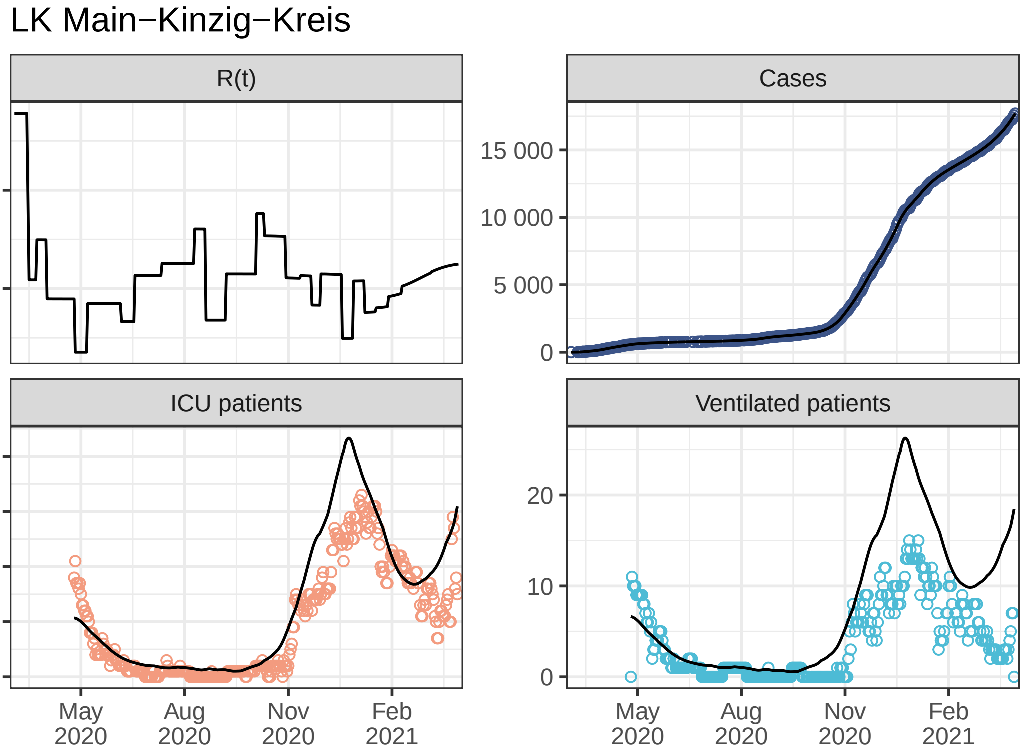
LK Main−Kinzig−Kreis
R(t)
Cases
ICU patients
Ventilated patients
2
1
15 000
10 000
5 000
0
40
30
20
10
0
20
10
0
May 2020
Aug 2020
Nov 2020
Feb 2021

LK Main−Spessart

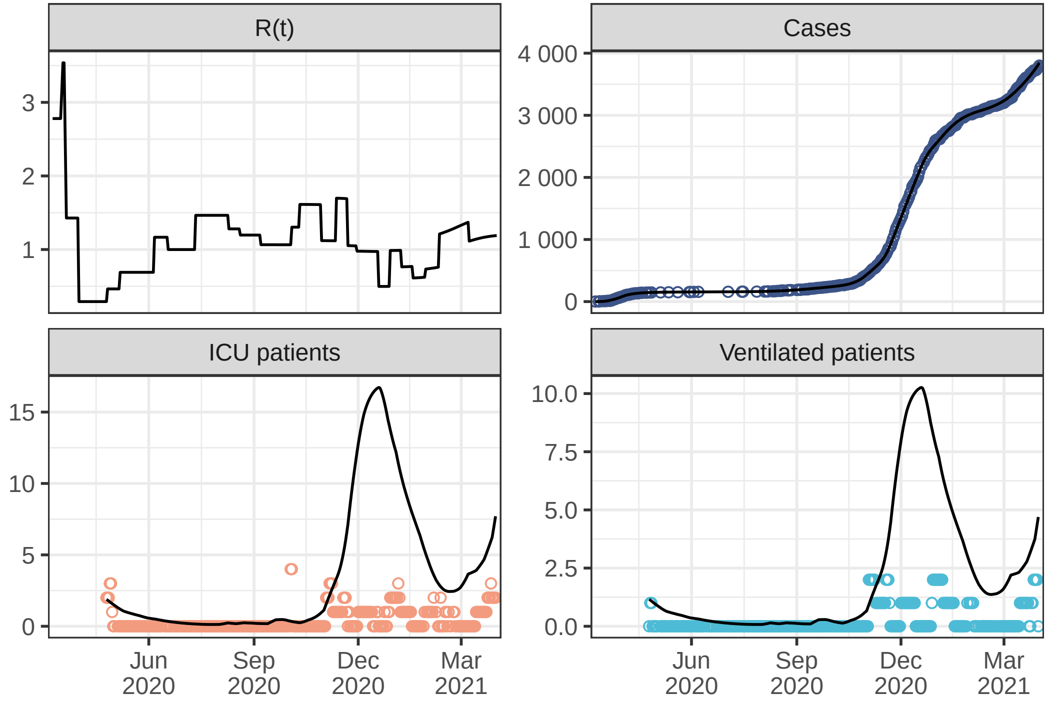

LK Main−Tauber−Kreis

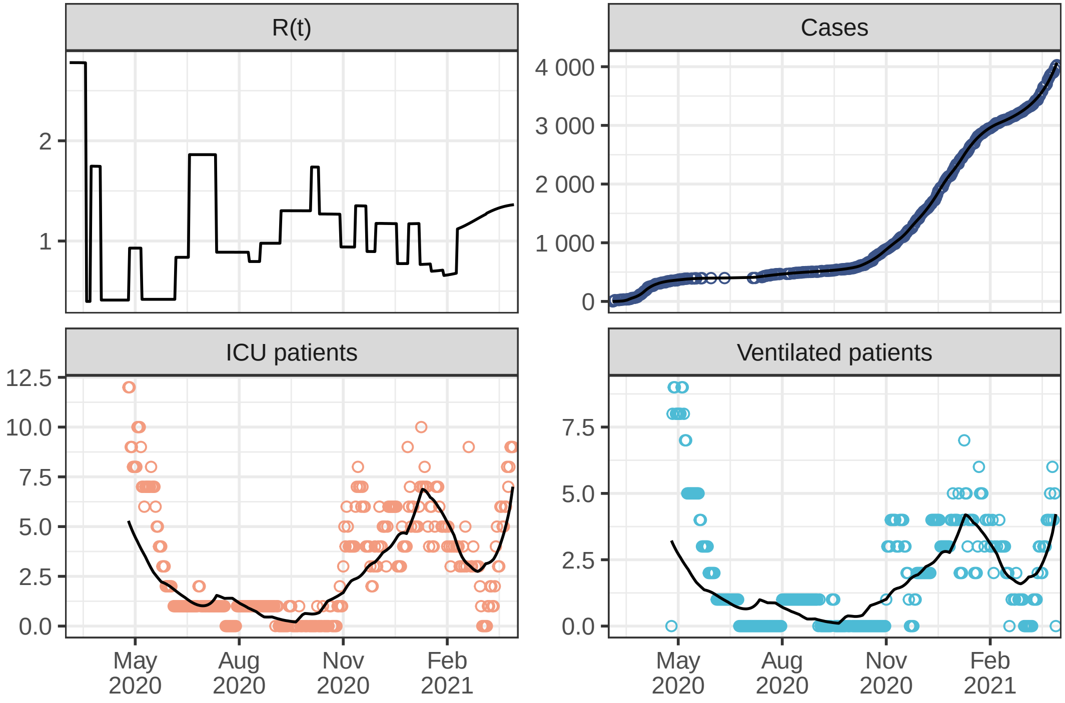

LK Main−Taunus−Kreis

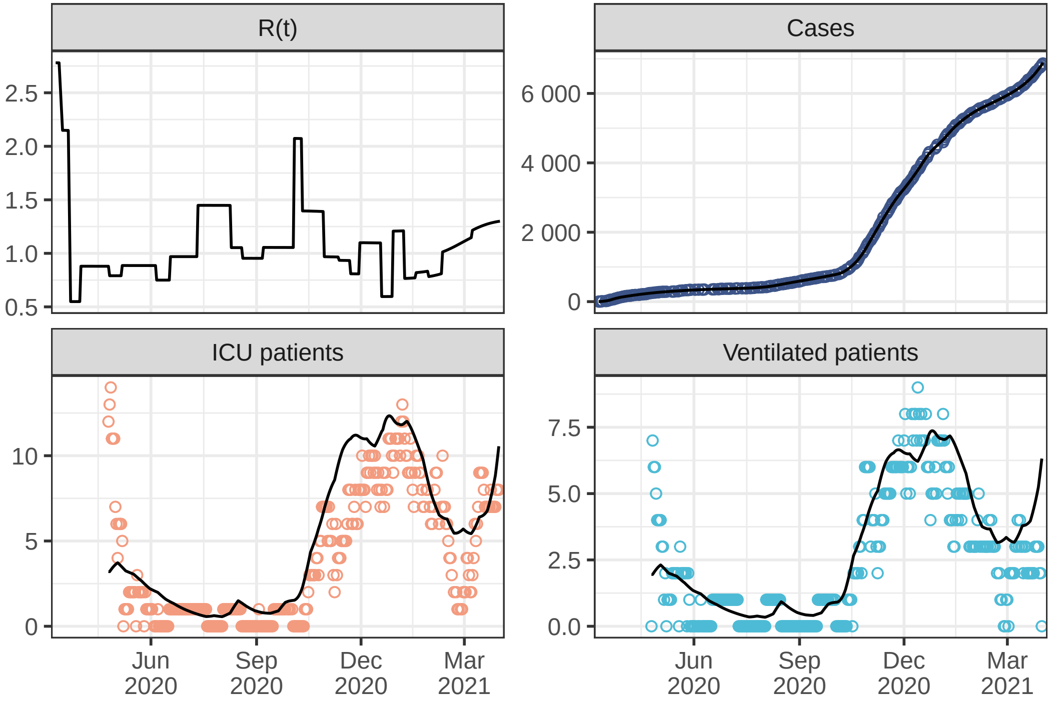

LK Mainz−Bingen

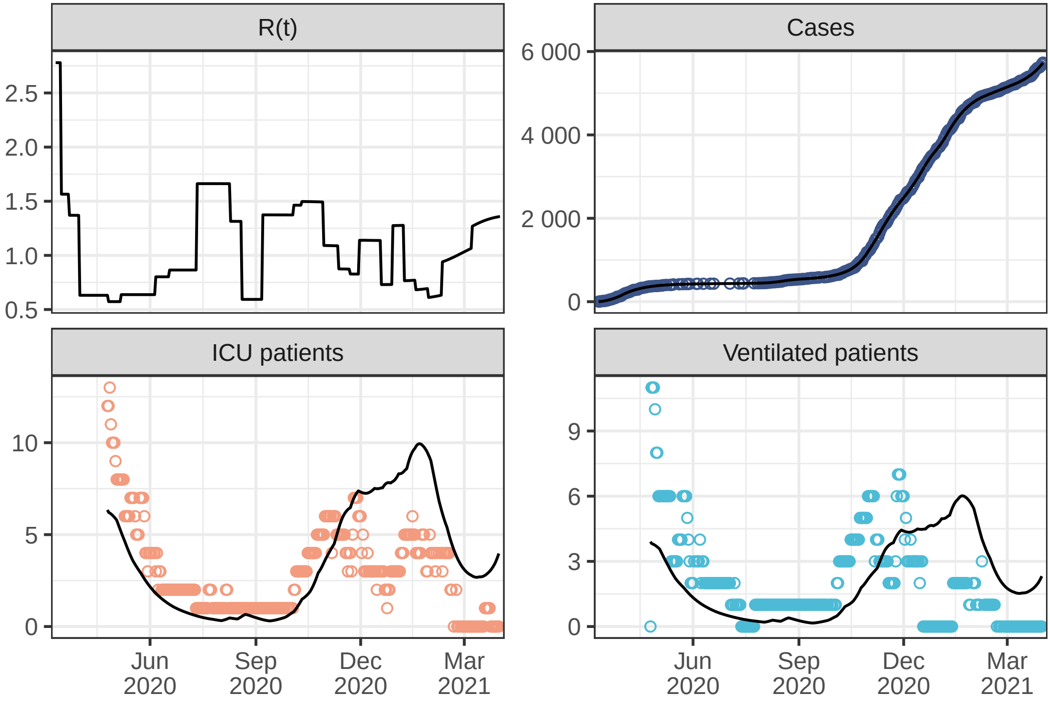

LK Mansfeld−Südharz

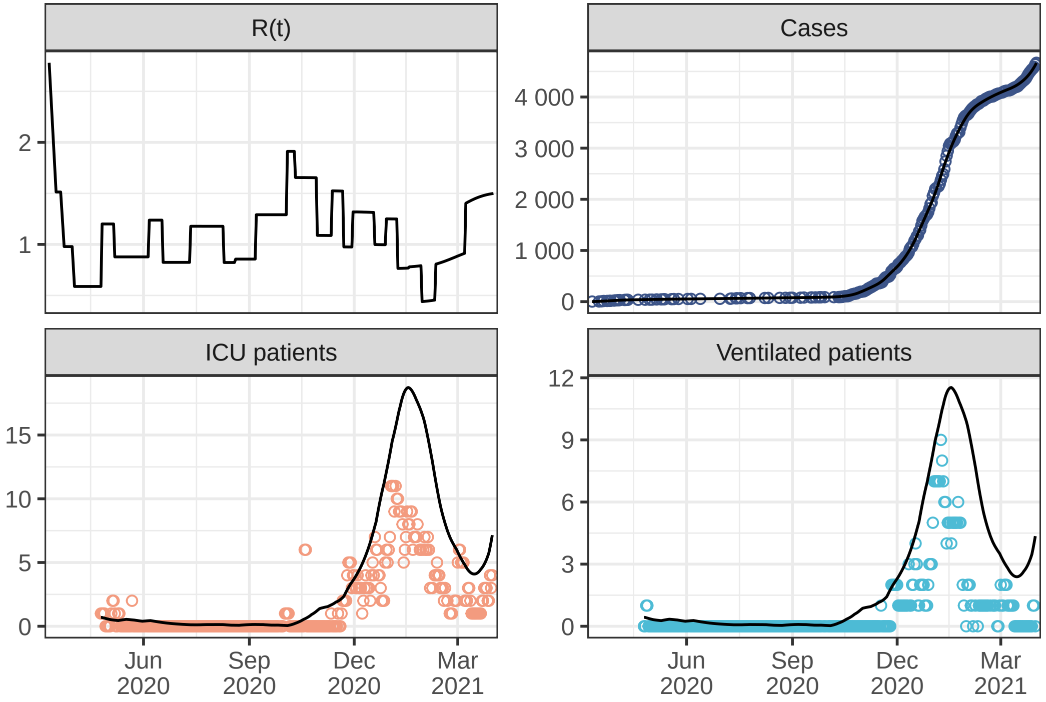

LK Marburg−Biedenkopf

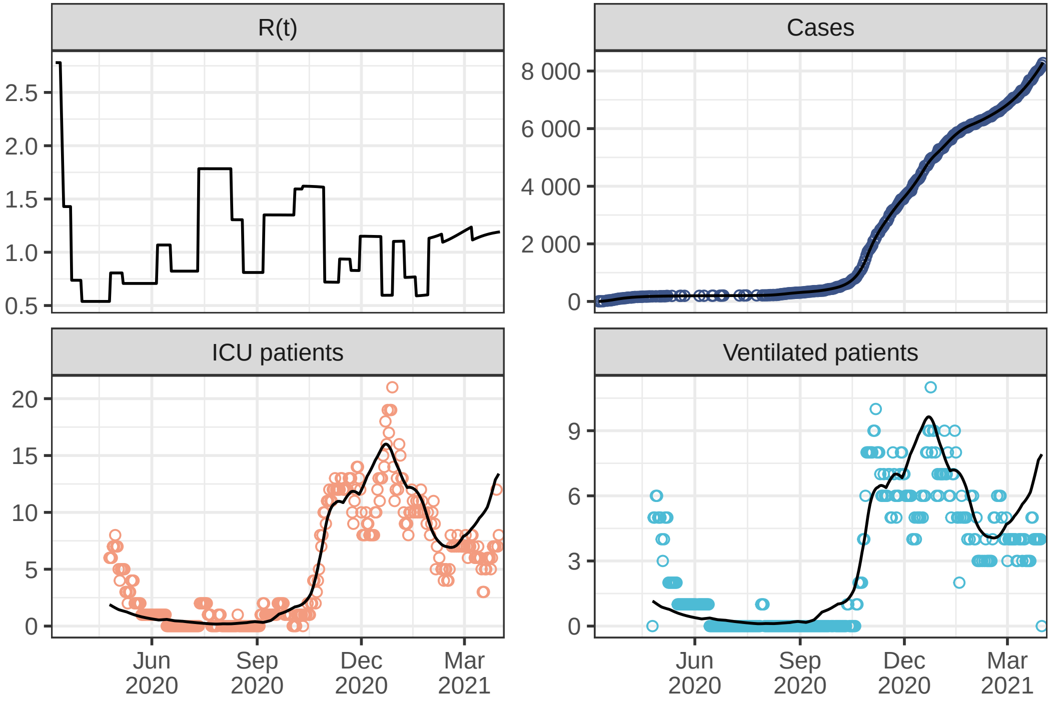

LK Märkisch−Oderland

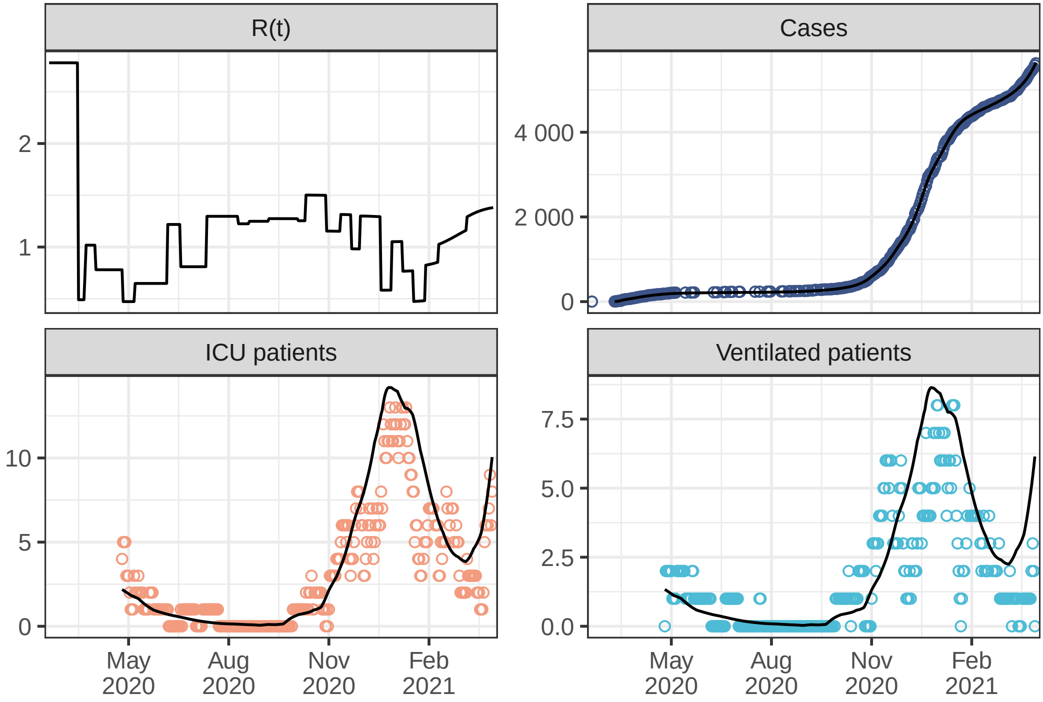

LK Märkischer Kreis

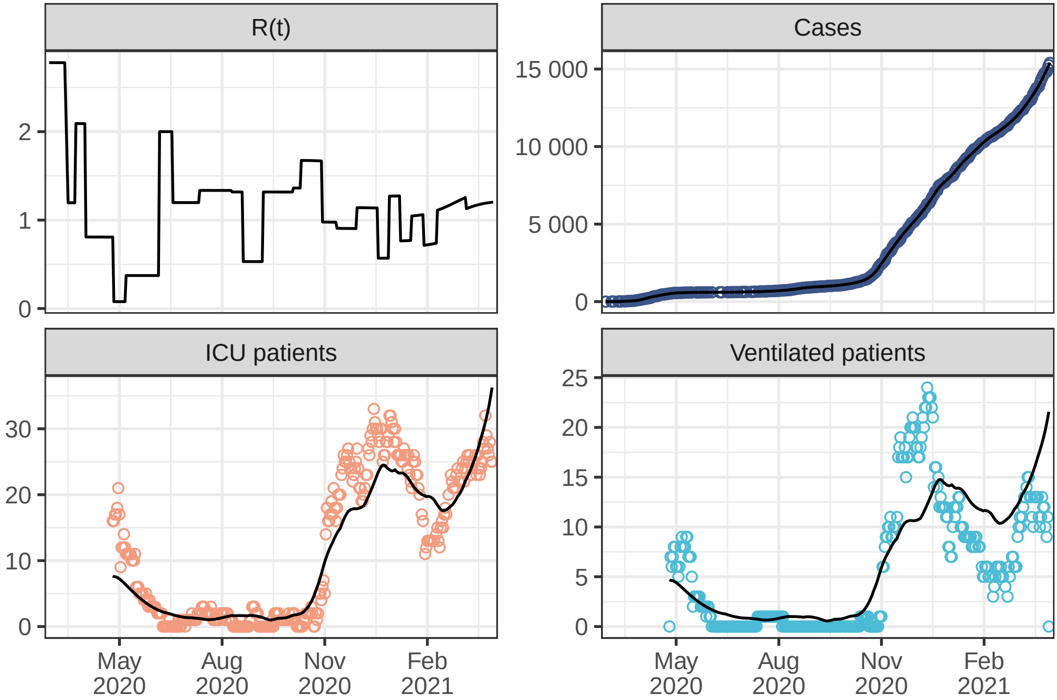

LK Mayen−Koblenz

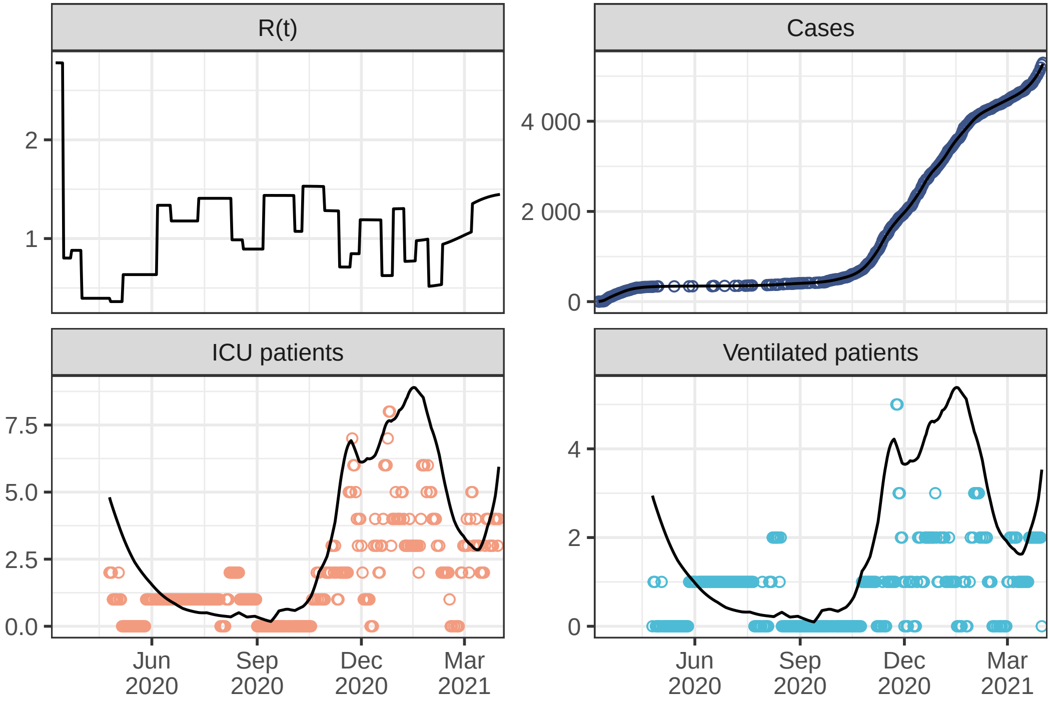

LK Mecklenburgische Seenplatte

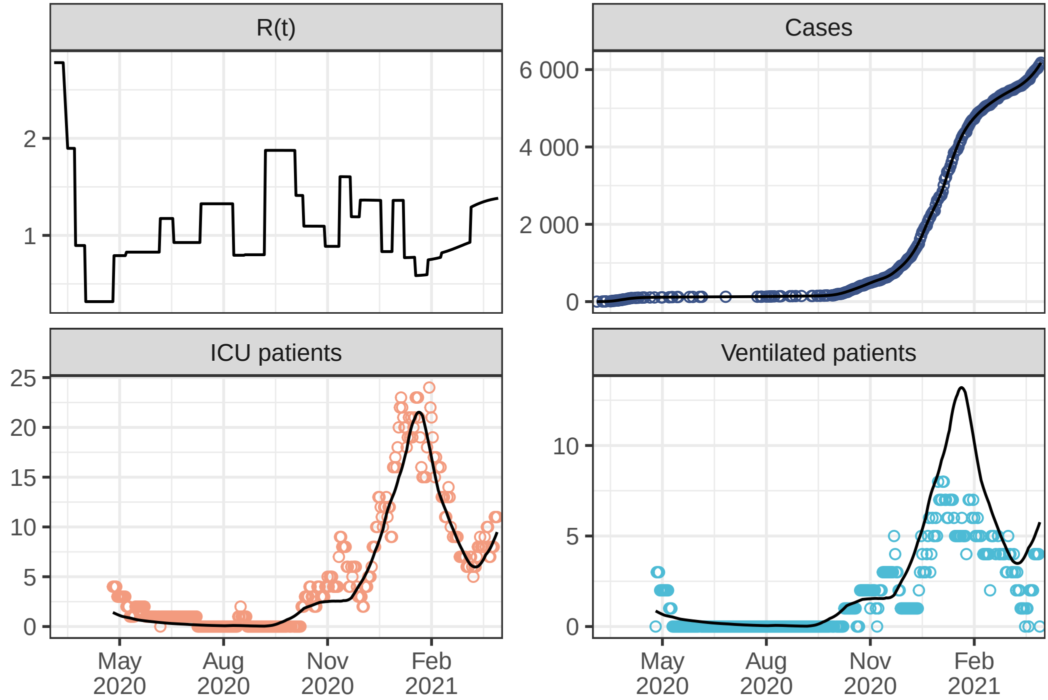

LK Meißen

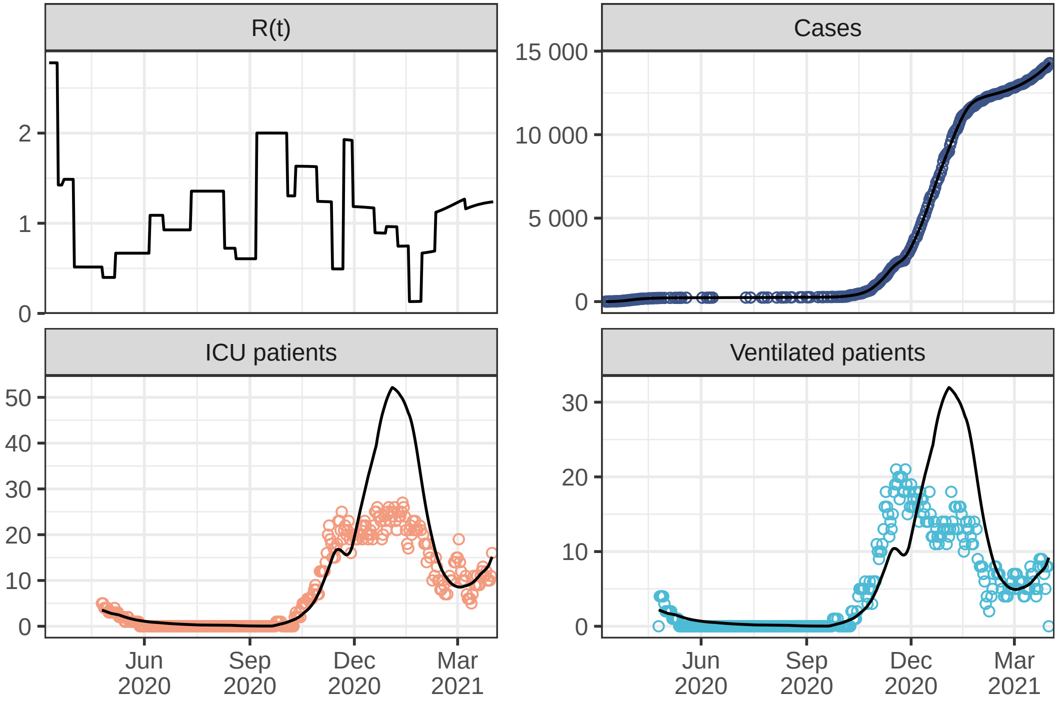

LK Merzig−Wadern

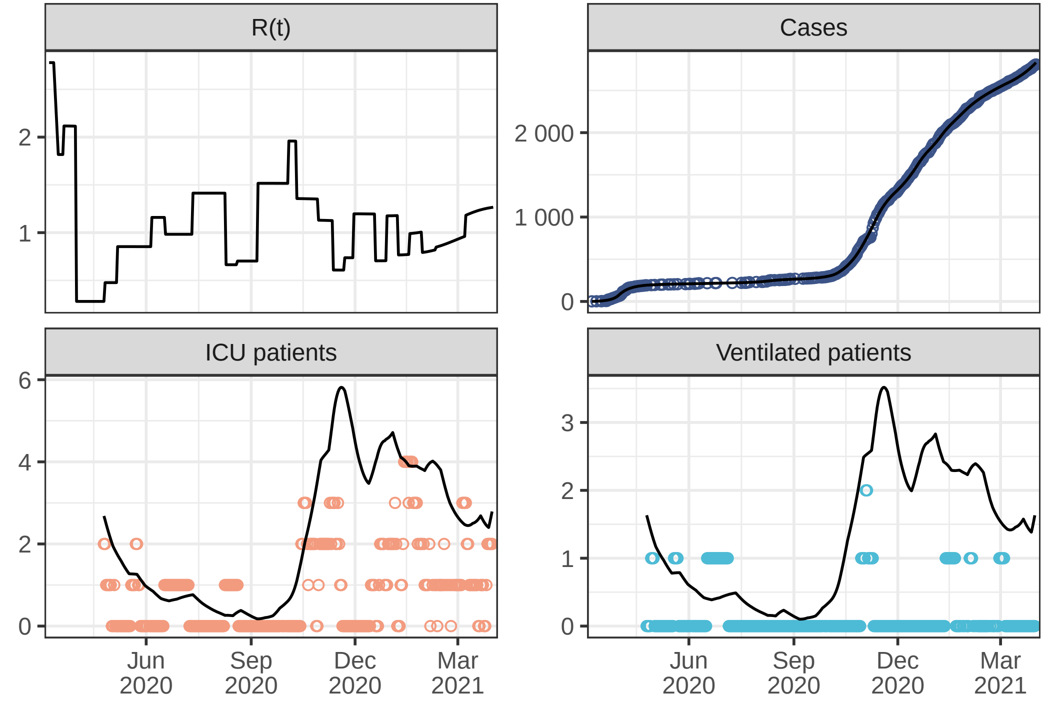

LK Mettmann

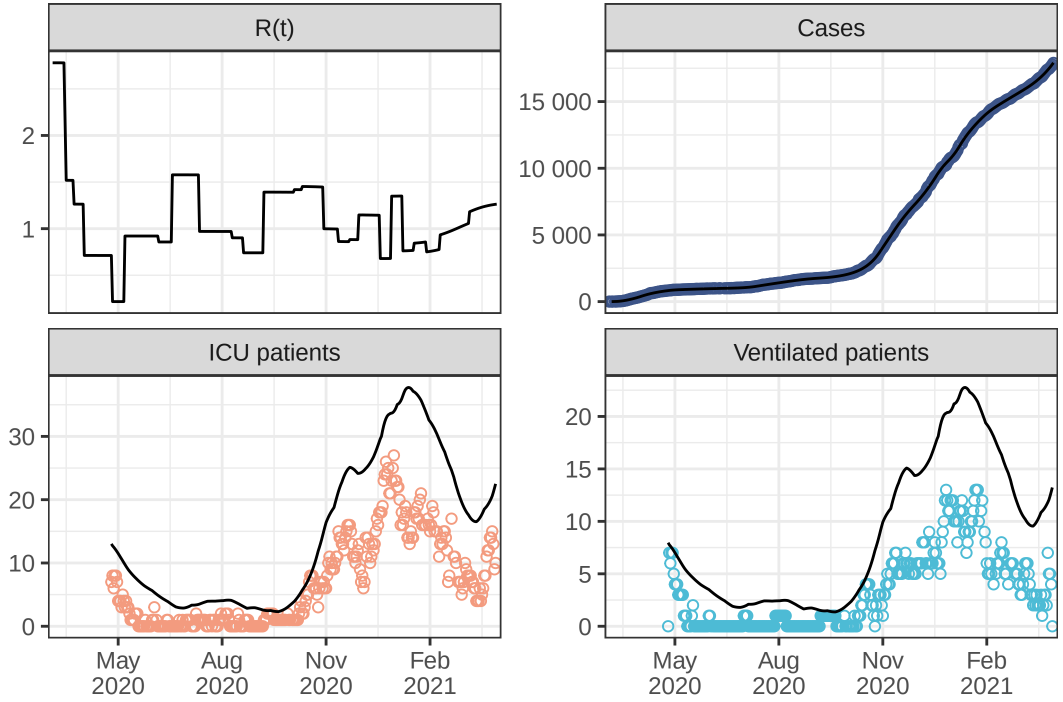

LK Miesbach

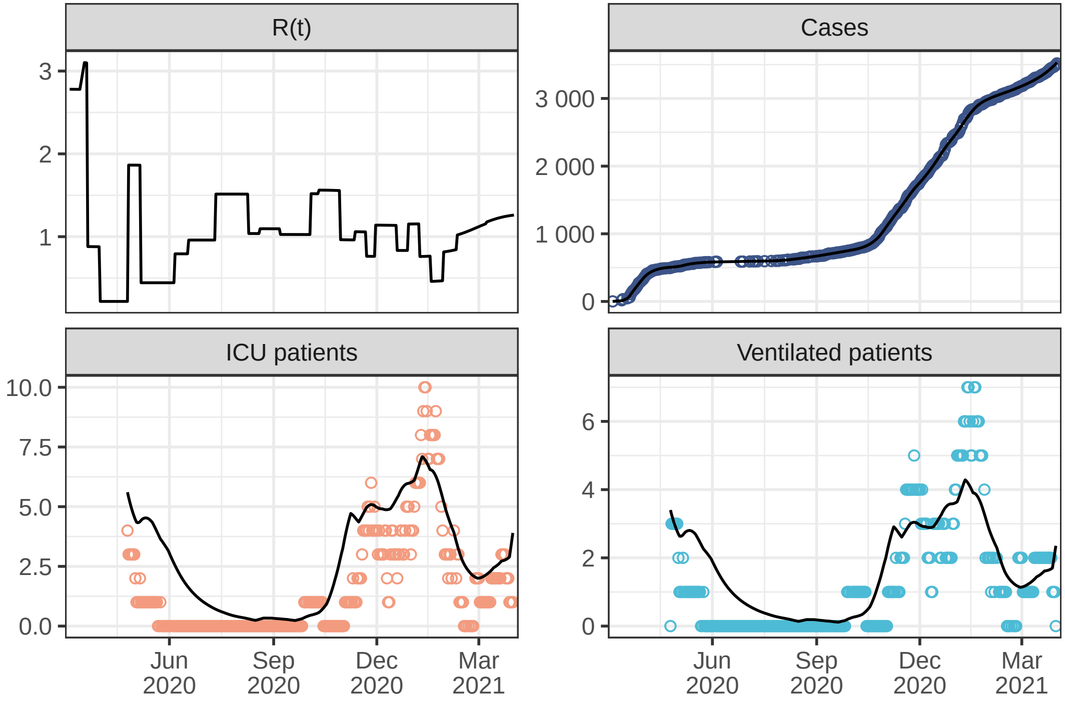

LK Miltenberg

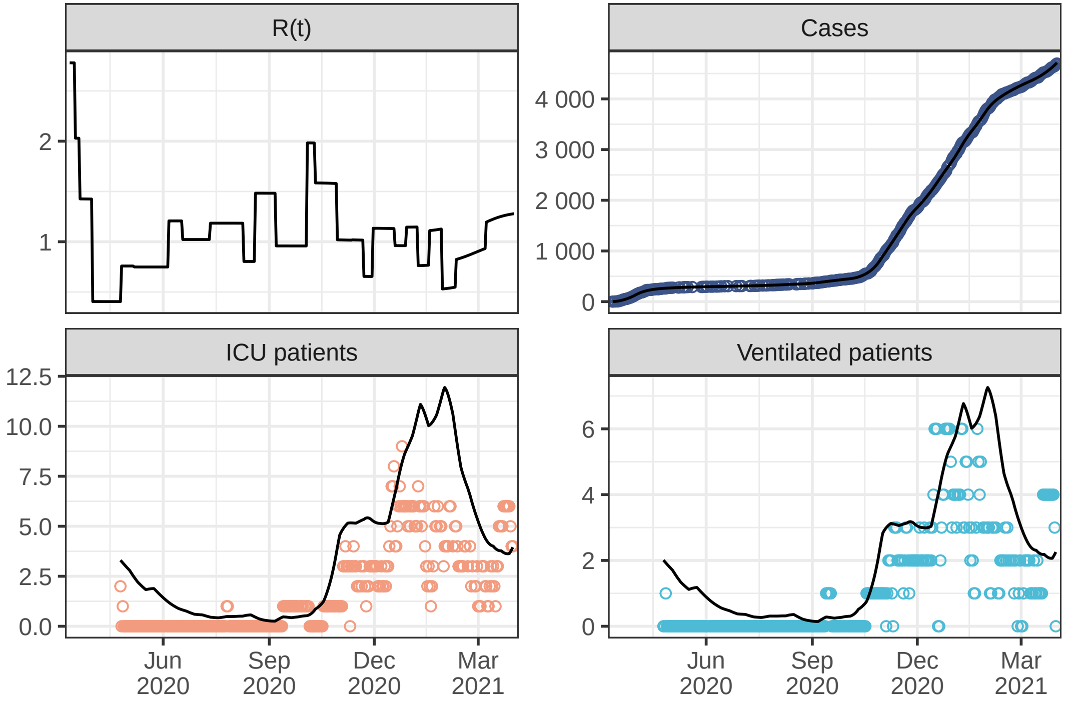

LK Minden−Lübbecke

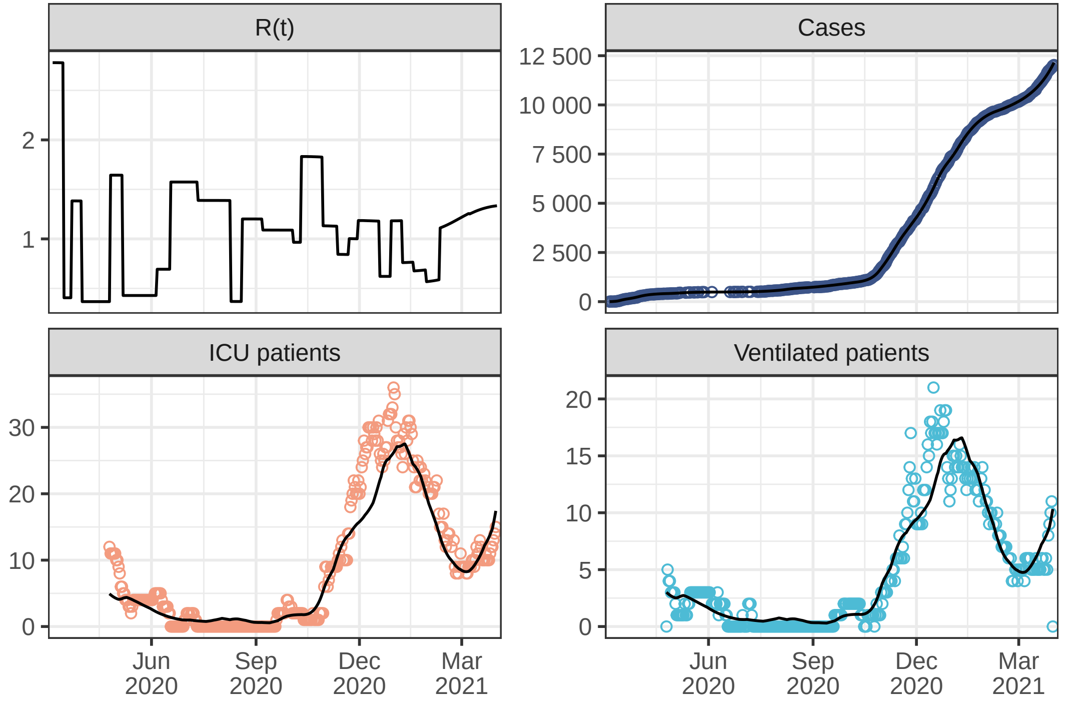

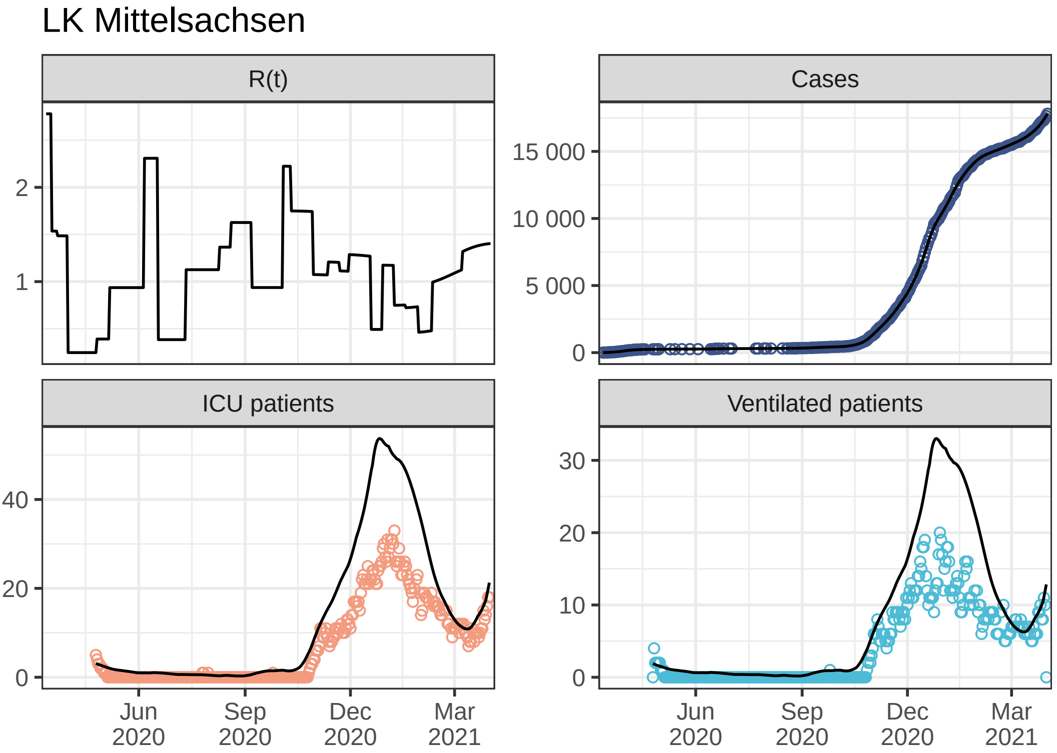
LK Mittelsachsen
R(t)
Cases
ICU patients
Ventilated patients
2
1
15 000
10 000
5 000
0
40
20
0
30
20
10
0
Jun 2020
Sep 2020
Dec 2020
Mar 2021

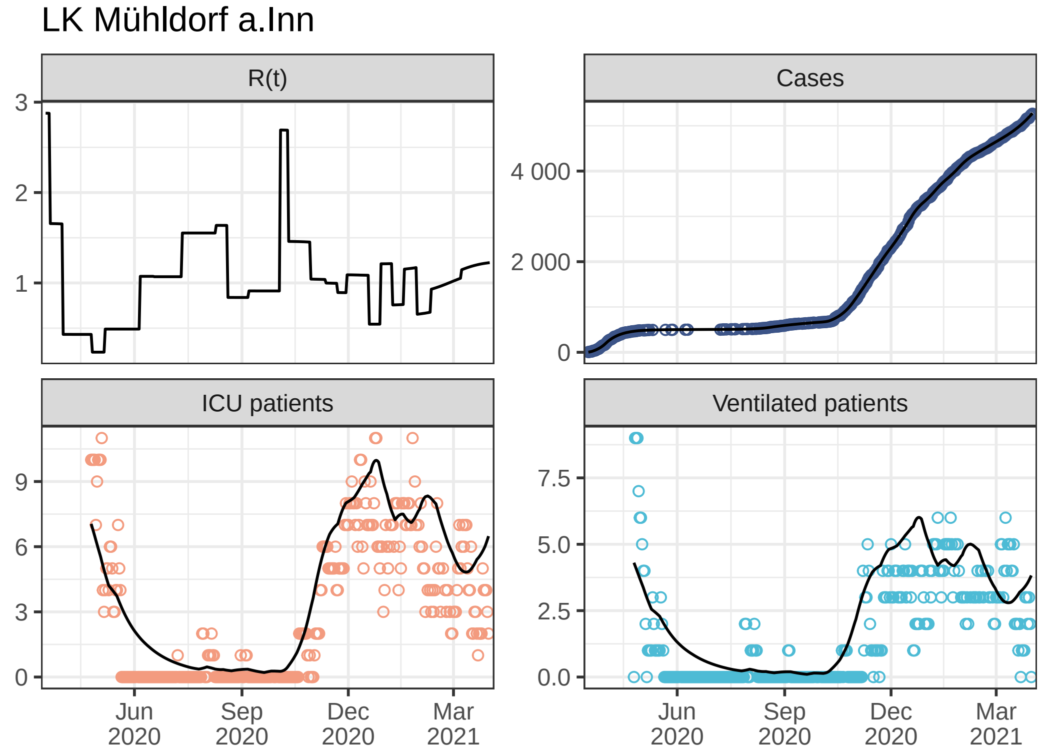
LK Mühldorf a.Inn
R(t)
Cases
ICU patients
Ventilated patients
3
2
1
4 000
2 000
0
9
6
3
0
7.5
5.0
2.5
0.0
Jun 2020
Sep 2020
Dec 2020
Mar 2021

LK München

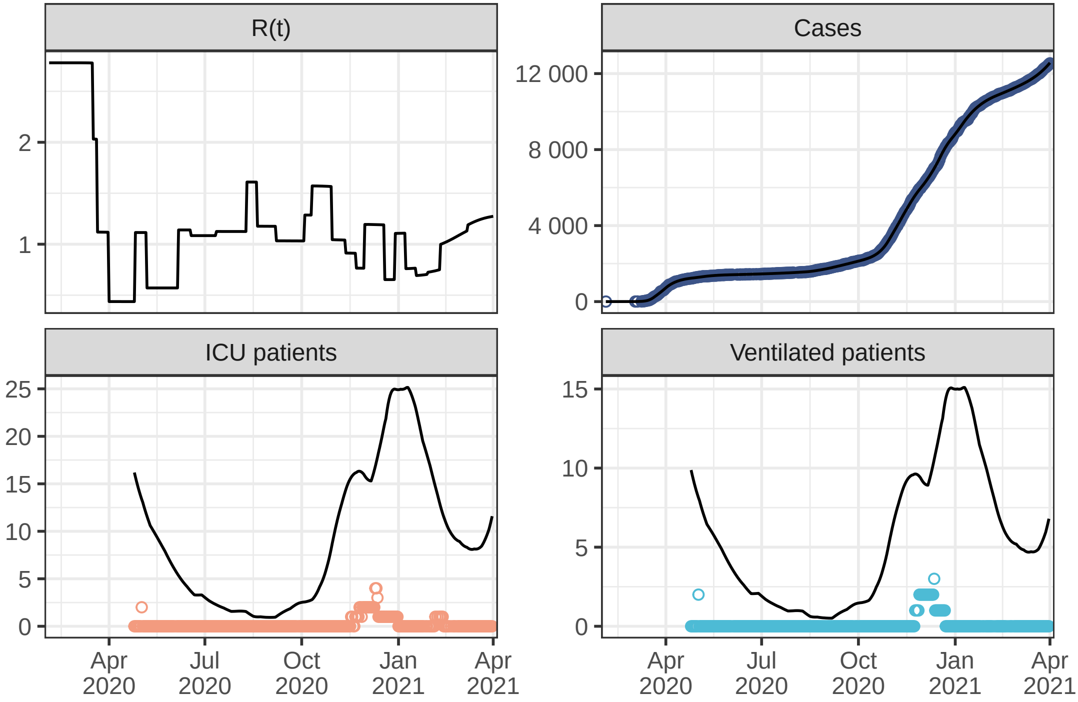

LK Neckar−Odenwald−Kreis

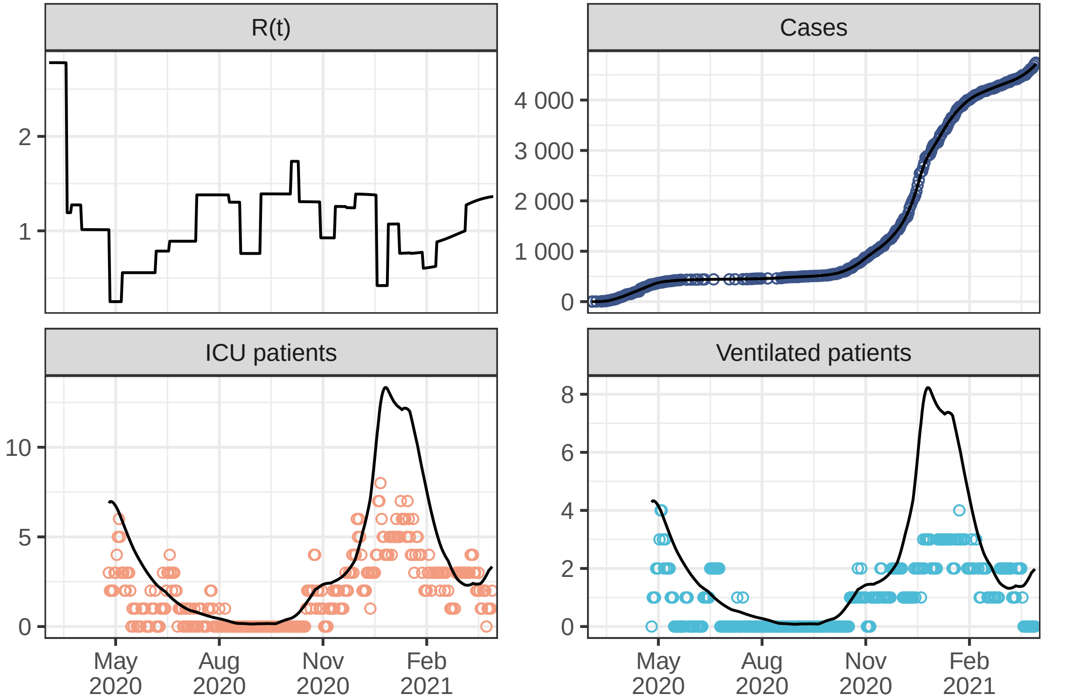

LK Neu−Ulm

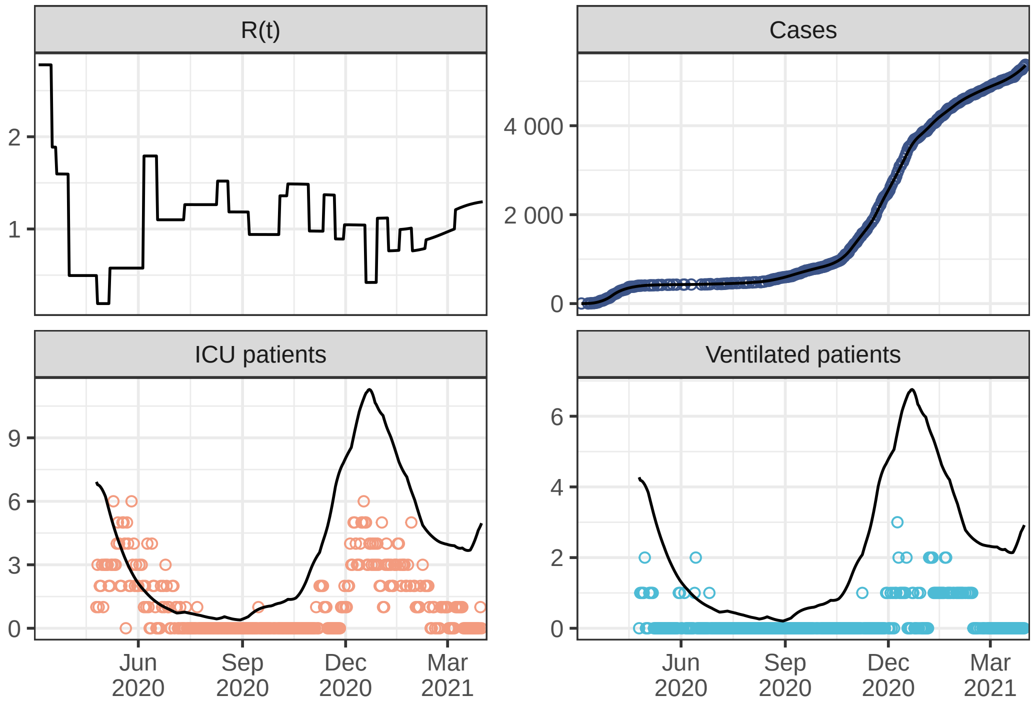

LK Neuburg−Schrobenhausen

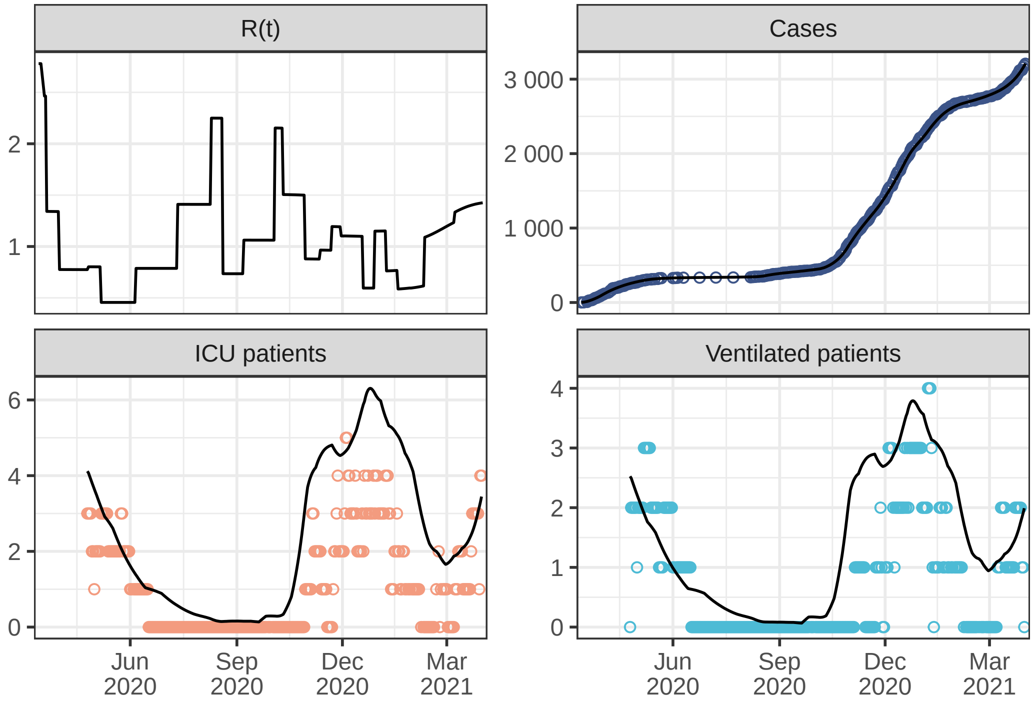

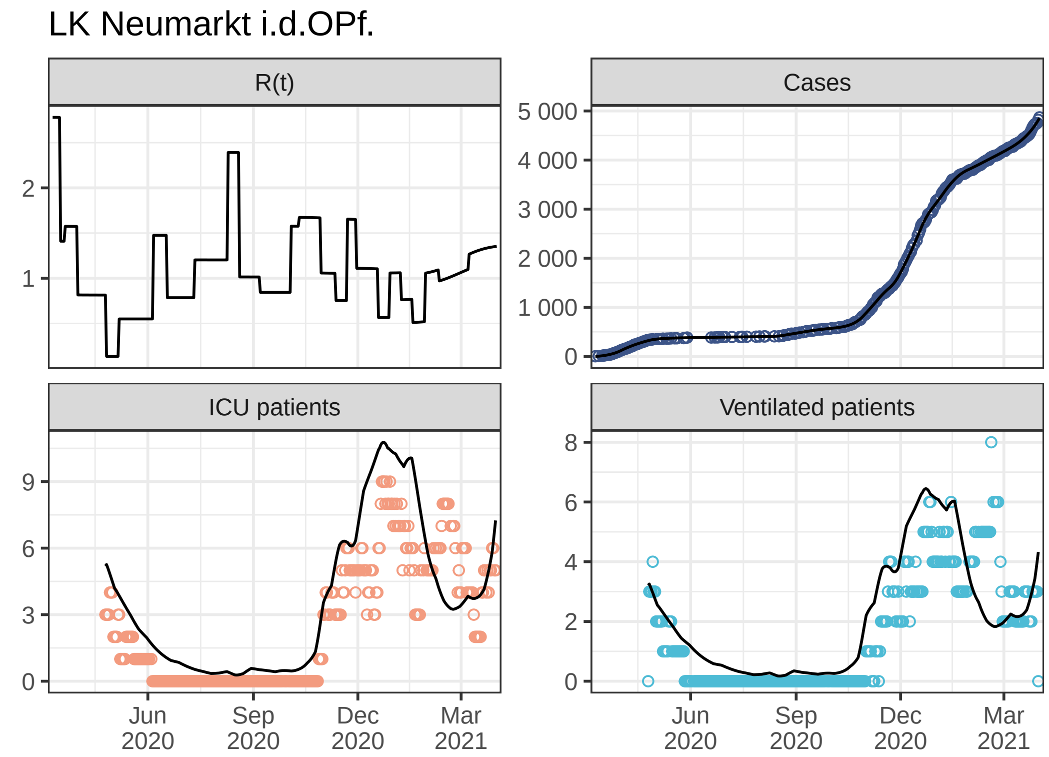
LK Neumarkt i.d.OPf.
R(t)
Cases
ICU patients
Ventilated patients
Jun 2020
Sep 2020
Dec 2020
Mar 2021

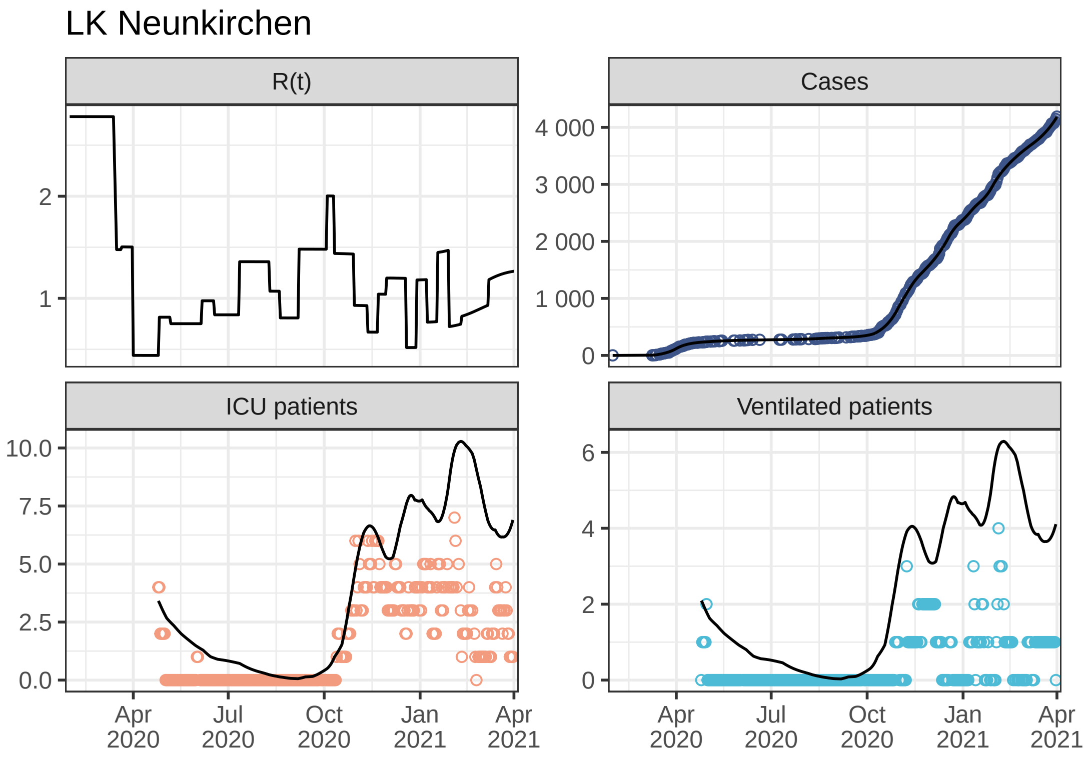
LK Neunkirchen
R(t)
Cases
ICU patients
Ventilated patients
Apr 2020
Jul 2020
Oct 2020
Jan 2021
Apr 2021

LK Neustadt a.d.Aisch−Bad Windsheim

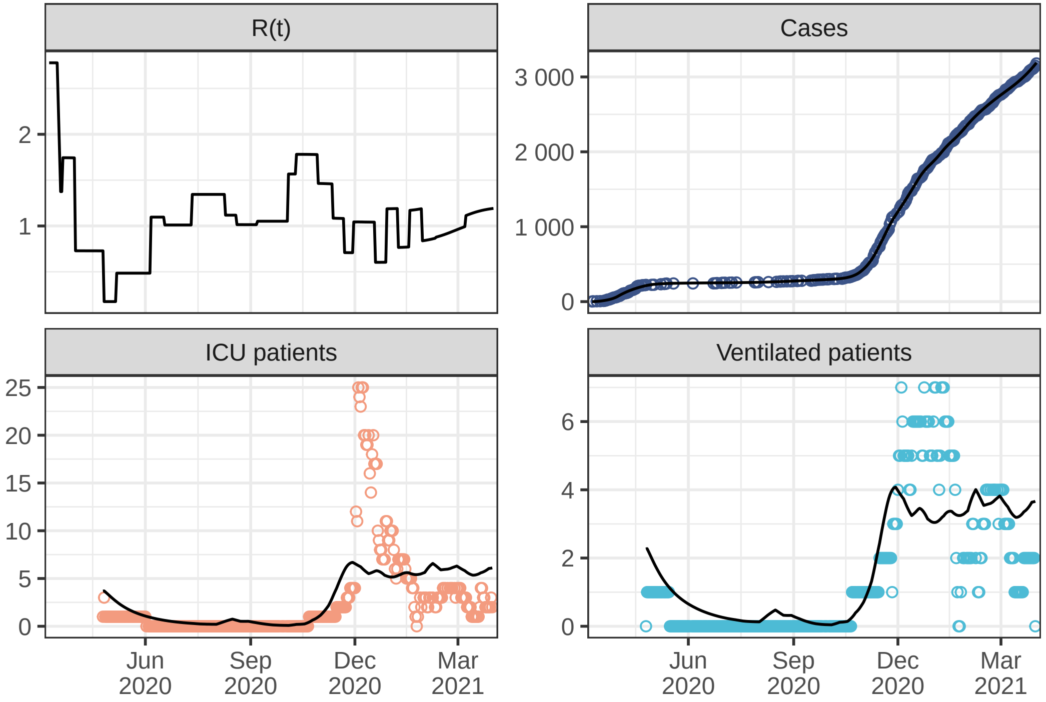

LK Neustadt a.d.Waldnaab

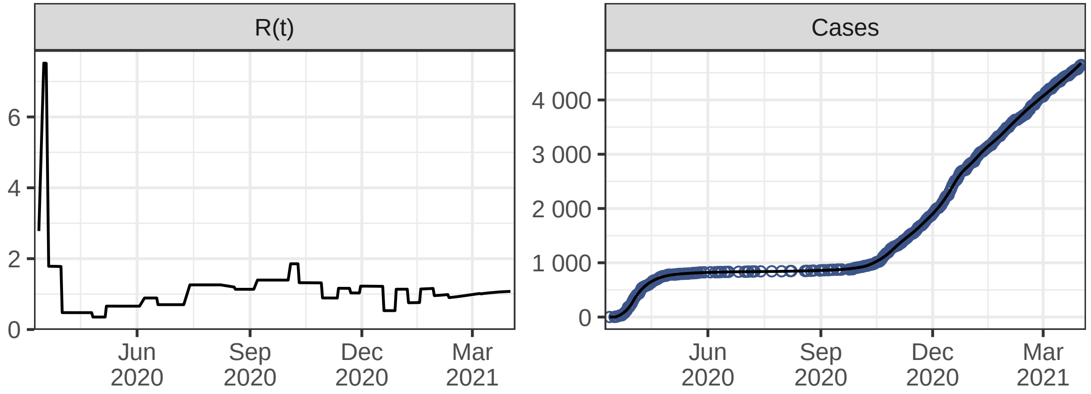

LK Neuwied

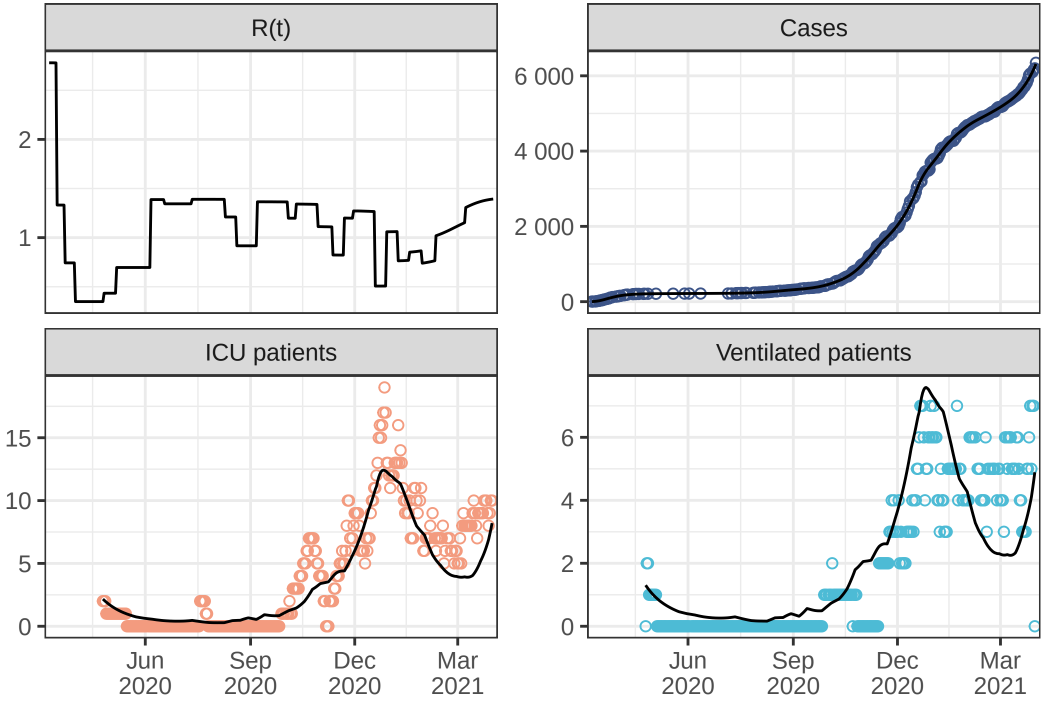

LK Nienburg (Weser)

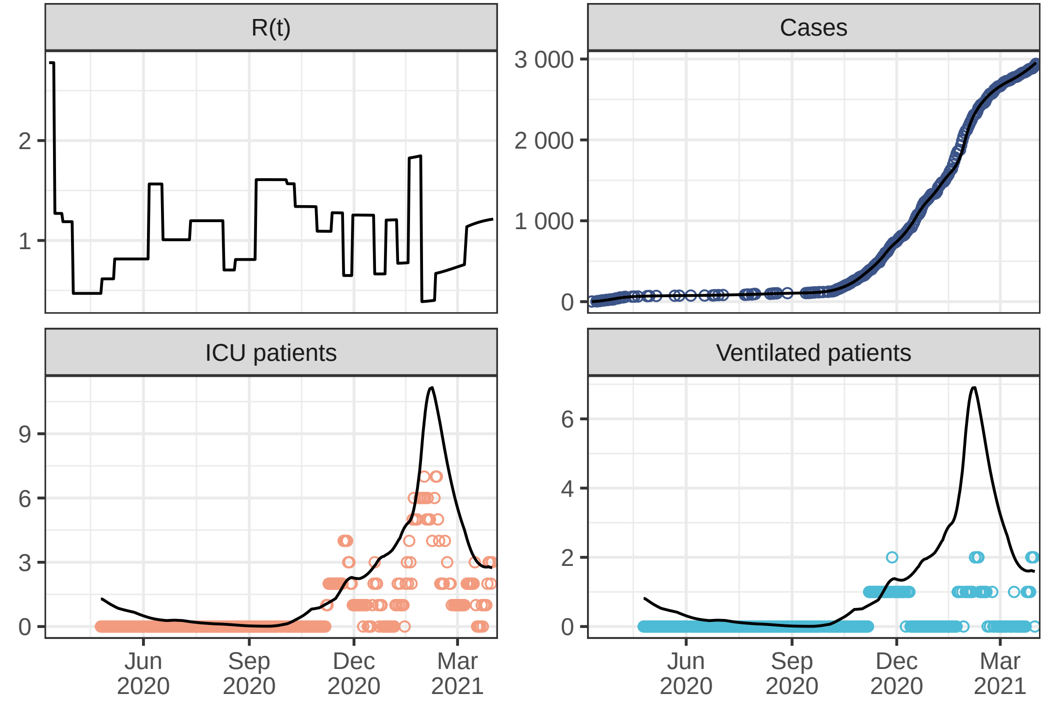

LK Nordfriesland

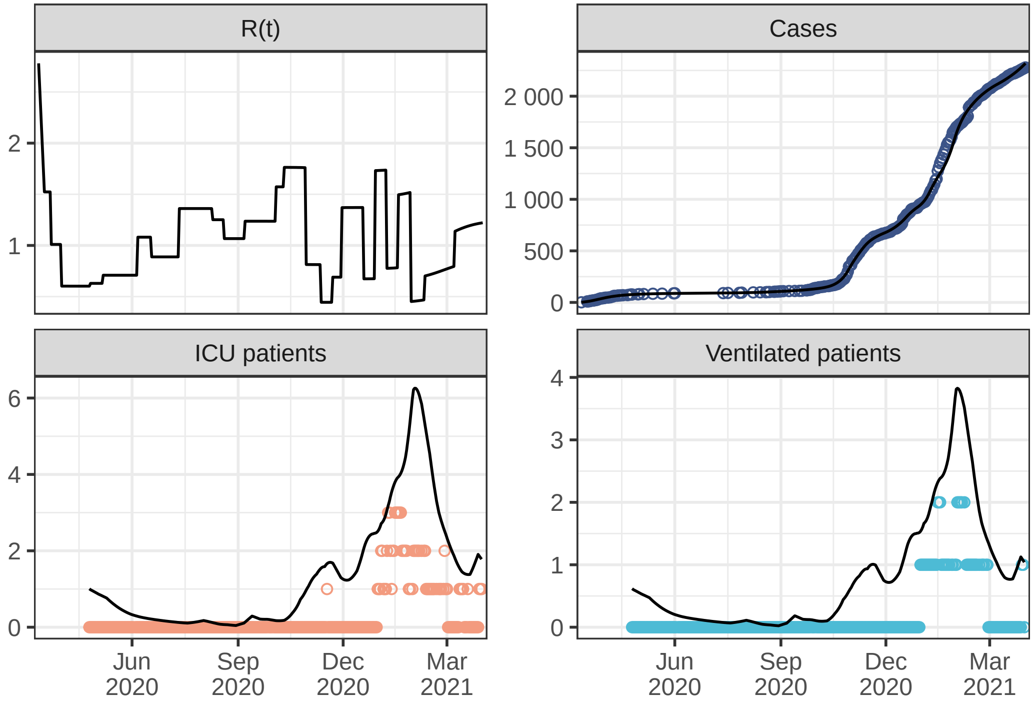

LK Nordhausen

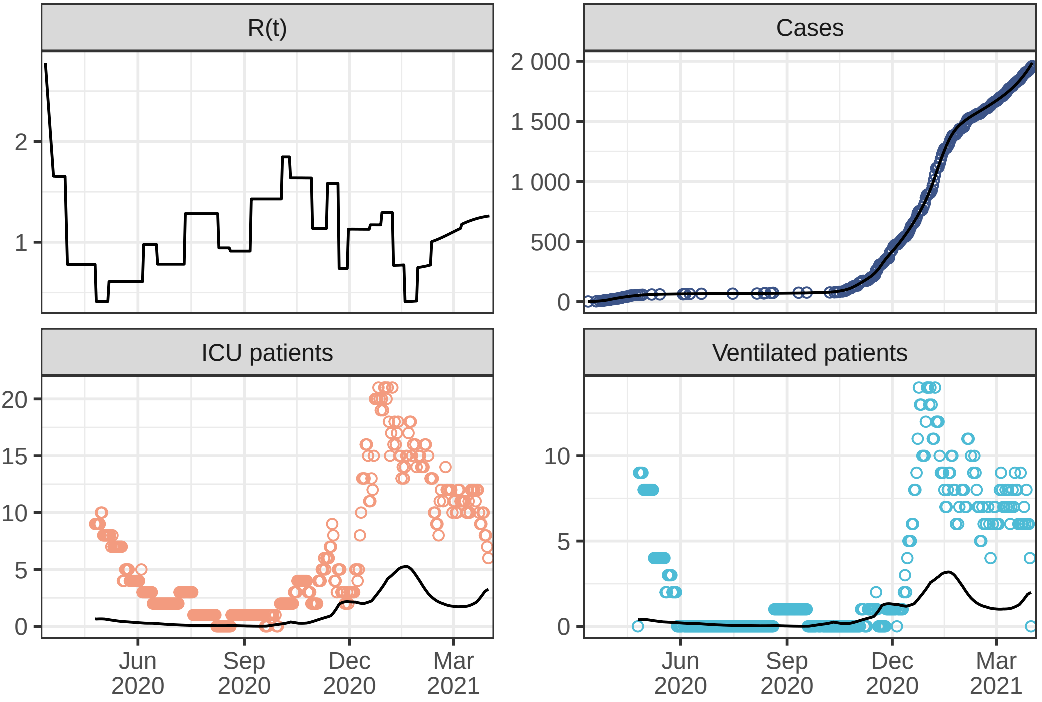

LK Nordsachsen

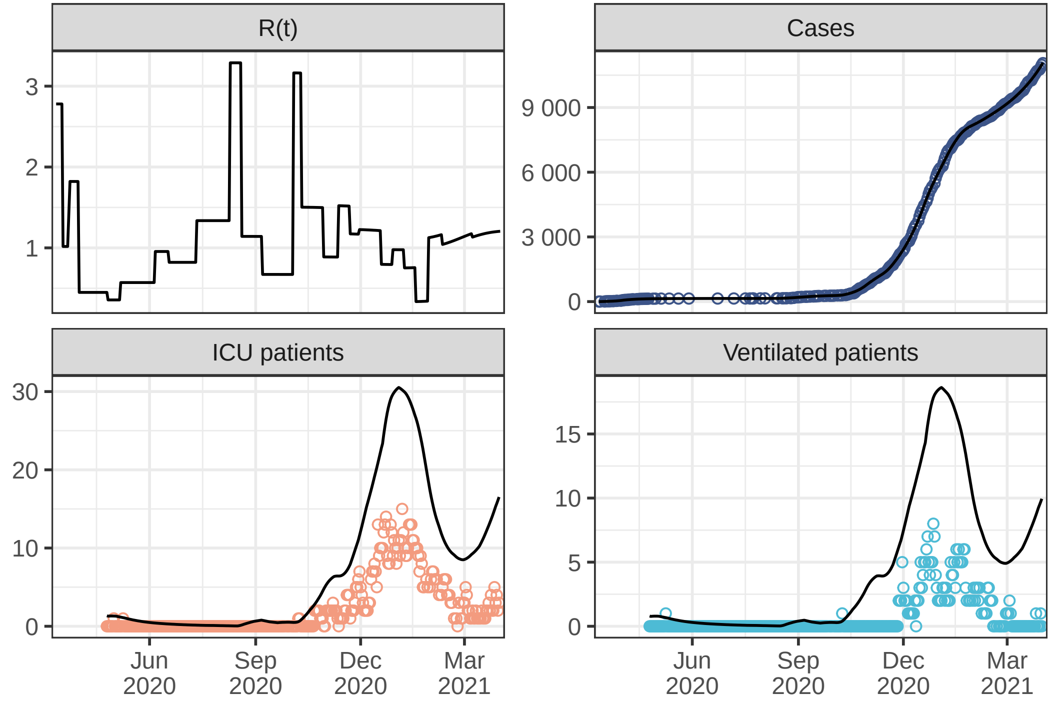

LK Nordwestmecklenburg

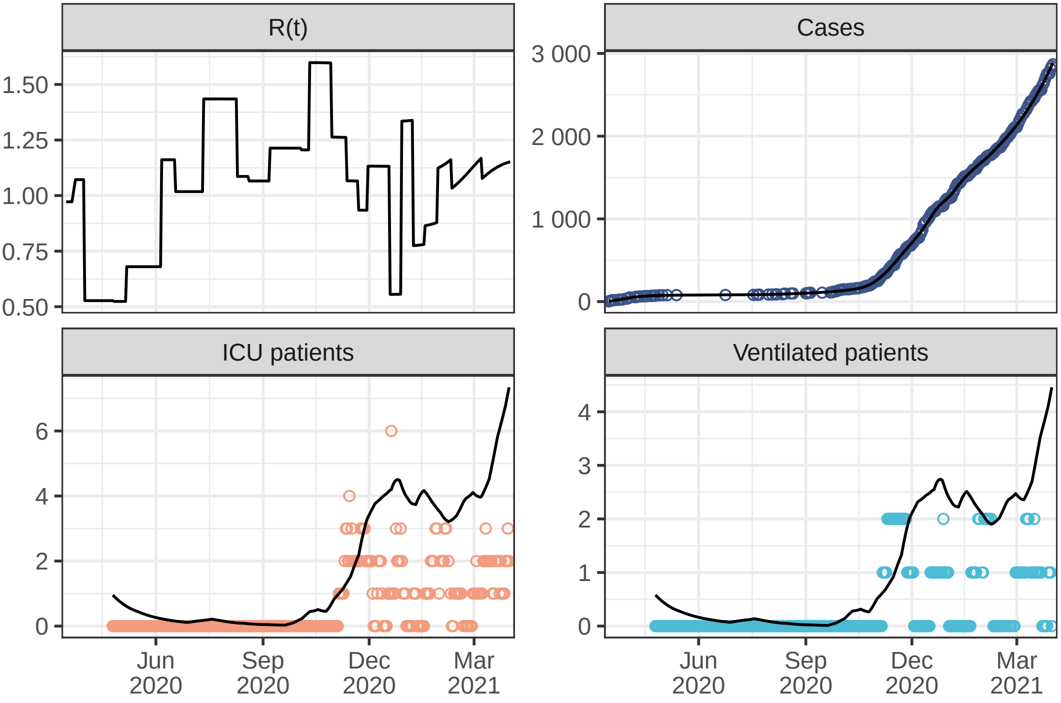

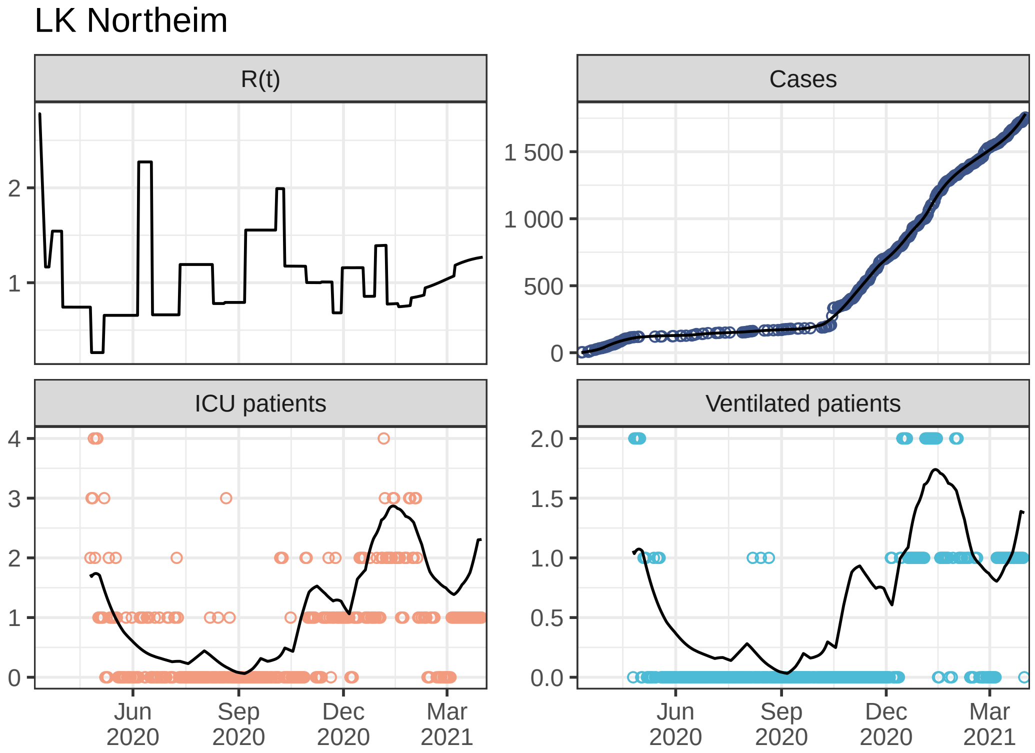
LK Northeim
R(t)
Cases
ICU patients
Ventilated patients
2
1
1 500
1 000
500
0
4
3
2
1
0
2.0
1.5
1.0
0.5
0.0
Jun 2020
Sep 2020
Dec 2020
Mar 2021

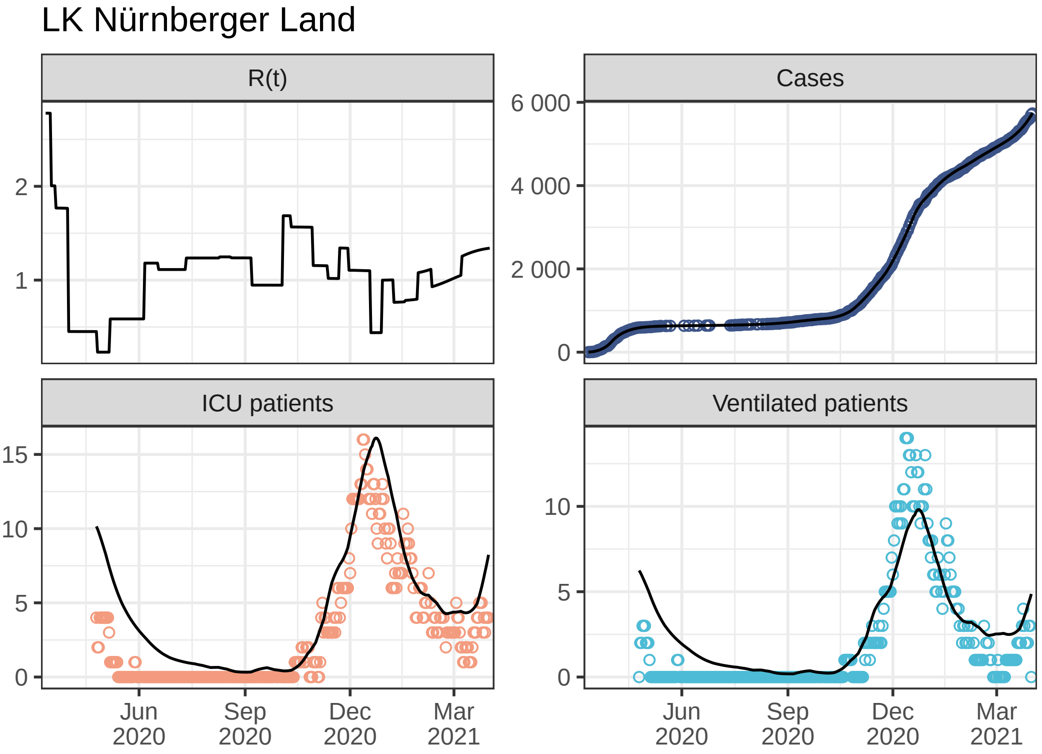
LK Nürnberger Land
R(t)
Cases
ICU patients
Ventilated patients
2
1
6 000
4 000
2 000
0
15
10
5
0
10
5
0
Jun 2020
Sep 2020
Dec 2020
Mar 2021

LK Oberallgäu

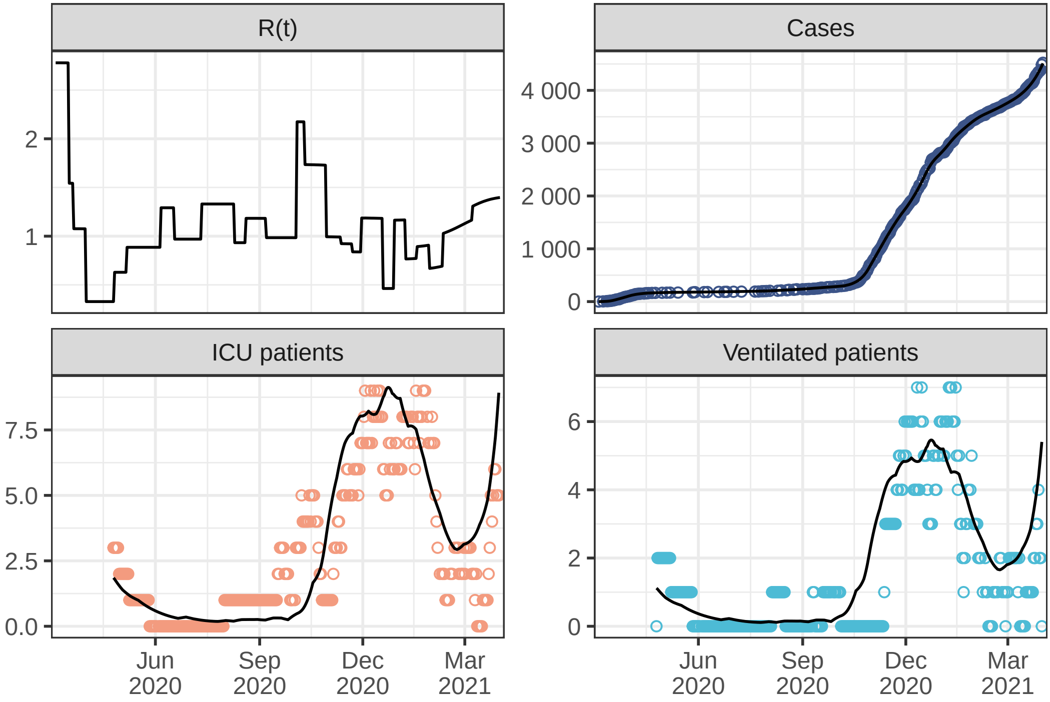

LK Oberbergischer Kreis

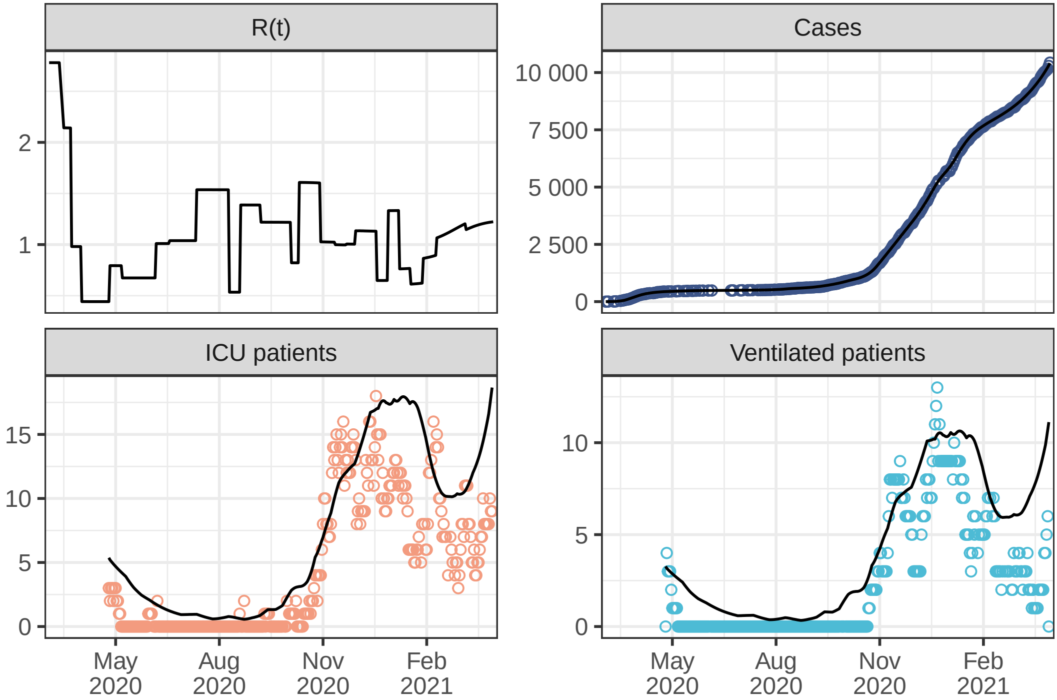

LK Oberhavel

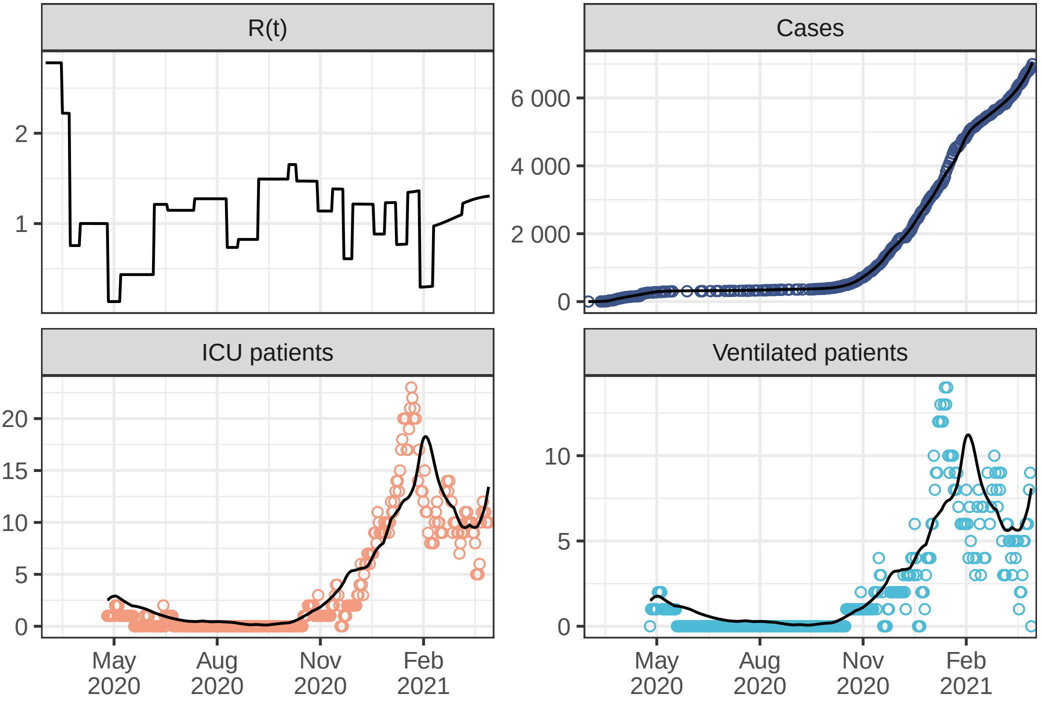

LK Oberspreewald−Lausitz

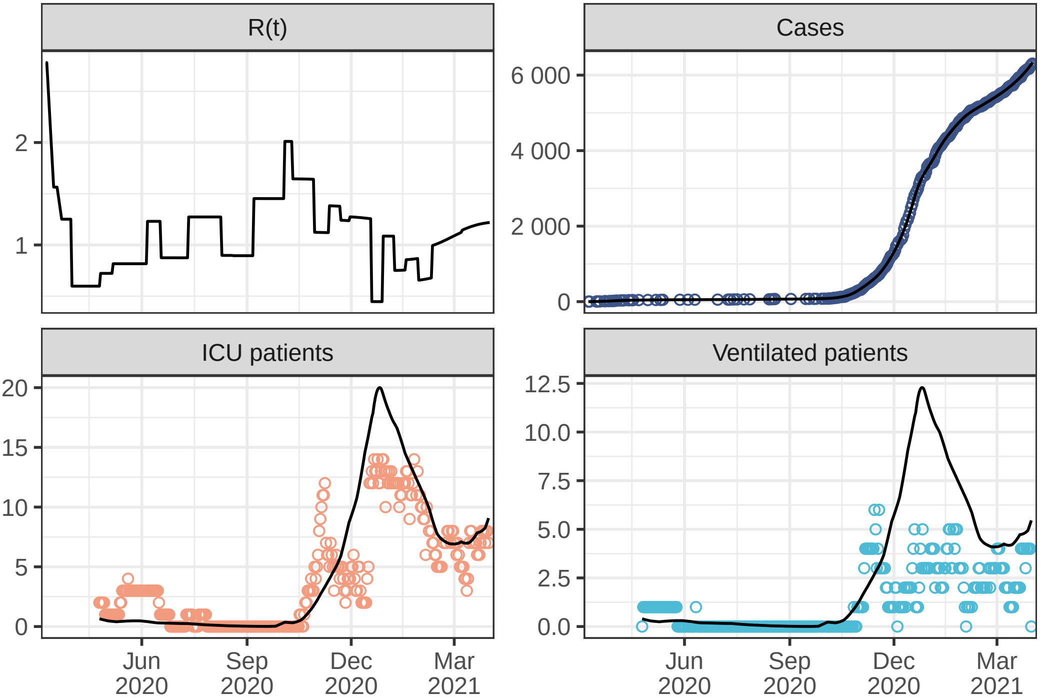

LK Odenwaldkreis

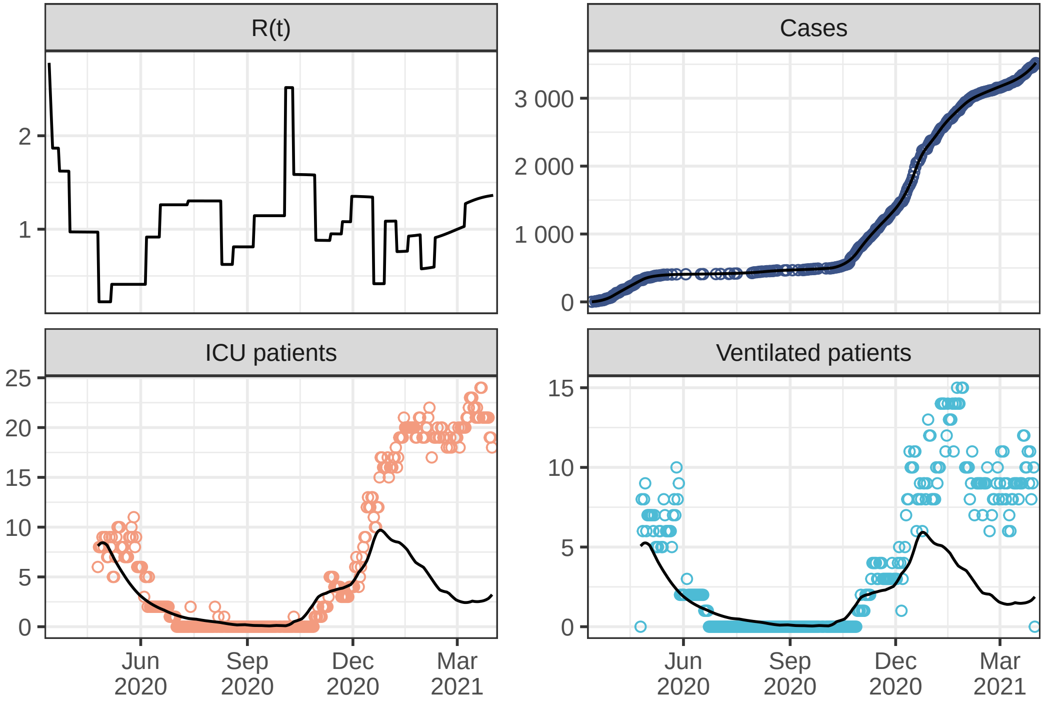

LK Oder−Spree

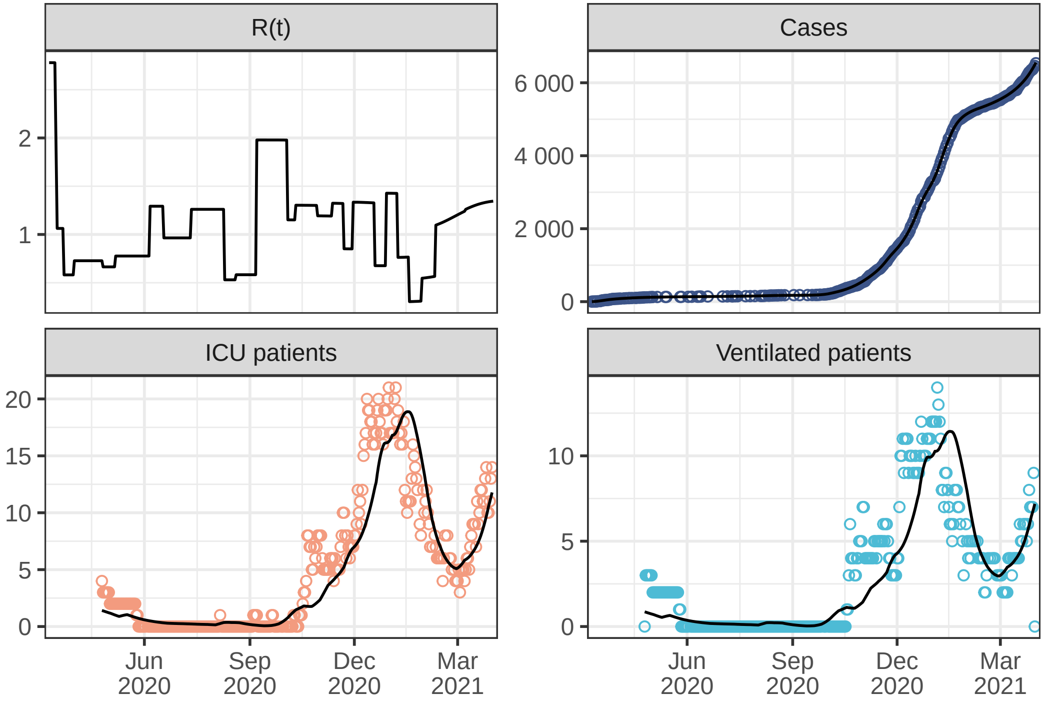

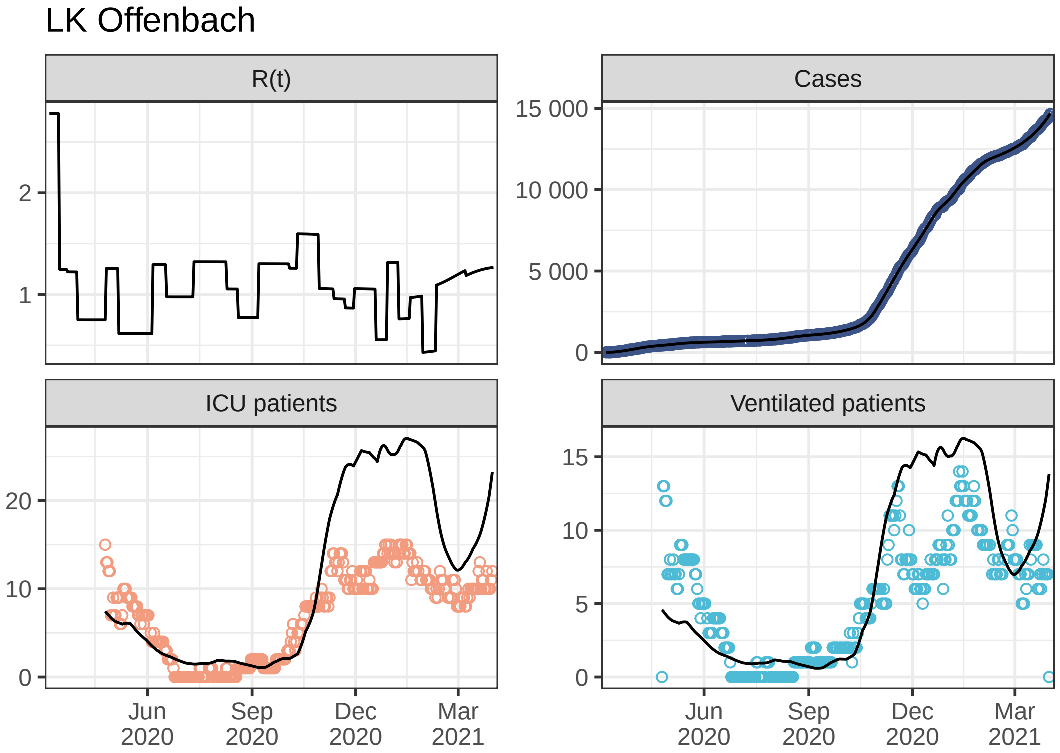
LK Offenbach
R(t)
Cases
ICU patients
Ventilated patients
2
1
15 000
10 000
5 000
0
20
10
0
15
10
5
0
Jun 2020
Sep 2020
Dec 2020
Mar 2021

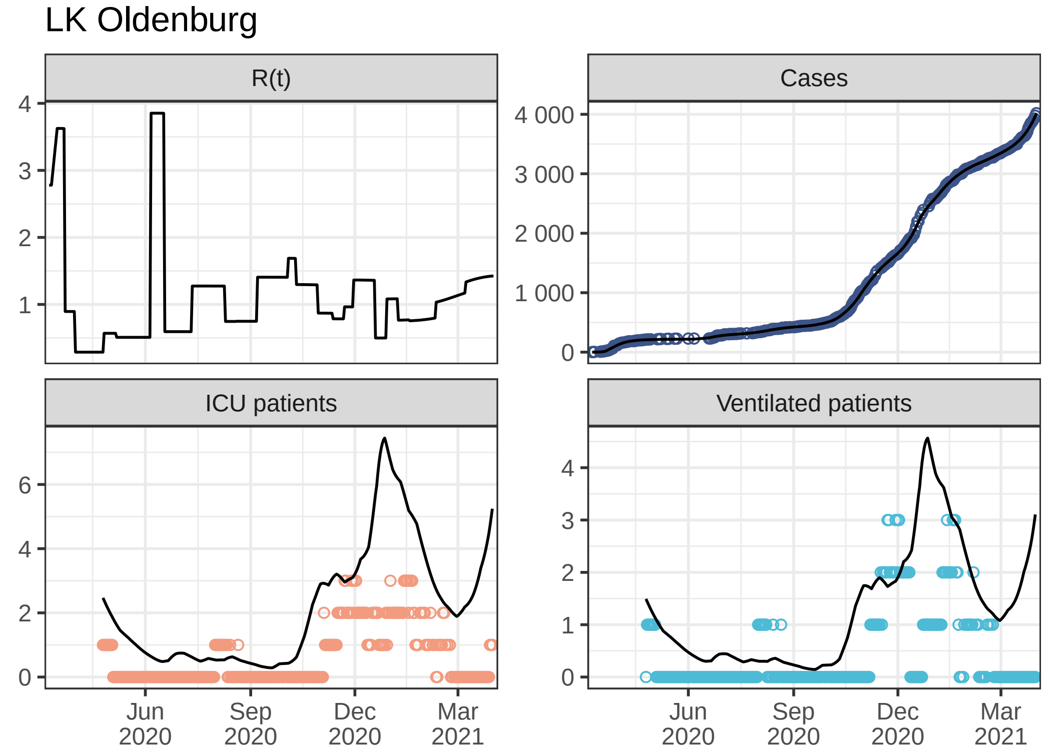
LK Oldenburg
R(t)
Cases
ICU patients
Ventilated patients
4
3
2
1
4 000
3 000
2 000
1 000
0
6
4
2
0
4
3
2
1
0
Jun 2020
Sep 2020
Dec 2020
Mar 2021

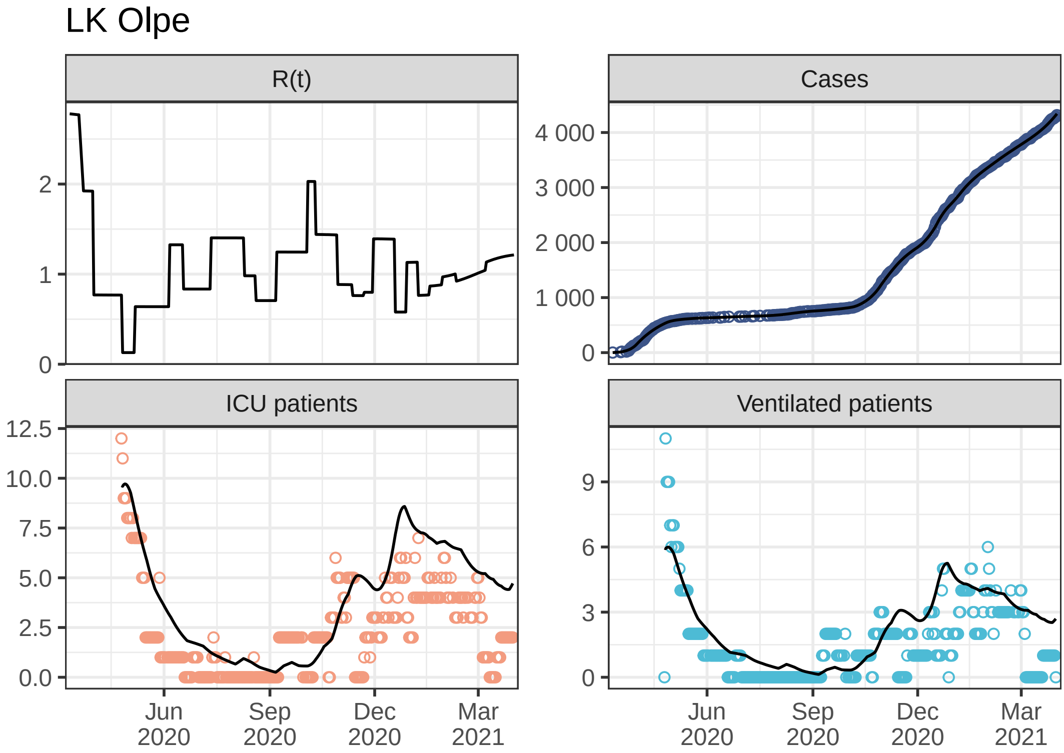
LK Olpe
R(t)
Cases
ICU patients
Ventilated patients
0
1
2
4 000
3 000
2 000
1 000
0
12.5
10.0
7.5
5.0
2.5
0.0
9
6
3
0
Jun 2020
Sep 2020
Dec 2020
Mar 2021
Jun 2020
Sep 2020
Dec 2020
Mar 2021

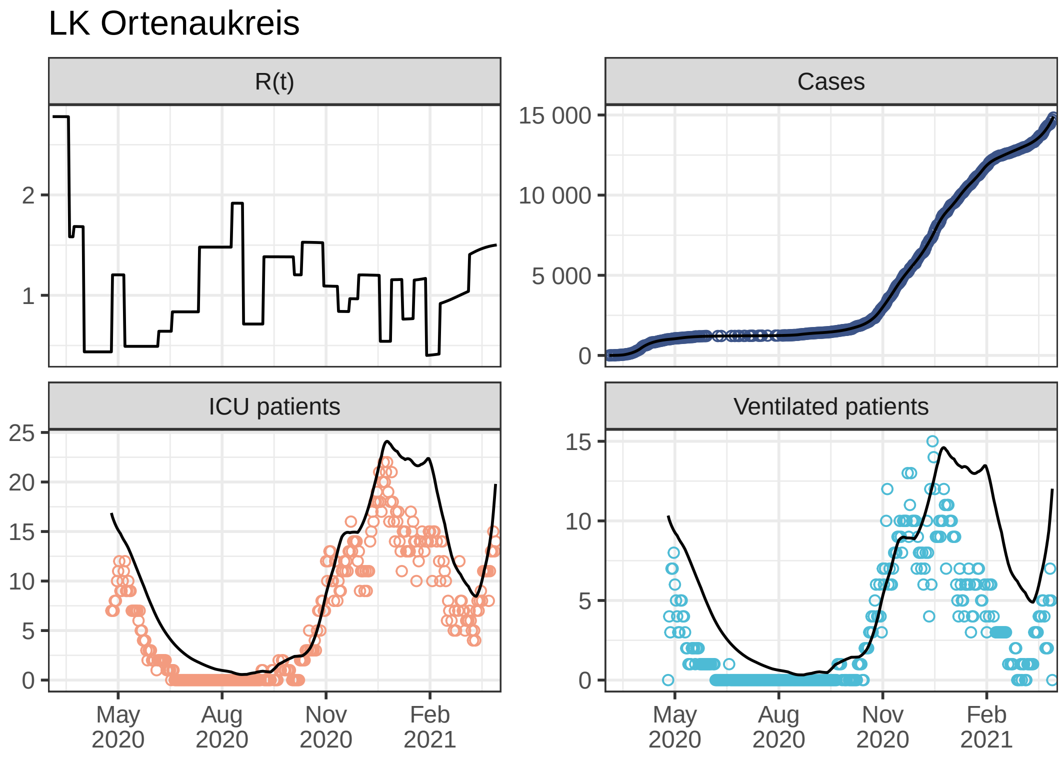
LK Ortenaukreis
R(t)
Cases
ICU patients
Ventilated patients
1
2
15 000
10 000
5 000
0
25
20
15
10
5
0
15
10
5
0
May 2020
Aug 2020
Nov 2020
Feb 2021
May 2020
Aug 2020
Nov 2020
Feb 2021

LK Osnabrück

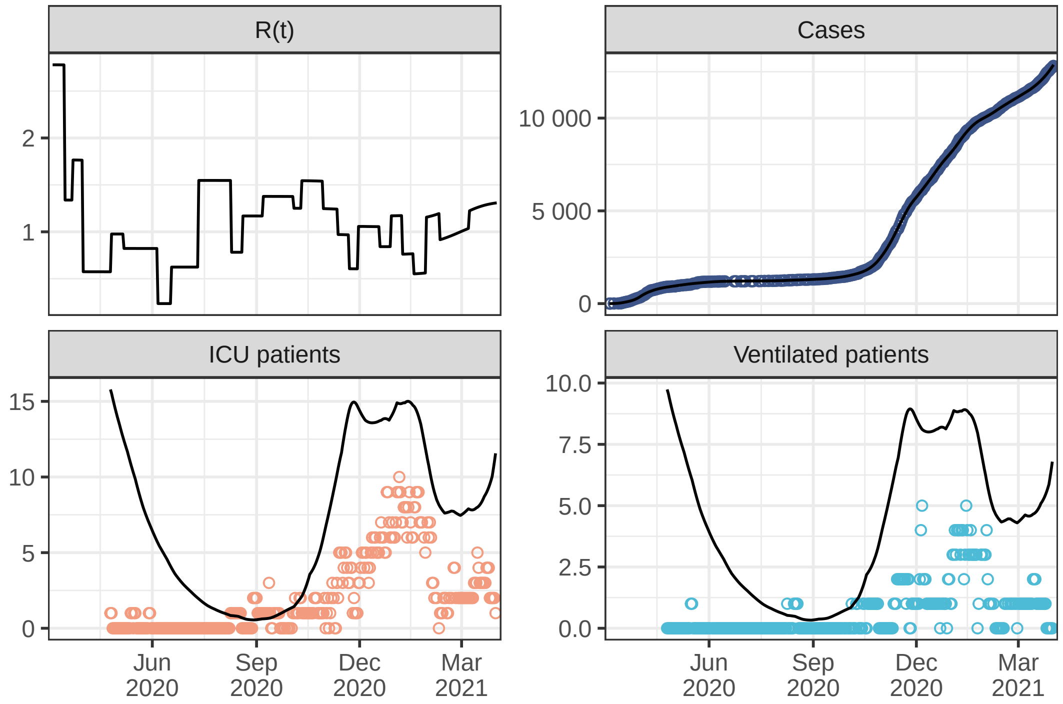

LK Ostalbkreis

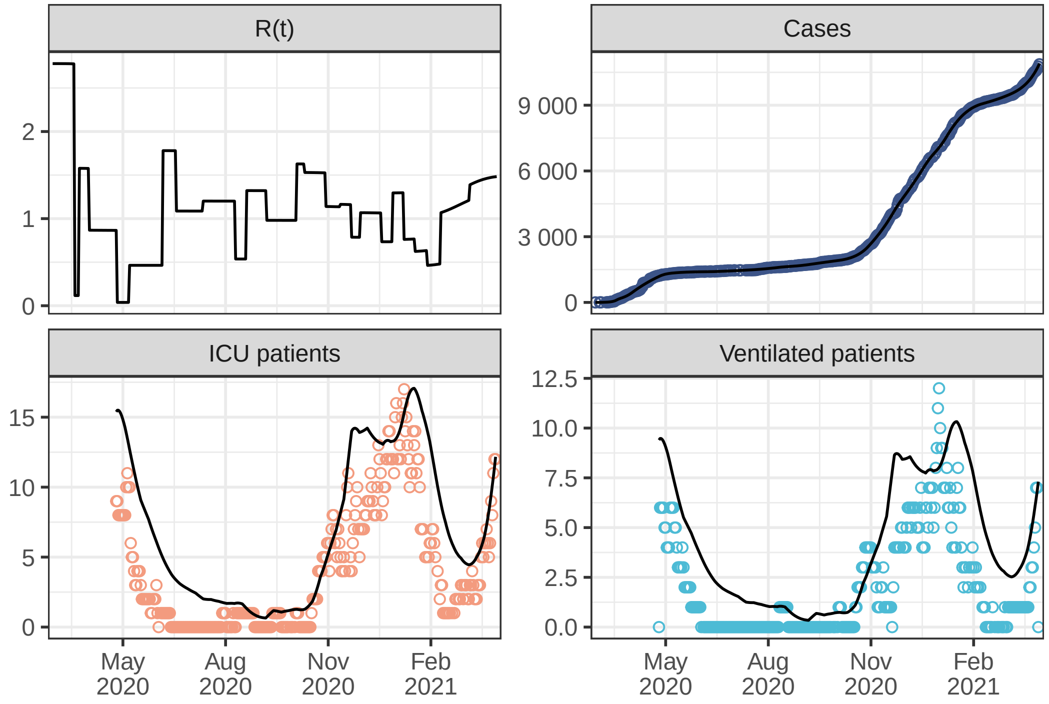

LK Ostallgäu

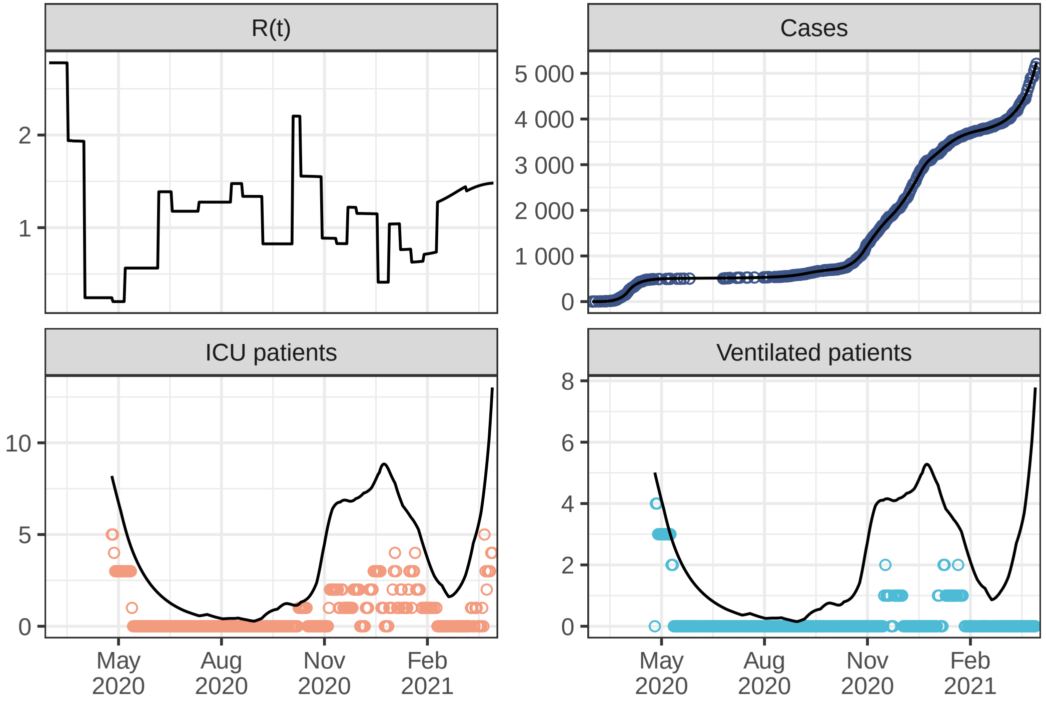

LK Osterholz

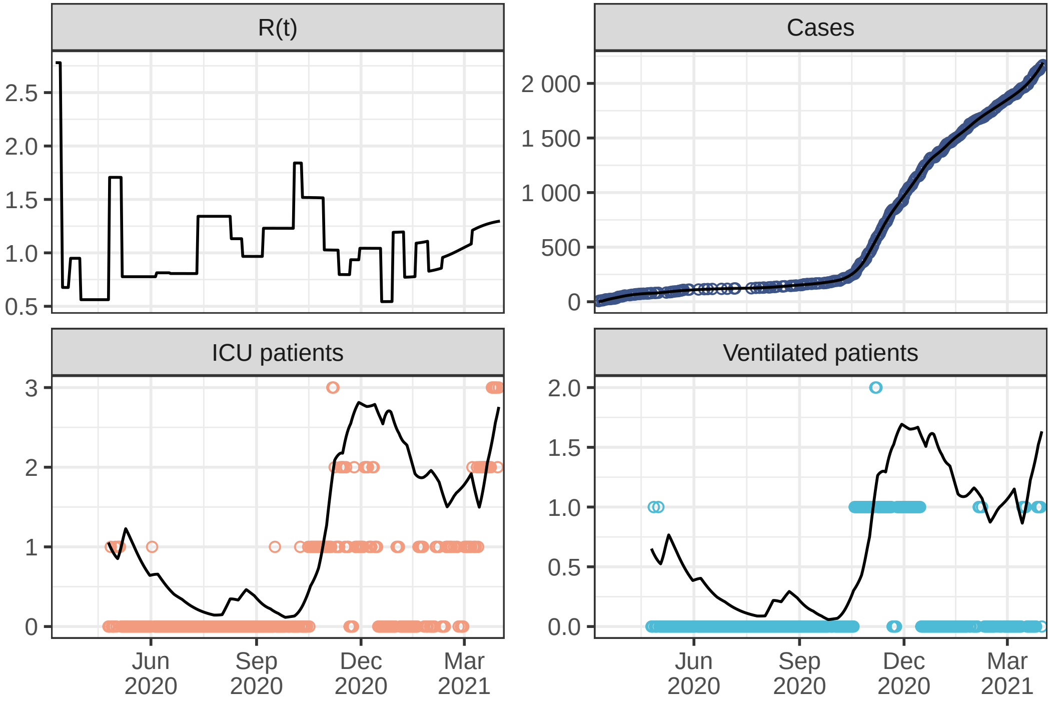

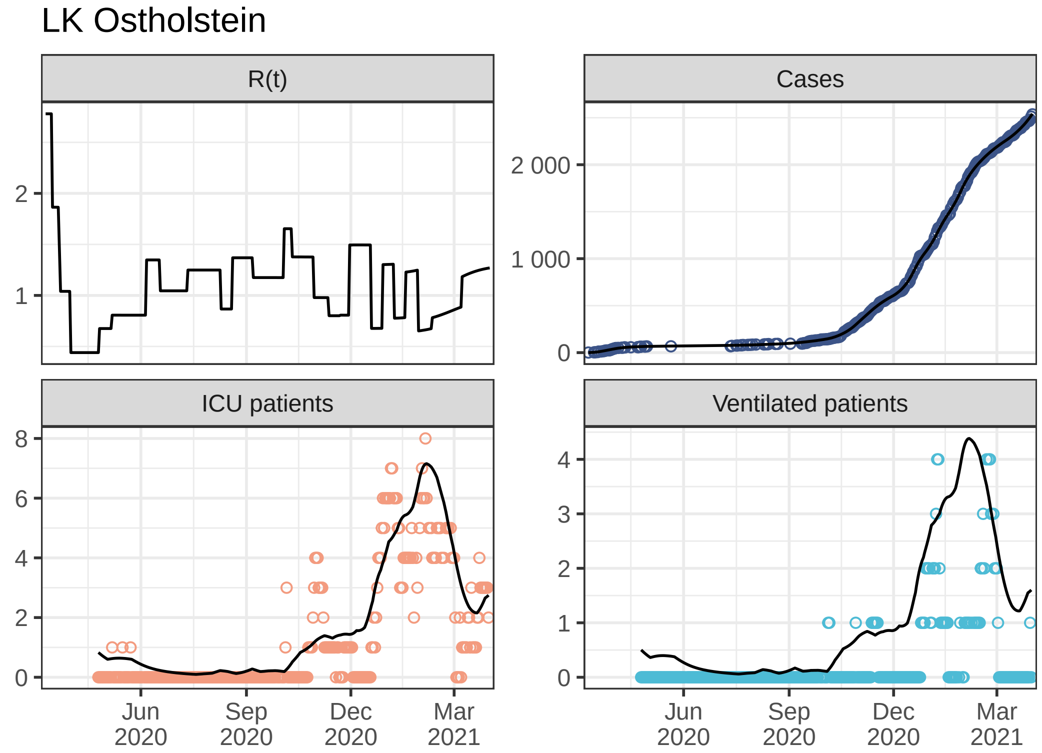
LK Ostholstein
R(t)
Cases
ICU patients
Ventilated patients
2
1
2 000
1 000
0
8
6
4
2
0
4
3
2
1
0
Jun 2020
Sep 2020
Dec 2020
Mar 2021

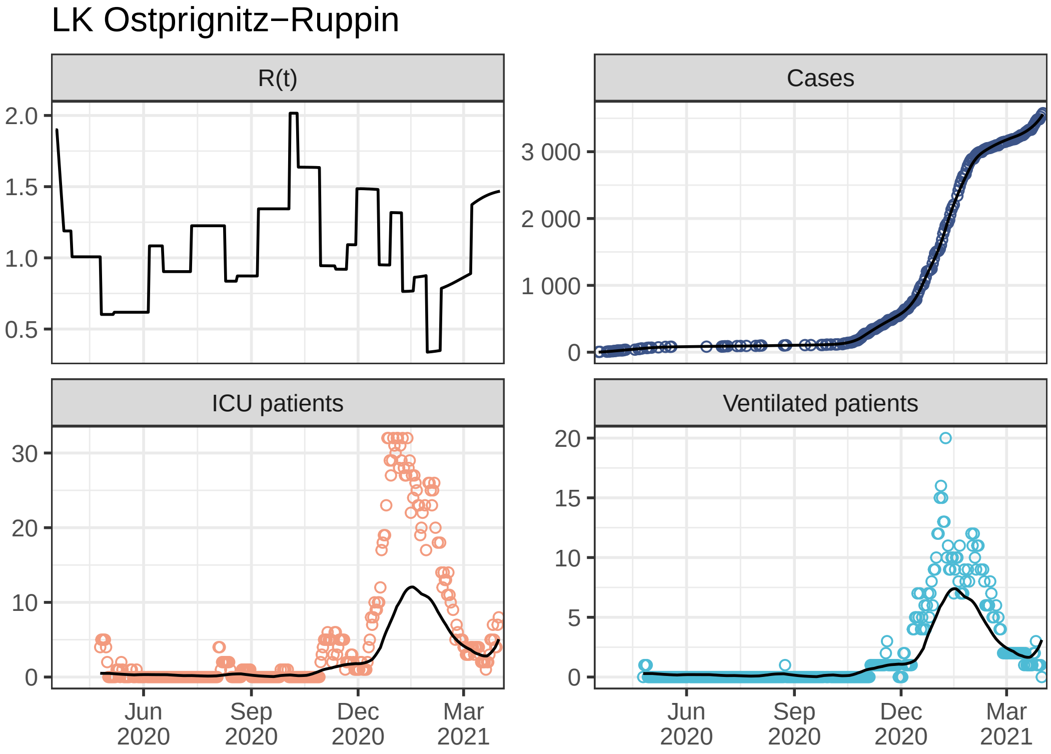
LK Ostprignitz−Ruppin
R(t)
Cases
ICU patients
Ventilated patients
2.0
1.5
1.0
0.5
3 000
2 000
1 000
0
30
20
10
0
20
15
10
5
0
Jun 2020
Sep 2020
Dec 2020
Mar 2021

LK Paderborn

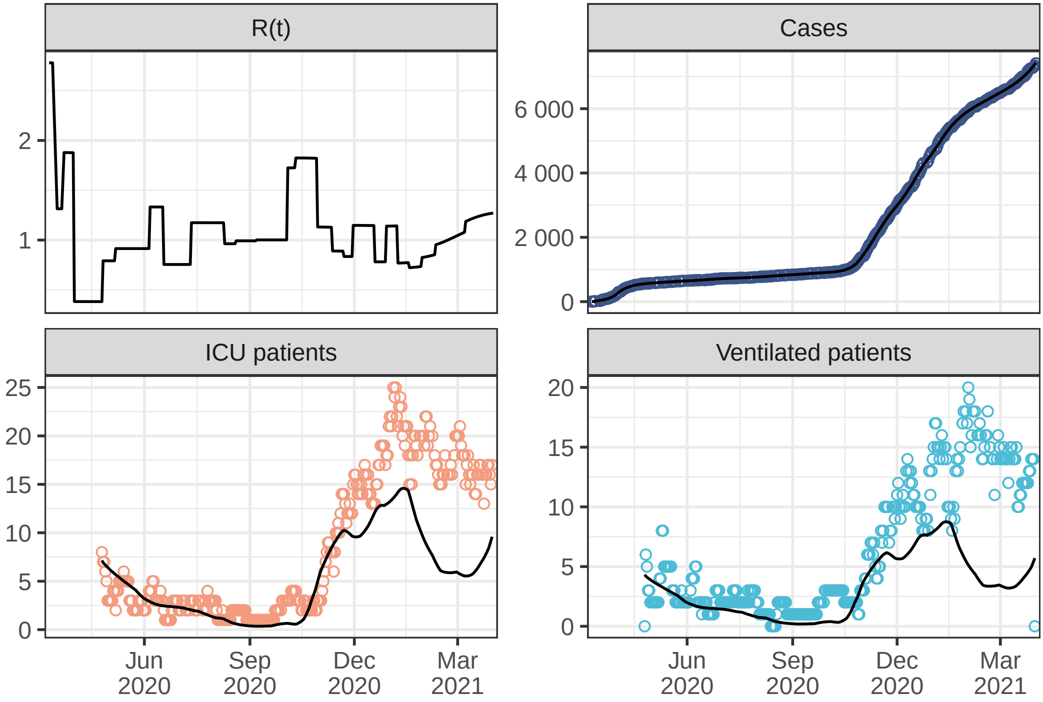

LK Passau

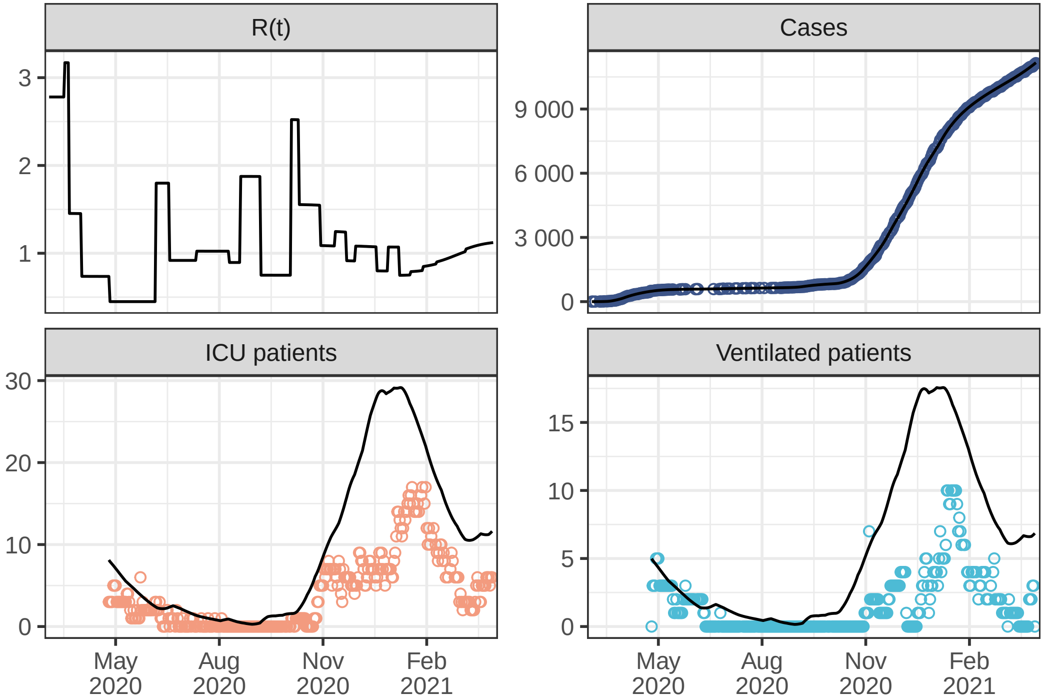

LK Peine

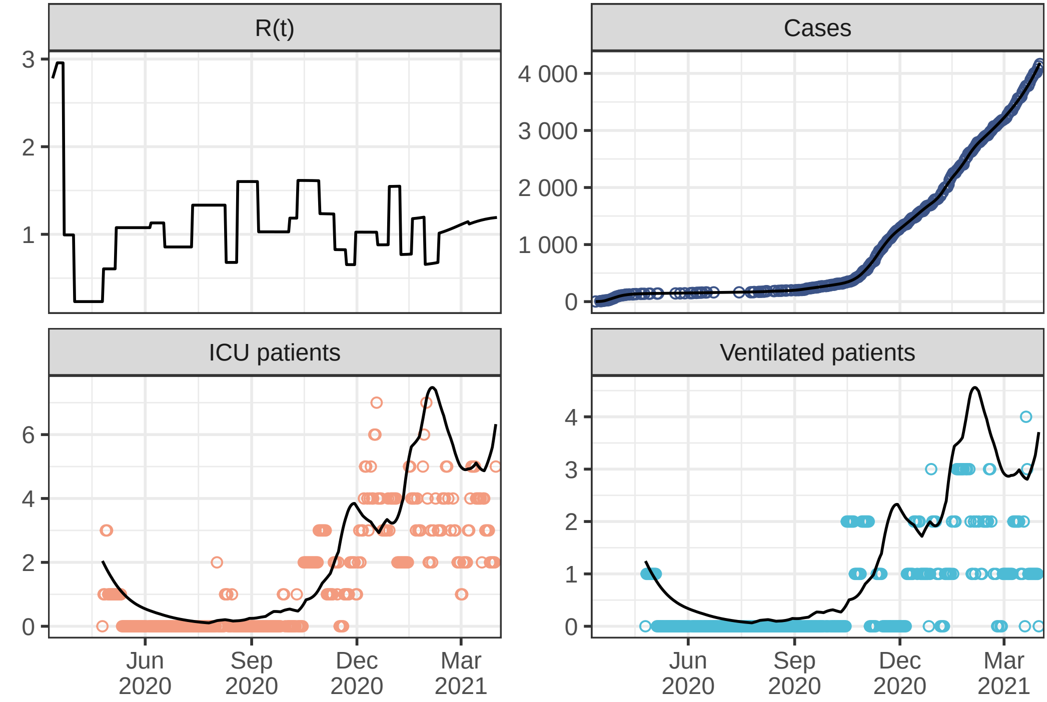

LK Pfaffenhofen a.d.Ilm

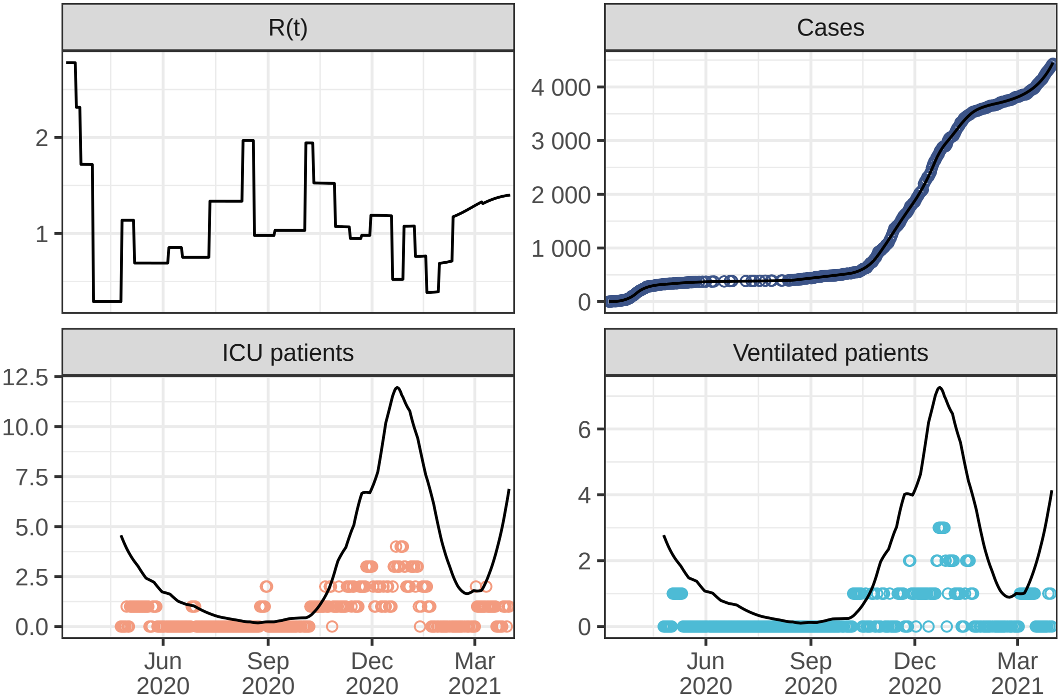

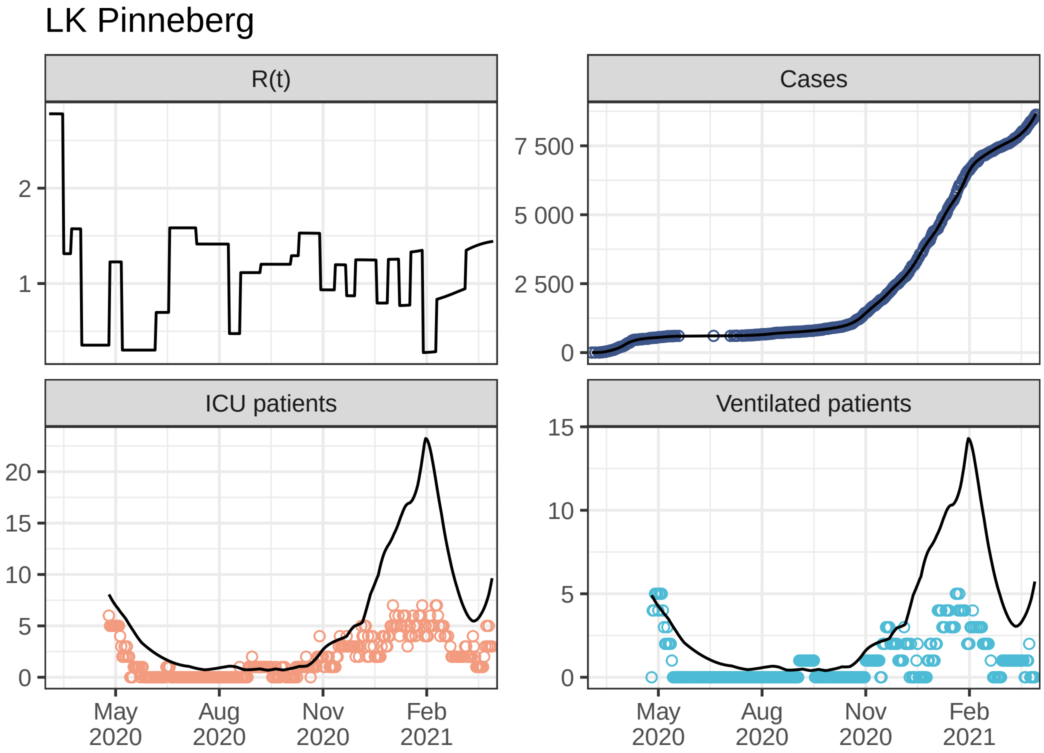
LK Pinneberg
R(t)
Cases
ICU patients
Ventilated patients
2
1
7 500
5 000
2 500
0
20
15
10
5
0
15
10
5
0
May 2020
Aug 2020
Nov 2020
Feb 2021

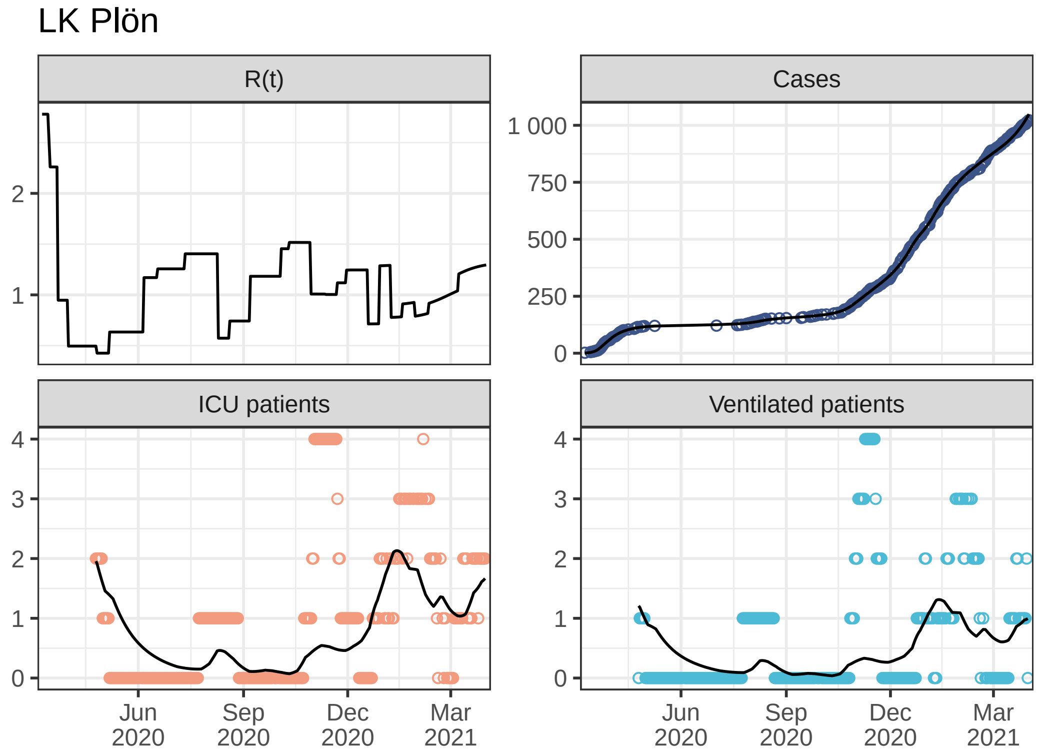
LK Plön
R(t)
Cases
ICU patients
Ventilated patients
2
1
1 000
750
500
250
0
4
3
2
1
0
Jun 2020
Sep 2020
Dec 2020
Mar 2021

LK Potsdam−Mittelmark

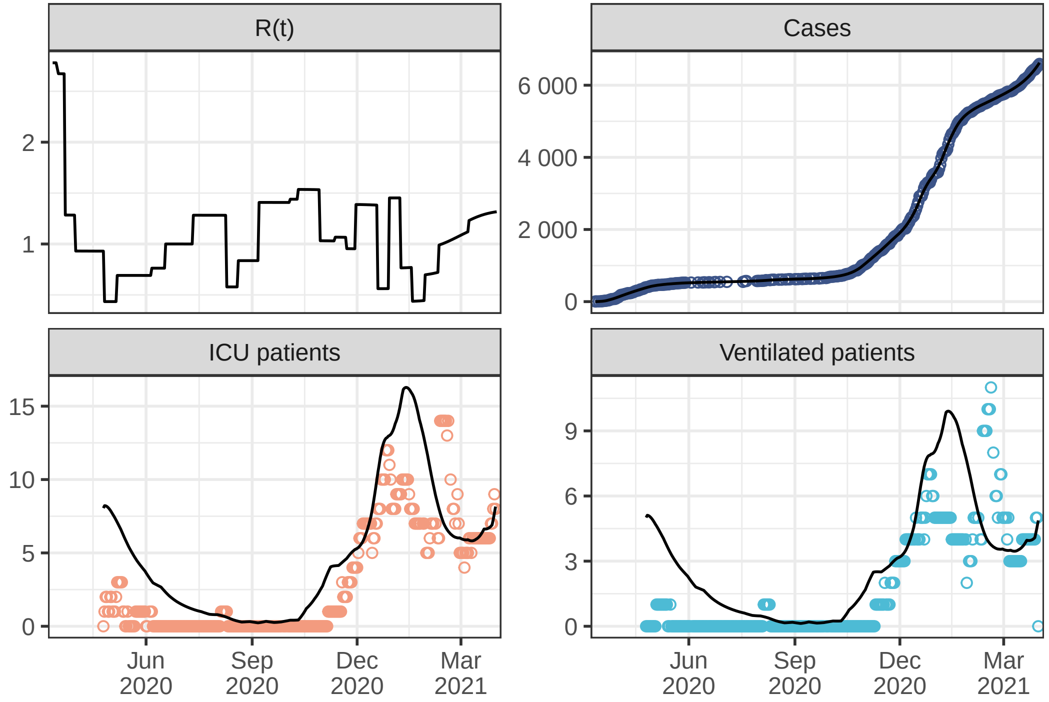

LK Prignitz

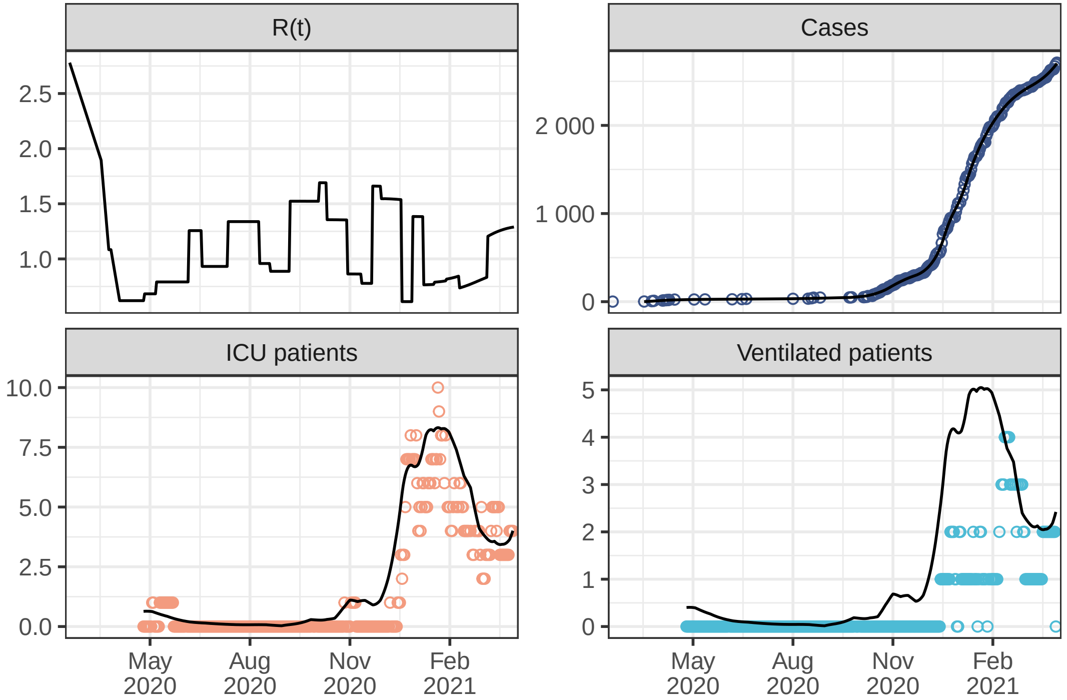

LK Rastatt

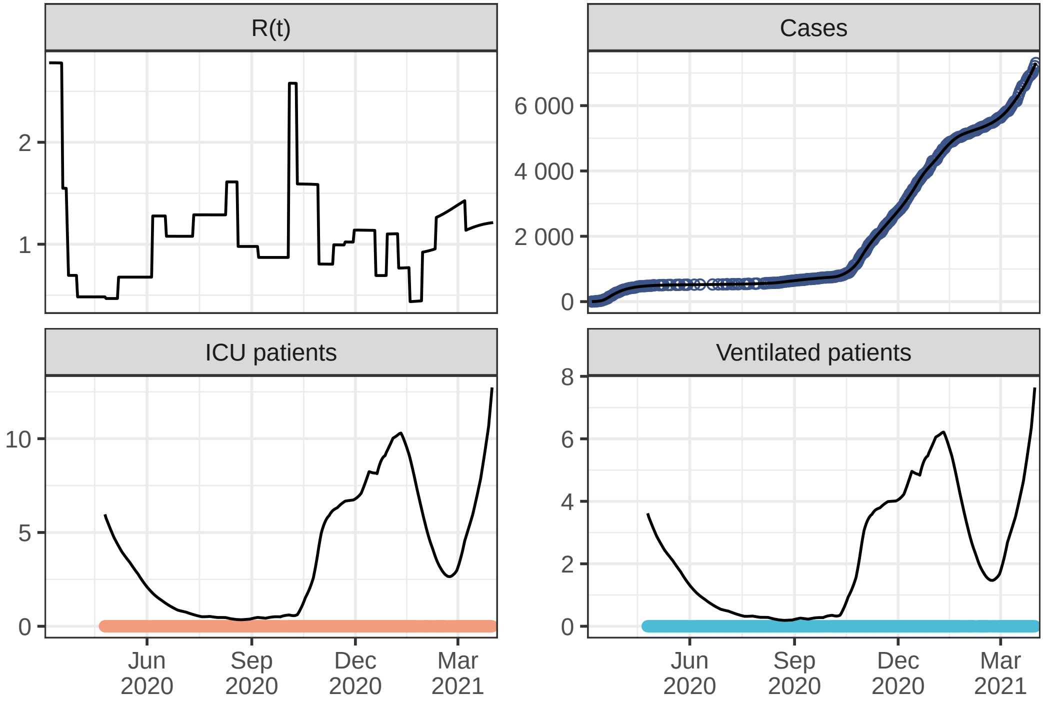

LK Ravensburg

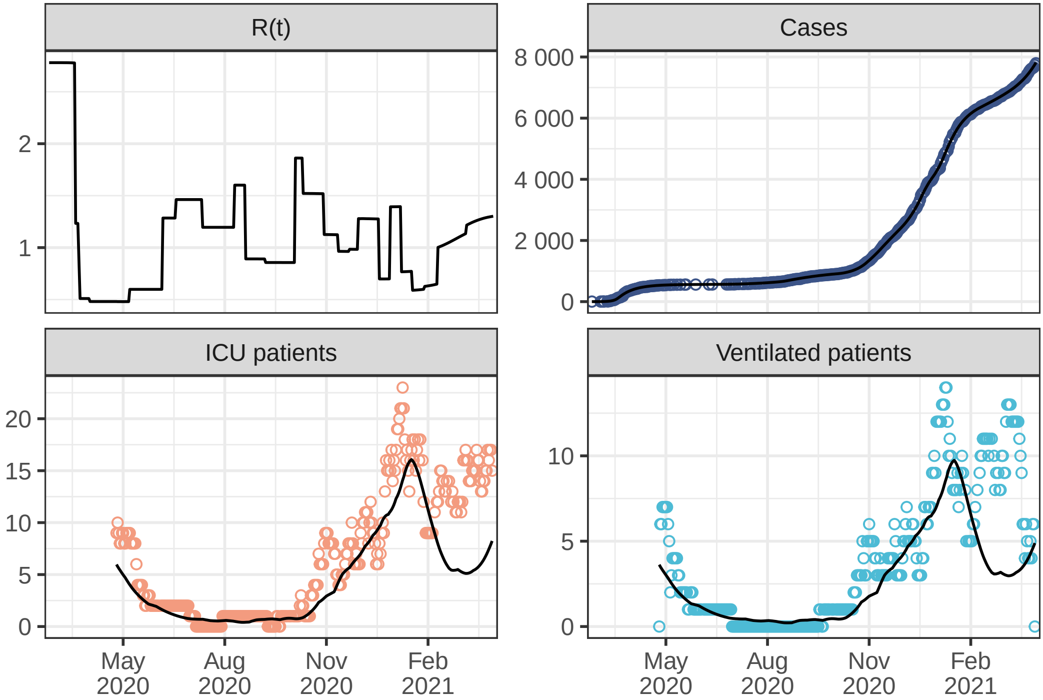

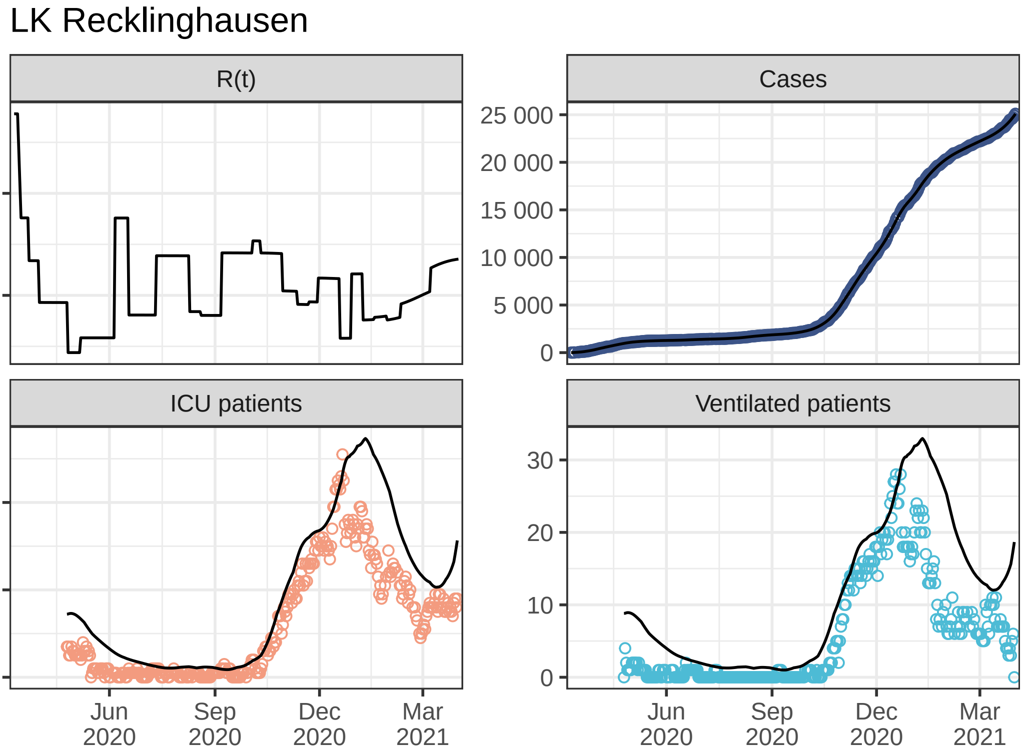
LK Recklinghausen
R(t)
Cases
ICU patients
Ventilated patients
2
1
25 000
20 000
15 000
10 000
5 000
0
40
20
0
30
20
10
0
Jun 2020
Sep 2020
Dec 2020
Mar 2021

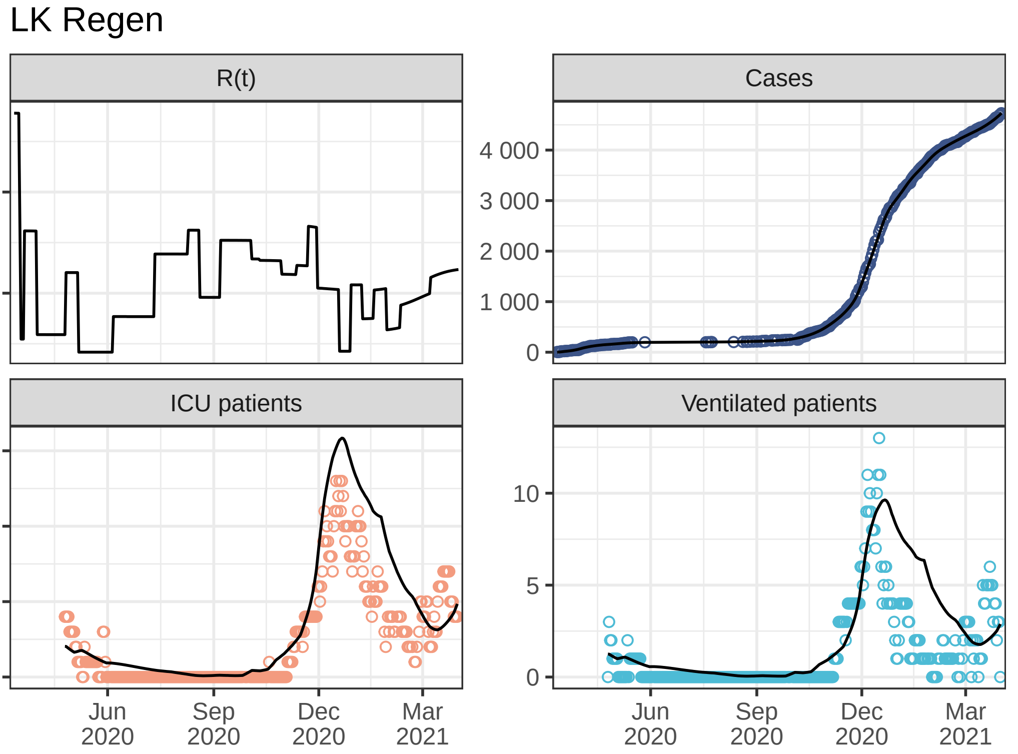
LK Regen
R(t)
Cases
ICU patients
Ventilated patients
2
1
4 000
3 000
2 000
1 000
0
15
10
5
0
10
5
0
Jun 2020
Sep 2020
Dec 2020
Mar 2021

LK Regensburg

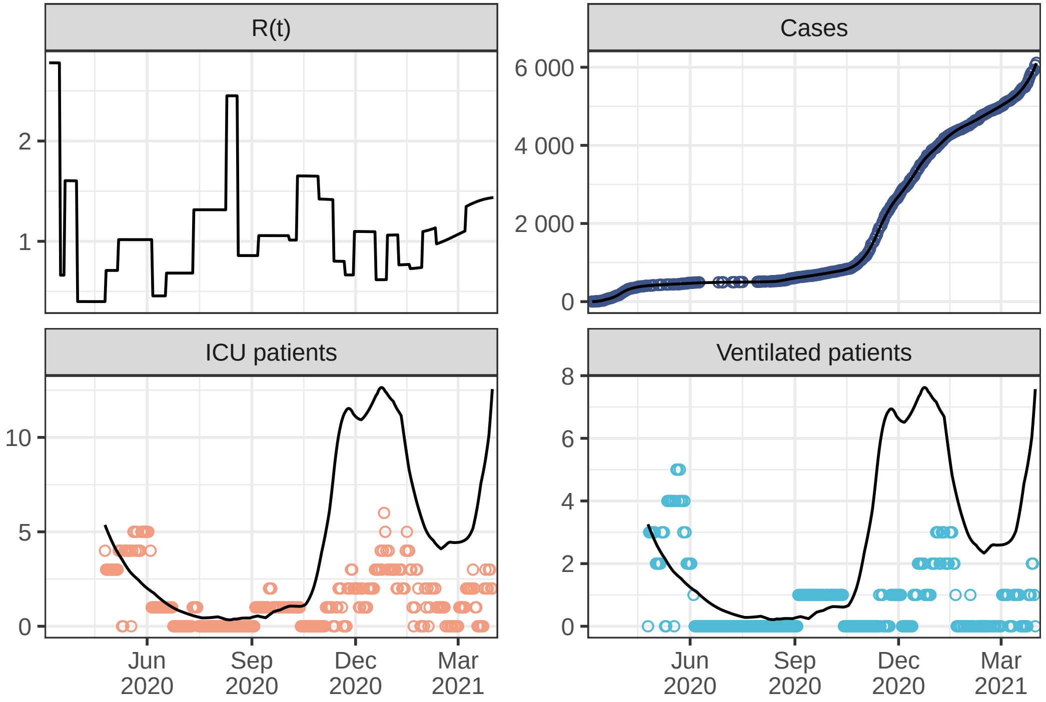

LK Rems−Murr−Kreis

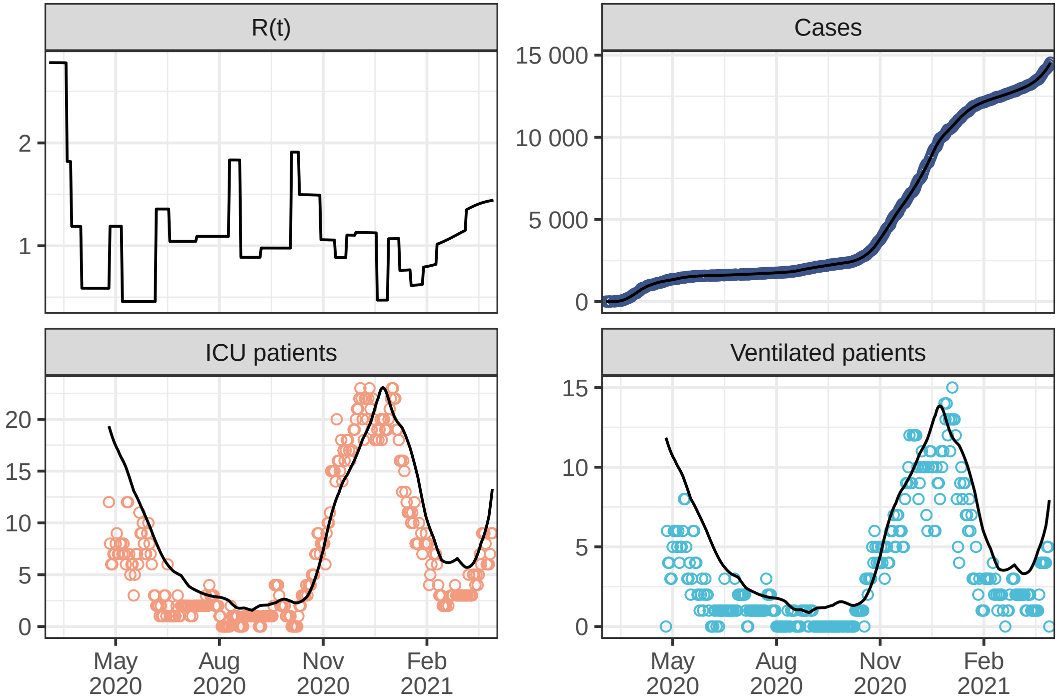

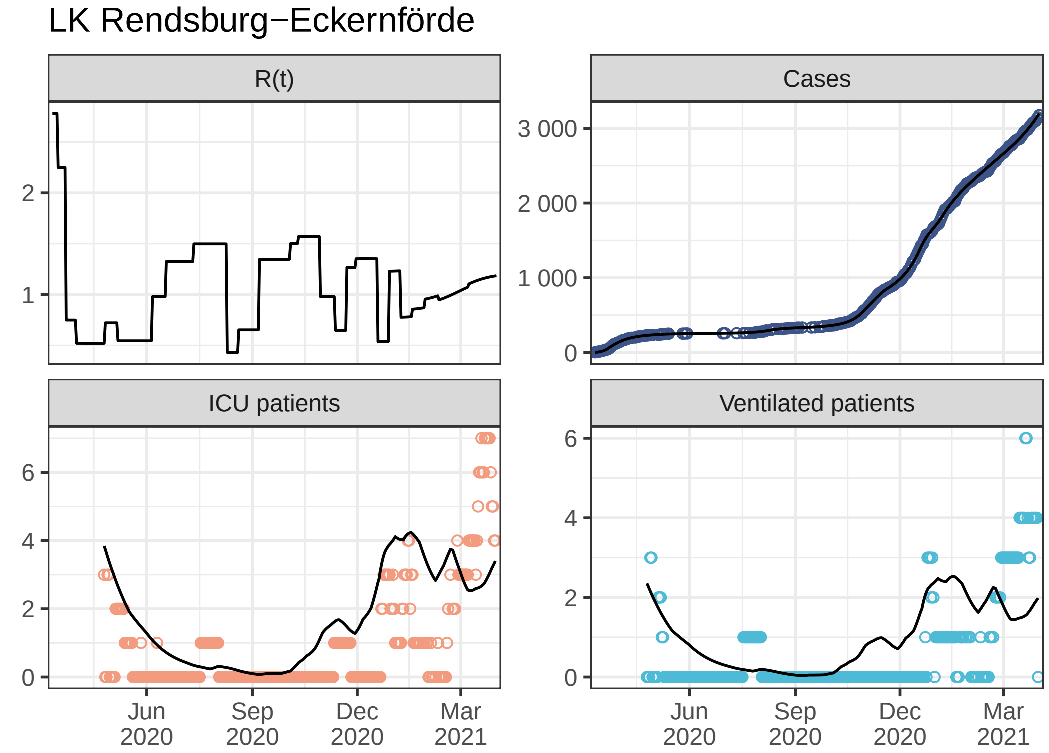
LK Rendsburg−Eckernförde
R(t)
Cases
ICU patients
Ventilated patients
1
2
0
1 000
2 000
3 000
0
2
4
6
0
2
4
6
Jun 2020
Sep 2020
Dec 2020
Mar 2021

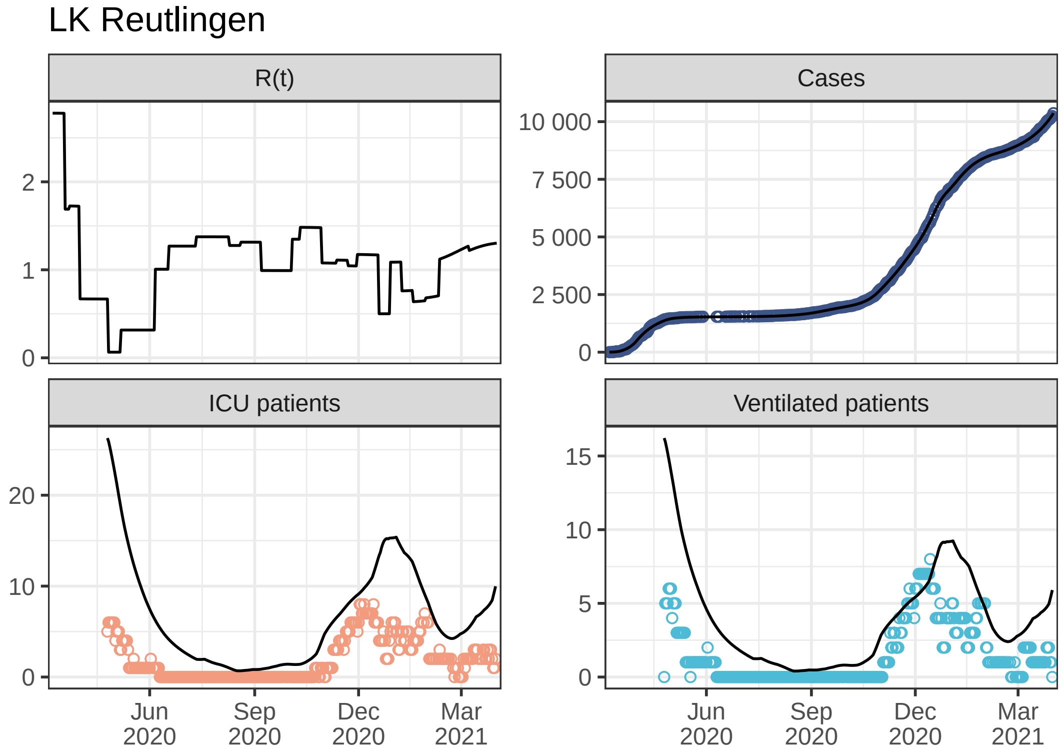
LK Reutlingen
R(t)
Cases
ICU patients
Ventilated patients
0
1
2
0
2 500
5 000
7 500
10 000
0
10
20
0
5
10
15
Jun 2020
Sep 2020
Dec 2020
Mar 2021

LK Rhein−Erft−Kreis

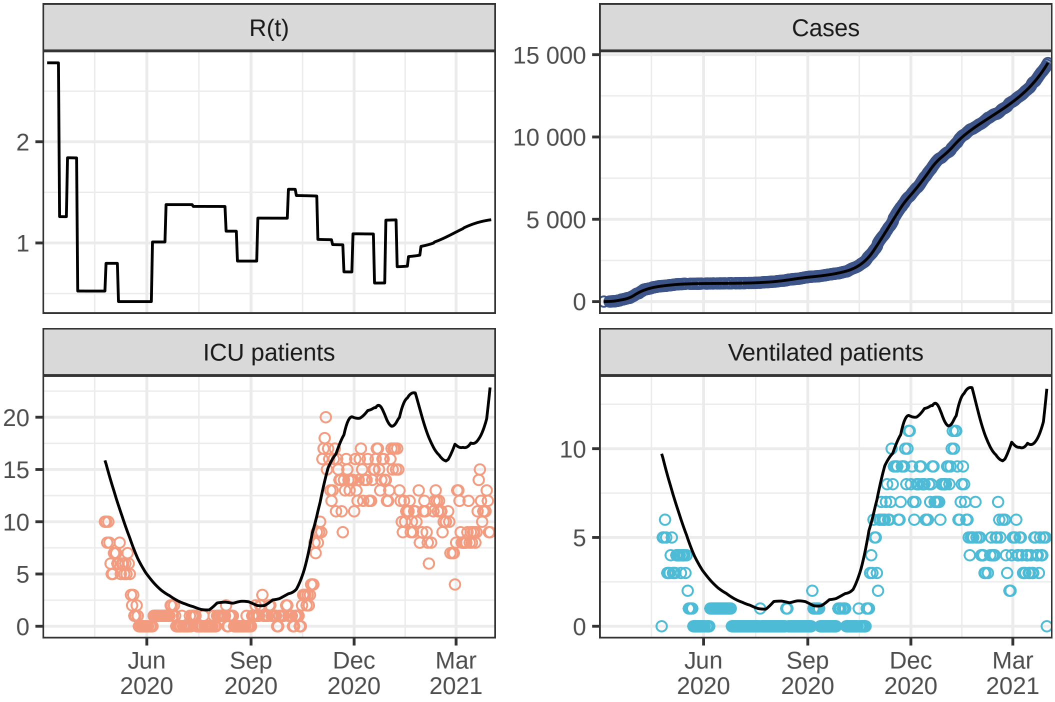

LK Rhein−Hunsrück−Kreis

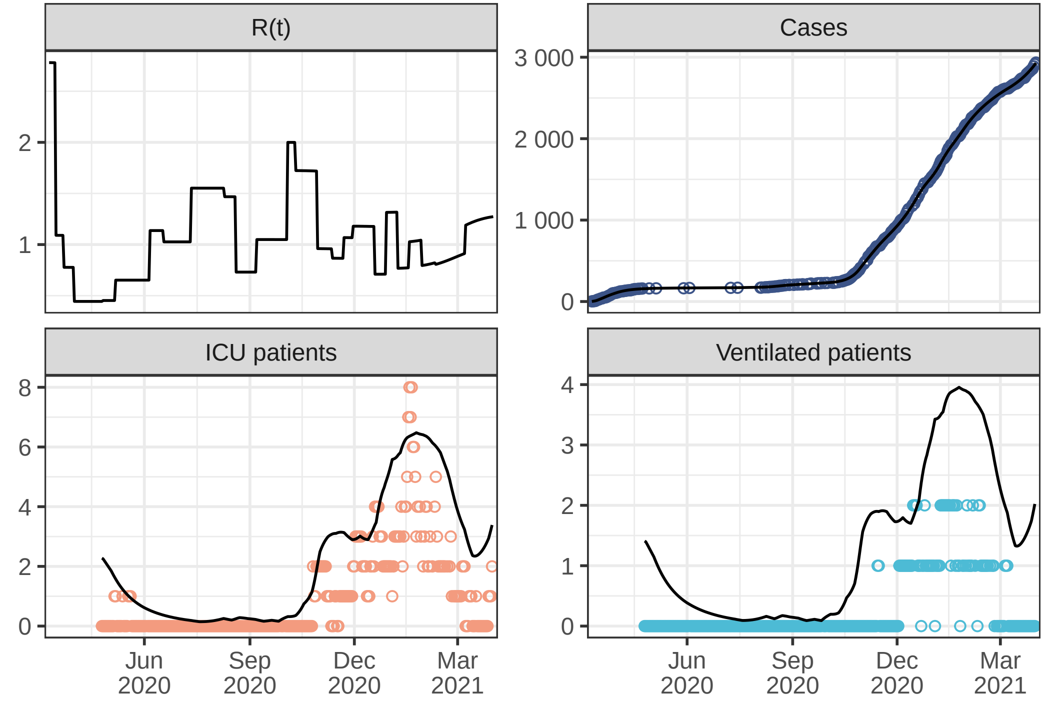

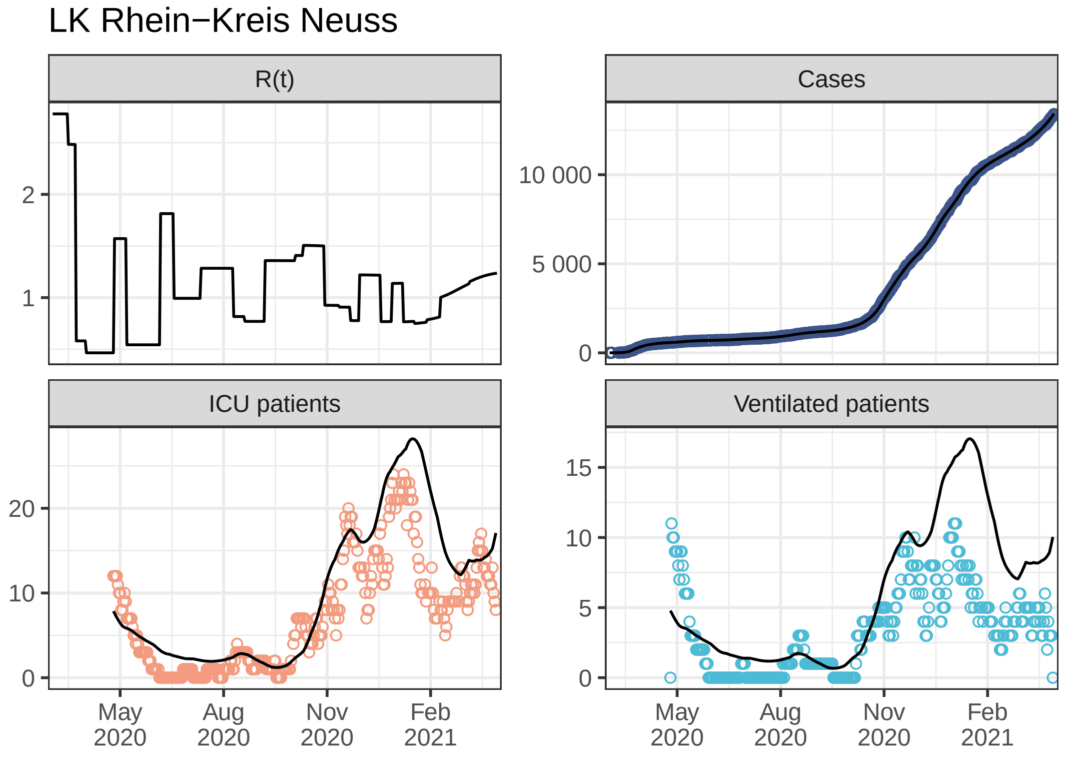
LK Rhein−Kreis Neuss
R(t)
Cases
ICU patients
Ventilated patients
1
2
0
5 000
10 000
0
10
20
0
5
10
15
May 2020
Aug 2020
Nov 2020
Feb 2021

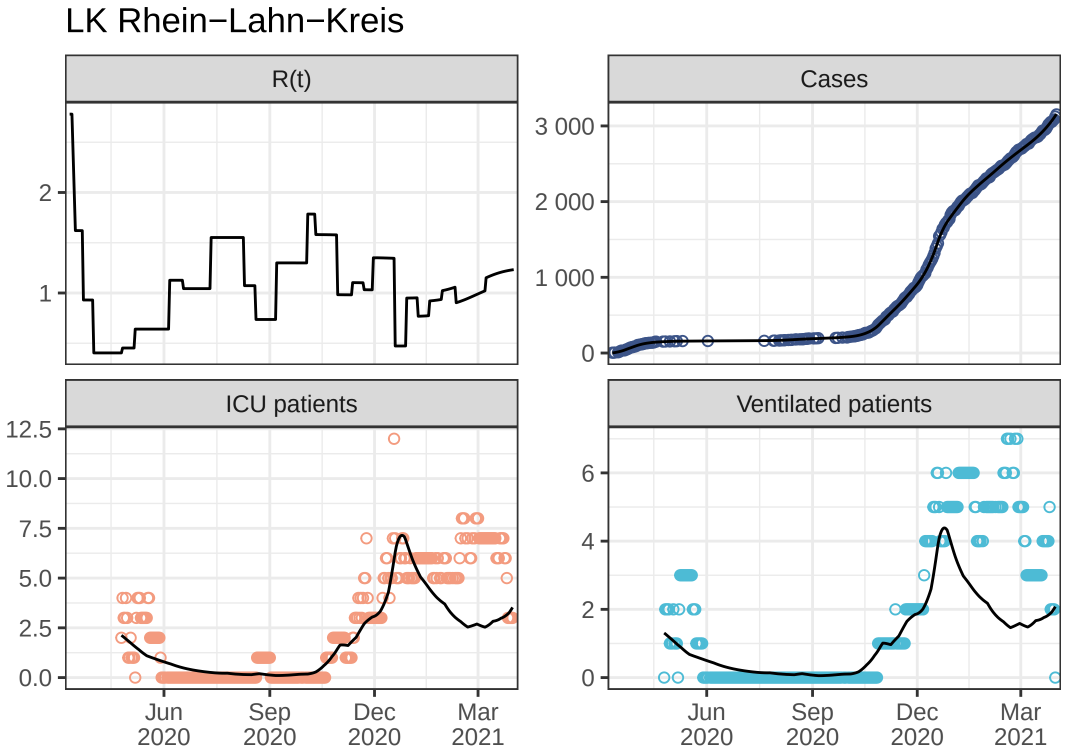
LK Rhein−Lahn−Kreis
R(t)
Cases
ICU patients
Ventilated patients
1
2
0
1 000
2 000
3 000
0.0
2.5
5.0
7.5
10.0
12.5
0
2
4
6
Jun 2020
Sep 2020
Dec 2020
Mar 2021

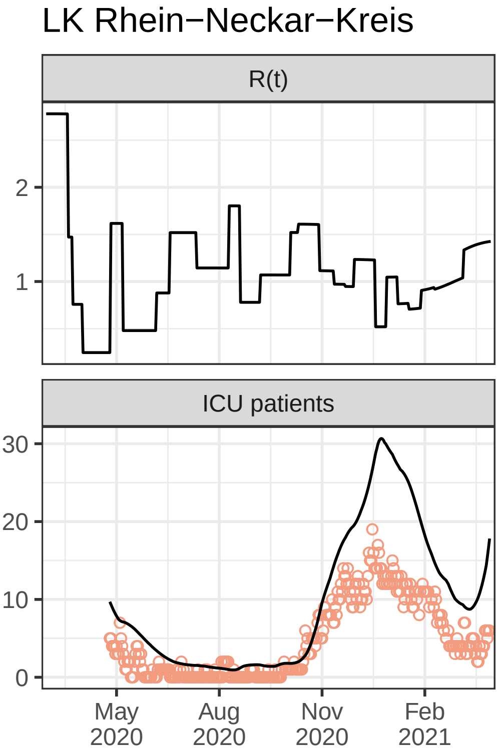

LK Rhein−Neckar−Kreis
R(t)
Cases
ICU patients
Ventilated patients
2
1
15 000
10 000
5 000
0
30
20
10
0
15
10
5
0
May 2020
Aug 2020
Nov 2020
Feb 2021

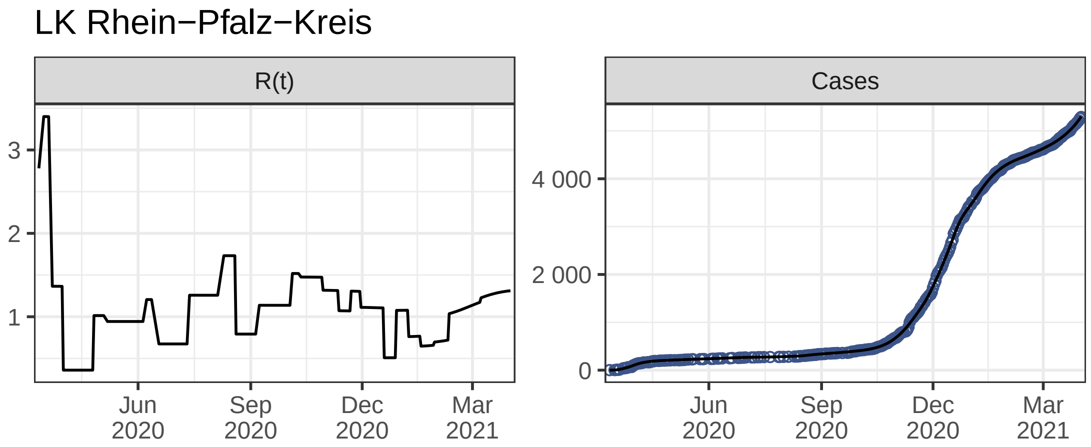

LK Rhein−Pfalz−Kreis
R(t)
Cases
3
2
1
4 000
2 000
0
Jun 2020
Sep 2020
Dec 2020
Mar 2021

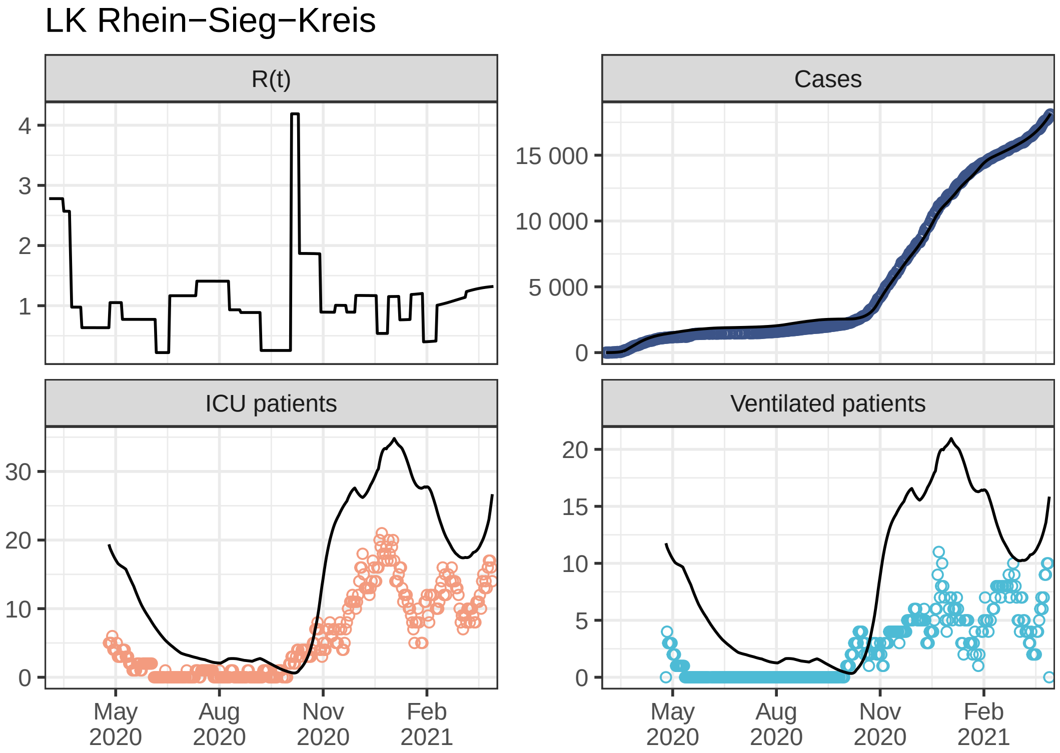
LK Rhein−Sieg−Kreis
R(t)
Cases
ICU patients
Ventilated patients
4
3
2
1
15 000
10 000
5 000
0
30
20
10
0
20
15
10
5
0
May 2020
Aug 2020
Nov 2020
Feb 2021

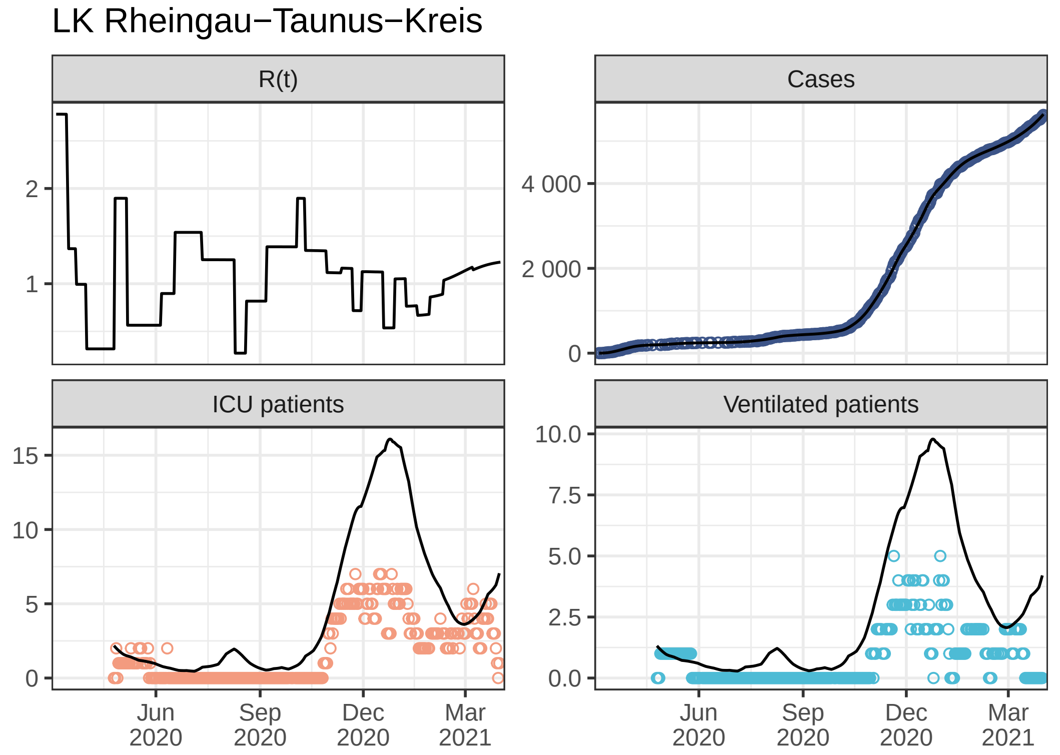
LK Rheingau−Taunus−Kreis
R(t)
Cases
ICU patients
Ventilated patients
2
1
4 000
2 000
0
15
10
5
0
10.0
7.5
5.0
2.5
0.0
Jun 2020
Sep 2020
Dec 2020
Mar 2021

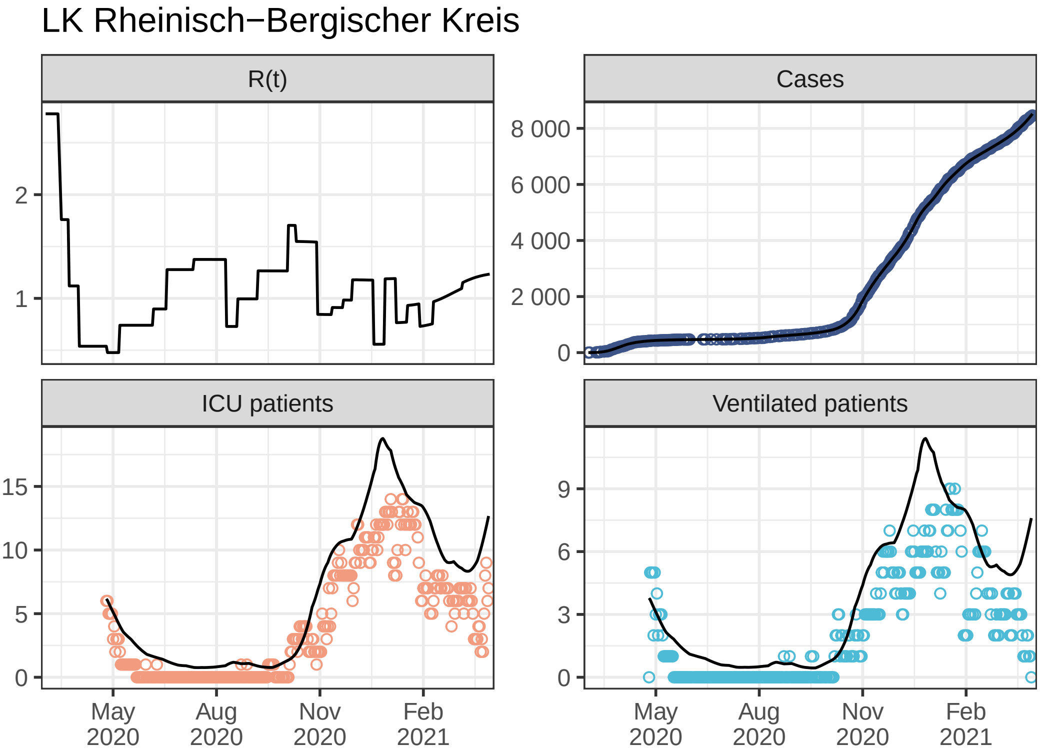
LK Rheinisch−Bergischer Kreis
R(t)
Cases
ICU patients
Ventilated patients
2
1
8 000
6 000
4 000
2 000
0
15
10
5
0
9
6
3
0
May 2020
Aug 2020
Nov 2020
Feb 2021

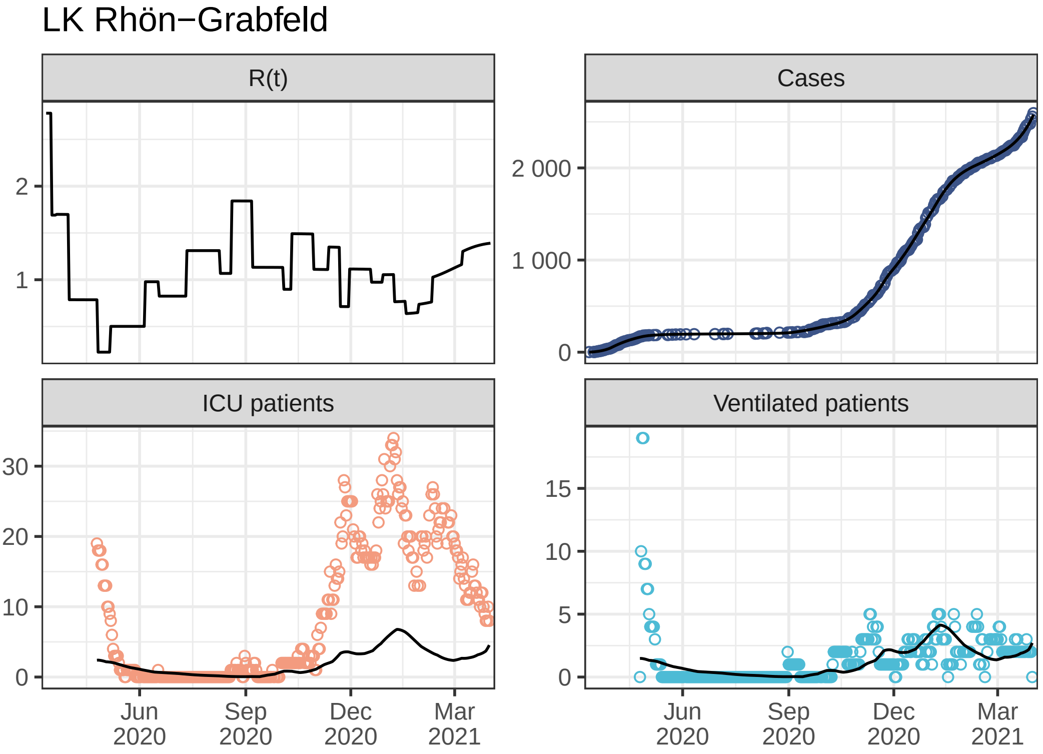
LK Rhön−Grabfeld
R(t)
Cases
ICU patients
Ventilated patients
2
1
2 000
1 000
0
30
20
10
0
15
10
5
0
Jun 2020
Sep 2020
Dec 2020
Mar 2021

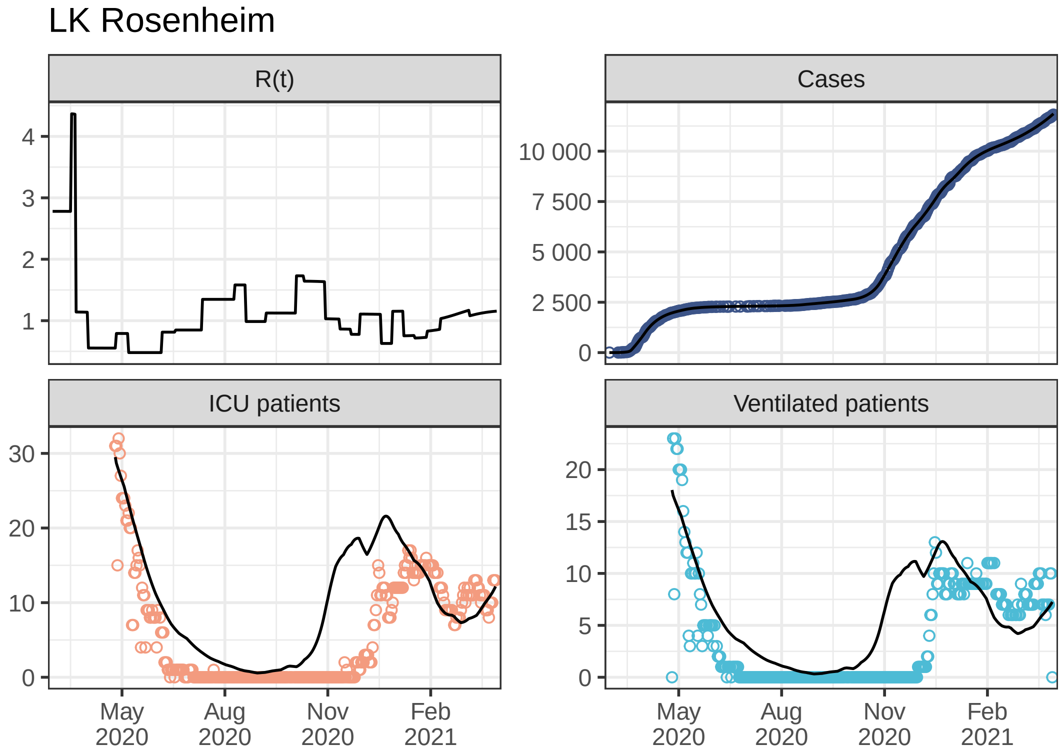
LK Rosenheim
R(t)
Cases
ICU patients
Ventilated patients
May 2020
Aug 2020
Nov 2020
Feb 2021
0
2 500
5 000
7 500
10 000

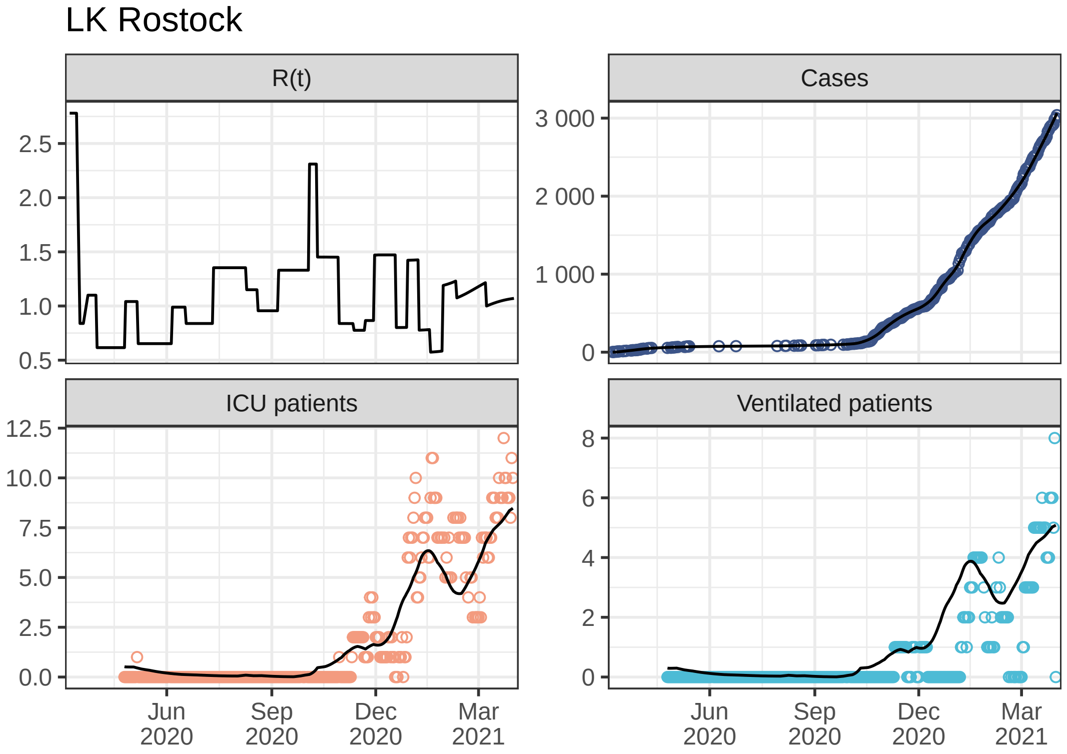
LK Rostock
R(t)
Cases
ICU patients
Ventilated patients
Jun 2020
Sep 2020
Dec 2020
Mar 2021
0
1 000
2 000
3 000

LK Rotenburg (Wümme)

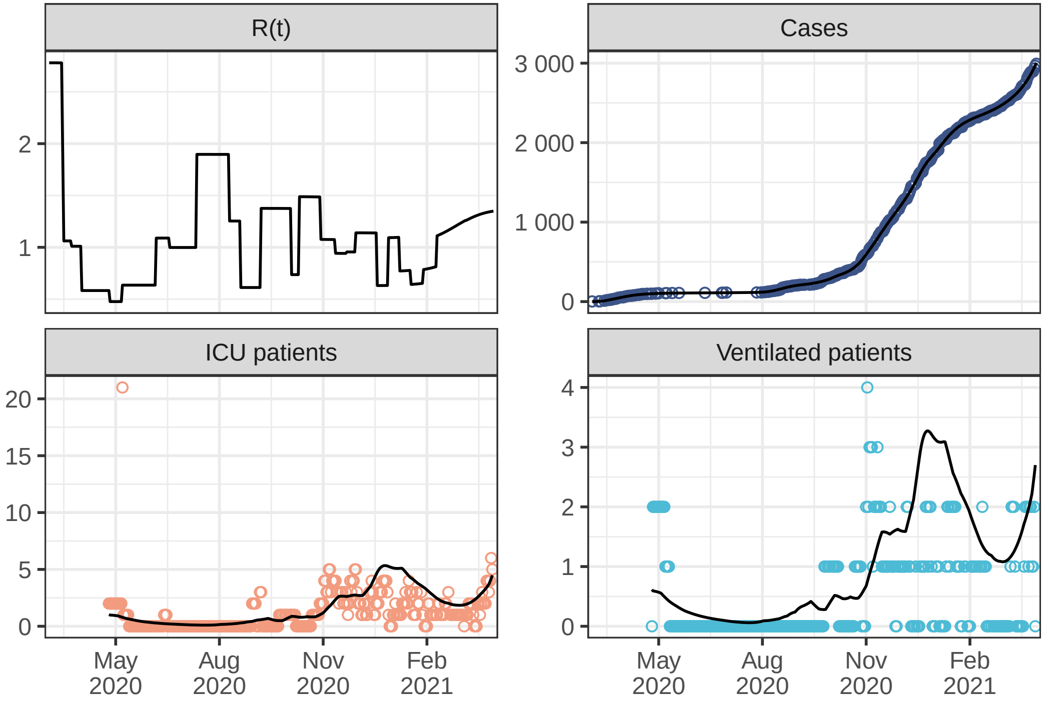

LK Roth

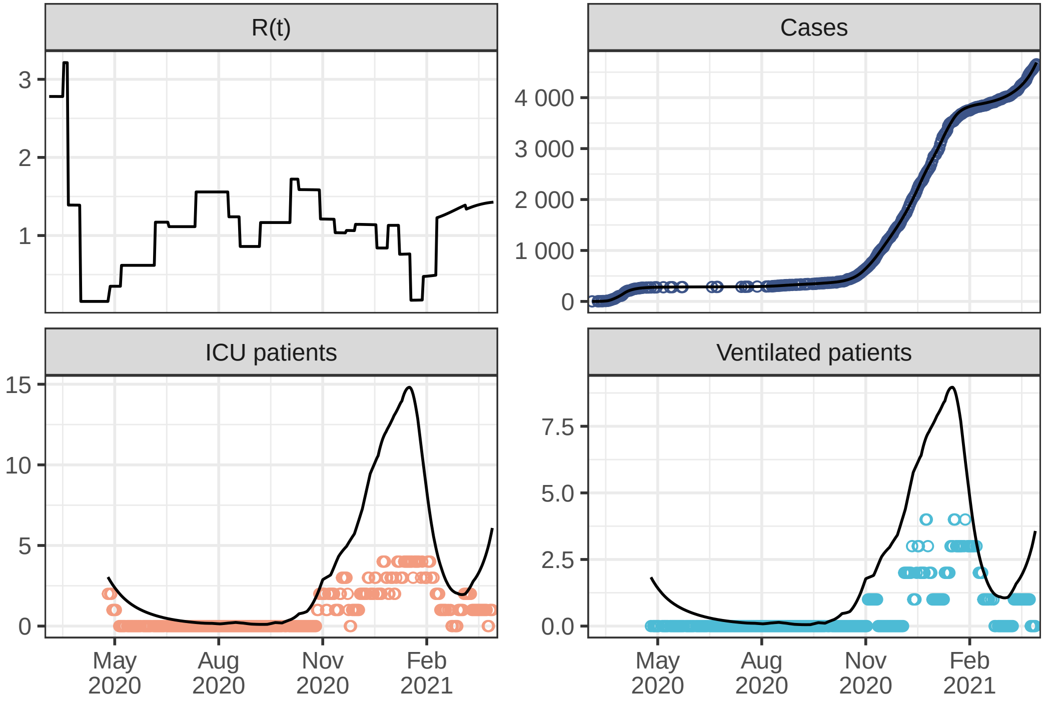

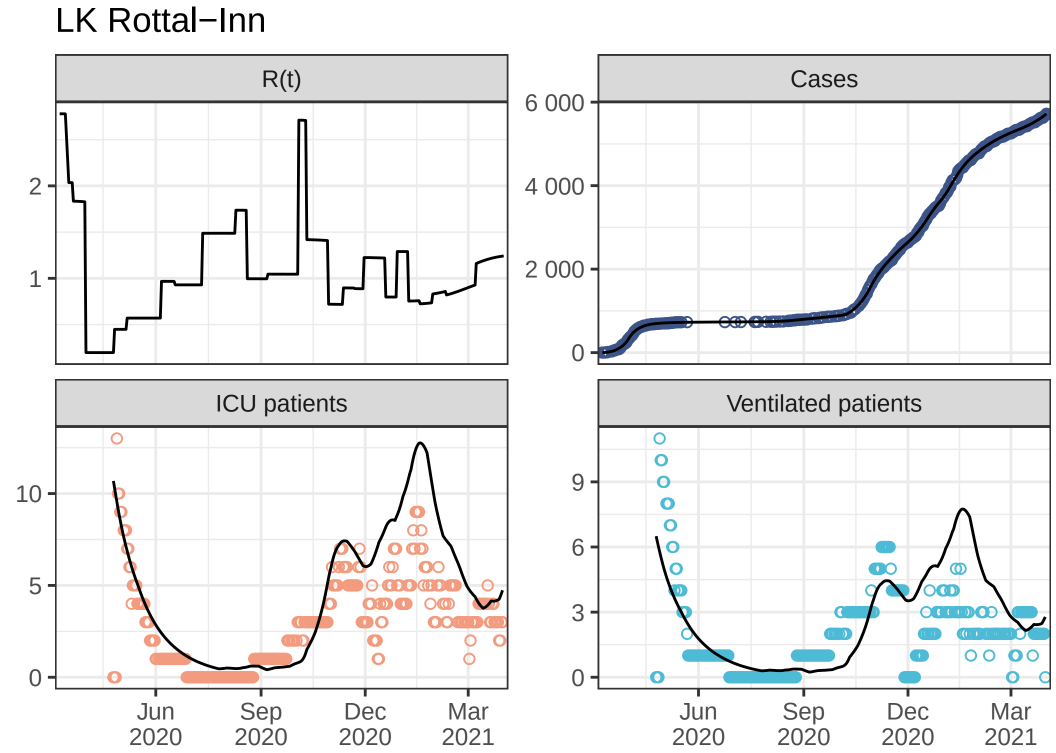
LK Rottal−Inn
R(t)
Cases
ICU patients
Ventilated patients
2
1
6 000
4 000
2 000
0
10
5
0
9
6
3
0
Jun 2020
Sep 2020
Dec 2020
Mar 2021

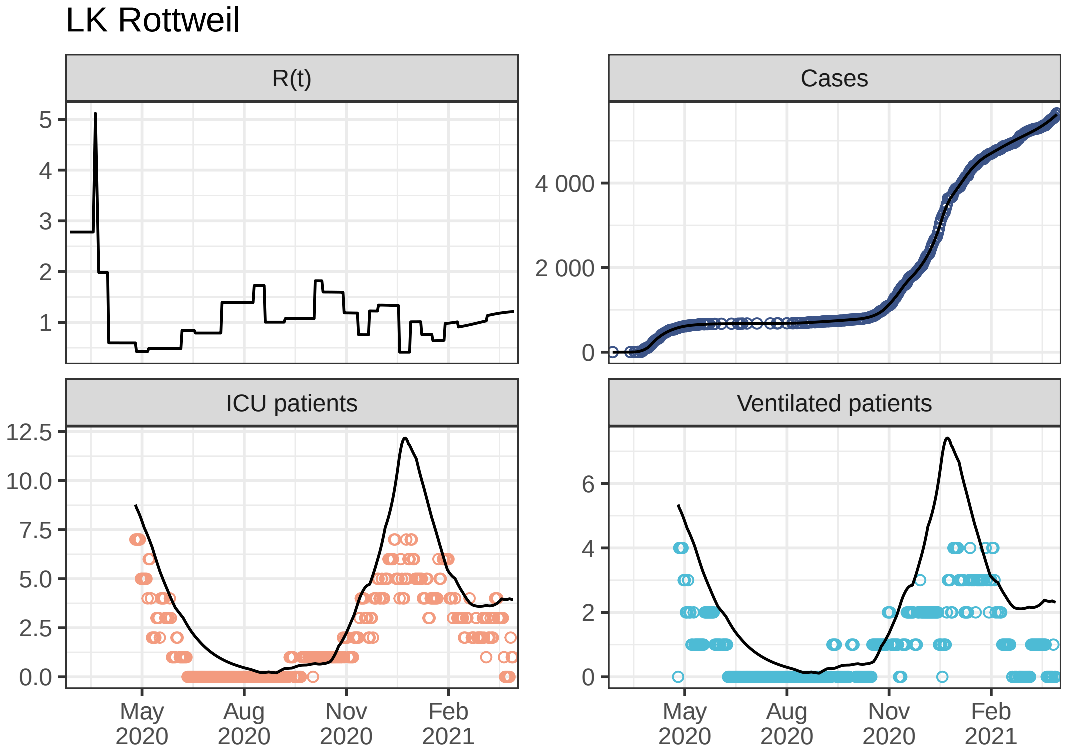
LK Rottweil
R(t)
Cases
ICU patients
Ventilated patients
5
4
3
2
1
4 000
2 000
0
12.5
10.0
7.5
5.0
2.5
0.0
6
4
2
0
May 2020
Aug 2020
Nov 2020
Feb 2021

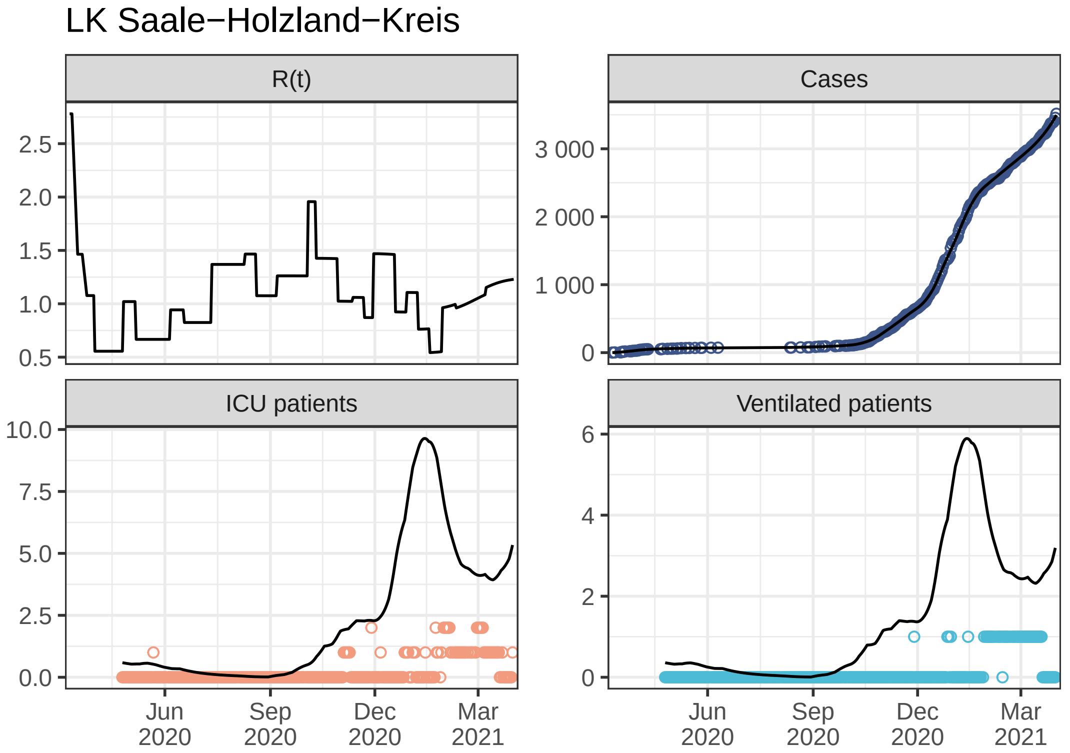
LK Saale−Holzland−Kreis
R(t)
Cases
ICU patients
Ventilated patients
2.5
2.0
1.5
1.0
0.5
3 000
2 000
1 000
0
10.0
7.5
5.0
2.5
0.0
6
4
2
0
Jun 2020
Sep 2020
Dec 2020
Mar 2021

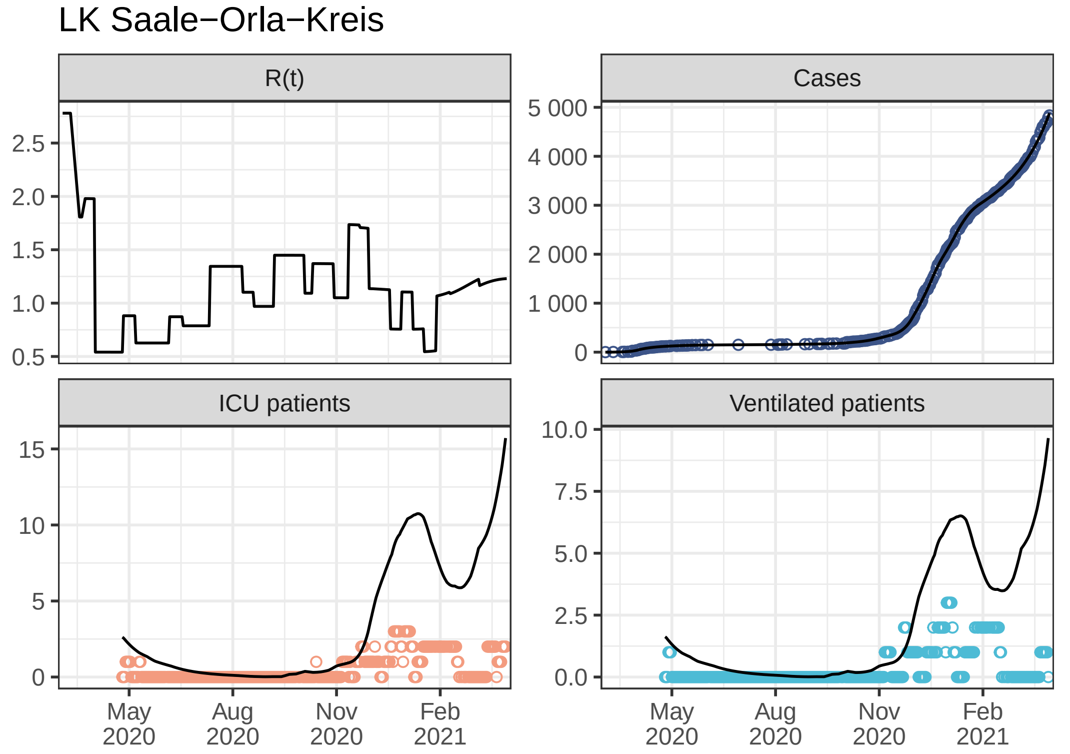
LK Saale−Orla−Kreis
R(t)
Cases
ICU patients
Ventilated patients
2.5
2.0
1.5
1.0
0.5
5 000
4 000
3 000
2 000
1 000
0
15
10
5
0
10.0
7.5
5.0
2.5
0.0
May 2020
Aug 2020
Nov 2020
Feb 2021

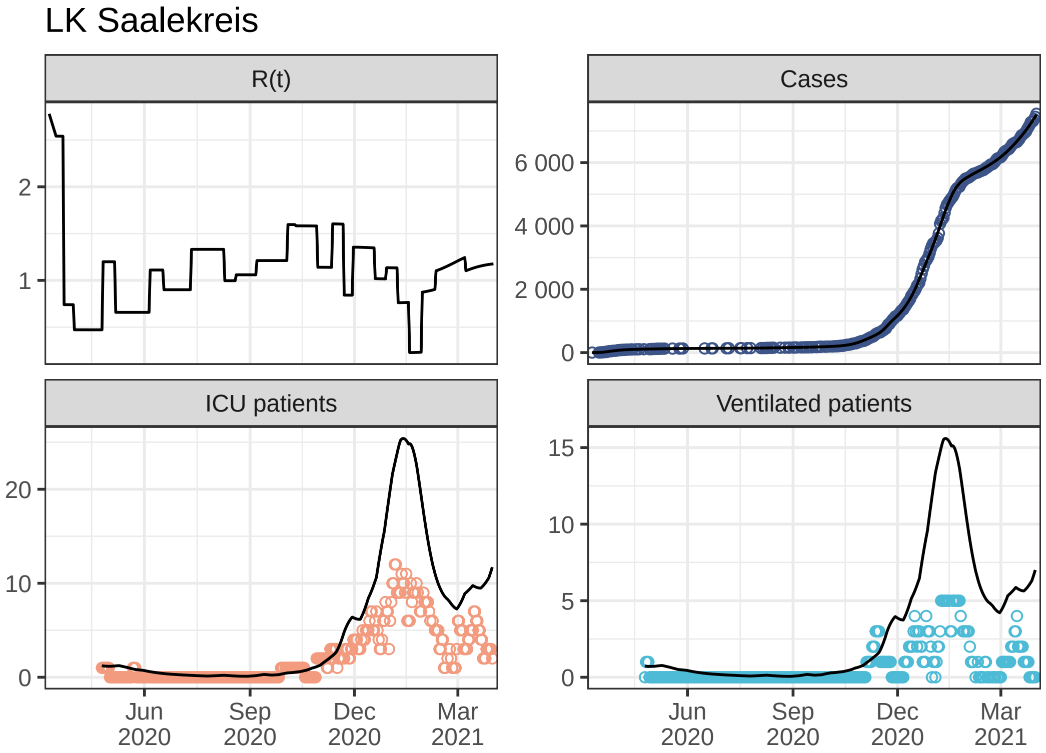
LK Saalekreis
R(t)
Cases
ICU patients
Ventilated patients
2
1
6 000
4 000
2 000
0
20
10
0
15
10
5
0
Jun 2020
Sep 2020
Dec 2020
Mar 2021

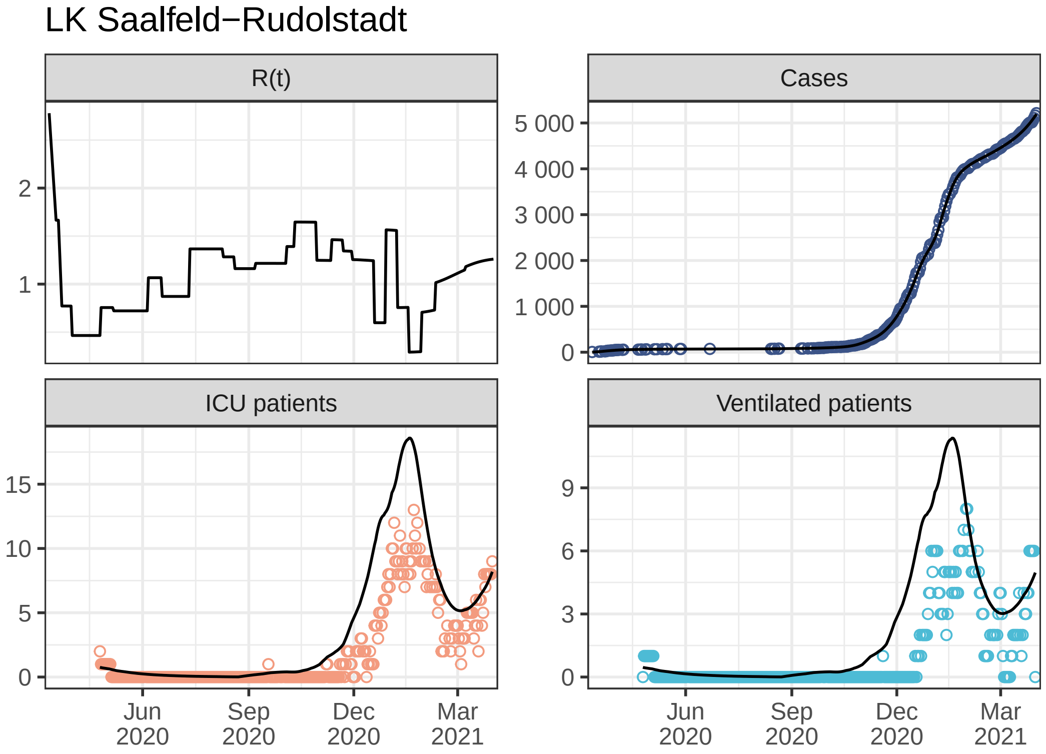
LK Saalfeld−Rudolstadt
R(t)
Cases
ICU patients
Ventilated patients
2
1
5 000
4 000
3 000
2 000
1 000
0
15
10
5
0
9
6
3
0
Jun 2020
Sep 2020
Dec 2020
Mar 2021

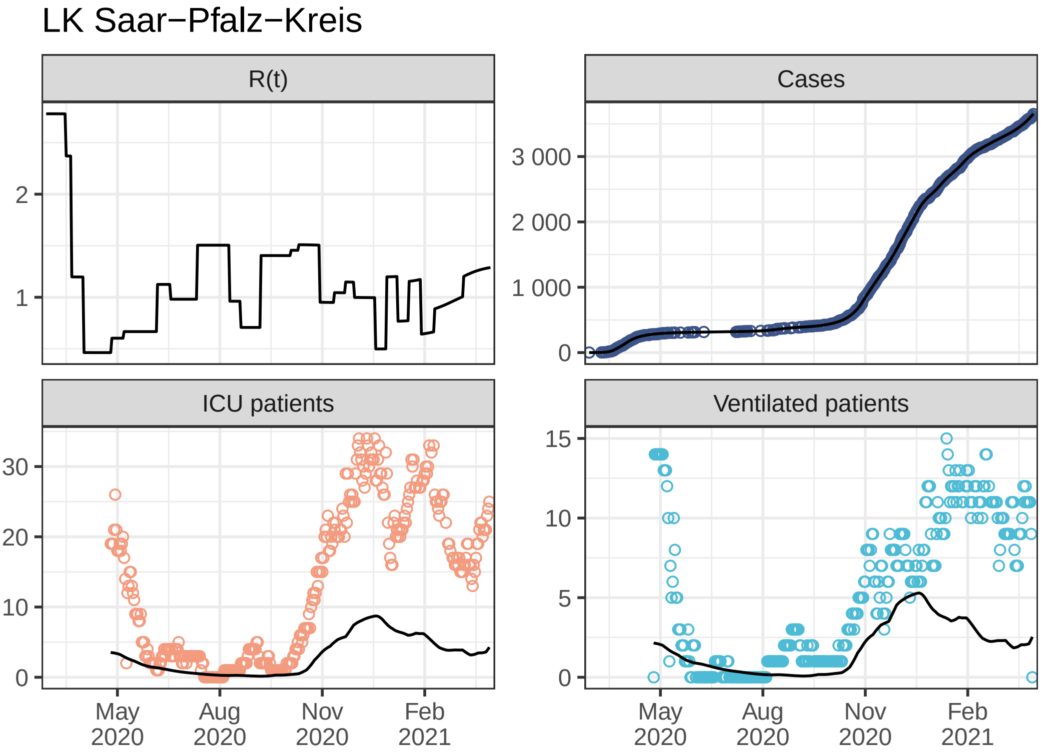
LK Saar−Pfalz−Kreis
R(t)
Cases
ICU patients
Ventilated patients
2
1
3 000
2 000
1 000
0
30
20
10
0
15
10
5
0
May 2020
Aug 2020
Nov 2020
Feb 2021

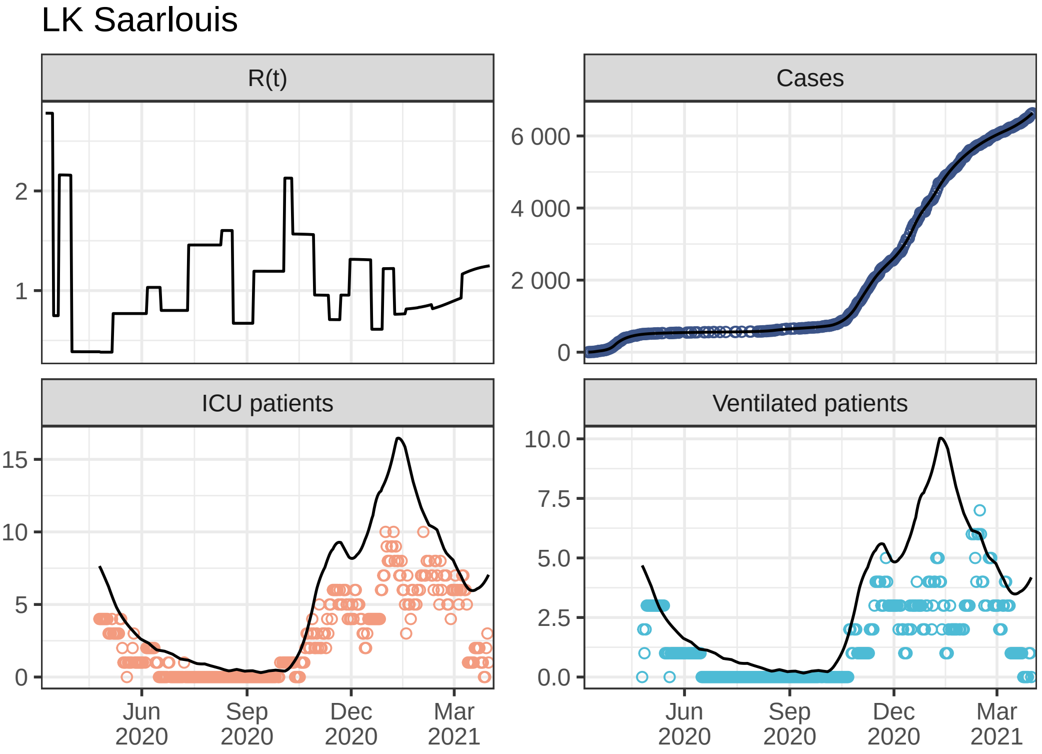
LK Saarlouis
R(t)
Cases
ICU patients
Ventilated patients
2
1
6 000
4 000
2 000
0
15
10
5
0
10.0
7.5
5.0
2.5
0.0
Jun 2020
Sep 2020
Dec 2020
Mar 2021

LK Sächsische Schweiz−Osterzgebirge

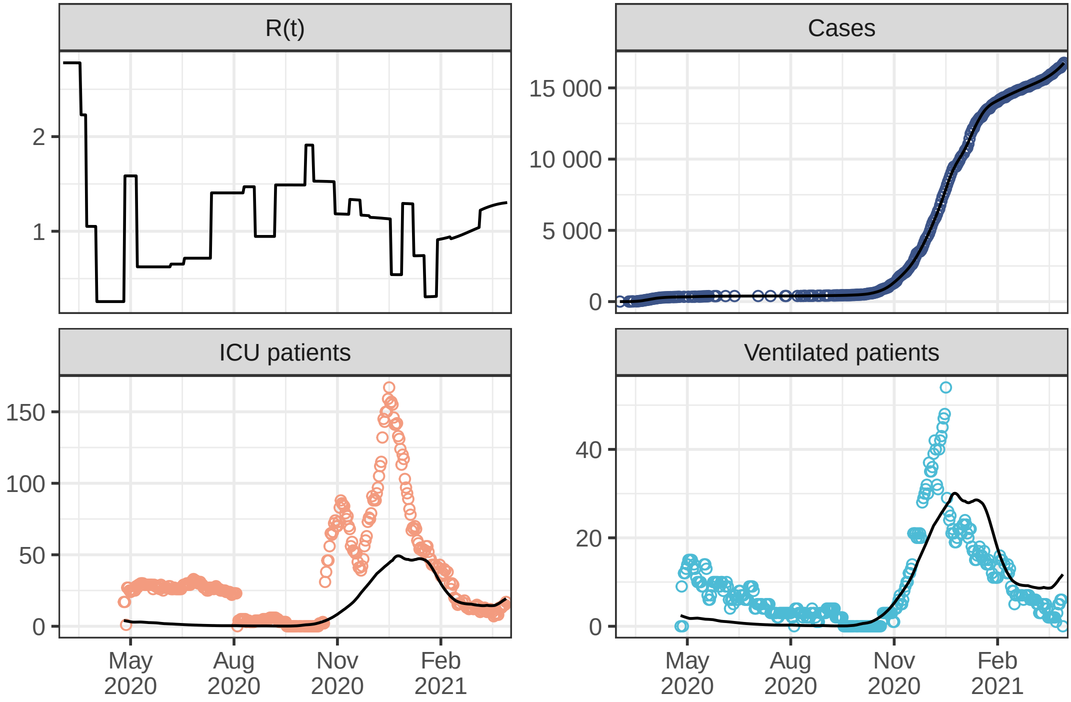

LK Salzlandkreis

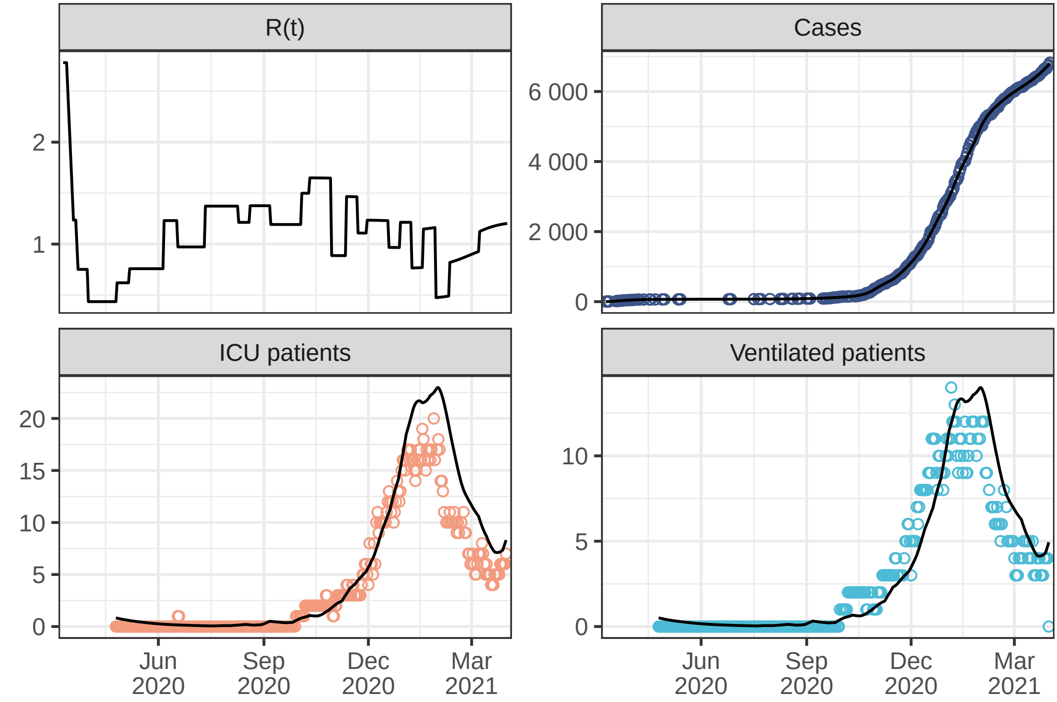

LK Sankt Wendel

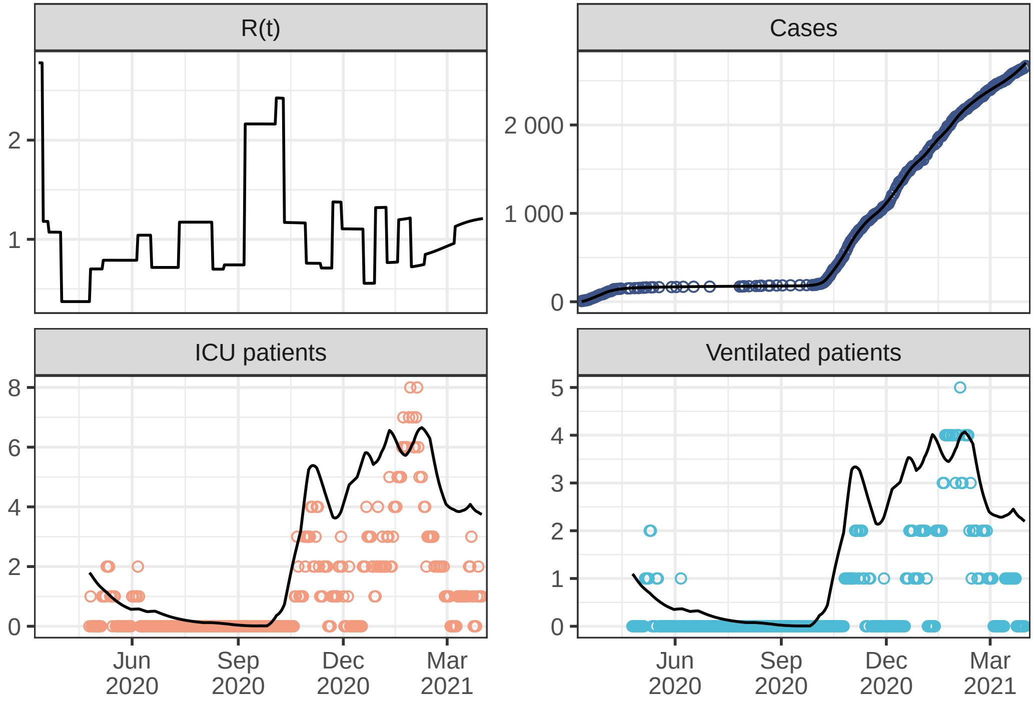

LK Schaumburg

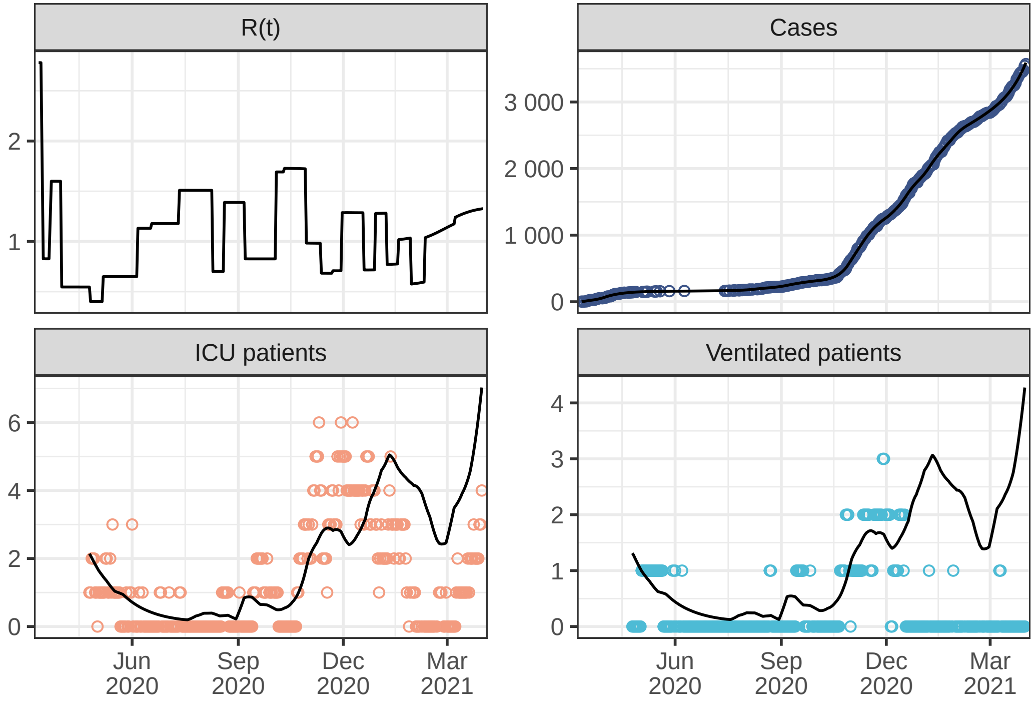

LK Schleswig−Flensburg

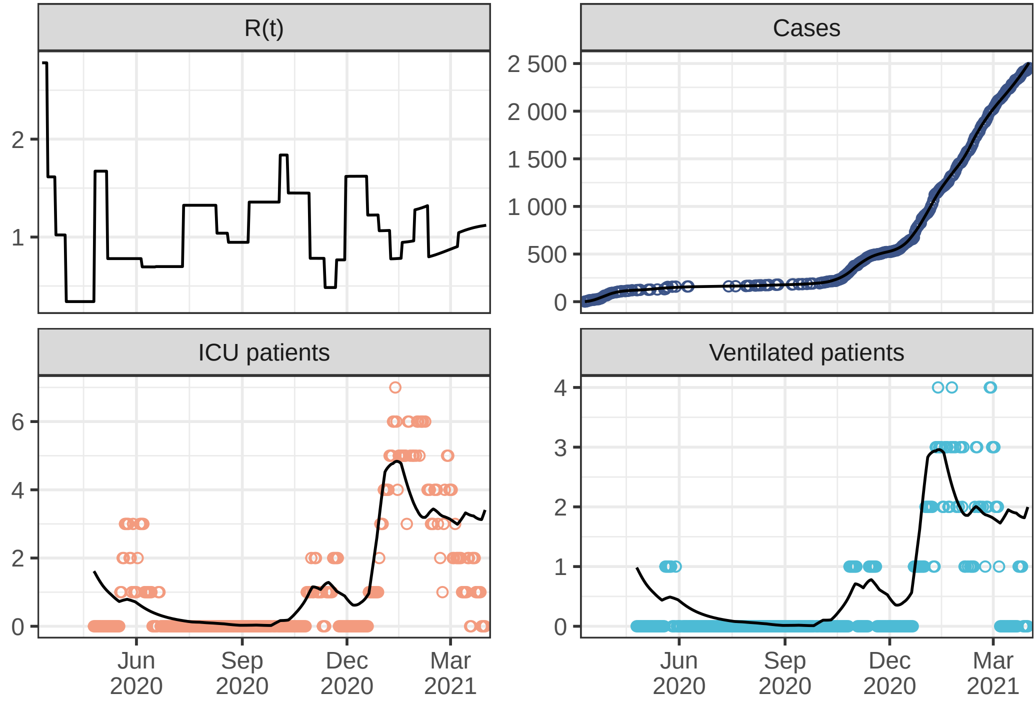

LK Schmalkalden−Meiningen

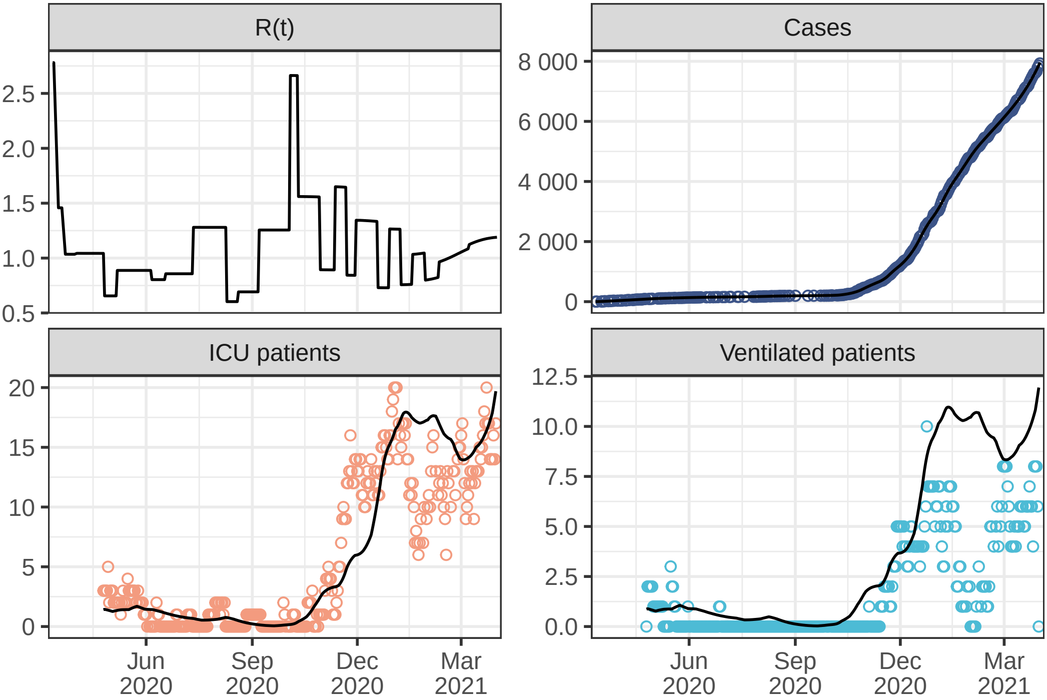

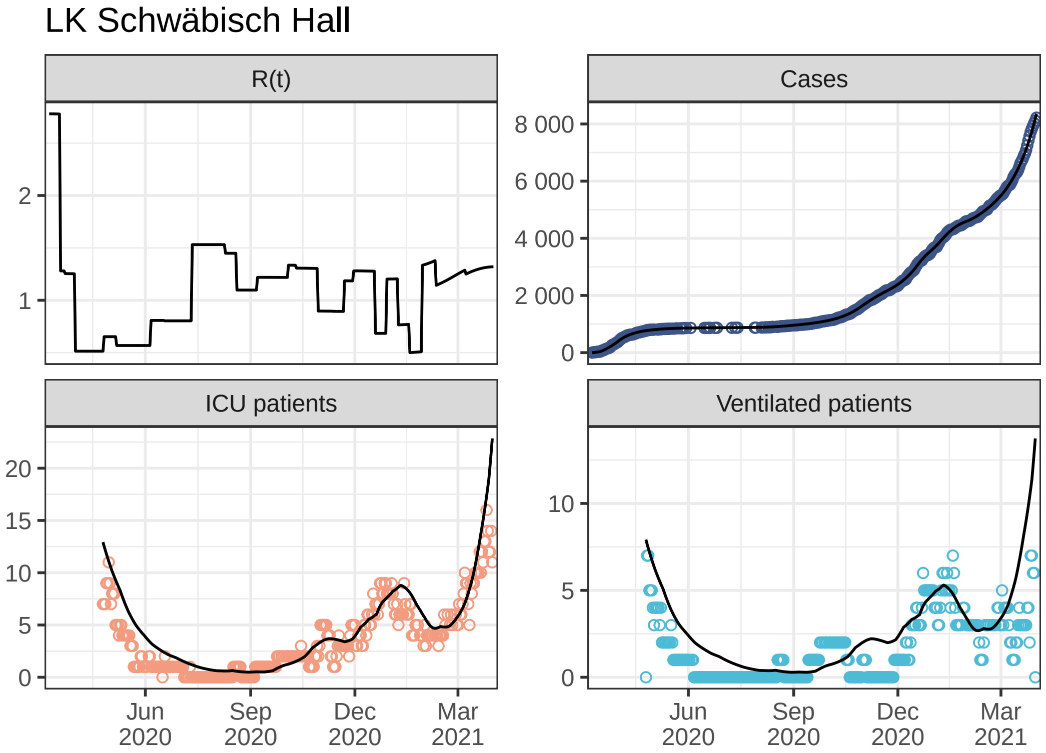
LK Schwäbisch Hall
R(t)
Cases
ICU patients
Ventilated patients
2
1
8 000
6 000
4 000
2 000
0
20
15
10
5
0
10
5
0
Jun 2020
Sep 2020
Dec 2020
Mar 2021

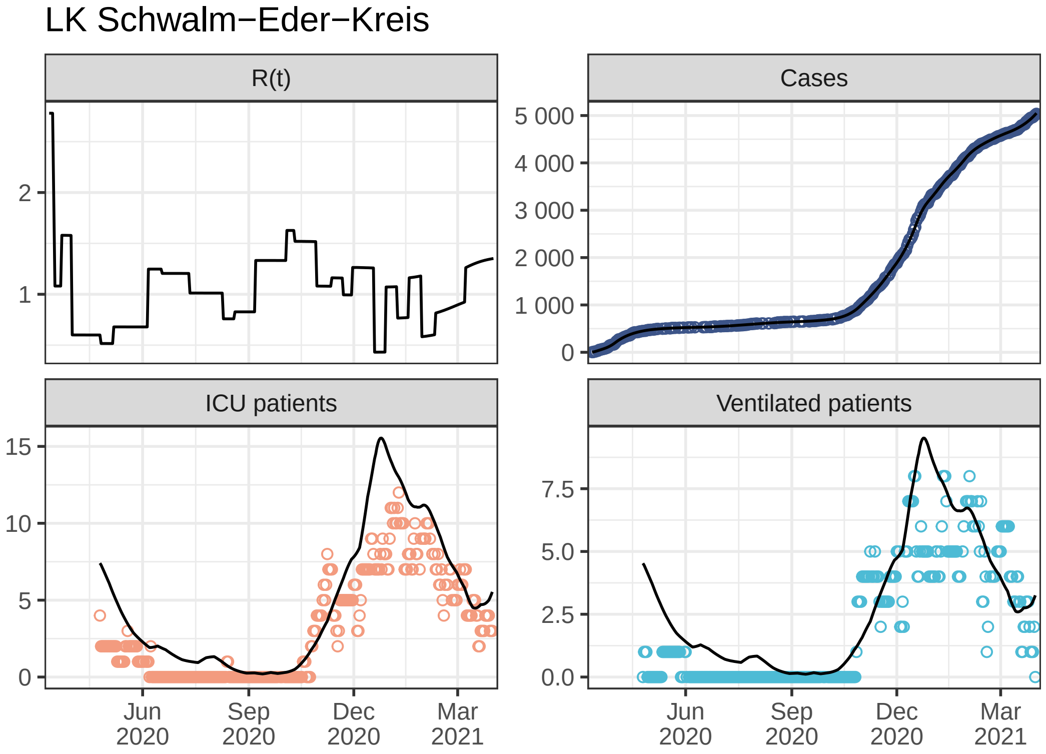
LK Schwalm−Eder−Kreis
R(t)
Cases
ICU patients
Ventilated patients
2
1
5 000
4 000
3 000
2 000
1 000
0
15
10
5
0
7.5
5.0
2.5
0.0
Jun 2020
Sep 2020
Dec 2020
Mar 2021

LK Schwandorf

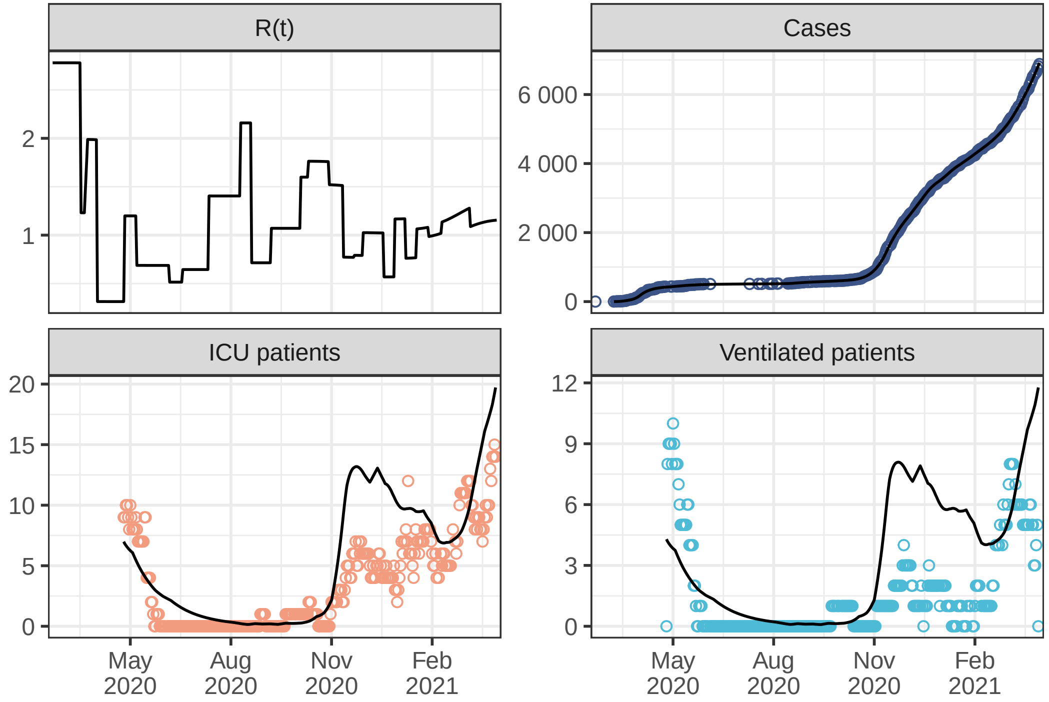

LK Schwarzwald−Baar−Kreis

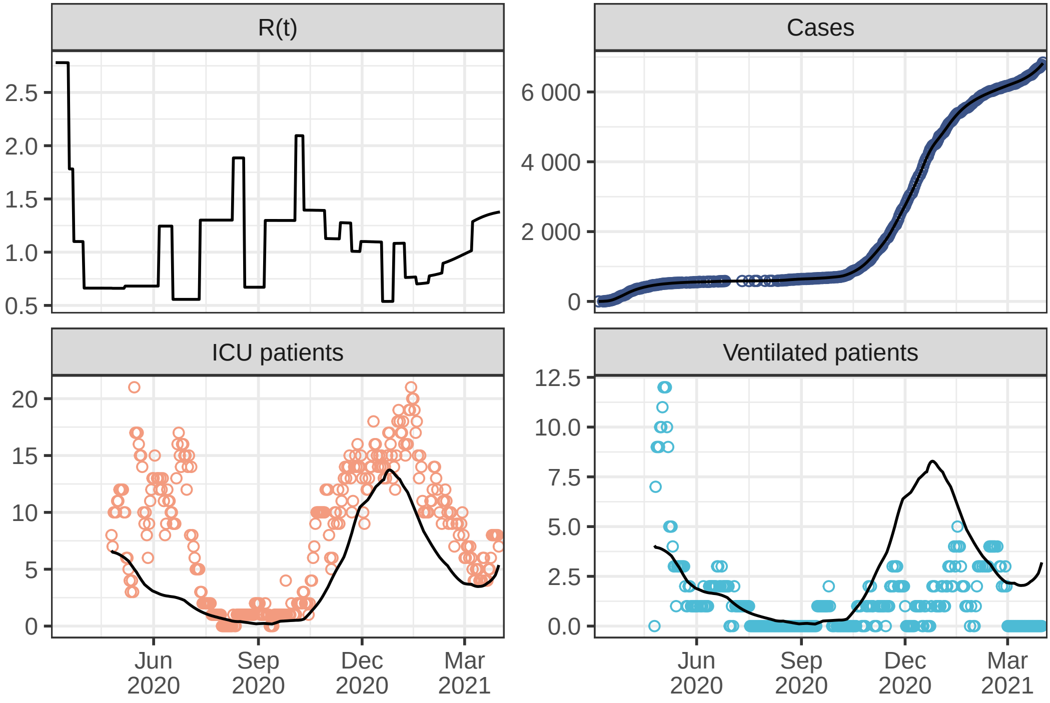

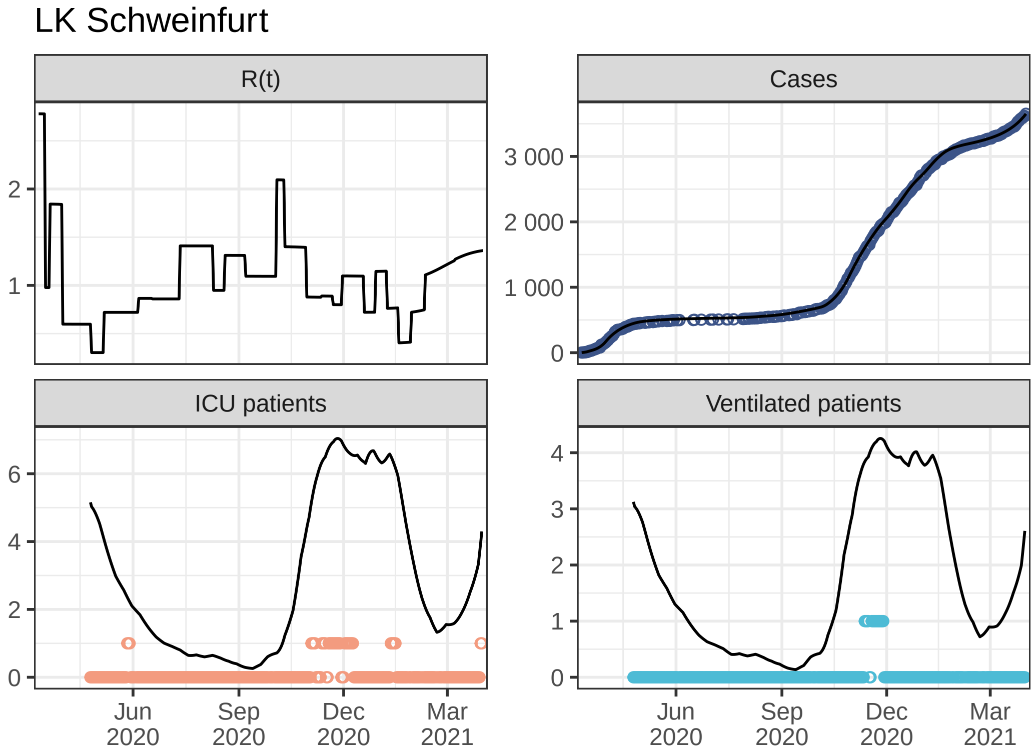
LK Schweinfurt
R(t)
Cases
ICU patients
Ventilated patients
2
1
3 000
2 000
1 000
0
6
4
2
0
4
3
2
1
0
Jun 2020
Sep 2020
Dec 2020
Mar 2021

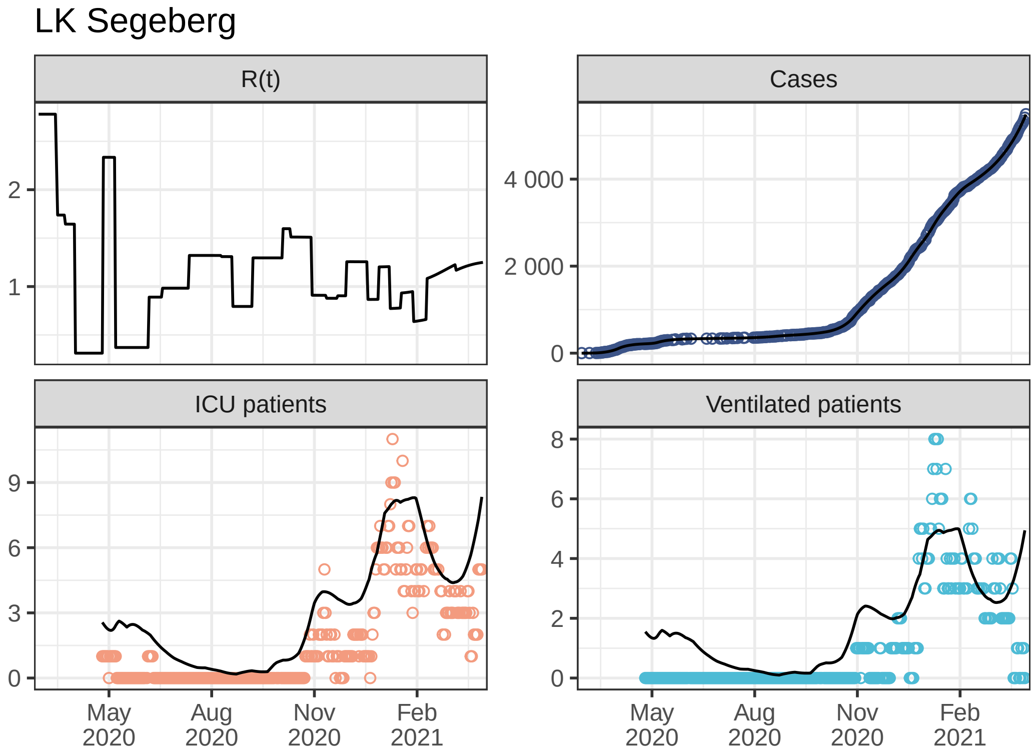
LK Segeberg
R(t)
Cases
ICU patients
Ventilated patients
2
1
4 000
2 000
0
9
6
3
0
8
6
4
2
0
May 2020
Aug 2020
Nov 2020
Feb 2021

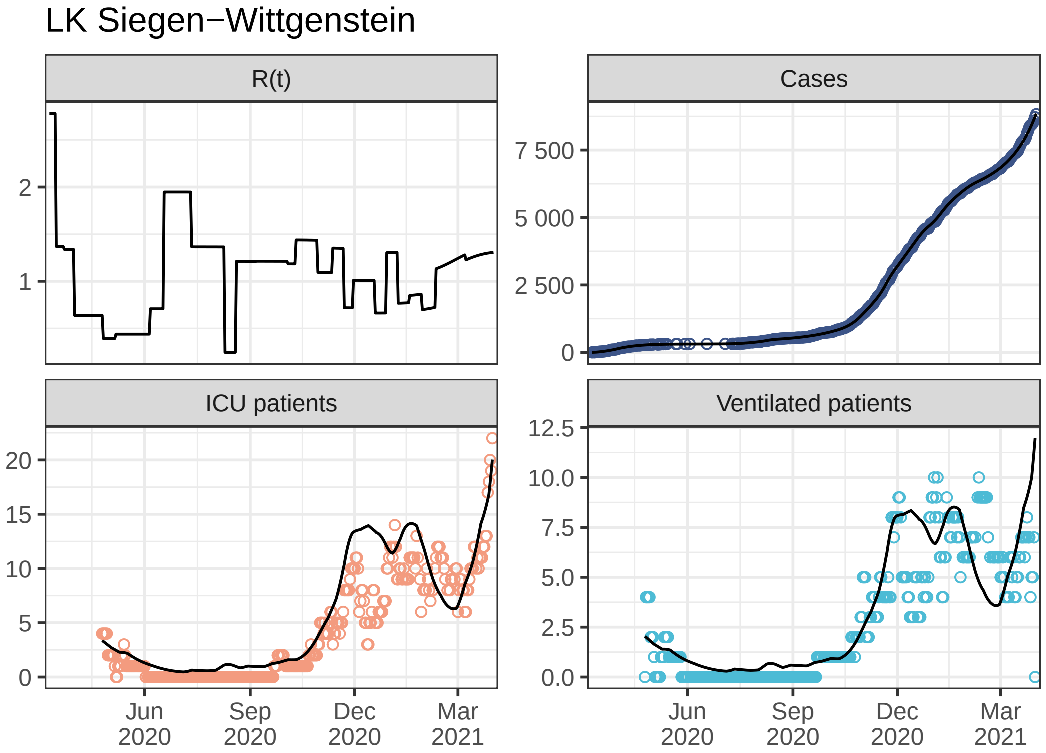
LK Siegen−Wittgenstein
R(t)
Cases
ICU patients
Ventilated patients
2
1
7 500
5 000
2 500
0
20
15
10
5
0
12.5
10.0
7.5
5.0
2.5
0.0
Jun 2020
Sep 2020
Dec 2020
Mar 2021

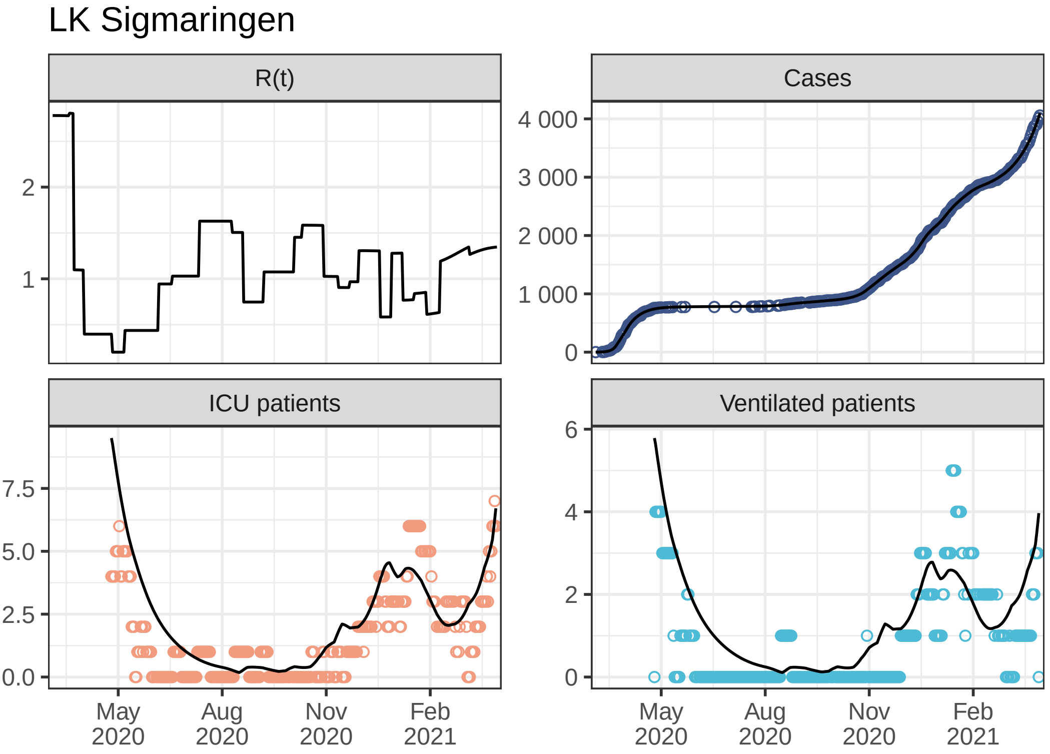
LK Sigmaringen
R(t)
Cases
ICU patients
Ventilated patients
2
1
4 000
3 000
2 000
1 000
0
7.5
5.0
2.5
0.0
6
4
2
0
May 2020
Aug 2020
Nov 2020
Feb 2021

LK Soest

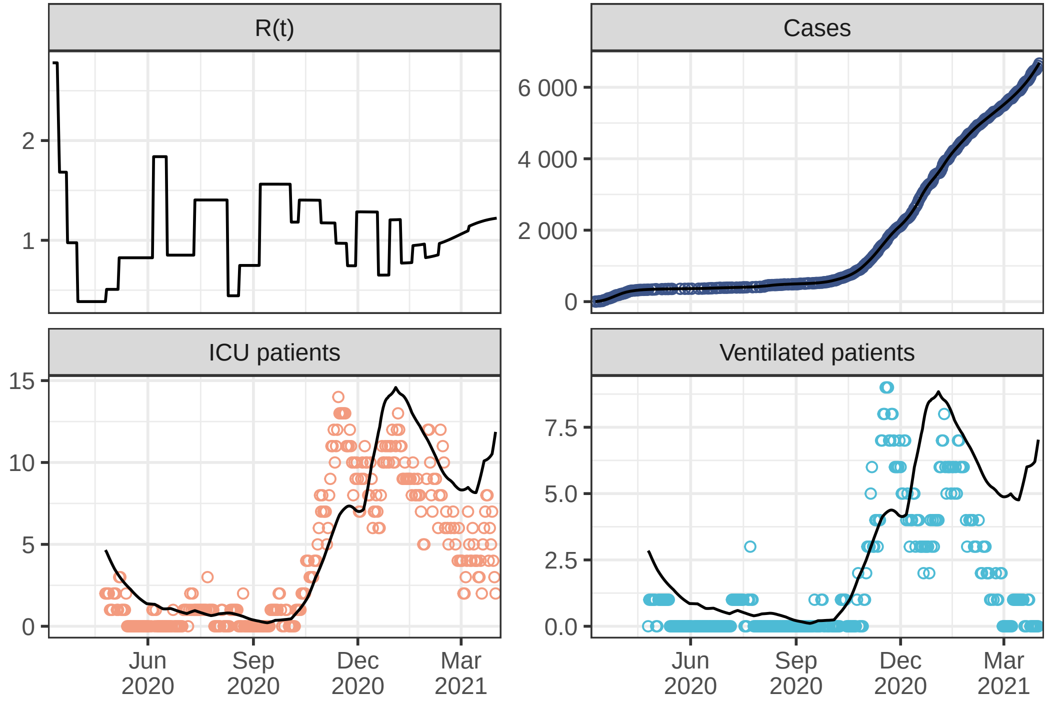

LK Sömmerda

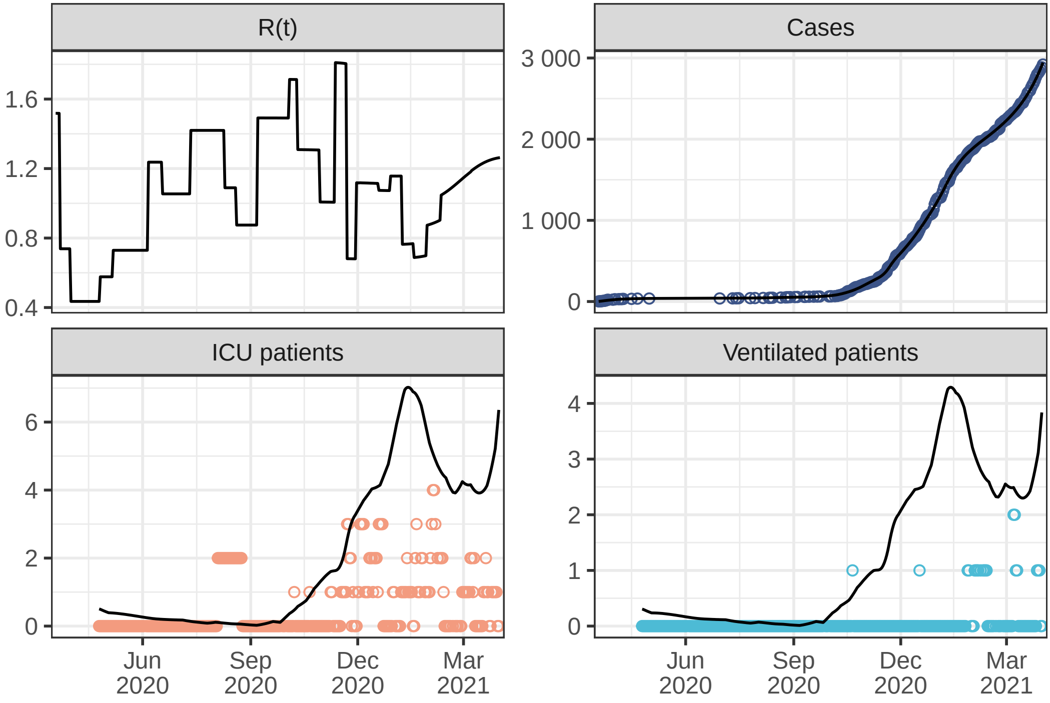

LK Sonneberg

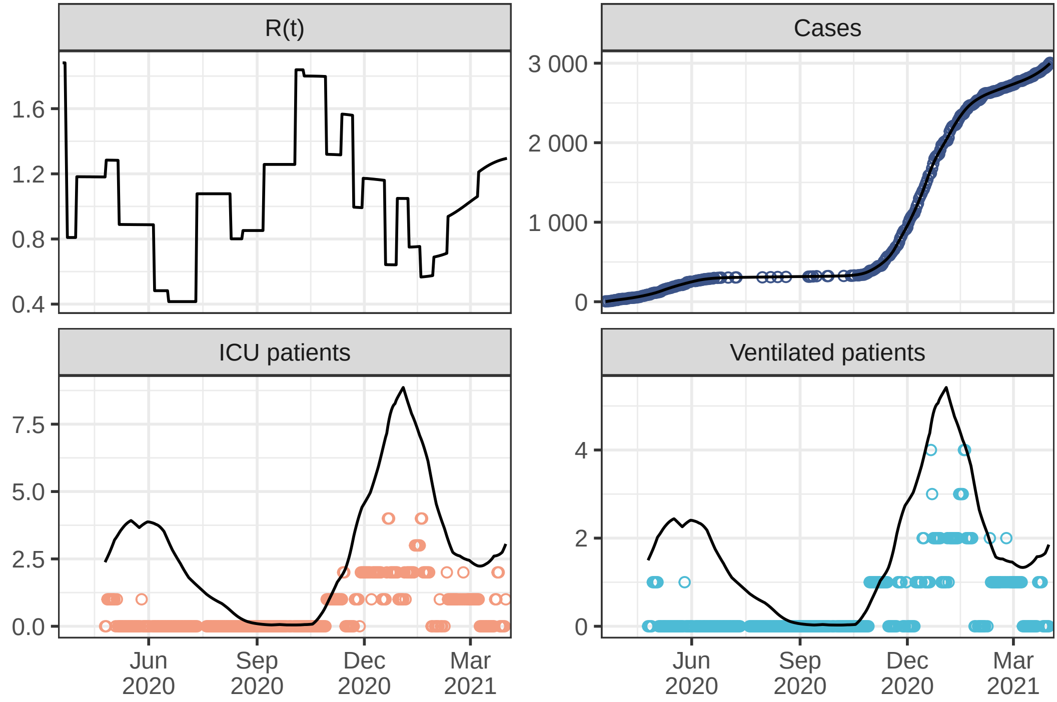

LK Spree−Neiße

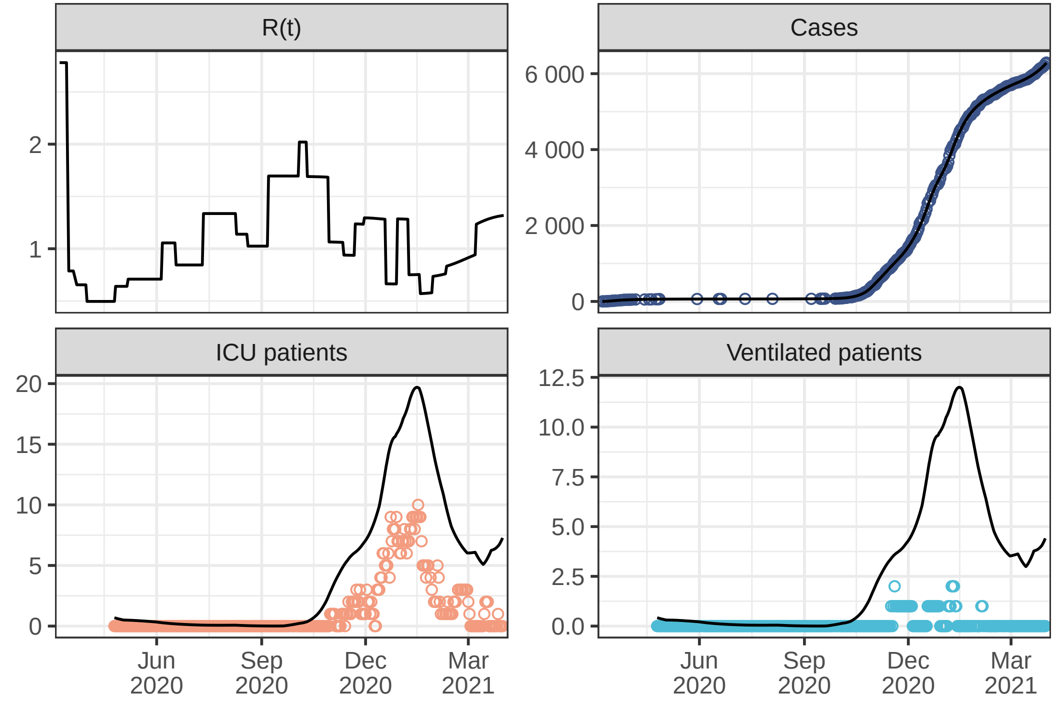

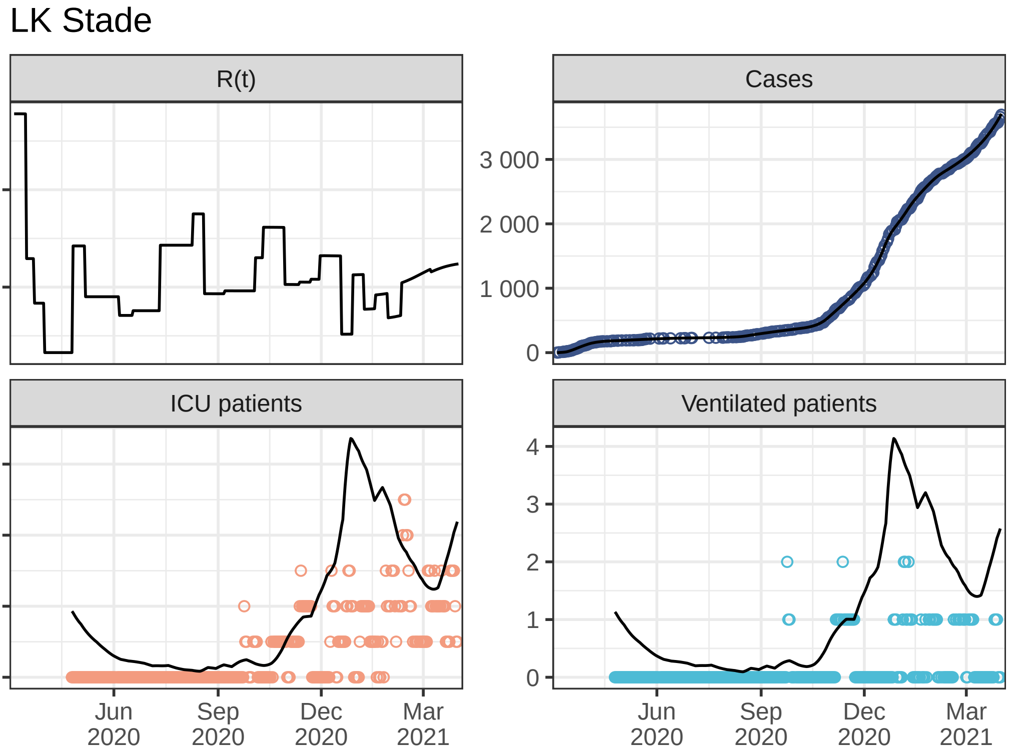
LK Stade
R(t)
Cases
ICU patients
Ventilated patients
2
1
3 000
2 000
1 000
0
6
4
2
0
4
3
2
1
0
Jun 2020
Sep 2020
Dec 2020
Mar 2021

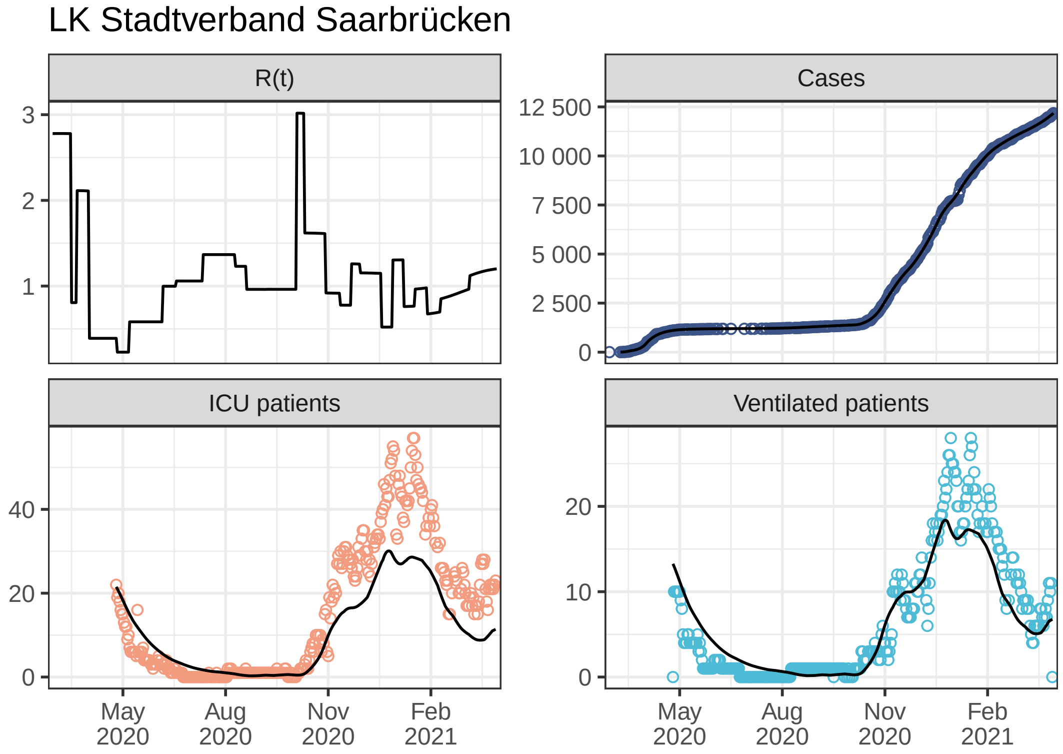
LK Stadtverband Saarbrücken
R(t)
Cases
ICU patients
Ventilated patients
3
2
1
12 500
10 000
7 500
5 000
2 500
0
40
20
0
20
10
0
May 2020
Aug 2020
Nov 2020
Feb 2021

LK Starnberg

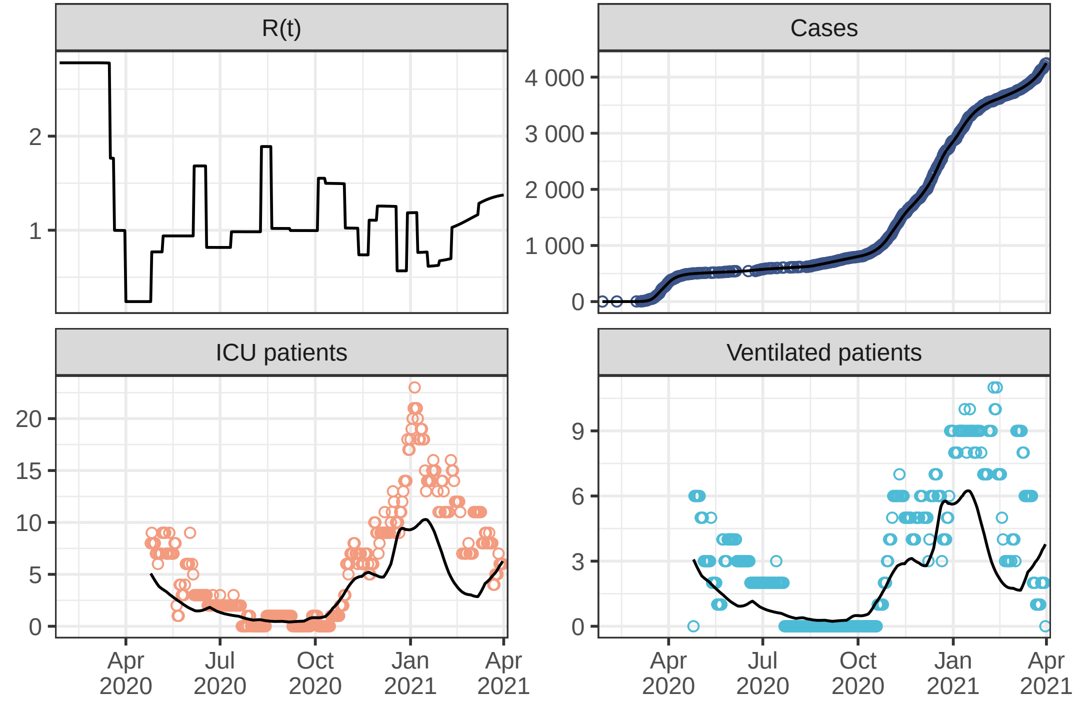

LK Steinburg

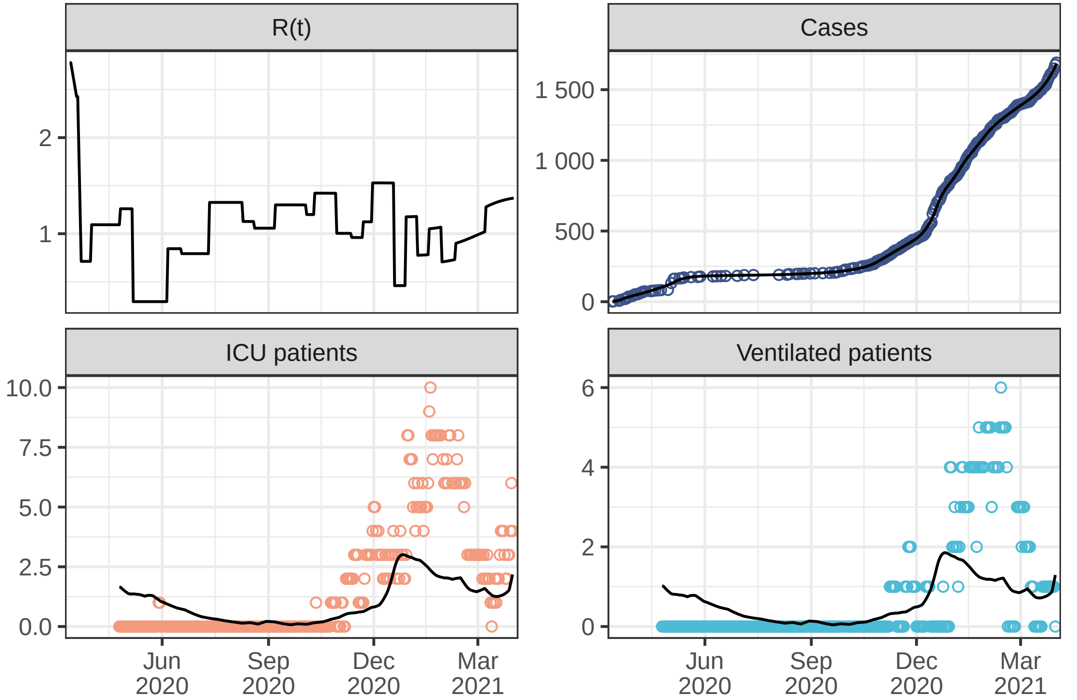

LK Steinfurt

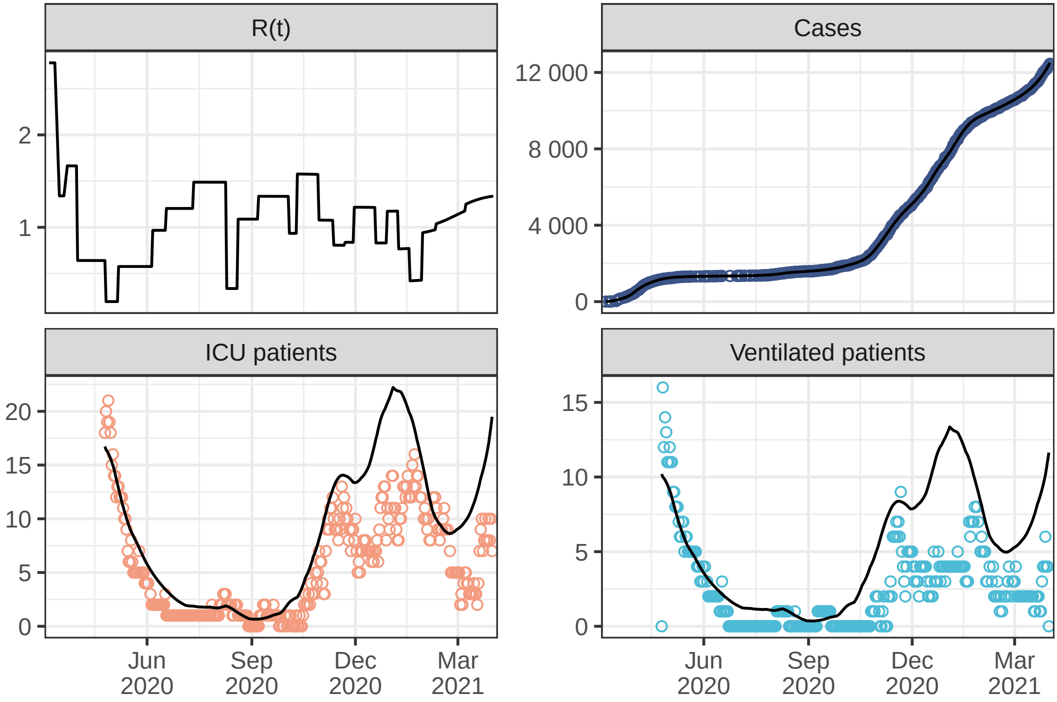

LK Stendal

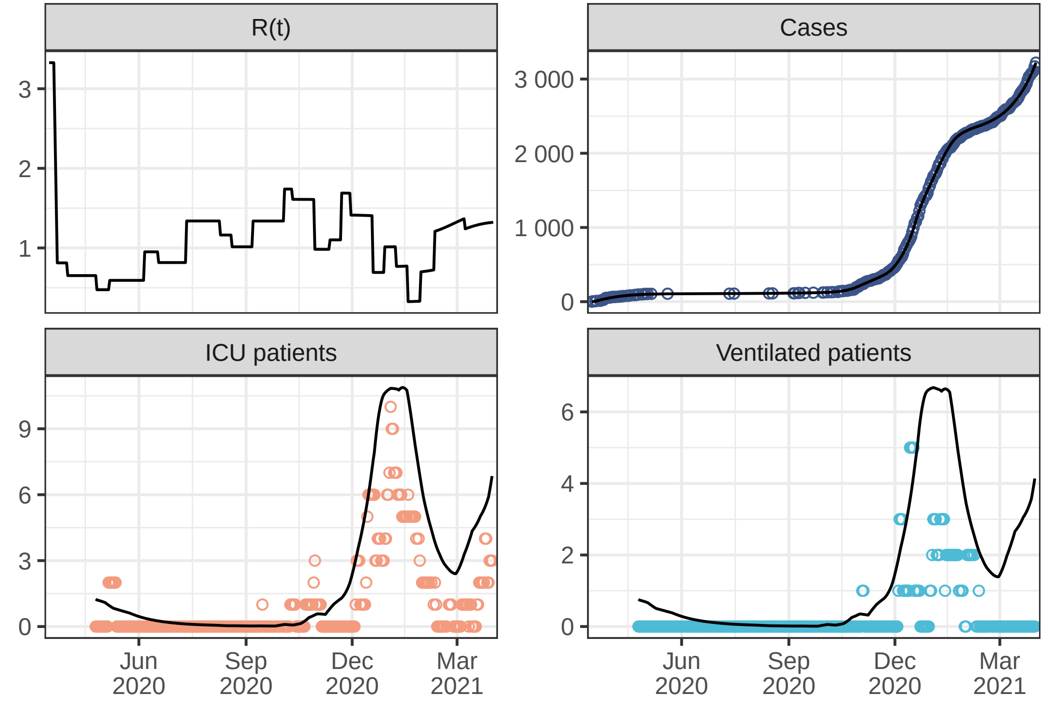

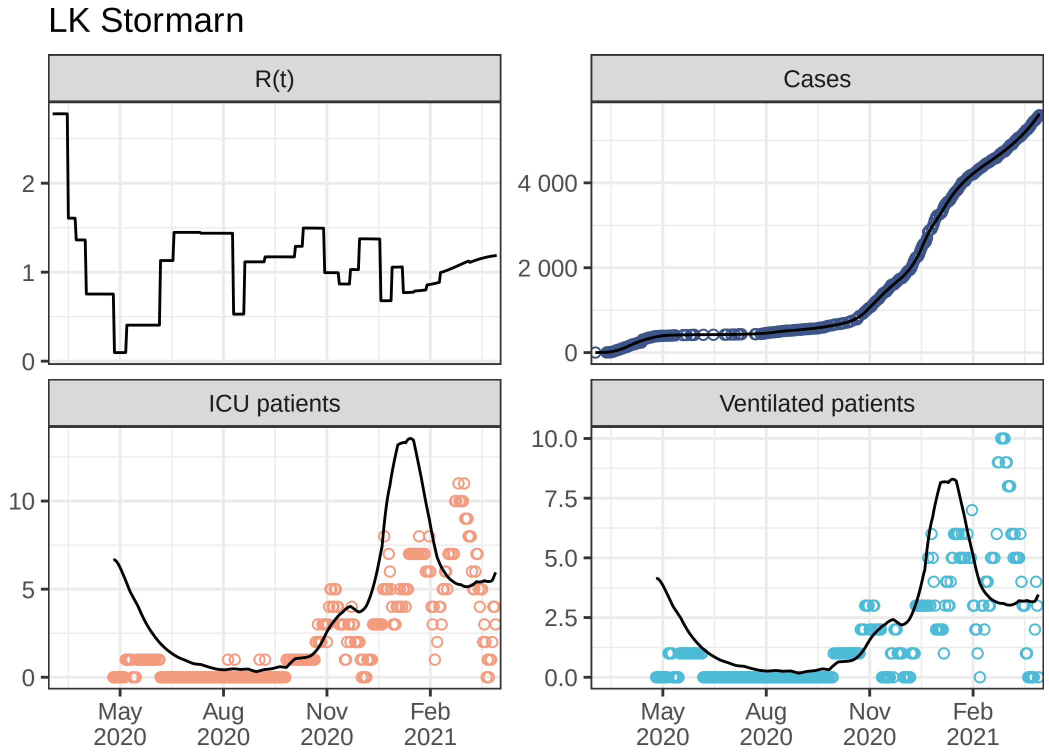

LK Stormarn
R(t)
Cases
ICU patients
Ventilated patients
2
1
0
4 000
2 000
0
10
5
0
10.0
7.5
5.0
2.5
0.0
May 2020
Aug 2020
Nov 2020
Feb 2021

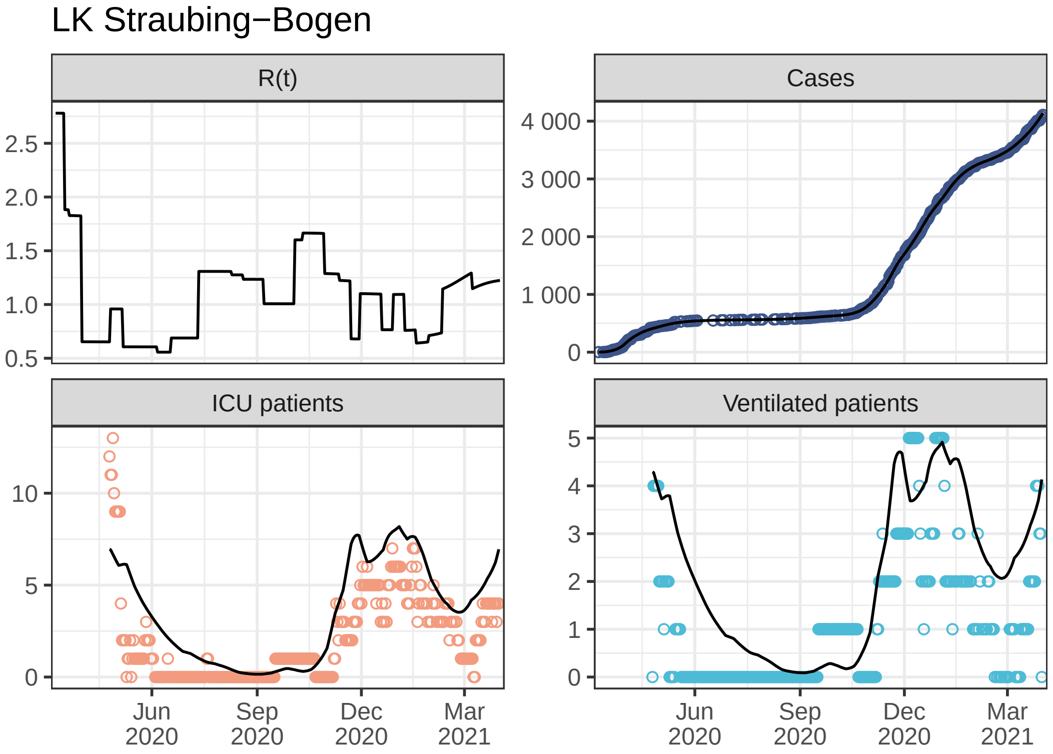

LK Straubing−Bogen
R(t)
Cases
ICU patients
Ventilated patients
2.5
2.0
1.5
1.0
0.5
4 000
3 000
2 000
1 000
0
10
5
0
5
4
3
2
1
0
Jun 2020
Sep 2020
Dec 2020
Mar 2021

LK Südliche Weinstraße

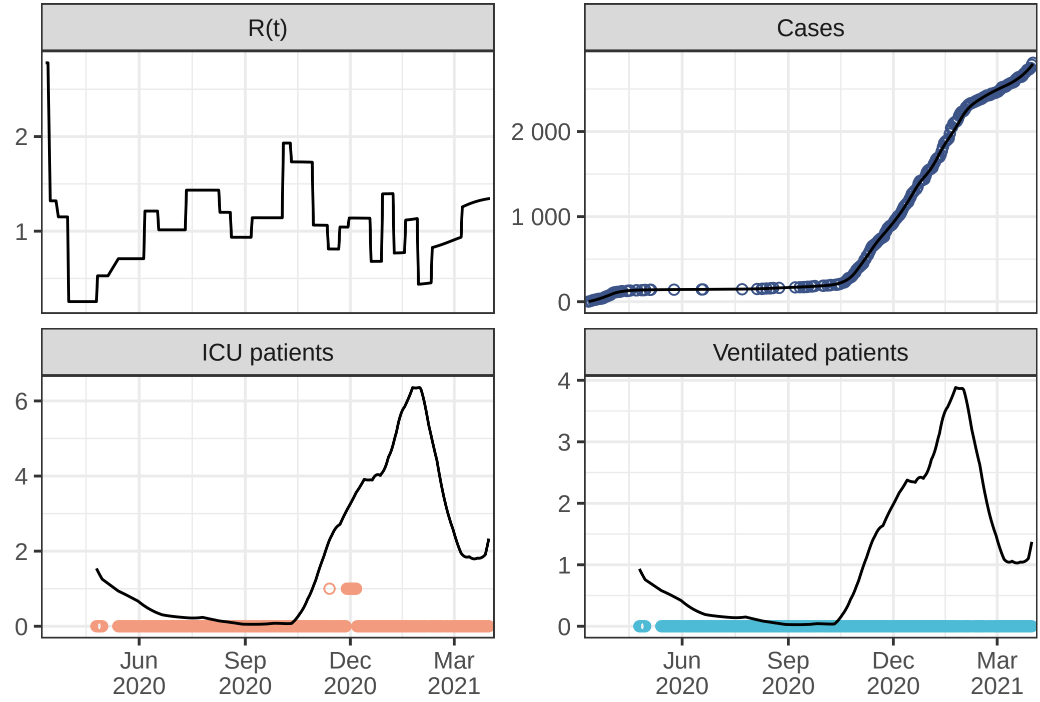

LK Südwestpfalz

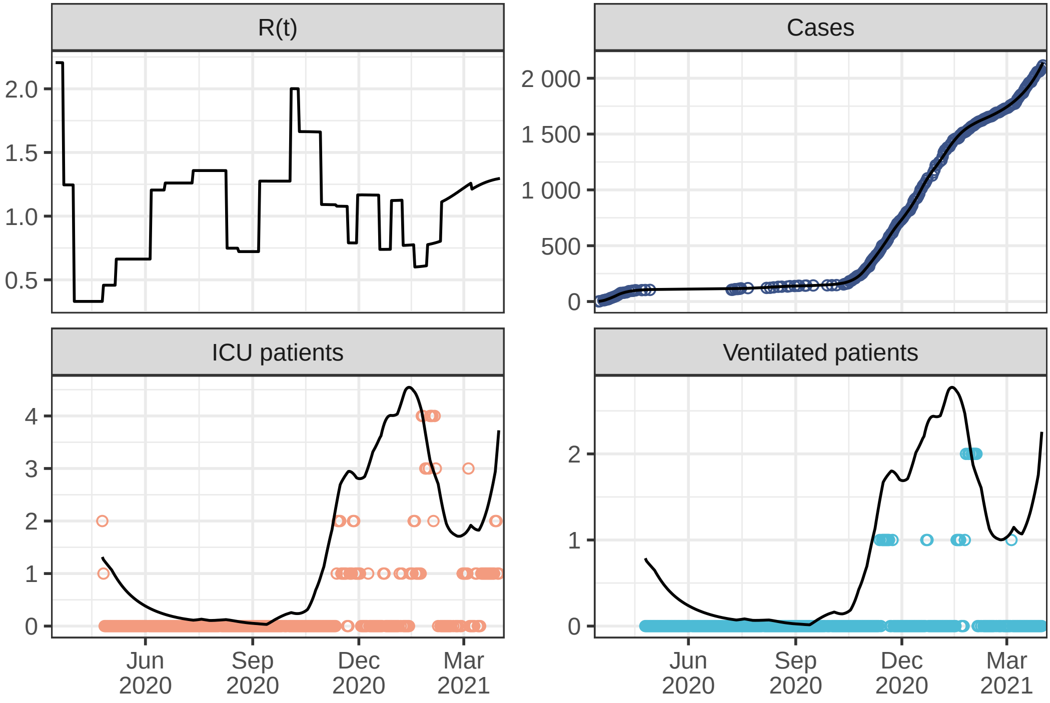

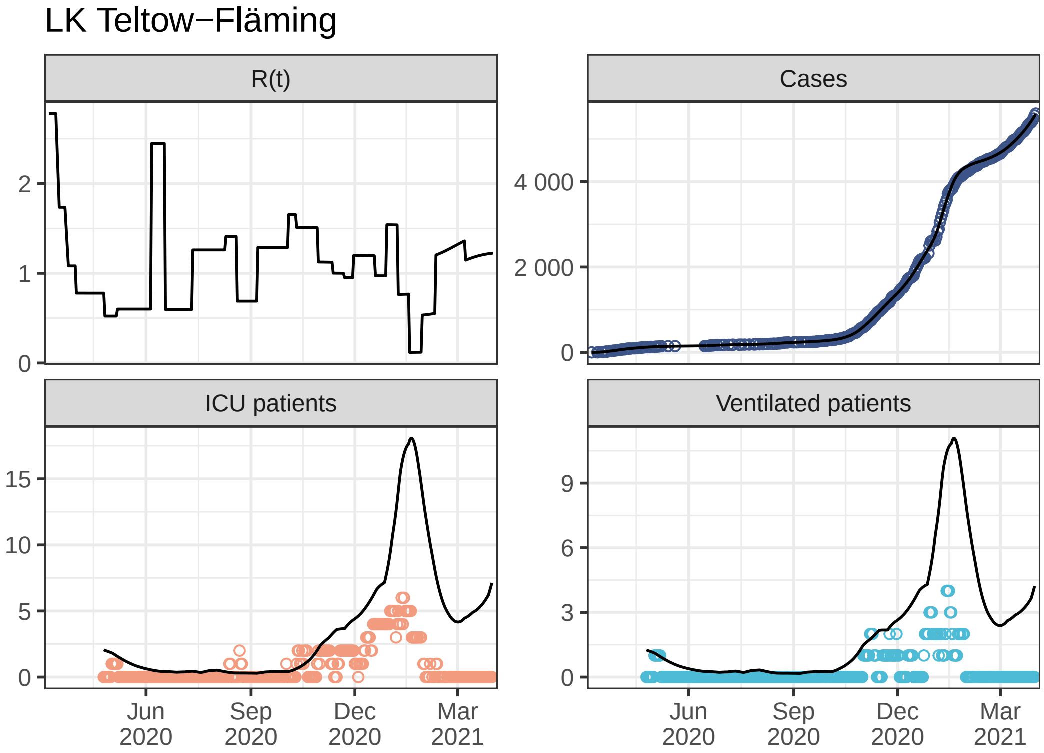
LK Teltow−Fläming
R(t)
Cases
ICU patients
Ventilated patients
0
1
2
0
2 000
4 000
0
5
10
15
0
3
6
9
Jun 2020
Sep 2020
Dec 2020
Mar 2021

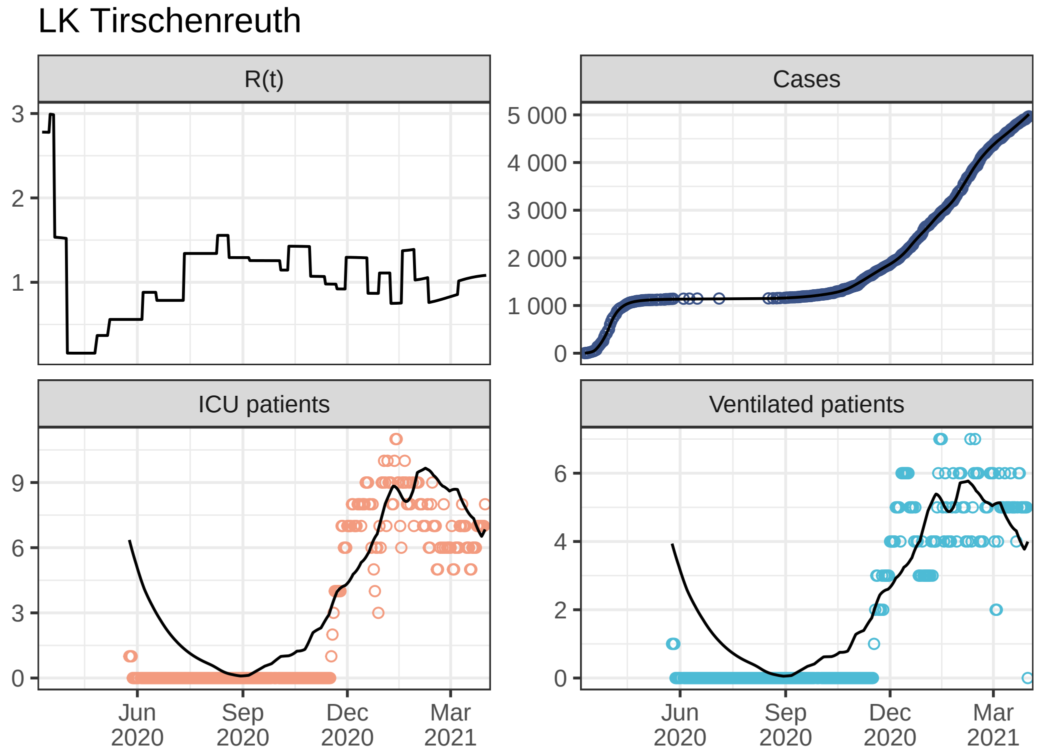
LK Tirschenreuth
R(t)
Cases
ICU patients
Ventilated patients
1
2
3
0
1 000
2 000
3 000
4 000
5 000
0
3
6
9
0
2
4
6
Jun 2020
Sep 2020
Dec 2020
Mar 2021

LK Traunstein

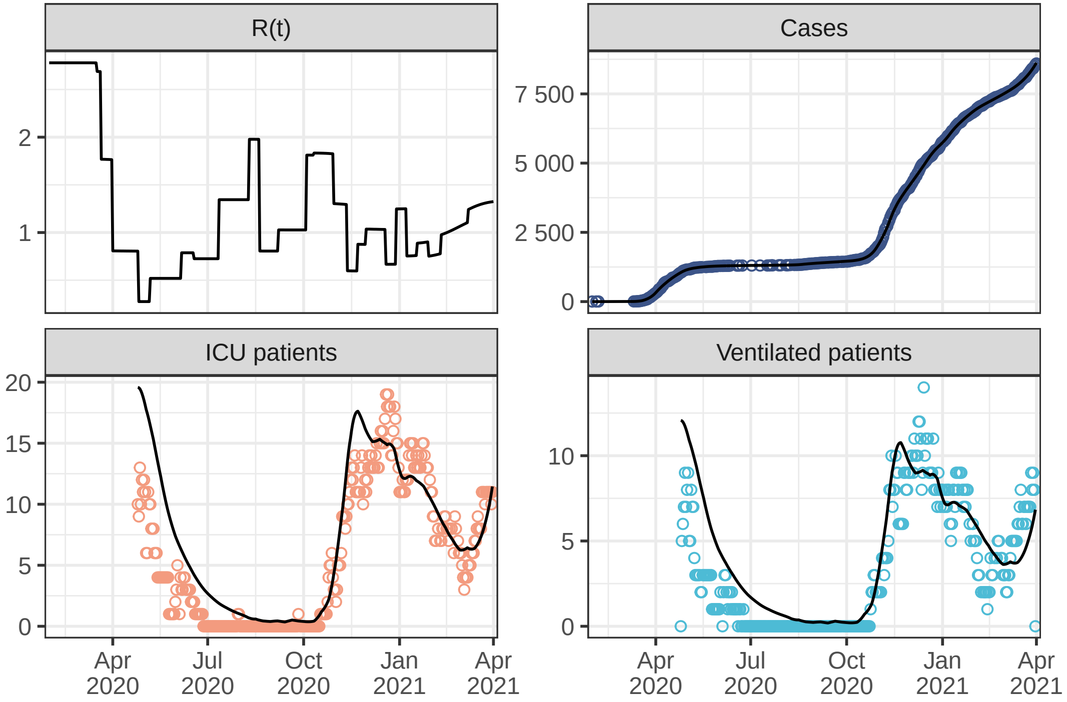

LK Trier−Saarburg

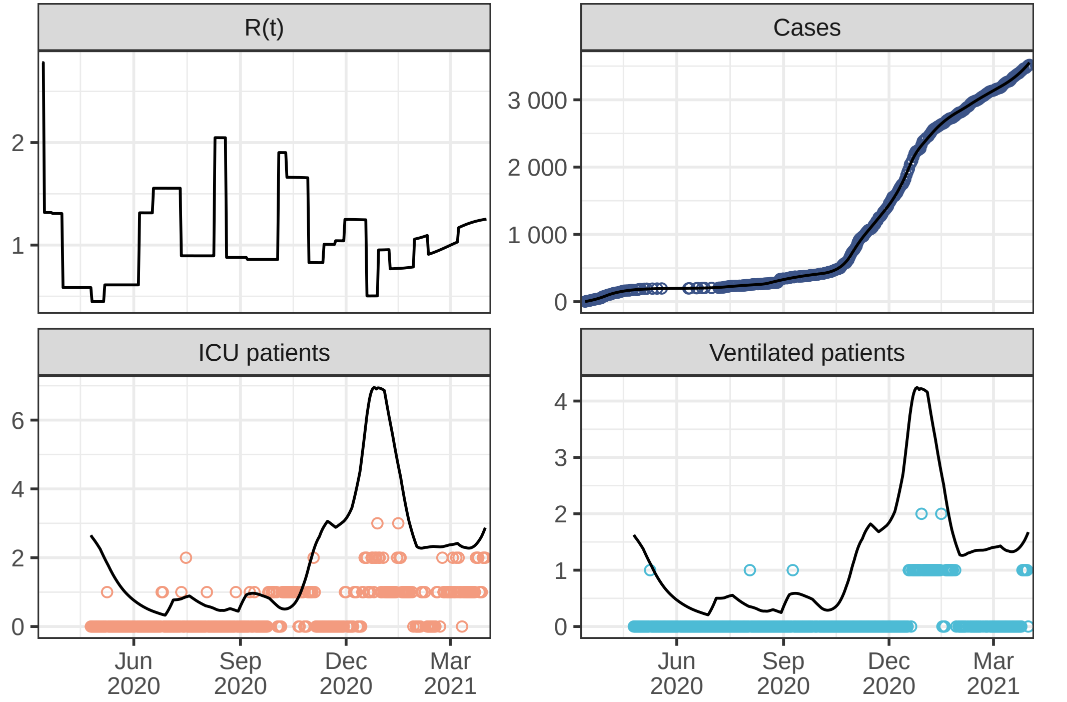

LK Tübingen

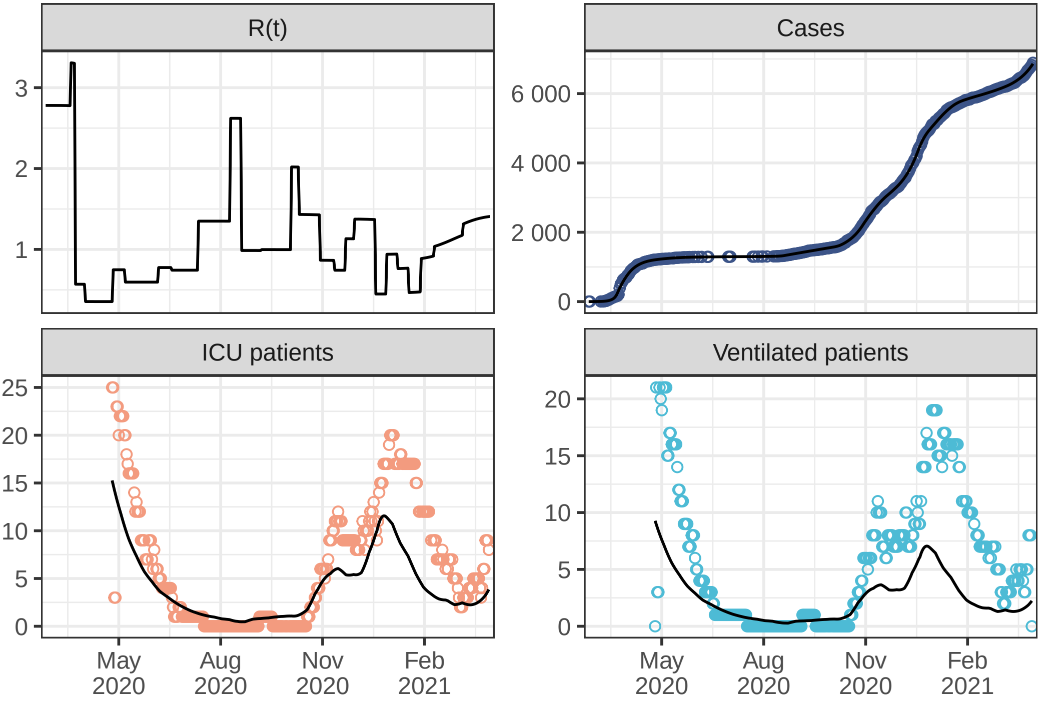

LK Tuttlingen

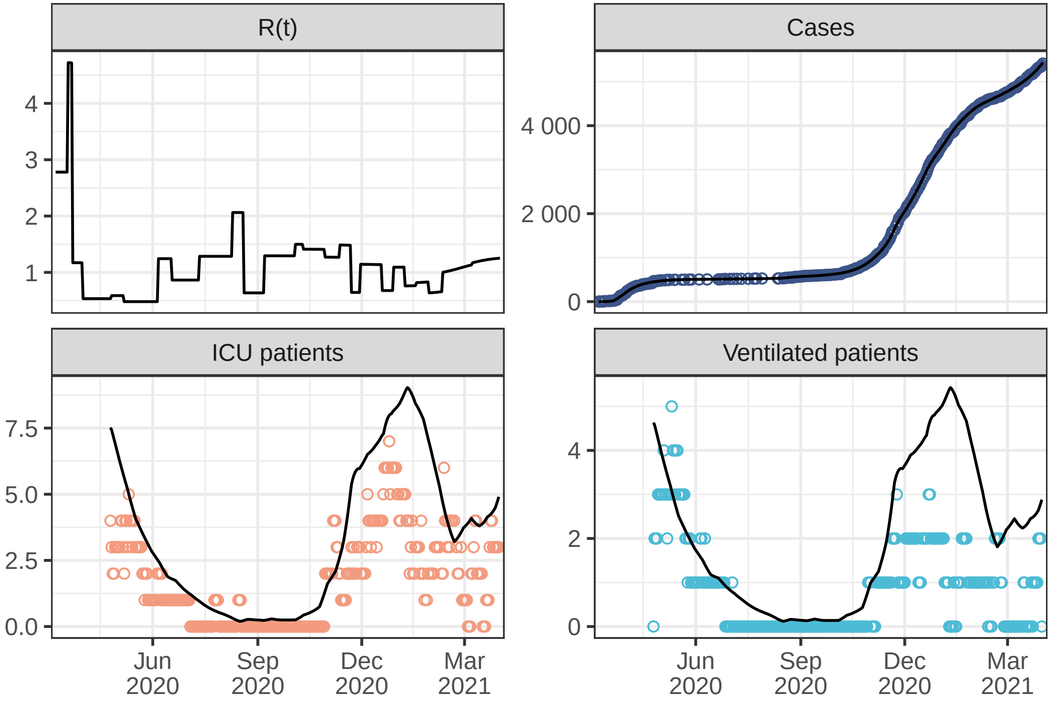

LK Uckermark

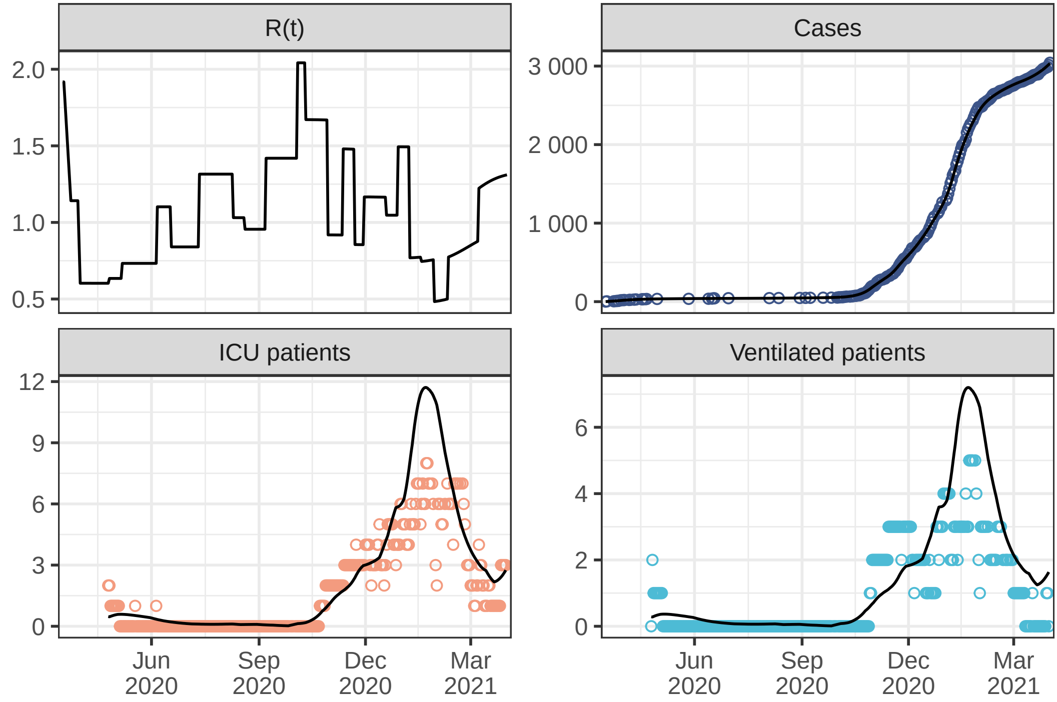

LK Uelzen

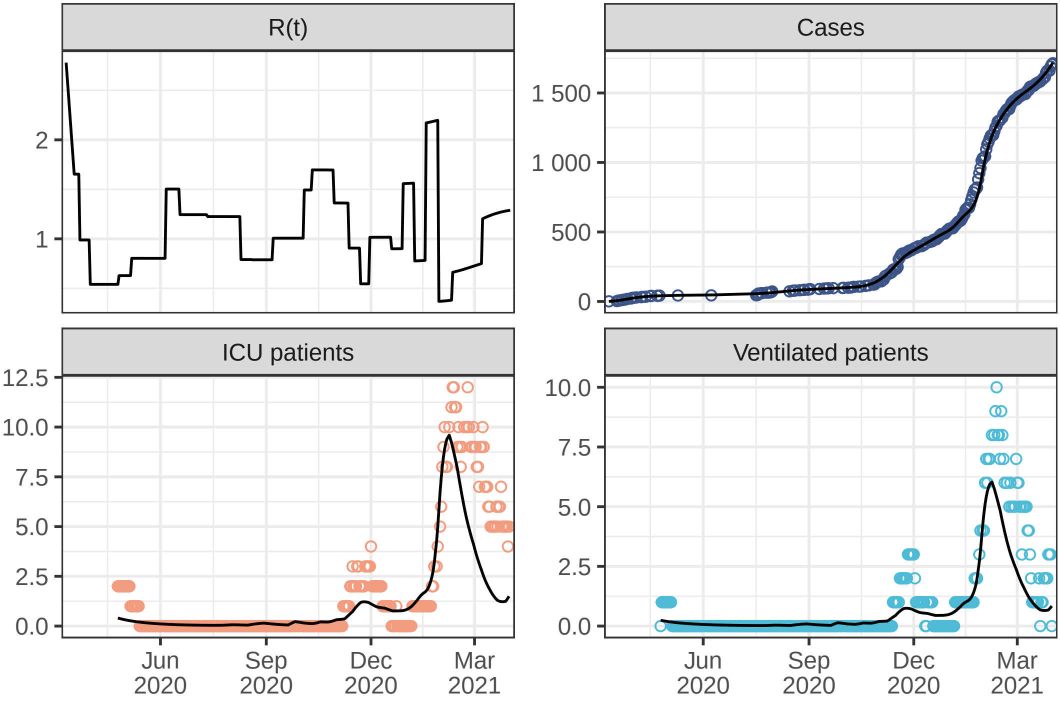

LK Unna

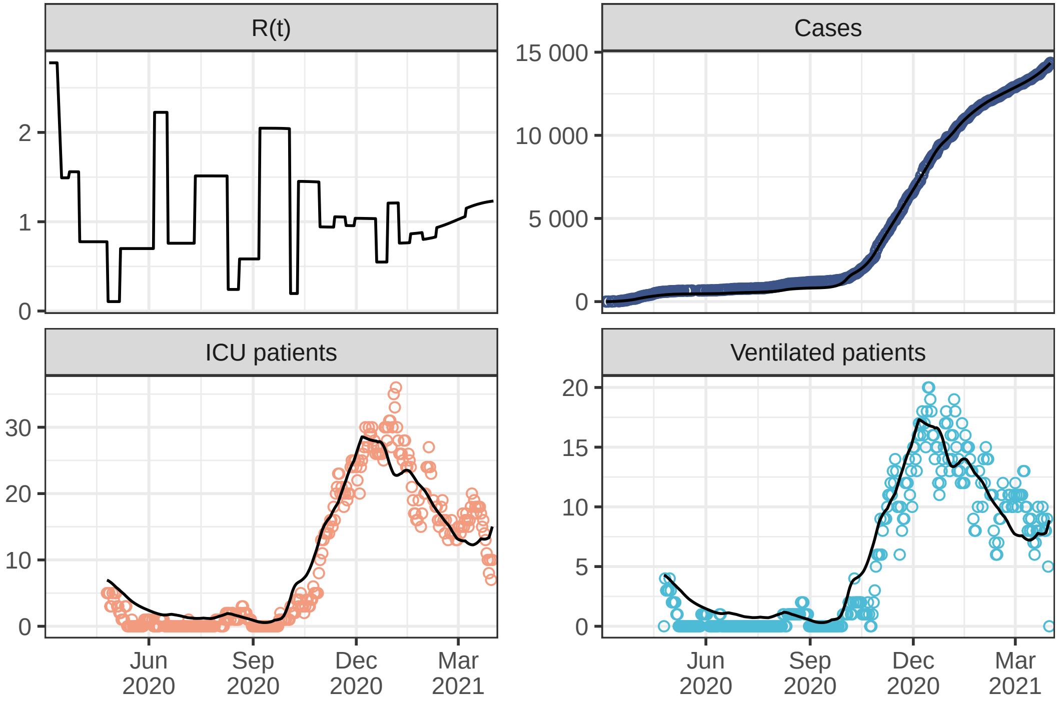

LK Unstrut−Hainich−Kreis

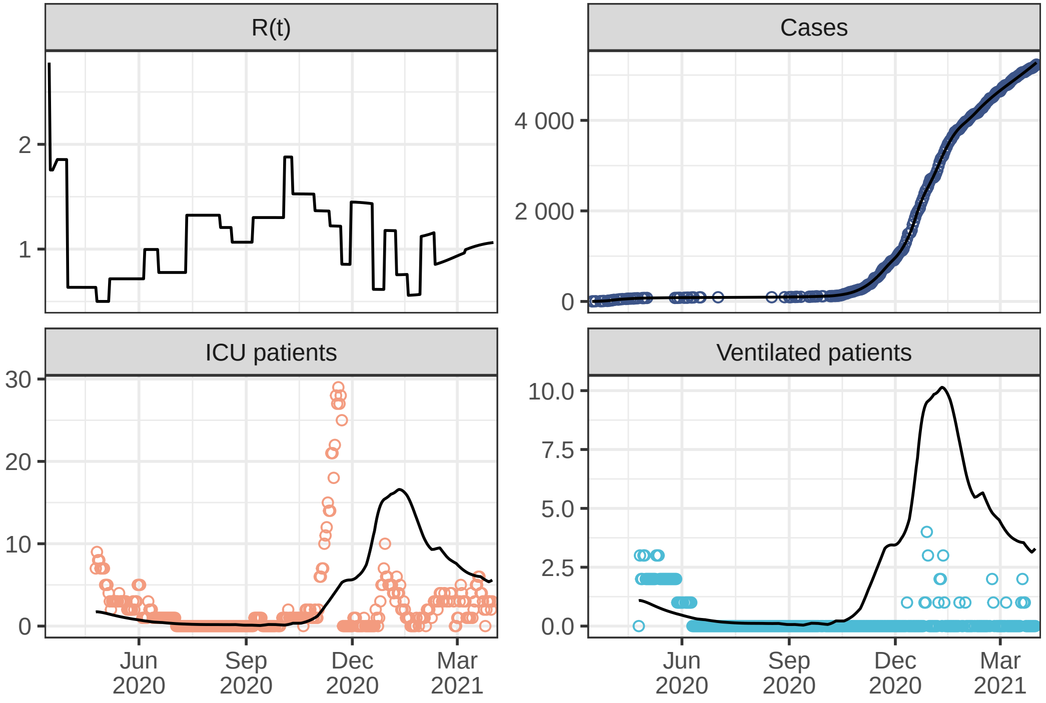

LK Unterallgäu

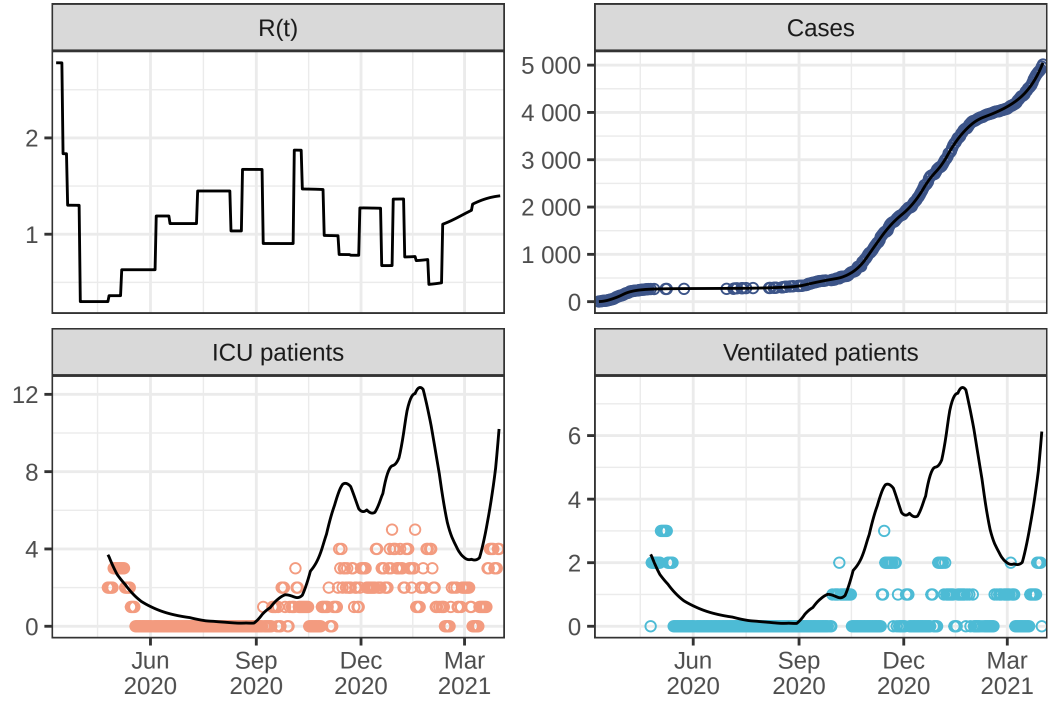

LK Vechta

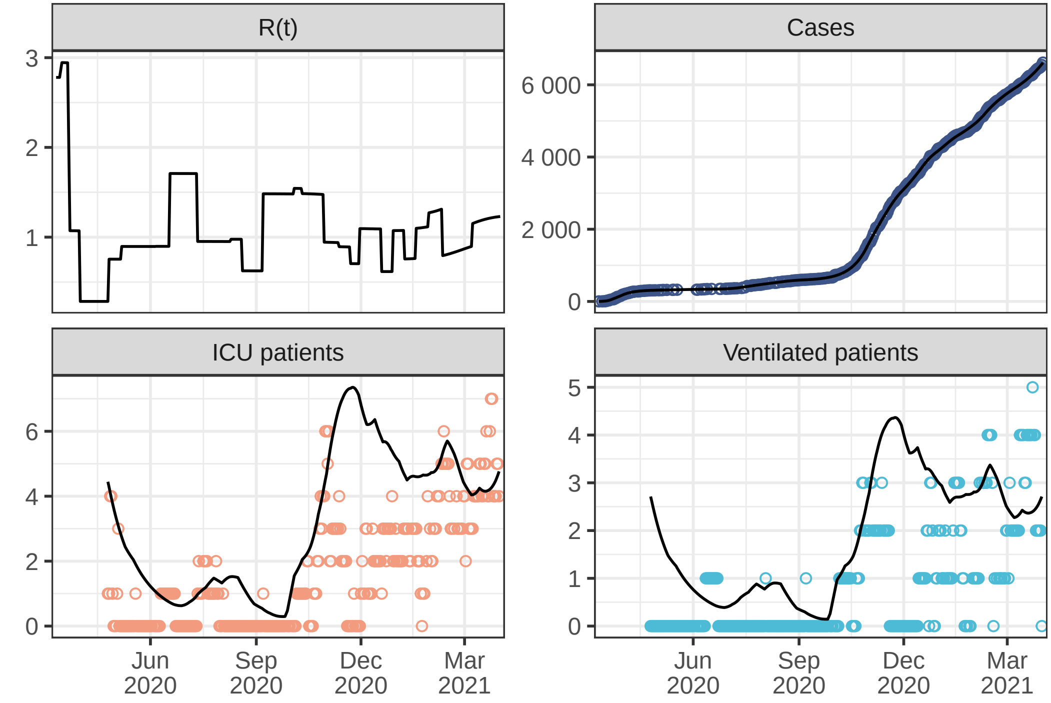

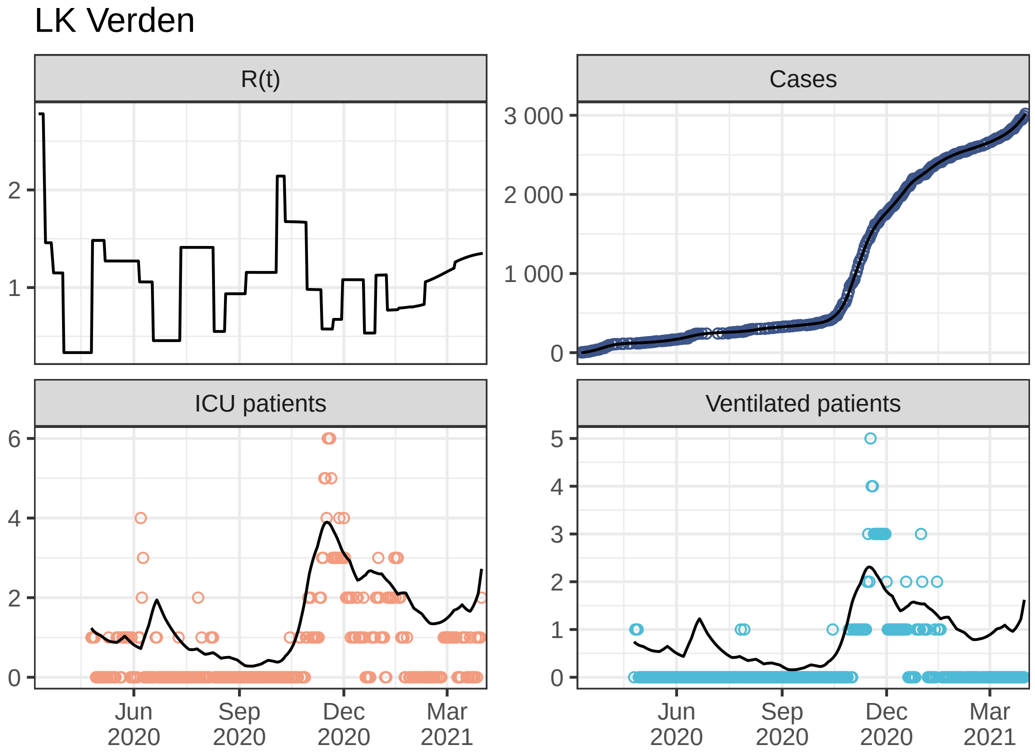
LK Verden
R(t)
Cases
ICU patients
Ventilated patients
2
1
3 000
2 000
1 000
0
6
4
2
0
5
4
3
2
1
0
Jun 2020
Sep 2020
Dec 2020
Mar 2021

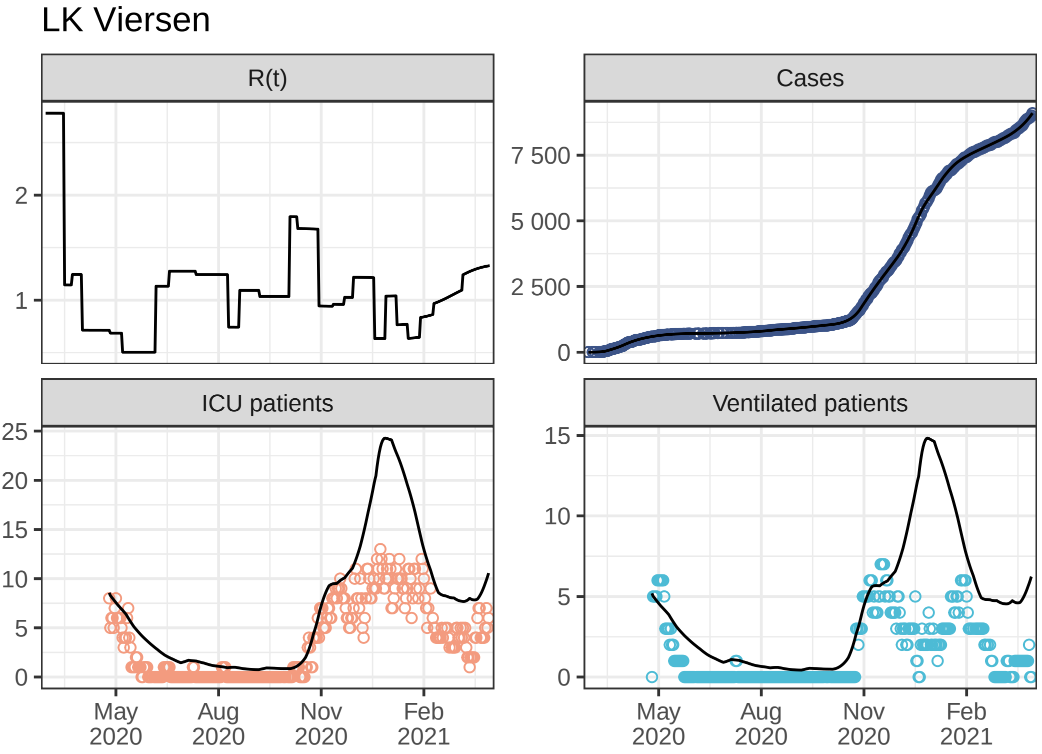
LK Viersen
R(t)
Cases
ICU patients
Ventilated patients
2
1
7 500
5 000
2 500
0
25
20
15
10
5
0
15
10
5
0
May 2020
Aug 2020
Nov 2020
Feb 2021

LK Vogelsbergkreis

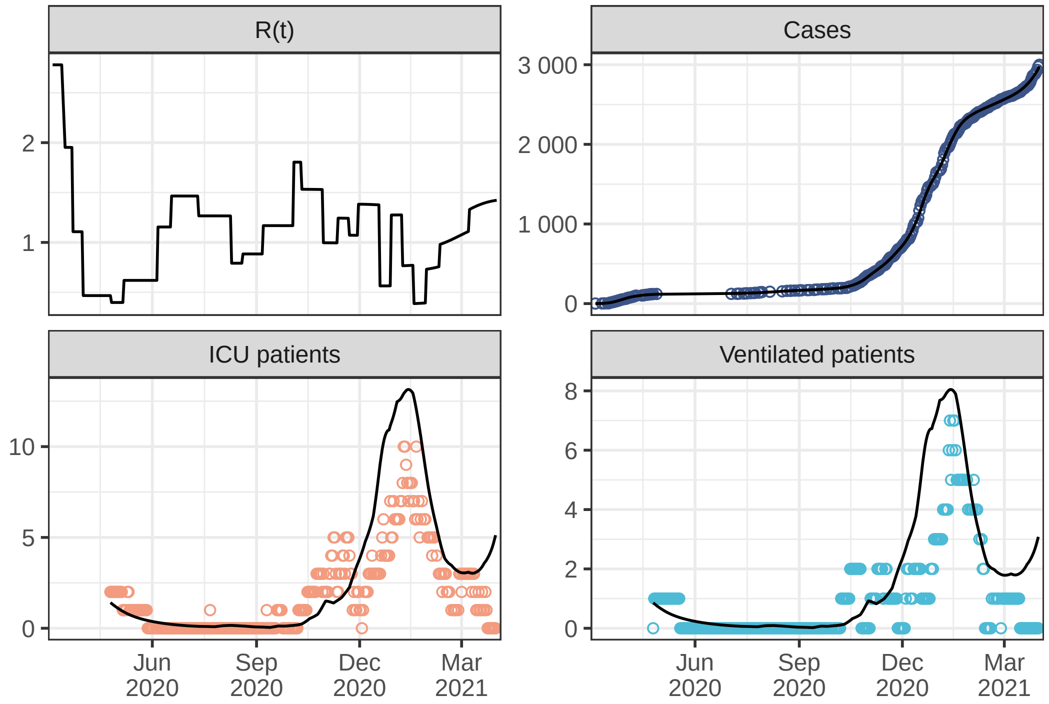

LK Vogtlandkreis

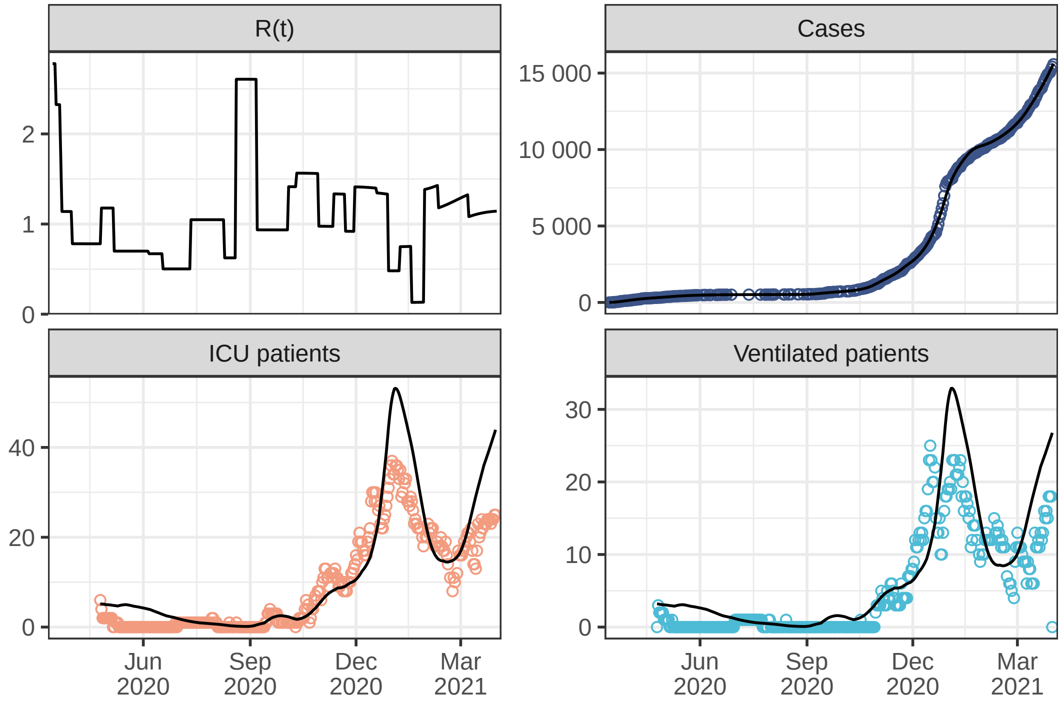

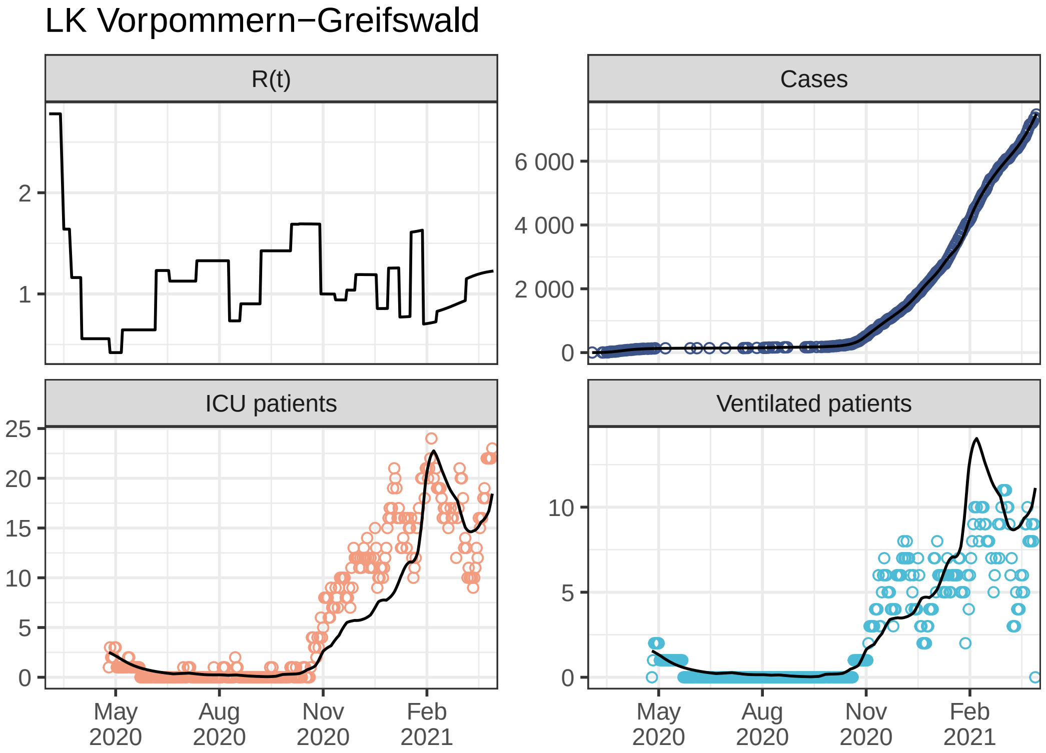
LK Vorpommern−Greifswald
R(t)
Cases
ICU patients
Ventilated patients
2
1
6 000
4 000
2 000
0
25
20
15
10
5
0
10
5
0
May 2020
Aug 2020
Nov 2020
Feb 2021
May 2020
Aug 2020
Nov 2020
Feb 2021

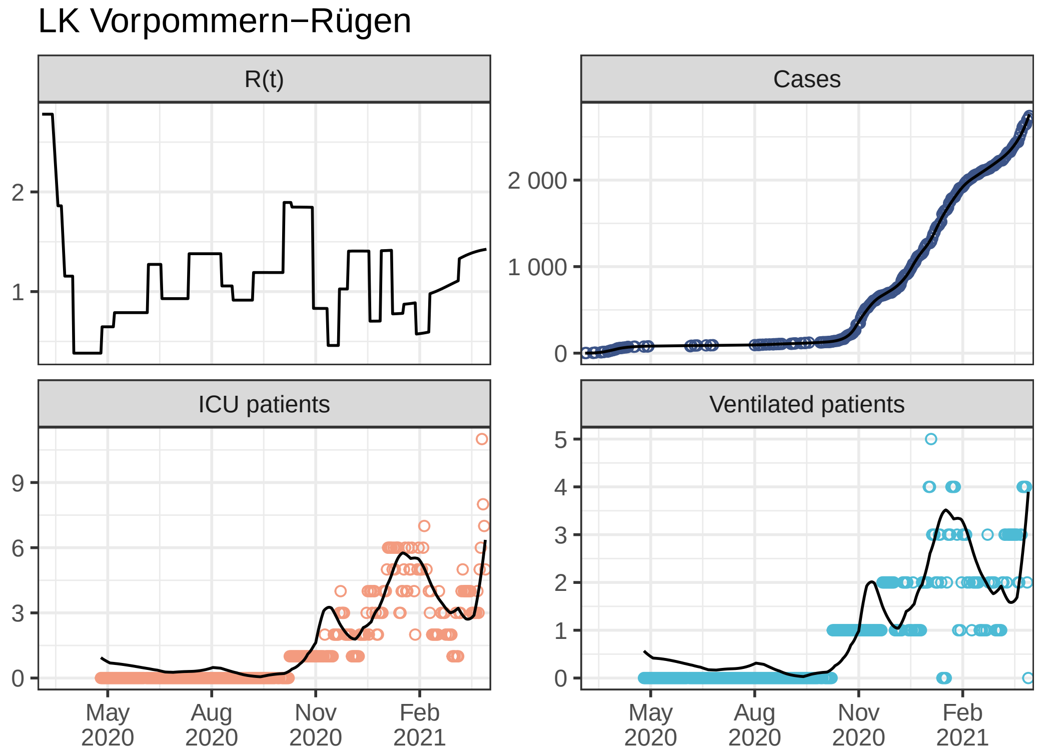
LK Vorpommern−Rügen
R(t)
Cases
ICU patients
Ventilated patients
2
1
2 000
1 000
0
9
6
3
0
5
4
3
2
1
0
May 2020
Aug 2020
Nov 2020
Feb 2021
May 2020
Aug 2020
Nov 2020
Feb 2021

LK Vulkaneifel

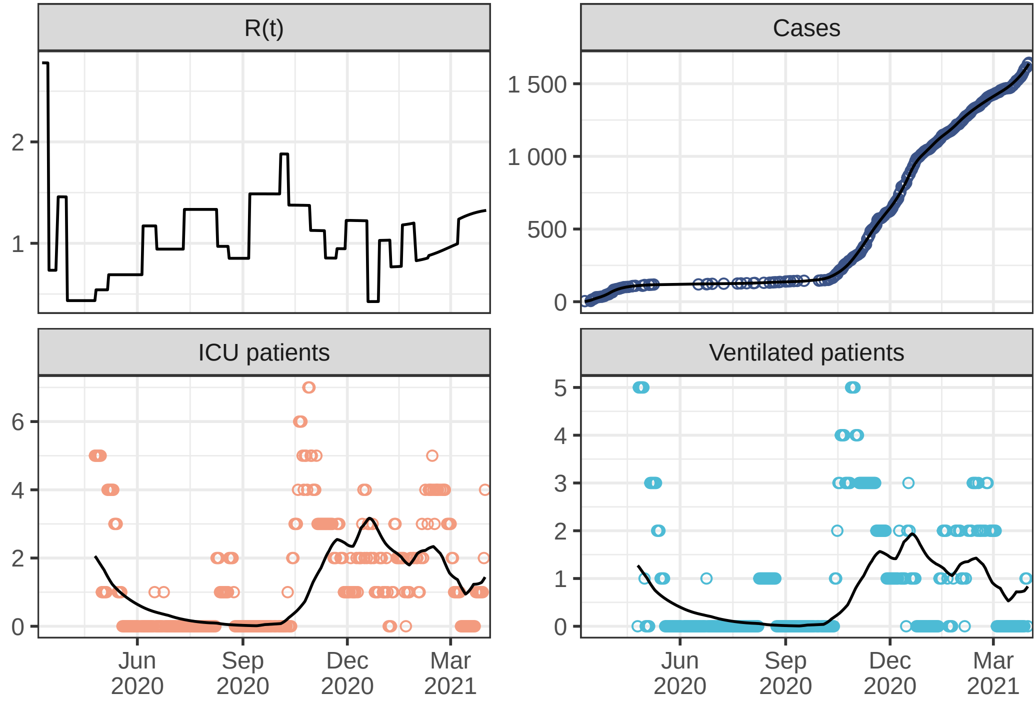

LK Waldeck−Frankenberg

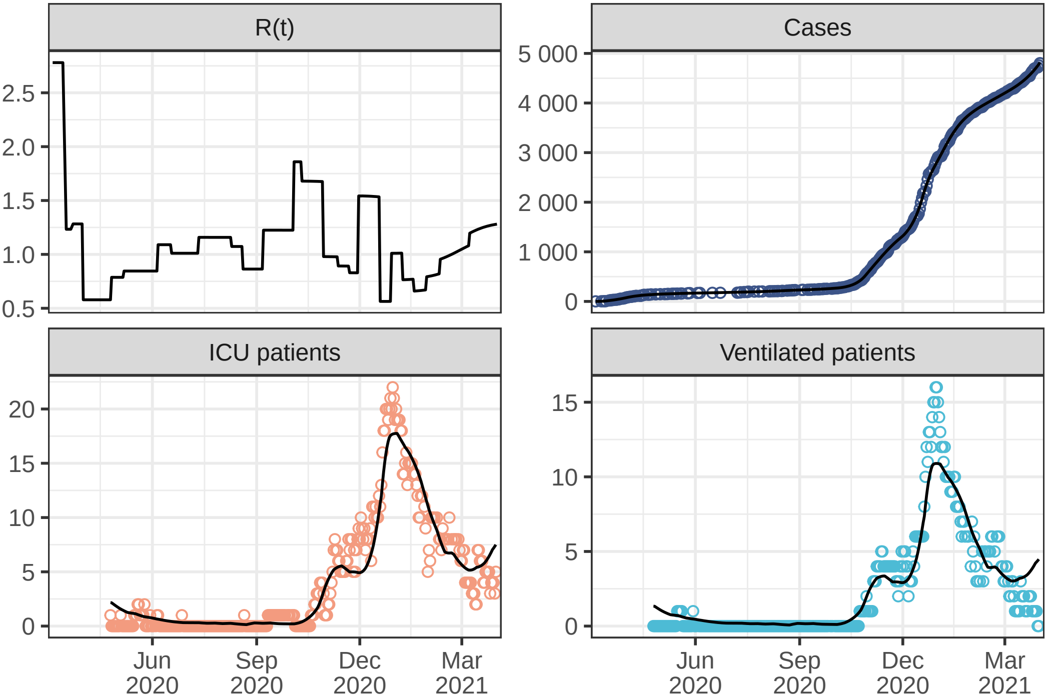

LK Waldshut

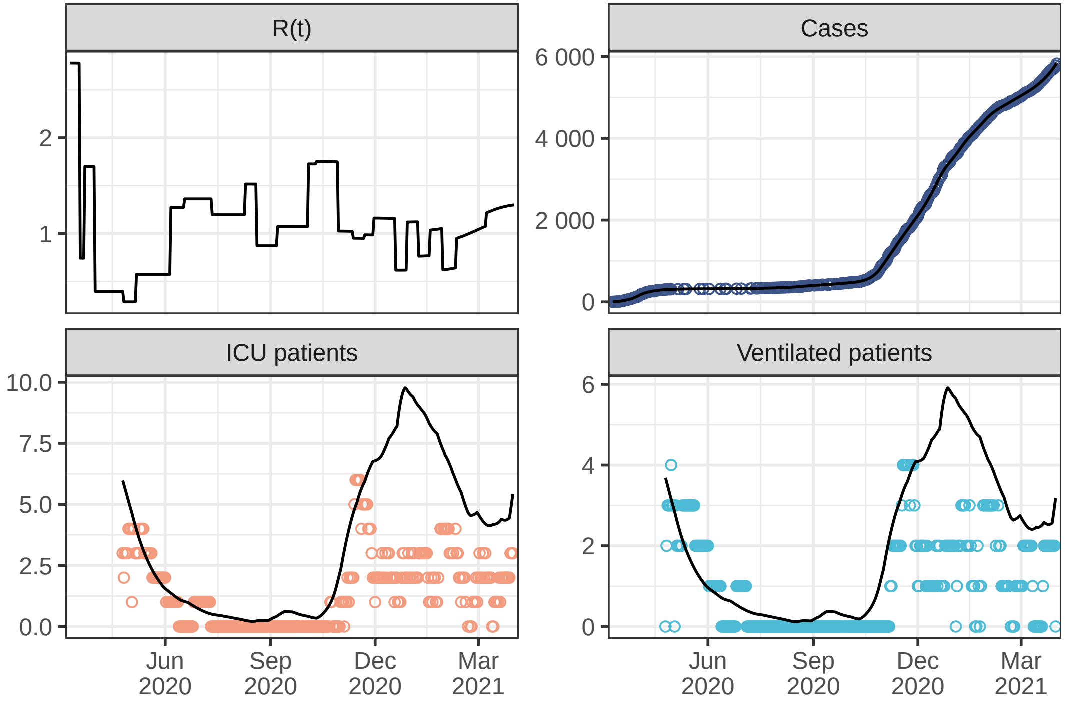

LK Warendorf

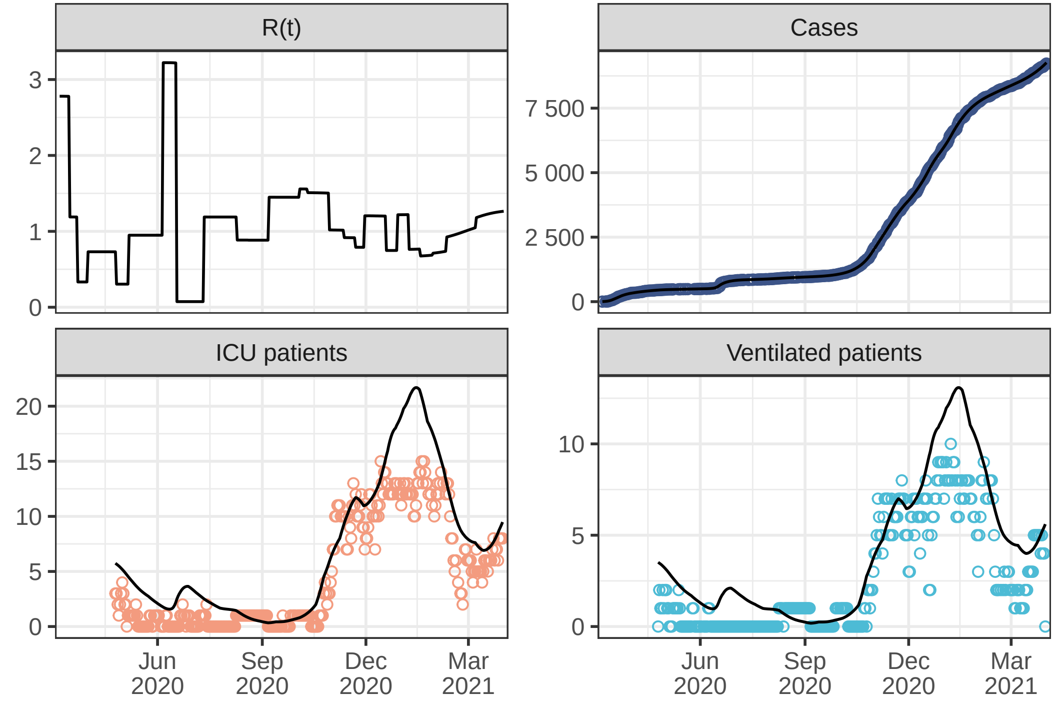

LK Wartburgkreis

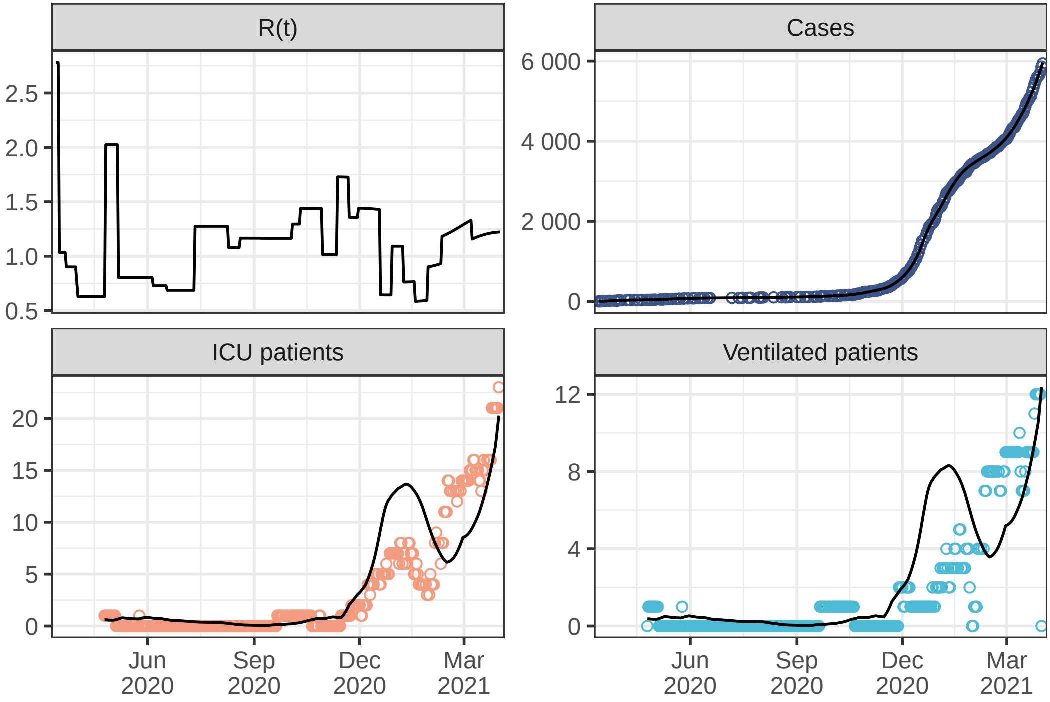

LK Weißenburg−Gunzenhausen

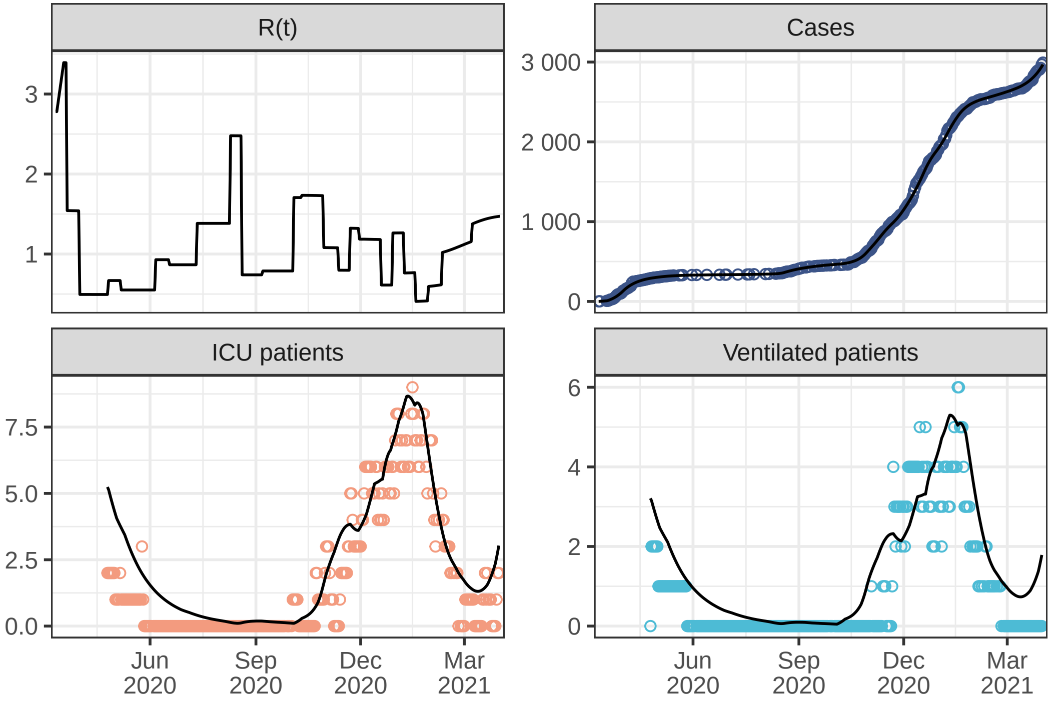

LK Weilheim−Schongau

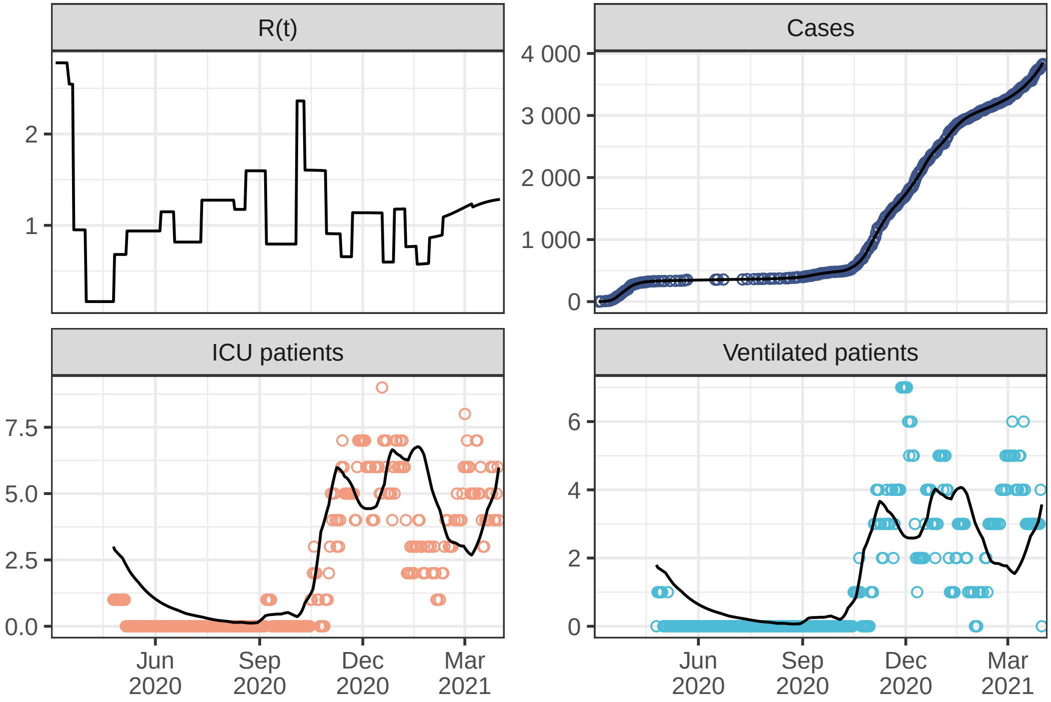

LK Weimarer Land

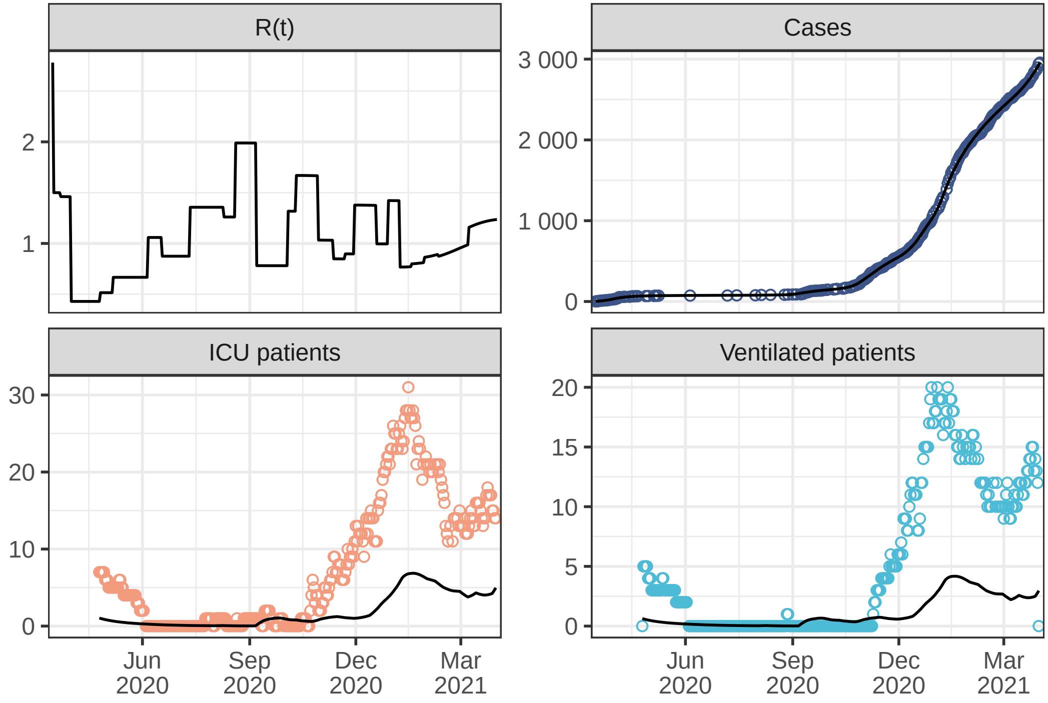

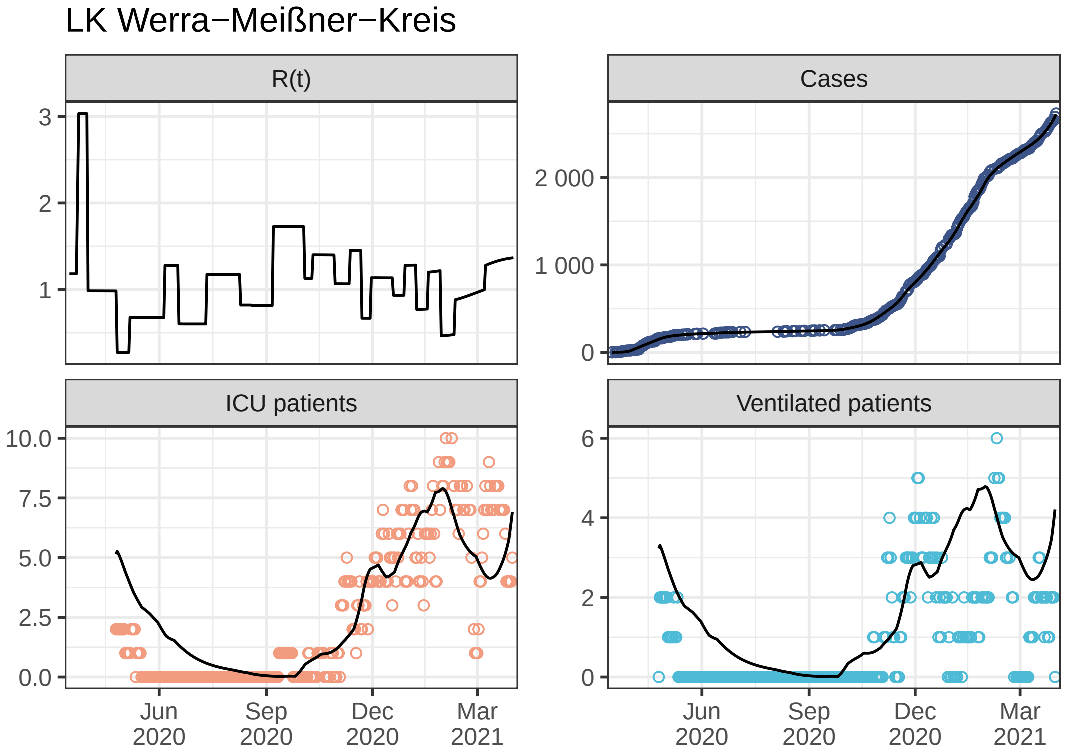
LK Werra−Meißner−Kreis
R(t)
Cases
ICU patients
Ventilated patients
3
2
1
2 000
1 000
0
10.0
7.5
5.0
2.5
0.0
6
4
2
0
Jun 2020
Sep 2020
Dec 2020
Mar 2021

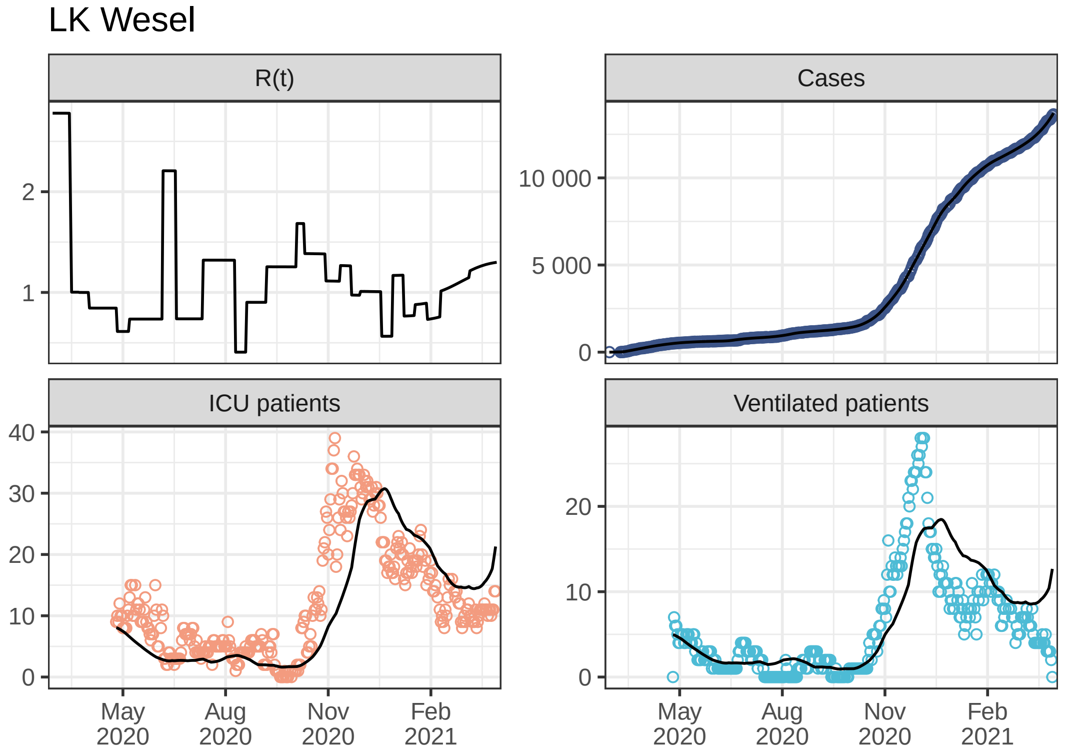
LK Wesel
R(t)
Cases
ICU patients
Ventilated patients
2
1
10 000
5 000
0
40
30
20
10
0
20
10
0
May 2020
Aug 2020
Nov 2020
Feb 2021

LK Wesermarsch

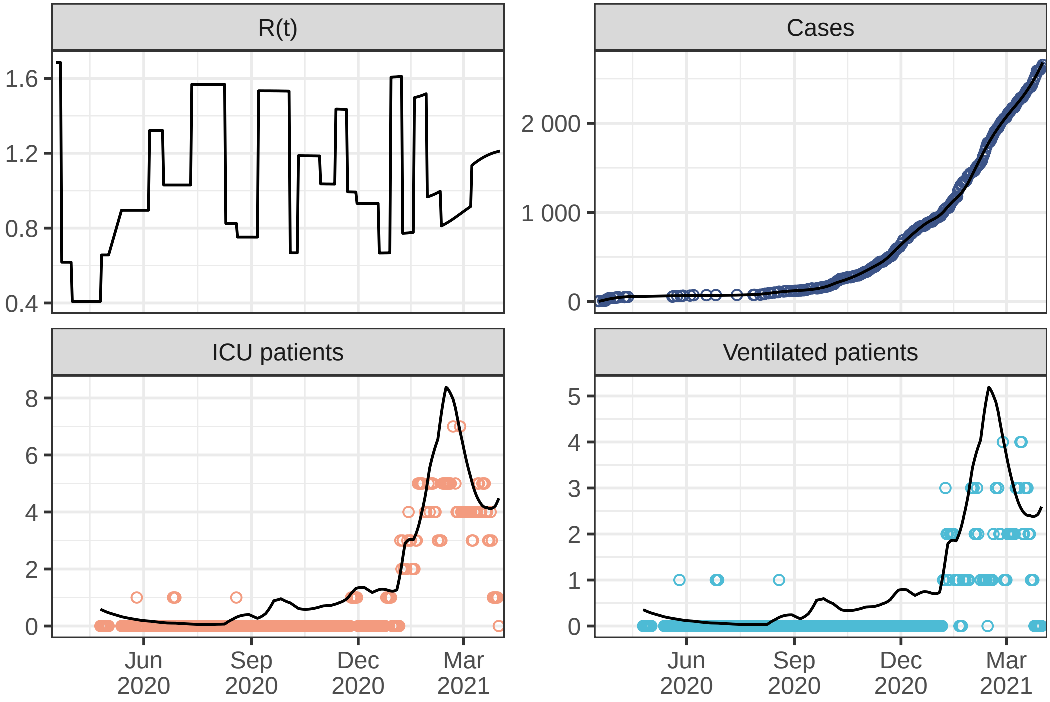

LK Westerwaldkreis

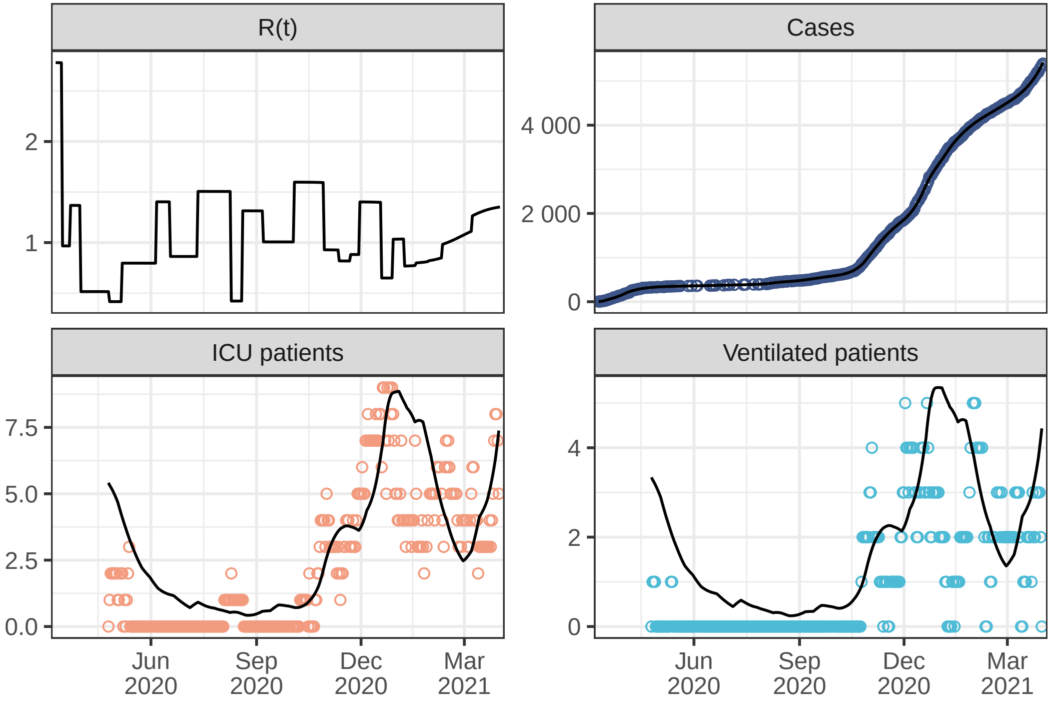

LK Wetteraukreis

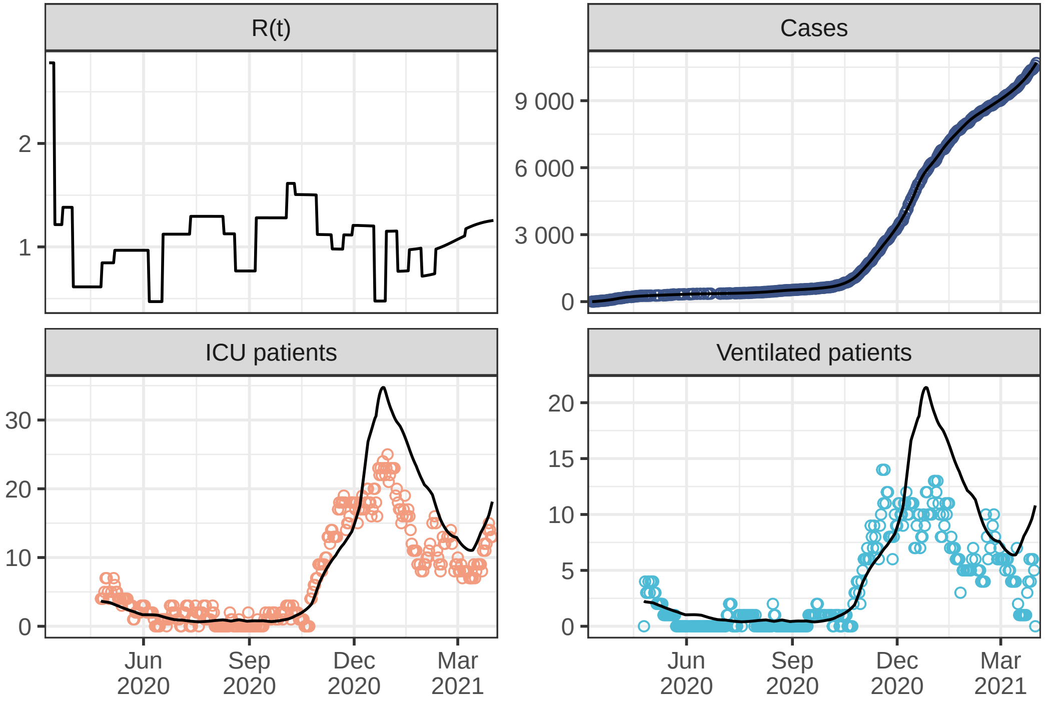

LK Wittenberg

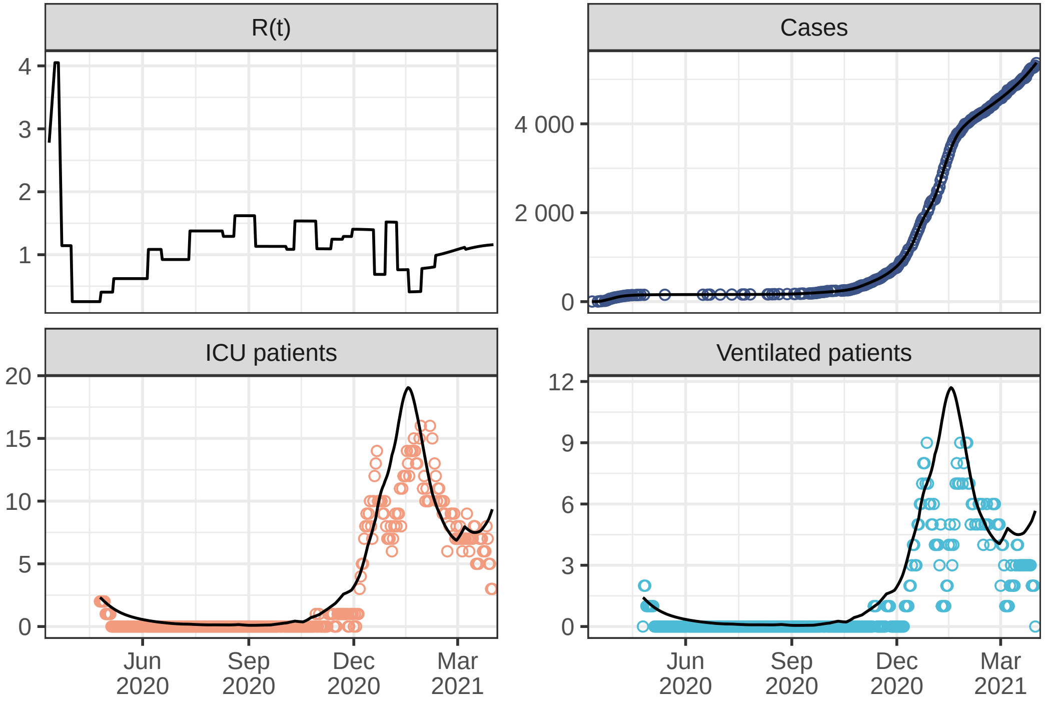

LK Wittmund

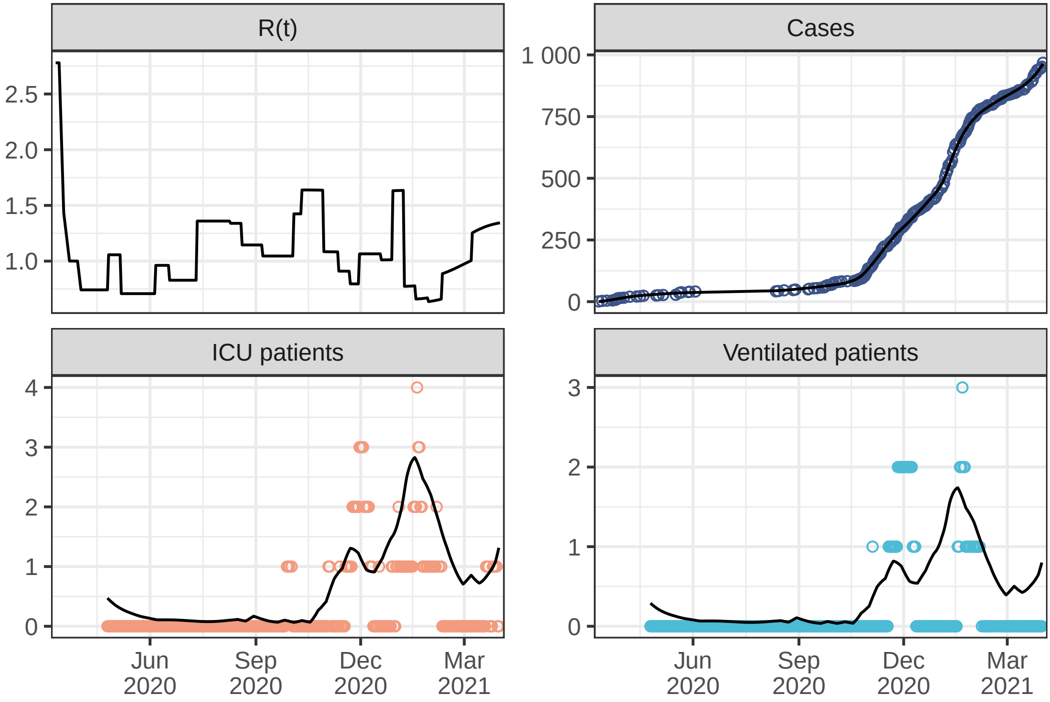

LK Wolfenbüttel

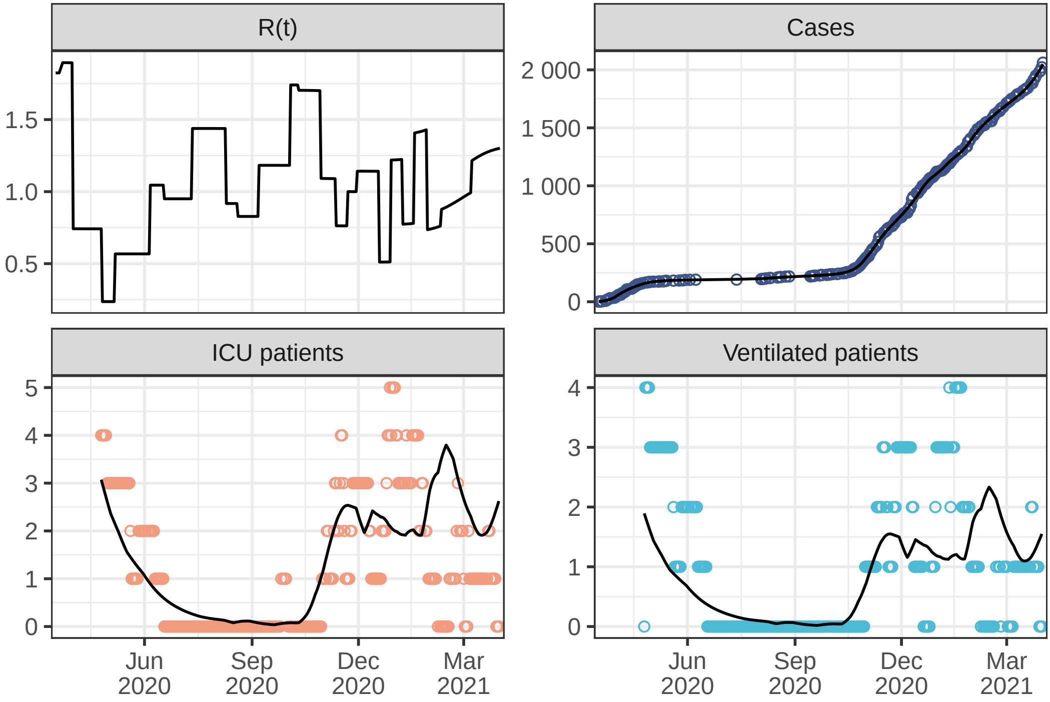

LK Wunsiedel i.Fichtelgebirge

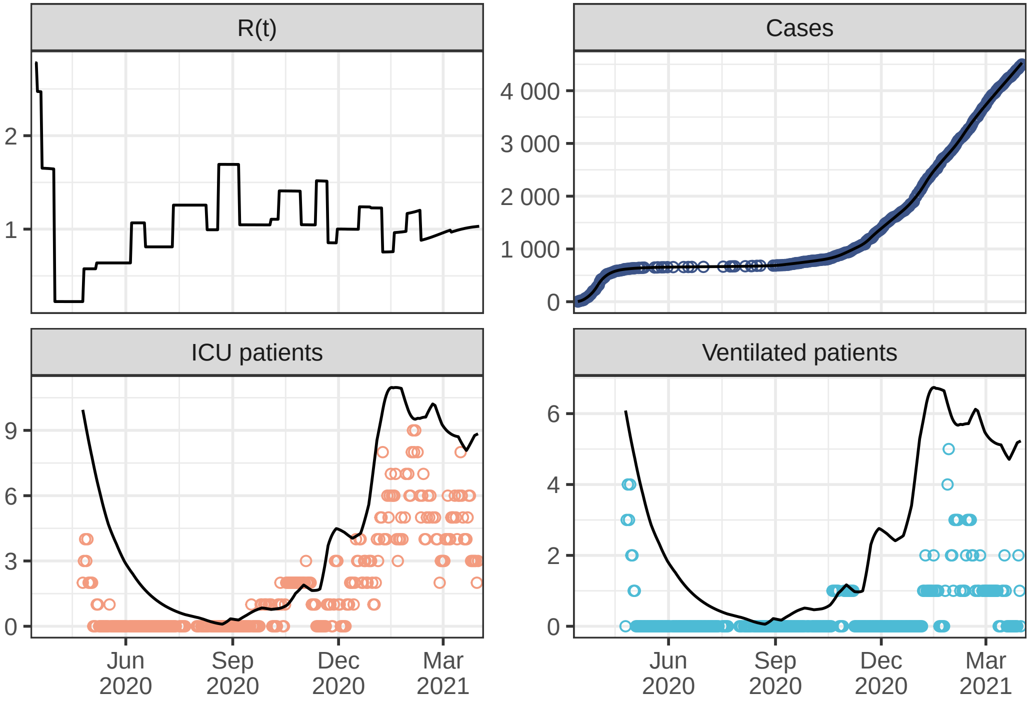

LK Würzburg

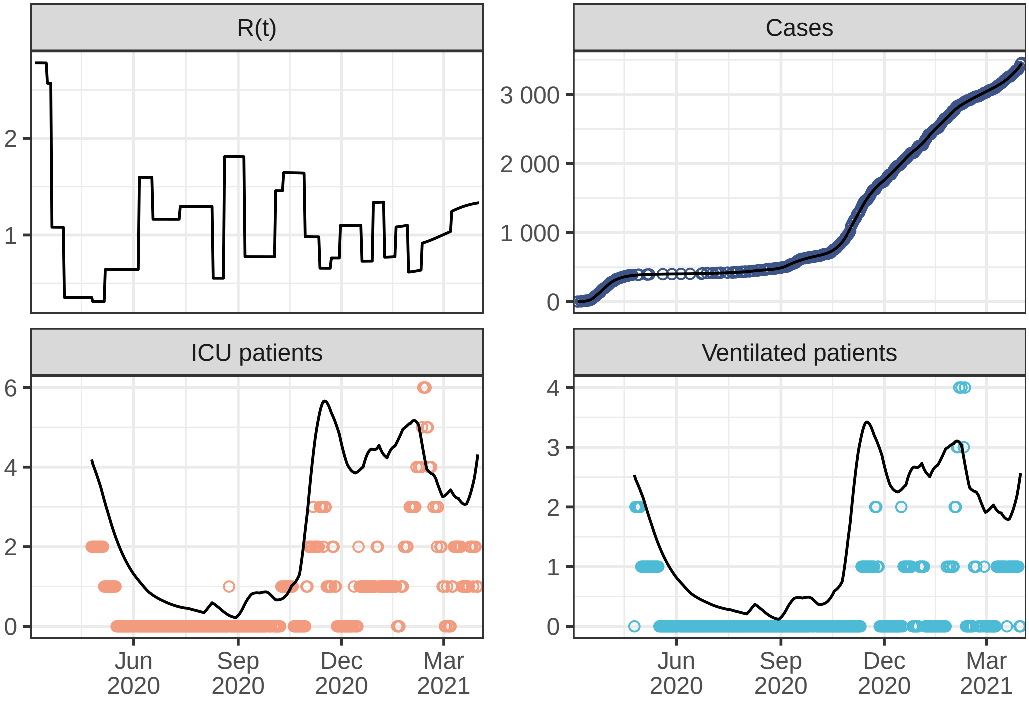

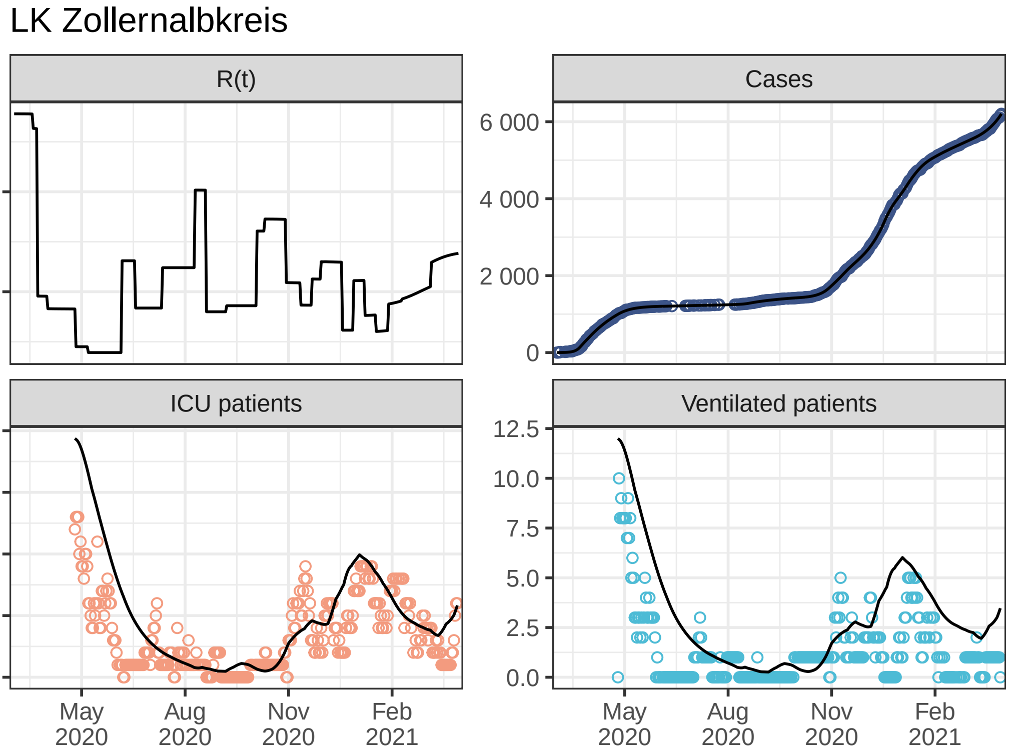
LK Zollernalbkreis
R(t)
Cases
ICU patients
Ventilated patients
2
1
6 000
4 000
2 000
0
20
15
10
5
0
12.5
10.0
7.5
5.0
2.5
0.0
May 2020
Aug 2020
Nov 2020
Feb 2021

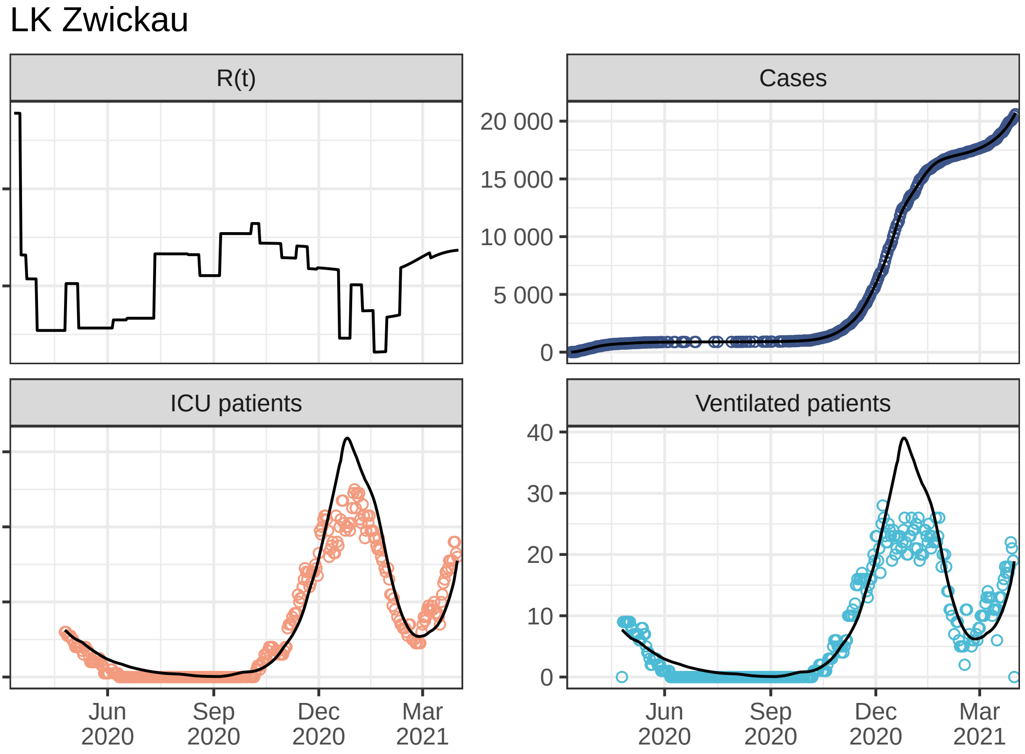
LK Zwickau
R(t)
Cases
ICU patients
Ventilated patients
2
1
20 000
15 000
10 000
5 000
0
60
40
20
0
40
30
20
10
0
Jun 2020
Sep 2020
Dec 2020
Mar 2021

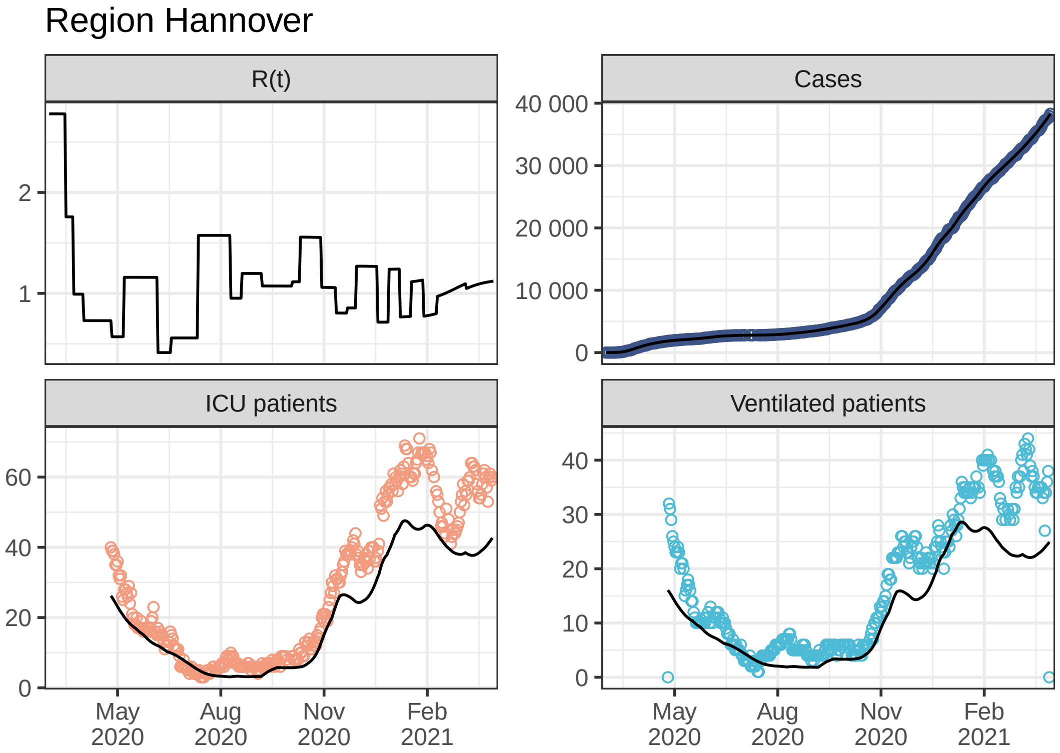
Region Hannover
R(t)
Cases
ICU patients
Ventilated patients
2
1
40 000
30 000
20 000
10 000
0
60
40
20
0
40
30
20
10
0
May 2020
Aug 2020
Nov 2020
Feb 2021

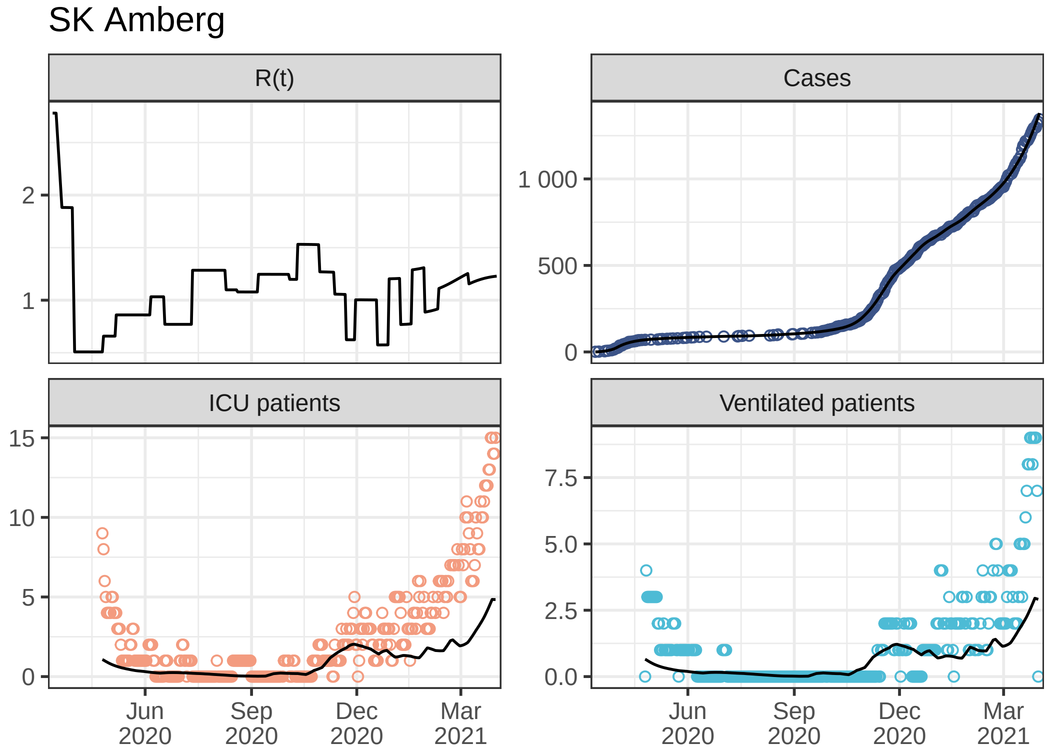
SK Amberg
R(t)
Cases
ICU patients
Ventilated patients
2
1
1 000
500
0
15
10
5
0
7.5
5.0
2.5
0.0
Jun 2020
Sep 2020
Dec 2020
Mar 2021

SK Ansbach

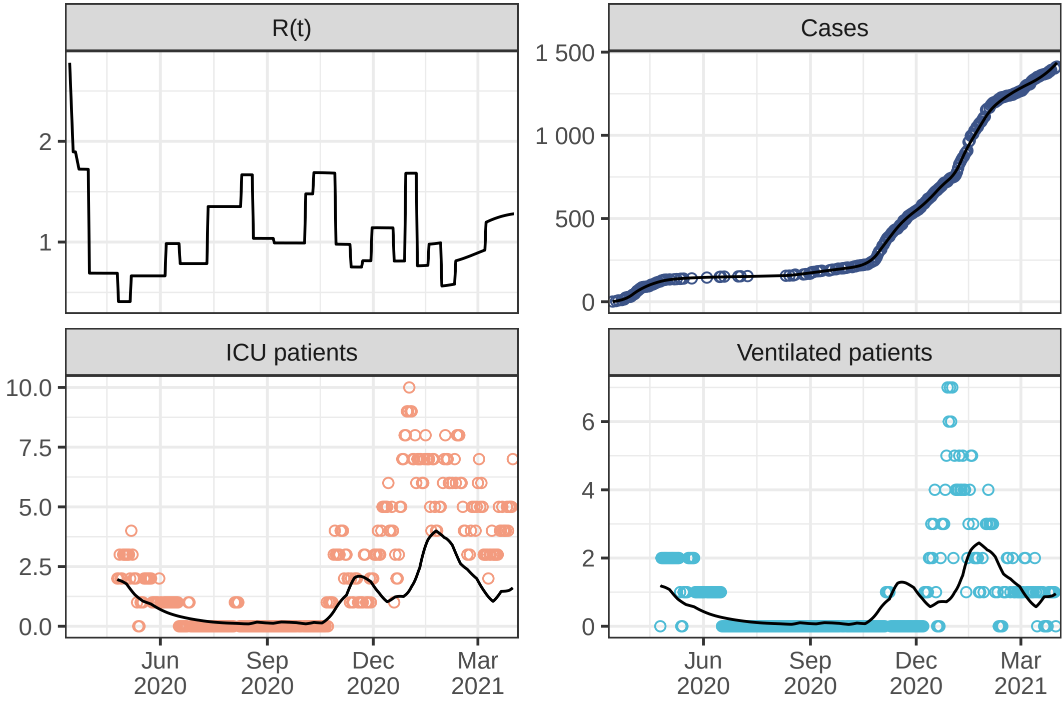

SK Aschaffenburg

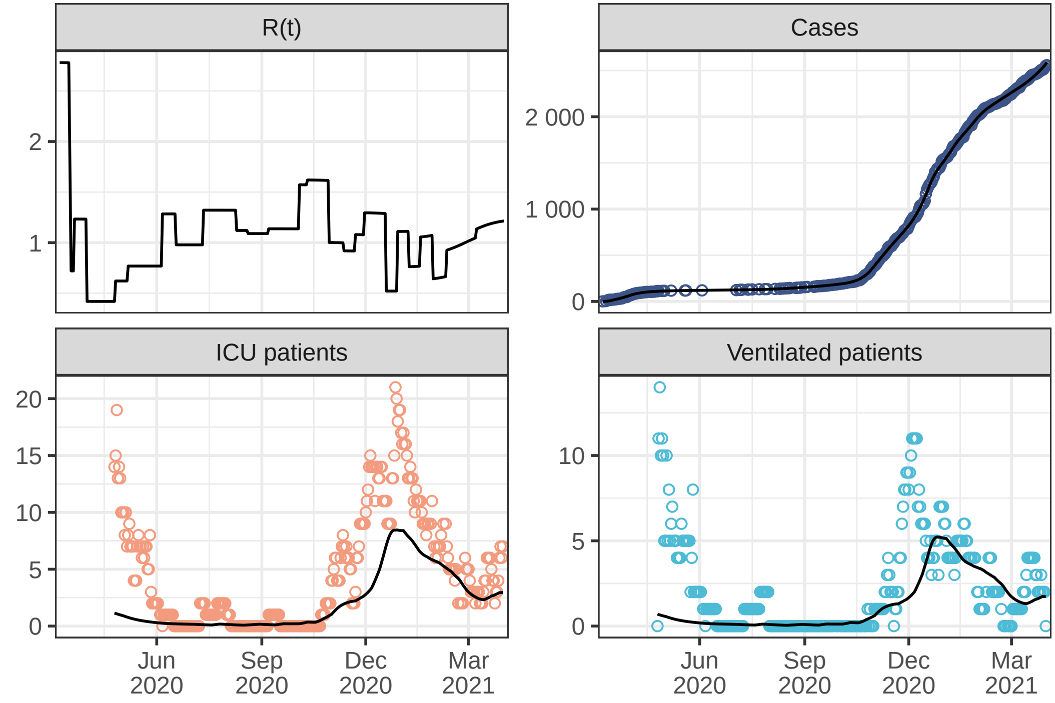

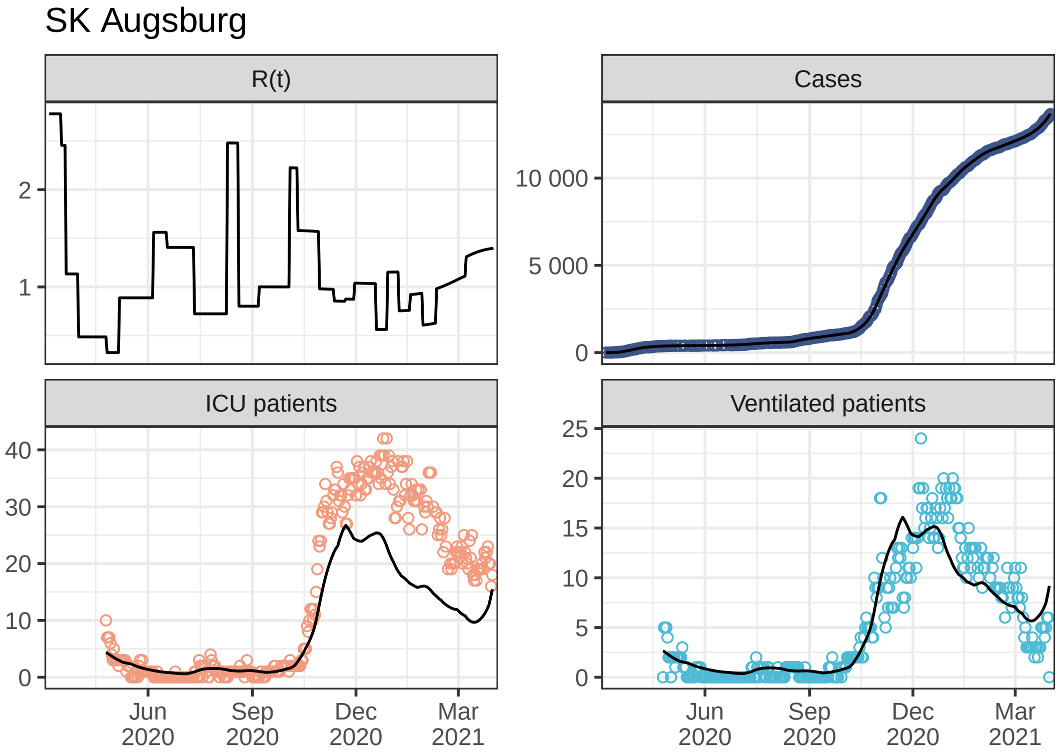
SK Augsburg
R(t)
2
1
Cases
10 000
5 000
0
ICU patients
40
30
20
10
0
Jun 2020
Sep 2020
Dec 2020
Mar 2021
Ventilated patients
25
20
15
10
5
0
Jun 2020
Sep 2020
Dec 2020
Mar 2021

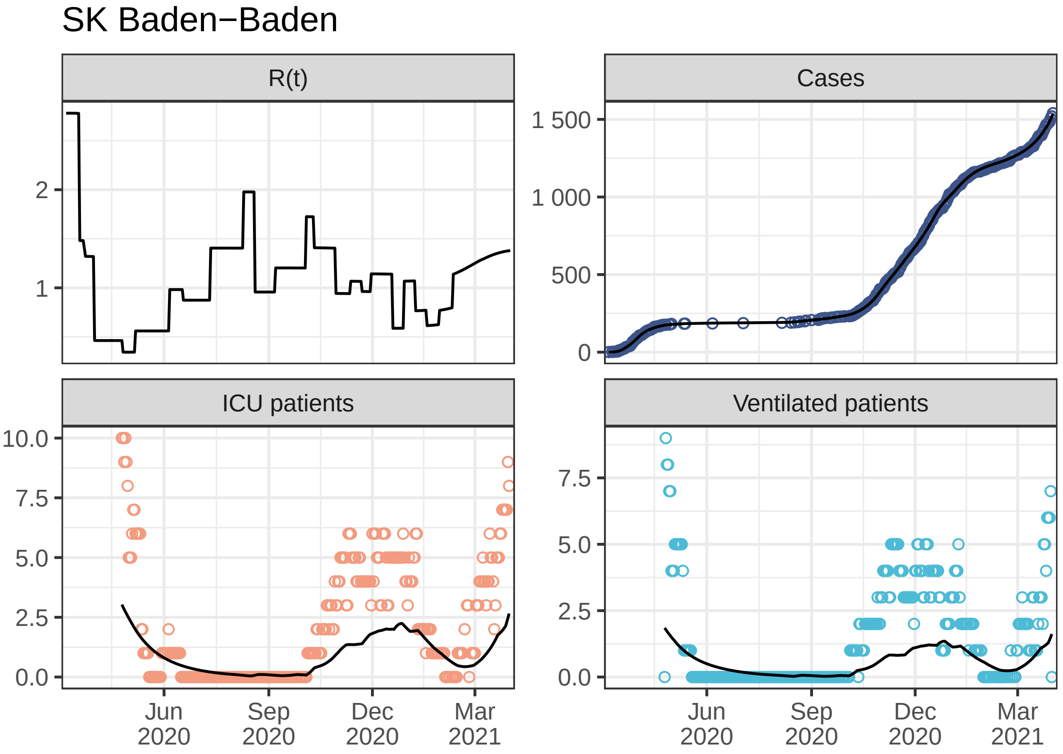
SK Baden−Baden
R(t)
2
1
Cases
1 500
1 000
500
0
ICU patients
10.0
7.5
5.0
2.5
0.0
Jun 2020
Sep 2020
Dec 2020
Mar 2021
Ventilated patients
7.5
5.0
2.5
0.0
Jun 2020
Sep 2020
Dec 2020
Mar 2021

SK Bamberg

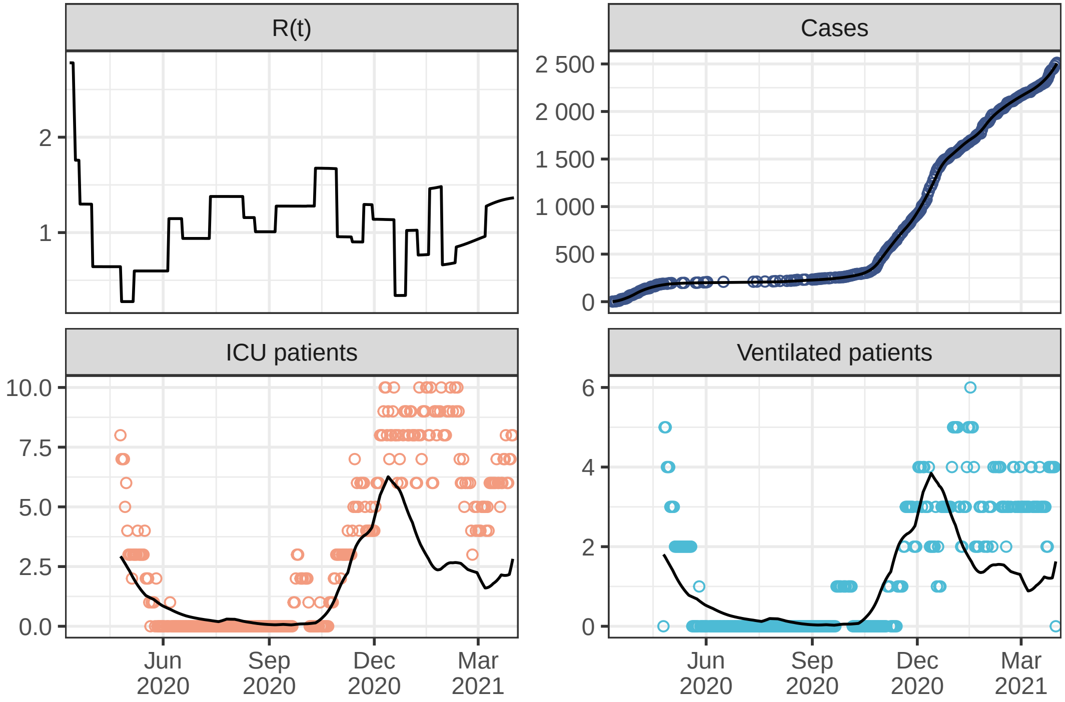

SK Bayreuth

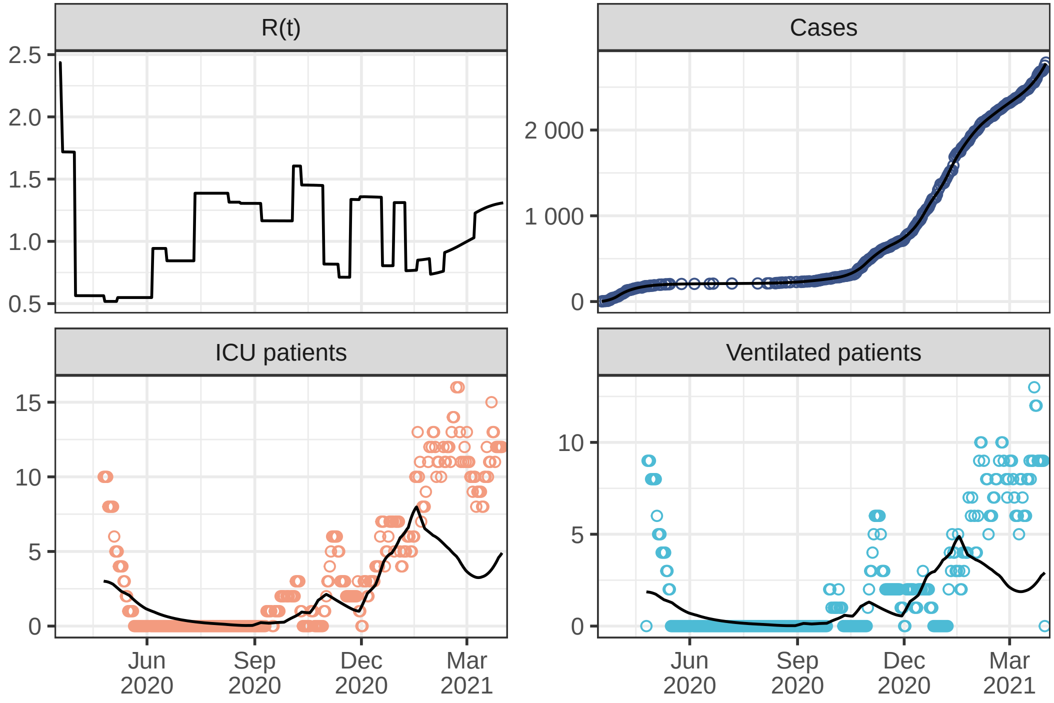

SK Bielefeld

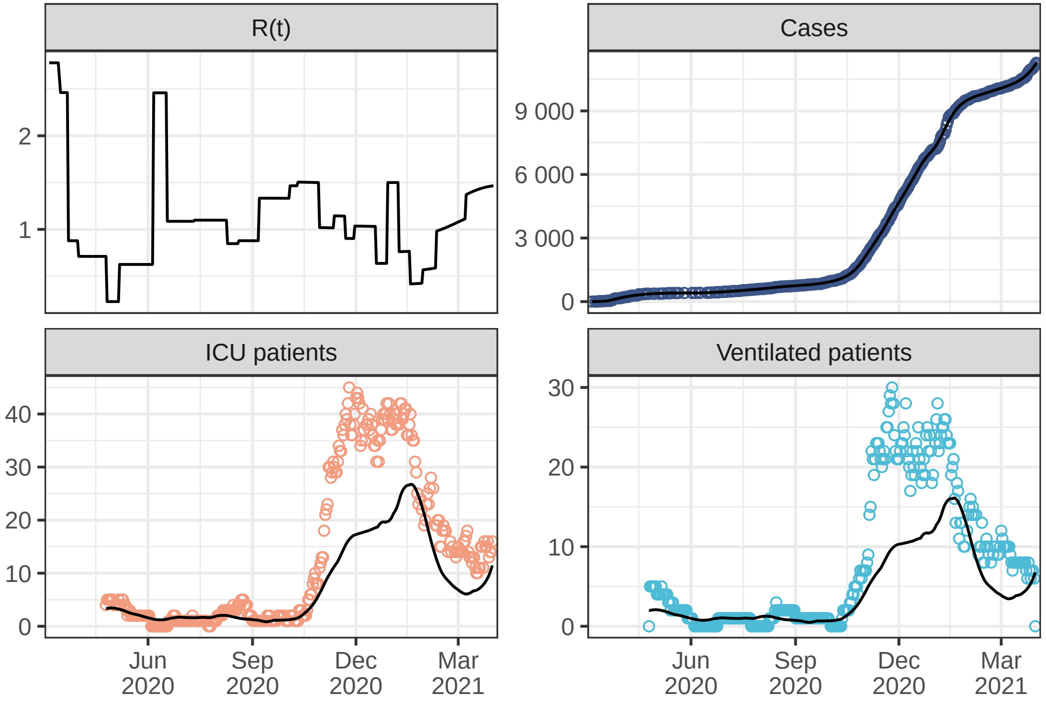

SK Bochum

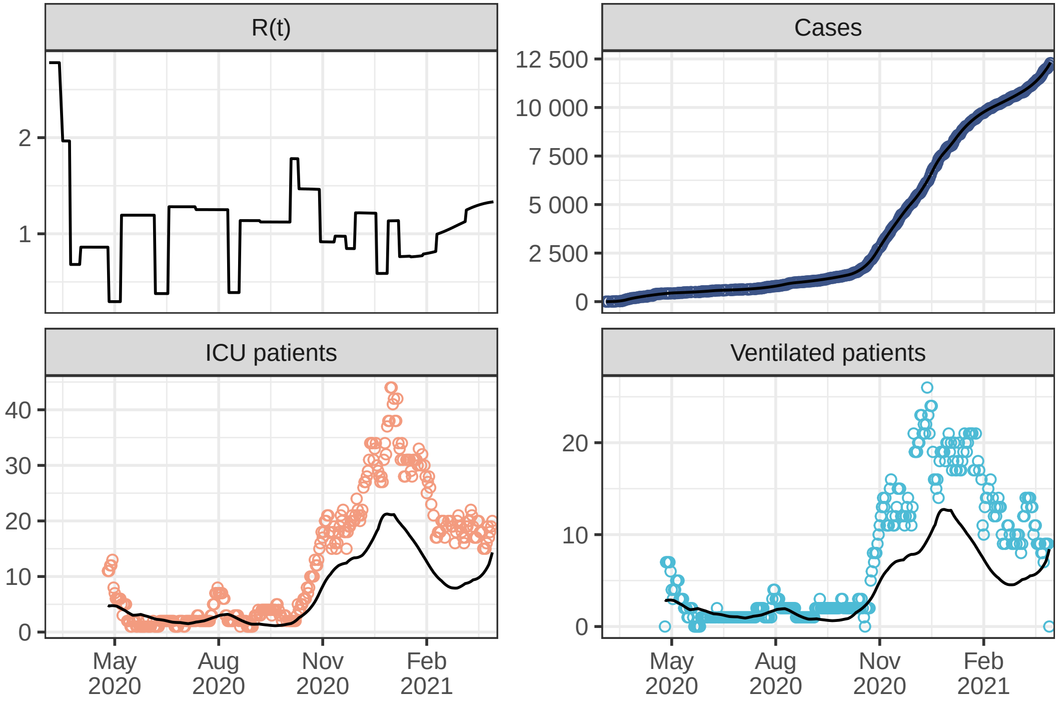

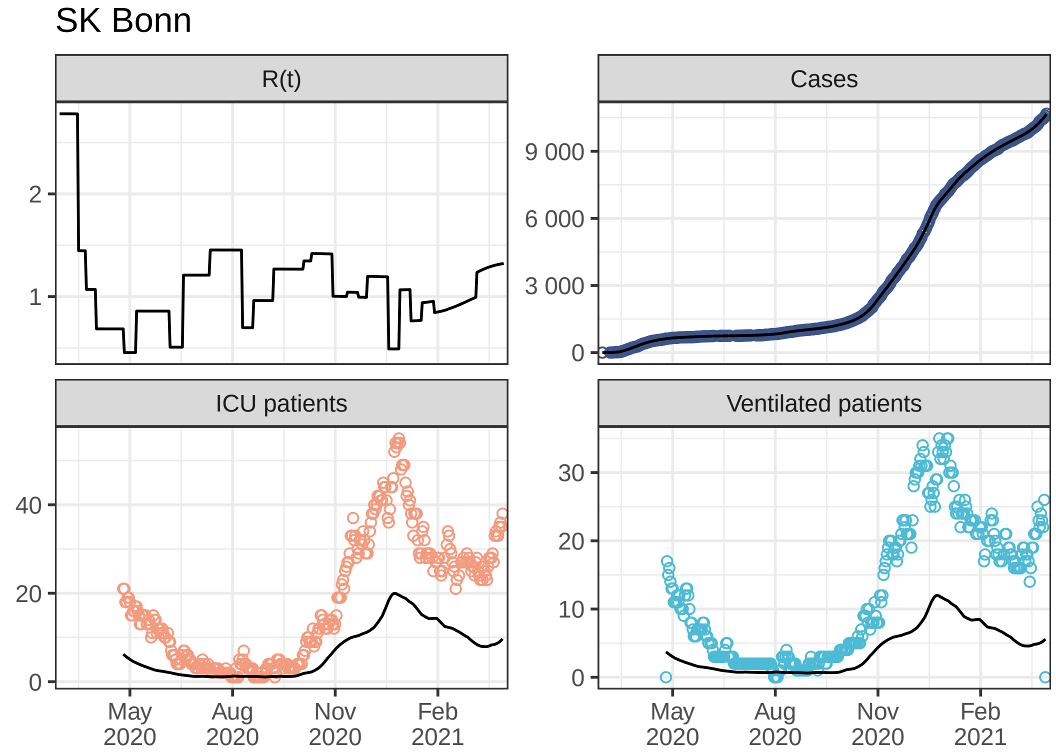
SK Bonn
R(t)
Cases
ICU patients
Ventilated patients
2
1
9 000
6 000
3 000
0
40
20
0
30
20
10
0
May 2020
Aug 2020
Nov 2020
Feb 2021

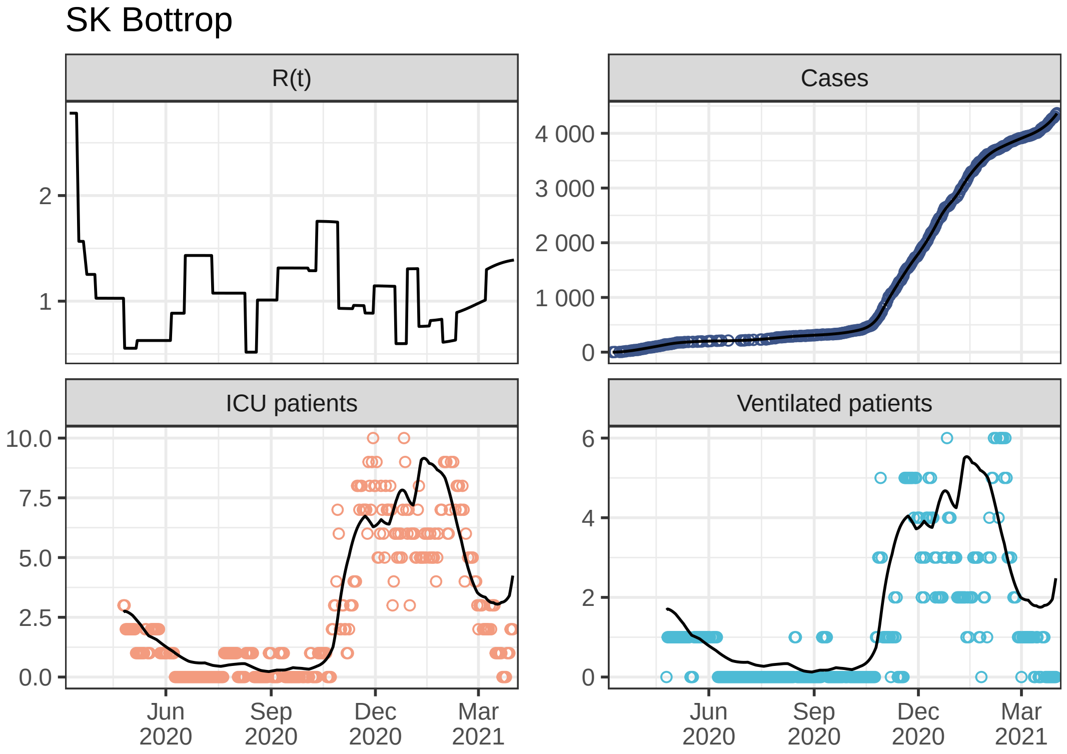
SK Bottrop
R(t)
Cases
ICU patients
Ventilated patients
2
1
4 000
3 000
2 000
1 000
0
10.0
7.5
5.0
2.5
0.0
6
4
2
0
Jun 2020
Sep 2020
Dec 2020
Mar 2021

SK Brandenburg a.d.Havel

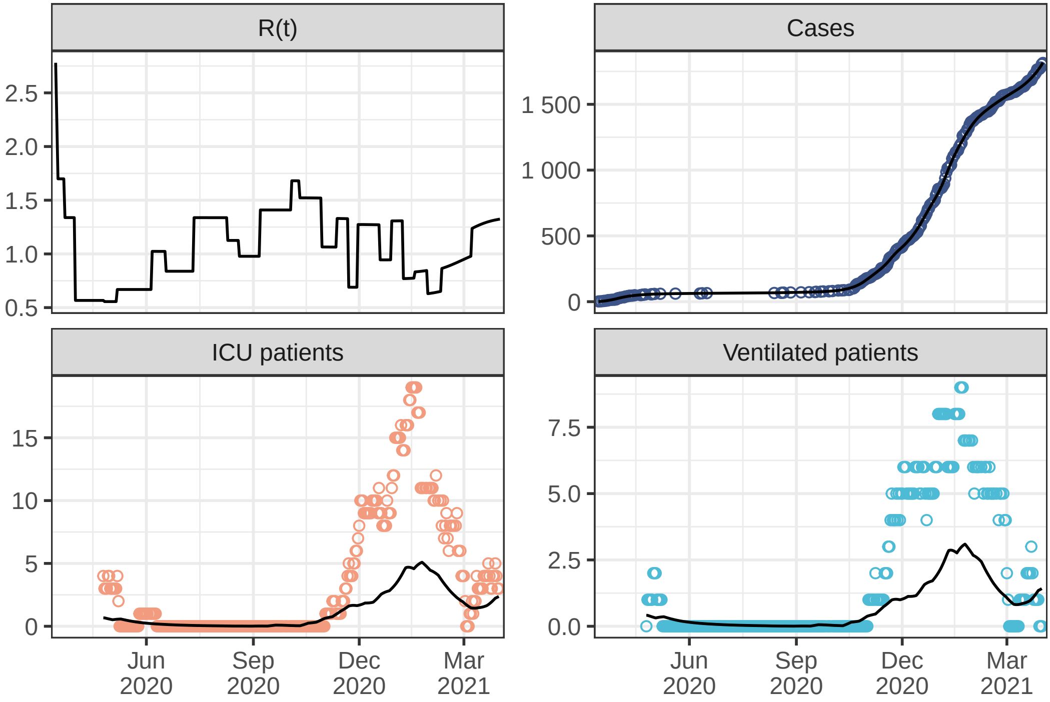

SK Braunschweig

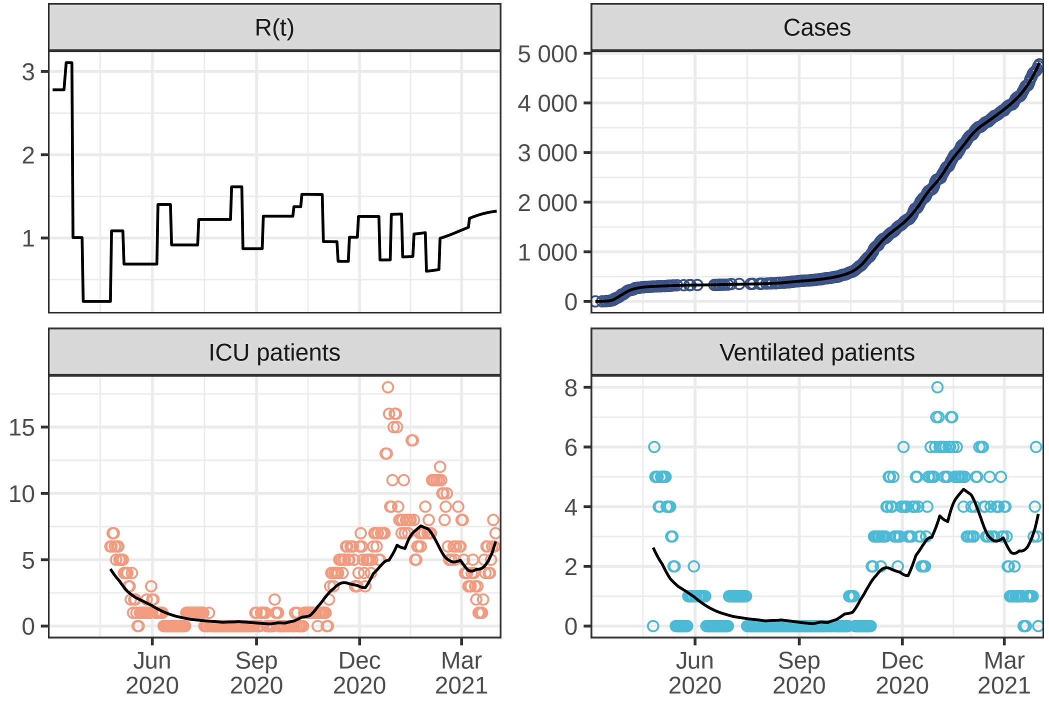

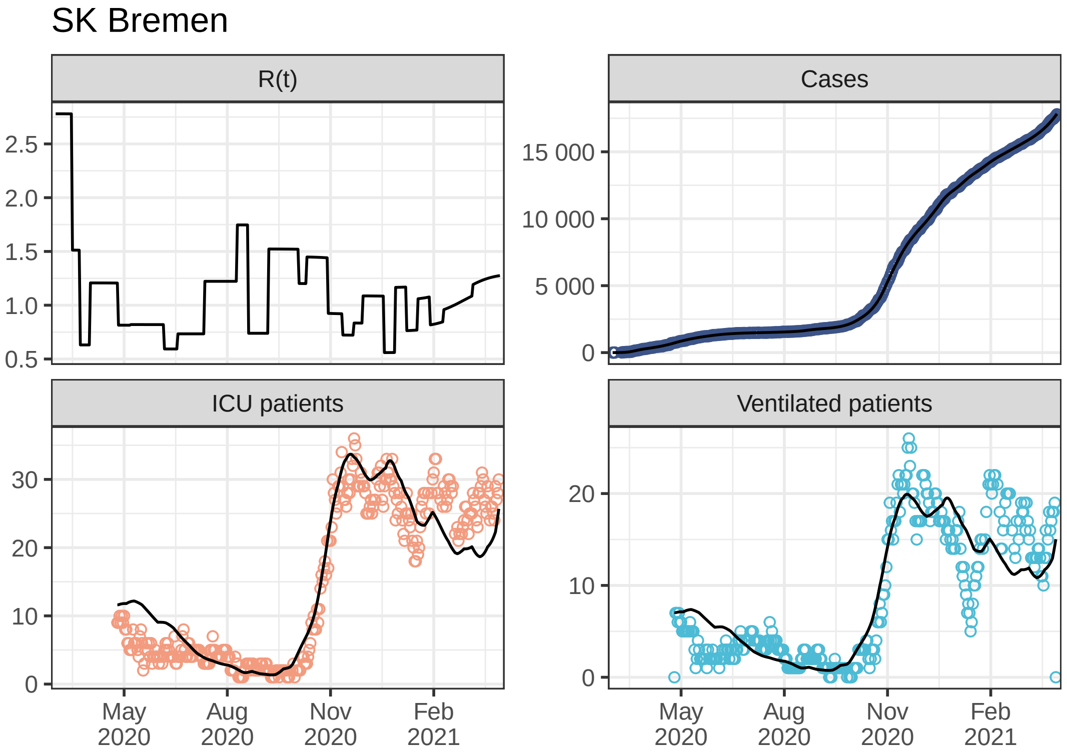
SK Bremen
R(t)
Cases
ICU patients
Ventilated patients
2.5
2.0
1.5
1.0
0.5
15 000
10 000
5 000
0
30
20
10
0
20
10
0
May 2020
Aug 2020
Nov 2020
Feb 2021

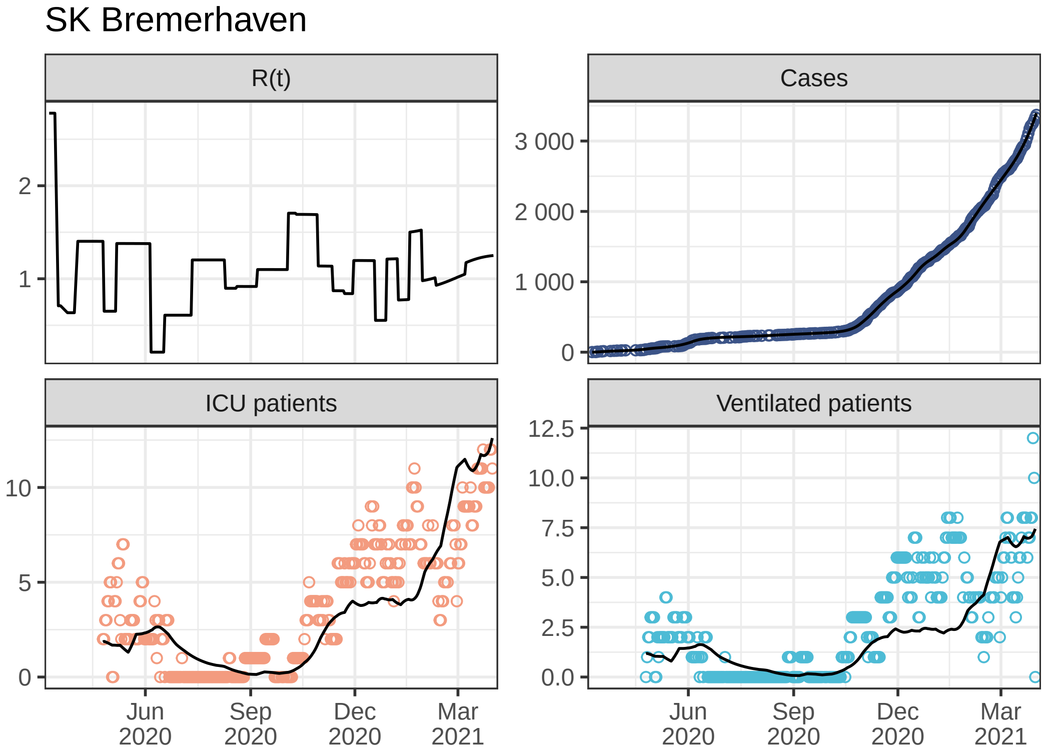
SK Bremerhaven
R(t)
Cases
ICU patients
Ventilated patients
2
1
3 000
2 000
1 000
0
10
5
0
12.5
10.0
7.5
5.0
2.5
0.0
Jun 2020
Sep 2020
Dec 2020
Mar 2021

SK Chemnitz

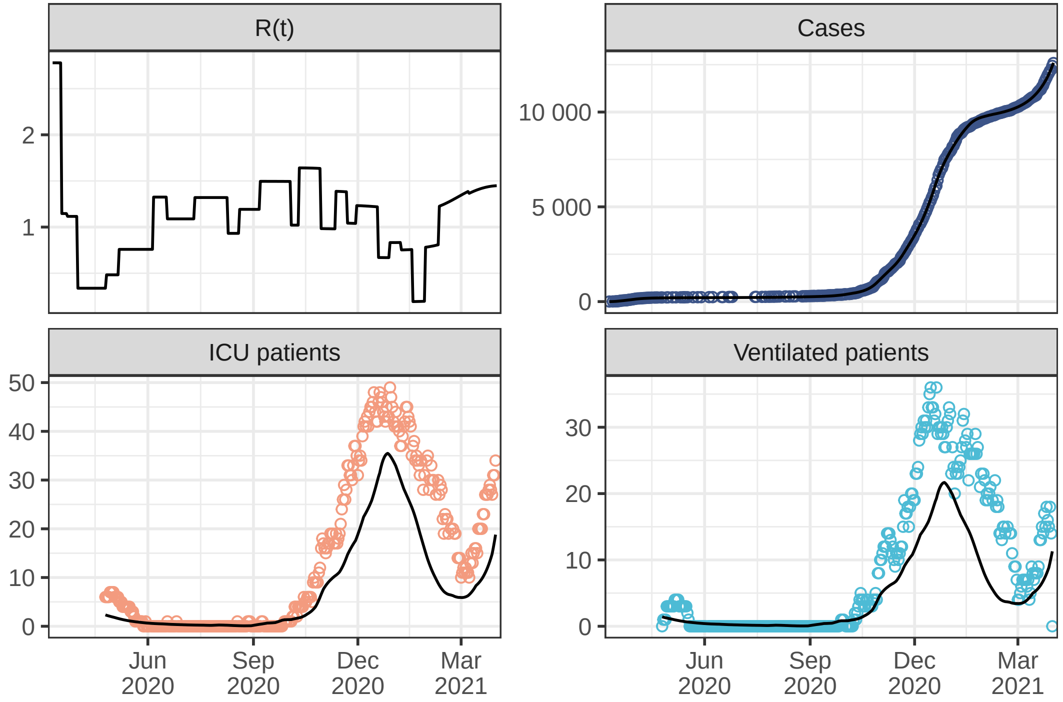

SK Coburg

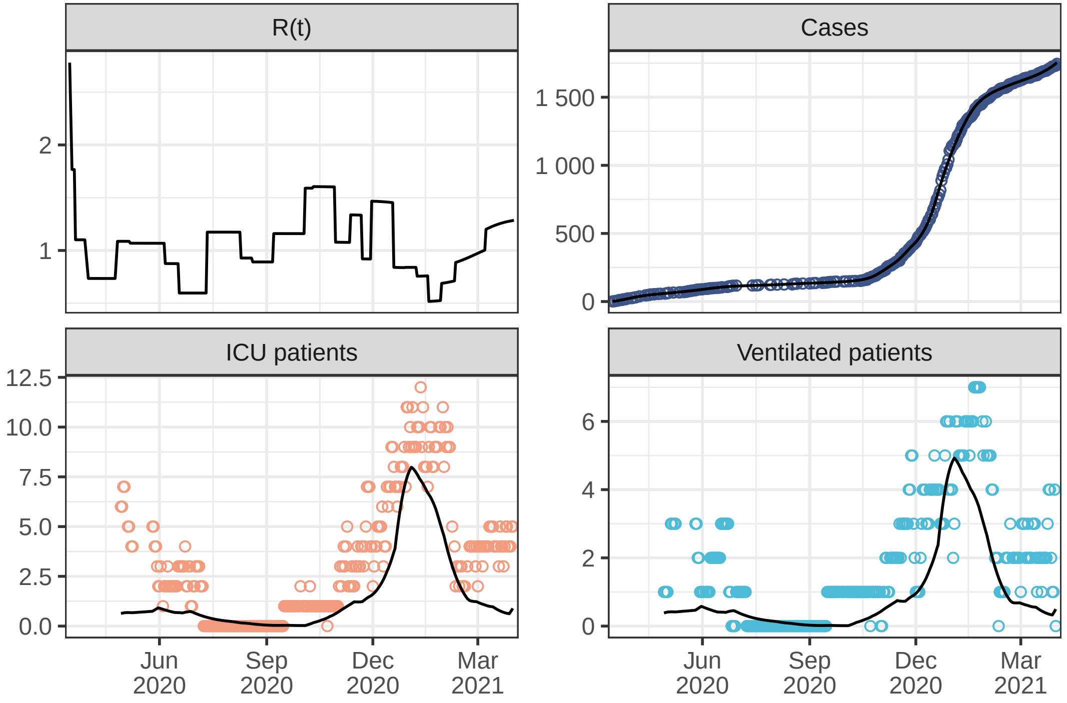

SK Cottbus

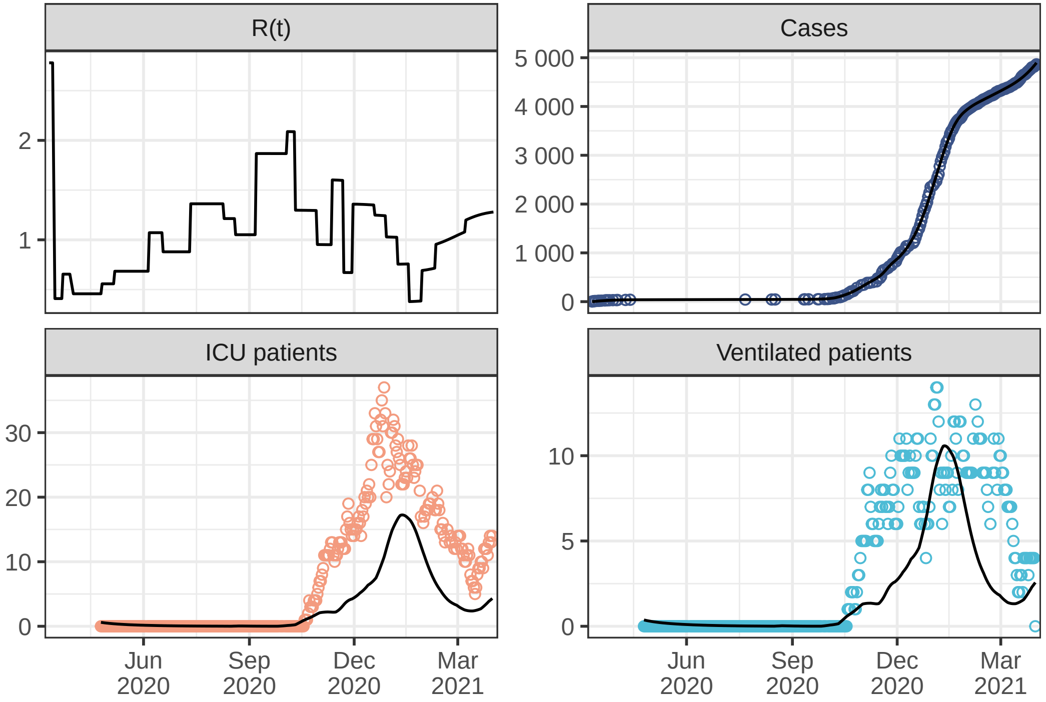

SK Darmstadt

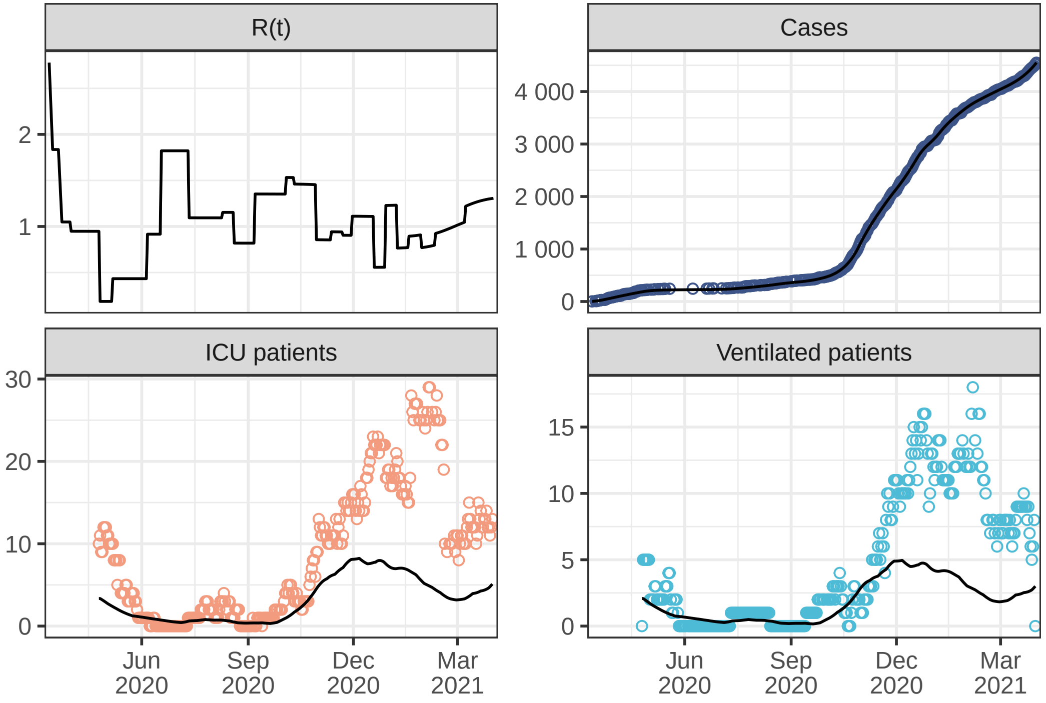

SK Delmenhorst

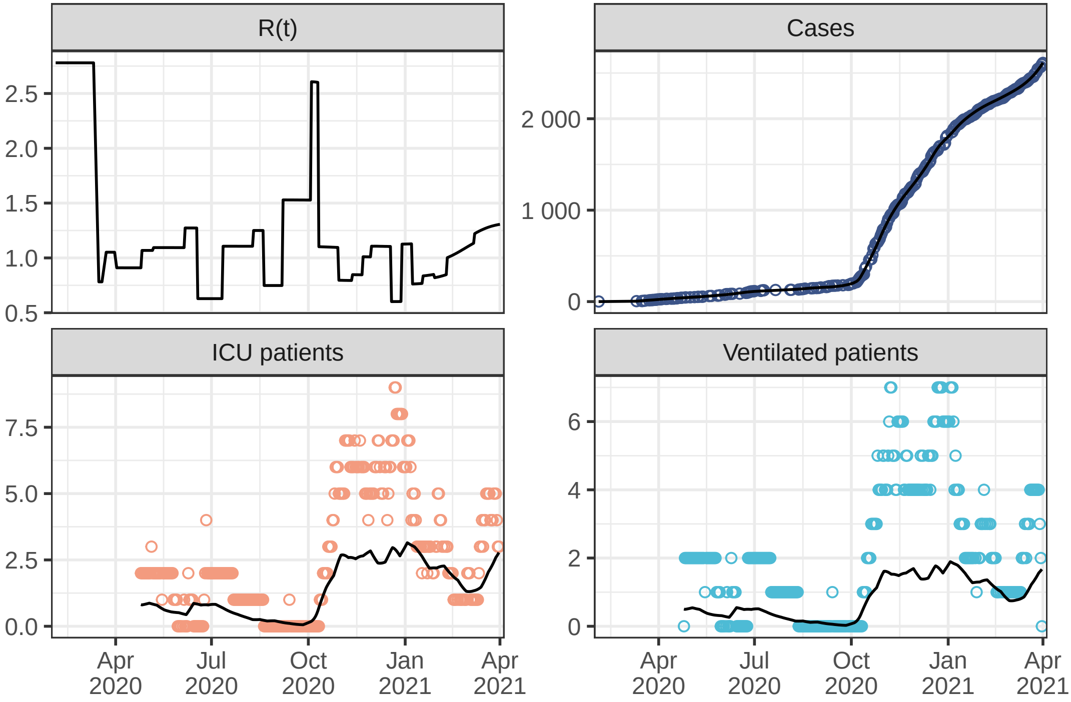

SK Dessau−Roßlau

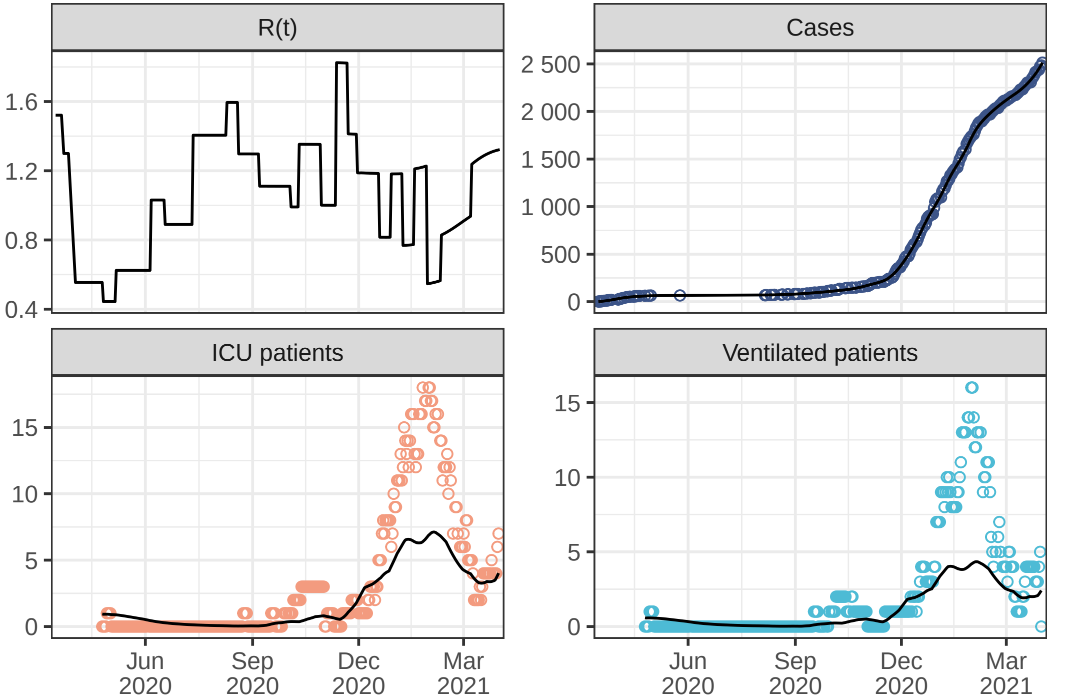

SK Dortmund

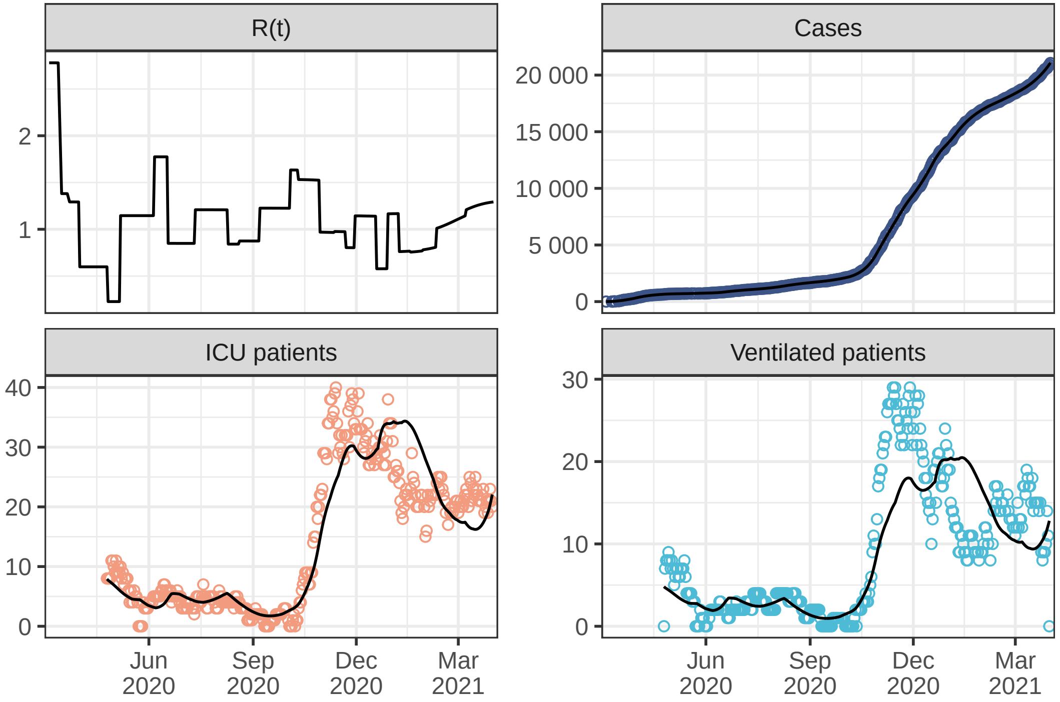

SK Dresden

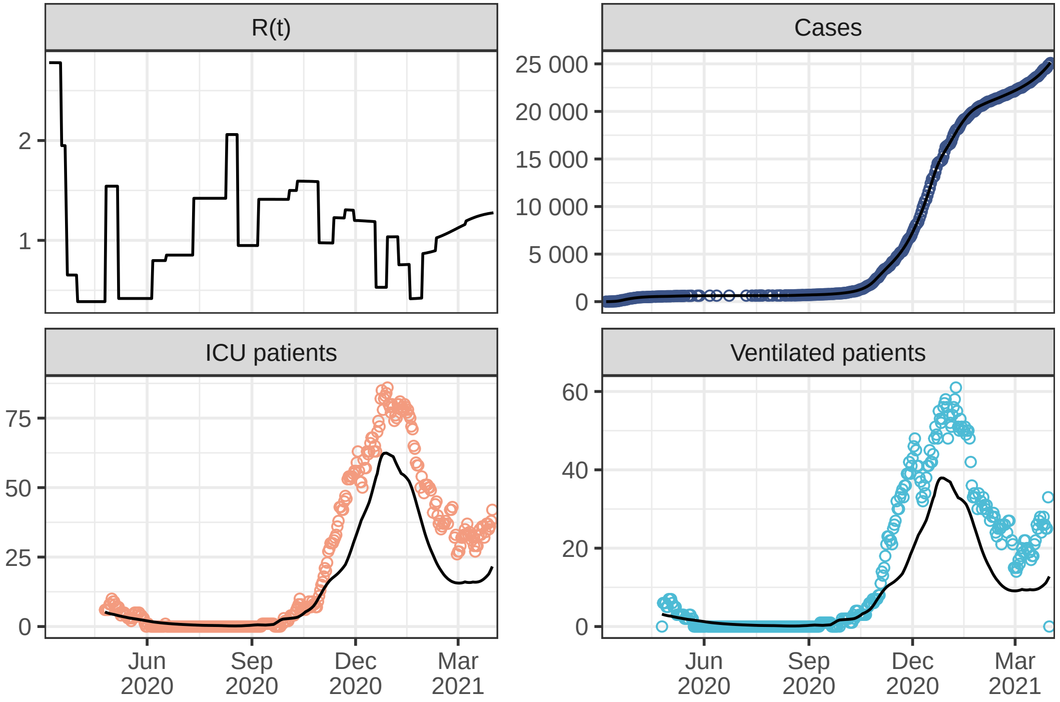

SK Duisburg

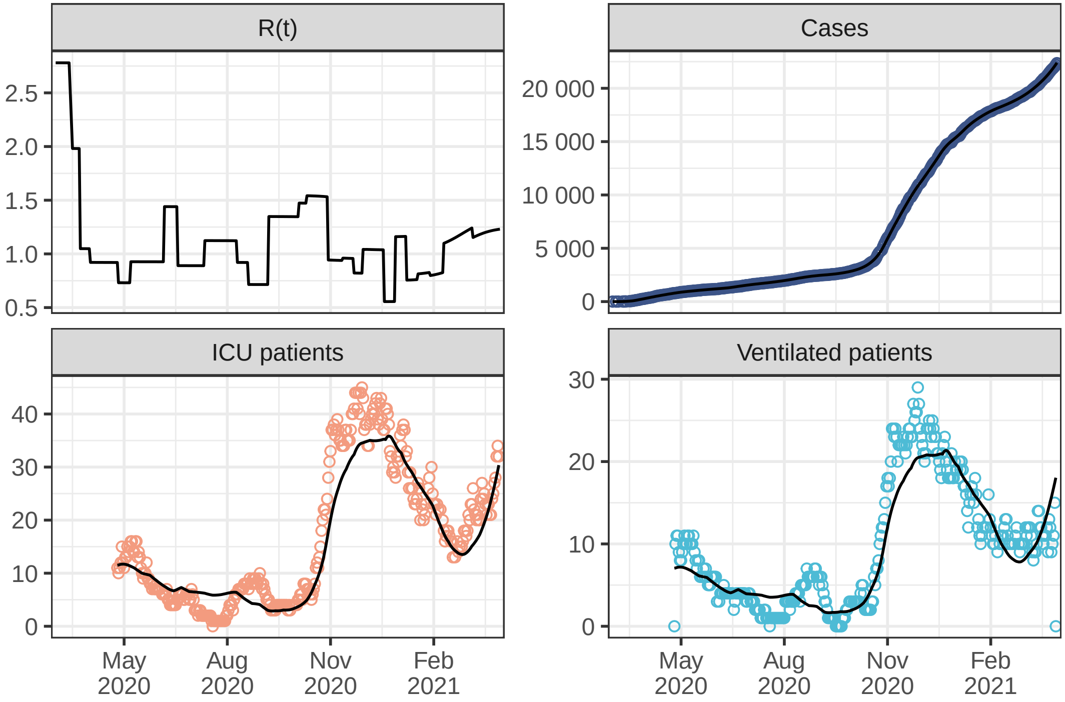

SK Düsseldorf

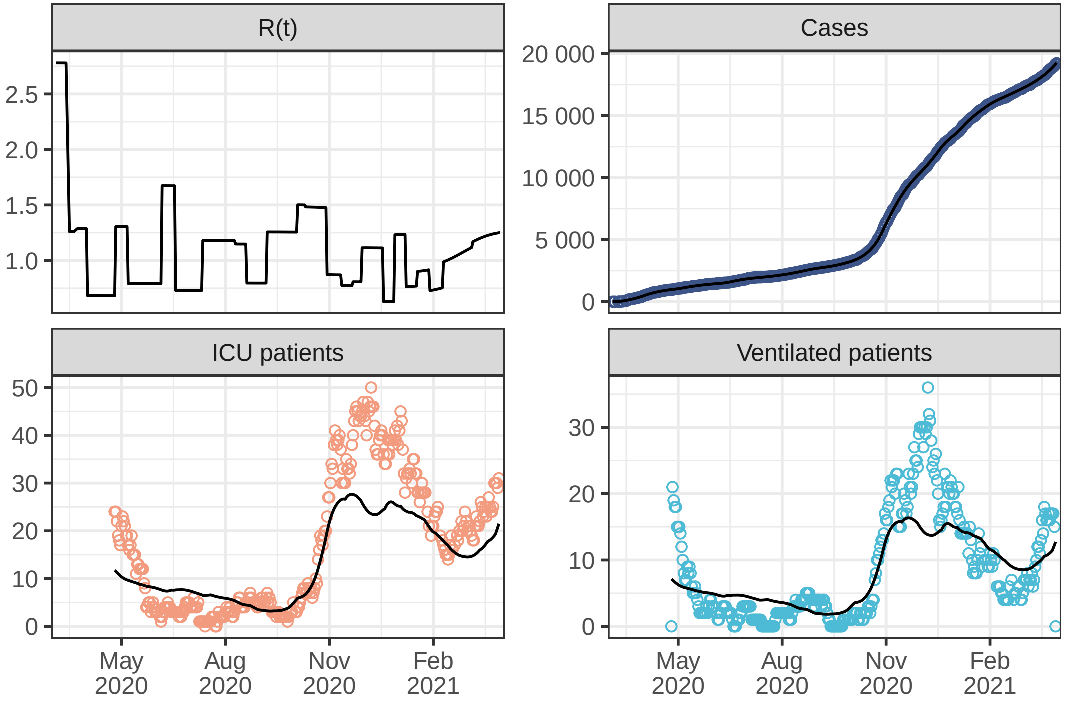

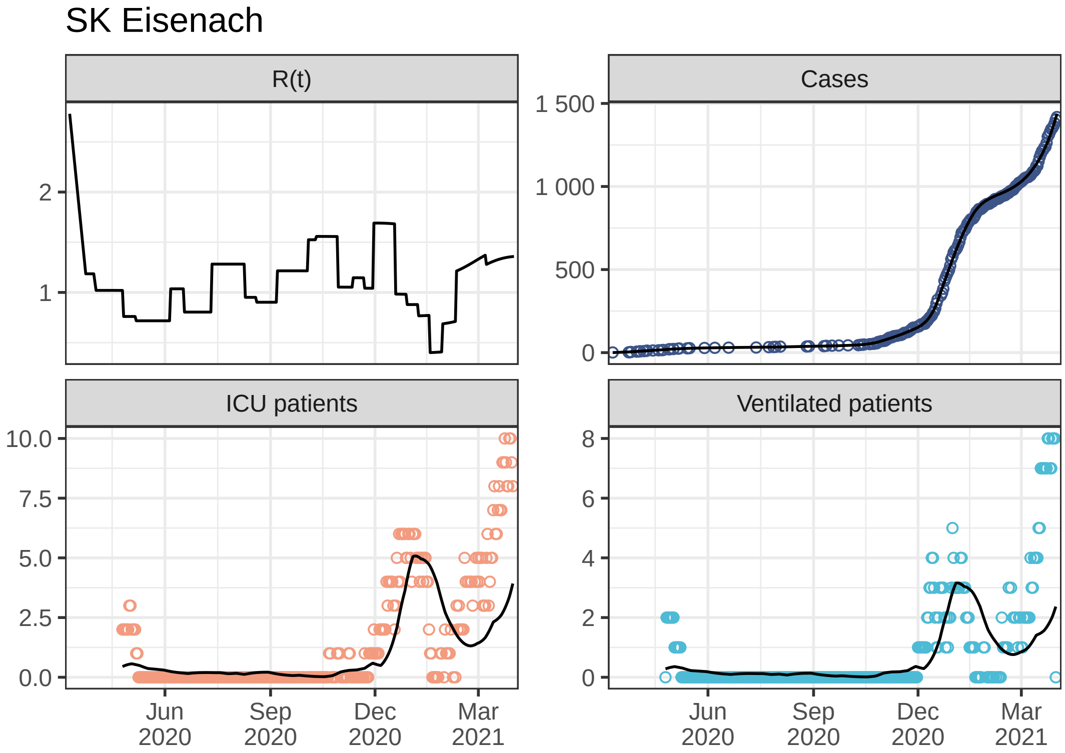
SK Eisenach
R(t)
Cases
ICU patients
Ventilated patients
1
2
0
500
1 000
1 500
0.0
2.5
5.0
7.5
10.0
0
2
4
6
8
Jun 2020
Sep 2020
Dec 2020
Mar 2021

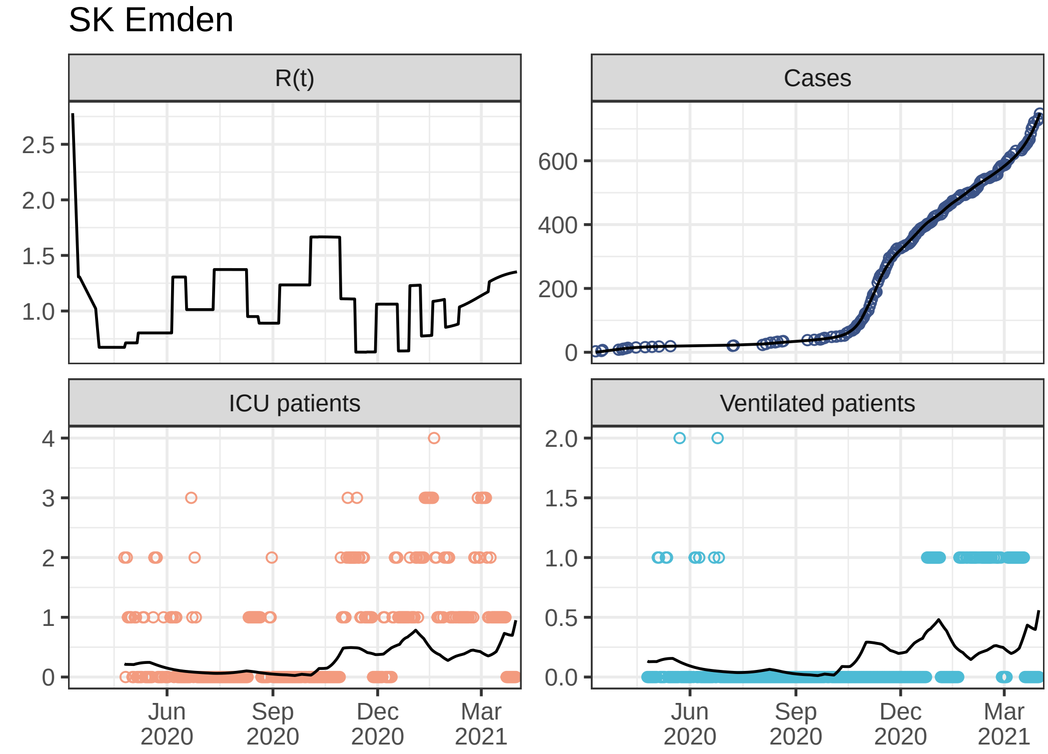
SK Emden
R(t)
Cases
ICU patients
Ventilated patients
1.0
1.5
2.0
2.5
0
200
400
600
0
1
2
3
4
0.0
0.5
1.0
1.5
2.0
Jun 2020
Sep 2020
Dec 2020
Mar 2021

SK Erfurt

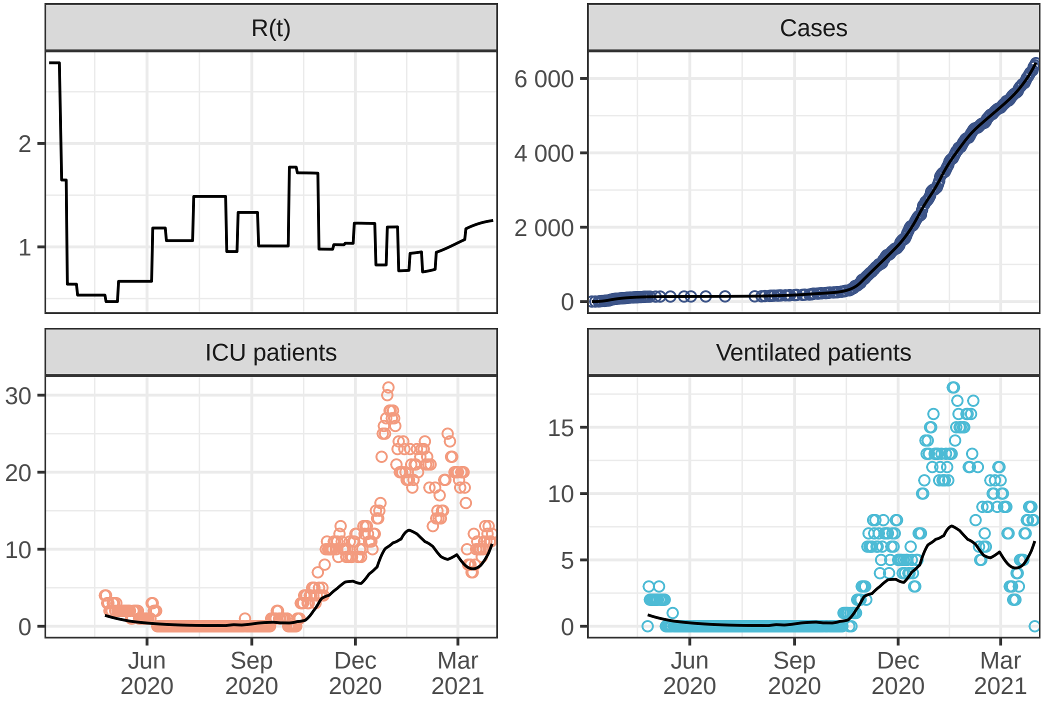

SK Erlangen

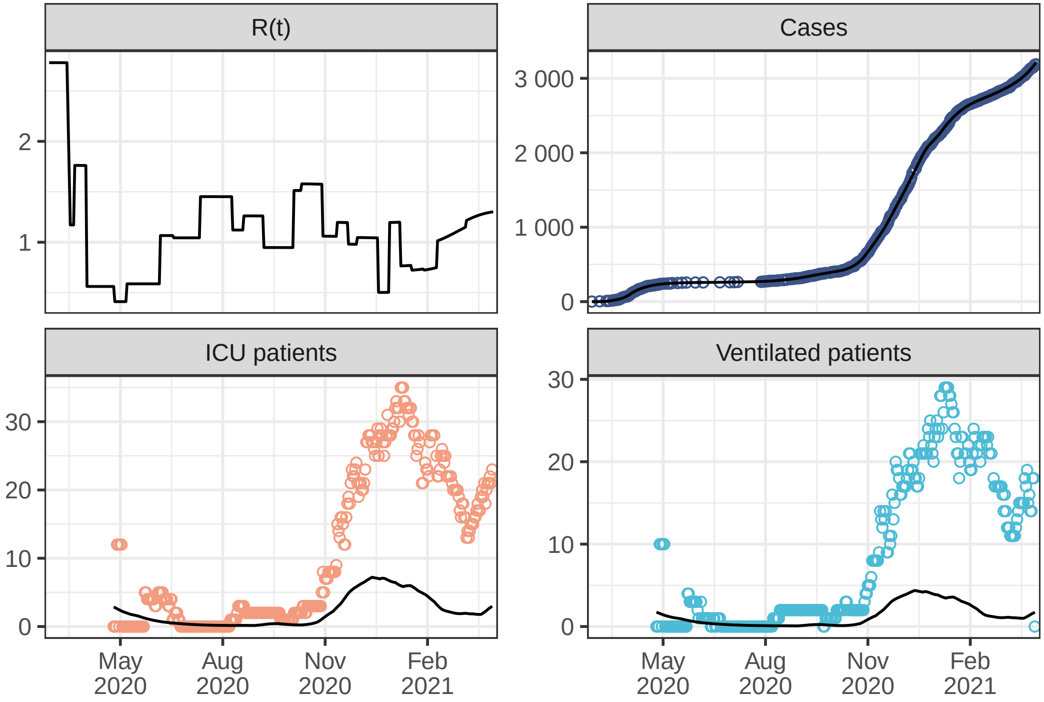

SK Essen

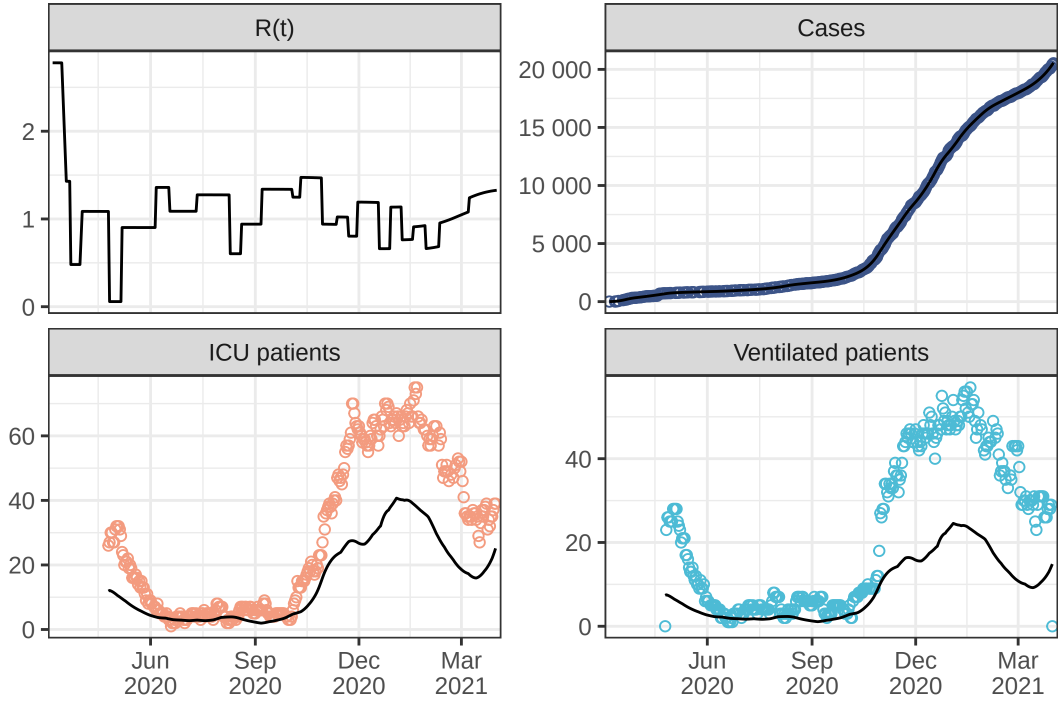

SK Flensburg

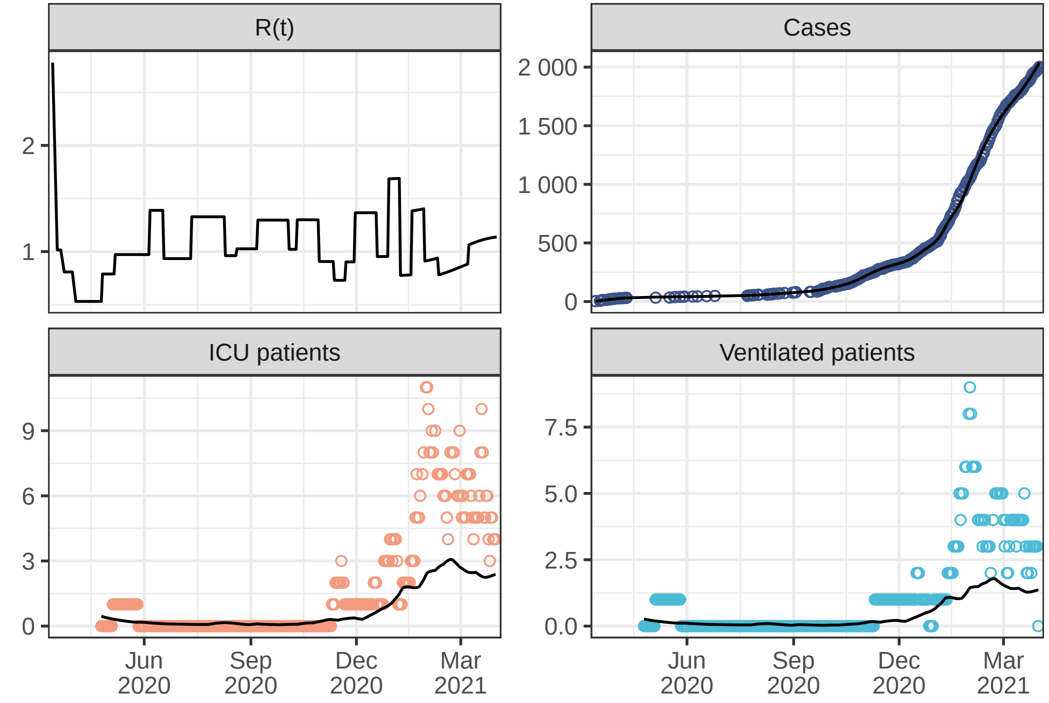

SK Frankenthal

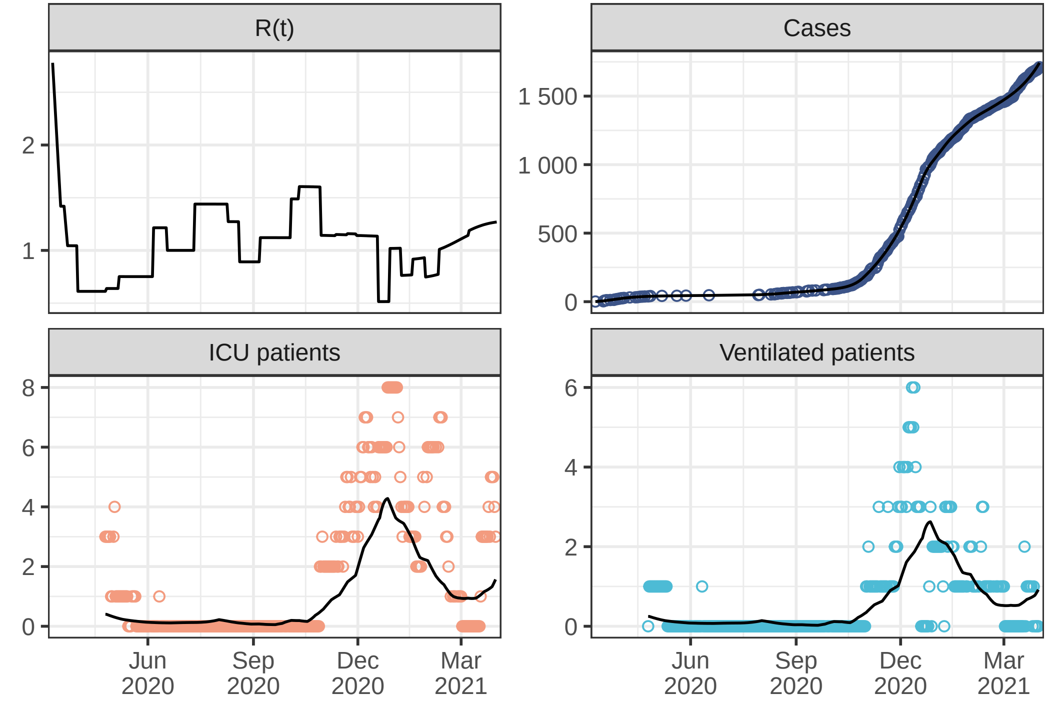

SK Frankfurt (Oder)

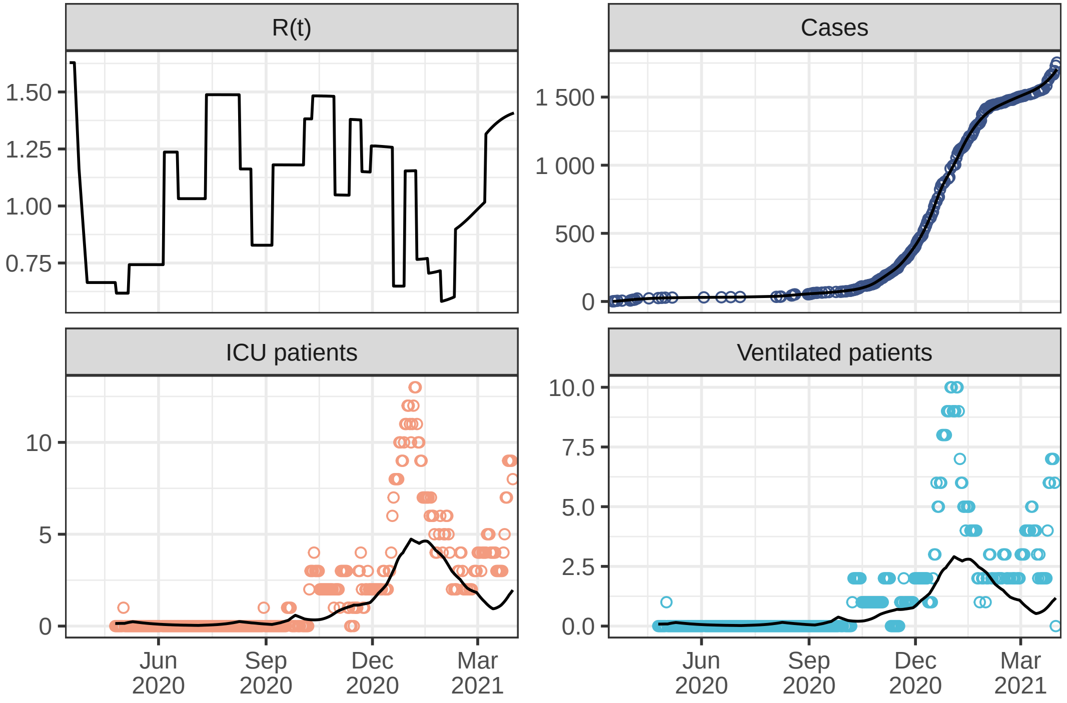

SK Frankfurt am Main

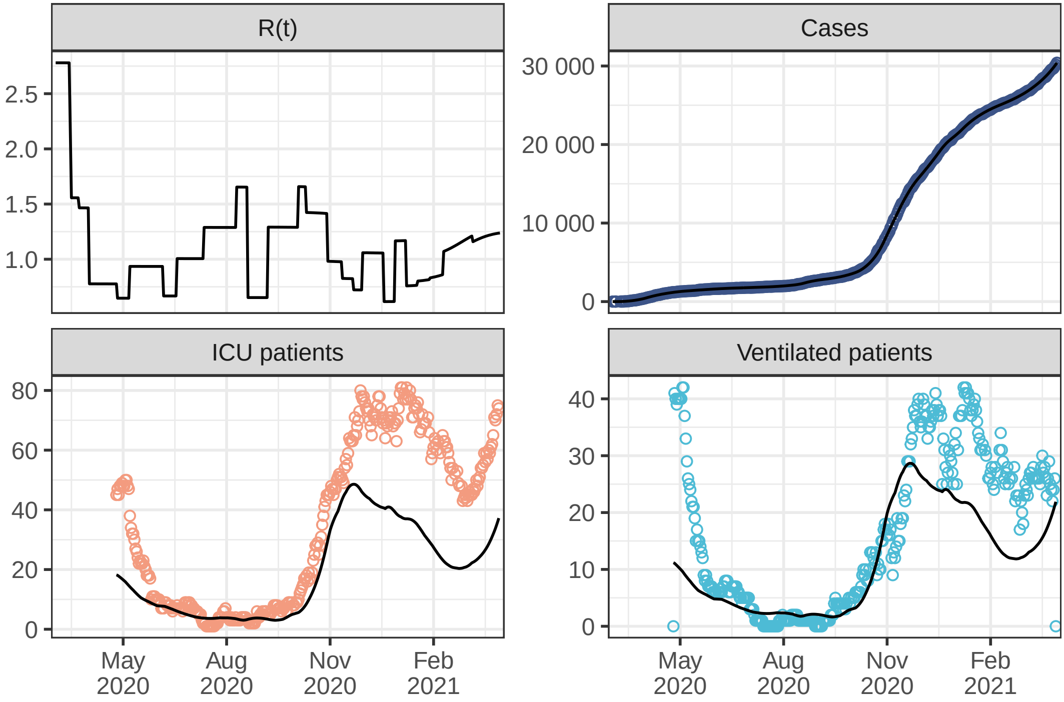

SK Freiburg i.Breisgau

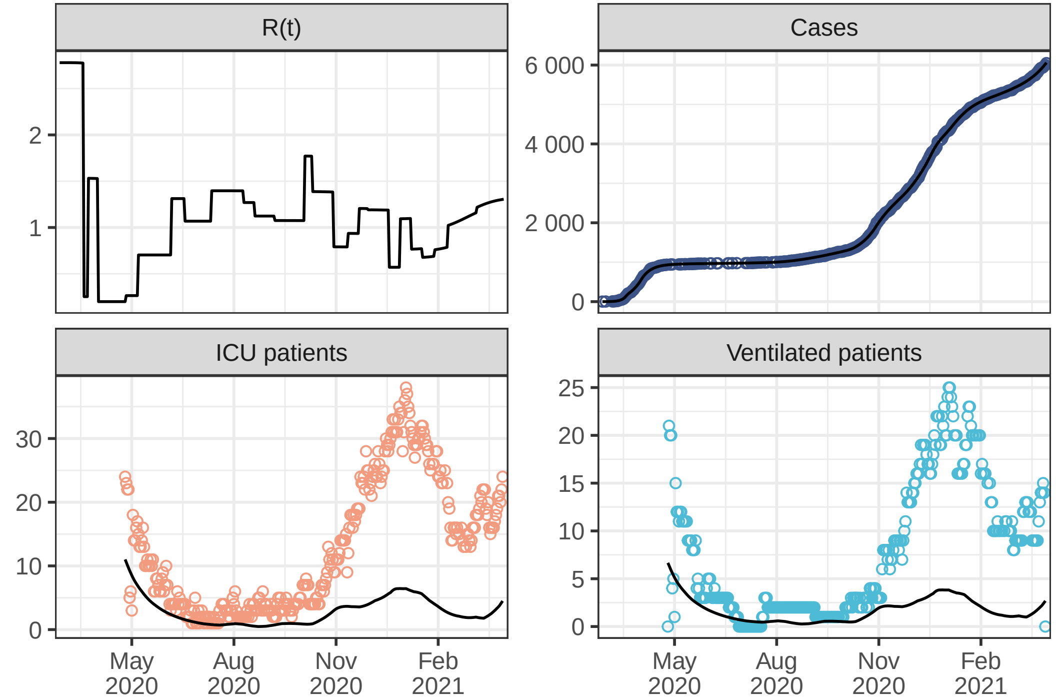

SK Fürth

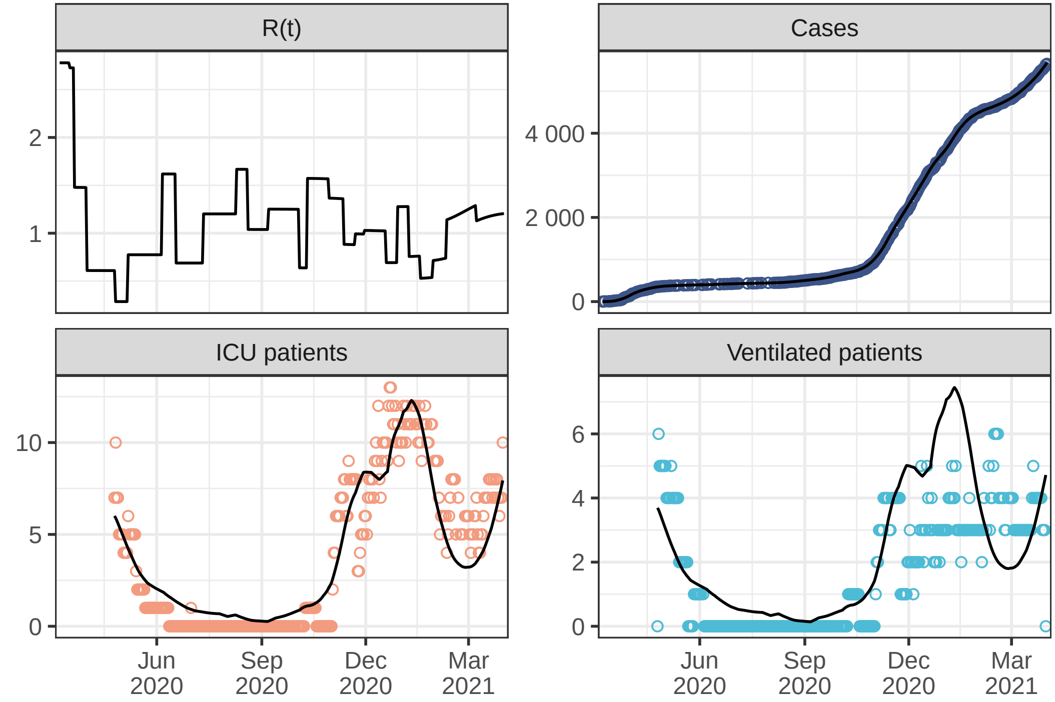

SK Gelsenkirchen

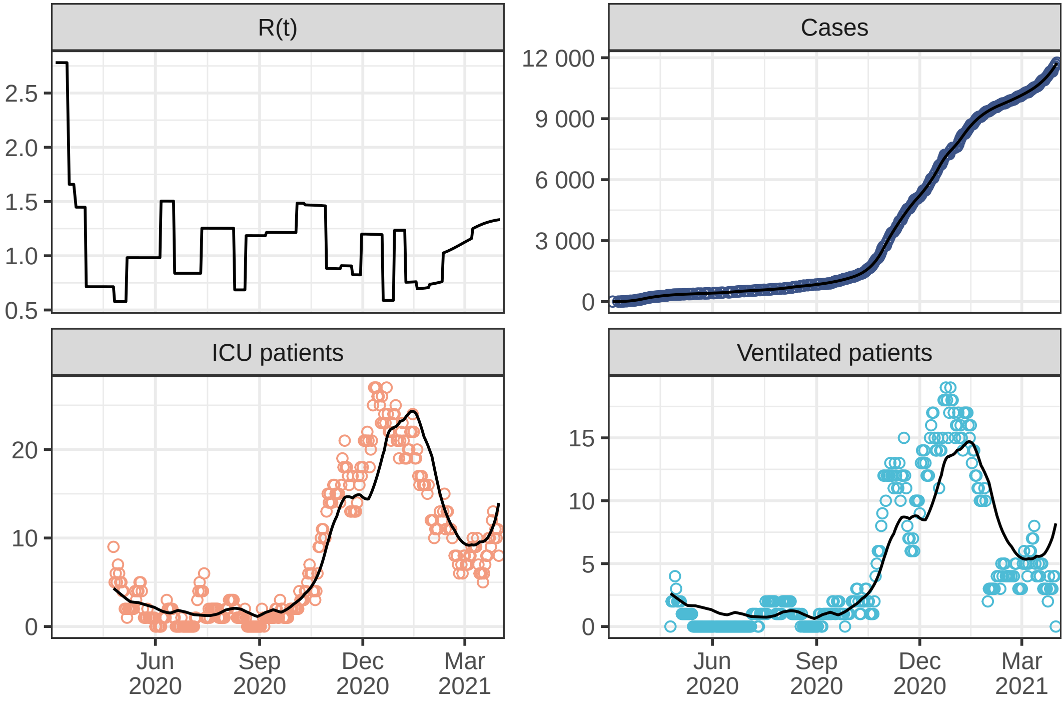

SK Gera

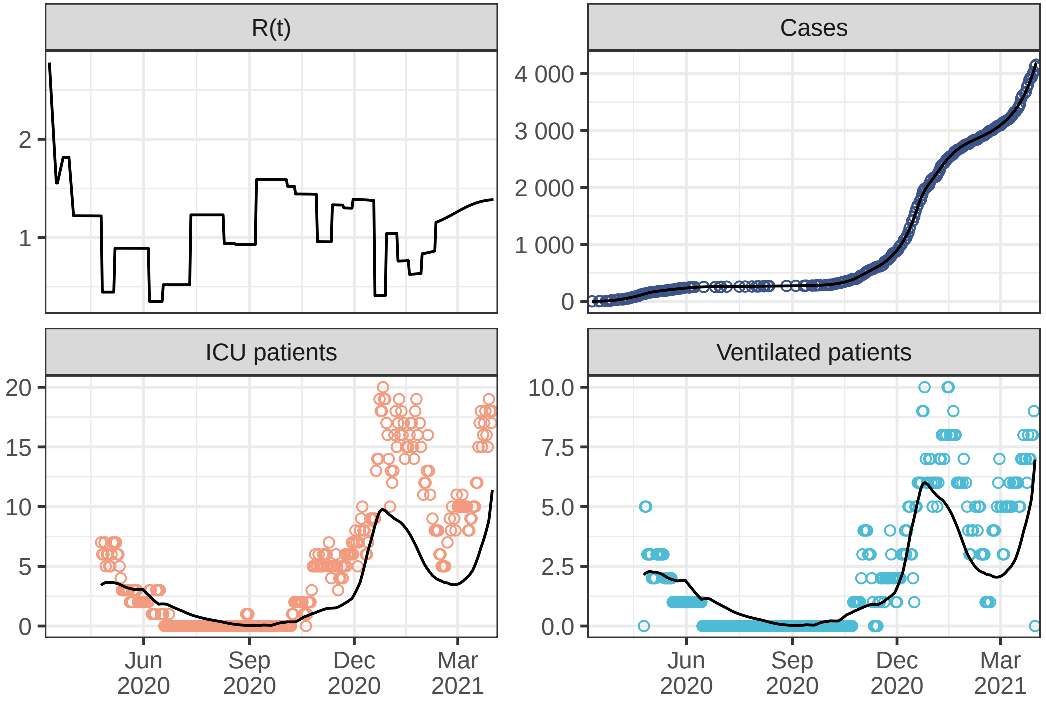

SK Hagen

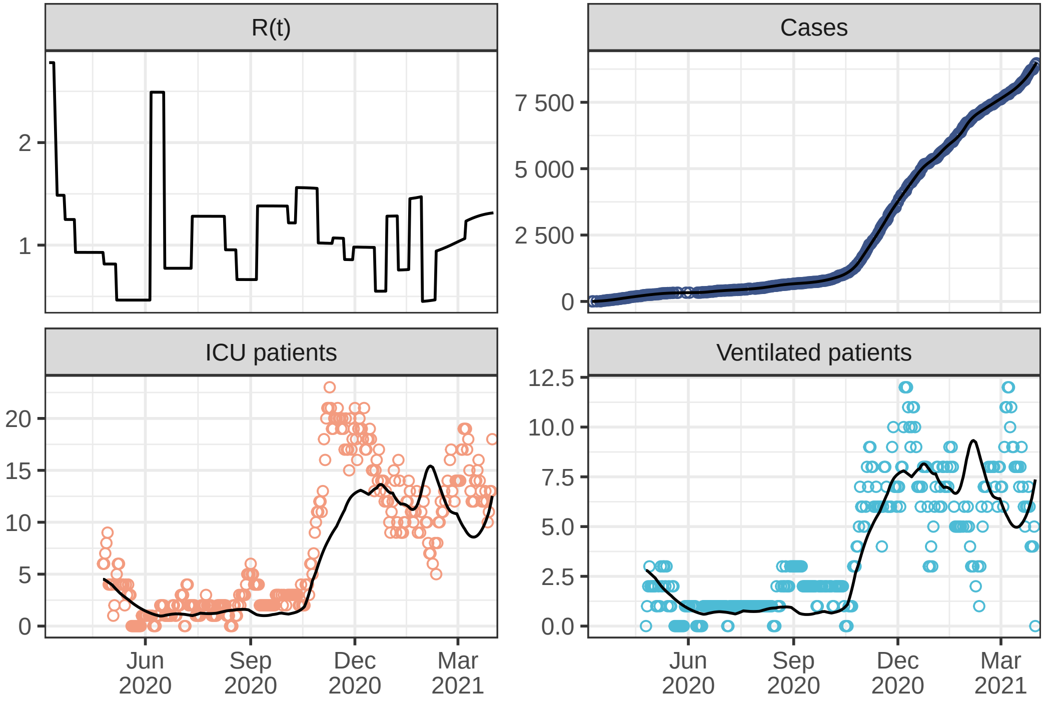

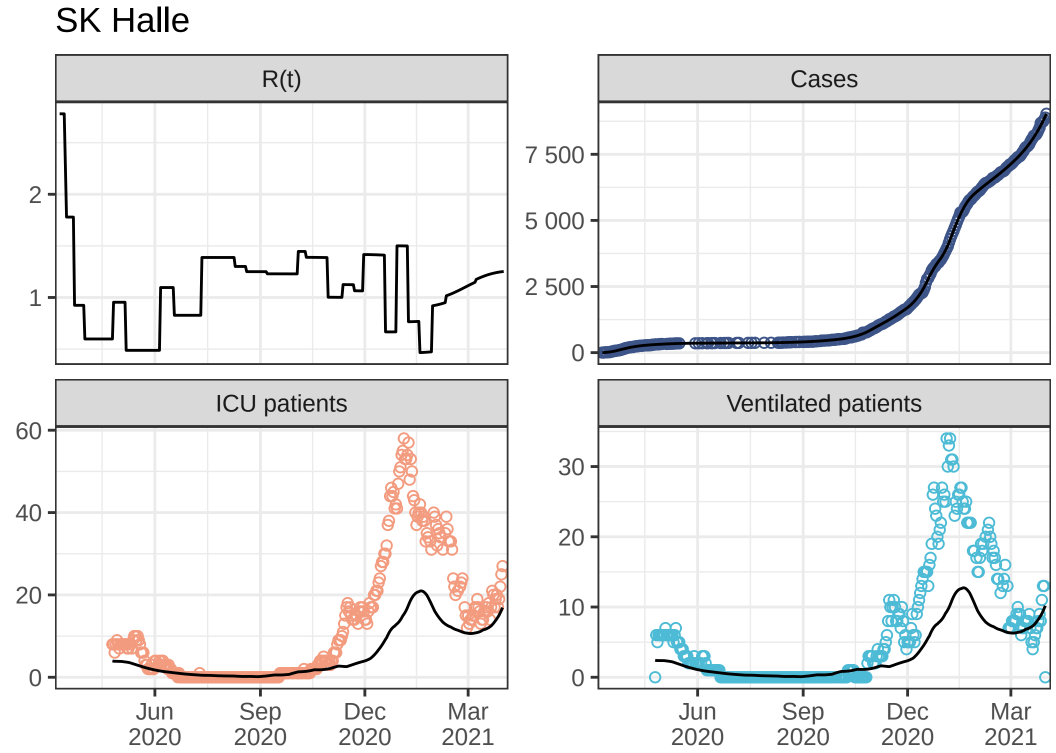
SK Halle
R(t)
Cases
ICU patients
Ventilated patients
2
1
7 500
5 000
2 500
0
60
40
20
0
30
20
10
0
Jun 2020
Sep 2020
Dec 2020
Mar 2021

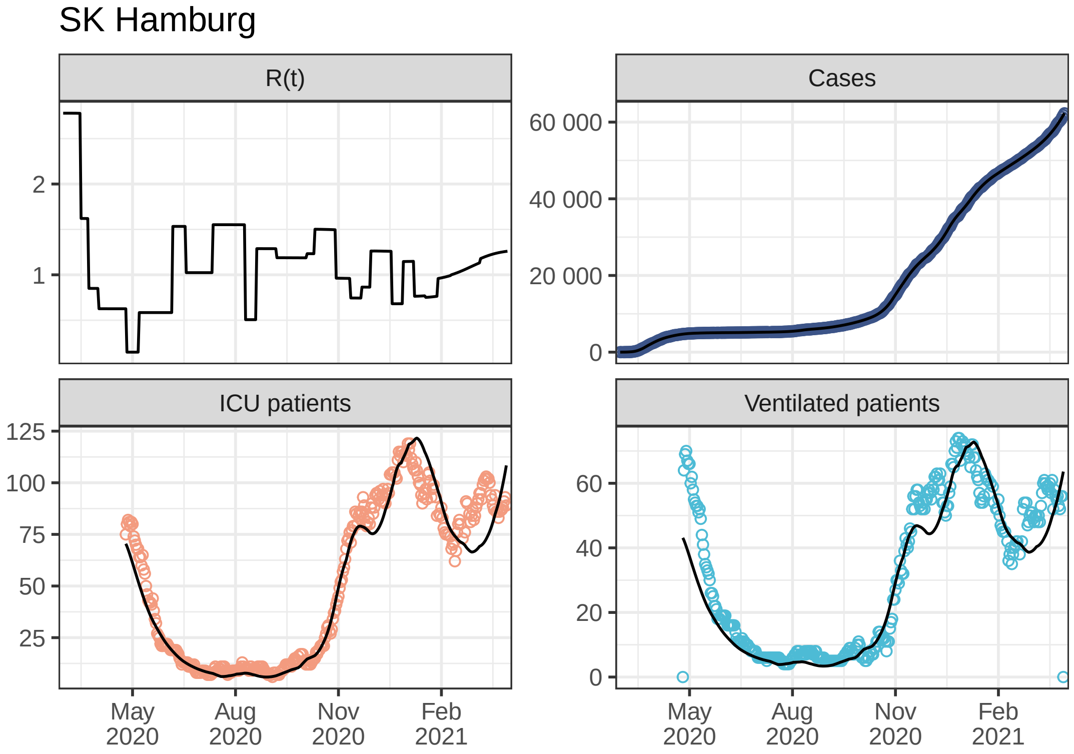
SK Hamburg
R(t)
Cases
ICU patients
Ventilated patients
2
1
60 000
40 000
20 000
0
125
100
75
50
25
60
40
20
0
May 2020
Aug 2020
Nov 2020
Feb 2021

SK Hamm

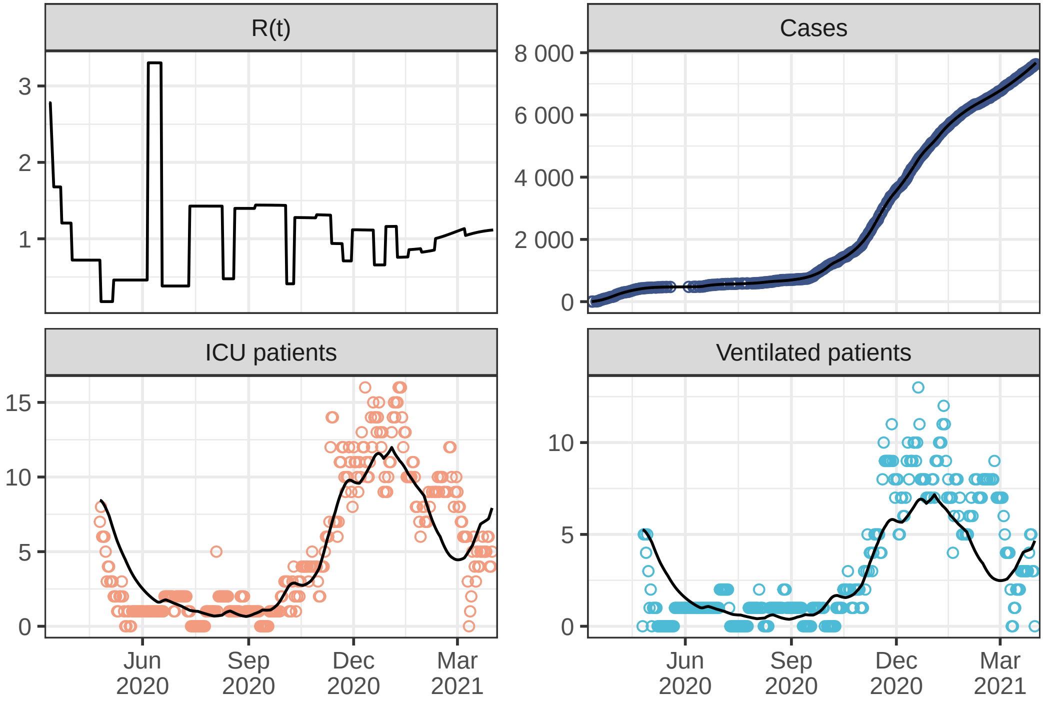

SK Heidelberg

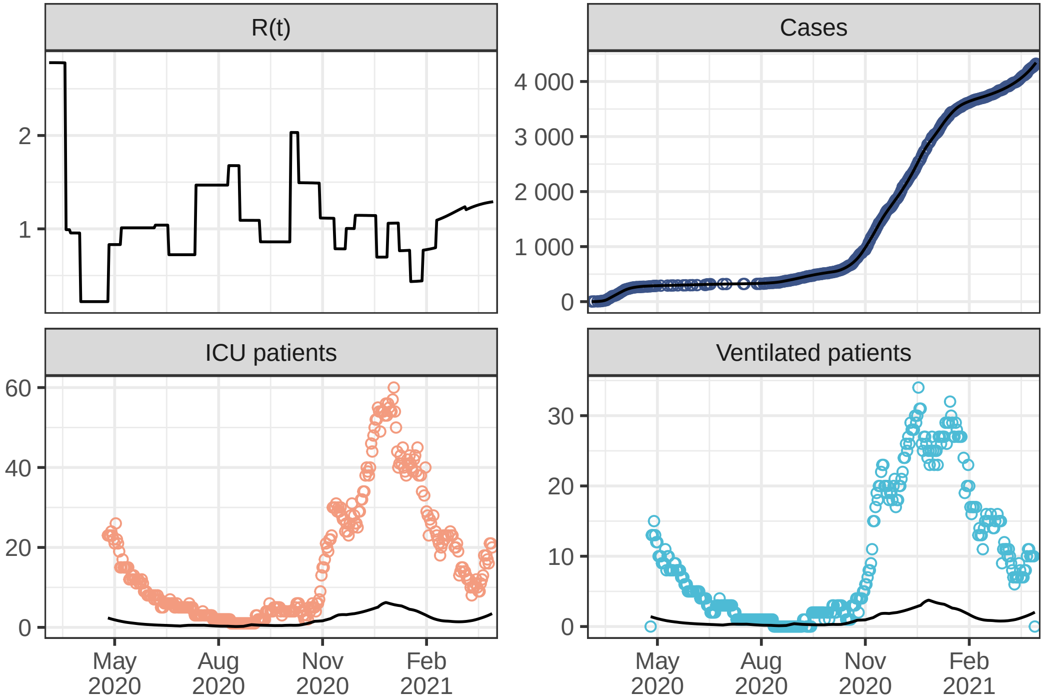

SK Heilbronn

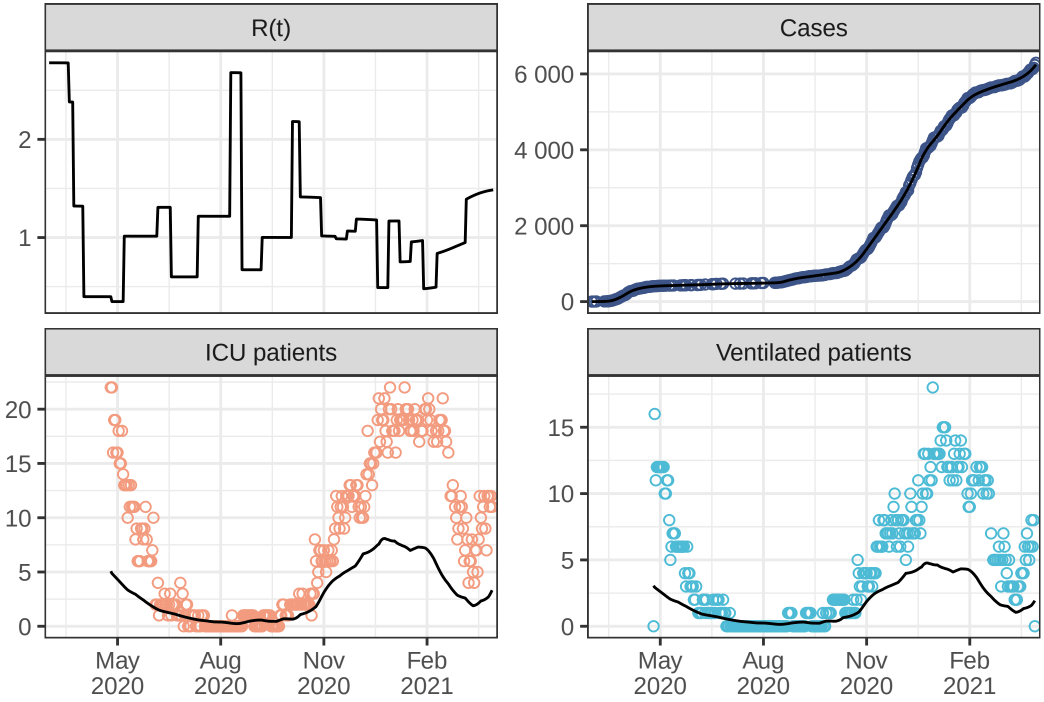

SK Herne

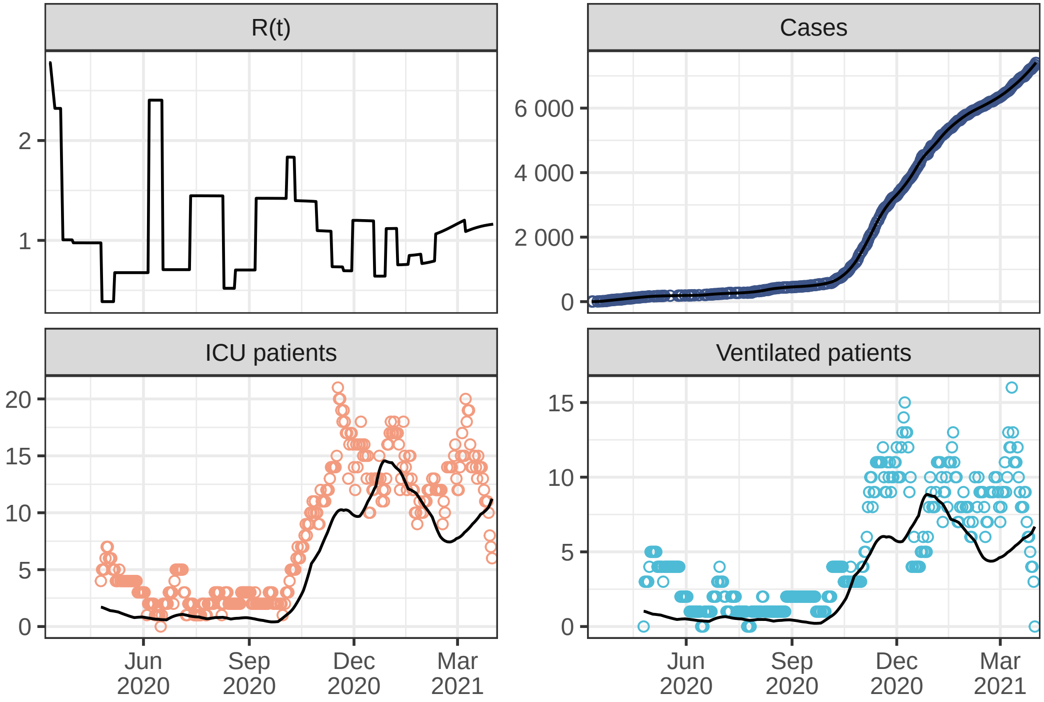

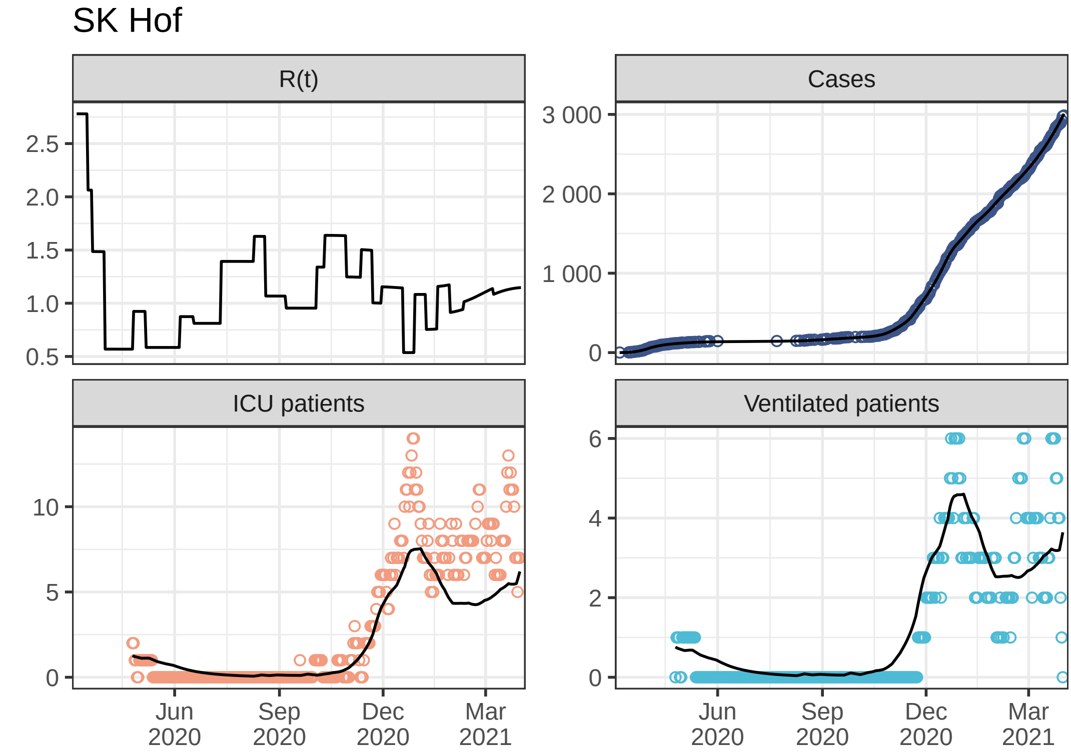
SK Hof
R(t)
Cases
ICU patients
Ventilated patients
2.5
2.0
1.5
1.0
0.5
3 000
2 000
1 000
0
10
5
0
6
4
2
0
Jun 2020
Sep 2020
Dec 2020
Mar 2021

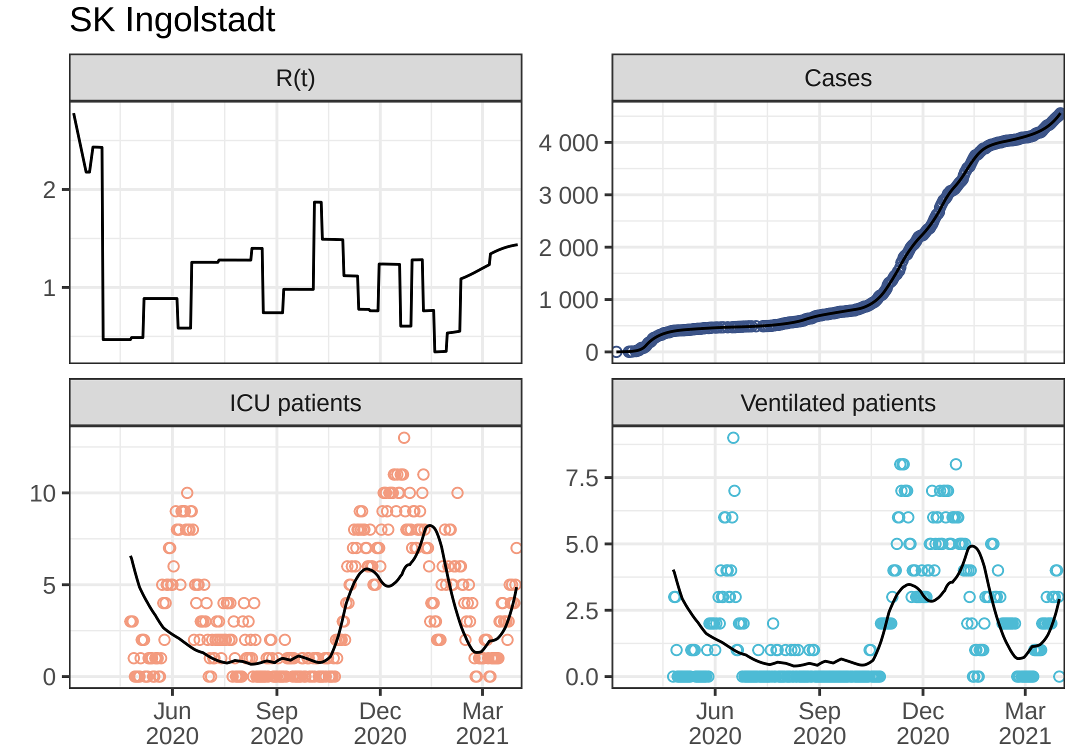
SK Ingolstadt
R(t)
Cases
ICU patients
Ventilated patients
2
1
4 000
3 000
2 000
1 000
0
10
5
0
7.5
5.0
2.5
0.0
Jun 2020
Sep 2020
Dec 2020
Mar 2021

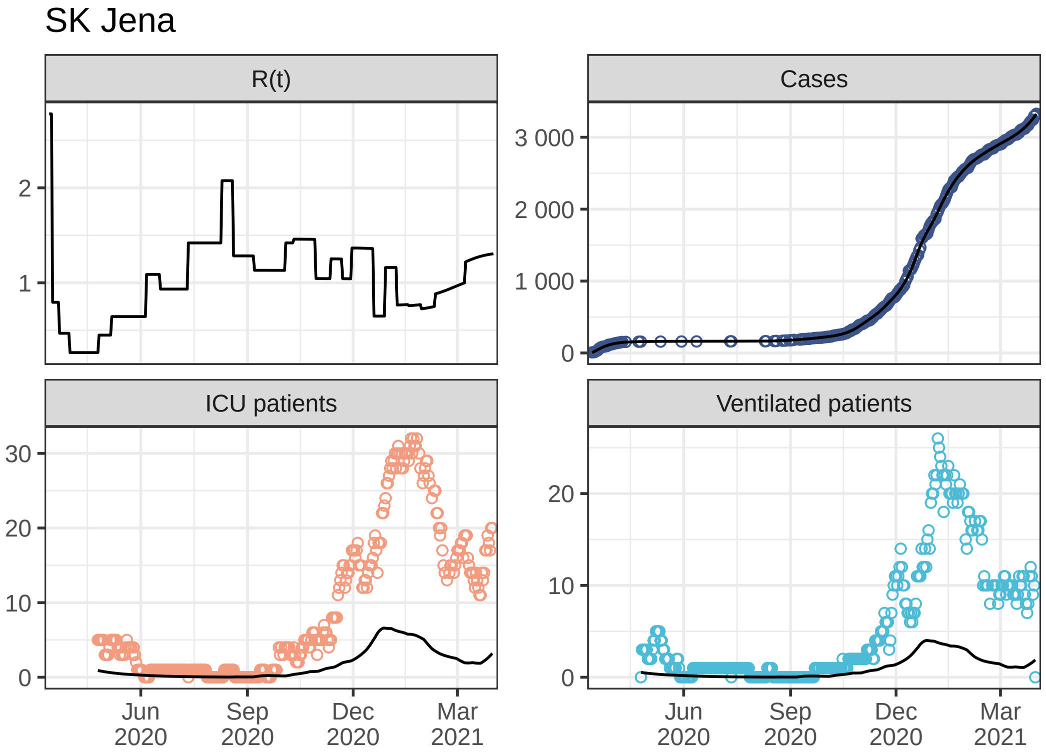
SK Jena
R(t)
Cases
ICU patients
Ventilated patients
1
2
0
1 000
2 000
3 000
0
10
20
30
0
10
20
Jun 2020
Sep 2020
Dec 2020
Mar 2021

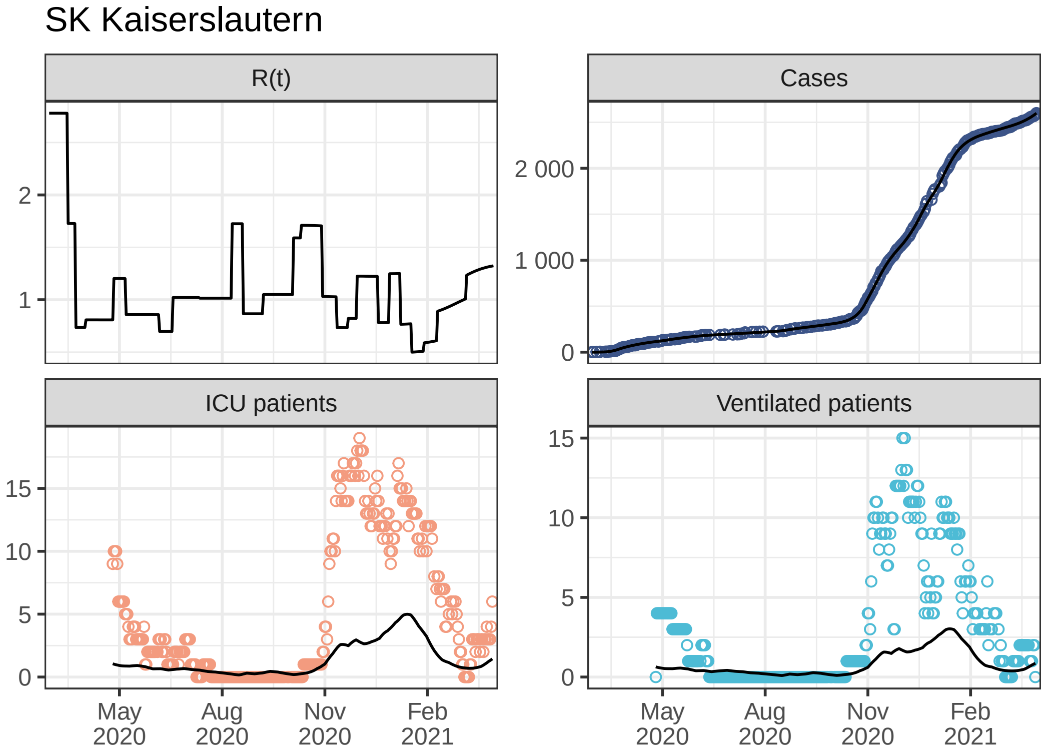
SK Kaiserslautern
R(t)
Cases
ICU patients
Ventilated patients
1
2
0
1 000
2 000
0
5
10
15
0
5
10
15
May 2020
Aug 2020
Nov 2020
Feb 2021

SK Karlsruhe

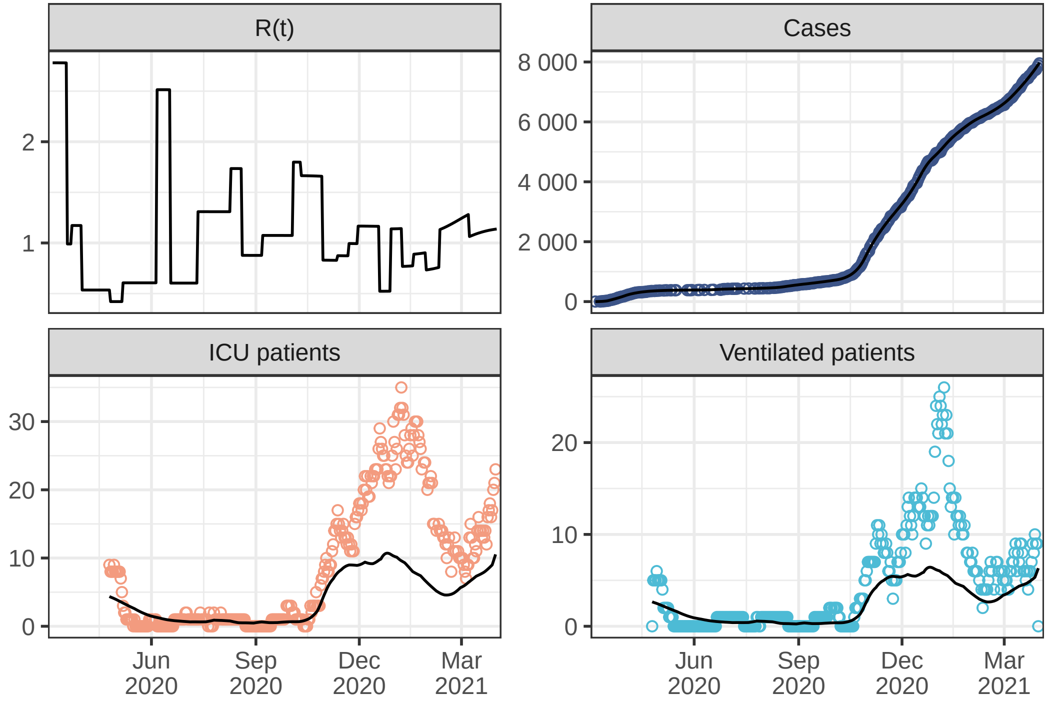

SK Kassel

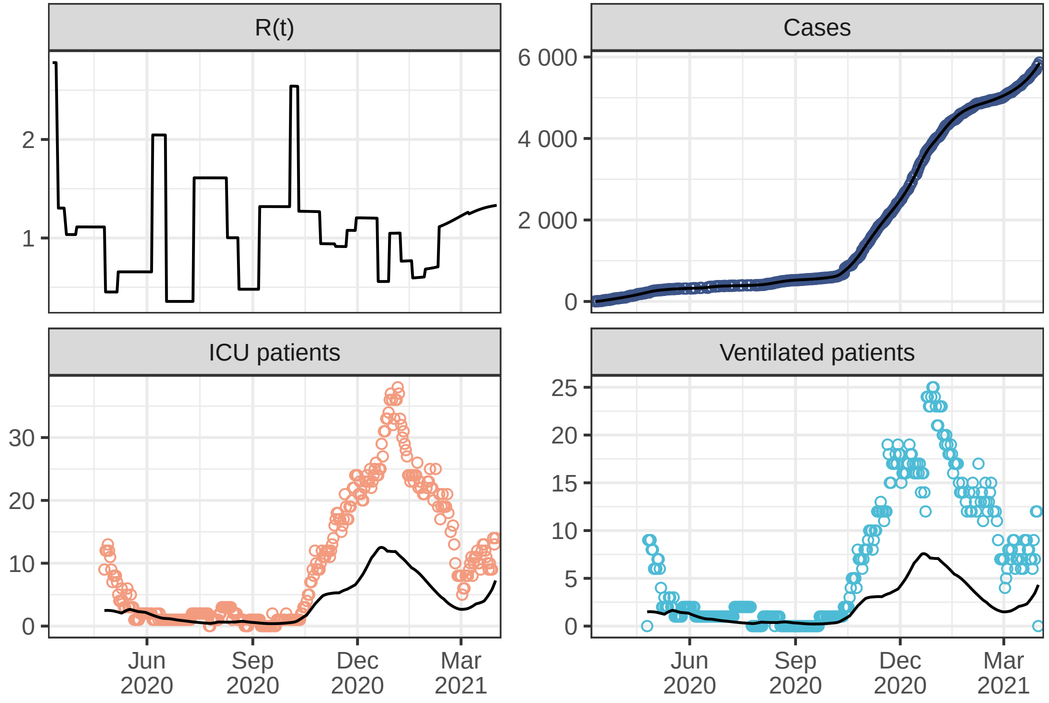

SK Kaufbeuren

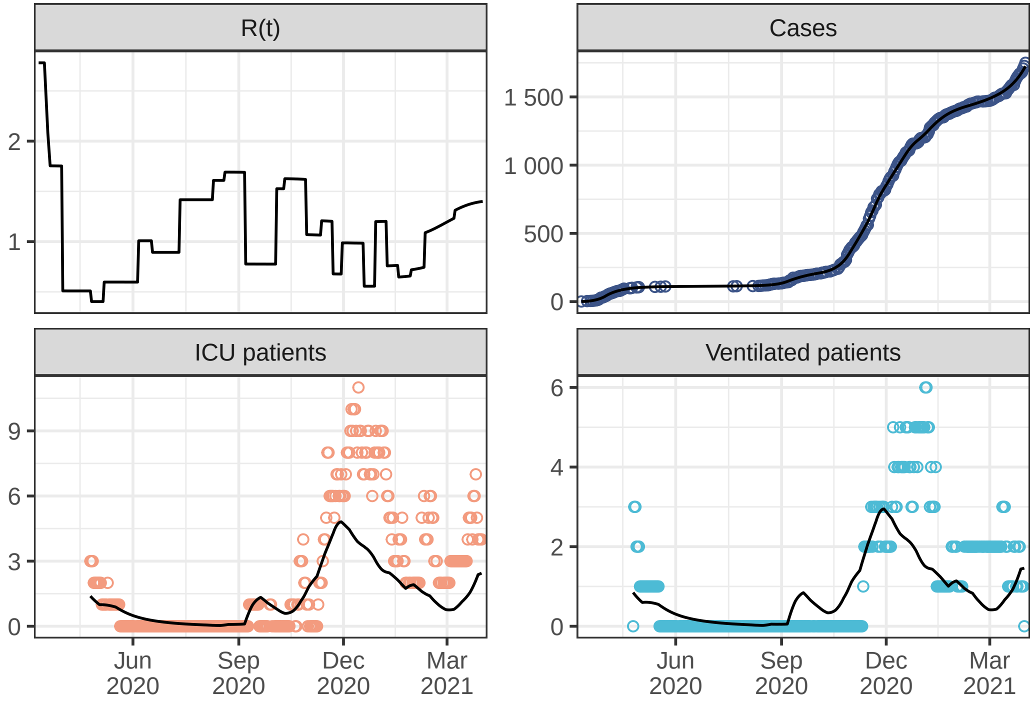

SK Kempten

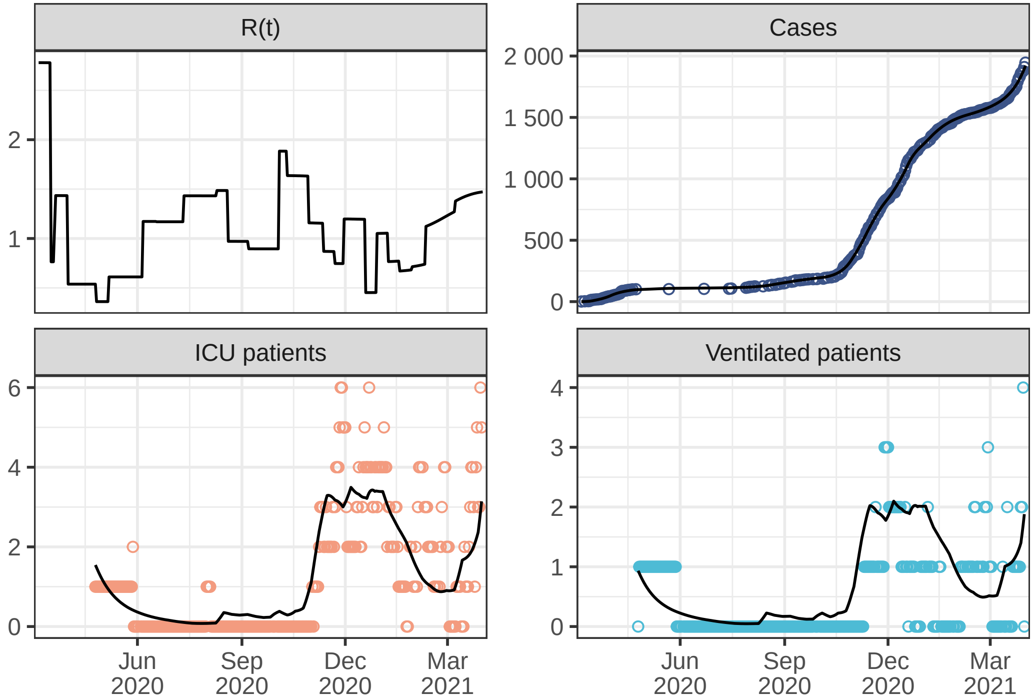

SK Kiel

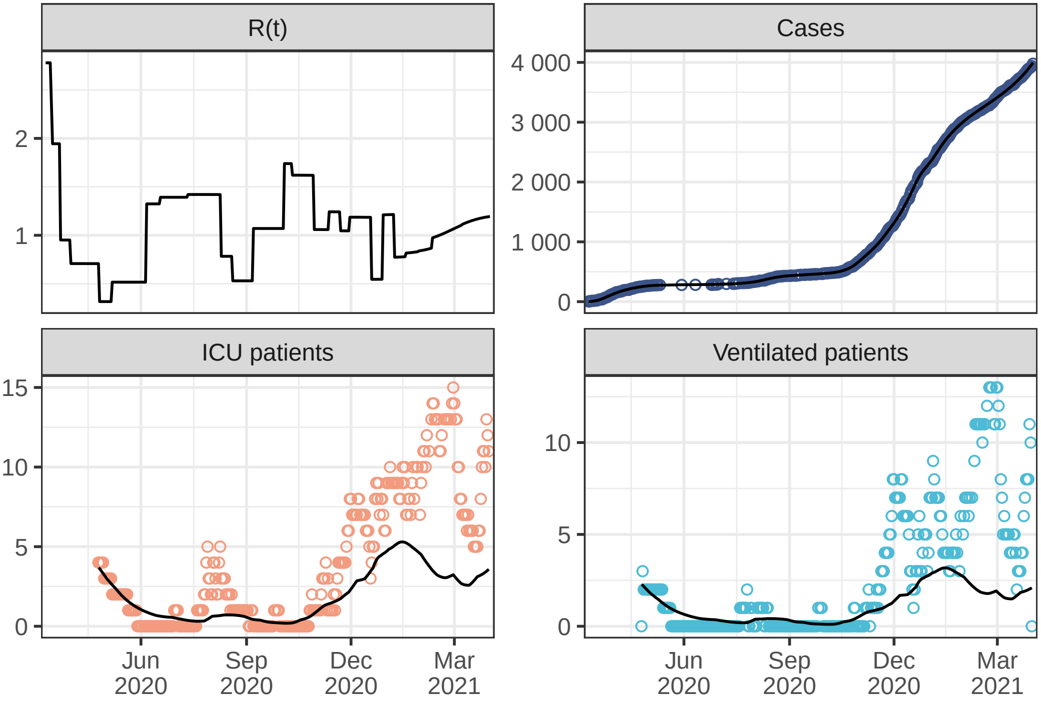

SK Koblenz

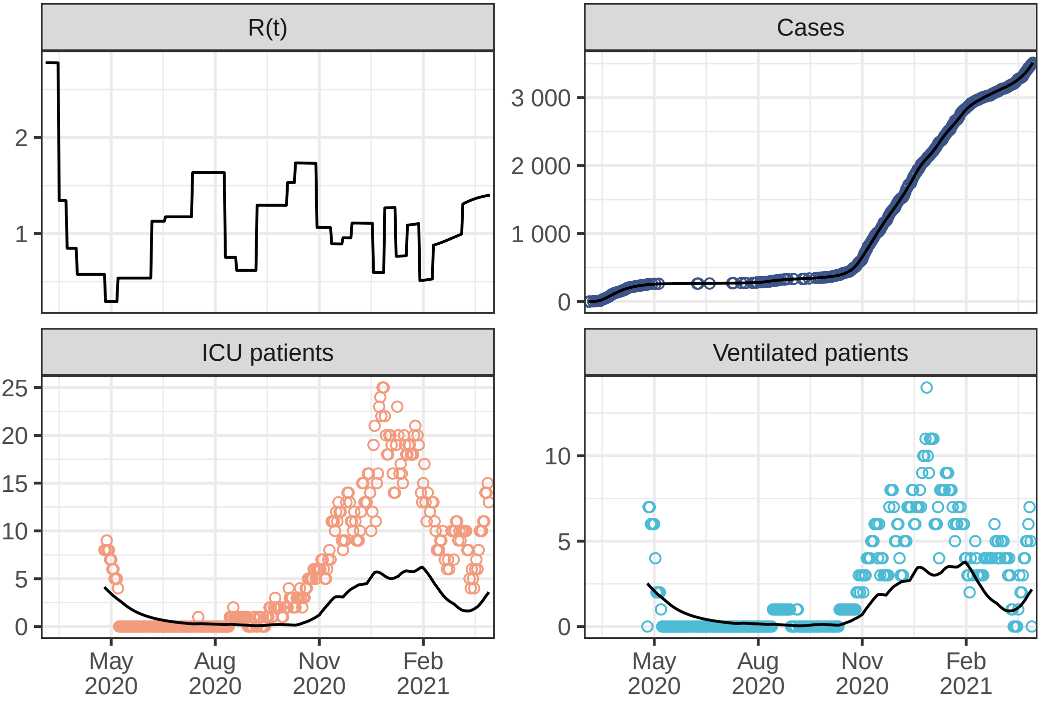

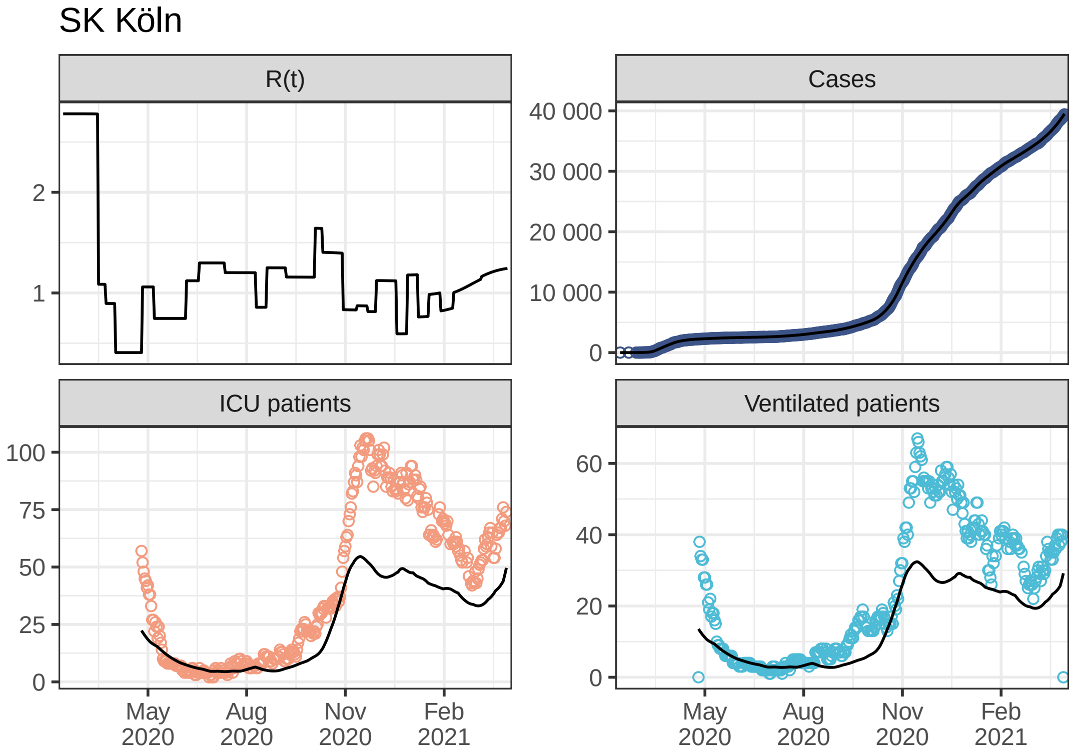
SK Köln
R(t)
Cases
ICU patients
Ventilated patients
May 2020
Aug 2020
Nov 2020
Feb 2021

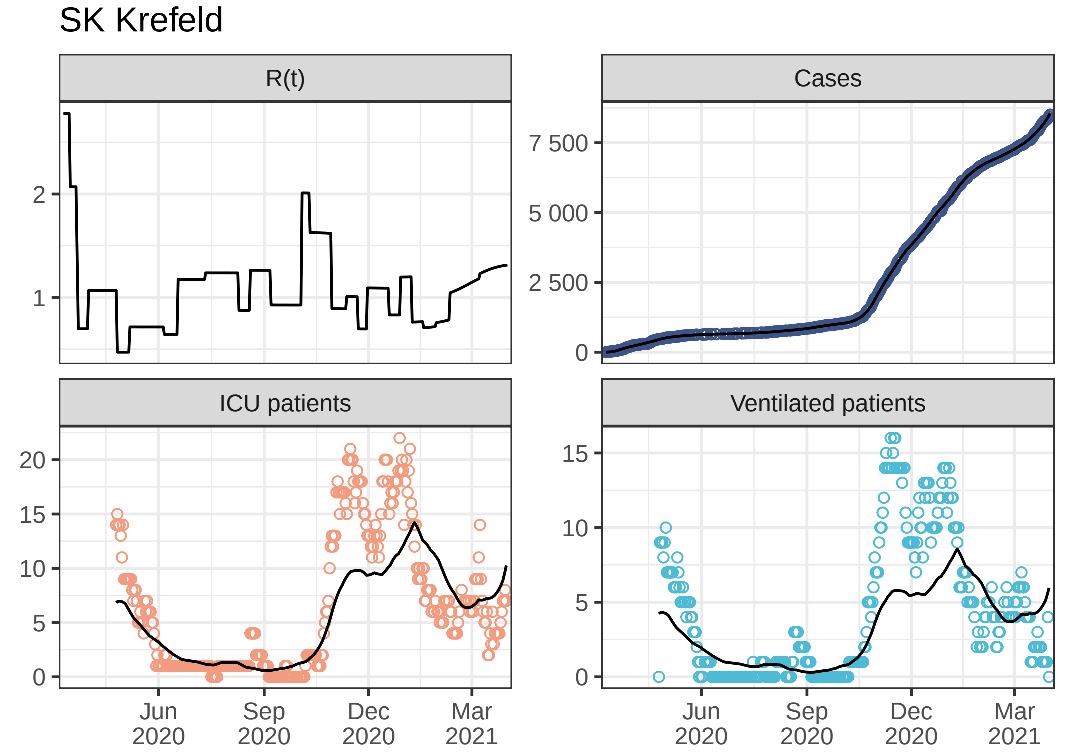
SK Krefeld
R(t)
Cases
ICU patients
Ventilated patients
Jun 2020
Sep 2020
Dec 2020
Mar 2021

SK Landau i.d.Pfalz

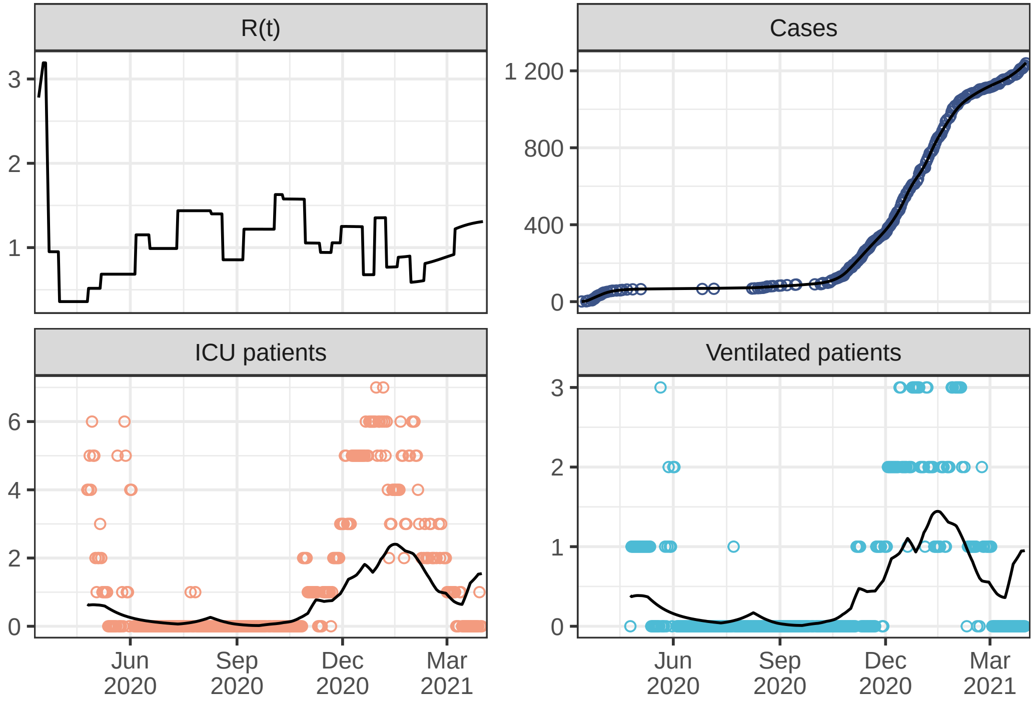

SK Landshut

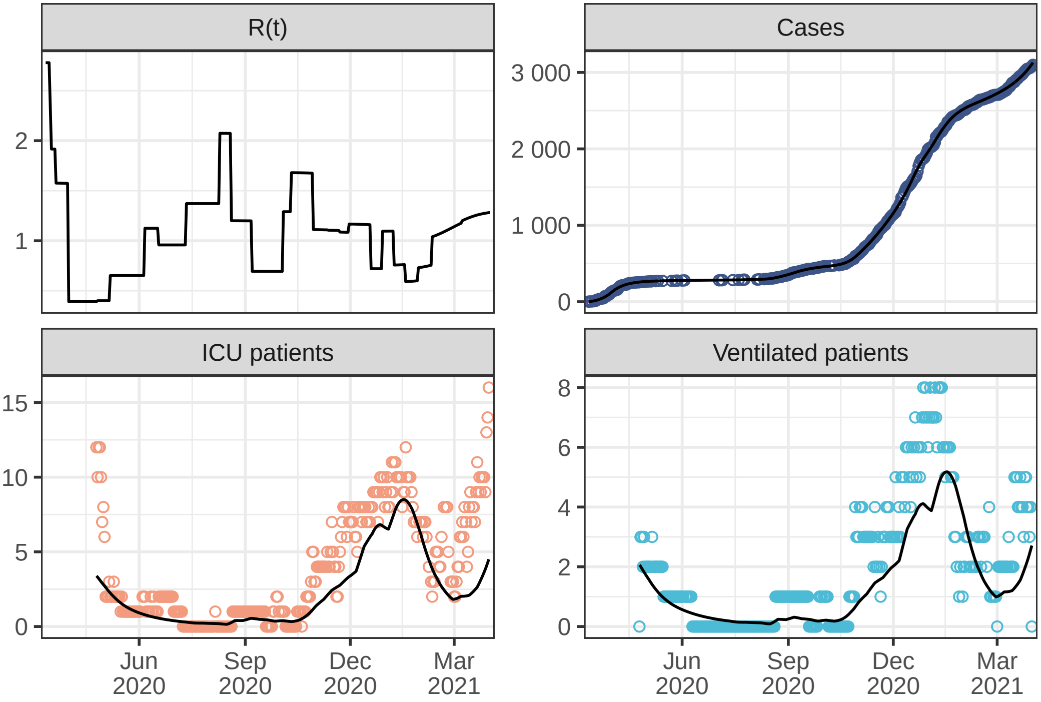

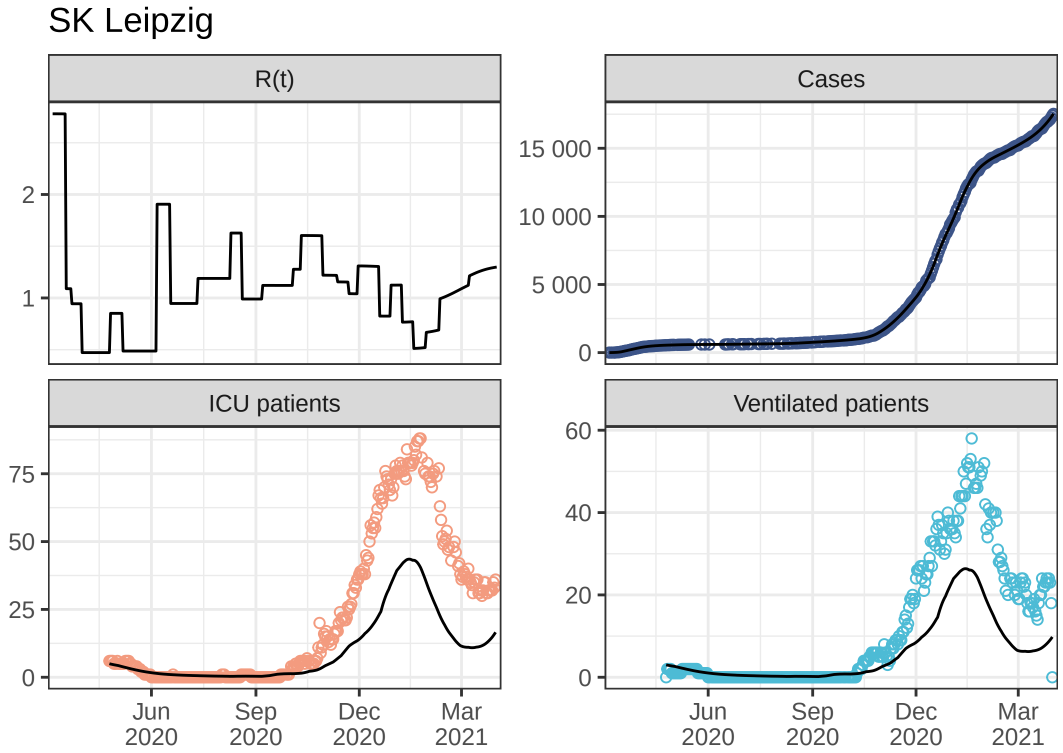
SK Leipzig
R(t)
Cases
ICU patients
Ventilated patients
2
1
15 000
10 000
5 000
0
75
50
25
0
60
40
20
0
Jun 2020
Sep 2020
Dec 2020
Mar 2021

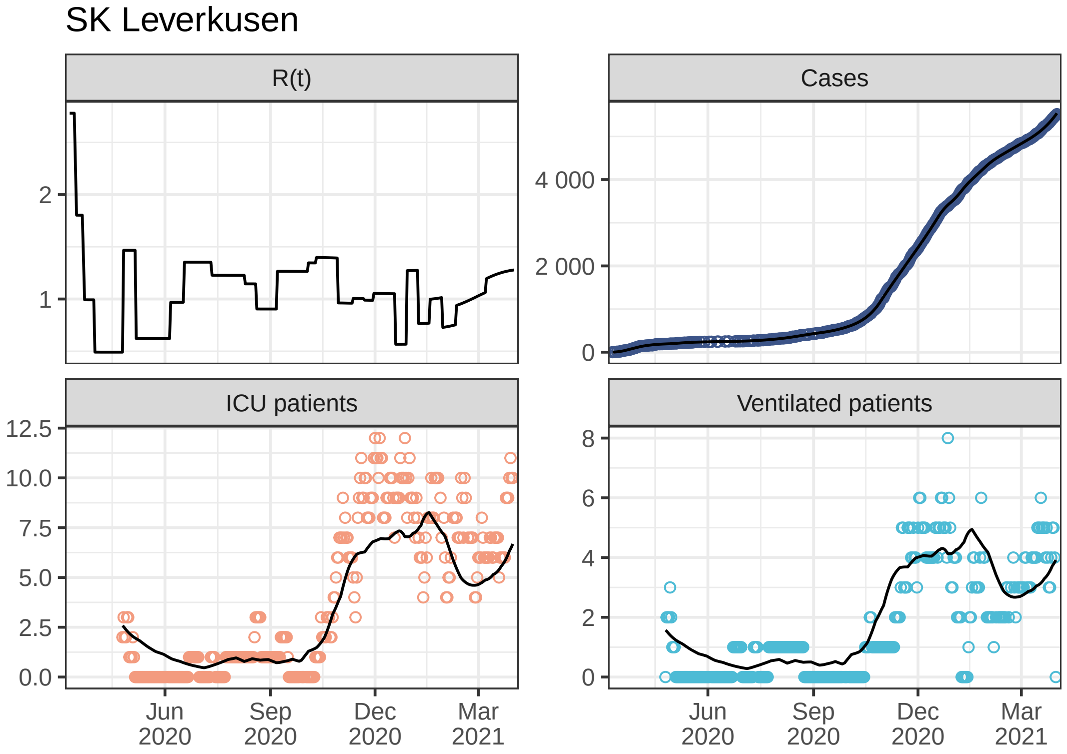
SK Leverkusen
R(t)
Cases
ICU patients
Ventilated patients
2
1
4 000
2 000
0
12.5
10.0
7.5
5.0
2.5
0.0
8
6
4
2
0
Jun 2020
Sep 2020
Dec 2020
Mar 2021

SK Lübeck

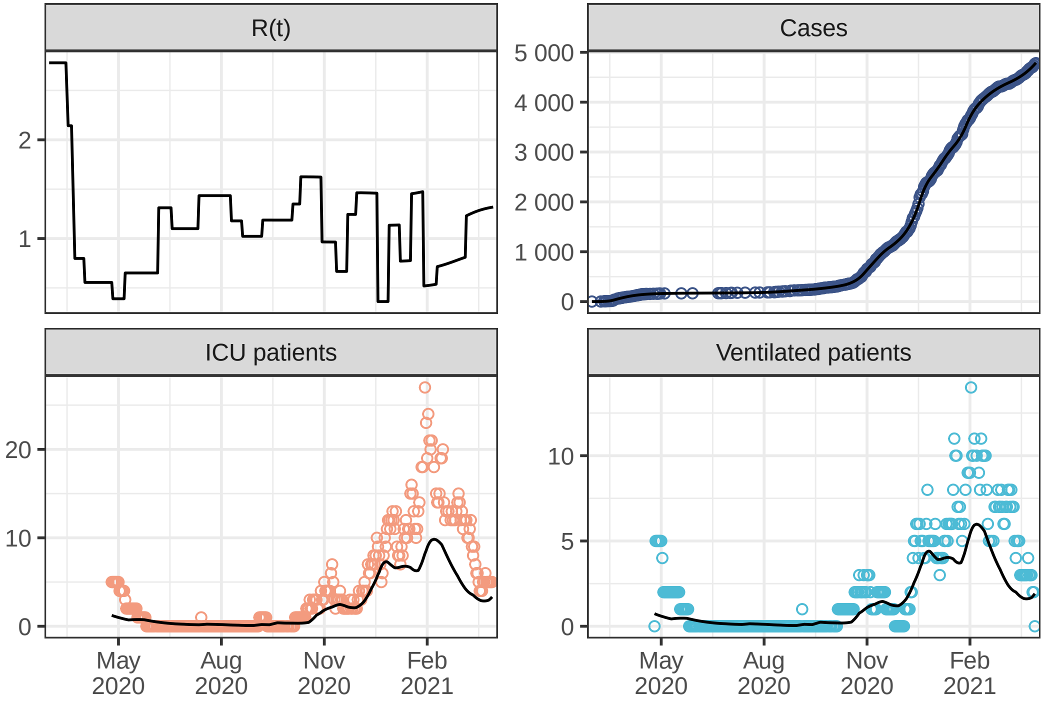

SK Ludwigshafen

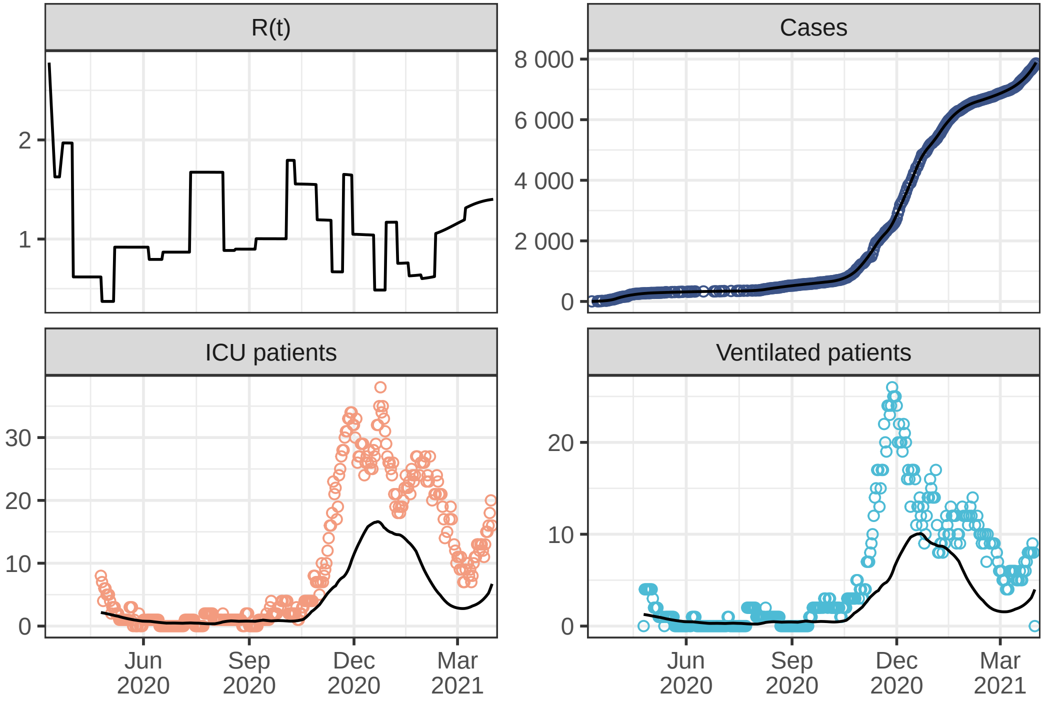

SK Magdeburg

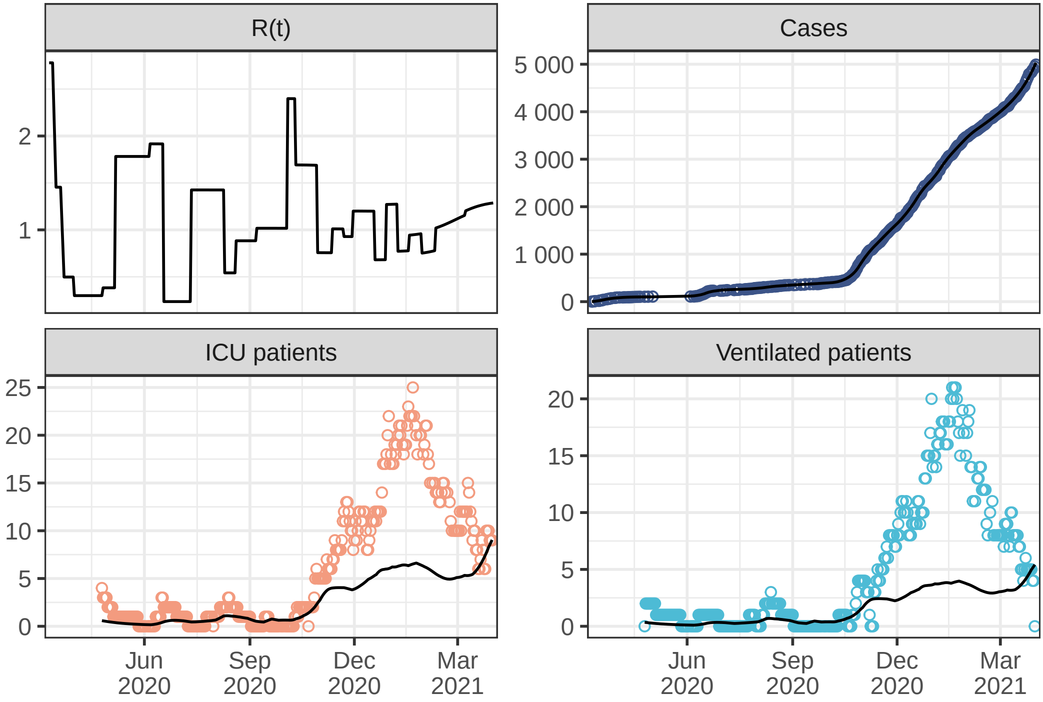

SK Mainz

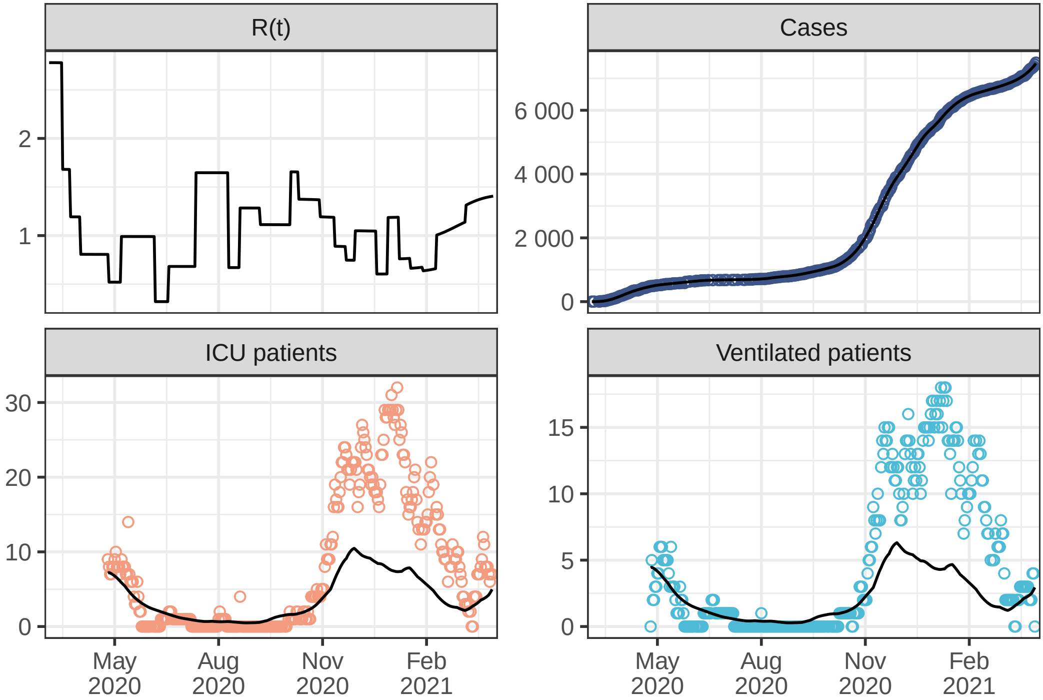

SK Mannheim

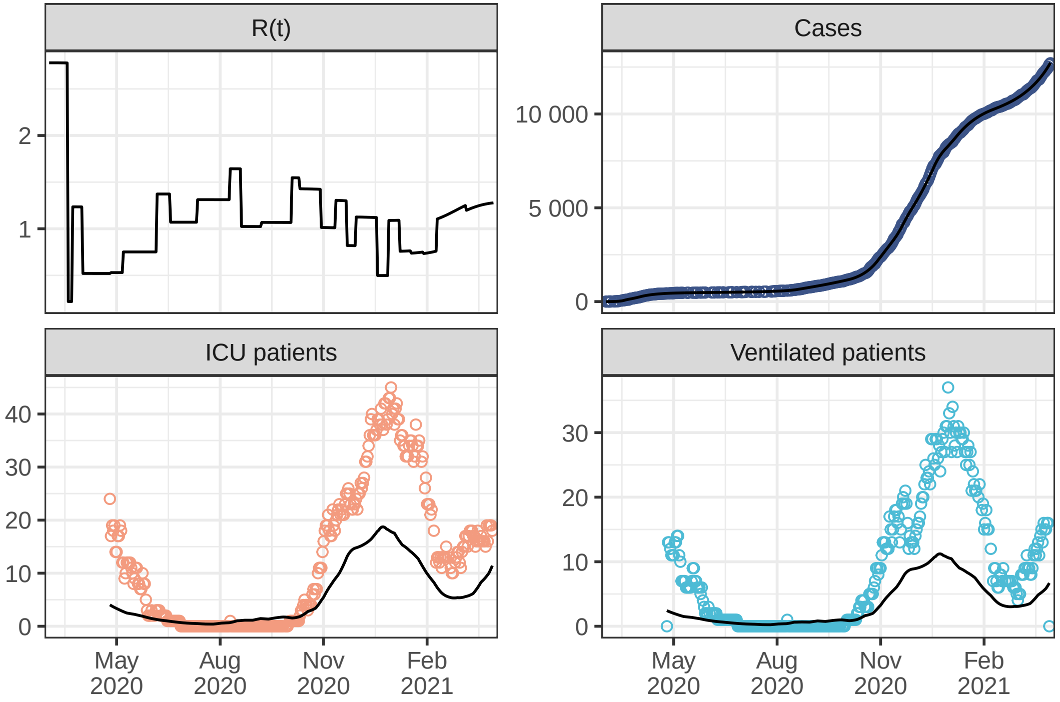

SK Memmingen

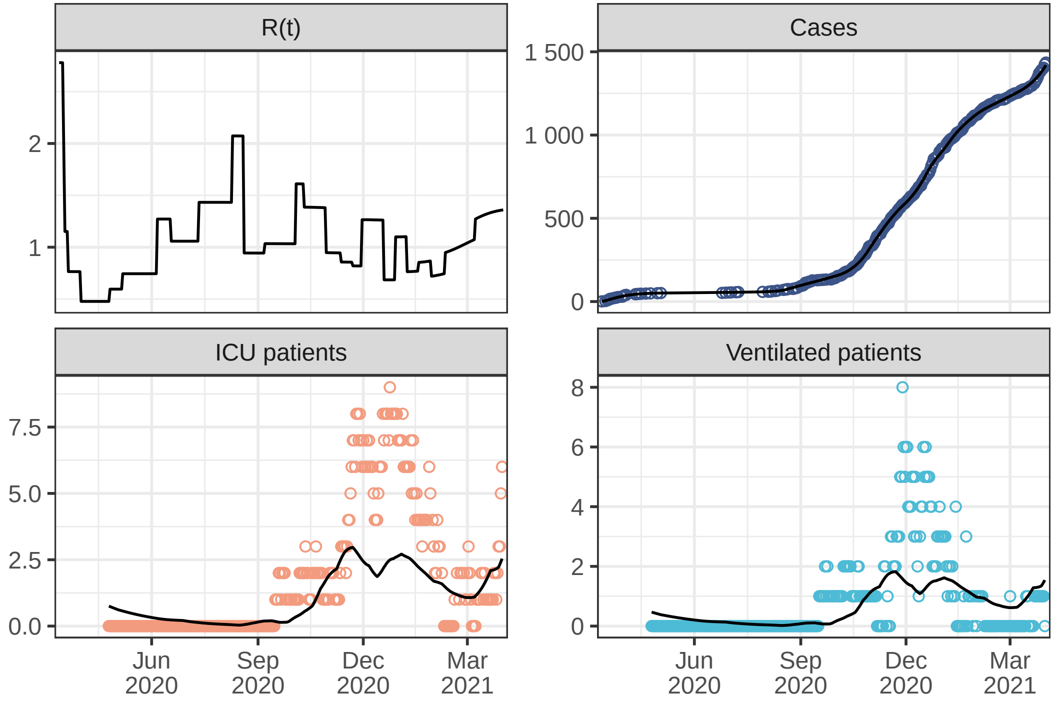

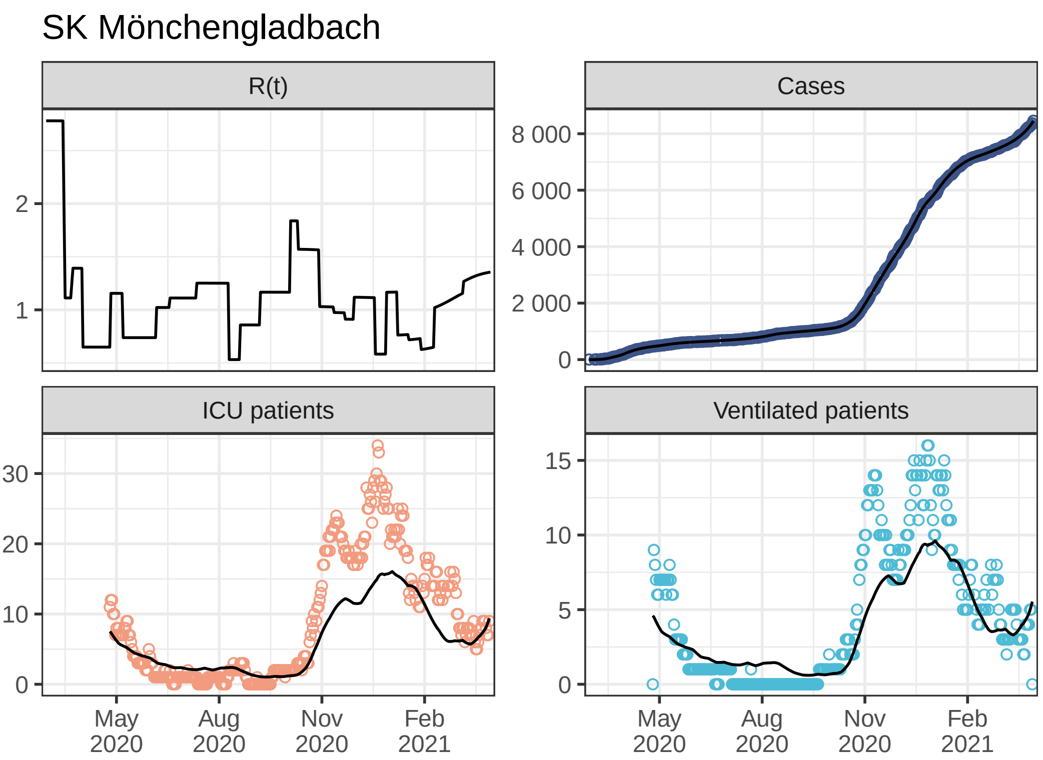
SK Mönchengladbach
R(t)
Cases
ICU patients
Ventilated patients
2
1
8 000
6 000
4 000
2 000
0
30
20
10
0
15
10
5
0
May 2020
Aug 2020
Nov 2020
Feb 2021

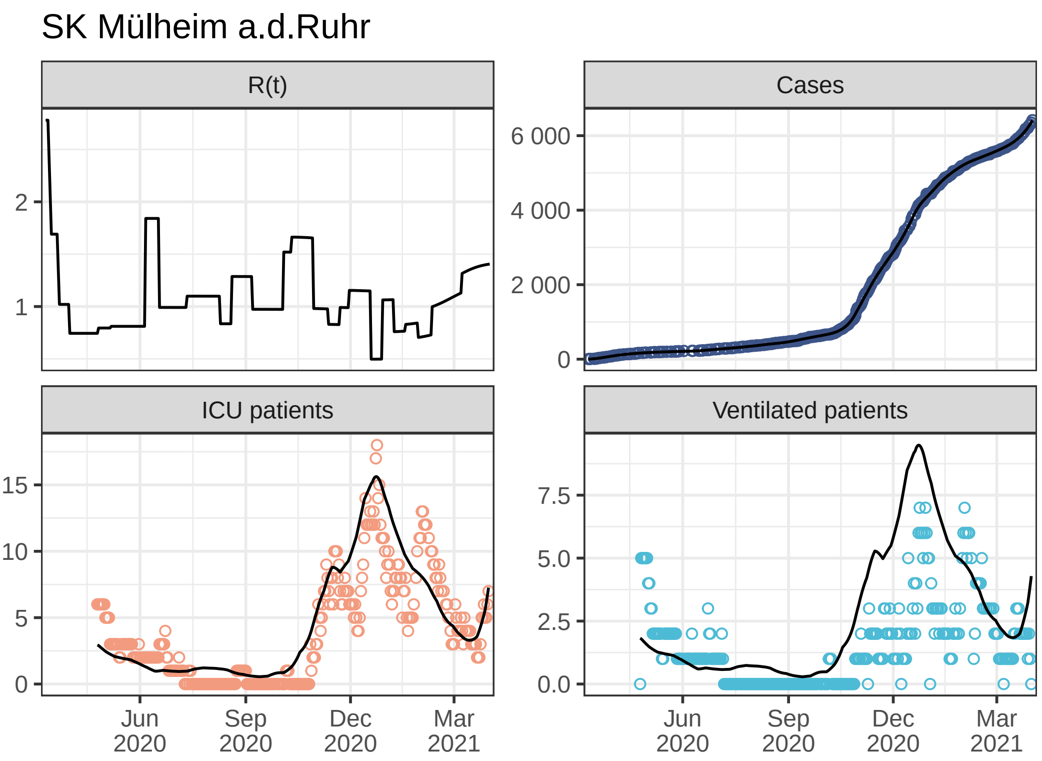
SK Mülheim a.d.Ruhr
R(t)
Cases
ICU patients
Ventilated patients
2
1
6 000
4 000
2 000
0
15
10
5
0
7.5
5.0
2.5
0.0
Jun 2020
Sep 2020
Dec 2020
Mar 2021

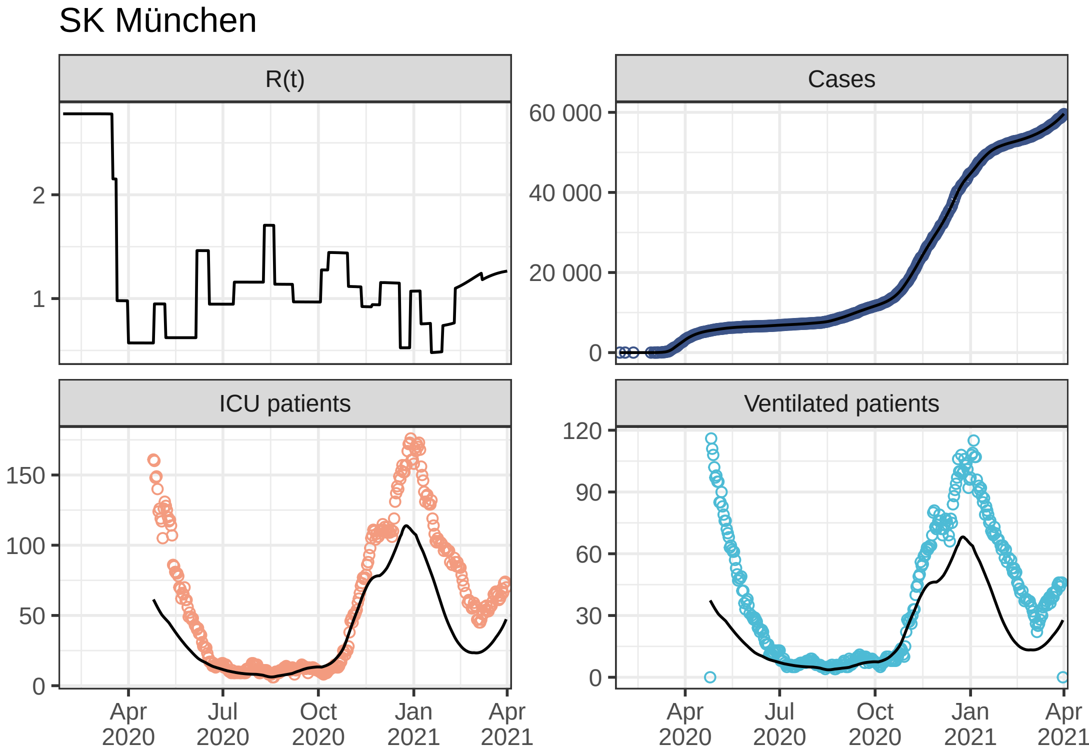
SK München
R(t)
Cases
ICU patients
Ventilated patients
2
1
60 000
40 000
20 000
0
150
100
50
0
120
90
60
30
0
Apr 2020
Jul 2020
Oct 2020
Jan 2021
Apr 2021

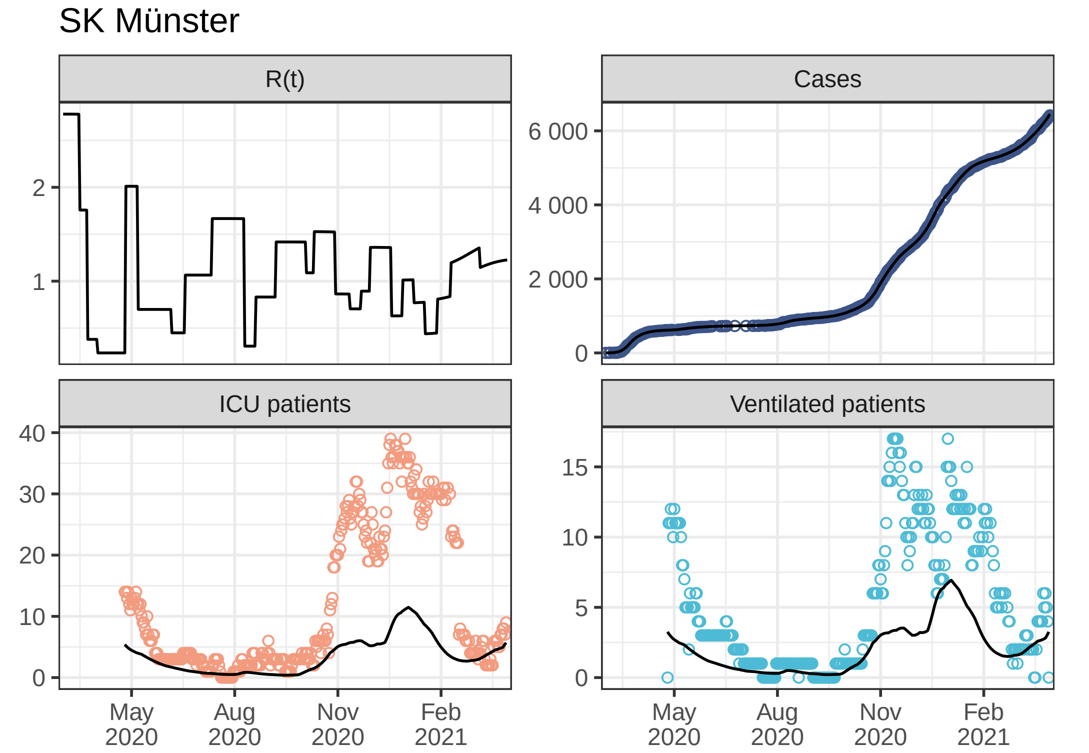
SK Münster
R(t)
Cases
ICU patients
Ventilated patients
2
1
6 000
4 000
2 000
0
40
30
20
10
0
15
10
5
0
May 2020
Aug 2020
Nov 2020
Feb 2021

SK Neumünster

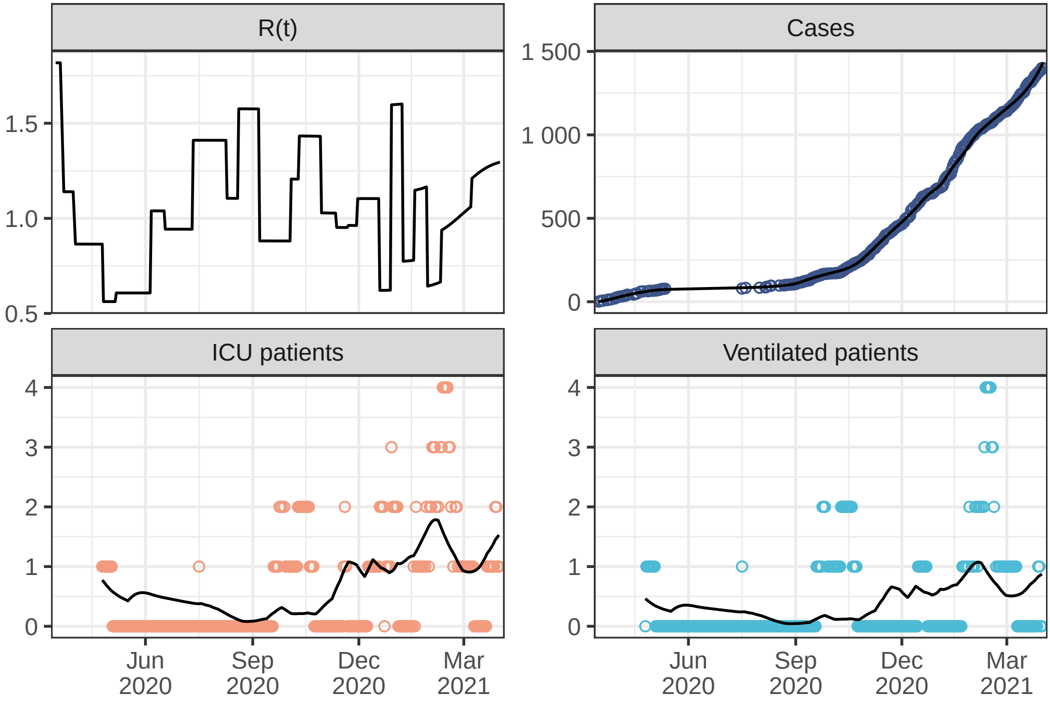

SK Neustadt a.d.Weinstraße

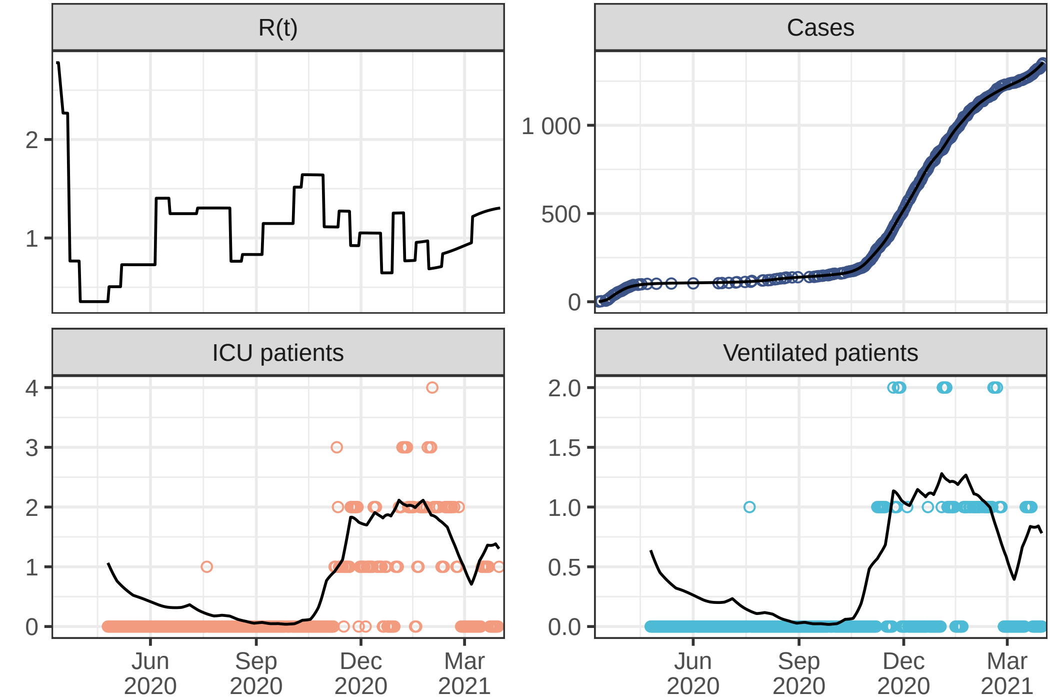

SK Nürnberg

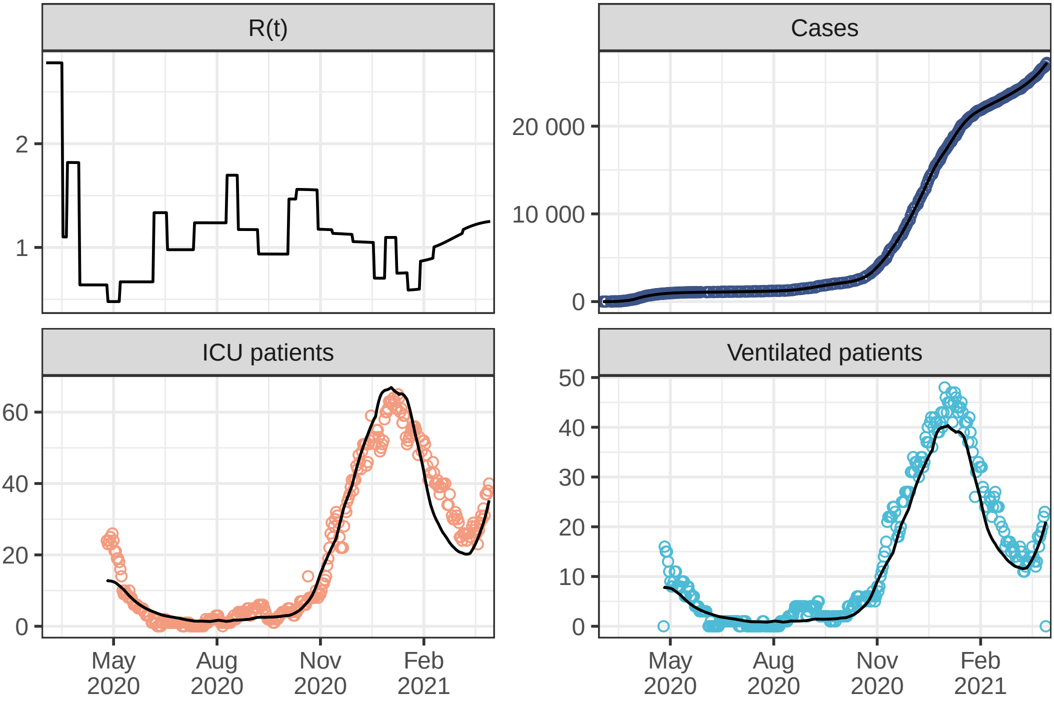

SK Oberhausen

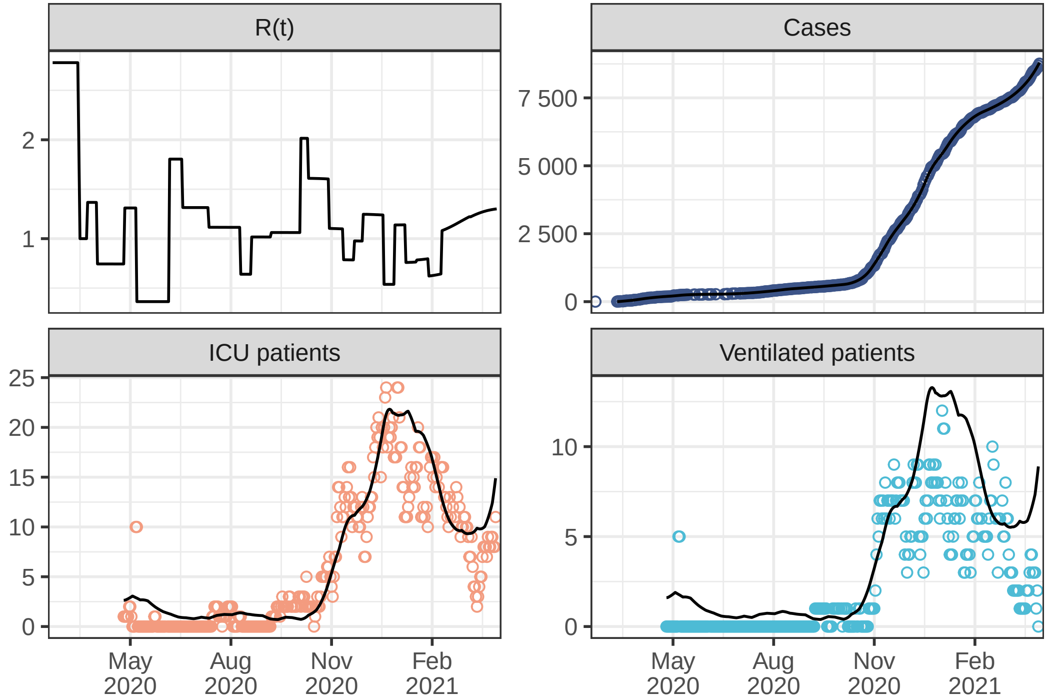

SK Offenbach

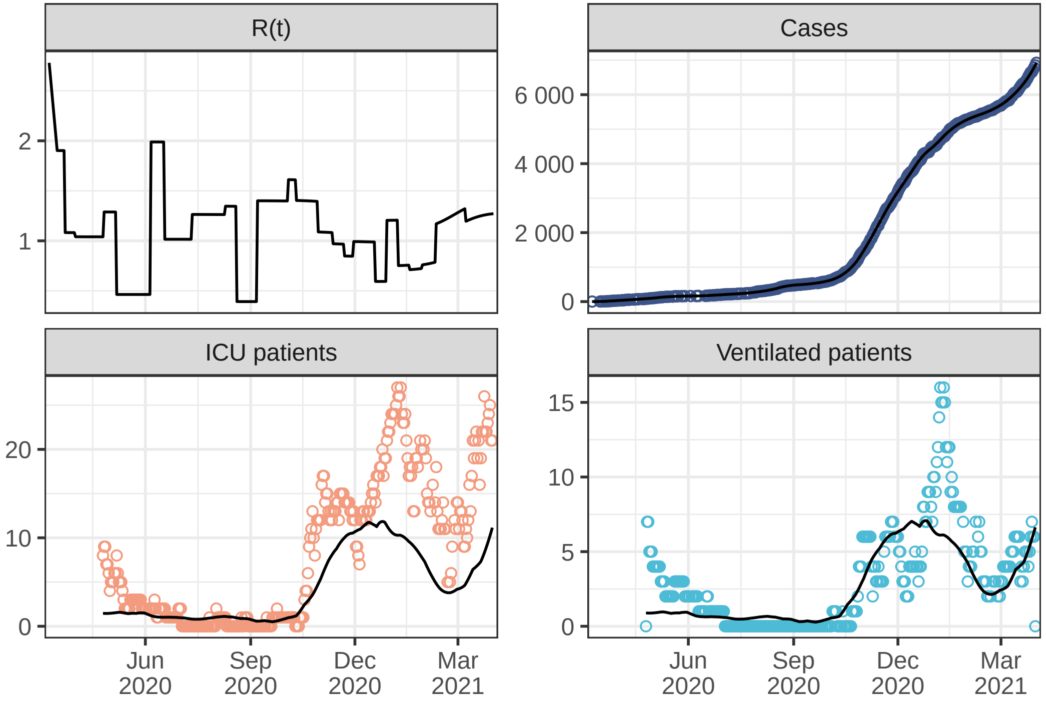

SK Oldenburg

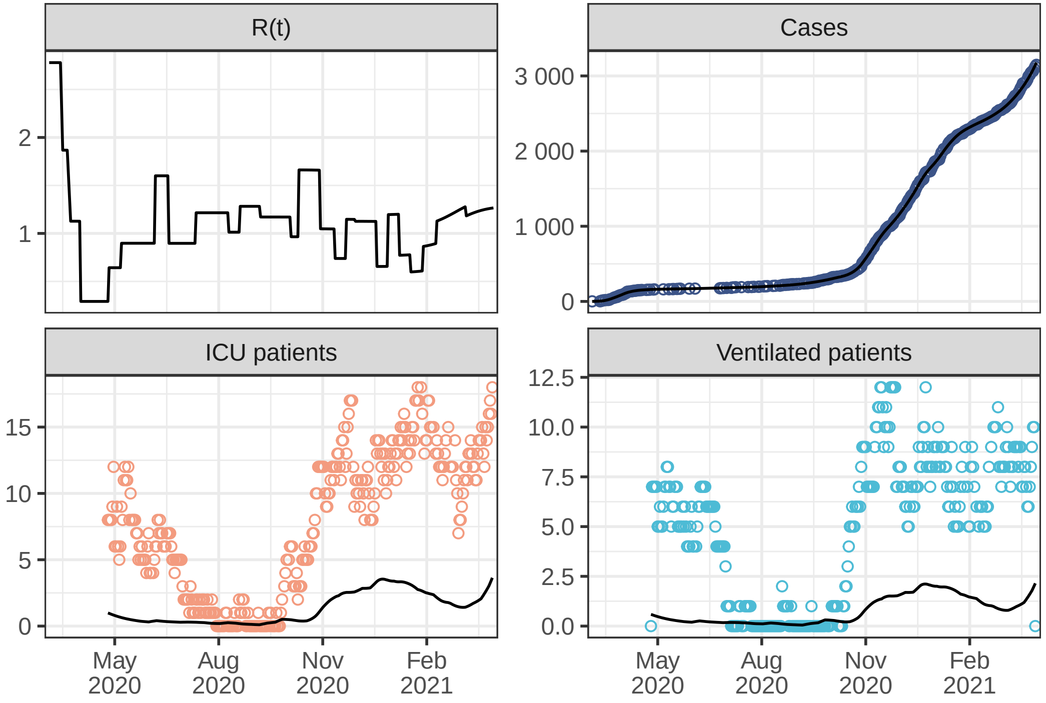

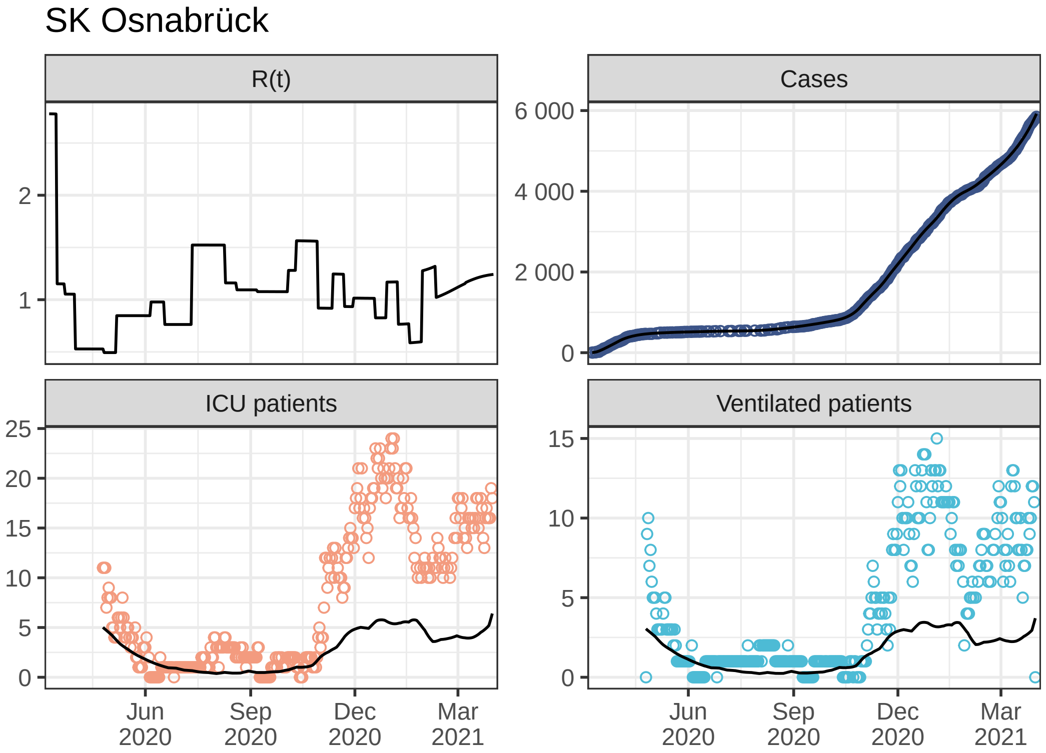
SK Osnabrück
R(t)
Cases
ICU patients
Ventilated patients
1
2
0
2 000
4 000
6 000
0
5
10
15
20
25
0
5
10
15
Jun 2020
Sep 2020
Dec 2020
Mar 2021

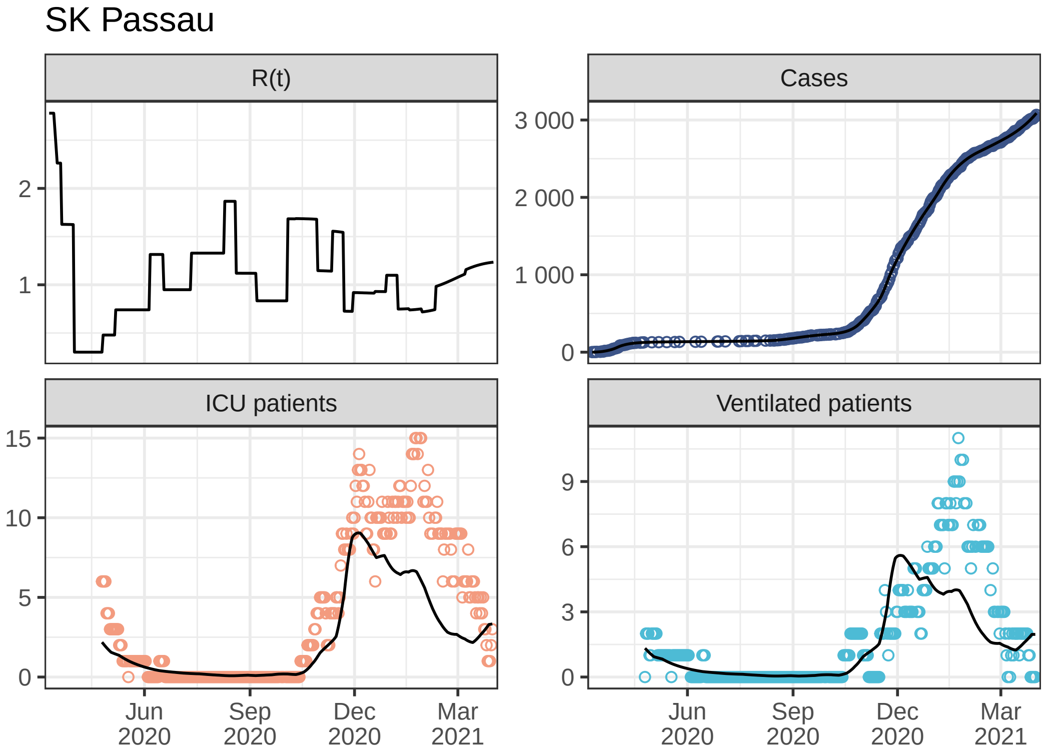
SK Passau
R(t)
Cases
ICU patients
Ventilated patients
1
2
0
1 000
2 000
3 000
0
5
10
15
0
3
6
9
Jun 2020
Sep 2020
Dec 2020
Mar 2021

SK Pforzheim

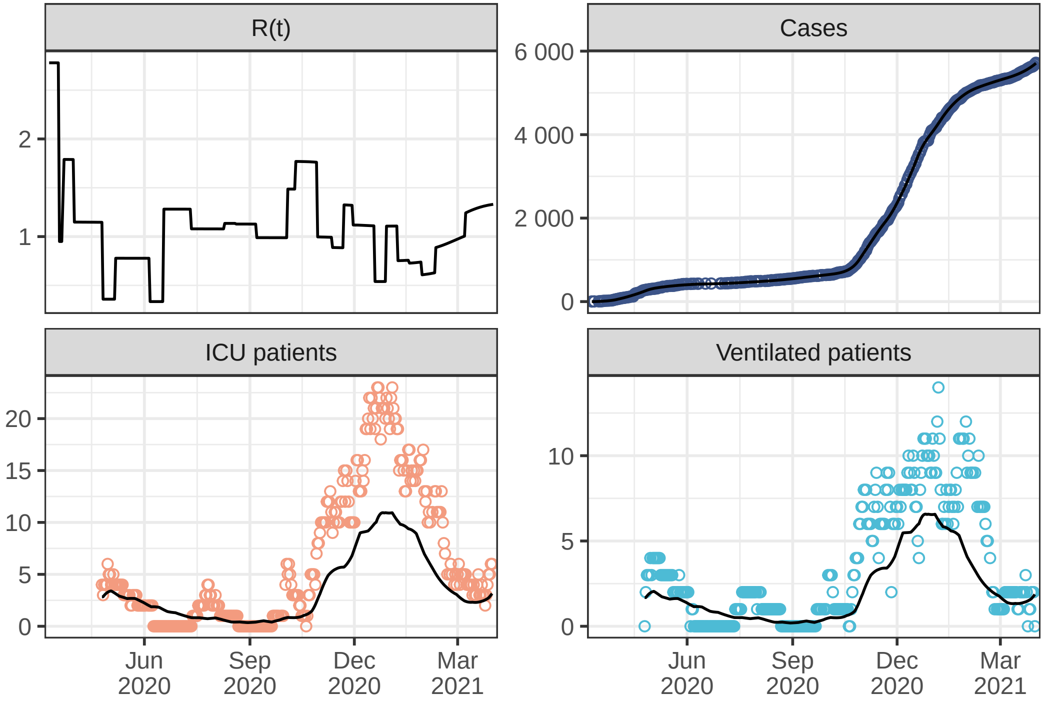

SK Pirmasens

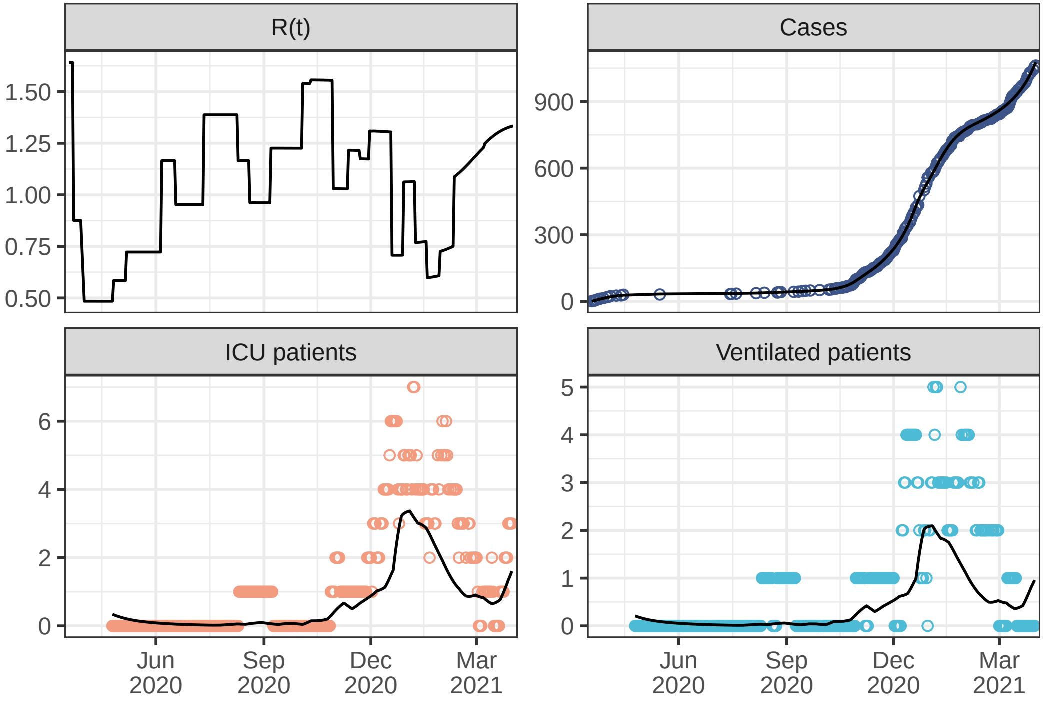

SK Potsdam

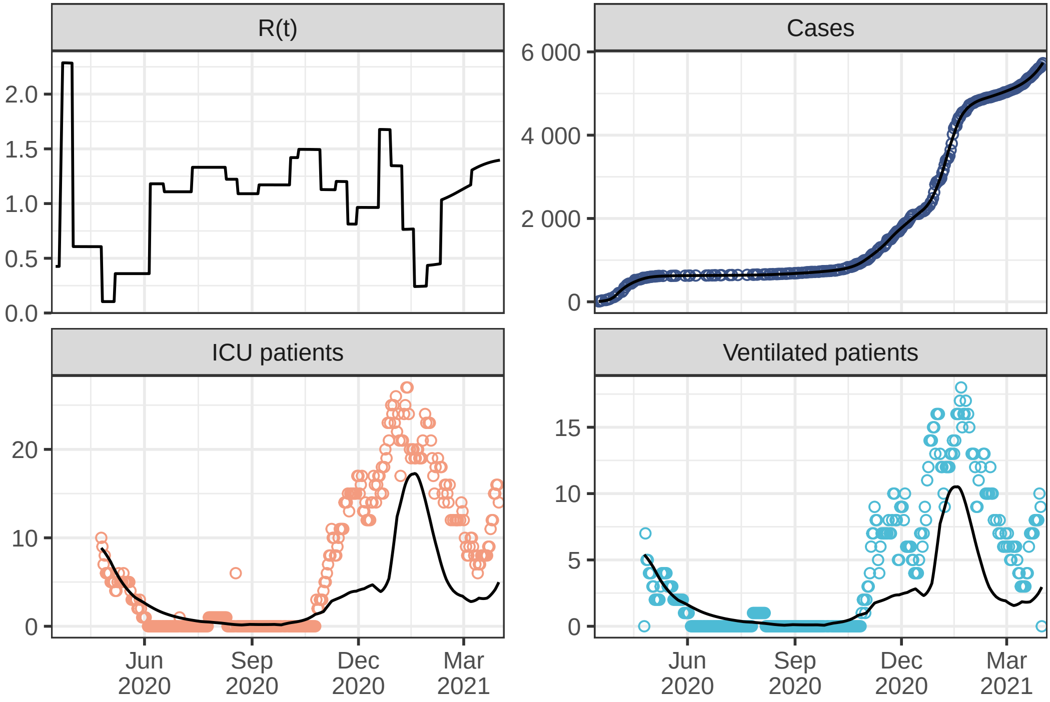

SK Regensburg

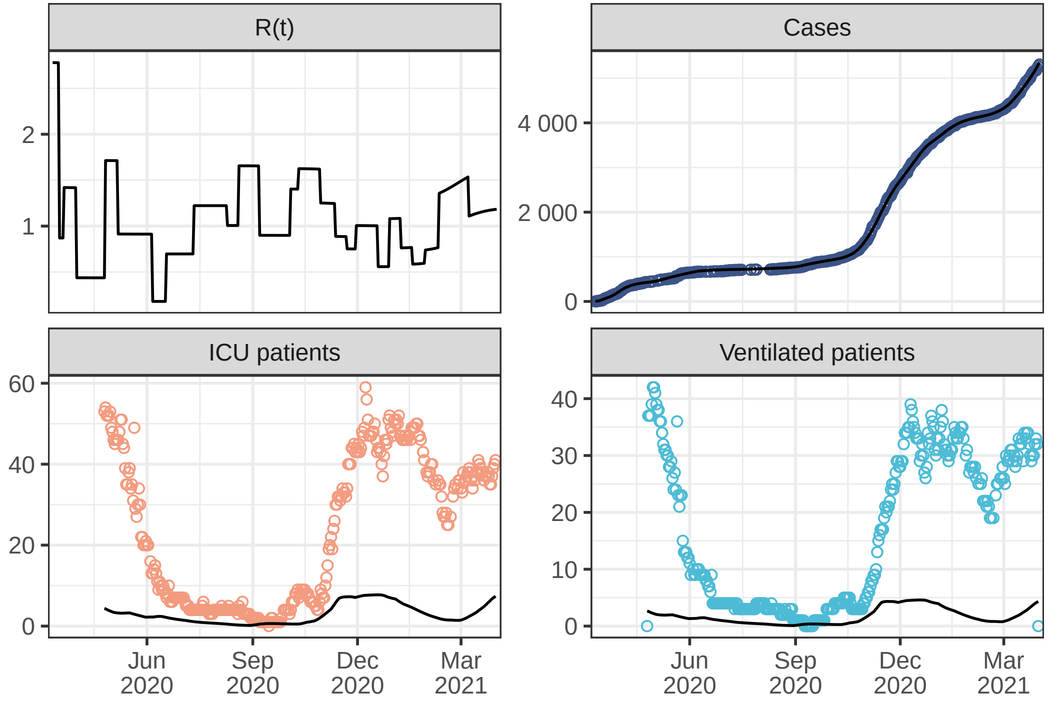

SK Remscheid

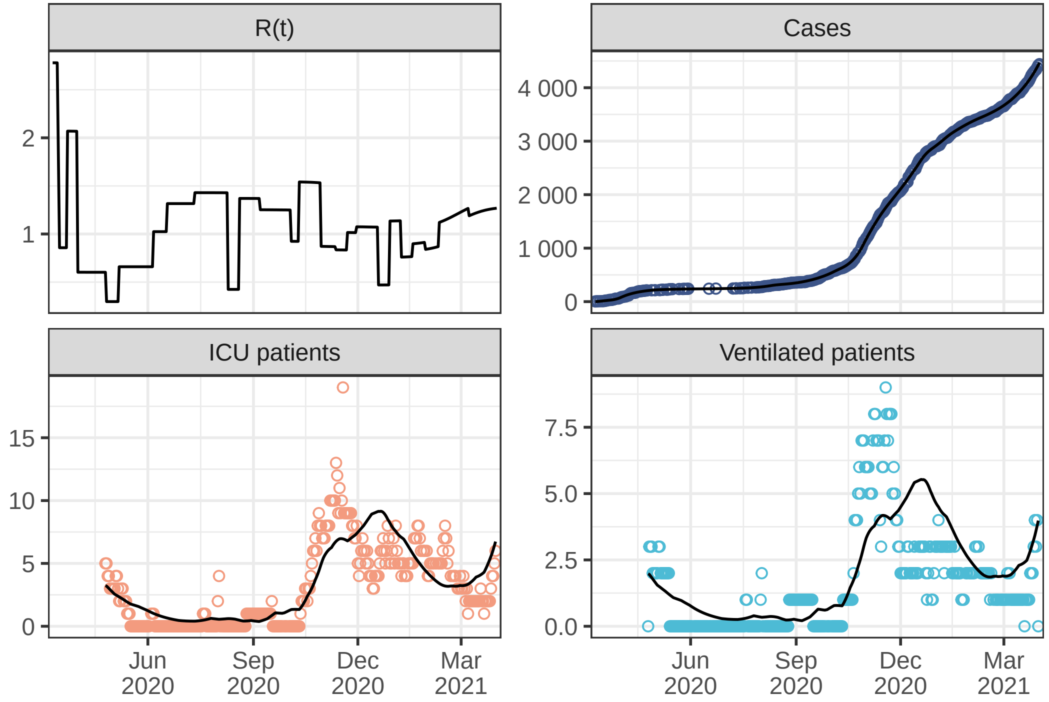

SK Rosenheim

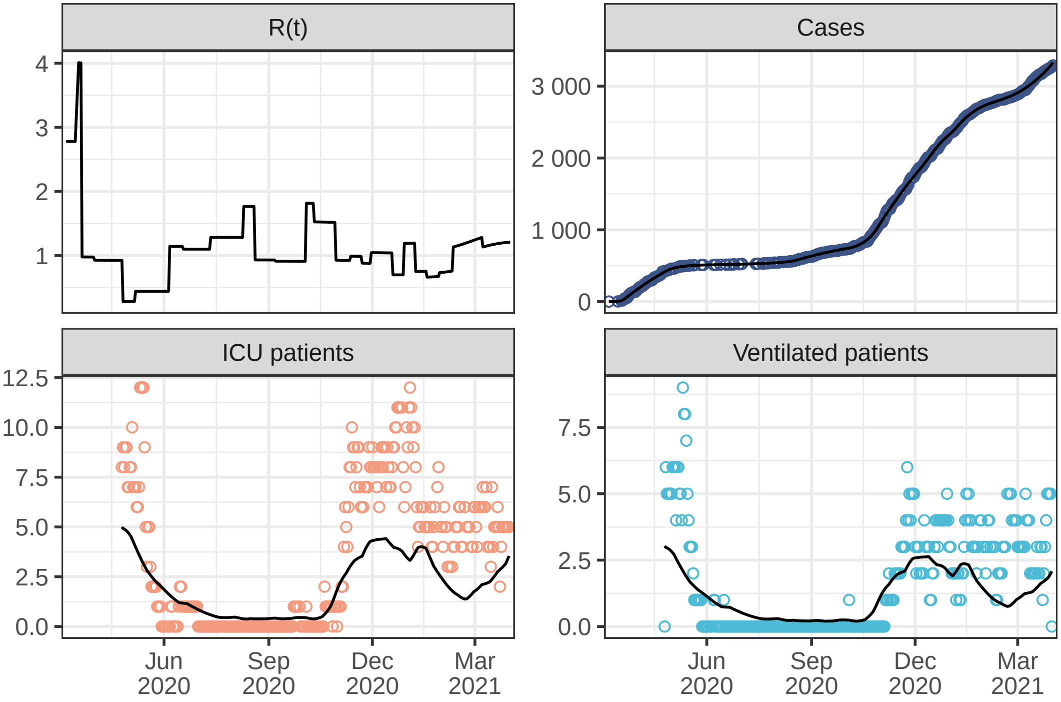

SK Rostock

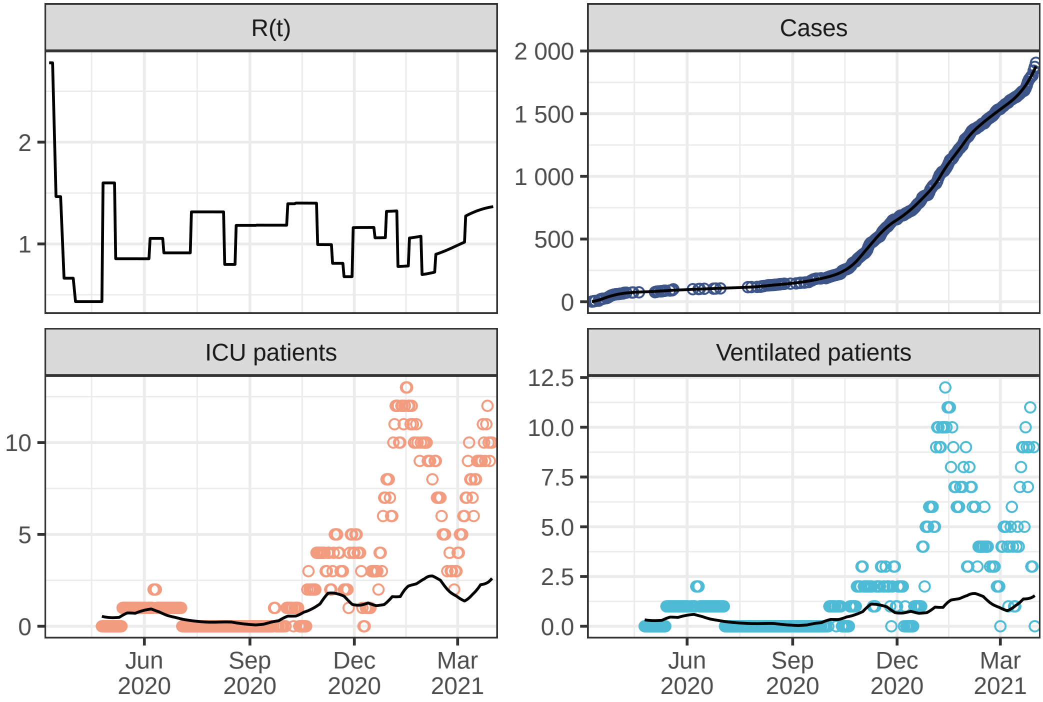

SK Salzgitter

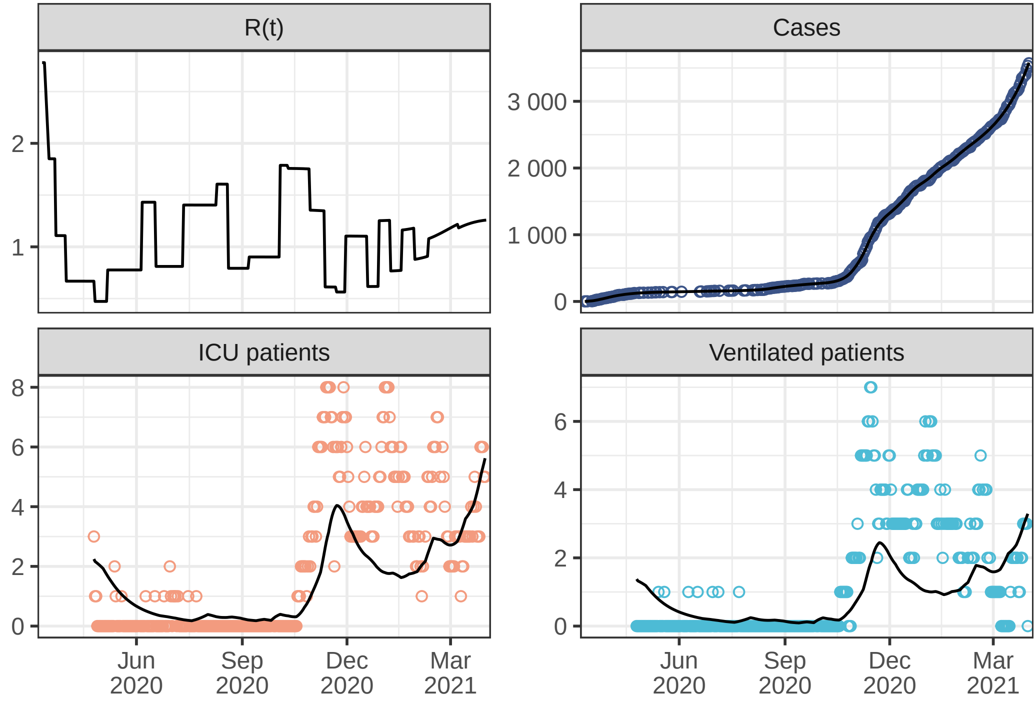

SK Schwabach

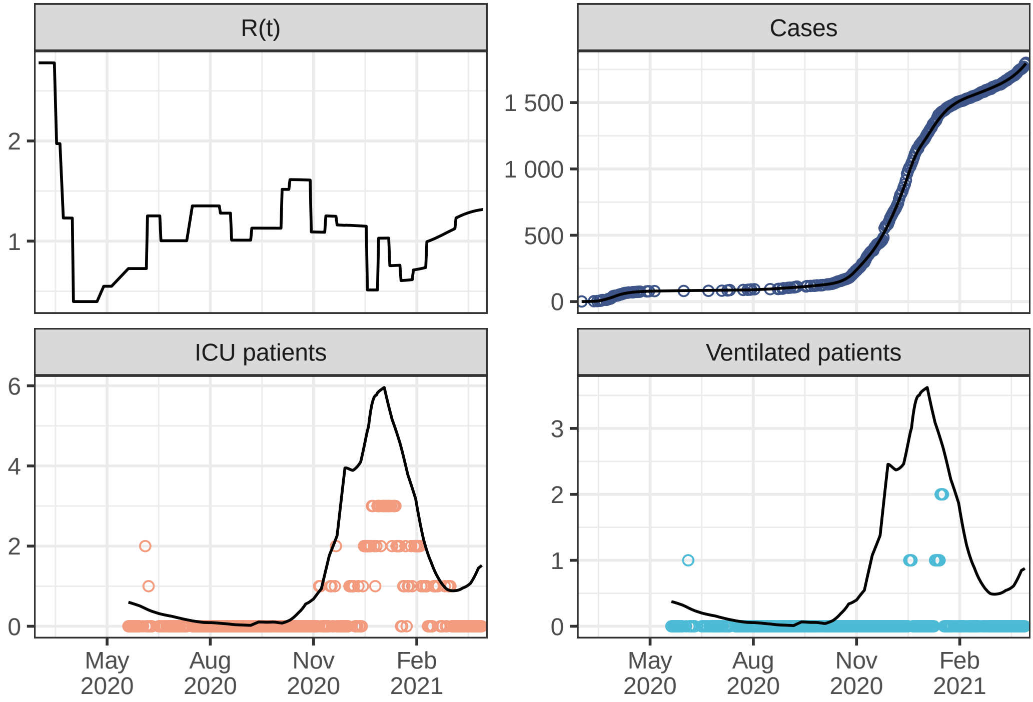

SK Schweinfurt

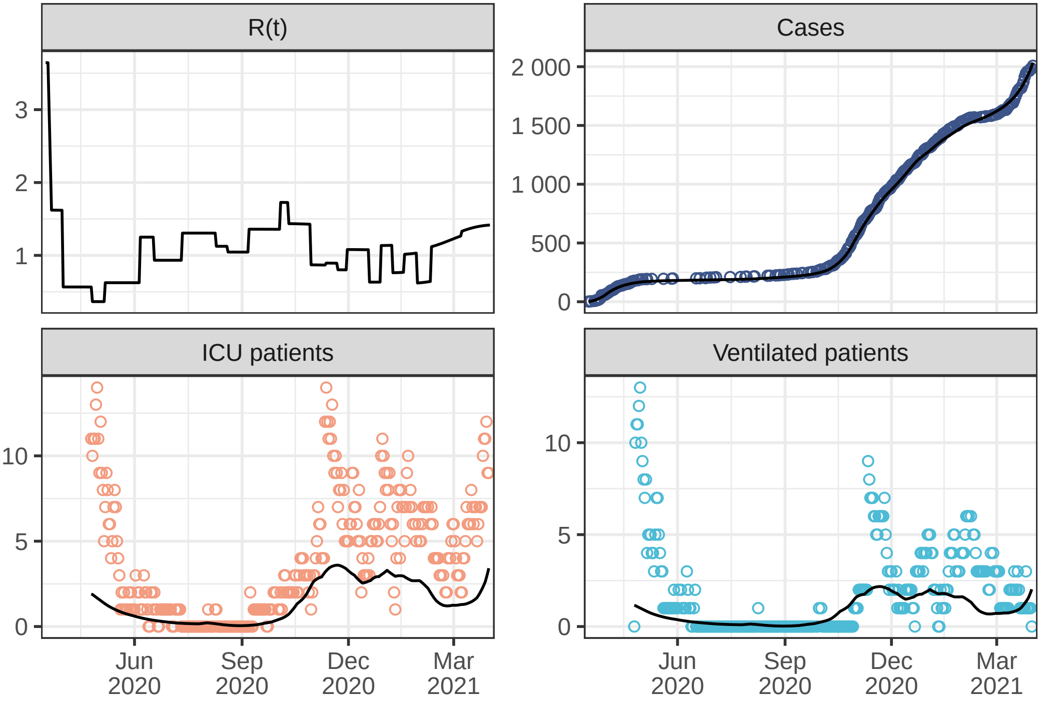

SK Schwerin

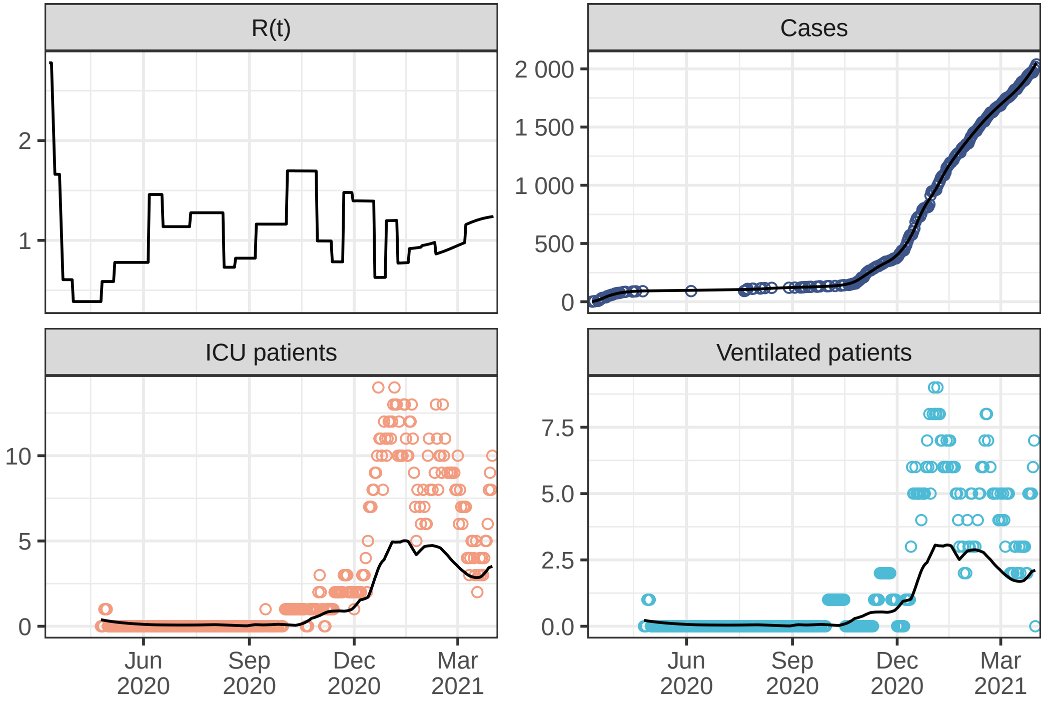

SK Solingen

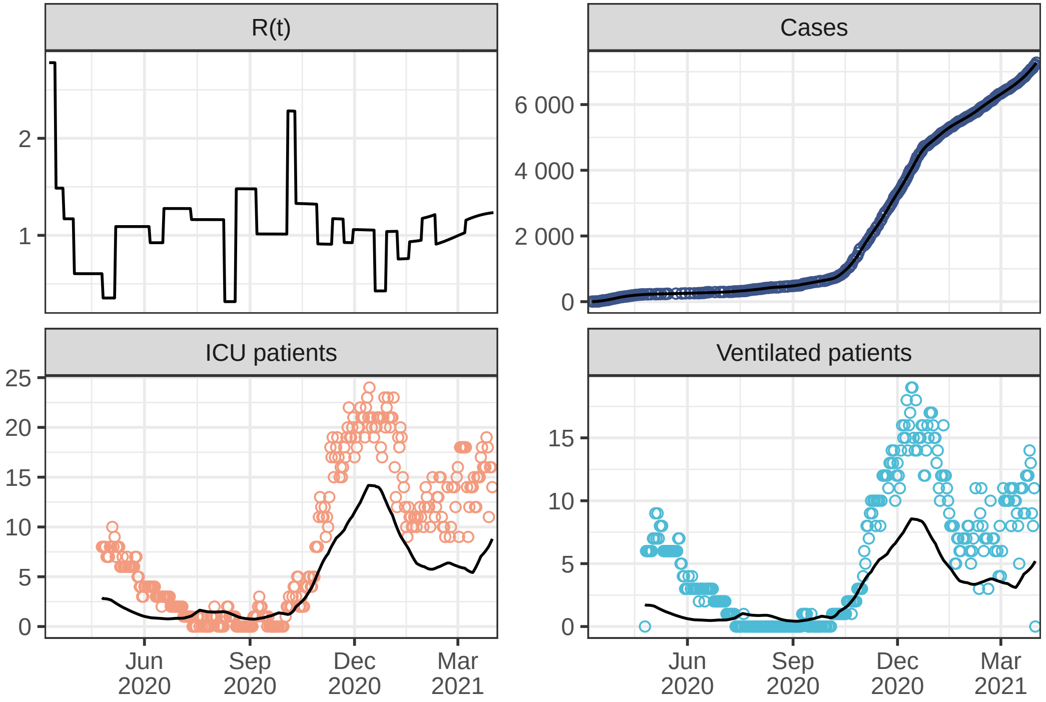

SK Speyer

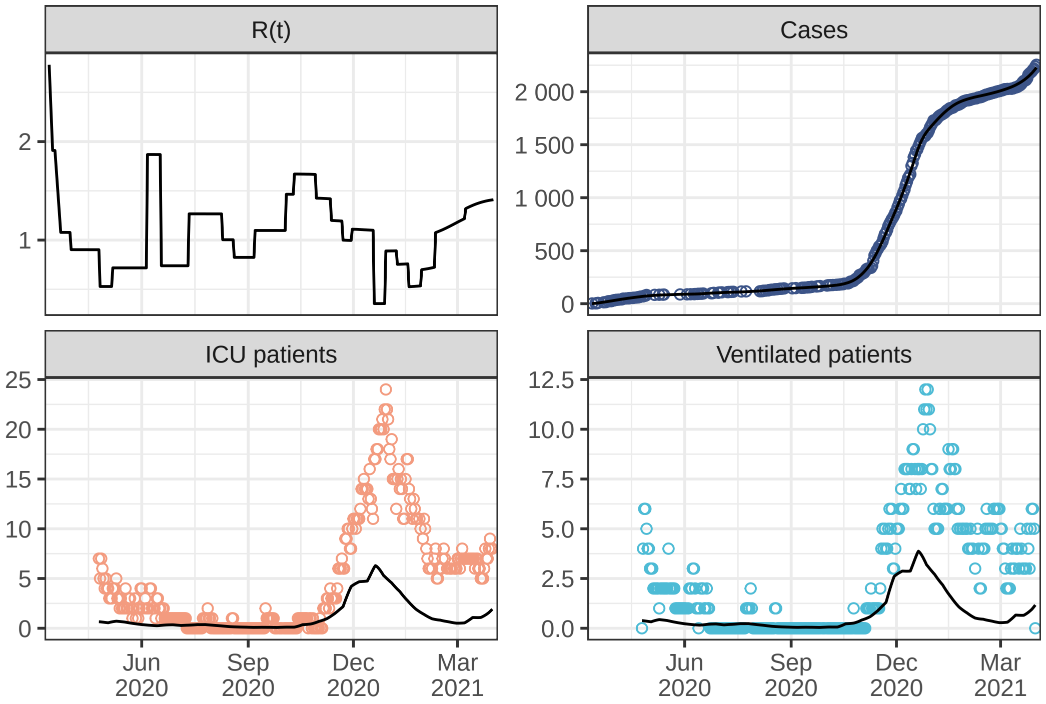

SK Straubing

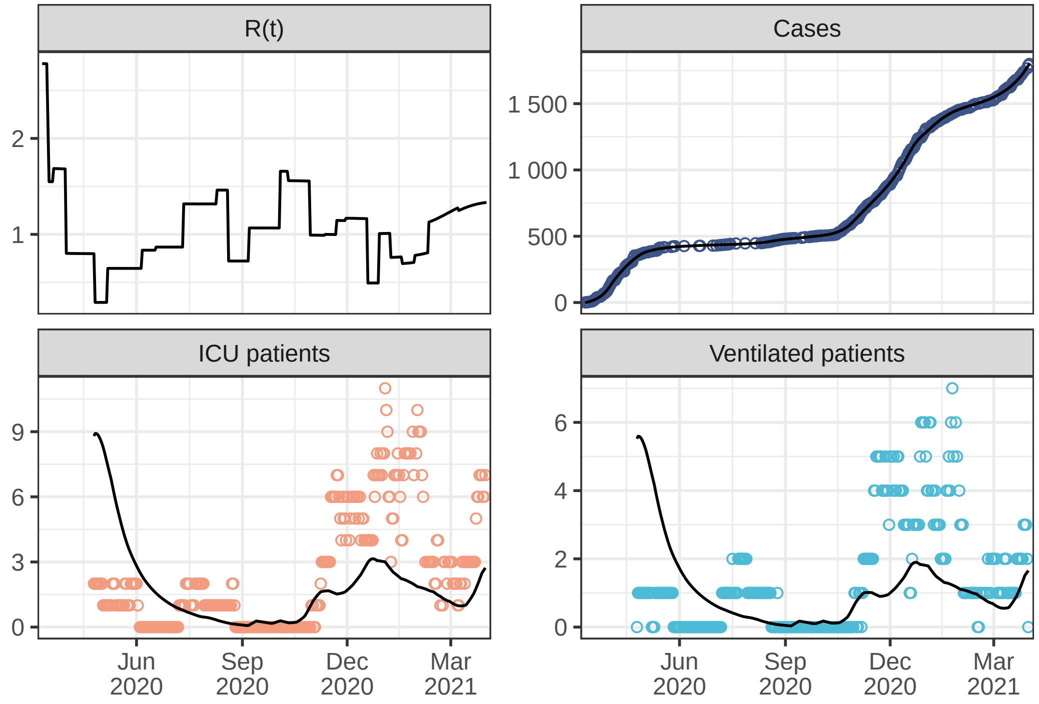

SK Stuttgart

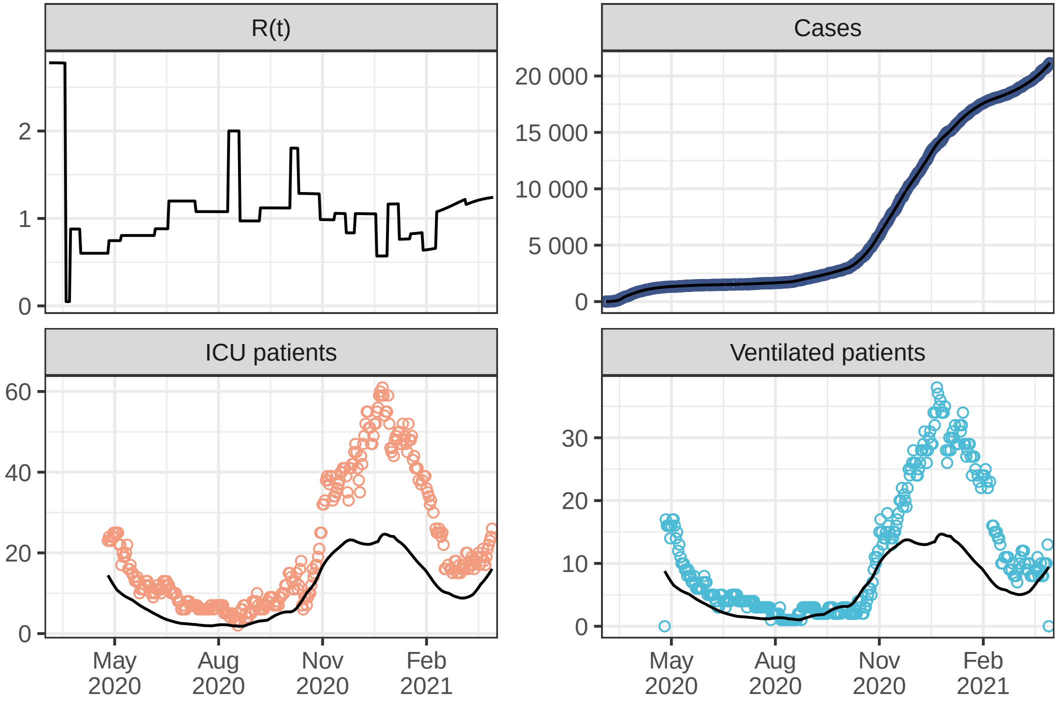

SK Suhl

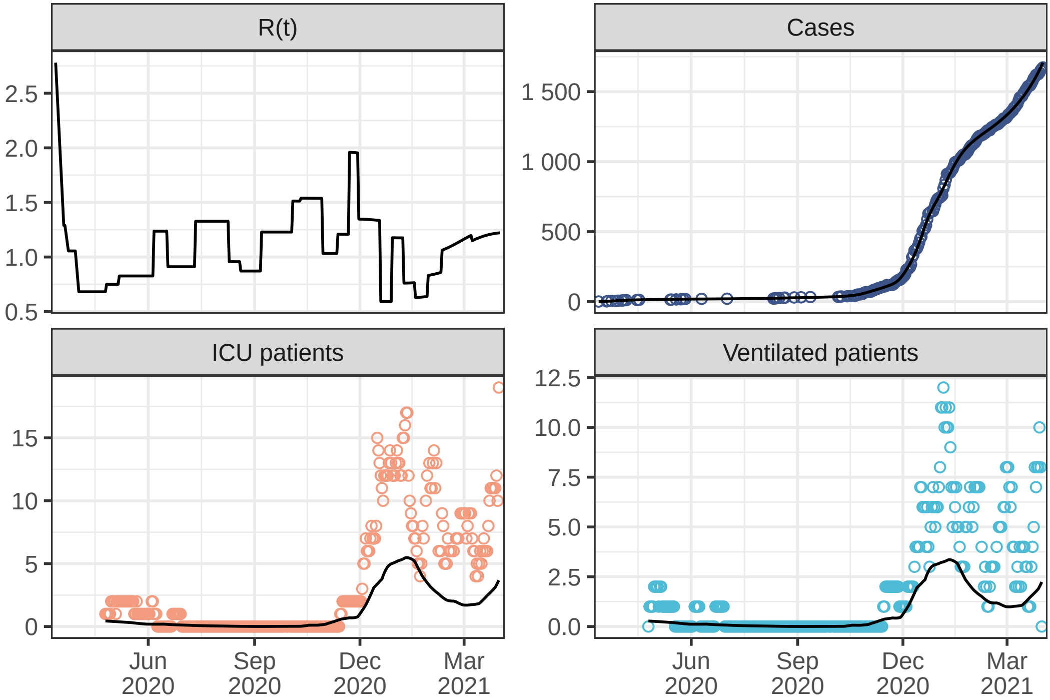

SK Trier

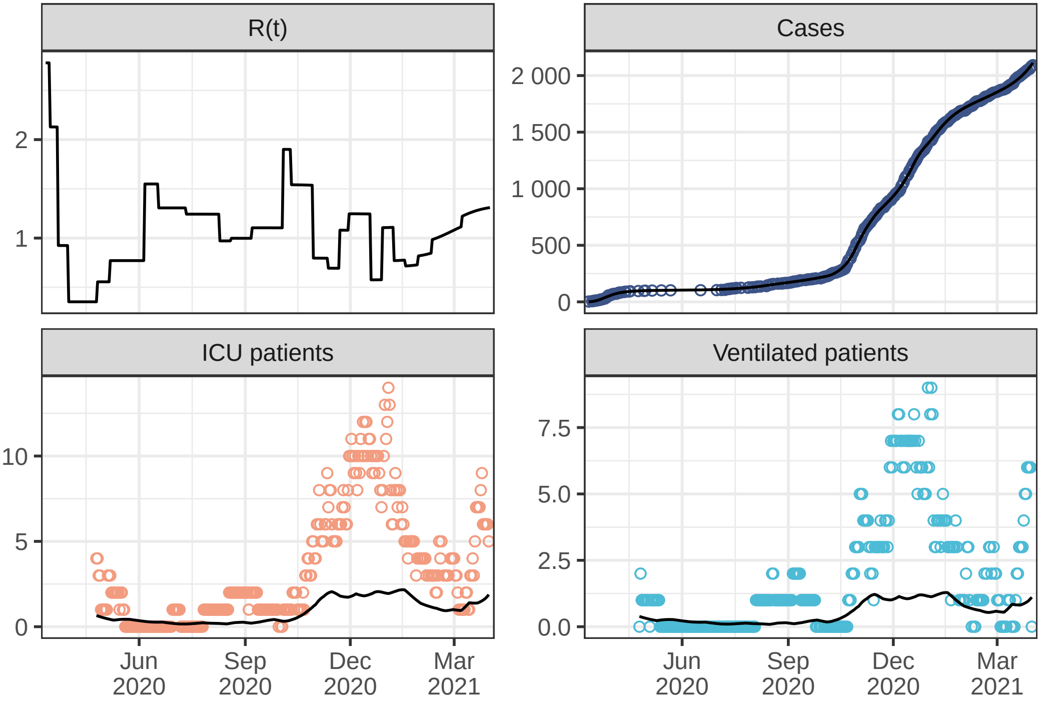

SK Ulm

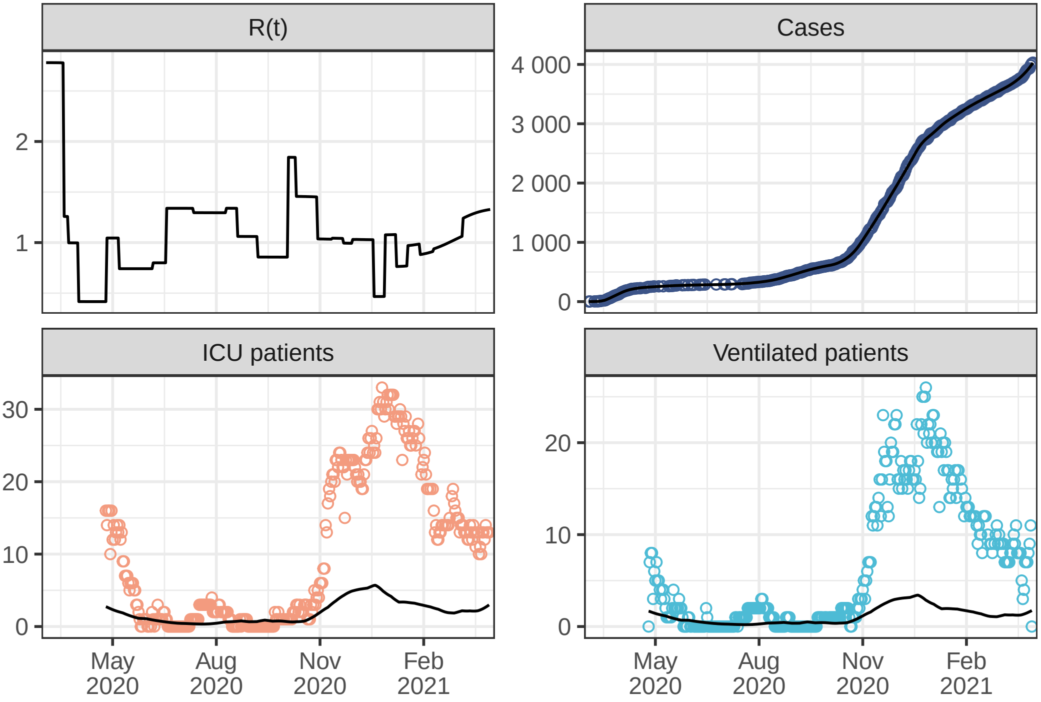

SK Weiden i.d.OPf.

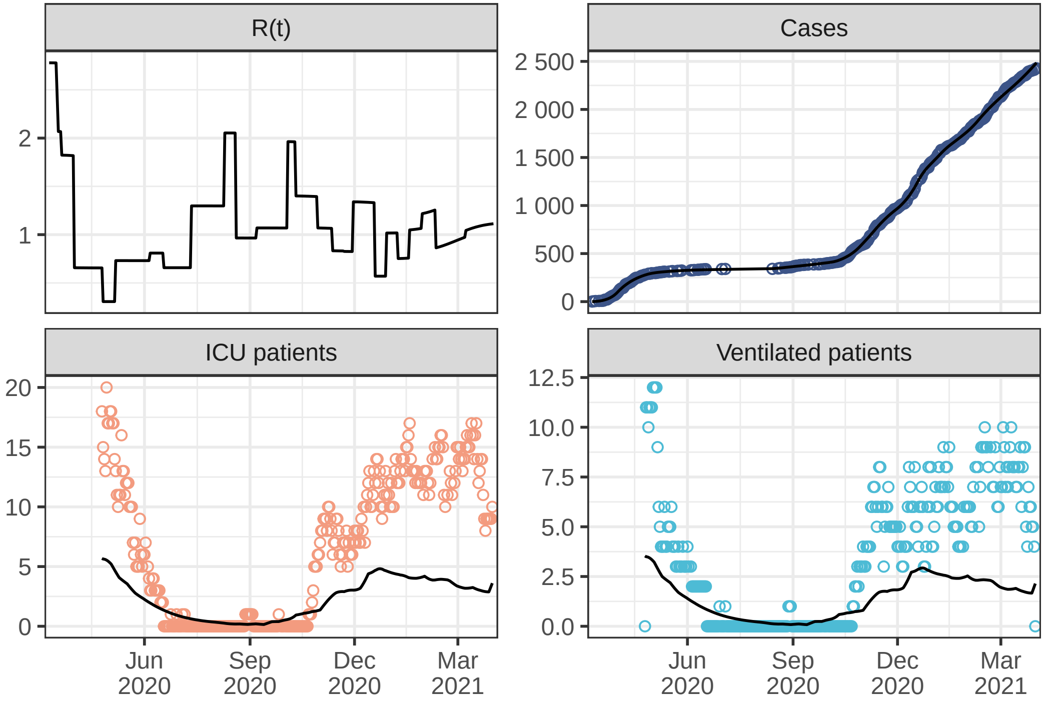

SK Weimar

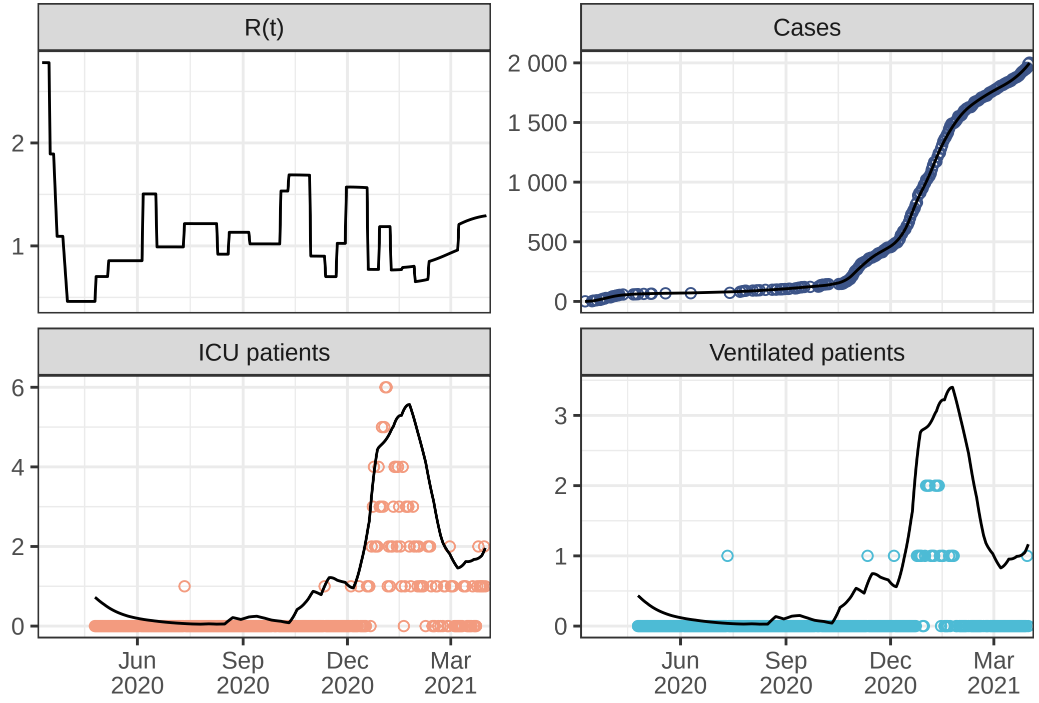

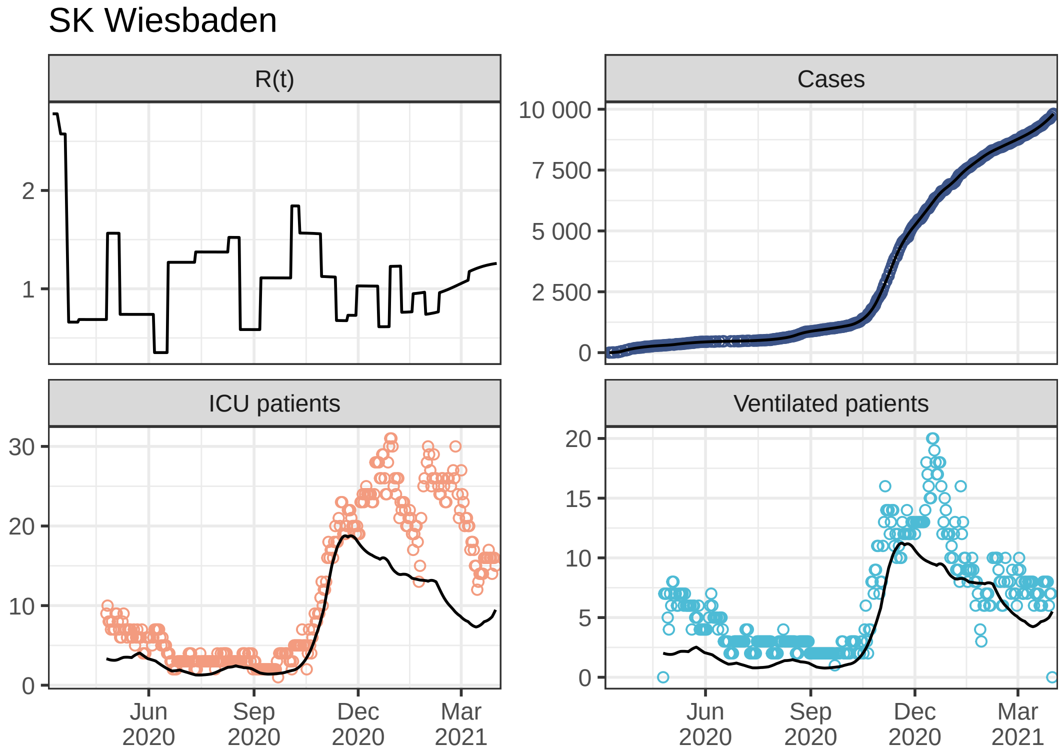
SK Wiesbaden
R(t)
Cases
ICU patients
Ventilated patients
2
1
10 000
7 500
5 000
2 500
0
30
20
10
0
20
15
10
5
0
Jun 2020
Sep 2020
Dec 2020
Mar 2021

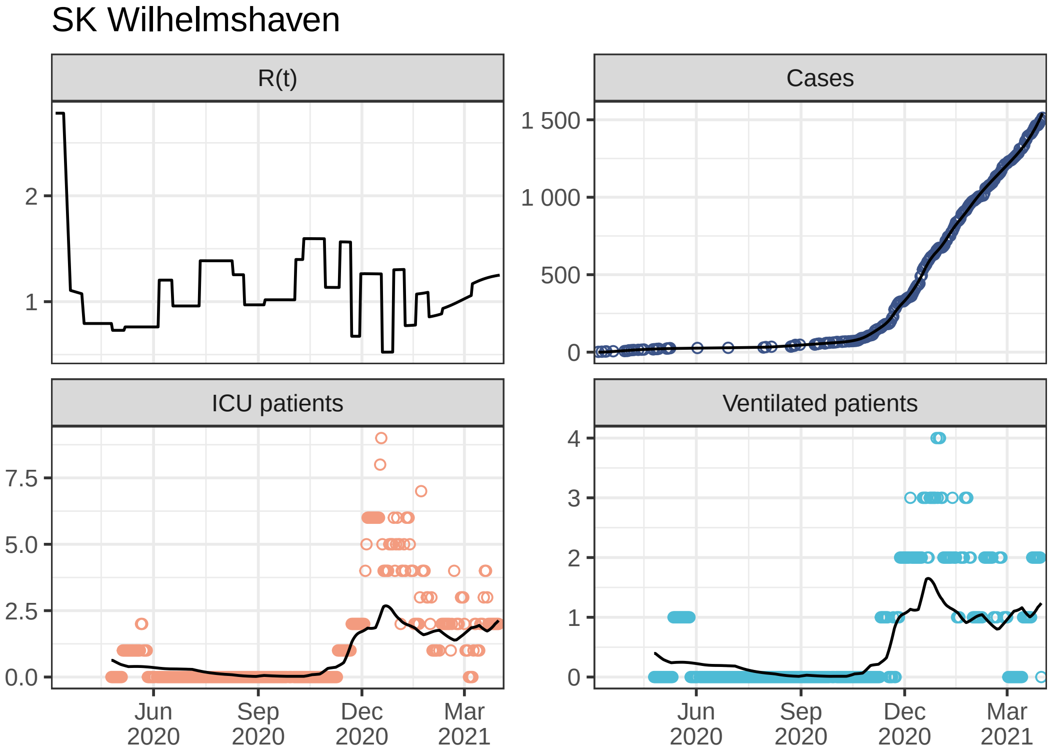
SK Wilhelmshaven
R(t)
Cases
ICU patients
Ventilated patients
2
1
1 500
1 000
500
0
7.5
5.0
2.5
0.0
4
3
2
1
0
Jun 2020
Sep 2020
Dec 2020
Mar 2021

SK Wolfsburg

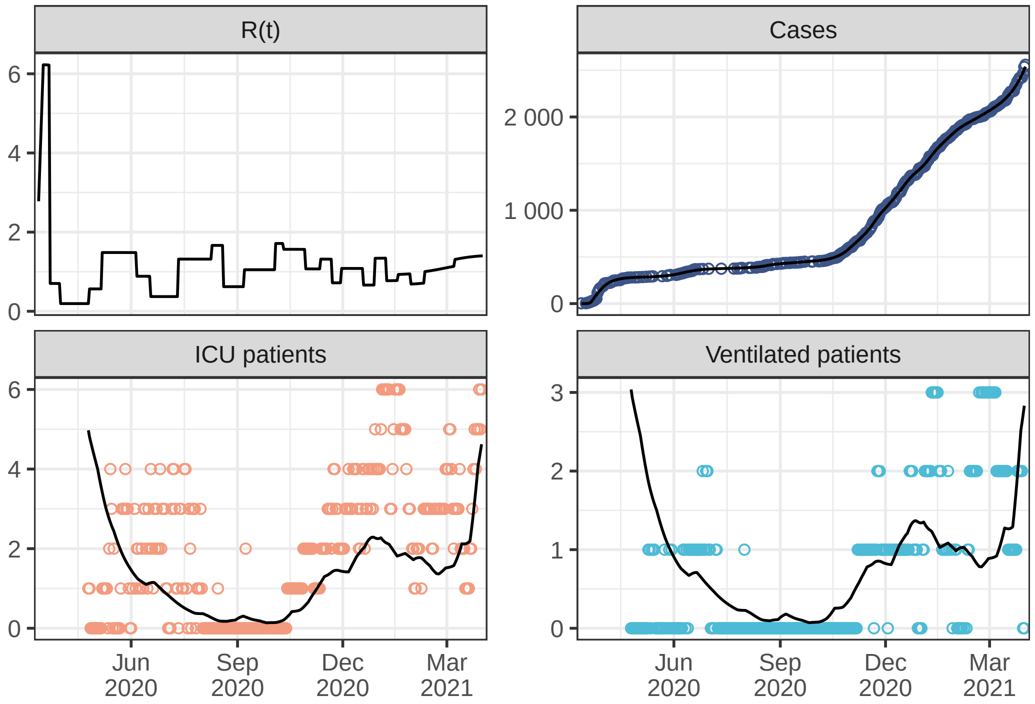

SK Worms

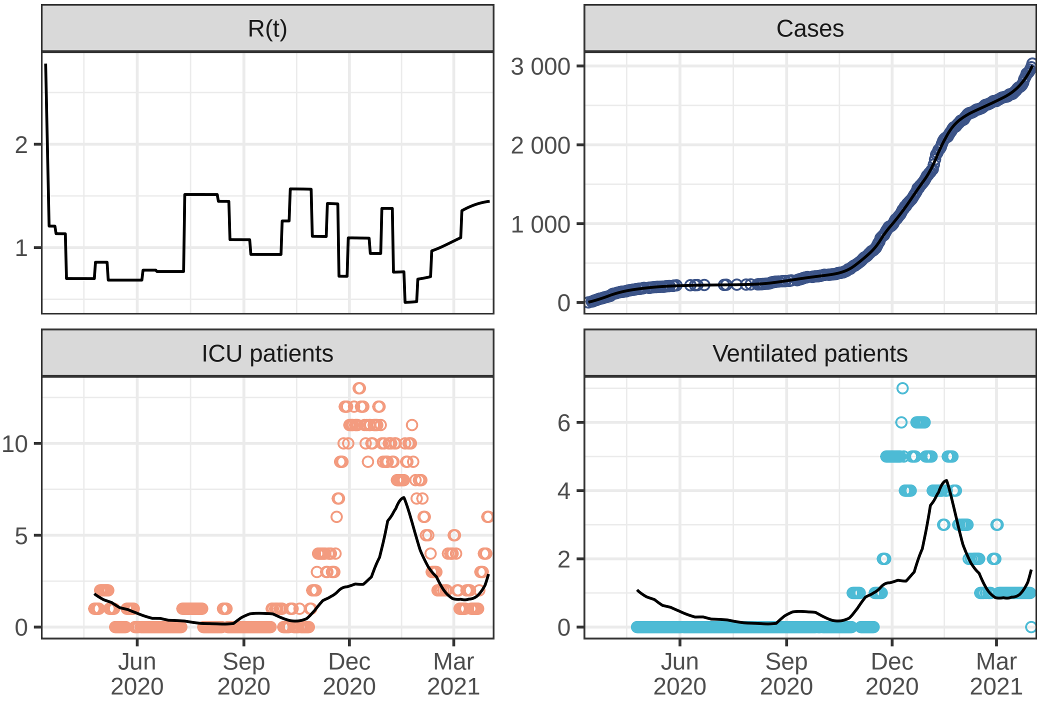

SK Wuppertal

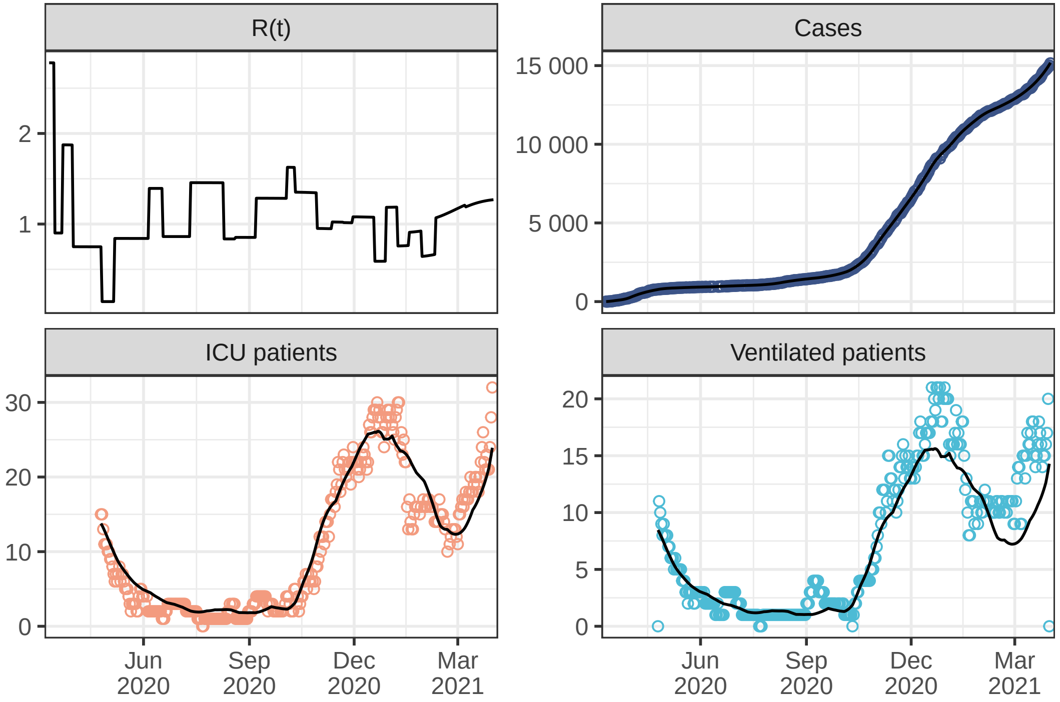

SK Würzburg

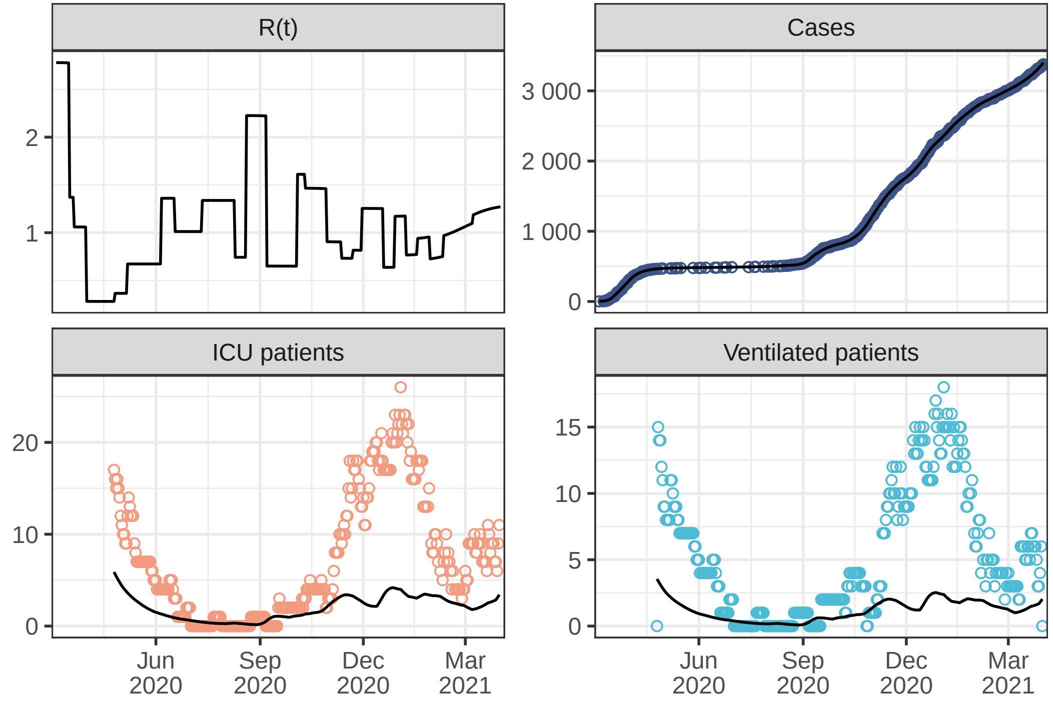

SK Zweibrücken

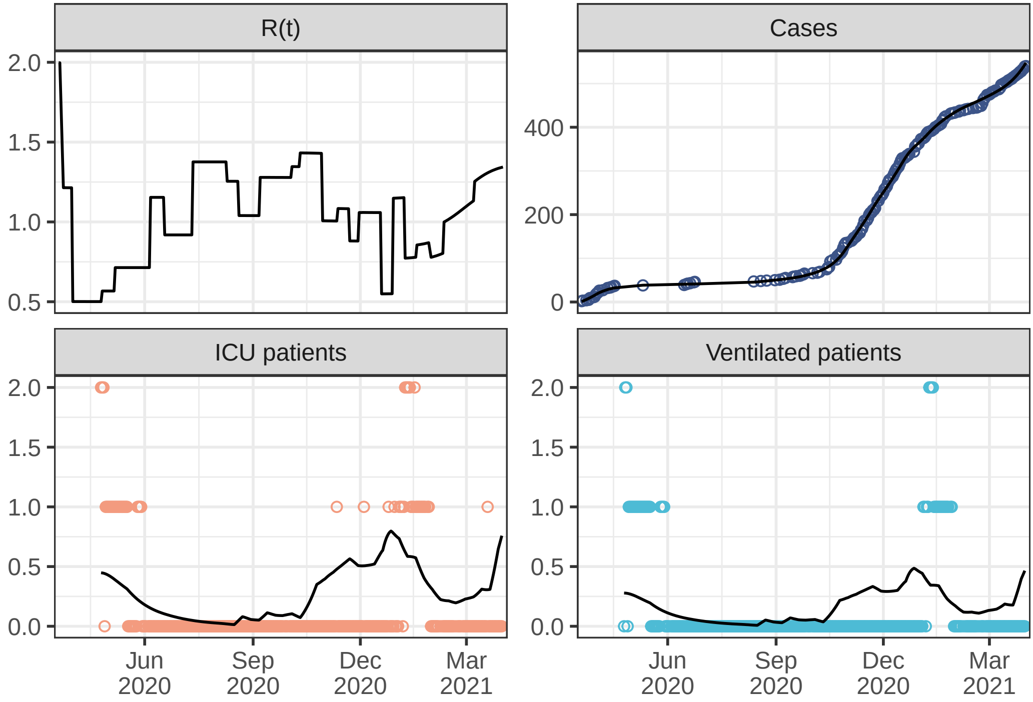

StadtRegion Aachen

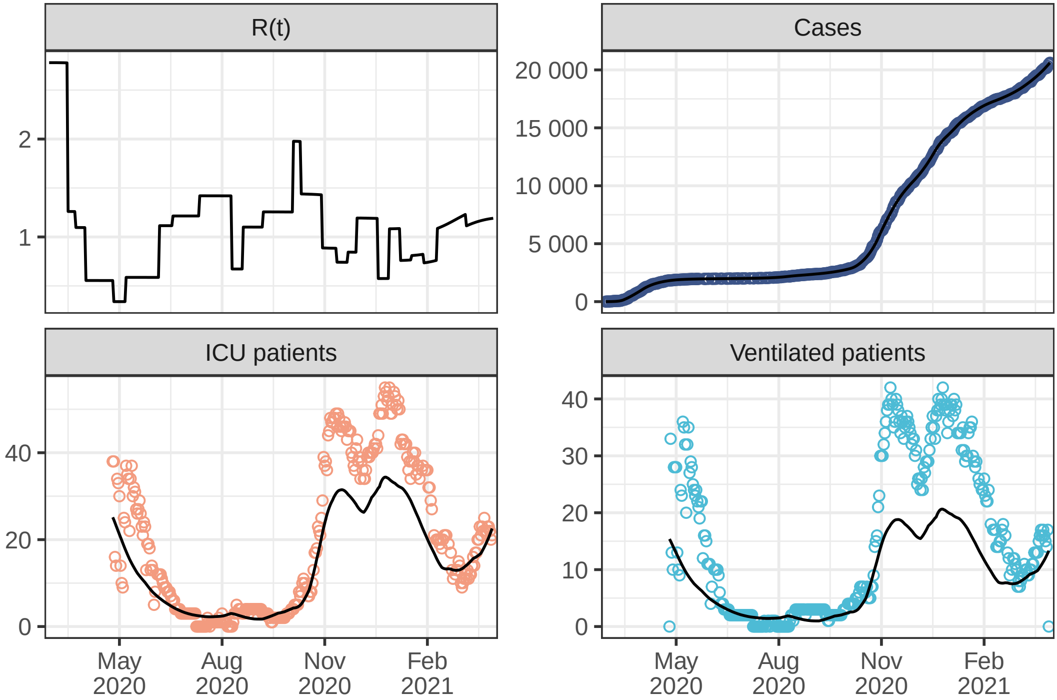

Supplement: Supplementary file 1 [file viruses-14-02114-s001.zip › Figure S2.pdf]
