# Supplementary material for: Model-Based Analysis of SARS-CoV-2 Infections, Hospitalization and Outcome in Germany, the Federal States and Districts
Source: Viruses. 2022 Sep 24;14(10):2114. doi: 10.3390/v14102114 (PMC9607468; doi:10.3390/v14102114)
Supplement: Supplementary file 1 [file viruses-14-02114-s001.zip › Tables S1-S4.pdf]

Attached as PDF: Figure S1.pdf

Figure S1. Descriptive performance plots for Germany and all federal states. Points: observations, lines: model simulations. Information about the number of inpatients was not available for Germany and several federal states.

Attached as PDF: Figure S2.pdf

Figure S2. Descriptive performance plots for German districts (NUTS-3). Points: observations, lines: model simulations. Information about the number of ICU and ventilated patients was not available for 4 districts.

Attached as .mod-file: ModelFile S1.mod

Model File S1. NONMEM model file of the infectiousness model

Attached as .mod-file: ModelFile S2. mod

Model File S2. NONMEM model file of the full model

Supplementary Table S1. Data Sources

Area	Data	Source
Germany and federal states	Cases, recoveries, fatalities	Berliner Morgenpost [18]
Germany and federal states	ICU with and without ventilation	DIVI Intensivregister [33]
Germany and federal states	Number of tests and positive rates	RKI [19]
German districts	Cases with age groups and sex distribution	RKI [19]
German districts	ICU with and without ventilation	RKI documentation of DIVI data [34]
Bavaria	Occupied hospital beds	Bavarian government [20]
Berlin	Occupied hospital beds and daily hospitalizations	Berlin government [21]
Brandenburg	Occupied hospital beds	Brandenburg government [25]
Bremen	Occupied hospital beds	Bremen government [26]
Hamburg	Occupied hospital beds	Hamburg government [27]
Hesse	Occupied hospital beds and daily hospitalizations	Hesse government, via email
Lower Saxony	Occupied hospital beds	Lower Saxony government [28]
Mecklenburg-Vorpommern	Occupied hospital beds and daily hospitalizations	Mecklenburg-Vorpommern government [29]
North Rhine-Westphalia	Occupied hospital beds and daily hospitalizations	North Rhine-Westphalia government [30]

Rhineland-Palatinate	Occupied hospital beds and daily hospitalizations	Rhineland-Palatinate government, via email
Rhineland-Palatinate	Daily hospitalizations	Rhineland-Palatinate government [31]
Saarland	Occupied hospital beds	Saarland government, via email
Saxony	Occupied hospital beds	Saxony government [32]
Saxony-Anhalt	Daily hospitalizations	Saxony-Anhalt government [22]
Schleswig-Holstein	Occupied hospital beds and daily hospitalizations	Christian-Albrechts-Universität zu Kiel [23]
Thuringia	Daily hospitalizations	Thuringia government [24]

Table S2. Summary of the clinical database. Age is summarized as median and interquartile range.

		**Total inpatients**	**General ward patients**	**ICU patients**	**ICU patients not ventilated**	**ICU patients ventilated**
N		28847	23612	5235	1727	3508
Age [years]		73 (57-83)	73 (56-83)	72 (61-80)	71 (57-81)	73 (63-80)
Sex	Male	15300 (53%)	11869 (50%)	3431 (66%)	1078 (62%)	2353 (67%)
	Female	13547 (47%)	11743 (50%)	1804 (34%)	649 (38%)	1155 (33%)
Fatalities		6913 (24%)	4204 (18%)	2709 (52%)	412 (24%)	2297 (65%)

Table S3. Changes in infectiousness (Rt) according to NPIs and model estimated changepoints (CP).

**Date of CP**	**NPI/Explanation for change in infectiousness**	**Model estimated CP**	**Rt Germany (lowest – highest in federal states)**	**Relative Change**	**Inter-state variability [%CV]**
Initial R	No NPIs in action	-	2.78	-	-
16-19 Mar 2020	School closure (differs between federal states)	No	1.92 (1.37-2.79)	-31%	18.7%
21-23 Mar 2020	Curfew or restraining order (differs between federal states)	No	1.1 (0.70-1.83)	-43%	24.1%
1 Apr 2020	No distinguishable NPIs, might be accountable to a raised awareness in population	Yes	0.64 (0.41-1.13)	-42%	25.5%
26 Apr 2020	Mandatory face masks in public buildings and public transport becoming effective between 22 and 29 April (differs between federal states); the average Rt in Germany is stable, however, there are significant changes between the federal state Rts	Yes	0.64 (0.29-1.05)	0%	36.7%
7 May 2020	Lifting of some restrictions on 6 May	Yes	0.72 (0.50-1.07)	+13%	21.7%

6 Jun 2020	Local hotspots with many infected individuals while the total number of weekly cases is low leading to local short term changes of Rt	Yes	1.08 (0.55-2.01)	+51%	40.6%
18 Jun 2020	Local hotspots with many infected individuals while the total number of weekly cases is low leading to local short term changes of Rt	Yes	0.86 (0.58-1.35)	-21%	27.2%
12 Jul 2020	Many infections among travelers returning to Germany	Yes	1.33 (1.19-1.58)	+55%	6.9%
10 Aug 2020	Obligatory PCR test for travelers since 8 August	Yes	1.05 (0.57-1.70)	-21%	34.1%
20 Aug 2020	Various local increases or liftings of restrictions over the course of August -September	Yes	0.95 (0.68-1.33)	-10%	31.6%
7 Sep 2020	Various local increases or liftings of restrictions over the course of August -September	Yes	1.25 (0.99-1.50)	+32%	10.2%
4 Oct 2020	-	Yes	1.51 (1.07-2.18)	+21%	18.3%
12 Oct 2020	14 October new local resolution for counties with high incidence	Yes	1.55 (1.34-1.81)	+3%	6.3%
28 Oct 2020	Resolution of “Lockdown light”	Yes	1.12 (0.92-1.44)	-28%	11.3%
6 Nov 2020	Resolutions of “Lockdown light” effective on 2 November	Yes	1.00 (0.75-1.31)	-11%	12.6%
19 Nov 2020	New infection protection law resoluted on 18 November	Yes	0.98 (0.75-1.36)	-2%	15.1%
30 Nov 2020	-	Yes	1.2 (1.1-1.36)	+25%	6.2%
18 Dec 2020	Further lockdown restrictions in force at 16 December	Yes	0.67 (0.53-0.89)	-45%	14.6%
28 Dec 2020	Increase of personal contacts and delay in reporting during the Christmas holidays	Yes	1.22 (1.08-1.45)	83%	6.3%
8 Jan 2021	Further lockdown restrictions in force an 11 January	Yes	0.80 (0.64-0.95)	-35%	9.3%
22 Jan 2021	Differing reopening of schools between federal states, 19 January: expansion of federal lockdown until 14 February	Yes	0.82 (0.48-1.42)	+4%	29%
29 Jan 2021	Travel restrictions from countries with high incidence of VOC B.1.1.7 at 30 January	Yes	0.76 (0.67-0.87)	-8%	8.6%
11 Feb 2021	Federal government expands lockdown until 7 March; only some restrictions are lifted	Yes	0.93 (0.82-0.99)	+23%	6.1%
8 Mar 2021	Lifting of several restrictions by the German federal government	No	0.99 (0.94-1.04)	+6%	3.9%

Table S4. Fractions of confirmed cases hospitalized, treated at ICU, ventilated and death rates as functions of age (a) and sex (s) as extracted from the clinical database.

	Model function	Age group [years]	Female (F) [%]	Male (M) [%]
Fraction hospitalized	fh(a, s)	0 to 4	3.02	3.70
		5 to 14	0.90	0.90
		15 to 35	2.38	1.54
		35 to 59	4.45	7.45
		60 to 79	20.6	29.1
		80+	29.6	50.3
Fraction treated at ICU of inpatients	fi(a, s)	0 to 4	6.05	23.7
		5 to 14	5.87	19.6
		15 to 35	9.64	22.2
		35 to 59	21.4	35.7
		60 to 79	35.3	54.1
		80+	21.4	35.1
Fraction ventilated of ICU patients	fv(a, s)	0 to 4	30.8	30.8
		5 to 14	44.4	44.4
		15 to 35	46.3	46.3
		35 to 59	60.0	60.0
		60 to 79	71.9	71.9
		80+	66.6	66.6
Fraction death (general ward)	fdh(a, s)	0 to 34	0	0
		35 to 59	1.25	1.25
		60 to 79	10.1	14.7
		80+	33.4	41.4
Fraction death (ICU not ventilated)	fdi(a, s)	0 to 34	0	0
		35 to 59	4.53	4.53
		60 to 79	19.4	19.4
		80+	33.4	33.4
Fraction death (ICU ventilated)	fdv(a, s)	0 to 4	0	0
		5 to 14	25.0	25.0
		15 to 35	18.0	18.0
		35 to 59	37.2	37.2
		60 to 79	65.3	65.3
		80+	84.1	84.1
Fraction death (outpatients)	fda(a, s)	0 to 59	0	0
		60 to 79	1.72	2.73
		80+	18.3	22.6

References

18. Corona Zahlen Aktuell: Karte Für Deutschland + Weltweit. Available online: https://interaktiv.morgenpost.de/corona-virus-karte-infektionen-deutschland-weltweit/ (accessed on 4 August 2022).
19. SurvStat@RKI 2.0. Available online: https://survstat.rki.de/Content/Query/Create.aspx (accessed on 4 August 2022).

20. Informationen Zu Corona: Häufige Fragen-Bayerisches Staatsministerium Des Innern, Für Sport Und Integration. Available online: https://www.stmi.bayern.de/miniwebs/coronavirus/lage/index.php (accessed on 4 August 2022).
21. COVID-19 Berlin. Available online: https://www.berlin.de/corona/lagebericht/desktop/corona.html#stationäre-behandlung (accessed on 4 August 2022).
22. Pressemitteilungen|Ministerium Für Soziales, Gesundheit, Integration Und Verbraucherschutz. Available online: https://msgiv.brandenburg.de/msgiv/de/presse/pressemitteilungen/ (accessed on 4 August 2022).
23. Corona-Fallzahlen-Die Senatorin Für Gesundheit, Frauen Und Verbraucherschutz. Available online: https://www.gesundheit.bremen.de/corona/zahlen/corona-fallzahlen-37649 (accessed on 4 August 2022).
24. Corona: Zahlen, Fälle, Statistik von COVID-19-Hamburg.De. Available online: https://www.hamburg.de/corona-zahlen/ (accessed on 4 August 2022).
25. Presseinformationen|Nds. Ministerium Für Soziales, Gesundheit Und Gleichstellung. Available online: https://www.ms.niedersachsen.de/startseite/service_kontakt/presseinformationen/ (accessed on 4 August 2022).
26. Daten Zur Corona-Pandemie-LAGuS. Available online: https://www.lagus.mv-regierung.de/Gesundheit/InfektionsschutzPraevention/Daten-Corona-Pandemie (accessed on 4 August 2022).
27. NRW-Dashboard Zur Corona-Pandemie. Available online: https://www.giscloud.nrw.de/corona-dashboard.html (accessed on 4 August 2022).
28. Coronavirus SARS-CoV-2: Aktuelle Fallzahlen Für Rheinland-Pfalz. Available online: https://lua.rlp.de/de/presse/detail/news/News/detail/coronavirus-sars-cov-2-aktuelle-fallzahlen-fuer-rheinland-pfalz/ (accessed on 4 August 2022).
29. Infektionsfälle in Sachsen-Coronavirus in Sachsen-Sachsen.De. Available online: https://www.coronavirus.sachsen.de/infektionsfaelle-in-sachsen-4151.html (accessed on 4 August 2022).
30. Pressemitteilungen. Available online: https://ms.sachsen-anhalt.de/presse/pressemitteilungen/ (accessed on 4 August 2022).
31. COVID-19—Institut Für Infektionsmedizin. Available online: https://www.infmed.uni-kiel.de/de/epidemiologie/covid-19 (accessed on 4 August 2022).
32. TMASGFF: Infektionslage. Available online: https://www.tmasgff.de/covid-19/fallzahlen (accessed on 4 August 2022).
33. DIVI Intensivregister. Available online: https://www.intensivregister.de/#/aktuelle-lage/laendertabelle (accessed on 4 August 2022).
34. Tagesdaten-CSV Aus Dem DIVI-Intensivregister. Available online: https://edoc.rki.de/ (accessed on 4 August 2022).
